# Catalytic Asymmetric Carbosilylation of Methyl Propiolate with Bis-silyl Ketene Acetals

**DOI:** 10.1021/jacs.5c17310

**Published:** 2025-12-22

**Authors:** Chendan Zhu, Benjamin Mitschke, Benjamin List

**Affiliations:** 28314 Max-Planck-Institut für Kohlenforschung, Kaiser-Wilhelm-Platz 1, 45470 Mülheim an der Ruhr, Germany

## Abstract

We report a catalytic asymmetric silylative α-alkenylation of bis-silyl ketene acetals (bis-SKAs) with methyl propiolate via conjugate addition. In contrast to prior Brønsted-base activation of C–H acidic nucleophiles (β-ketoesters and oxazolones), electrophilic activation of the alkynoate through silylium-asymmetric counteranion-directed catalysis (Si-ACDC) enables this transformation. DFT studies support a pathway in which silylium-mediated addition forms a substituted alkoxy­(trimethylsilyloxy)­propadiene (silyl propadienone acetal, SPA) that rapidly isomerizes via C-silylation accompanied by an O-desilylation to the α,β-unsaturated α-silyl ester with high stereocontrol. The reaction shows a broad scope with uniformly excellent enantio- and diastereoselectivities, and we demonstrate several downstream modifications of the products.

## Graphical abstract

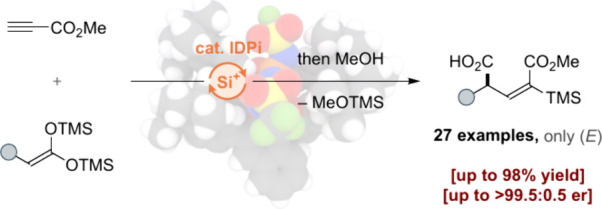

The conjugate addition of nucleophiles to α,β-unsaturated electrophiles is a cornerstone of chemical synthesis and has been extensively developed across catalytic asymmetric platforms.^1–3^ For α,β-unsaturated esters (alkenoates), two activation modes predominate: nucleophile activation by deprotonation and electrophile polarization by Lewis-acid coordination. Despite advances in catalytic asymmetric Michael additions to alkenoates, their alkynoate counterparts remain conspicuously underdeveloped (Figure 1A). To date, conjugate additions of β-diketones^4^ and β-ketoesters,^5–7^ oxazolones,^8,9^ oxindoles,^10^ butenolides,^11^ and related pronucleophiles have been accomplished exclusively via nucleophile activation.^12,13^ In contrast, electrophilic activation of alkynoates is exceedingly rare,^14–18^ and yet no general strategy for asymmetric conjugate additions has emerged. This gap likely reflects two intrinsic challenges: 1) the sp-hybridized, linear alkynoate places the electrophilic β-carbon farther from the chiral environment, undermining stereocontrol; and 2) the α,β-unsaturated ester product is prone to overaddition or oligomerization, especially under nucleophile excess.

**Figure 1:**
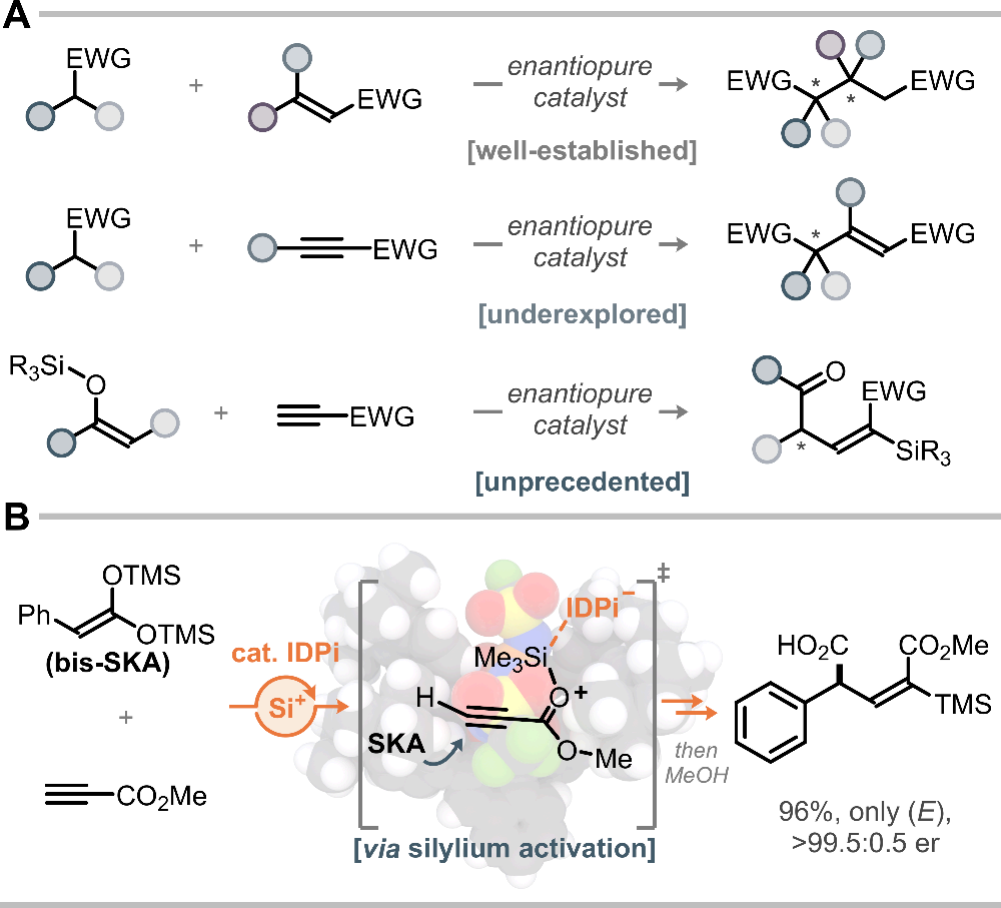
(A) Overview of available methods for catalytic asymmetric conjugate additions to alkenoates and alkynoates. (B) This work: catalytic asymmetric conjugate addition of bis-silyl ketene acetals to alkynoates via Si-ACDC.

Building on our Si-ACDC aminomethylation of bis-SKAs to access highly enantioenriched β-amino acids,^19^ and drawing on our expertise in catalytic asymmetric Mukaiyama–Michael reactions,^20,21^ we hypothesized that silylated imidodiphosphorimidate (IDPi) combining exceptional Lewis acidity with a confined active site could surmount the hurdles of electrophilic alkynoate activation.^22,23^ Specifically, we anticipated that the pronounced IDPi acidity would confer powerful activation of the alkynoate substrate, while its precisely engineered microenvironment would enable efficient enantioselection at the remote β-carbon and additionally suppress sterically hindered α-silyl-substituted product from oligomerization and overreaction.^21,24^ Here we report an IDPi-catalyzed silylative alkenylation of bis-SKAs that tolerates a wide substrate range and delivers structurally diverse alkenoates with excellent enantio- and diastereoselectivities (Figure 1B).

Optimization began with α-phenyl bis-SKA **1a** and methyl propiolate **2a** as the model system. Common chiral Brønsted acids (CPAs, IDPs, *i*IDPs, DSIs) gave no conversion, whereas the more strongly acidic IDPi catalysts furnished quantitative yield of carbosilylation product **4a** (see Supporting Information).^25^ To improve the enantioselectivity, we varied the 3,3′-aryl substituents on the BINOL-derived IDPi scaffold. Alkyl-substituted phenyl rings at these positions were optimal, providing excellent reactivity and regio- and enantioselectivity (Table 1). Catalysts **3a**–**3c**, bearing 4-methylphenyl, 3-methylphenyl, and 4-*tert*-butylphenyl groups at the 3,3′-positions, delivered exclusively the (*E*)-isomer product with promising enantioselectivity at room temperature (entries 1–3). Catalyst **3d**, with a 3,4-disubstituted phenyl group, afforded slightly higher stereocontrol (entry 4). Increasing steric hindrance at the 3- and 4-positions enhanced enantioselection: catalyst **3e**, bearing bulky alkyl groups at both sites, raised the enantiomeric ratio of **4a** to 93.5:6.5 (entry 5). Cooling to −60 °C further improved this ratio to a superb >99.5:0.5 (entries 6 and 7). Reaction concentration had a minimal effect on yield or selectivity, whereas replacing methyl propiolate with ethyl propiolate drastically reduced reactivity (see Supporting Information). Among solvents, methylcyclohexane gave the highest enantioselectivity (Supporting Information). Interestingly, when we used alkyl silyl ketene acetals (SKAs) instead of bis-SKAs, the desired conjugate addition did not take place (see the Supporting Information).

**Table 1:**
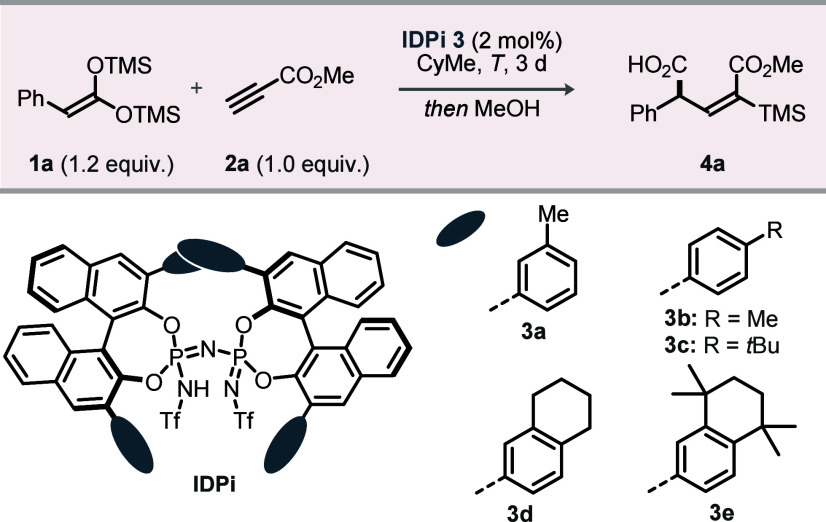
Reaction Development^a^

entry	IDPi	*T* (°C)	yield^b^ (%)	er^c^
1	**3a**	rt	99	60:40
2	**3b**	rt	99	65.5:34.5
3	**3c**	rt	99	75:25
4	**3d**	rt	99	70.5:29.5
5	**3e**	rt	99	93.5:6.5
6	**3e**	0	99	97:3
**7**	**3e**	**–60**	**99**	**>99.5:0.5**

a Reactions were conducted on a 0.02 mmol scale.

b Yields were determined by ^1^H NMR using dibromomethane as internal standard. *E*/*Z* > 50:1.

c Enantiomeric ratios (er) were measured by chiral HPLC. See the SI for further information.

Under the optimized conditions, catalyst **3e** was evaluated across a broad scope (Figure 2). Aryl-substituted bis-SKAs **1a**–**1j** underwent conjugate addition to furnish (*E*)-configured products **4a**–**j** in moderate to good yields and outstanding enantioselectivities (≥99:1 er), irrespective of electronic or steric variation (**4b**–**f**). Substrates containing 3-, 4-, and combined 3,4-disubstitution furnished products **4g**–**h** in >90% yield and >99.5:0.5 er. The method also tolerated hetero- and polyaromatic partners, affording esters **4i**–**j** in good efficiency.

**Figure 2:**
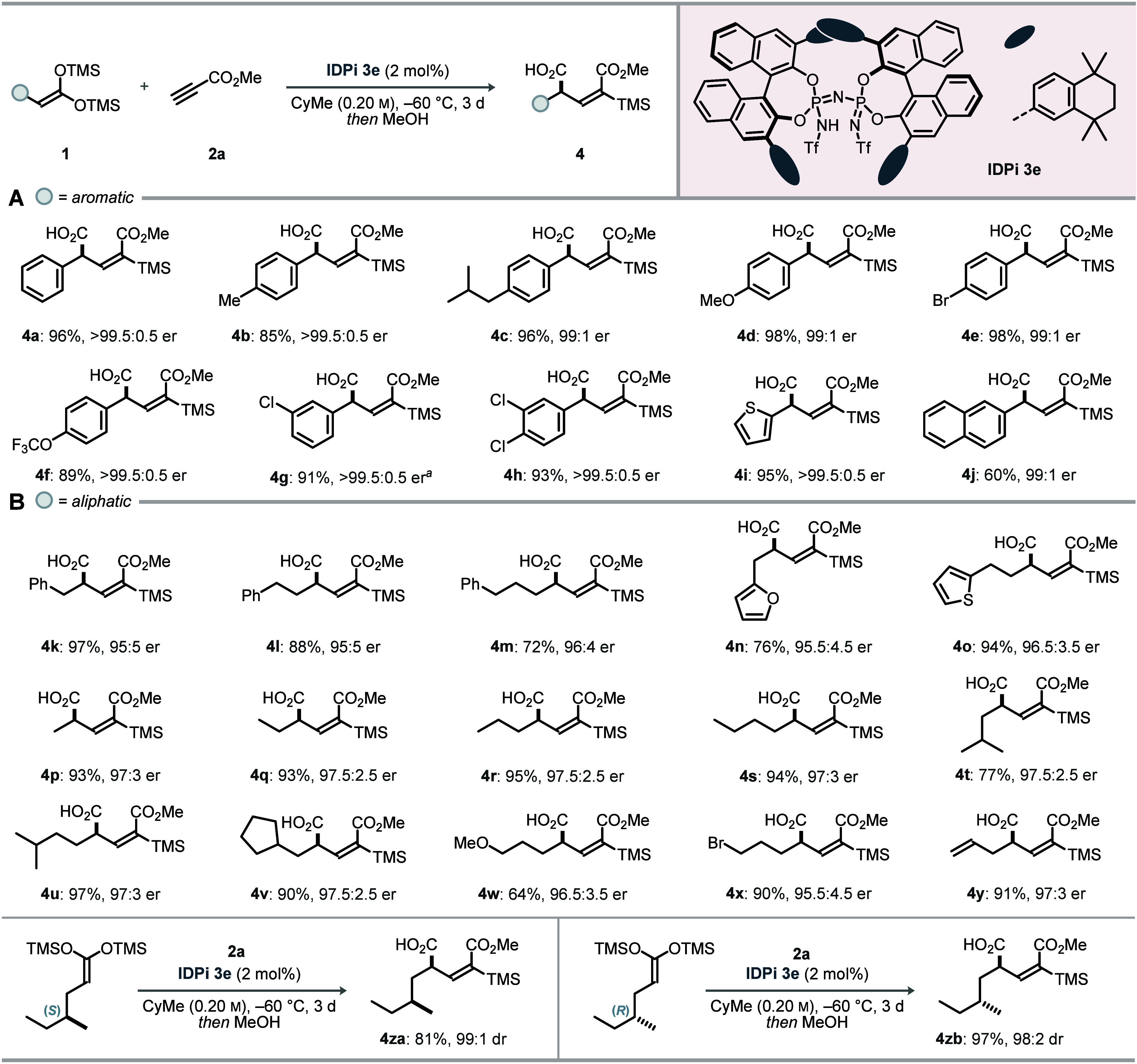
Substrate scope. Reactions were conducted on a 0.2 mmol scale: **1**:**2a** = 1.2:1. Yields were determined after isolation by flash column chromatography, and enantiomeric ratios were measured by chiral HPLC. ^*a*^–40 °C. CyMe, methylcyclohexane.

Varying the methylene tether between the phenyl ring and the bis-SKA (**1k**–**m**) furnished products **4k**–**m** in good yields with high enantioselectivity. Heteroaromatic-tethered substrates **1n**–**o** gave esters **4n**–**o** with comparable efficiency. We next examined the aliphatic bis-SKAs. The shortest alkyl tether, **1p**, coupled with methyl propiolate **2a** to afford **4p** in 93% yield and 97:3 er. Systematic changes in chain length (**4q**–**s**), incorporation of branched (**4t**–**u**) or cyclic (**4v**) frameworks, and installation of methoxy (**4w**), bromo (**4x**), or terminal olefin (**4y**) groups all delivered exclusively (*E*)-configured products in good to excellent yields with excellent enantioselectivities.

Enantiopure bis-SKA **1z** and its enantiomer *ent*-**1z** underwent catalyst-controlled addition to give **4za** and **4zb** in good yields with exceptional diastereocontrol.

To showcase utility, product **4a** was prepared on a multigram scale without a loss of yield or enantiomeric purity and served as a versatile intermediate for downstream transformations (Figure 3A). Triflimide-promoted esterification with alcohols furnished alkyl esters **5a** and **5b** in excellent yields while retaining >99:1 er. A palladium-catalyzed C–H activation on the aryl ring delivered **6** in 78% yield. Epoxidation with *m*CPBA gave a separable 3:1 mixture of epoxides **7a** and **7b**; each diastereomer was isolated in the near-enantiopure form. Iodination with *N*-iodosuccinimide provided vinyl iodide **8**, whereas bromination with bromine afforded a diastereomeric mixture whose ratio could be tuned by lowering the temperature from 25 °C to −78 °C. Finally, catalytic hydrogenation over Pd/C furnished saturated product **10** with >50:1 diastereomeric ratio (dr) and without erosion of enantiomeric excess. Desilylation of hydrogenation product **10** with KF afforded **12** in a quantitative yield. Subsequent global reduction of **10** and **12** with lithium aluminum hydride furnished the corresponding enantioenriched diols **11** and **13**, respectively. The absolute and relative configurations of epoxide **7b** and diol **11** were unambiguously assigned by single-crystal X-ray diffraction.

**Figure 3:**
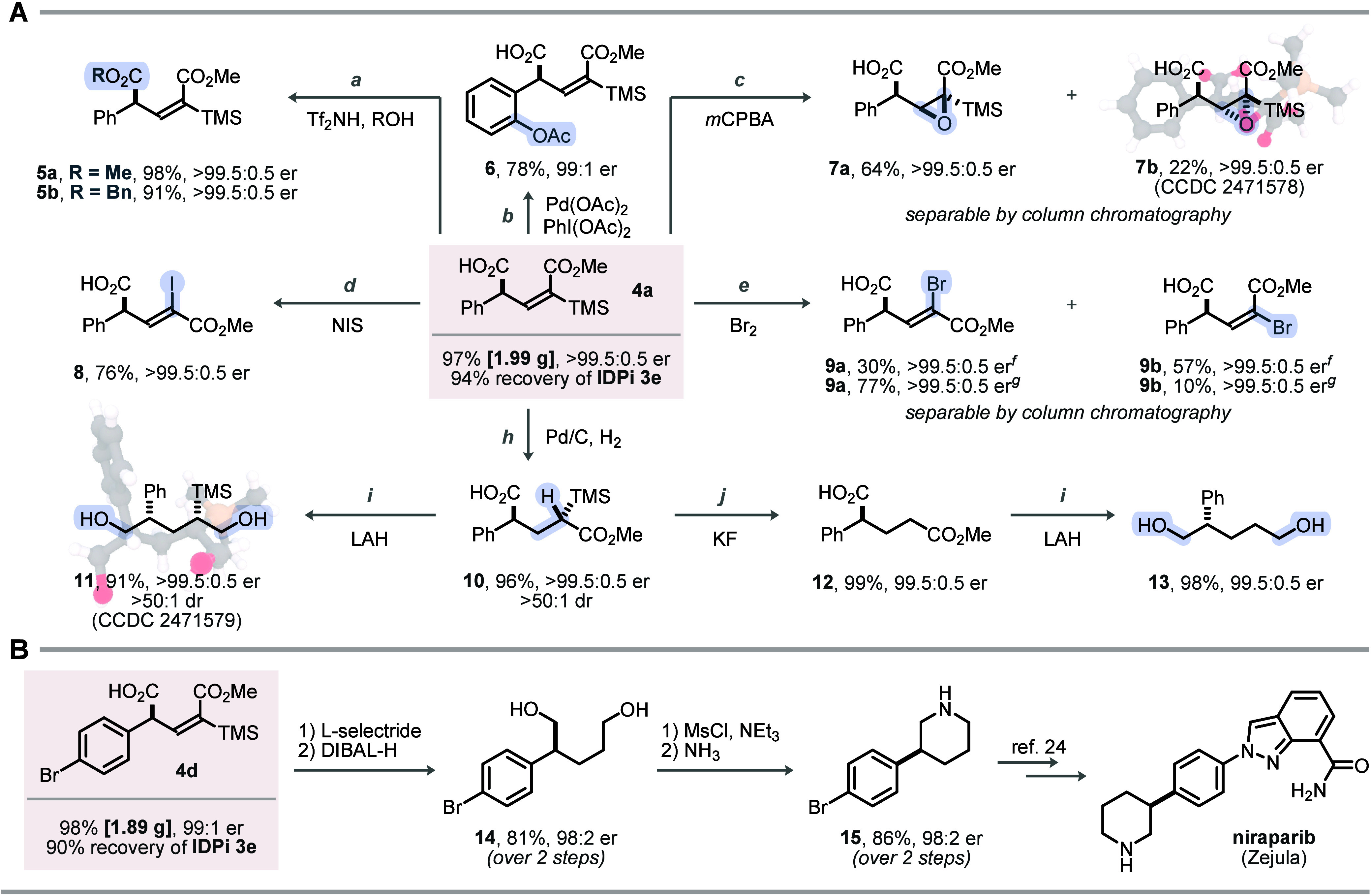
Derivatizations and applications. ^*a*^TfNH_2_, ROH, DCM, rt. ^*b*^Pd­(OAc)_2_, PhI­(OAc)_2_, AcOH, 100 °C. ^*c*^ *m*CPBA, DCM, rt. ^*d*^NIS, HFIP, rt. ^*e*^Br_2_, DCM. ^*f*^ −78 °C. ^*g*^rt. ^*h*^Pd/C, H_2_, MeOH, rt. ^*i*^LAH, THF, 0 °C to rt. ^*j*^KF, MeCN/MeOH, rt. See the Supporting Information for detailed conditions.

Given the broad scope, we developed a concise synthesis of piperidine fragment **15**, a key intermediate en route to the anticancer agent niraparib.^26^ For comparison, the first-generation commercial route delivers niraparib in 11 linear steps (11% overall yield), whereas Merck’s second-generation process proceeds in nine steps with 23–30% overall yield. On a 5 mmol scale, the conjugate addition furnished product **4d** in excellent yield and enantiomeric purity (Figure 3B); catalyst **3e** was recovered in 90% yield. Sequential reduction with l-selectride followed by DIBAL-H delivered diol **14** in an 81% yield. A four-step sequence from diol **14** via methanesulfonylation and ammonia-promoted cyclization assembled piperidine **15** in excellent overall yield without erosion of enantiopurity.

Motivated by the exceptional enantio- and diastereocontrol, we investigated the mechanism of our process. Based on precedents for nonasymmetric nucleophilic additions of bis-SKAs to alkynoates,^27^ we posited SPA intermediates, which introduce an additional layer of complexity through a stereogenic axis. To probe enantioinduction and the network of transient diastereomers, we performed high-level DFT calculations^28–38^ on the conjugate addition between methyl-substituted bis-SKA **1p** and methyl propiolate **2a**.

To map the key features of the potential energy surface, we modeled transition states of the catalytic cycle using the achiral yet strongly acidic bistriflimide (NTf_2_^–^) counteranion (Figure 4A).

**Figure 4:**
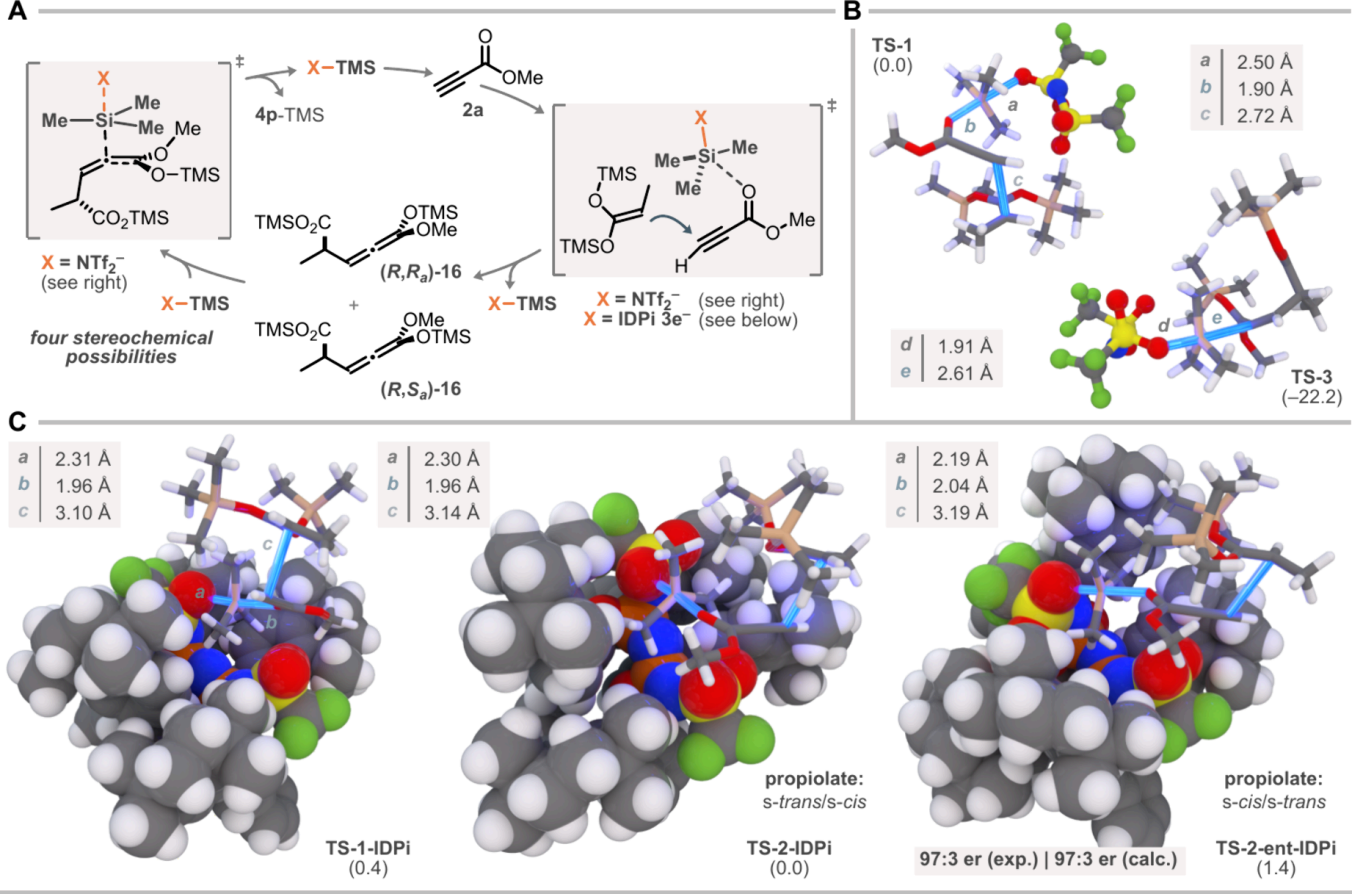
Mechanistic considerations. All calculations were at CPCM­(CyMe)-ωB97M-V/def2-QZVP//r^2^SCAN-3c; 213 K, 1.0 M. (A) Proposed mechanism, with main transition states evaluated by DFT. (B) Comparison of the nucleophilic addition and SPA isomerization transition states (TMS–NTf_2_). (C) Nucleophilic addition transition states (with IDPi **3e**), offering an explanation for the enantioinduction.

At the CPCM­(CyMe)-ωB97M-V/def2-QZVP//r^2^SCAN-3c level, no discrete ion pair formation with methyl propiolate was located; instead, a single transition state (**TS-1**, Figure 4B) emerged, corresponding to a concerted silylation–nucleophilic addition. In **TS-1**, methyl propiolate is activated toward nucleophilic attack and adopts a trigonal-bipyramidal structure in which TMS transfer and C–C bond formation occur synchronously, with NTf_2_^–^ and the propiolate carbon atom in the apical positions.

The NTf_2_^–^ anion binds bidentately to a TMS substituent of bis-SKA (Si···O = 3.41 Å), positioning it to accept the silyl group concomitant with C–C bond formation. This coordination differentiates the two diastereomers of the SPA, giving a 1.5 kcal/mol computational preference for (*R*,*R*_*a*_)-**16** over (*R*,*S*_*a*_)-**16** (see Supporting Information for **TS-2**). The concerted mechanism also explains why larger silyl donors (TBS, TIPS) would encounter greater steric barriers and, presumably, favor more discrete ion pair-like structures.

Under Lewis-acidic silylium conditions, TMS ketene acetals undergo rapid O–Si to C–Si isomerization,^39^ prompting examination of this step. In our model, TMS–NTf_2_ approaches the electron-rich C=C of the ketene acetal in SPA **16**, inducing O–TMS cleavage with concurrent C–Si bond formation to deliver the silyl esters of **4p**. To capture all stereochemical outcomes, we evaluated both diastereomers of **16** and, for each, computed transition states to the (*E*)- and (*Z*)-*C*-silylated isomers, giving four pathways.

Three distinct silyl transfer manifolds onto the electron-rich double bond were located (apical *O*-TMS, equatorial *O*-TMS, apical *N*-TMS), giving 12 transition states in total (see Supporting Information). In every stereochemical scenario, the apical *O*-TMS pathways exhibited the lowest barrier, with **TS-3** (from (*R*,*R*_*a*_)-**16** to (*E*)) being the most favorable (ΔΔ*G* ^‡^ = −22.2 kcal/mol, referenced to TS-1, see Supporting Information for barriers; Figure S13). Ultimately, this manifests as kinetic selectivity to exclusively produce (*E*)-configured alkenoate products. Notably, considering a reversible isomerization regime, the (*E*)-products would be favored likewise (see Supporting Information).

Both the calculated barriers and large energy gap between the initial silylation−addition **TS-1** and isomerization **TS-3** indicate that enantioinduction is established in the first C–C bond forming step, after which rapid, diastereoselective isomerization of the enantioenriched acetal intermediate ensues under IDPi **3e** catalysis.

To test the model under the chiral environment of IDPi **3e**, we computed the four diastereomeric silylation–addition transition states with the enantiopure IDPi anion (Figure 4C).

Remarkably, the confined IDPi counteranion reverses the diastereomeric bias seen with TMS–NTf_2_: now, the (*R*,*S*_*a*_)-isomer (via **TS-2-IDPi**) is favored over (*R*,*R*_*a*_)-**16** (via **TS-1-IDPi**). The competing transition state leading to the epimer at the stereogenic center (**TS-2-ent-IDPi**) lies 1.4 kcal/mol higher than **TS-2-IDPi**, in good agreement with the experimentally measured 97:3 er (observed) vs 97:3 er (calculated).

A distortion–interaction analysis^40^ (see Supporting Information) attributes the penalty to greater structural distortion in **TS-2-ent-IDPi**. Specifically, the propiolate adopts a stereoelectronically disfavored s-*cis*/s-*trans* conformation for silyl transfer, whereas **TS-2-IDPi** proceeds via a more favorable s-*trans*/s-*cis* arrangement. The IDPi anion partially offsets this through a stronger interaction energy in **TS-2-ent-IDPi**. This analysis shows that in TS-2-IDPi-ent the anion forms a closed active site that forces the propiolate into a stereoelectronically unfavorable conformation, maximizing stabilizing intermolecular and potentially noncovalent interactions to compensate for this penalty.

In both pathways, propiolate activation is further stabilized by secondary coordination to a sulfonyl group of the IDPi inner core, and the bulky TMS groups are accommodated by multiple noncovalent (e.g., London dispersion) contacts with the BINOL alkyl substituents.

In summary, we developed a highly enantioselective IDPi **3e**-catalyzed conjugate addition of aromatic and aliphatic bis-SKAs to methyl propiolate. Under the optimized conditions, all substrates furnish (*E*)-configured products in quantitative yield with excellent enantioselectivity. The products undergo diverse downstream modifications without loss of enantiopurity, and the route enables a concise synthesis of enantiopure piperidine fragment **15** en route to niraparib. DFT calculations support an enantiodetermining synchronous silylation–addition followed by rapid, kinetically controlled, diastereoselective isomerization of a SPA.

## Supplementary Material



## Notes

Experimental details and analytical data for all new compounds, crystallographic data for compounds **7b** and **11**, HPLC traces, NMR spectra, and computational studies (PDF)

## #

Laboratorium für Organische Chemie, ETH Zürich, 8093 Zürich, Switzerland
