# Supplementary material for: Catalytic Asymmetric Carbosilylation of Methyl Propiolate with Bis-silyl Ketene Acetals
Source: J Am Chem Soc. 2025 Dec 22;148(1):61–6. doi: 10.1021/jacs.5c17310 (PMC12814322; doi:10.1021/jacs.5c17310)
Supplement: Supplementary file 1 [file ja5c17310_si_003.pdf]

Catalytic Asymmetric Carbosilylation of Methyl Propiolate with bis-Silyl Ketene Acetals

(Supporting Information)

Chendan Zhu, Benjamin Mitschke, and Benjamin List*
Max-Planck-Institut für Kohlenforschung, Kaiser-Wilhelm-Platz 1, 45470 Mülheim an der Ruhr, Germany
*Email: list@kofo.mpg.de

Table of Contents

1. General Information and Instrumentation....S2
2. Synthesis and Characterization of bis-Silyl Ketene Acetals....S4
3. Reaction Development & Optimization....S8
5. Synthesis and Characterization of Catalysts....S11
6. Enantioselective and *E*-Selective Organocatalytic Alkenylation of bis-SKAs....S16
7. Downstream Transformations....S37
8. Synthesis of Piperidine Fragment....S45
9. Crystallographic Data....S48
10. HPLC Traces of the Products....S54
11. ^{1}H , ^{13}C, ^{31}P and ^{19}F NMR spectra of substrates and products....S96
12. Computational Details....S169
13. References....S243

1. General Information and Instrumentation

Chemicals: Unless otherwise indicated, starting materials were obtained from Sigma-Aldrich, ABCR-GmbH, TCI, or Acros Co. Ltd. Commercially available reagents were used without additional purification.

Solvents: Solvents (Et_2O, THF, 1,4-dioxane, cyclohexane, CH_2Cl_2, $CHCl_3$, benzene and toluene) were dried by distillation from an appropriate drying agent in the technical department of the Max-Planck-Institut für Kohlenforschung and received in Schlenk flasks under argon. In addition, more solvents (MTBE, MeCN and methylcyclohexane) were purchased from commercial suppliers and dried over molecular sieves.

Inert Gas: Dry argon was purchased from Air Liquide with >99.5% purity.

Thin Layer Chromatography: Thin-layer chromatography (TLC) was performed using silica gel pre-coated plastic sheets (Polygram SIL G/UV_{254}, 0.2 mm, with fluorescent indicator; Macherey-Nagel) which was visualized with a UV lamp (254 nm) and/or phosphomolybdic acid (PMA), and/or Cerium Ammonium Molybdate (CAM), and/or ninhydrin. PMA stain: PMA (20 g) in EtOH (200 mL). CAM stain: Ammonium molybdate tetrahydrate (2.5 g), Cerium ammonium sulfate dihydrate (1 g) and Sulfuric acid (10 mL) in Water (90 mL). Ninhydrin stain: ninhydrin (1.5 g) in EtOH (200 mL) with AcOH (3 mL).

Column Chromatography: Column chromatography was carried out using Merck silica gel (60 Å, 230−400 mesh, particle size 0.040−0.063 mm) using technical grade solvents. Elution was accelerated using compressed argon. All reported yields, unless otherwise specified, refer to spectroscopically and chromatographically pure compounds.

Nomenclature: Nomenclature follows the suggestions proposed by the computer program ChemDraw (23.1.2.7).

Nuclear Magnetic Resonance Spectroscopy: ^{1}H, ^{13}C, ^{19}F, ^{31}P Nuclear magnetic resonance (NMR) spectra for compound characterization were recorded on Bruker AVIII-500 MHz, NMR spectrometer in a suitable deuterated solvent. The solvent employed and the respective measuring frequency are indicated for each experiment. Chemical shifts are reported with tetramethylsilane (TMS) serving as a universal reference of all nuclides. The resonance multiplicity is described as s (singlet), d (doublet), t (triplet), q (quadruplet), p (pentet), h (heptet), m (multiplet), and br (broad).

All spectra were recorded at 298 K, processed with MestReNova 15.0.0 suits of program, and coupling constants are reported as observed. The residual deuterated solvent signal relative to tetramethylsilane was used as the internal reference in ^{1}H NMR spectra (e.g. $CDCl_3$ = 7.26 ppm, CD_2Cl_2 = 5.32 ppm). Signals are reported as follows: chemical shift δ in ppm (multiplicity, coupling constant *J* in Hz, number of protons). All X-nuclei spectra were acquired proton decoupled unless otherwise noted.

Mass Spectrometry: Electrospray ionization (ESI) mass spectrometry was conducted on a Bruker ESQ 3000 spectrometer. High resolution mass spectrometry (HRMS) was performed on a Finnigan MAT 95 (EI) or Bruker APEX III FTMS (7 T magnet, ESI). The ionization method and mode of detection employed is indicated for the respective experiment. The ionization method and mode of detection employed is indicated for the respective experiment and all masses are reported in atomic units per elementary charge (*m/z*) with an intensity normalized to the most intense peak.

Specific Rotations: Specific rotations ($[\alpha]_D{}^t[\alpha]_D^T$) were measured with a Rudolph RA Autopol IV Automatic Polarimeter at the indicated temperature with a sodium lamp (sodium D line, λ = 589 nm). Measurements were performed in an acid resistant 1 mL cell (50 mm length) with concentrations (g/(100 mL)) reported in the corresponding solvent.

High Performance Liquid Chromatography: High performance liquid chromatography (HPLC) was performed on a Shimadzu LC-20AD liquid chromatograph SIL-20AC auto sampler, CMB-20A using Daicel/Merck columns with a chiral stationary phase. All solvents used were HPLC-grade solvents purchased from Sigma-Aldrich. The column employed and the respective solvent mixture are indicated for each experiment.

Abbreviations: rt = room temperature, er = enantiomeric ratio, TLC = thin layer chromatography, THF = tetrahydrofuran, MTBE = methyl *tert*-butyl ether, MeCN = acetonitrile, Mesitylene = 1,3,5-trimethylbenzene, TBS = $SiMe_2{}^tBu$, TMS = $SiMe_3$, TES = triethylsilyl, Tf = SO_2CF_3, MOM = methoxymethyl ether.

2. Synthesis and Characterization of bis-Silyl Ketene Acetals

The substrates **1a**,[1] **1e**,[2] **1g-1h**,[2] **1k-1m**,[2] **1o-1x**,[2] **1z**[2] were synthesized according to literature procedures.

General Procedure:[2]

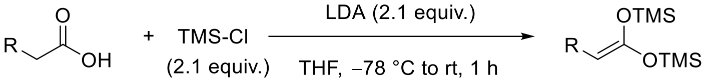

Preparation of lithium diisopropyl amine (LDA) solution: To a stirring solution of freshly distilled diisopropyl amine (21 mmol, 3.0 mL, 2.1 equiv.) in THF (10 mL) under an atmosphere of argon, *n*-BuLi (2.5 M solution in hexane, 21 mmol, 8.4 mL, 2.1 equiv.) was added dropwise at –78 °C and the resulting reaction mixture was stirred for additional 30 min at rt.

A stirring solution of acid (10 mmol, 1.0 equiv.) and TMSCl (2.6 mL, 21 mmol, 2.1 equiv.) in THF (5 mL) under an atmosphere of argon was cooled to –78 °C. The LDA solution was added via cannula. The cooling bath was removed after addition and the reaction solution was stirred for 1 h at rt. The reaction mixture was concentrated in vacuo, and then dry hexane (20 mL) was added to the residue and the mixture was filtered under an atmosphere of argon. After concentration under reduced pressure, the crude product was purified by distillation under reduced pressure to afford the bis-silyl ketene acetal as colorless liquid, which was stored in a Schlenk flask under an atmosphere of argon at 4 °C or –20 °C.

2,2,6,6-tetramethyl-4-(4-methybenzylidene)-3,5-dioxa-2,6-disilaheptane (1b)

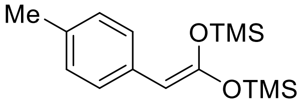

Prepared according to the general procedure using 2-(4-methoxyphenyl)acetic acid (1.66 g, 10 mmol) and obtained after distillation (bp = 74 °C at $2.6*10^{-2}$ mbar) as colorless liquid (2.64 g, 90% yield).

^{1}H NMR (501 MHz, $CDCl_2$) δ 7.26 (d, *J* = 8.2 Hz, 2H), 7.02 (d, *J* = 8.0 Hz, 2H), 4.59 (s, 1H), 2.27 (s, 3H), 0.32 (s, 9H), 0.29 (s, 9H).

^{13}C NMR (126 MHz, $CDCl_2$) δ 152.21, 135.05, 133.46, 129.22, 126.70, 85.56, 21.23, 0.85, 0.17.

HRMS m/z (EI): calculated for $C_{15}H_{26}O_2Si_2$ $[M]^+$: 294.1466, found 294.1463.

2,2,6,6-tetramethyl-4-(4-isobutylbenzylidene)-3,5-dioxa-2,6-disilaheptane (1c)

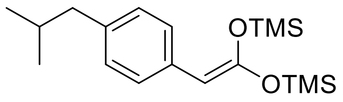

Prepared according to the general procedure using 2-(4-isobutylphenyl)acetic acid (1.92 g, 10 mmol) and obtained after distillation (bp = 90 °C at 4.4*10^{-2} mbar) as colorless liquid (2.76 g, 82% yield).

^{1}H NMR (501 MHz, $CDCl_2$) δ 7.28 (d, *J* = 8.4 Hz, 2H), 7.00 (d, *J* = 8.4 Hz, 2H), 4.59 (s, 1H), 2.40 (d, *J* = 7.1 Hz, 2H), 1.82 (dp, *J* = 13.6, 6.7 Hz, 1H), 0.89 (d, *J* = 6.6 Hz, 6H), 0.32 (s, 9H), 0.29 (s, 9H).

^{13}C NMR (126 MHz, $CDCl_2$) δ 152.3, 137.5, 135.3, 129.4, 126.5, 85.6, 45.5, 30.9, 22.7, 0.9, 0.2.

HRMS m/z (EI): calculated for $C_{18}H_{32}O_2Si_2$ $[M]^+$: 336.1935, found 336.1933.

2,2,6,6-tetramethyl-4-(4-methoxybenzylidene)-3,5-dioxa-2,6-disilaheptane (1d)

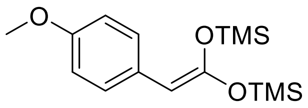

Prepared according to the general procedure using 2-(4-methoxyphenyl)acetic acid (1.66 g, 10 mmol) and obtained after distillation (bp = 90 °C at 3.0*10^{-2} mbar) as colorless liquid (2.25 g, 72% yield).

^{1}H NMR (501 MHz, $CDCl_2$) δ 7.34–7.26 (m, 2H), 6.83–6.73 (m, 2H), 4.57 (s, 1H), 3.76 (s, 3H), 0.32 (s, 9H), 0.28 (s, 9H).

^{13}C NMR (126 MHz, $CDCl_2$) δ 156.8, 151.6, 130.6, 127.8, 114.1, 85.1, 55.7, 0.8, 0.2.

HRMS m/z (EI): calculated for $C_{15}H_{26}O_3Si_2$ $[M]^+$: 310.1415, found 310.1414.

2,2,6,6-tetramethyl-4-(4-(trifluoromethoxy)benzylidene)-3,5-dioxa-2,6-disilaheptane (1f)

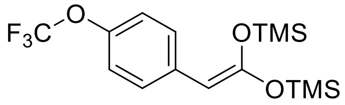

Prepared according to the general procedure using 2-(4-(trifluoromethoxy)phenyl)acetic acid (2.20 g, 10 mmol) and obtained after distillation (bp = 75 °C at 2.0*10^{-2} mbar) as colorless liquid (2.95 g, 81% yield).

^{1}H NMR (501 MHz, $CDCl_2$) δ 7.40 (d, *J* = 9.0 Hz, 2H), 7.07 (d, *J* = 9.0 Hz, 2H), 4.60 (s, 1H), 0.33 (s, 9H), 0.30 (s, 9H).

^{19}F NMR (471 MHz, $CDCl_2$) δ –58.37.

^{13}C NMR (126 MHz, $CDCl_2$) δ 153.4, 145.6, 137.4, 127.6, 121.2 (q, *J* = 255.9 Hz), 121.2, 84.3, 0.8, 0.1.

HRMS m/z (EI): calculated for $C_{15}H_{23}O_3F_3Si_2$ $[M]^+$: 364.1132, found 364.1128.

2,2,6,6-tetramethyl-4-(thiophen-2-ylmethylene)-3,5-dioxa-2,6-disilaheptane (1i)

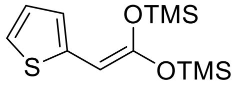

Prepared according to the general procedure using 2-(thiophen-2-yl)acetic acid (1.42 g, 10 mmol) and obtained after distillation (bp = 70 °C at 3.1*10^{-2} mbar) as colorless liquid (2.32 g, 81% yield).

^{1}H NMR (501 MHz, $CDCl_2$) δ 6.97 (dt, *J* = 5.0, 1.0 Hz, 1H), 6.87 (dd, *J* = 5.2, 3.5 Hz, 1H), 6.71 (dd, *J* = 3.6, 1.4 Hz, 1H), 5.00 (s, 1H), 0.32 (s, 9H), 0.32 (s, 9H).

^{13}C NMR (126 MHz, $CDCl_2$) δ 152.0, 141.5, 126.6, 121.2, 120.9, 81.5, 0.9, 0.1.

HRMS m/z (EI): calculated for $C_{12}H_{22}O_2Si_2S$ [M]$^+$: 286.0874, found 286.0873.

2,2,6,6-tetramethyl-4-(naphthalen-2-ylmethylene)-3,5-dioxa-2,6-disilaheptane (1j)

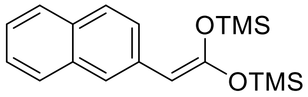

Prepared according to the general procedure using 2-(naphthalen-2-yl)acetic acid (1.86 g, 10 mmol) and obtained after distillation (bp = 108-110 °C at 1.5*10^{-2} mbar) as colorless liquid (2.58 g, 78% yield).

^{1}H NMR (300 MHz, $CDCl_2$) δ 7.85–7.80 (m, 1H), 7.75–7.66 (m, 3H), 7.58 (dd, *J* = 8.7, 1.8 Hz, 1H), 7.42–7.28 (m, 2H), 4.78 (s, 1H), 0.37 (s, 9H), 0.35 (s, 9H).

^{13}C NMR (75 MHz, $CDCl_2$) δ 153.3, 135.9, 134.6, 131.4, 127.9, 127.8, 127.7, 126.8, 126.2, 124.7, 123.8, 85.8, 0.9, 0.2.

HRMS m/z (EI): calculated for $C_{18}H_{26}O_2Si_2$ [M]$^+$: 330.1466, found 330.1460.

2,2,6,6-tetramethyl-4-(2-(furan-2-yl)ethylidene)-3,5-dioxa-2,6-disilaheptane (1n)

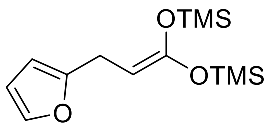

Prepared according to the general procedure using 3-(furan-2-yl)propanoic acid (1.40 g, 10 mmol) and obtained after distillation (bp = 55-56 °C at 1.2*10^{-2} mbar) as colorless liquid (2.12 g, 74% yield).

^{1}H NMR (501 MHz, $CDCl_2$) δ 7.29 (d, *J* = 1.1 Hz, 1H), 6.27 (dd, *J* = 3.2, 1.8 Hz, 1H), 5.95 (dq, *J* = 3.2, 1.0 Hz, 1H), 3.73 (t, *J* = 7.1 Hz, 1H), 3.25 (d, *J* = 7.1 Hz, 2H), 0.25 (s, 9H), 0.20 (s, 9H).

^{13}C NMR (126 MHz, $CDCl_2$) δ 157.5, 152.3, 141.1, 110.6, 104.6, 78.8, 24.9, 0.6, 0.1.

HRMS m/z (EI): calculated for $C_{13}H_{24}O_3Si_2$ [M]$^+$: 284.1256, found 284.1257.

4-(but-3-en-1-ylidene)-2,2,6,6-tetramethyl-3,5-dioxa-2,6-disilaheptane (1y)

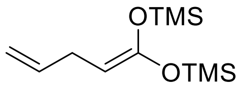

Prepared according to the general procedure using pent-4-enoic acid (1.00 g, 10 mmol) and obtained after distillation (bp = 70-71 °C at 12 mbar) as colorless liquid (1.88 g, 77% yield).

^{1}H NMR (501 MHz, $CDCl_3$) δ 5.81 (ddt, *J* = 17.2, 10.1, 6.0 Hz, 1H), 5.00 (dq, *J* = 17.0, 1.9 Hz, 1H), 4.89 (dq, *J* = 10.1, 1.6 Hz, 1H), 3.56 (t, *J* = 7.2 Hz, 1H), 2.67 (ddt, *J* = 7.4, 6.0, 1.6 Hz, 2H), 0.23 (s, 9H), 0.19 (s, 9H).

^{13}C NMR (126 MHz, $CDCl_3$) δ 151.0, 139.1, 113.2, 80.6, 29.7, 0.6, 0.0.

HRMS m/z (EI): calculated for $C_{11}H_{24}O_2Si_2$ $[M]^+$: 244.1309, found 244.1306.

2,2,6,6-tetramethyl-4-(4-(trifluoromethyl)benzylidene)-3,5-dioxa-2,6-disilaheptane (s1b)

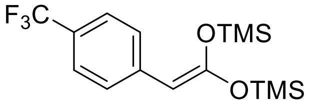

Prepared according to the general procedure using 2-(4-hydroxyphenyl)acetic acid (2.04 g, 10 mmol) and obtained after distillation (bp = 68 °C at 2.1*10^{-2} mbar) as colorless liquid (2.80 g, 80% yield).

^{1}H NMR (501 MHz, $CDCl_2$) δ 7.48 (d, *J* = 8.7 Hz, 2H), 7.44 (d, *J* = 8.7 Hz, 2H), 4.65 (s, 1H), 0.35 (s, 9H), 0.31 (s, 9H).

^{13}C NMR (126 MHz, $CDCl_2$) δ 154.5, 142.4, 126.4, 125.4 (q, *J* = 4.0 Hz), 84.6, 0.8, 0.1.

^{19}F NMR (471 MHz, $CDCl_2$) δ –62.22.

HRMS m/z (EI): calculated for $C_{15}H_{23}O_2F_3Si_2$ $[M]^+$: 348.1178, found 348.1183.

3. Reaction Development & Optimization

General Procedure:

An oven-dried 1.5 mL vial was charged with catalyst and a magnetic stir bar under an atmosphere of argon. Dry solvent and bis-silyl ketene acetal **1a** (8 µL, 0.024 mmol, 1.2 equiv.) were added. The vial was cooled to required reaction temperature. Then **2** (0.02 mmol, 1.0 equiv.) was added and the reaction was stirred for 18 h or 3 d.

Then the reaction mixture was treated with methanol (0.12 mmol, 5 µL) at reaction temperature and stirred for 30 min before reaching to rt. Dibromomethane (internal standard, 1.4 µL, 0.02 mmol, 1.0 equiv.) was added and ^{1}H NMR analysis was carried out to determine the yield and *E*/*Z* ratio. The product was isolated by prep-TLC and er was determined by chiral HPLC.

Table S1. Initial screening of catalysts

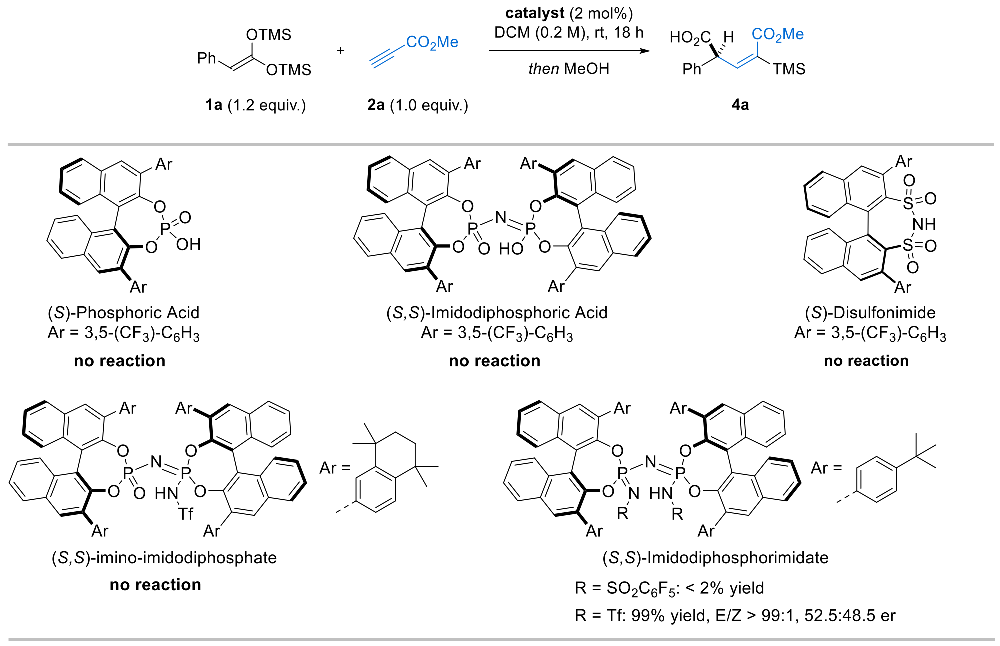

Table S2. Solvent screening

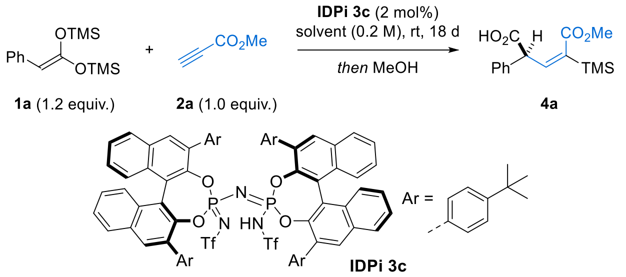

entry	solvent	yield	E/Z	er
1	DCM	99	>99:1	52.5:47.5
2	toluene	99	>99:1	72.5:27.5
3	CyMe	99	>99:1	75:25
4	pentane	99	>99:1	74.5:25.5
5	Et_2O	99	>99:1	71.5:28.5

Table S3. Temperature and catalyst loading screening

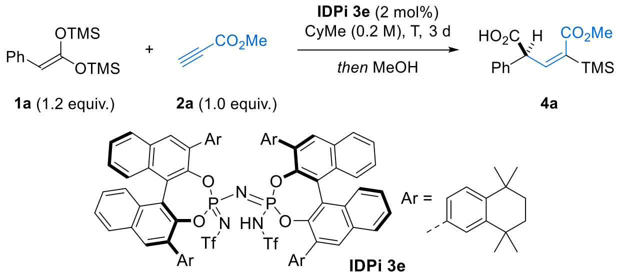

entry	T (°C)	yield	E/Z	er
1	rt	99	>99:1	93.5:6.5
2	10	99	>99:1	95.5:4.5
3	0	99	>99:1	97:3
4	–20	99	>99:1	97.5:2.5
5	–40	99	>99:1	98:2
6	–60	99	>99:1	>99.5:0.5
7[a]	–60	99	>99:1	>99.5:0.5
8[b]	–60	95	>99:1	>99.5:0.5
9	–80	0	-	-

[a]1 mol% **IDPi 3e**. [b]0.02 mmol scale of bis-SKA **1a** with 1.5 equiv. of **2a**.

Table S4. Screening of concentration effects

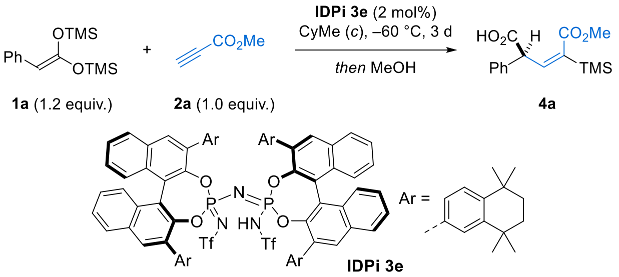

entry	*c*	yield	E/Z	er
1	0.2	99	>99:1	>99.5:0.5
2	0.4	99	>99:1	>99.5:0.5
3	2	97	>99:1	>99.5:0.5
4	4	45	>99:1	>99.5:0.5
5	neat	0	-	-

Table S5. Screening of propiolate

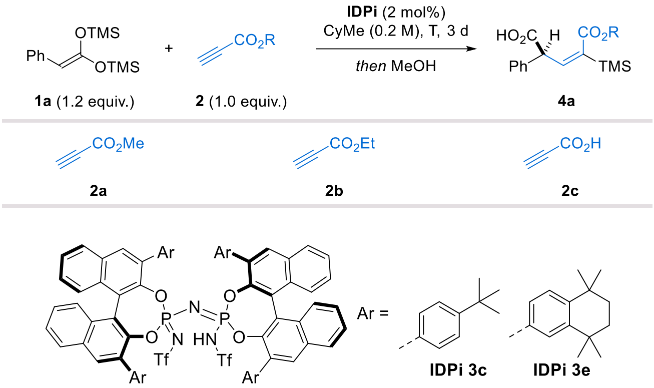

entry	propiolate	**IDPi**	T (°C)	yield	E/Z	er
1	**2a**	**3e**	–60	99	>99:1	>99.5:0.5
2	**2b**	**3e**	–60	5	>99:1	95:5
3[a]	**2c**	**3e**	–60	0	-	-
4	**2a**	**3c**	0	99	>99:1	77.5:22.5
5	**2b**	**3c**	0	99	>99:1	73.5:26.5
6[a]	**2c**	**3c**	0	99	>99:1	61:39

[a]2.4 equiv. of bis-SKA **1a**.

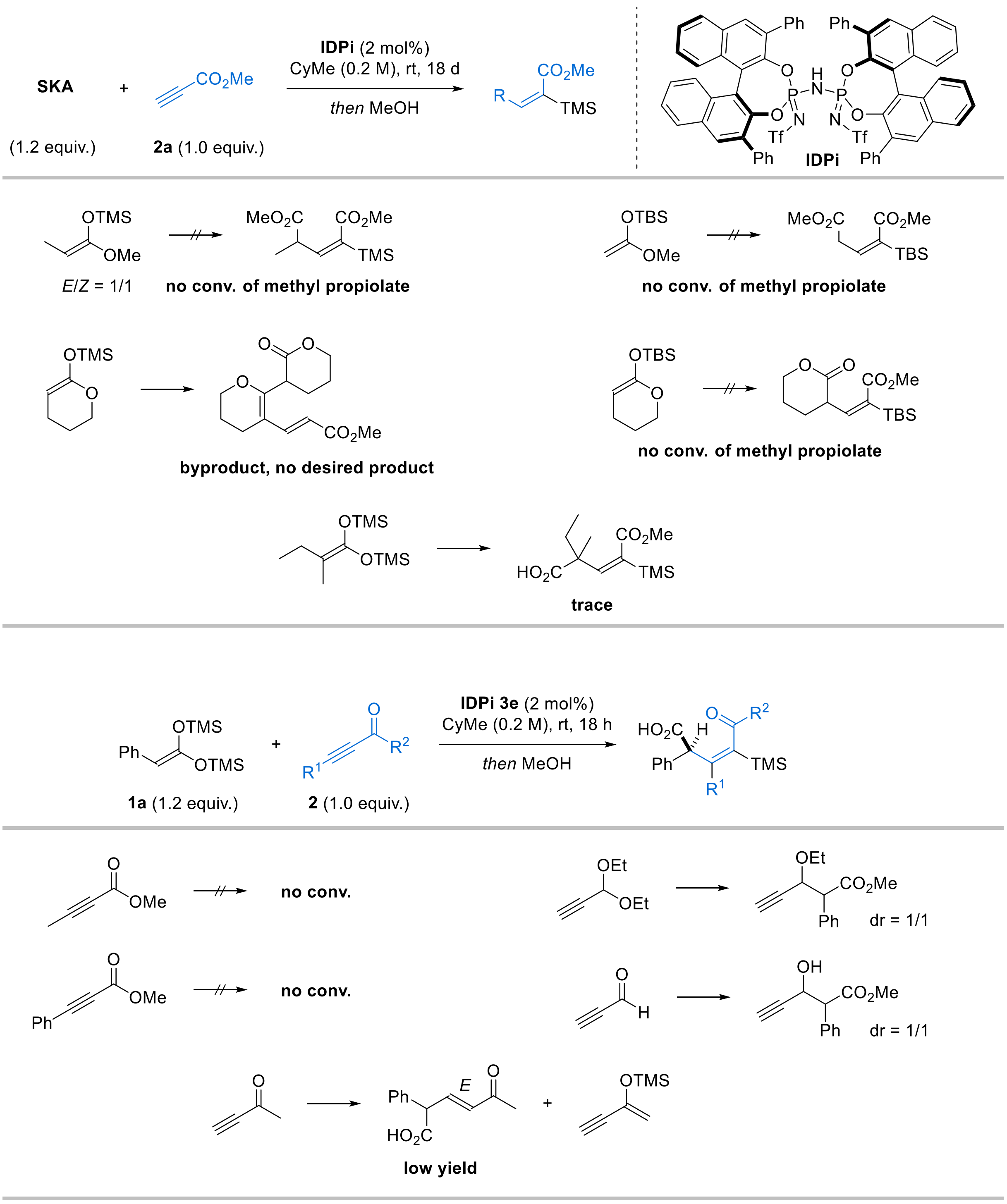

Scheme S1. *Limitations.*

5. Synthesis and Characterization of Catalysts

The chiral imidodiphosphorimidate acids (IDPis) **3a,**[3] **3b-3c,**[4] and **3d**[5] were synthesized according to literature procedures.

(*S*)-5,5,5''',5''',8,8,8''',8'''-octamethyl-5,5''',6,6''',7,7''',8,8'''-octahydro-[2,2':4',1'':3'',2'''-quaternaphthalene]-2'',3'-diol

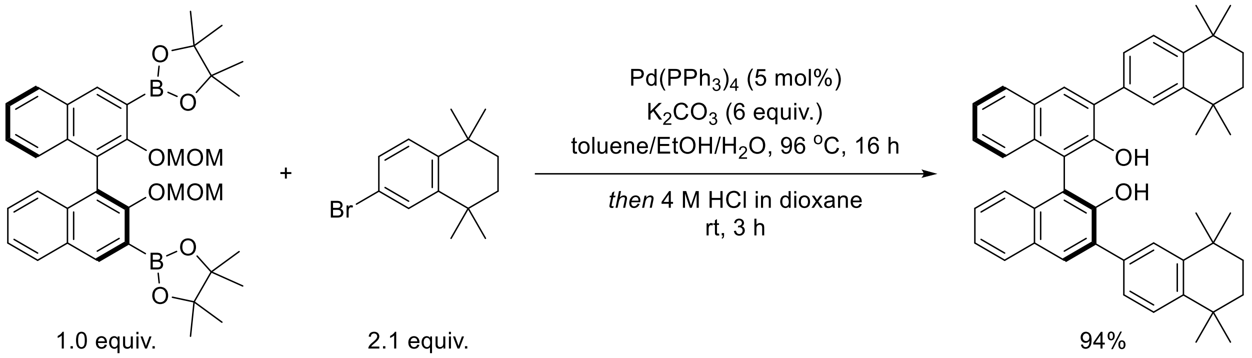

To a two-neck round-bottom flask with a condenser was added (*S*)-2,2'-(2,2'-bis(methoxymethoxy)-1,1'binaphthyl-3,3'-diyl)bis(4,4,-5,5-tetramethyl-1,3,2-dioxaborolane) (250 mg, 0.4 mmol, 1.0 equiv.), 6-bromo-1,1,4,4-tetramethyl-1,2,3,4-tetrahydronaphthalene (224 mg, 0.84 mmol, 2.1 equiv.), tetrakis(triphenylphosphine)palladium (23.1 mg, 0.02 mmol, 0.5 equiv.) and K_2CO_3 (332 mg, 2.4 mmol, 6 equiv.) under argon atmosphere. Degassed toluene (3 mL), ethanol (2 mL) and water (1 mL) were sequentially added. The mixture was then heated to 96 °C and stirred at that temperature overnight. After cooling the reaction to rt, the organic layer was separated and the aqueous phase was extracted with EtOAc (3x10 mL). The organic phase was combined and filtered through a thin layer of silica gel using a Büchner funnel, and the silica gel layer was washed with some extra EtOAc. The solvent was removed under reduced pressure and the crude MOM-protected diol was obtained. Subsequently, the crude product was dissolved in a small amount of DCM (1 mL). A solution of HCl (4 M in 1,4-dioxane, 3 mL) was added at rt and the mixture was stirred at rt for 3 h. The solvent was removed under reduced pressure and the crude was purified by column chromatography (hexanes:DCM = 2:1) to afford the corresponding diol as an off-white solid (248.8 mg, 94%).

^{1}H NMR (501 MHz, $CDCl_3$) δ 7.92 (s, 2H), 7.81 (d, *J* = 8.2 Hz, 2H), 7.59 (s, 2H), 7.40 (d, *J* = 8.4 Hz, 2H), 7.33 (d, *J* = 8.2 Hz, 2H), 7.26 (t, *J* = 7.4 Hz, 2H), 7.18 (t, *J* = 7.6 Hz, 2H), 7.14 (d, *J* = 8.5 Hz, 2H), 5.34 (s, 2H), 1.63 (s, 8H), 1.24 (d, *J* = 4.9 Hz, 24H).

^{13}C NMR (126 MHz, $CDCl_3$) δ 150.3, 145.2, 144.7, 134.5, 133.0, 131.1, 131.0, 129.6, 128.5, 127.9, 127.2, 126.9, 126.9, 124.5, 124.3, 112.8, 35.3, 35.2, 34.6, 34.4, 32.1, 32.0, 32.0.

HRMS m/z (ESI): calculated for $C_{48}H_{49}O_2$ $[M-H]^-$: 657.3738, found 657.3743.

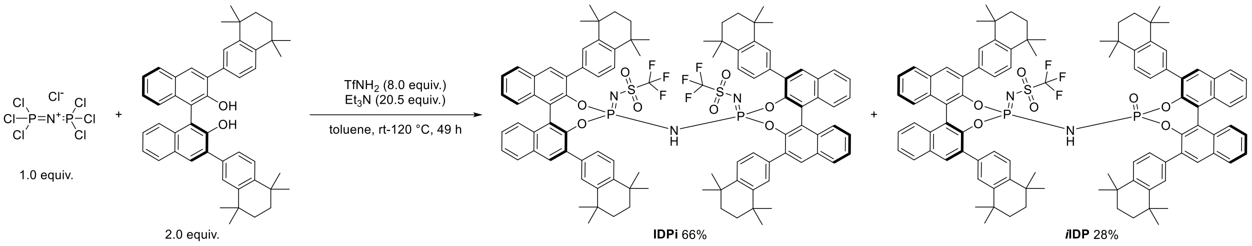

Modified according to the literature.[6] A flame dried Schlenk tube under argon was charged with 3,3'-substituted (*S*,*S*)-BINOL (1.6 mmol, 2.0 equiv., 1.054 g) and hexachlorobisphosphazonium chloride (HCPC, 0.8 mol, 1.0 equiv., 259 mg) and suspended in toluene (1.5 mL). Triethylamine (3.6 mmol, 4.5 equiv., 0.5 mL) was added dropwise via a syringe under vigorous stirring at rt. The reaction mixture was stirred for additional 1 h at rt followed by the addition of trifluoromethanesulfonamide ($TfNH_2$, 6.4 mmol, 8.0 equiv., 955 mg) and additional triethylamine (12.8 mmol, 16 equiv., 1.8 mL) and stirred at 80 °C for 48 h. After cooling to rt, aq. HCl (10%) was added and the mixture was extracted with DCM. The combined organic layers were washed with brine, dried over $MgSO_4$ and concentrated under reduced pressure. The crude material was purified by column chromatography on silica gel (hexanes:DCM = 1:1→1:4) to afford the desired products as a salt. The corresponding IDPi (889.6 mg, 66%) and *i*IDP (348.1 mg, 28%) Brønsted acids were obtained after acidification in DCM with aq. HCl (6 M) and evaporation of the solvent followed by drying under high vacuum as typically off-white solids. [Acidification procedure: The product as a salt was dissolved in 5 mL DCM, then aq. HCl (6 M, 8 mL) was added, and the mixture was stirred vigorously for 10 minutes. The organic layer was separated and the aqueous phase was extracted with DCM for several times until no product could be detected by TLC in the last DCM extract. The organic layers were combined and the solvent was removed under reduced pressure. The obtained IDPi and *i*IDP were dried under high vacuum for 16 h at rt.]

(*S*,*S*)-Imidodiphosphorimidate (IDPi 3e)

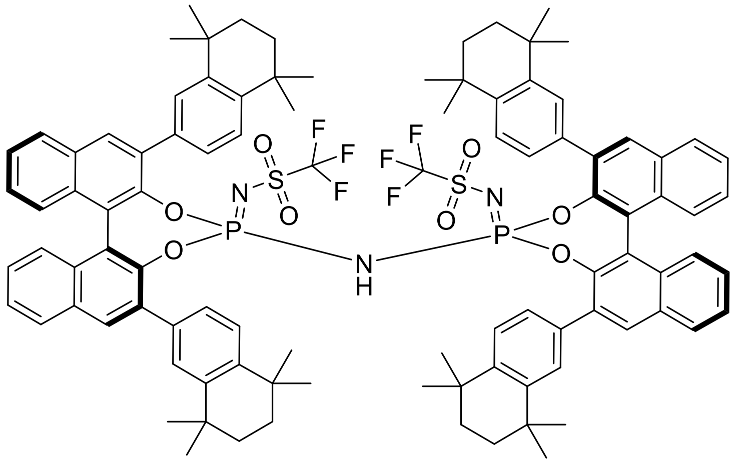

^{1}H NMR (501 MHz, CD_2Cl_2) δ 8.13 (s, 2H), 8.06 (d, *J* = 8.4 Hz, 2H), 7.94 (d, *J* = 8.2 Hz, 2H), 7.79–7.72 (m, 2H), 7.63–7.51 (m, 8H), 7.35–7.25 (m, 4H), 7.15 (d, *J* = 2.2 Hz, 2H), 7.13 (d, *J* = 8.2 Hz, 2H), 7.07 (s, 2H), 6.76 (dd, *J* = 8.4, 2.0 Hz, 2H), 6.73 (d, *J* = 8.2 Hz, 2H), 6.07 (dd, *J* = 8.3, 2.1 Hz, 2H), 1.73–1.63 (m, 10H), 1.59–1.52 (m, 6H), 1.42 (s, 6H), 1.29 (s, 6H), 1.27 (s, 6H), 1.25 (s, 6H), 1.18 (s, 6H), 1.09 (s, 6H), 1.00 (s, 6H), 1.00 (s, 6H).

^{13}C NMR (126 MHz, CD_2Cl_2) δ 145.9, 145.4, 145.3, 145.0, 134.3, 134.2, 133.6, 133.0, 132.8, 132.3, 132.2, 132.2, 132.0, 129.7, 129.2, 128.6, 127.7, 127.7, 127.6, 127.5, 127.4, 127.1, 127.0, 127.0, 126.9, 126.9, 126.5, 126.0, 123.7, 122.6, 35.8, 35.7, 35.6, 35.4, 34.8, 34.7, 34.6, 34.5, 32.5, 32.5, 32.2, 32.1, 31.9, 31.9, 31.7, 31.6. (other signals not detected or observed)

^{19}F NMR (471 MHz, CD_2Cl_2) δ –78.6.

^{31}P NMR (203 MHz, CD_2Cl_2) δ –16.5.

HRMS m/z (ESI): calculated for $C_{98}H_{96}F_6N_3O_8P_2S_2$ $[M-H]^-$: 1682.6024, found 1682.6027.

(*S*,*S*)-Iminoimidodiphosphates (*i*IDP)

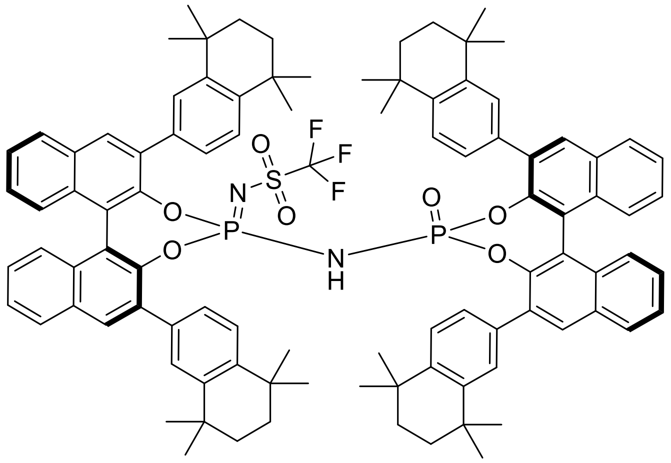

^{1}H NMR (501 MHz, $CDCl_2$) δ 8.14–8.08 (m, 3H), 8.07–7.99 (m, 3H), 7.75–7.67 (m, 3H), 7.62 (t, *J* = 8.0 Hz, 2H), 7.59–7.52 (m, 6H), 7.47 (d, *J* = 8.4 Hz, 1H), 7.43 (d, *J* = 2.0 Hz, 1H), 7.39–7.31 (m, 3H), 7.29–7.22 (m, 3H), 7.20–7.12 (m, 2H), 6.90 (dd, *J* = 8.4, 2.0 Hz, 1H), 6.07 (d, *J* = 8.2 Hz, 1H), 6.01 (d, *J* = 8.2 Hz, 1H), 5.78 (dd, *J* = 8.1, 2.0 Hz, 1H), 5.59 (dd, *J* = 8.2, 2.0 Hz, 1H), 1.76–1.65 (m, 8H), 1.64–1.46 (m, 8H), 1.40 (s, 3H), 1.33 (d, *J* = 1.4 Hz, 6H), 1.26 (s, 3H), 1.25 (s, 3H), 1.23 (s, 3H), 1.20 (s, 3H), 1.14 (s, 3H), 1.08 (s, 3H), 1.06 (s, 3H), 0.99 (s, 3H), 0.95 (s, 3H), 0.91 (s, 3H), 0.74 (s, 3H).

^{13}C NMR (126 MHz, $CDCl_2$) δ 146.0, 145.6, 145.4, 145.3, 145.1, 145.0, 144.9, 144.9, 144.6, 144.5, 144.5, 144.2, 143.7, 143.7, 135.7, 135.7, 135.2, 135.2, 134.4, 134.4, 134.3, 134.2, 134.1, 133.9, 133.8, 133.6, 132.6, 132.6, 132.4, 132.3, 132.2, 131.6, 131.4, 131.0, 129.4, 129.3, 129.2, 129.0, 129.0, 128.5, 128.1, 128.1, 127.8, 127.7, 127.5, 127.5, 127.4, 127.4, 127.2, 127.2, 127.1, 127.0, 127.0, 126.8, 126.7, 126.7, 126.5, 126.4, 126.2, 126.0, 123.6, 123.6, 123.1, 123.1, 122.8, 122.8, 35.8, 35.8, 35.6, 35.6, 35.5, 35.5, 35.4, 34.9, 34.8, 34.6, 34.6, 34.6, 34.5, 34.4, 34.4, 32.5, 32.3, 32.2, 32.2, 32.2, 32.1, 32.1, 32.0, 32.0, 31.9, 31.8, 31.6, 31.4. (other signals not detected or observed)

^{19}F NMR (471 MHz, $CDCl_2$) δ –79.61.

^{31}P NMR (203 MHz, $CDCl_2$) δ –1.31 (d, *J* = 108.6 Hz), –6.91 (d, *J* = 108.6 Hz).

6. Enantioselective and *E*-Selective Organocatalytic Alkenylation of bis-SKAs

General Procedure:

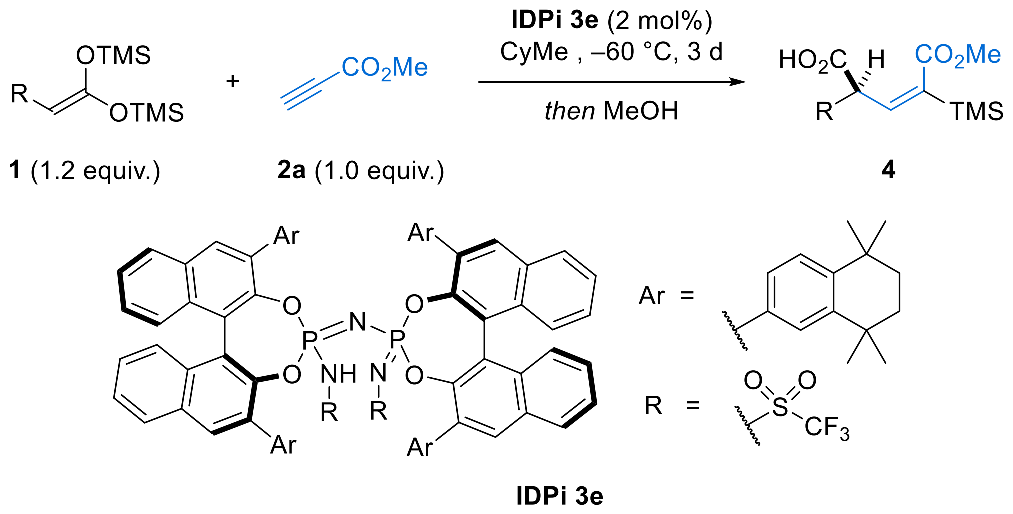

An oven-dried 2 ml vial was charged with **IDPi-3e** (4.0 µmol, 6.8 mg, 2 mol%) and a magnetic stir bar under an atmosphere of argon. Dry CyMe (1 mL) and bis-silyl ketene acetal **1** (0.24 mmol, 1.2 equiv.) were added. The vial was cooled to –60 °C. The **2a** (16.8 mg, 0.2 mmol, 1.0 equiv.) was added dropwise at –60 °C and the reaction was stirred at –60 °C for 3 d.

Afterwards, the reaction mixture was treated with MeOH (0.3 mL) at –60 °C. After the mixture was stirred for 30 min at –60 °C, the mixture was warmed to rt and the solvent was removed under reduced pressure. The residue was purified by column chromatography on silica gel (hexane/acetone/AcOH from 20/1/0.1 to 10/1/0.1) to afford product **4**.

Racemate synthesis: The racemic product was synthesized at rt for 6 h by using racemic **IDPi-3c** as the catalyst with the same purification process.

(*S*,*E*)-5-methoxy-5-oxo-2-phenyl-4-(trimethylsilyl)pent-3-enoic acid (4a)

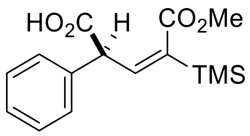

The reaction was performed according to the general procedure and after purification process afforded 56.4 mg (96%, >99.5:0.5 er) of **4a** as a colorless oil:

^{1}H NMR (501 MHz, $CDCl_3$) δ 7.37–7.32 (m, 4H), 7.32–7.27 (m, 1H), 6.58 (d, *J* = 9.5 Hz, 1H), 5.09 (d, *J* = 9.3 Hz, 1H), 3.77 (s, 3H), 0.15 (s, 9H).

^{13}C NMR (126 MHz, $CDCl_3$) δ 175.5, 170.7, 147.6, 138.3, 137.4, 129.1, 128.3, 127.9, 52.7, 51.8, –1.3.

HRMS m/z (ESI): calculated for $C_{15}H_{20}O_4SiNa$ $[M+Na]^+$: 315.1023, found 315.1023.

$[\alpha]_D^{25}$ = +28.0 (*c* 1.02, CH_2Cl_2).

The enantiomeric excess was determined by chiral HPLC analysis on IE-3 column. Conditions: heptane/isopropanol = 98:2, flow rate = 1.0 mL/min, uv-vis detection at λ = 220 nm, t_{R1} = 7.7 min (minor), t_{R2} = 9.2 min (major).

For 7 mmol scale: The reaction was performed in 7 mmol scale of **2a** according to the general procedure using 1 mol% **IDPi-3e** (110 mg), which afforded 1.99 g (97%, >99.5:0.5 er) of **4a** as a colorless oil after purification. Catalyst **IDPi-3e** (103 mg, 94%) was recovered from column chromatography on silica and acidified by HCl (see a detailed procedure in the paragraph of Synthesis and Characterization of Catalysts).

(*S*,*E*)-5-methoxy-5-oxo-2-(p-tolyl)-4-(trimethylsilyl)pent-3-enoic acid (4b)

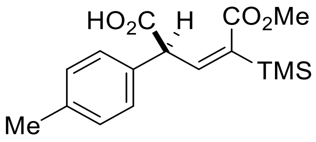

The reaction was performed according to the general procedure and after purification process afforded 52.0 mg (85%, >99.5:0.5 er) of **4b** as a colorless oil:

^{1}H NMR (501 MHz, $CDCl_3$) δ 10.63 (br s, 1H), 7.25 (d, *J* = 8.0 Hz, 2H), 7.16 (d, *J* = 8.0 Hz, 2H), 6.57 (d, *J* = 9.5 Hz, 1H), 5.04 (d, *J* = 9.5 Hz, 1H), 3.78 (s, 3H), 2.33 (s, 3H), 0.15 (s, 9H).

^{13}C NMR (126 MHz, $CDCl_3$) δ 174.5, 171.1, 148.2, 138.0, 137.7, 134.3, 129.7, 128.2, 52.4, 51.9, 21.2, –1.3.

HRMS m/z (ESI): calculated for $C_{16}H_{22}O_4SiNa$ $[M+Na]^+$: 329.1180, found 329.1184.

$[\alpha]_D^{25}$ = +36.8 (*c* 0.60, CH_2Cl_2).

The enantiomeric excess was determined by chiral HPLC analysis on IE-3 column. Conditions: heptane/isopropanol = 98:2, flow rate = 1.0 mL/min, uv-vis detection at λ = 220 nm, t_{R1} = 9.0 min (minor), t_{R2} = 11.9 min (major).

(*S*,*E*)-2-(4-isobutylphenyl)-5-methoxy-5-oxo-4-(trimethylsilyl)pent-3-enoic acid (4c)

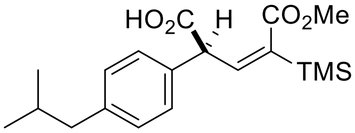

The reaction was performed according to the general procedure and after purification process afforded 66.9 mg (96%, 99:1 er) of **4c** as a colorless oil:

^{1}H NMR (501 MHz, $CDCl_3$) δ 7.25 (d, *J* = 8.2 Hz, 2H), 7.12 (d, *J* = 8.4 Hz, 2H), 6.57 (d, *J* = 9.5 Hz, 1H), 5.04 (d, *J* = 9.5 Hz, 1H), 3.76 (s, 3H), 2.45 (d, *J* = 7.3 Hz, 2H), 1.85 (hept, *J* = 6.8 Hz, 1H), 0.90 (d, *J* = 6.5 Hz, 6H), 0.15 (s, 9H).

^{13}C NMR (126 MHz, $CDCl_3$) δ 175.9, 170.8, 147.8, 141.4, 138.0, 134.5, 129.8, 128.0, 52.4, 51.8, 45.2, 30.3, 22.6, 22.5, –1.3.

HRMS m/z (ESI): calculated for $C_{19}H_{29}O_4Si$ $[M+H]^+$: 349.1830, found 349.1830.

$[\alpha]_D^{25}$ = +33.6 (*c* 0.61, CH_2Cl_2).

The enantiomeric excess was determined by chiral HPLC analysis on IE-3 column. Conditions: heptane/isopropanol = 98:2, flow rate = 1.0 mL/min, uv-vis detection at λ = 220 nm, t_{R1} = 7.4 min (minor), t_{R2} = 9.2 min (major).

(*S*,*E*)-5-methoxy-2-(4-methoxyphenyl)-5-oxo-4-(trimethylsilyl)pent-3-enoic acid (4d)

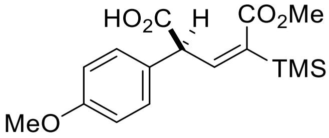

The reaction was performed according to the general procedure and after purification process afforded 63.4 mg (98%, 99:1 er) of **4d** as a colorless oil:

^{1}H NMR (501 MHz, $CDCl_3$) δ 7.29–7.25 (m, 2H), 6.91–6.85 (m, 2H), 6.56 (d, *J* = 9.3 Hz, 1H), 5.03 (d, *J* = 9.3 Hz, 1H), 3.79 (s, 3H), 3.75 (s, 3H), 0.15 (s, 9H).

^{13}C NMR (126 MHz, $CDCl_3$) δ 176.5, 170.6, 159.2, 147.7, 137.8, 129.5, 129.3, 114.5, 55.4, 51.8, 51.7, –1.3.

HRMS m/z (ESI): calculated for $C_{16}H_{22}O_5SiNa$ $[M+Na]^+$: 345.1129, found 345.1131.

$[\alpha]_D^{25}$ = +29.4 (*c* 0.46, CH_2Cl_2).

The enantiomeric excess was determined by chiral HPLC analysis on IE-3 column. Conditions: heptane/isopropanol = 98:2, flow rate = 1.0 mL/min, uv-vis detection at λ = 220 nm, t_{R1} = 14.2 min (minor), t_{R2} = 16.7 min (major).

(*S*,*E*)-2-(4-bromophenyl)-5-methoxy-5-oxo-4-(trimethylsilyl)pent-3-enoic acid (4e)

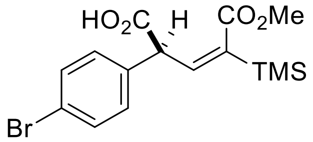

The reaction was performed according to the general procedure and after purification process afforded 72.7 mg (98%, 99:1 er) of **4e** as a colorless oil:

^{1}H NMR (501 MHz, $CDCl_3$) δ 7.50–7.45 (m, 2H), 7.25–7.21 (m, 2H), 6.52 (d, *J* = 9.1 Hz, 1H), 5.06 (d, *J* = 9.3 Hz, 1H), 3.76 (s, 3H), 0.15 (s, 9H).

^{13}C NMR (126 MHz, $CDCl_3$) δ 175.5, 170.5, 146.8, 138.8, 136.4, 132.2, 130.0, 122.0, 52.0, 51.9, –1.4.

HRMS m/z (ESI): calculated for $C_{15}H_{19}O_4BrSiNa$ $[M+Na]^+$: 393.0128, found 393.0130.

$[\alpha]_D^{25}$ = +29.3 (*c* 0.46, CH_2Cl_2).

The enantiomeric excess was determined by chiral HPLC analysis on IE-3 column. Conditions: heptane/isopropanol = 98:2, flow rate = 1.0 mL/min, uv-vis detection at λ = 220 nm, t_{R1} = 6.1 min (minor), t_{R2} = 7.0 min (major).

For 5.2 mmol scale: The reaction was performed in 5.2 mmol scale of **2a** according to the general procedure using 1 mol% **IDPi-3e** (85 mg), which afforded 1.89 g (98%, 99:1 er) of **4e** as a colorless oil after purification. Catalyst **IDPi-3e** (77 mg, 91%) was recovered from column chromatography on silica and acidified by HCl (see a detailed procedure in the paragraph of Synthesis and Characterization of Catalysts).

(*S*,*E*)-5-methoxy-5-oxo-2-(4-(trifluoromethoxy)phenyl)-4-(trimethylsilyl)pent-3-enoic acid (4f)

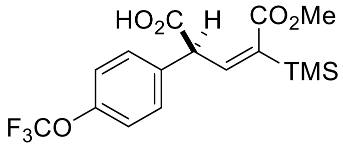

The reaction was performed according to the general procedure and after purification process afforded 67.1 mg (89%, >99.5:0.5 er) of **4f** as a colorless oil:

^{1}H NMR (501 MHz, $CDCl_3$) δ 7.40 (d, *J* = 8.8 Hz, 2H), 7.21 (dt, *J* = 7.7, 0.9 Hz, 2H), 6.52 (d, *J* = 9.3 Hz, 1H), 5.10 (d, *J* = 9.3 Hz, 1H), 3.80 (s, 3H), 0.17 (s, 9H).

^{13}C NMR (126 MHz, $CDCl_3$) δ 175.5, 170.6, 148.9, 146.8, 139.0, 135.9, 129.8, 121.5, 121.9 (q, *J* = 257.5 Hz), 51.9, –1.4.

^{19}F NMR (471 MHz, $CDCl_3$) δ –57.84.

HRMS m/z (ESI): calculated for $C_{16}H_{19}O_5F_3SiNa$ $[M+Na]^+$: 399.0846, found 399.0849.

$[\boldsymbol{\alpha}]_{\boldsymbol{D}}^{\mathbf{25}}$ = –27.0 (*c* 0.13, CH_2Cl_2).

The enantiomeric excess was determined by chiral HPLC analysis on IE-3 column. Conditions: heptane/isopropanol = 98:2, flow rate = 1.0 mL/min, uv-vis detection at λ = 220 nm, t_{R1} = 8.4 min (minor), t_{R2} = 9.6 min (major).

(*S*,*E*)-2-(3-chlorophenyl)-5-methoxy-5-oxo-4-(trimethylsilyl)pent-3-enoic acid (4g)

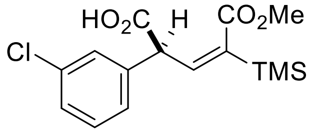

The reaction was performed according to the general procedure at –40 °C and after purification process afforded 59.5 mg (91%, >99.5:0.5 er) of **4g** as a colorless oil:

^{1}H NMR (501 MHz, $CDCl_3$) δ 7.38–7.24 (m, 4H), 6.51 (d, *J* = 9.3 Hz, 1H), 5.07 (d, *J* = 9.3 Hz, 1H), 3.78 (s, 3H), 0.16 (s, 9H).

^{13}C NMR (126 MHz, $CDCl_3$) δ 174.2, 170.8, 146.8, 139.1, 134.8, 130.3, 128.6, 128.1, 126.6, 52.3, 52.0, –1.3.

HRMS m/z (ESI): calculated for $C_{15}H_{20}O_4ClSi$ $[M+H]^+$: 327.0814, found 327.0814.

$[\boldsymbol{\alpha}]_{\boldsymbol{D}}^{\mathbf{25}}$ = –6.3 (*c* 0.42, CH_2Cl_2).

The enantiomeric excess was determined by chiral HPLC analysis on IE-3 column. Conditions: heptane/isopropanol = 98:2, flow rate = 1.0 mL/min, uv-vis detection at λ = 220 nm, t_{R1} = 6.3 min (minor), t_{R2} = 7.4 min (major).

(*S*,*E*)-2-(3,4-dichlorophenyl)-5-methoxy-5-oxo-4-(trimethylsilyl)pent-3-enoic acid (4h)

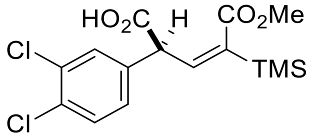

The reaction was performed according to the general procedure and after purification process afforded 67.2 mg (93%, >99.5:0.5 er) of **4h** as a colorless oil:

^{1}H NMR (501 MHz, $CDCl_3$) δ 7.46 (d, *J* = 2.2 Hz, 1H), 7.43 (d, *J* = 8.4 Hz, 1H), 7.22 (dd, *J* = 8.4, 2.0 Hz, 1H), 6.48 (d, *J* = 9.3 Hz, 1H), 5.07 (d, *J* = 9.3 Hz, 1H), 3.78 (s, 3H), 0.16 (s, 9H).

^{13}C NMR (126 MHz, $CDCl_3$) δ 174.6, 170.6, 146.3, 139.5, 137.3, 133.1, 132.2, 131.0, 130.4, 127.8, 52.0, 51.6, –1.4.

HRMS m/z (ESI): calculated for $C_{15}H_{18}O_4Cl_2SiNa$ $[M+Na]^+$: 383.0244, found 383.0249.

$[\alpha]_D^{25}$ = +19.8 (*c* 1.30, CH_2Cl_2).

The enantiomeric excess was determined by chiral HPLC analysis on IE-3 column. Conditions: heptane/isopropanol = 98:2, flow rate = 1.0 mL/min, uv-vis detection at λ = 220 nm, t_{R1} = 5.6 min (minor), t_{R2} = 6.0 min (major).

(*R*,*E*)-5-methoxy-5-oxo-2-(thiophen-2-yl)-4-(trimethylsilyl)pent-3-enoic acid (4i)

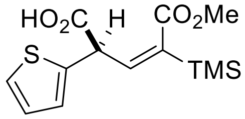

The reaction was performed according to the general procedure and after purification process afforded 56.6 mg (95%, >99.5:0.5 er) of **4i** as a colorless oil:

^{1}H NMR (501 MHz, $CDCl_3$) δ 7.28–7.26 (m, 1H), 7.02 (dt, *J* = 3.5, 1.0 Hz, 1H), 6.99 (dd, *J* = 5.1, 3.5 Hz, 1H), 6.51 (d, *J* = 9.3 Hz, 1H), 5.38 (dd, *J* = 9.3, 0.6 Hz, 1H), 3.81 (s, 3H), 0.18 (s, 9H).

^{13}C NMR (126 MHz, $CDCl_3$) δ 173.3, 171.2, 147.2, 138.8, 138.6, 127.0, 126.2, 125.5, 52.2, 48.1, –1.4.

HRMS m/z (ESI): calculated for $C_{13}H_{18}O_4SSiNa$ $[M+Na]^+$: 321.0587, found 321.0587.

$[\alpha]_D^{25}$ = –37.9 (*c* 1.03, CH_2Cl_2).

The enantiomeric excess was determined by chiral HPLC analysis on IE-3 column. Conditions: heptane/isopropanol = 98:2, flow rate = 1.0 mL/min, uv-vis detection at λ = 220 nm, t_{R1} = 8.4 min (minor), t_{R2} = 9.7 min (major).

(*S*,*E*)-5-methoxy-2-(naphthalen-2-yl)-5-oxo-4-(trimethylsilyl)pent-3-enoic acid (4j)

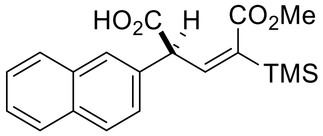

The reaction was performed according to the general procedure and after purification process afforded 41.2 mg (60%, 99:9 er) of **4j** as a colorless oil:

^{1}H NMR (501 MHz, $CDCl_3$) δ 7.90–7.74 (m, 4H), 7.55–7.43 (m, 3H), 6.68 (d, *J* = 9.3 Hz, 1H), 5.26 (d, *J* = 9.3 Hz, 1H), 3.80 (s, 3H), 0.16 (s, 9H).

^{13}C NMR (126 MHz, $CDCl_3$) δ 176.0, 170.7, 147.4, 138.4, 134.8, 133.6, 132.9, 128.9, 128.1, 127.8, 127.3, 126.4, 126.3, 126.1, 52.7, 51.8, –1.3.

HRMS m/z (ESI): calculated for $C_{19}H_{22}O_4SiNa$ $[M+Na]^+$: 365.1180, found 365.1181.

$[\alpha]_D^{25}$ = +93.3 (*c* 0.91, CH_2Cl_2).

The enantiomeric excess was determined by chiral HPLC analysis on IE-3 column. Conditions: heptane/isopropanol = 98:2, flow rate = 1.0 mL/min, uv-vis detection at λ = 220 nm, t_{R1} = 10.0 min (minor), t_{R2} = 11.7 min (major).

(*R*,*E*)-2-benzyl-5-methoxy-5-oxo-4-(trimethylsilyl)pent-3-enoic acid (4k)

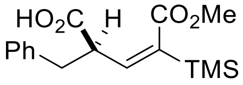

The reaction was performed according to the general procedure and after purification process afforded 59.7 mg (97%, 95:5 er) of **4k** as a colorless oil:

^{1}H NMR (501 MHz, $CDCl_3$) δ 11.12 (br s, 1H), 7.28–7.24 (m, 2H), 7.22–7.17 (m, 1H), 7.17–7.11 (m, 2H), 6.20 (d, *J* = 9.5 Hz, 1H), 4.03 (ddd, *J* = 9.5, 7.6, 6.8 Hz, 1H), 3.75 (s, 3H), 3.25 (dd, *J* = 14.0, 6.9 Hz, 1H), 2.93 (dd, *J* = 14.0, 7.6 Hz, 1H), 0.10 (s, 9H).

^{13}C NMR (126 MHz, $CDCl_3$) δ 173.6, 172.3, 149.5, 139.4, 138.1, 129.2, 128.6, 126.8, 52.3, 49.8, 37.8, –1.4.

HRMS m/z (ESI): calculated for $C_{16}H_{22}O_4SiNa$ $[M+Na]^+$: 329.1180, found 329.1183.

$[\alpha]_D^{25}$ = –225.7 (*c* 0.47, CH_2Cl_2).

The enantiomeric excess was determined by chiral HPLC analysis on IE-3 column. Conditions: heptane/isopropanol = 98:2, flow rate = 1.0 mL/min, uv-vis detection at λ = 220 nm, t_{R1} = 5.2 min (minor), t_{R2} =9.2 min (major).

(*R*,*E*)-5-methoxy-5-oxo-2-phenethyl-4-(trimethylsilyl)pent-3-enoic acid (4l)

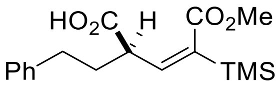

The reaction was performed according to the general procedure and after purification process afforded 56.3 mg (88%, 95:5 er) of **4l** as a colorless oil:

^{1}H NMR (501 MHz, $CDCl_3$) δ 11.02 (br s, 1H), 7.28–7.24 (m, 2H), 7.21–7.16 (m, 1H), 7.13 (dd, *J* = 8.2, 1.4 Hz, 2H), 6.13 (d, *J* = 9.5 Hz, 1H), 3.79 (s, 3H), 3.58 (ddd, *J* = 9.6, 7.9, 6.4 Hz, 1H), 2.69–2.55 (m, 2H), 2.29–2.20 (m, 1H), 2.00–1.91 (m, 1H), 0.17 (s, 9H).

^{13}C NMR (126 MHz, $CDCl_3$) δ 173.2, 172.6, 149.9, 140.9, 139.9, 128.6, 128.6, 126.3, 52.6, 47.8, 33.1, 32.8, –1.3.

HRMS m/z (ESI): calculated for $C_{17}H_{24}O_4SiNa$ $[M+Na]^+$: 343.1336, found 343.1337.

$[\alpha]_D^{25}$ = –245.4 (*c* 0.73, CH_2Cl_2).

The enantiomeric excess was determined by chiral HPLC analysis on IE-3 column. Conditions: heptane/isopropanol = 98:2, flow rate = 1.0 mL/min, uv-vis detection at λ = 220 nm, t_{R1} = 5.1 min (minor), t_{R2} = 8.3 min (major).

(*R*,*E*)-5-methoxy-5-oxo-2-(3-phenylpropyl)-4-(trimethylsilyl)pent-3-enoic acid (4m)

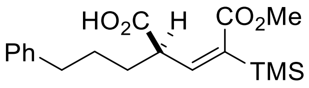

The reaction was performed according to the general procedure and after purification process afforded 50.0 mg (72%, 96:4 er) of **4m** as a colorless oil:

^{1}H NMR (501 MHz, $CDCl_3$) δ 11.02 (br s, 1H), 7.30–7.26 (m, 2H), 7.20–7.12 (m, 3H), 6.10 (d, *J* = 9.5 Hz, 1H), 3.81 (s, 3H), 3.70–3.63 (m, 1H), 2.68–2.56 (m, 2H), 2.00–1.89 (m, 1H), 1.70–1.53 (m, 3H), 0.16 (s, 9H).

^{13}C NMR (126 MHz, $CDCl_3$) δ 173.6, 172.7, 150.0, 142.0, 139.3, 128.5, 126.0, 52.5, 48.3, 35.7, 31.3, 28.9, –1.3.

HRMS m/z (ESI): calculated for $C_{18}H_{26}O_4SiNa$ $[M+Na]^+$: 357.1493, found 357.1494.

$[\alpha]_D^{25}$ = –259.3 (*c* 0.27, CH_2Cl_2).

The enantiomeric excess was determined by chiral HPLC analysis on IE-3 column. Conditions: heptane/isopropanol = 98:2, flow rate = 1.0 mL/min, uv-vis detection at λ = 220 nm, t_{R1} = 7.0 min (minor), t_{R2} =8.1 min (major).

(*R*,*E*)-2-(furan-2-ylmethyl)-5-methoxy-5-oxo-4-(trimethylsilyl)pent-3-enoic acid (4n)

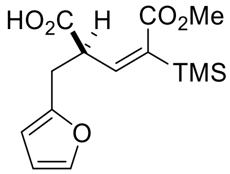

The reaction was performed according to the general procedure and after purification process afforded 45.2 mg (76%, 95.5:4.5 er) of **4n** as a colorless oil:

^{1}H NMR (501 MHz, $CDCl_3$) δ 7.27 (dd, *J* = 1.9, 0.9 Hz, 1H), 6.25 (dd, *J* = 3.2, 1.8 Hz, 1H), 6.18 (d, *J* = 9.3 Hz, 1H), 6.03 (dd, *J* = 3.2, 0.8 Hz, 1H), 4.04 (ddd, *J* = 9.3, 8.1, 6.1 Hz, 1H), 3.84 (s, 3H), 3.25 (ddd, *J* = 15.4, 6.2, 0.9 Hz, 1H), 3.02 (dd, *J* = 15.3, 8.2 Hz, 1H), 0.13 (s, 9H).

^{13}C NMR (126 MHz, $CDCl_3$) δ 173.1 171.4, 152.1, 149.9, 141.7, 139.7, 110.4, 107.0, 52.8, 47.6, 29.9, –1.4.

HRMS m/z (ESI): calculated for $C_{14}H_{20}O_5SiNa$ $[M+Na]^+$: 319.0972, found 319.0973.

$[\alpha]_D^{25}$ = –265.6 (*c* 1.15, CH_2Cl_2).

The enantiomeric excess was determined by chiral HPLC analysis on IE-3 column. Conditions: heptane/isopropanol = 98:2, flow rate = 1.0 mL/min, uv-vis detection at λ = 220 nm, t_{R1} = 8.3 min (minor), t_{R2} = 9.8 min (major).

(*R*,*E*)-5-methoxy-5-oxo-2-(2-(thiophen-2-yl)ethyl)-4-(trimethylsilyl)pent-3-enoic acid (4o)

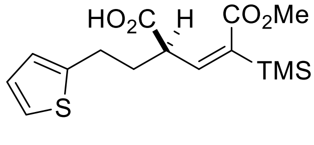

The reaction was performed according to the general procedure and after purification process afforded 61.2 mg (94%, 96.5:3.5 er) of **4o** as a colorless oil:

^{1}H NMR (501 MHz, $CDCl_3$) δ 11.17 (br s, 1H), 7.12 (dd, *J* = 5.2, 1.3 Hz, 1H), 6.90 (dd, *J* = 5.1, 3.4 Hz, 1H), 6.75 (dd, *J* = 3.5, 1.1 Hz, 1H), 6.12 (d, *J* = 9.5 Hz, 1H), 3.79 (s, 3H), 3.69 (ddd, *J* = 9.5, 7.7, 6.4 Hz, 1H), 2.85 (t, *J* = 7.6 Hz, 2H), 2.32–2.23 (m, 1H), 2.04–1.95 (m, 1H), 0.17 (s, 9H).

^{13}C NMR (126 MHz, $CDCl_3$) δ 173.6, 172.7, 148.9, 143.5, 140.1, 126.9, 124.8, 123.6, 52.5, 47.5, 33.3, 27.3, –1.3.

HRMS m/z (ESI): calculated for $C_{15}H_{22}O_4SSiNa$ $[M+Na]^+$: 349.0900, found 349.0903.

$[\alpha]_D^{25}$ = –210.7 (*c* 1.85, CH_2Cl_2).

The enantiomeric excess was determined by chiral HPLC analysis on IE-3 column. Conditions: heptane/isopropanol = 98:2, flow rate = 1.0 mL/min, uv-vis detection at λ = 220 nm, t_{R1} = 8.3 min (minor), t_{R2} = 9.5 min (major).

(*R*,*E*)-5-methoxy-2-methyl-5-oxo-4-(trimethylsilyl)pent-3-enoic acid (4p)

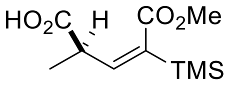

The reaction was performed according to the general procedure and after purification process afforded 42.9 mg (93%, 97:3 er) of **4p** as a colorless oil:

^{1}H NMR (501 MHz, $CDCl_3$) δ 6.14 (d, *J* = 9.3 Hz, 1H), 3.82 (s, 3H), 3.78 (dq, *J* = 9.3, 6.8 Hz, 1H), 1.32 (d, *J* = 6.8 Hz, 3H), 0.16 (s, 9H).

^{13}C NMR (126 MHz, $CDCl_3$) δ 174.7, 172.7, 151.0, 138.2, 52.4, 42.6, 17.0, –1.4.

HRMS m/z (ESI): calculated for $C_{10}H_{18}O_4SiNa$ $[M+Na]^+$: 253.0867, found 253.0869.

$[\alpha]_D^{25}$ = –418.9 (*c* 0.51, CH_2Cl_2).

The enantiomeric excess was determined by chiral HPLC analysis on IE-3 column. Conditions: heptane/isopropanol = 98:2, flow rate = 0.5 mL/min, uv-vis detection at λ = 220 nm, t_{R1} = 11.3 min (minor), t_{R2} = 12.1 min (major).

(*R*,*E*)-2-ethyl-5-methoxy-5-oxo-4-(trimethylsilyl)pent-3-enoic acid (4q)

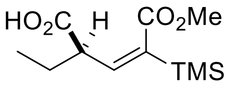

The reaction was performed according to the general procedure and after purification process afforded 45.5 mg (99%, 97.5:2.5 er) of **4q** as a colorless oil:

^{1}H NMR (501 MHz, $CDCl_3$) δ 6.13 (d, *J* = 9.6 Hz, 1H), 3.81 (s, 3H), 3.59 (dt, *J* = 9.5, 7.1 Hz, 1H), 1.93 (dp, *J* = 14.7, 7.4 Hz, 1H), 1.64 (dt, *J* = 13.6, 7.4 Hz, 1H), 0.92 (t, *J* = 7.5 Hz, 3H), 0.16 (s, 9H).

^{13}C NMR (126 MHz, $CDCl_3$) δ 174.8, 172.4, 149.6, 139.2, 52.3, 49.7, 25.3, 11.7, –1.3.

HRMS m/z (ESI): calculated for $C_{11}H_{20}O_4SiNa$ $[M+Na]^+$: 267.1023, found 267.1026.

$[\alpha]_D^{25}$ = –346.1 (*c* 0.68, CH_2Cl_2).

The enantiomeric excess was determined by chiral HPLC analysis on IE-3 column. Conditions: heptane/isopropanol = 98:2, flow rate = 1.0 mL/min, uv-vis detection at λ = 220 nm, t_{R1} = 5.5 min (minor), t_{R2} = 6.2 min (major).

(*R*,*E*)-5-methoxy-5-oxo-2-propyl-4-(trimethylsilyl)pent-3-enoic acid (4r)

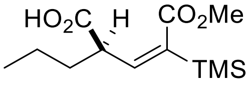

The reaction was performed according to the general procedure and after purification process afforded 49.3 mg (95%, 97.5:2.5 er) of **4r** as a colorless oil:

^{1}H NMR (501 MHz, $CDCl_3$) δ 6.13 (d, *J* = 9.5 Hz, 1H), 3.81 (s, 3H), 3.67 (dt, *J* = 9.5, 7.1 Hz, 1H), 1.92–1.83 (m, 1H), 1.63–1.54 (m, 1H), 1.36–1.26 (m, 2H), 0.91 (t, *J* = 7.4 Hz, 3H), 0.16 (s, 9H).

^{13}C NMR (126 MHz, $CDCl_3$) δ 174.4, 172.5, 149.9, 139.0, 52.3, 48.1, 34.0, 20.4, 14.0, –1.3.

HRMS m/z (ESI): calculated for $C_{12}H_{22}O_4SiNa$ $[M+Na]^+$: 281.1180, found 281.1182.

$[\alpha]_D^{25}$ = –301.7 (*c* 1.12, CH_2Cl_2).

The enantiomeric excess was determined by chiral HPLC analysis on IE-3 column. Conditions: heptane/isopropanol = 98:2, flow rate = 1.0 mL/min, uv-vis detection at λ = 220 nm, t_{R1} = 5.7 min (minor), t_{R2} = 6.3 min (major).

(*R*,*E*)-2-(3-methoxy-3-oxo-2-(trimethylsilyl)prop-1-en-1-yl)hexanoic acid (4s)

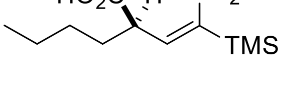

The reaction was performed according to the general procedure and after purification process afforded 51.3 mg (94%, 97:3 er) of **4s** as a colorless oil:

^{1}H NMR (501 MHz, $CDCl_3$) δ 11.31 (br s, 1H), 6.13 (d, *J* = 9.5 Hz, 1H), 3.81 (s, 3H), 3.66 (dt, *J* = 9.6, 7.1 Hz, 1H), 1.94–1.83 (m, 1H), 1.64–1.55 (m, 1H), 1.35–1.22 (m, 4H), 0.89 (t, *J* = 7.1 Hz, 3H), 0.16 (s, 9H).

^{13}C NMR (126 MHz, $CDCl_3$) δ 175.0, 172.3, 149.8, 138.9, 52.2, 48.2, 31.7, 29.3, 22.6, 14.0, –1.3.

HRMS m/z (ESI): calculated for $C_{13}H_{24}O_4SiNa$ $[M+Na]^+$: 295.1336, found 295.1336.

$[\alpha]_D^{25}$ = –271.9 (*c* 0.45, CH_2Cl_2).

The enantiomeric excess was determined by chiral HPLC analysis on IE-3 column. Conditions: heptane/isopropanol = 98:2, flow rate = 1.0 mL/min, uv-vis detection at λ = 220 nm, t_{R1} = 5.2 min (minor), t_{R2} = 6.1 min (major).

(*R*,*E*)-2-isobutyl-5-methoxy-5-oxo-4-(trimethylsilyl)pent-3-enoic acid (4t)

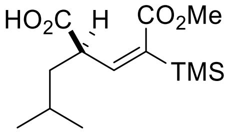

The reaction was performed according to the general procedure and after purification process afforded 41.8 mg (77%, 97.5:2.5 er) of **4t** as a colorless oil:

^{1}H NMR (501 MHz, $CDCl_3$) δ 11.21 (br s, 1H), 6.11 (d, *J* = 9.6 Hz, 1H), 3.82 (s, 3H), 3.76 (dt, *J* = 9.5, 7.2 Hz, 1H), 1.81–1.72 (m, 1H), 1.59–1.47 (m, 2H), 0.92 (d, *J* = 6.5 Hz, 3H), 0.85 (d, *J* = 6.6 Hz, 3H), 0.16 (s, 9H).

^{13}C NMR (126 MHz, $CDCl_3$) δ 174.8, 172.5, 149.9, 138.9, 52.3, 46.5, 40.8, 25.9, 22.8, 22.3, –1.3.

HRMS m/z (ESI): calculated for $C_{13}H_{24}O_4SiNa$ $[M+Na]^+$: 295.1336, found 295.1335.

$[\alpha]_D^{25}$ = –282.2 (*c* 0.65, CH_2Cl_2).

The enantiomeric excess was determined by chiral HPLC analysis on IE-3 column. Conditions: heptane/isopropanol = 98:2, flow rate = 1.0 mL/min, uv-vis detection at λ = 239 nm, t_{R1} = 4.8 min (minor), t_{R2} = 5.4 min (major).

(*R*,*E*)-2-(3-methoxy-3-oxo-2-(trimethylsilyl)prop-1-en-1-yl)-5-methylhexanoic acid (4u)

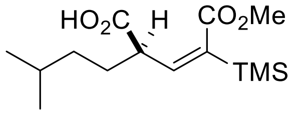

The reaction was performed according to the general procedure and after purification process afforded 55.7 mg (97%, 97:3 er) of **4u** as a colorless oil:

^{1}H NMR (501 MHz, $CDCl_3$) δ 11.10 (br s, 1H), 6.13 (d, *J* = 9.5 Hz, 1H), 3.80 (s, 3H), 3.63 (dt, *J* = 9.5, 7.1 Hz, 1H), 1.94–1.83 (m, 1H), 1.63–1.49 (m, 2H), 1.24–1.07 (m, 2H), 0.88 (dd, *J* = 6.6, 1.1 Hz, 6H), 0.16 (s, 9H).

^{13}C NMR (126 MHz, $CDCl_3$) δ 175.1, 172.3, 149.8, 139.0, 52.2, 48.4, 36.2, 29.9, 28.0, 22.6, 22.6, –1.3.

HRMS m/z (ESI): calculated for $C_{14}H_{26}O_4SiNa$ $[M+Na]^+$: 309.1493, found 309.1495.

$[\alpha]_D^{25}$ = –279.5 (*c* 0.52, CH_2Cl_2).

The enantiomeric excess was determined by chiral HPLC analysis on IE-3 column. Conditions: heptane/isopropanol = 98:2, flow rate = 1.0 mL/min, uv-vis detection at λ = 220 nm, t_{R1} = 4.9 min (minor), t_{R2} = 5.9 min (major).

(*R*,*E*)-2-(cyclopentylmethyl)-5-methoxy-5-oxo-4-(trimethylsilyl)pent-3-enoic acid (4v)

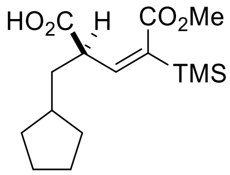

The reaction was performed according to the general procedure and after purification process afforded 53.6 mg (90%, 97.5:2.5 er) of **4v** as a colorless oil:

^{1}H NMR (501 MHz, $CDCl_3$) δ 11.33 (s, 1H), 6.14 (d, *J* = 9.5 Hz, 1H), 3.80 (s, 3H), 3.73 (dt, *J* = 9.5, 7.1 Hz, 1H), 1.94–1.85 (m, 1H), 1.82–1.67 (m, 3H), 1.66–1.55 (m, 3H), 1.54–1.44 (m, 2H), 1.14–1.01 (m, 2H), 0.16 (s, 9H).

^{13}C NMR (126 MHz, $CDCl_3$) δ 175.7, 172.1, 149.6, 138.8, 52.2, 47.7, 38.4, 37.9, 32.8, 32.6, 25.2, 25.2, –1.3.

HRMS m/z (ESI): calculated for $C_{15}H_{26}O_4SiNa$ $[M+Na]^+$: 321.1493, found 321.1496.

$[\alpha]_D^{25}$ = –260.8 (*c* 0.52, CH_2Cl_2).

The enantiomeric excess was determined by chiral HPLC analysis on IE-3 column. Conditions: heptane/isopropanol = 98:2, flow rate = 0.5 mL/min, uv-vis detection at λ = 220 nm, t_{R1} = 11.3 min (minor), t_{R2} = 13.1 min (major).

(*R*,*E*)-5-methoxy-2-(3-methoxypropyl)-5-oxo-4-(trimethylsilyl)pent-3-enoic acid (4w)

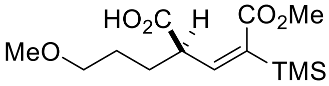

The reaction was performed according to the general procedure and after purification process afforded 37.4 mg (64%, 96.5:3.5 er) of **4w** as a colorless oil:

^{1}H NMR (501 MHz, $CDCl_3$) δ 11.26 (br s, 1H), 6.14 (d, *J* = 9.5 Hz, 1H), 3.80 (s, 3H), 3.71 (dt, *J* = 9.5, 7.1 Hz, 1H), 3.41–3.35 (m, 2H), 3.32 (s, 3H), 2.00–1.88 (m, 1H), 1.73–1.64 (m, 1H), 1.63–1.53 (m, 2H), 0.15 (s, 9H).

^{13}C NMR (126 MHz, $CDCl_3$) δ 174.5, 172.3, 149.5, 139.1, 72.4, 58.7, 52.2, 48.0, 28.9, 27.2, –1.3.

HRMS m/z (ESI): calculated for $C_{13}H_{25}O_5Si$ $[M+H]^+$: 289.1466, found 289.1465.

$[\boldsymbol{\alpha}]_{\boldsymbol{D}}^{\mathbf{25}}$ = –263.3 (*c* 0.77, CH_2Cl_2).

The enantiomeric excess was determined by chiral HPLC analysis on IE-3 column. Conditions: heptane/isopropanol = 98:2, flow rate = 1.0 mL/min, uv-vis detection at λ = 220 nm, t_{R1} = 11.7 min (minor), t_{R2} = 14.1 min (major).

(*R*,*E*)-2-(3-bromopropyl)-5-methoxy-5-oxo-4-(trimethylsilyl)pent-3-enoic acid (4x)

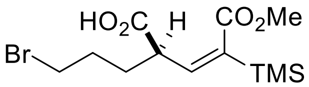

The reaction was performed according to the general procedure and after purification process afforded 60.5 mg (90%, 95.5:4.5 er) of **4x** as a colorless oil:

^{1}H NMR (501 MHz, $CDCl_3$) δ 11.20 (br s, 1H), 6.12 (d, *J* = 9.5 Hz, 1H), 3.83 (s, 3H), 3.70 (dt, *J* = 9.6, 7.1 Hz, 1H), 3.45–3.36 (m, 2H), 2.08–2.00 (m, 1H), 1.97–1.89 (m, 1H), 1.86–1.74 (m, 2H), 0.17 (s, 9H).

^{13}C NMR (126 MHz, $CDCl_3$) δ 173.7, 172.5, 149.0, 139.7, 52.5, 47.5, 33.1, 30.4, 30.3, –1.3.

HRMS m/z (ESI): calculated for $C_{12}H_{21}BrO_4SiNa$ $[M+Na]^+$: 359.0285, found 359.0287.

$[\boldsymbol{\alpha}]_{\boldsymbol{D}}^{\mathbf{25}}$ = –236.7 (*c* 0.74, CH_2Cl_2).

The enantiomeric excess was determined by chiral HPLC analysis on IE-3 column. Conditions: heptane/isopropanol = 98:2, flow rate = 0.5 mL/min, uv-vis detection at λ = 220 nm, t_{R1} = 12.7 min (minor), t_{R2} = 15.6 min (major).

(*R,E*)-2-(3-methoxy-3-oxo-2-(trimethylsilyl)prop-1-en-1-yl)hex-5-enoic acid (4y)

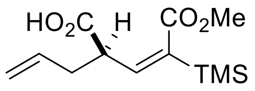

The reaction was performed according to the general procedure and after purification process afforded 46.6 mg (91%, 97:3 er) of **4y** as a colorless oil:

^{1}H NMR (501 MHz, $CDCl_3$) δ 10.79 (br s, 1H), 6.15 (d, *J* = 9.3 Hz, 1H), 5.72 (ddt, *J* = 17.2, 10.2, 6.9 Hz, 1H), 5.11–5.01 (m, 2H), 3.80 (s, 3H), 2.67–2.57 (m, 1H), 2.45–2.35 (m, 1H), 0.15 (s, 9H).

^{13}C NMR (126 MHz, $CDCl_3$) δ 174.5, 172.2, 149.0, 139.3, 134.4, 117.7, 52.3, 47.6, 36.2, –1.3.

HRMS m/z (ESI): calculated for $C_{12}H_{20}O_4SiNa$ $[M+Na]^+$: 279.1023, found 279.1022.

$[\alpha]_D^{25}$ = –320.6 (*c* 0.36, CH_2Cl_2).

The enantiomeric excess was determined by chiral HPLC analysis on IE-3 column. Conditions: heptane/isopropanol = 98:2, flow rate = 1.0 mL/min, uv-vis detection at λ = 220 nm, t_{R1} = 5.7 min (minor), t_{R2} = 6.5 min (major).

(2*R*,4*S*)-2-((*E*)-3-methoxy-3-oxo-2-(trimethylsilyl)prop-1-en-1-yl)-4-methylhexanoic acid (4za)

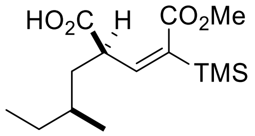

The reaction was performed according to the general procedure and after purification process afforded 46.3 mg (99%, 99:1 er) of **4za** as a colorless oil:

^{1}H NMR (501 MHz, $CDCl_3$) δ 11.09 (br s, 1H), 6.14 (d, *J* = 9.5 Hz, 1H), 3.82 (s, 3H), 3.83–3.76 (m, 1H), 1.98–1.86 (m, 1H), 1.40–1.27 (m, 3H), 1.16–1.06 (m, 1H), 0.88 (d, *J* = 6.3 Hz, 3H), 0.86 (t, *J* = 7.4 Hz, 3H)., 0.16 (s, 9H).

^{13}C NMR (126 MHz, $CDCl_3$) δ 174.5, 172.4, 150.2, 138.5, 52.3, 46.4, 38.9, 32.3, 29.3, 19.3, 11.2, –1.3.

HRMS m/z (ESI): calculated for $C_{14}H_{26}O_4SiNa$ $[M+Na]^+$: 309.1493, found 309.1491.

$[\alpha]_D^{25}$ = –312.9 (*c* 0.50, CH_2Cl_2).

The enantiomeric excess was determined by chiral HPLC analysis on IE-3 column. Conditions: heptane/isopropanol = 98:2, flow rate = 1.0 mL/min, uv-vis detection at λ = 220 nm, t_{R1} = 5.9 min (minor), t_{R2} = 6.8 min (major).

(*2R,4R*)-2-((*E*)-3-methoxy-3-oxo-2-(trimethylsilyl)prop-1-en-1-yl)-4-methylhexanoic acid (4zb)

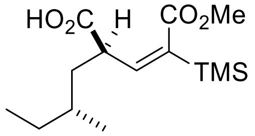

The reaction was performed according to the general procedure and after purification process afforded 55.7 mg (97%, 98:2 er) of **4zb** as a colorless oil:

^{1}H NMR (501 MHz, $CDCl_3$) δ 11.10 (br s, 1H), 6.09 (d, *J* = 9.6 Hz, 1H), 3.82 (s, 3H), 3.77 (dt, *J* = 9.5, 7.3 Hz, 1H), 1.67 (t, *J* = 7.1 Hz, 2H), 1.42–1.11 (m, 4H), 0.87 (t, *J* = 7.3 Hz, 3H), 0.81 (d, *J* = 6.5 Hz, 3H), 0.16 (s, 9H).

^{13}C NMR (126 MHz, $CDCl_3$) δ 174.5, 172.6, 150.0, 139.2, 52.4, 46.3, 38.4, 32.2, 29.7, 18.9, 11.3, –1.3.

HRMS m/z (ESI): calculated for $C_{14}H_{26}O_4SiNa$ $[M+Na]^+$: 309.1493, found 309.1490.

$[\alpha]_D^{25}$ = –374.8 (*c* 0.38, CH_2Cl_2).

The enantiomeric excess was determined by chiral HPLC analysis on IE-3 column. Conditions: heptane/isopropanol = 98:2, flow rate = 1.0 mL/min, uv-vis detection at λ = 220 nm, t_{R1} = 4.6 min (minor), t_{R2} = 5.4 min (major).

Table S6. Additional Substrate Scope

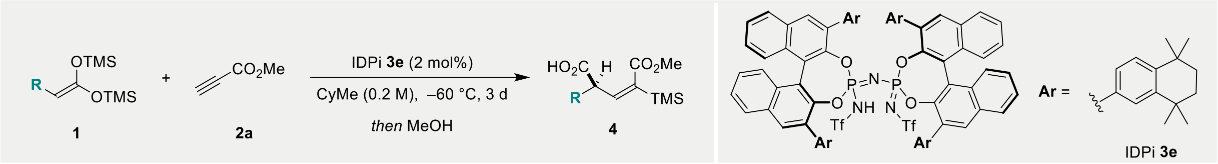

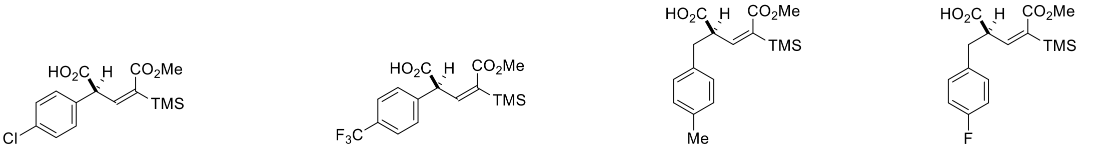

s4a: 74%, >99.5:0.5 er | **s4b**: 86%, 99:1 er | **s4c**: 61%, 95:5 er | **s4d**: 98%, 94:6 er

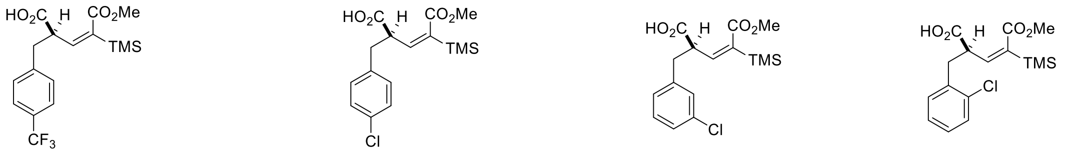

s4e: 77%, 94:6 er | **s4f**: 87%, 95:5 er | **s4g**: 90%, 95:5 er | **s4h**: 93%, 95:5 er

Reactions were conducted on a 0.2 mmol scale with isolated yield.

(*S*,*E*)-2-(4-chlorophenyl)-5-methoxy-5-oxo-4-(trimethylsilyl)pent-3-enoic acid (s4a)

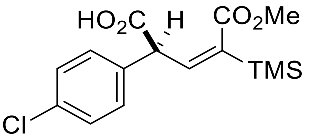

^{1}H NMR (501 MHz, $CDCl_3$) δ 7.35–7.28 (m, 4H), 6.51 (d, *J* = 9.3 Hz, 1H), 5.07 (d, *J* = 9.5 Hz, 1H), 3.78 (s, 3H), 0.16 (s, 9H).

^{13}C NMR (126 MHz, $CDCl_3$) δ 173.8, 171.0, 147.4, 138.8, 135.7, 133.9, 129.8, 129.2, 52.1, 52.0, –1.3.

HRMS m/z (ESI): calculated for $C_{15}H_{19}O_4ClSiNa$ $[M+Na]^+$: 349.0633, found 349.0632.

$[\alpha]_D^{25}$ = +58.7 (*c* 1.37, CH_2Cl_2).

The enantiomeric excess was determined by chiral HPLC analysis on IE-3 column. Conditions: heptane/isopropanol = 98:2, flow rate = 1.0 mL/min, uv-vis detection at λ = 220 nm, t_{R1} = 6.3 min (minor), t_{R2} = 7.1 min (major). >99.5:0.5 er

(*S*,*E*)-5-methoxy-5-oxo-2-(4-(trifluoromethyl)phenyl)-4-(trimethylsilyl)pent-3-enoic acid (s4b)

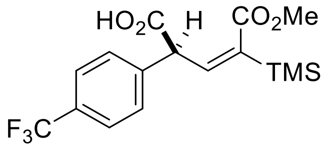

The reaction was performed according to the general procedure and after purification process afforded 62.0 mg (95%, 99:1 er) of **s4b** as a colorless oil:

^{1}H NMR (501 MHz, $CDCl_3$) δ 7.61 (d, *J* = 8.0 Hz, 2H), 7.49 (d, *J* = 8.4 Hz, 2H), 6.55 (d, *J* = 9.3 Hz, 1H), 5.18 (d, *J* = 9.3 Hz, 1H), 3.77 (s, 3H), 0.16 (s, 9H).

^{13}C NMR (126 MHz, $CDCl_3$) δ 174.7, 170.6, 146.5, 141.2, 139.3, 128.8, 126.0 (q, *J* = 4.0 Hz), 52.3, 51.9, –1.4.

^{19}F NMR (471 MHz, $CDCl_3$) δ –62.66.

HRMS m/z (ESI): calculated for $C_{16}H_{18}O_4F_3Si$ $[M–H]^-$: 359.0932, found 359.0930.

$[\alpha]_D^{25}$ = –18.9 (*c* 0.11, CH_2Cl_2).

The enantiomeric excess was determined by chiral HPLC analysis on IE-3 column. Conditions: heptane/isopropanol = 98:2, flow rate = 1.0 mL/min, uv-vis detection at λ = 220 nm, t_{R1} = 8.3 min (minor), t_{R2} = 9.6 min (major). 99:1 er

(*R*,*E*)-5-methoxy-2-(4-methylbenzyl)-5-oxo-4-(trimethylsilyl)pent-3-enoic acid (s4c)

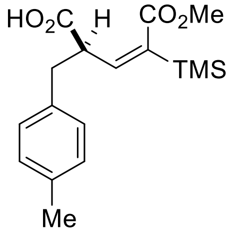

^{1}H NMR (501 MHz, $CDCl_3$) δ 11.20 (br s, 1H), 7.05 (q, *J* = 8.1 Hz, 4H), 6.20 (d, *J* = 9.5 Hz, 1H), 4.02 (dt, *J* = 9.5, 7.1 Hz, 1H), 3.75 (s, 3H), 3.19 (dd, *J* = 13.9, 6.9 Hz, 1H), 2.89 (dd, *J* = 13.9, 7.3 Hz, 1H), 2.30 (s, 3H), 0.11 (s, 9H).

^{13}C NMR (126 MHz, $CDCl_3$) δ 174.3, 172.0, 149.4, 139.2, 136.2, 135.0, 129.2, 129.1, 52.2, 49.8, 37.6, 21.2, –1.4.

HRMS m/z (ESI): calculated for $C_{17}H_{24}O_4SiNa$ $[M+Na]^+$: 343.1336, found 343.1340.

$[\alpha]_D^{25}$ = –227.6 (*c* 0.10, CH_2Cl_2).

The enantiomeric excess was determined by chiral HPLC analysis on IE-3 column. Conditions: heptane/isopropanol = 98:2, flow rate = 1.0 mL/min, uv-vis detection at λ = 220 nm, t_{R1} = 7.9 min (minor), t_{R2} = 11.9 min (major). 95:5 er

(*R*,*E*)-2-(4-fluorobenzyl)-5-methoxy-5-oxo-4-(trimethylsilyl)pent-3-enoic acid (s4d)

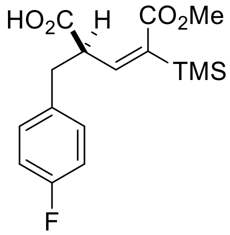

^{1}H NMR (501 MHz, Chloroform-*d*) δ 10.87 (br s, 1H), 7.12 (dd, *J* = 8.7, 5.4 Hz, 2H), 6.95 (t, *J* = 8.7 Hz, 2H), 6.17 (d, *J* = 9.5 Hz, 1H), 3.98 (dt, *J* = 9.6, 7.2 Hz, 1H), 3.77 (s, 3H), 3.22 (dd, *J* = 14.0, 7.1 Hz, 1H), 2.89 (dd, *J* = 14.0, 7.4 Hz, 1H), 0.11 (s, 9H).

^{19}F NMR (471 MHz, $CDCl_3$) δ –116.39.

^{13}C NMR (126 MHz, $CDCl_3$) δ 174.3, 171.9, 161.9 (d, *J* = 244.2 Hz), 148.6, 139.6, 133.7 (d, *J* = 3.7 Hz), 130.7 (d, *J* = 7.9 Hz), 115.4 (d, *J* = 21.2 Hz), 52.2, 49.7, 37.2, –1.4.

HRMS m/z (ESI): calculated for $C_{16}H_{21}O_4F_1SiNa$ $[M+Na]^+$: 347.1085, found 347.1084.

$[\alpha]_D^{25}$ = –249.1 (*c* 0.57, CH_2Cl_2).

The enantiomeric excess was determined by chiral HPLC analysis on IE-3 column. Conditions: heptane/isopropanol = 98:2, flow rate = 1.0 mL/min, uv-vis detection at λ = 220 nm, t_{R1} = 6.6 min (minor), t_{R2} = 8.5 min (major). 94:6 er

(*R*,*E*)-5-methoxy-5-oxo-2-(4-(trifluoromethyl)benzyl)-4-(trimethylsilyl)pent-3-enoic acid (s4e)

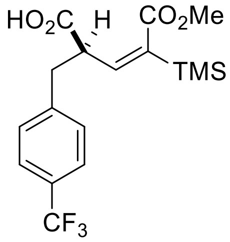

^{1}H NMR (501 MHz, $CDCl_3$) δ 10.84 (br s, 1H), 7.52 (d, *J* = 8.0 Hz, 2H), 7.28 (d, *J* = 7.9 Hz, 2H), 6.17 (d, *J* = 9.5 Hz, 1H), 4.05 (dt, *J* = 9.5, 7.3 Hz, 1H), 3.77 (s, 3H), 3.31 (dd, *J* = 14.0, 7.2 Hz, 1H), 2.96 (dd, *J* = 14.0, 7.3 Hz, 1H), 0.11 (s, 9H).

^{13}C NMR (126 MHz, $CDCl_3$) δ 172.7, 172.3, 148.8, 142.3, 140.0, 129.6, 125.5 (q, J = 4.0 Hz), 52.5, 49.5, 37.5, –1.4.

^{19}F NMR (471 MHz, $CDCl_3$) δ –62.52.

HRMS m/z (ESI): calculated for $C_{17}H_{21}O_4F_3SiNa$ $[M+Na]^+$: 397.1053, found 397.1053.

$[\alpha]_D^{25}$ = –22.3 (c 0.50, CH_2Cl_2).

The enantiomeric excess was determined by chiral HPLC analysis on IE-3 column. Conditions: heptane/isopropanol = 98:2, flow rate = 1.0 mL/min, uv-vis detection at λ = 220 nm, t_{R1} = 4.5 min (minor), t_{R2} = 5.8 min (major). 94:6 er

(*R*,*E*)-2-(4-chlorobenzyl)-5-methoxy-5-oxo-4-(trimethylsilyl)pent-3-enoic acid (s4f)

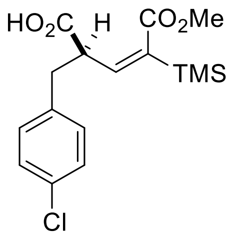

^{1}H NMR (501 MHz, $CDCl_3$) δ 7.23 (d, J = 8.5 Hz, 2H), 7.09 (d, J = 8.5 Hz, 2H), 6.17 (d, J = 9.3 Hz, 1H), 4.01 (dt, J = 9.5, 7.2 Hz, 1H), 3.76 (s, 3H), 3.20 (dd, J = 13.9, 7.1 Hz, 1H), 2.88 (dd, J = 13.9, 7.3 Hz, 1H), 0.12 (s, 9H).

^{13}C NMR (126 MHz, $CDCl_3$) δ 173.7, 172.0, 148.7, 139.8, 136.6, 132.6, 130.6, 128.7, 52.3, 49.6, 37.2, –1.4.

HRMS m/z (ESI): calculated for $C_{16}H_{21}O_4Cl_1SiNa$ $[M+Na]^+$: 363.0790, found 363.0788.

$[\alpha]_D^{25}$ = –218.3 (c 0.97, CH_2Cl_2).

The enantiomeric excess was determined by chiral HPLC analysis on IE-3 column. Conditions: heptane/isopropanol = 98:2, flow rate = 1.0 mL/min, uv-vis detection at λ = 220 nm, t_{R1} = 6.7 min (minor), t_{R2} = 8.6 min (major). 95:5 er

(*R*,*E*)-2-(3-chlorobenzyl)-5-methoxy-5-oxo-4-(trimethylsilyl)pent-3-enoic acid (s4g)

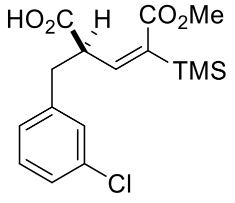

^{1}H NMR (501 MHz, $CDCl_3$) δ 7.22–7.15 (m, 2H), 7.14 (q, *J* = 1.7 Hz, 1H), 7.04 (ddd, *J* = 6.1, 2.8, 1.7 Hz, 1H), 6.17 (d, *J* = 9.5 Hz, 1H), 4.01 (dt, *J* = 9.5, 7.4 Hz, 1H), 3.76 (s, 3H), 3.21 (dd, *J* = 14.0, 6.9 Hz, 1H), 2.89 (dd, *J* = 13.9, 7.6 Hz, 1H), 0.12 (s, 9H).

^{13}C NMR (126 MHz, $CDCl_3$) δ 173.5, 172.1, 148.5, 140.1, 139.9, 134.3, 129.8, 129.4, 127.5, 127.0, 52.4, 49.4, 37.5, –1.4.

HRMS m/z (ESI): calculated for $C_{16}H_{21}O_4Cl_1SiNa$ $[M+Na]^+$: 363.0790, found 363.0788.

$[\alpha]_D^{25}$ = –244.9 (*c* 0.57, CH_2Cl_2).

The enantiomeric excess was determined by chiral HPLC analysis on IE-3 column. Conditions: heptane/isopropanol = 98:2, flow rate = 1.0 mL/min, uv-vis detection at λ = 220 nm, t_{R1} = 10.4 min (minor), t_{R2} = 11.9 min (major). 95:5 er

(*R*,*E*)-2-(2-chlorobenzyl)-5-methoxy-5-oxo-4-(trimethylsilyl)pent-3-enoic acid (s4h)

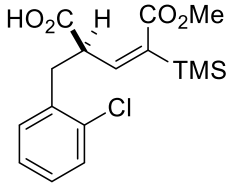

^{1}H NMR (501 MHz, $CDCl_3$) δ 10.91 (br s, 1H), 7.35–7.29 (m, 1H), 7.25–7.21 (m, 1H), 7.19–7.13 (m, 2H), 6.20 (d, *J* = 9.6 Hz, 1H), 4.15 (ddd, *J* = 9.6, 8.0, 6.5 Hz, 1H), 3.76 (s, 3H), 3.35 (dd, *J* = 13.9, 6.6 Hz, 1H), 3.09 (dd, *J* = 13.9, 8.0 Hz, 1H), 0.09 (s, 9H).

^{13}C NMR (126 MHz, $CDCl_3$) δ 172.9, 172.2, 148.8, 139.9, 135.9, 134.3, 131.8, 129.7, 128.3, 126.9, 52.5, 48.1, 35.1, –1.5.

HRMS m/z (ESI): calculated for $C_{16}H_{21}O_4Cl_1SiNa$ $[M+Na]^+$: 363.0790, found 363.0788.

$[\alpha]_D^{25}$ = –250.4 (*c* 0.46, CH_2Cl_2).

The enantiomeric excess was determined by chiral HPLC analysis on IE-3 column. Conditions: heptane/isopropanol = 98:2, flow rate = 1.0 mL/min, uv-vis detection at λ = 220 nm, t_{R1} = 10.3 min (minor), t_{R2} = 11.9 min (major). 95:5 er

7. Downstream Transformations

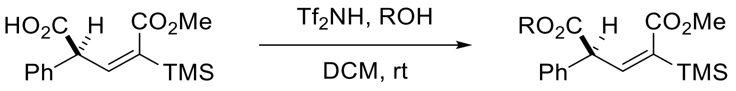

4a
>99.5:0.5 er

5a, R = Me, 98%, >99.5:0.5 er
5b, R = Bn, 91%, >99.5:0.5 er

The **4a** (58.5 mg, 0.2 mmol, 1.0 equiv.) and Tf_2NH (5.6 mg, 0.02 mmol, 0.1 equiv.) were dissolved in DCM (0.1 mL) under argon. MeOH (0.01 mL, 0.6 mmol, 3.0 equiv.) or BnOH (2.76 mL, 2.76 mmol, 3.0 equiv.) was added and the reaction solution was stirred at rt for 16 h. After full conversion of **4a**, the solvent was evaporated. The residue was purified by column chromatography on silica gel (eluent: EtOAc) to afford 60.3 mg (98% yield) of **5a** or 69.8 mg (91% yield) of **5b** as a colourless oil.

dimethyl (*S*,*E*)-4-phenyl-2-(trimethylsilyl)pent-2-enedioate (5a)

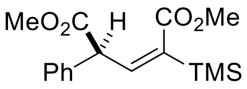

^{1}H NMR (501 MHz, $CDCl_3$) δ 7.36–7.31 (m, 4H), 7.30–7.25 (m, 1H), 6.62 (d, J = 9.5 Hz, 1H), 5.10 (d, J = 9.3 Hz, 1H), 3.74 (s, 3H), 3.69 (s, 3H), 0.15 (s, 9H).

^{13}C NMR (126 MHz, $CDCl_3$) δ 172.6, 170.0, 147.5, 138.2, 137.7, 129.0, 128.0, 127.6, 52.5, 52.3, 51.4, –1.3.

HRMS m/z (ESI): calculated for $C_{16}H_{22}O_4Si_1Na_1$ $[M+Na]^+$: 329.1180, found 329.1179.

$[\alpha]_D^{25}$ = +142.5 (*c* 0.52, CH_2Cl_2).

The enantiomeric excess was determined by chiral HPLC analysis on IE-3 column. Conditions: heptane/isopropanol = 99:1, flow rate = 1.0 mL/min, uv-vis detection at λ = 220 nm, t_{R1} = 4.3 min (minor), t_{R2} = 4.7 min (major). >99.5:0.5 er

5-benzyl 1-methyl (*S*,*E*)-4-phenyl-2-(trimethylsilyl)pent-2-enedioate (5b)

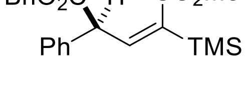

^{1}H NMR (501 MHz, $CDCl_3$) δ 7.33–7.22 (m, 10H), 6.63 (d, J = 9.3 Hz, 1H), 5.15 (d, J = 9.3 Hz, 1H), 5.14 (s, 2H), 3.70 (s, 3H), 0.15 (s, 9H).

^{13}C NMR (126 MHz, $CDCl_3$) δ 171.8, 170.0, 147.4, 138.1, 137.9, 135.9, 129.0, 128.6, 128.2, 128.1, 128.0, 127.6, 66.9, 52.5, 51.4, –1.3.

HRMS m/z (ESI): calculated for $C_{22}H_{26}O_4Si_1Na_1$ $[M+Na]^+$: 405.1493, found 405.1489.

$[\alpha]_D^{25}$ = +16.6 (*c* 0.42, CH_2Cl_2).

The enantiomeric excess was determined by chiral HPLC analysis on IE-3 column. Conditions: heptane/isopropanol = 99:1, flow rate = 1.0 mL/min, uv-vis detection at λ = 220 nm, t_{R1} = 6.0 min (minor), t_{R2} = 7.2 min (major). >99.5:0.5 er

(*S*,*E*)-2-(2-acetoxyphenyl)-5-methoxy-5-oxo-4-(trimethylsilyl)pent-3-enoic acid (6)

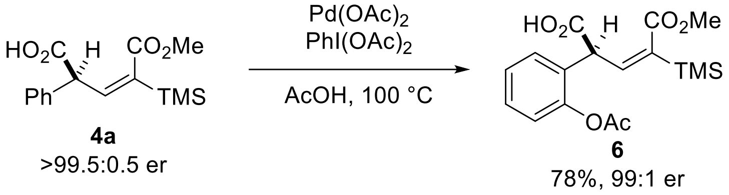

A stirring solution of **4a** (29.2 mg, 0.1 mmol, 1.0 equiv.), $Pd(OAc)_2$ (1.1 mg, 0.005 mmol, 0.05 equiv.), and $PhI(OAc)_2$ (48.3 mg, 0.15 mmol, 1.5 equiv.) in AcOH (2 mL) under argon was stirred at 100 °C for 24 h. The solution was then cooled to rt, the solvent was evaporated and the residue was purified by column chromatography on silica gel to afford 27.4 mg (78% yield) of **6** as a colourless oil.

^{1}H NMR (501 MHz, $CDCl_3$) δ 7.44 (dd, *J* = 7.7, 1.7 Hz, 1H), 7.32 (td, *J* = 7.6, 1.7 Hz, 1H), 7.24 (td, *J* = 7.6, 1.4 Hz, 1H), 7.12 (dd, *J* = 8.0, 1.4 Hz, 1H), 6.55 (d, *J* = 9.0 Hz, 1H), 5.34 (d, *J* = 9.0 Hz, 1H), 3.74 (s, 3H), 2.29 (s, 3H), 0.15 (s, 9H).

^{13}C NMR (126 MHz, $CDCl_3$) δ 174.8, 170.4, 169.3, 148.5, 146.5, 138.8, 129.9, 129.7, 128.9, 126.6, 123.2, 51.7, 47.0, 21.0, –1.3.

HRMS m/z (ESI): calculated for $C_{17}H_{22}O_6Si_1Na_1$ $[M+Na]^+$: 373.1078, found 373.1077.

$[\alpha]_D^{25}$ = –16.4 (*c* 0.09, CH_2Cl_2).

The enantiomeric excess was determined by chiral HPLC analysis on IG-3 column. Conditions: heptane/isopropanol = 95:5, flow rate = 1.0 mL/min, uv-vis detection at λ = 220 nm, t_{R1} = 6.7min (minor), t_{R2} = 8.0 min (major). 99:1 er

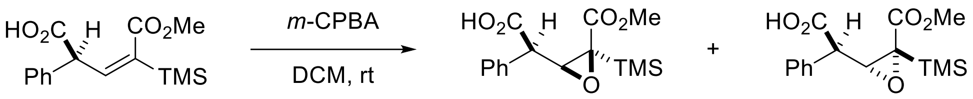

4a >99.5:0.5 er | **7a** 64%, >99.5:0.5 er | **7b** 22%, >99.5:0.5 er

Separable by column chromatography

A stirring solution of **4a** (102 mg, 0.35 mmol, 1.0 equiv.) and MCPBA (129 mg, 70%, 0.525 mmol, 1.5 equiv.) in DCM (3 mL) was stirred at rt for 24 h. The solvent was then evaporated and the residue was purified by column chromatography on silica gel (eluent: EtOAc/hexane/AcOH = 10/90/1) to afford 23.5 mg (22% yield) of **7a** as a white solid and 68.9 mg (64% yield) of **7b** as a white solid.

(*R*)-2-((2*S*,3*R*)-3-(methoxycarbonyl)-3-(trimethylsilyl)oxiran-2-yl)-2-phenylacetic acid (7a)

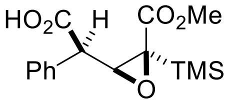

^{1}H NMR (501 MHz, $CDCl_3$) δ 9.23 (br s, 1H), 7.36–7.28 (m, 3H), 7.22–7.17 (m, 2H), 3.67 (d, J = 9.0 Hz, 1H), 3.61 (s, 3H), 3.59 (d, J = 8.8 Hz, 1H), 0.05 (s, 9H).

^{13}C NMR (126 MHz, $CDCl_3$) δ 177.2, 171.1, 134.2, 129.1, 128.4, 127.9, 60.0, 56.2, 52.0, 51.1, –3.4.

HRMS m/z (ESI): calculated for $C_{15}H_{20}O_5Si_1Na_1$ $[M+Na]^+$: 331.0972, found 331.0971.

$[\alpha]_D^{25}$ = +66.5 (c 0.42, CH_2Cl_2).

The enantiomeric excess was determined by chiral HPLC analysis on IG-3 column. Conditions: heptane/isopropanol = 95:5, flow rate = 1.0 mL/min, uv-vis detection at λ = 220 nm, t_{R1} = 5.4 min (minor), t_{R2} = 7.3 min (major). >99.5:0.5 er

(*R*)-2-((2*R*,3*S*)-3-(methoxycarbonyl)-3-(trimethylsilyl)oxiran-2-yl)-2-phenylacetic acid (7b)

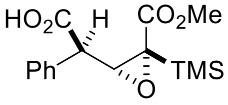

^{1}H NMR (501 MHz, $CDCl_3$) δ 7.41–7.32 (m, 5H), 3.78 (s, 3H), 3.52 (d, J = 9.0 Hz, 1H), 3.47 (d, J = 8.8 Hz, 1H), 0.15 (s, 9H).

^{13}C NMR (126 MHz, $CDCl_3$) δ 176.1, 171.3, 135.2, 129.2, 128.4, 128.2, 60.4, 58.0, 52.3, 51.5, –3.5.

HRMS m/z (ESI): calculated for $C_{15}H_{20}O_5Si_1Na_1$ $[M+Na]^+$: 331.0972, found 331.0970.

The enantiomeric excess was determined by chiral HPLC analysis on IG-3 column. Conditions: heptane/isopropanol = 95:5, flow rate = 1.0 mL/min, uv-vis detection at λ = 220 nm, t_{R1} = 11.2 min (minor), t_{R2} = 12.1 min (major). >99.5:0.5 er

(*S*,*Z*)-4-iodo-5-methoxy-5-oxo-2-phenylpent-3-enoic acid (8)

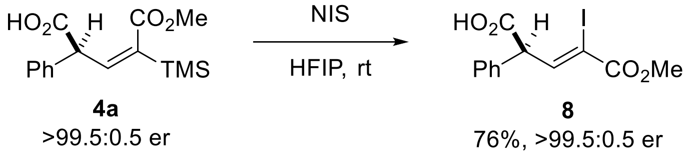

A stirring solution of **4a** (29.2 mg, 0.1 mmol, 1.0 equiv.) and NIS (33.8 mg, 0.15 mmol, 1.5 equiv.) in HFIP (3 mL) under argon was stirred at rt for 1 h. The solvent was then evaporated and the residue was purified by column chromatography on silica gel to afford 26.4 mg (76% yield) of **8** as a pale yellow solid.

^{1}H NMR (501 MHz, $CDCl_3$) δ 7.69 (d, *J* = 9.5 Hz, 1H), 7.41–7.31 (m, 5H), 4.73 (d, *J* = 9.5 Hz, 1H), 3.83 (s, 3H).

^{13}C NMR (126 MHz, $CDCl_3$) δ 175.5, 163.2, 147.9, 135.3, 129.4, 128.5, 128.4, 97.5, 58.3, 54.0.

HRMS m/z (ESI): calculated for $C_{12}H_{11}O_4I_1Na_1$ $[M+Na]^+$: 368.9594, found 368.9593.

$[\alpha]_D^{25}$ = +136.0 (*c* 0.82, CH_2Cl_2).

The enantiomeric excess was determined by chiral HPLC analysis on AD-3 column. Conditions: heptane/isopropanol = 95:5, flow rate = 1.0 mL/min, uv-vis detection at λ = 220 nm, t_{R1} = 11.6 min (major), t_{R2} = 15.3 min (minor). >99.5:0.5 er

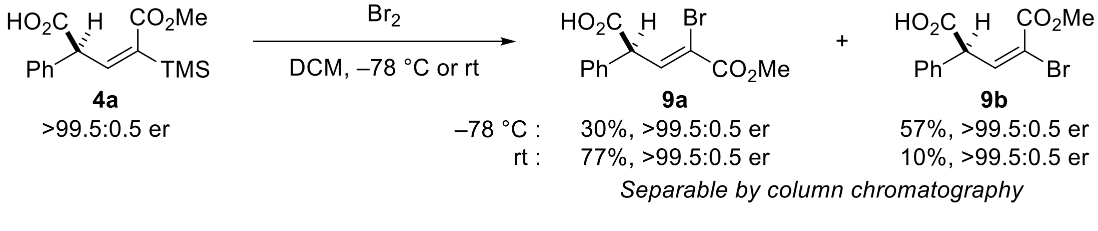

The **4a** (58.5 mg, 0.2 mmol, 1.0 equiv.) was dissolved in DCM (2 mL) under argon. The solution was cooled to –78 °C and Br_2 (0.03 mL, 0.6 mmol, 3 equiv.) was added dropwise. After addition, the reaction solution was stirred at –78 °C for 16 h. The solution was diluted with DCM (2 mL) and quenched with sat. aq. NaS_2O_3 (4 mL). The two layers were separated, and the aqueous layer was extracted with DCM (5 mL*2). The combined organic layers were washed with brine

and dried with Na_2SO_4, and the solvent was evaporated. The residue was purified by column chromatography on silica gel to afford 34.3 mg (57% yield) of **9b** as a pale yellow solid and 17.9 mg (30% yield) of **9a** as a pale yellow solid.

Alternatively, the same reaction was stirred at rt for 3 h. With the same purification, the reaction afforded 6.1 mg (10% yield) of **9b** as a pale yellow solid and 46.2 mg (77% yield) of **9a** as a pale yellow solid.

(*S*,*Z*)-4-bromo-5-methoxy-5-oxo-2-phenylpent-3-enoic acid (9a)

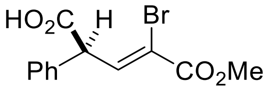

^{1}H NMR (501 MHz, $CDCl_3$) δ 8.68 (br s, 1H), 7.74 (d, J = 9.5 Hz, 1H), 7.39–7.30 (m, 5H), 4.84 (d, J = 9.5 Hz, 1H), 3.84 (s, 3H).

^{13}C NMR (126 MHz, $CDCl_3$) δ 176.2, 162.6, 141.1, 135.3, 129.4, 128.5, 128.3, 118.3, 53.9, 53.7.

HRMS m/z (ESI): calculated for $C_{12}H_{11}O_4Br_1Na_1$ $[M+Na]^+$: 320.9733, found 320.9732.

$[\boldsymbol{\alpha}]_{\boldsymbol{D}}^{\mathbf{25}}$ = +166.3 (c 0.33, CH_2Cl_2).

The enantiomeric excess was determined by chiral HPLC analysis on IG-3 column. Conditions: heptane/isopropanol = 95:5, flow rate = 1.0 mL/min, uv-vis detection at λ = 220 nm, t_{R1} = 9.5 min (major), t_{R2} = 14.1 min (minor). >99.5:0.5 er

(*S*,*E*)-4-bromo-5-methoxy-5-oxo-2-phenylpent-3-enoic acid (9b)

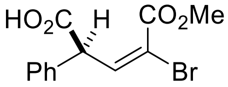

^{1}H NMR (501 MHz, $CDCl_3$) δ 9.30 (br s, 1H), 7.37–7.30 (m, 5H), 7.13 (d, J = 9.8 Hz, 1H), 5.38 (d, J = 9.8 Hz, 1H), 3.83 (s, 3H).

^{13}C NMR (126 MHz, $CDCl_3$) δ 176.4, 162.9, 143.9, 136.4, 129.3, 128.3, 128.2, 113.6, 53.4, 52.0.

HRMS m/z (ESI): calculated for $C_{12}H_{11}O_4Br_1Na_1$ $[M+Na]^+$: 320.9733, found 320.9731.

$[\boldsymbol{\alpha}]_{\boldsymbol{D}}^{\mathbf{25}}$ = +135.3 (c 0.44, CH_2Cl_2).

The enantiomeric excess was determined by chiral HPLC analysis on OZ-3 column. Conditions: heptane/isopropanol = 95:5, flow rate = 1.0 mL/min, uv-vis detection at λ = 220 nm, t_{R1} = 4.1 min (minor), t_{R2} = 6.1 min (major). >99.5:0.5 er

(2*S*,4*S*)-5-methoxy-5-oxo-2-phenyl-4-(trimethylsilyl)pentanoic acid (10)

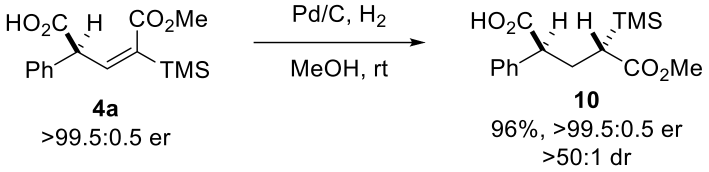

A stirring solution of **4a** (58.5 mg, 0.2 mmol, 1.0 equiv.) and Pd/C (21.3 mg, 5%, 0.01 mmol, 0.05 equiv.) in MeOH (2 mL) under an atmosphere of H_2 (1 atm) was stirred at rt for 16 h. The solution was diluted with Et_2O (5 mL) and filtered over celite. The solvent was then evaporated and the residue was purified by column chromatography on silica gel to afford 56.5 mg (96% yield) of **10** as a colourless oil.

^{1}H NMR (501 MHz, $CDCl_3$) δ 7.35–7.26 (m, 3H), 7.24–7.19 (m, 2H), 3.63 (s, 3H), 3.59 (dd, *J* = 11.0, 4.3 Hz, 1H), 2.50 (ddd, *J* = 14.2, 12.3, 4.3 Hz, 1H), 1.97 (ddd, *J* = 14.0, 11.2, 2.6 Hz, 1H), 1.72 (dd, *J* = 12.3, 2.5 Hz, 1H), 0.01 (s, 9H).

^{13}C NMR (126 MHz, $CDCl_3$) δ 178.8, 175.2, 137.3, 129.0, 128.5, 127.8, 51.3, 50.6, 34.2, 29.5, –2.7.

HRMS m/z (ESI): calculated for $C_{15}H_{22}O_4Si_1Na_1$ $[M+Na]^+$: 317.1180, found 317.1176.

$[\alpha]_D^{25}$ = +101.2 (*c* 0.33, CH_2Cl_2).

The enantiomeric excess was determined by chiral HPLC analysis on IE-3 column. Conditions: heptane/isopropanol = 95:5, flow rate = 1.0 mL/min, uv-vis detection at λ = 220 nm, t_{R1} = 6.3 min (minor), t_{R2} = 6.9 min (major). >99.5:0.5 er

(2*S*,4*S*)-2-phenyl-4-(trimethylsilyl)pentane-1,5-diol (11)

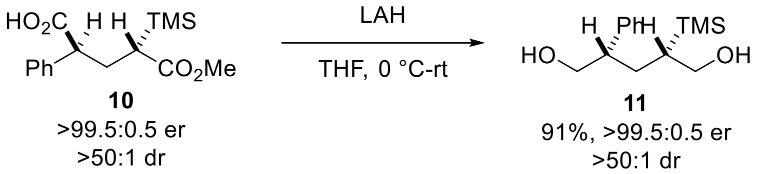

The compound **10** (29.5 mg, 0.1 mmol, 1.0 equiv.) was dissolved in THF (1 mL) under argon. The solution was cooled to 0 °C and LAH (0.2 mL, 1 M in THF, 0.2 mmol, 2.0 equiv.) was added dropwise. After addition, the reaction solution was stirred at rt for 2 h. The solution was quenched with sat. aq. NH_4Cl (1 mL) and 2 M aq. HCl (1 mL) at 0 °C and EtOAc (5 mL) was added. The two layers were separated, and the aqueous layer was extracted with EtOAc (5 mL*2). The

combined organic layers were washed with brine and dried with Na_2SO_4, and the solvent was evaporated. The residue was purified by column chromatography on silica gel to afford 24.8 mg (98% yield) of **11** as a colourless oil, and it will be solidified in pentane.

^{1}H NMR (501 MHz, $CDCl_3$) δ 7.35–7.29 (m, 2H), 7.26–7.19 (m, 3H), 3.85 (dd, J = 10.6, 3.9 Hz, 1H), 3.81–3.72 (m, 2H), 3.66 (dd, J = 10.6, 7.3 Hz, 1H), 2.99 (dq, J = 9.6, 5.9 Hz, 1H), 1.89 (ddd, J = 14.7, 9.4, 5.4 Hz, 1H), 1.78–1.73 (m, 1H), 1.71 (br s, 2H), 0.74 (ddt, J = 9.5, 7.4, 3.7 Hz, 1H), -0.03 (s, 9H).

^{13}C NMR (126 MHz, $CDCl_3$) δ 142.6, 128.8, 128.3, 126.9, 68.0, 64.1, 47.8, 29.4, 27.2, –2.4.

HRMS m/z (ESI): calculated for $C_{14}H_{24}O_2Si_1Na_1$ $[M+Na]^+$: 275.1438, found 275.1436.

$[\alpha]_D^{25}$ = +31.7 (c 0.31, CH_2Cl_2).

The enantiomeric excess was determined by chiral HPLC analysis on IA-3 column. Conditions: heptane/isopropanol = 90:10, flow rate = 1.0 mL/min, uv-vis detection at λ = 220 nm, t_{R1} = 7.3 min (major), t_{R2} = 9.9 min (minor). >99.5:0.5 er

(*S*)-5-methoxy-5-oxo-2-phenylpentanoic acid (12)

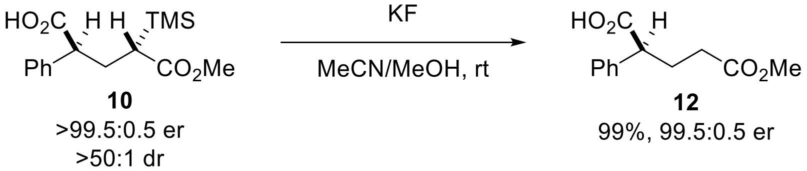

The **10** (29.5 mg, 0.1 mmol, 1.0 equiv.) was dissolved in MeCN/MeOH (v/v = 2/1, 1 mL). KF (6.4 mg, 0.11 mmol, 1.1 equiv.) was added and the reaction solution was stirred at rt for 10 min. The solution was quenched with 2 M aq. HCl (5 mL) at 0 °C and EtOAc (5 mL) was added. The two layers were separated, and the aqueous layer was extracted with EtOAc (5 mL*2). The combined organic layers were washed with brine and dried with Na_2SO_4, and the solvent was evaporated. The residue was purified by column chromatography on silica gel to afford 22.0. mg (99% yield) of **12** as a colourless oil.

^{1}H NMR (501 MHz, $CDCl_3$) δ 7.36–7.26 (m, 5H), 3.64 (s, 3H), 3.64 (t, J = 7.6 Hz, 1H), 2.43–2.34 (m, 1H), 2.29 (t, J = 7.7 Hz, 2H), 2.17–2.08 (m, 1H).

^{13}C NMR (126 MHz, $CDCl_3$) δ 178.7, 173.4, 137.7, 129.0, 128.2, 127.9, 51.8, 50.5, 31.7, 28.1.

HRMS m/z (ESI): calculated for $C_{12}H_{14}O_4Na_1$ $[M+Na]^+$: 245.0783, found 245.0784.

$[\alpha]_D^{25}$ = +36.4 (c 0.21, CH_2Cl_2).

The enantiomeric excess was determined by chiral HPLC analysis on AD-3 column. Conditions: heptane/isopropanol = 95:5, flow rate = 1.0 mL/min, uv-vis detection at λ = 220 nm, t_{R1} = 11.8 min (minor), t_{R2} = 13.2 min (major). 99.5:0.5 er

(*S*)-2-phenylpentane-1,5-diol (13)

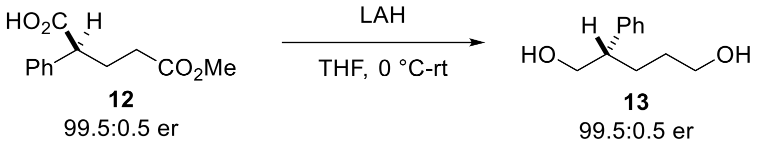

The **12** (22.2 mg, 0.1 mmol, 1.0 equiv.) was dissolved in THF (1 mL) under argon. The solution was cooled to 0 °C and LAH (0.2 mL, 1 M in THF, 0.2 mmol, 2.0 equiv.) was added dropwise. After addition, the reaction solution was stirred at rt for 2 h. The solution was quenched with sat. aq. NH_4Cl (1 mL) and 2 M aq. HCl (1 mL) at 0 °C and EtOAc (5 mL) was added. The two layers were separated, and the aqueous layer was extracted with EtOAc (5 mL*2). The combined organic layers were washed with brine and dried with Na_2SO_4, and the solvent was evaporated. The residue was purified by column chromatography on silica gel to afford 17.7 mg (98% yield) of **13** as a colourless oil, and it will be solidified in pentane.

^{1}H NMR (501 MHz, $CDCl_3$) δ 7.38–7.30 (m, 2H), 7.26–7.20 (m, 3H), 3.80–3.72 (m, 2H), 3.61 (t, J = 6.5 Hz, 2H), 2.85–2.76 (m, 1H), 1.88–1.77 (m, 1H), 1.69–1.61 (m, 1H), 1.54–1.44 (m, 2H), 1.33 (br s, 2H).

^{13}C NMR (126 MHz, $CDCl_3$) δ 142.2, 128.9, 128.2, 127.0, 67.7, 63.0, 48.6, 30.7, 28.3.

HRMS m/z (ESI): calculated for $C_{11}H_{16}O_2Na_1$ $[M+Na]^+$: 203.1043, found 203.1043.

The enantiomeric excess was determined by chiral HPLC analysis on OZ-3 column. Conditions: heptane/isopropanol = 98:2, flow rate = 1.0 mL/min, uv-vis detection at λ = 220 nm, t_{R1} = 7.9 min (minor), t_{R2} = 9.7 min (major). 99.5:0.5 er

8. Synthesis of Piperidine Fragment

(*S*)-2-(4-bromophenyl)pentane-1,5-diol (14)

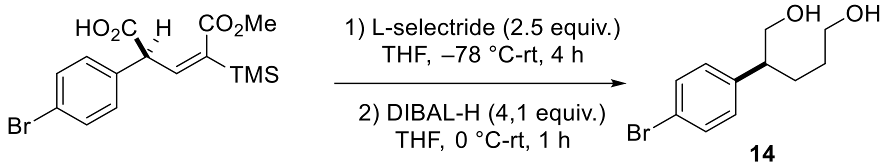

14
81%, 98:2 er
over 2 steps (1 column)

A stirring solution of **4e** (250 mg, 0.67 mmol, 1.0 equiv.) in THF (5 mL) under argon was cooled to –78 °C. L-selectride (1.68 mL, 1 M in THF, 1.68 mmol, 2.5 equiv.) was added dropwise at –78 °C. After addition, the reaction solution was stirred at –78 °C for 1 h before removing the cooling bath, and the reaction solution was stirred at rt for 3 h. The solution was quenched with 2 M aqueous HCl (10 mL) at 0 °C and Et_2O (5 mL) was added. The two layers were separated, and the aqueous layer was extracted with Et_2O (5 mL*2). The combined organic layers were washed with brine and dried with Na_2SO_4, and the solvent was evaporated and the residue was used without purification.

The residue was dissolved in THF (5 mL) under argon. The solution was cooled to 0 °C and DIBAL-H (2.76 mL, 1 M in toulene, 2.76 mmol, 4.1 equiv.) was added dropwise. After addition, the reaction solution was stirred at rt for 2 h. The solution was quenched with 2 M aq. HCl (10 mL) at 0 °C and EtOAc (5 mL) was added. The two layers were separated, and the aqueous layer was extracted with EtOAc (5 mL*2). The combined organic layers were washed with brine and dried with Na_2SO_4, and the solvent was evaporated. The residue was purified by column chromatography on silica gel (eluent: EtOAc) to afford 141 mg (81% yield) of **14** as a white solid.

^{1}H NMR (501 MHz, $CDCl_3$) δ 7.45 (d, J = 8.4 Hz, 2H), 7.10 (d, J = 8.4 Hz, 2H), 3.74 (qd, J = 10.8, 6.7 Hz, 2H), 3.60 (t, J = 6.4 Hz, 2H), 2.77 (ddt, J = 9.6, 7.4, 5.5 Hz, 1H), 1.87–1.78 (m, 1H), 1.65–1.56 (m, 1H), 1.53–1.38 (m, 4H).

^{13}C NMR (126 MHz, $CDCl_3$) δ 141.4, 131.9, 129.9, 120.7, 67.4, 62.8, 48.0, 30.5, 28.2.

HRMS m/z (ESI): calculated for $C_{11}H_{15}O_2Br_1Na_1$ [M+Na]$^+$: 281.1048, found 281.1047.

$[\alpha]_D^{25}$ = +10.8 (c 0.75, CH_2Cl_2).

The enantiomeric excess was determined by chiral HPLC analysis on IG-3R column. Conditions: acetonitrile/water = 40:60, flow rate = 1.0 mL/min, uv-vis detection at λ = 220 nm, t_{R1} = 5.7 min (major), t_{R2} = 6.6 min (minor). 98:2 er

(*S*)-3-(4-bromophenyl)piperidine (15)

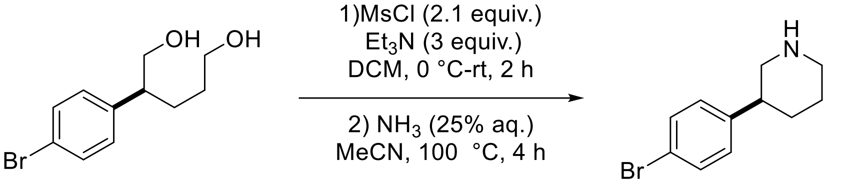

15
86%, 98:2 er
over 2 steps (1 column)

A stirring solution of **14** (52 mg, 0.2 mmol, 1.0 equiv.) and Et_3N (120 µL, 0.8 mmol, 4.0 equiv.) in DCM (1 mL) under argon was cooled to 0 °C. MsCl (39 µL, 0.50 mmol, 2.5 equiv.) was added dropwise at 0 °C. Then the reaction solution was stirred at rt for 2 h. The solution was quenched with H_2O (1 mL). The two layers were separated, and the aqueous layer was extracted with Et_2O (5 mL*2). The combined organic layers were washed with brine and dried with Na_2SO_4, and the solvent was evaporated and the residue was used without purification.

The residue was dissolved in MeCN (1 mL) in a Schlenk tube and ammonia (1 mL, 25% aq.) was added. The Schlenk tube was sealed and the reaction solution was stirred at 100 °C for 4 h. The solution was then cooled to rt, and H_2O (5 mL) and DCM (5 mL) was added. The two layers were separated, and the aqueous layer was extracted with DCM (5 mL*2). The combined organic layers were washed with brine and dried with Na_2SO_4, and the solvent was evaporated.

The residue was purified by column chromatography on silica gel (eluent: MeOH/DCM/Et_3N = 20/80/1) to afford 41.4 mg (86% yield) of **15** as a white solid.

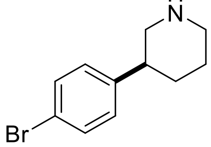

^{1}H NMR (501 MHz, $CDCl_3$) δ 7.40 (d, J = 8.4 Hz, 2H), 7.08 (d, J = 8.4 Hz, 2H), 3.18–3.04 (m, 2H), 2.68–2.56 (m, 3H), 2.00–1.92 (m, 1H), 1.83–1.74 (m, 1H), 1.70 (br s, 1H), 1.64–1.50 (m, 2H).

^{13}C NMR (126 MHz, $CDCl_3$) δ 144.0, 131.6, 129.0, 120.0, 54.1, 46.7, 43.9, 32.2, 27.1.

$[\alpha]_D^{25}$ = +6.4 (c 0.56, CH_2Cl_2).

Alternatively, the residue was purified by forming *p*-toluenesulfonate. The residue after removing the solvent was dissolved in diethyl ether to form a clear solution. To the residue solution an excess clear solution of *p*-Toluenesulfonic acid in diethyl ether was added dropwise and white precipitate was generated immediately. After standing for 15 minutes, the white solid was filtered and washed with diethyl ether to obtain 67.4 mg (82% yield) *p*-Toluenesulfonate of **15**. Characterization data is consistent with the literature.[7]

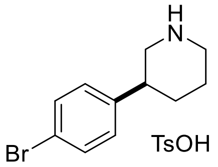

^{1}H NMR (501 MHz, DMSO-d$_6$) δ 8.75 (br s, 1H), 8.40 (br s, 1H), 7.55 (d, *J* = 8.3 Hz, 2H), 7.48 (d, *J* = 7.7 Hz, 2H), 7.26 (d, *J* = 8.3 Hz, 2H), 7.11 (d, *J* = 7.8 Hz, 2H), 3.30 (d, *J* = 11.9 Hz, 2H), 3.02 (q, *J* = 10.6 Hz, 1H), 2.96–2.80 (m, 2H), 2.29 (s, 3H), 1.93–1.82 (m, 2H), 1.79–1.62 (m, 2H).

^{13}C NMR (126 MHz, DMSO-d$_6$) δ 145.6, 141.1, 137.7, 131.5, 129.4, 128.1, 125.5, 120.1, 47.6, 43.0, 38.7, 29.2, 22.2, 20.8.

^{1}H NMR (501 MHz, CDCl$_3$) δ 9.02 (br s, 1H), 8.76 (br s, 1H), 7.75 (d, *J* = 8.1 Hz, 2H), 7.39 (d, *J* = 8.4 Hz, 2H), 7.19 (d, *J* = 8.0 Hz, 2H), 6.95 (d, *J* = 8.4 Hz, 2H), 3.57–3.46 (m, 2H), 3.12–3.01 (m, 1H), 2.91–2.75 (m, 2H), 2.37 (s, 3H), 2.02–1.86 (m, 3H), 1.62–1.51 (m, 1H).

^{13}C NMR (126 MHz, CDCl$_3$) δ 141.3, 141.1, 139.9, 132.0, 129.3, 128.9, 126.0, 121.3, 49.6, 44.3, 39.1, 29.8, 22.7, 21.5.

HRMS m/z (EI): calculated for $C_{11}H_{14}Br_1N_1$ $[M]^+$: 239.0304, found 239.0306.

The enantiomeric excess was determined by converting product **15** to its corresponding benzamide.

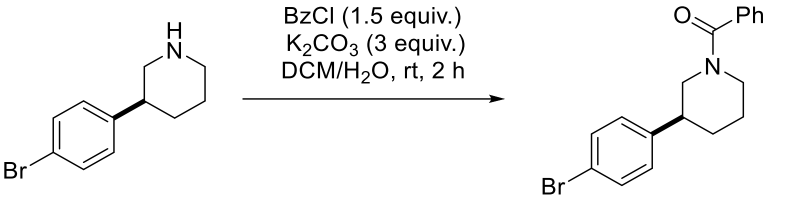

^{1}H NMR (501 MHz, CDCl$_3$) δ 7.52–6.81 (m, 9H), 4.81 (br s, 1H), 3.80 (br s, 1H), 3.12–2.88 (m, 1H), 2.88–2.59 (m, 2H), 2.16–2.05 (m, 1H), 2.00–1.53 (m, 3H).

HRMS m/z (EI): calculated for $C_{18}H_{18}O_1Br_1N_1$ $[M]^+$: 343.0566, found 343.0568.

Chiral HPLC conditions: IA-3 column, heptane/isopropanol = 90:10, flow rate = 1.0 mL/min, uv-vis detection at λ = 220 nm, t_{R1} = 17.8 min (minor), t_{R2} = 25.1 min (major). 98:2 er

(*R*)-15 was purchased from merck and converted to its corresponding benzamide to confirm the absolute configuration.

9. Crystallographic Data

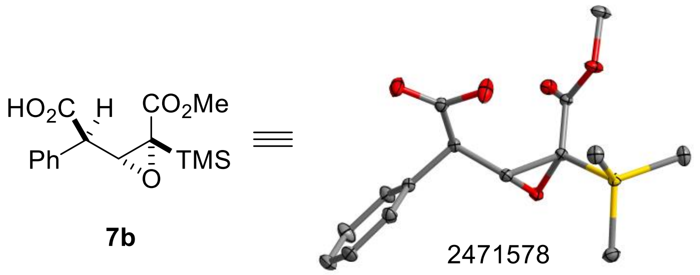

7b

Table S7. Crystal data and structure refinement of 7b

Identification code	14240	
Empirical formula	$C_{15} H_{20} O_5 Si$	
Color	colourless	
Formula weight	308.40 g·mol^{-1}	
Temperature	100(2) K	
Wavelength	0.71073 Å	
Crystal system	ORTHORHOMBIC	
Space group	**$P2_12_12_1$, (no. 19)**	
Unit cell dimensions	a = 5.9772(6) Å	α= 90°.
	b = 16.1168(8) Å	β= 90°.
	c = 16.3493(12) Å	γ = 90°.
Volume	1575.0(2) Å^3	
Z	4	
Density (calculated)	1.301 Mg · m^{-3}	
Absorption coefficient	0.167 mm^{-1}	
F(000)	656 e	
Crystal size	0.26 x 0.14 x 0.065 mm^3	
θ range for data collection	2.794 to 36.013°.	
Index ranges	-9 ≤ h ≤ 9, -26≤ k ≤ 26, -27 ≤ l ≤ 27	
Reflections collected	57565	
Independent reflections	7453 [R_{int} = 0.0441]	
Reflections with I>2σ(I)	6708	
Completeness to θ = 25.242°	99.8 %	
Absorption correction	Gaussian	
Max. and min. transmission	0.99 and 0.97	
Refinement method	Full-matrix least-squares on F^2	
Data / restraints / parameters	7453 / 0 / 206	
Goodness-of-fit on F^2	1.058	
Final R indices [I>2σ(I)]	R_1 = 0.0291	wR^2 = 0.0705
R indices (all data)	R_1 = 0.0362	wR^2 = 0.0732
Absolute structure parameter	0.00(2)	
Largest diff. peak and hole	0.3 and -0.2 e · Å^{-3}	

Table S8. Bond lengths [Å] and angles [°] of 7b

Si(1)-C(3)	1.9105(10)	Si(1)-C(7)	1.8613(12)
Si(1)-C(8)	1.8617(11)	Si(1)-C(9)	1.8607(12)
O(1)-C(1)	1.4540(13)	O(1)-C(2)	1.3274(12)
O(2)-C(2)	1.2168(12)	O(3)-C(3)	1.4610(12)
O(3)-C(4)	1.4251(13)	O(4)-C(6)	1.2038(13)
O(5)-H(5)	0.83(3)	O(5)-C(6)	1.3225(13)
C(2)-C(3)	1.4965(14)	C(3)-C(4)	1.4853(13)
C(4)-H(4)	0.976(18)	C(4)-C(5)	1.5180(14)
C(5)-H(5A)	0.963(16)	C(5)-C(6)	1.5307(14)
C(5)-C(10)	1.5162(14)	C(10)-C(11)	1.3896(16)
C(10)-C(15)	1.3973(15)	C(11)-C(12)	1.3998(16)
C(12)-C(13)	1.384(2)	C(13)-C(14)	1.3878(19)
C(14)-C(15)	1.3923(16)		
C(7)-Si(1)-C(3)	112.03(5)	C(7)-Si(1)-C(8)	109.68(6)
C(8)-Si(1)-C(3)	107.30(5)	C(9)-Si(1)-C(3)	105.87(5)
C(9)-Si(1)-C(7)	110.52(6)	C(9)-Si(1)-C(8)	111.36(6)
C(2)-O(1)-C(1)	115.67(9)	C(4)-O(3)-C(3)	61.93(6)
C(6)-O(5)-H(5)	107.8(16)	O(1)-C(2)-C(3)	110.57(8)
O(2)-C(2)-O(1)	123.22(9)	O(2)-C(2)-C(3)	126.19(9)
O(3)-C(3)-Si(1)	117.39(7)	O(3)-C(3)-C(2)	112.55(8)
O(3)-C(3)-C(4)	57.85(6)	C(2)-C(3)-Si(1)	121.32(7)
C(4)-C(3)-Si(1)	114.64(7)	C(4)-C(3)-C(2)	117.02(9)
O(3)-C(4)-C(3)	60.22(6)	O(3)-C(4)-H(4)	114.0(10)
O(3)-C(4)-C(5)	117.18(8)	C(3)-C(4)-H(4)	116.6(10)
C(3)-C(4)-C(5)	125.59(9)	C(5)-C(4)-H(4)	112.6(10)
C(4)-C(5)-H(5A)	109.5(9)	C(4)-C(5)-C(6)	111.09(8)
C(6)-C(5)-H(5A)	106.4(9)	C(10)-C(5)-C(4)	109.46(8)
C(10)-C(5)-H(5A)	108.5(9)	C(10)-C(5)-C(6)	111.84(8)
O(4)-C(6)-O(5)	124.36(11)	O(4)-C(6)-C(5)	124.41(10)
O(5)-C(6)-C(5)	111.21(9)	C(11)-C(10)-C(5)	120.74(9)
C(11)-C(10)-C(15)	119.42(10)	C(15)-C(10)-C(5)	119.77(9)
C(10)-C(11)-C(12)	120.08(11)	C(13)-C(12)-C(11)	120.14(12)
C(12)-C(13)-C(14)	120.03(11)	C(13)-C(14)-C(15)	120.04(11)
C(14)-C(15)-C(10)	120.28(11)		

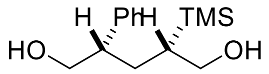

11

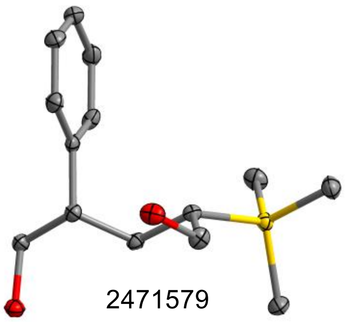

2471579

Table S9. Crystal data and structure refinement of 11

Identification code	14393	
Empirical formula	$C_{14} H_{24} O_2 Si$	
Color	colourless	
Formula weight	252.42 g·mol^{-1}	
Temperature	100(2) K	
Wavelength	0.71073 Å	
Crystal system	Orthorhombic	
Space group	$P2_12_12_1$, (no. 19)	
Unit cell dimensions	a = 6.4942(2) Å	α= 90°.
	b = 17.4366(6) Å	β= 90°.
	c = 26.4891(9) Å	γ = 90°.
Volume	2999.54(17) Å^3	
Z	8	
Density (calculated)	1.118 Mg·m^{-3}	
Absorption coefficient	0.147 mm^{-1}	
F(000)	1104 e	
Crystal size	0.07 x 0.052 x 0.042 mm^3	
θ range for data collection	1.398 to 30.998°.	
Index ranges	$-9 \le h \le 9$, $-25 \le k \le 25$, $-37 \le l \le 38$	
Reflections collected	71120	
Independent reflections	9570 [R_{int} = 0.0658]	
Reflections with I>2σ(I)	7639	
Completeness to θ = 25.242°	100.0 %	
Absorption correction	Gaussian	
Max. and min. transmission	0.99454 and 0.99175	
Refinement method	Full-matrix least-squares on F^2	
Data / restraints / parameters	9570 / 0 / 320	
Goodness-of-fit on F^2	1.019	
Final R indices [I>2σ(I)]	R_1 = 0.0408	wR^2 = 0.0772
R indices (all data)	R_1 = 0.0621	wR^2 = 0.0840
Absolute structure parameter	-0.04(3)	
Extinction coefficient	n/a	
Largest diff. peak and hole	0.298 and -0.288 e·Å^{-3}	

Table S10. Bond lengths [Å] and angles [°] of 11

Si(1)-C(1)	1.8881(19)	Si(1)-C(12)	1.869(2)
Si(1)-C(13)	1.869(2)	Si(1)-C(14)	1.871(2)
Si(2)-C(15)	1.896(2)	Si(2)-C(26)	1.870(2)
Si(2)-C(27)	1.870(2)	Si(2)-C(28)	1.865(2)
O(1)-H(1)	0.8400	O(1)-C(2)	1.434(2)
O(2)-H(2)	0.8400	O(2)-C(5)	1.442(2)
O(3)-C(16)	1.441(3)	O(3)-H(3)	0.78(3)
O(4)-H(4A)	0.8400	O(4)-C(19)	1.439(2)
C(1)-H(1A)	1.0000	C(1)-C(2)	1.525(3)
C(1)-C(3)	1.543(3)	C(2)-H(2A)	0.9900
C(2)-H(2B)	0.9900	C(3)-H(3A)	0.9900
C(3)-H(3B)	0.9900	C(3)-C(4)	1.536(3)
C(4)-H(4)	1.0000	C(4)-C(5)	1.525(3)
C(4)-C(6)	1.515(3)	C(5)-H(5A)	0.9900
C(5)-H(5B)	0.9900	C(6)-C(7)	1.393(3)
C(6)-C(11)	1.398(3)	C(7)-H(7)	0.9500
C(7)-C(8)	1.388(3)	C(8)-H(8)	0.9500
C(8)-C(9)	1.392(3)	C(9)-H(9)	0.9500
C(9)-C(10)	1.380(3)	C(10)-H(10)	0.9500
C(10)-C(11)	1.393(3)	C(11)-H(11)	0.9500
C(12)-H(12A)	0.9800	C(12)-H(12B)	0.9800
C(12)-H(12C)	0.9800	C(13)-H(13A)	0.9800
C(13)-H(13B)	0.9800	C(13)-H(13C)	0.9800
C(14)-H(14A)	0.9800	C(14)-H(14B)	0.9800
C(14)-H(14C)	0.9800	C(15)-H(15)	1.0000
C(15)-C(16)	1.528(3)	C(15)-C(17)	1.546(3)
C(16)-H(16A)	0.9900	C(16)-H(16B)	0.9900
C(17)-H(17A)	0.9900	C(17)-H(17B)	0.9900
C(17)-C(18)	1.539(3)	C(18)-H(18)	1.0000
C(18)-C(19)	1.531(3)	C(18)-C(20)	1.520(3)
C(19)-H(19A)	0.9900	C(19)-H(19B)	0.9900
C(20)-C(21)	1.393(3)	C(20)-C(25)	1.398(3)
C(21)-H(21)	0.9500	C(21)-C(22)	1.390(3)
C(22)-H(22)	0.9500	C(22)-C(23)	1.385(3)
C(23)-H(23)	0.9500	C(23)-C(24)	1.382(3)
C(24)-H(24)	0.9500	C(24)-C(25)	1.389(3)
C(25)-H(25)	0.9500	C(26)-H(26A)	0.9800
C(26)-H(26B)	0.9800	C(26)-H(26C)	0.9800
C(27)-H(27A)	0.9800	C(27)-H(27B)	0.9800
C(27)-H(27C)	0.9800	C(28)-H(28A)	0.9800
C(28)-H(28B)	0.9800	C(28)-H(28C)	0.9800
C(12)-Si(1)-C(1)	109.14(10)	C(12)-Si(1)-C(13)	108.97(11)

C(12)-Si(1)-C(14)	108.91(10)	C(13)-Si(1)-C(1)	109.12(9)
C(13)-Si(1)-C(14)	111.17(10)	C(14)-Si(1)-C(1)	109.49(9)
C(26)-Si(2)-C(15)	107.68(10)	C(26)-Si(2)-C(27)	110.11(11)
C(27)-Si(2)-C(15)	111.09(11)	C(28)-Si(2)-C(15)	109.05(10)
C(28)-Si(2)-C(26)	109.04(11)	C(28)-Si(2)-C(27)	109.81(11)
C(2)-O(1)-H(1)	109.5	C(5)-O(2)-H(2)	109.5
C(16)-O(3)-H(3)	110(2)	C(19)-O(4)-H(4A)	109.5
Si(1)-C(1)-H(1A)	106.8	C(2)-C(1)-Si(1)	112.51(13)
C(2)-C(1)-H(1A)	106.8	C(2)-C(1)-C(3)	112.19(16)
C(3)-C(1)-Si(1)	111.40(12)	C(3)-C(1)-H(1A)	106.8
O(1)-C(2)-C(1)	111.37(16)	O(1)-C(2)-H(2A)	109.4
O(1)-C(2)-H(2B)	109.4	C(1)-C(2)-H(2A)	109.4
C(1)-C(2)-H(2B)	109.4	H(2A)-C(2)-H(2B)	108.0
C(1)-C(3)-H(3A)	108.3	C(1)-C(3)-H(3B)	108.3
H(3A)-C(3)-H(3B)	107.4	C(4)-C(3)-C(1)	115.86(15)
C(4)-C(3)-H(3A)	108.3	C(4)-C(3)-H(3B)	108.3
C(3)-C(4)-H(4)	108.2	C(5)-C(4)-C(3)	111.51(15)
C(5)-C(4)-H(4)	108.2	C(6)-C(4)-C(3)	111.30(15)
C(6)-C(4)-H(4)	108.2	C(6)-C(4)-C(5)	109.44(15)
O(2)-C(5)-C(4)	112.84(15)	O(2)-C(5)-H(5A)	109.0
O(2)-C(5)-H(5B)	109.0	C(4)-C(5)-H(5A)	109.0
C(4)-C(5)-H(5B)	109.0	H(5A)-C(5)-H(5B)	107.8
C(7)-C(6)-C(4)	121.06(18)	C(7)-C(6)-C(11)	118.14(18)
C(11)-C(6)-C(4)	120.79(18)	C(6)-C(7)-H(7)	119.5
C(8)-C(7)-C(6)	121.08(19)	C(8)-C(7)-H(7)	119.5
C(7)-C(8)-H(8)	119.9	C(7)-C(8)-C(9)	120.1(2)
C(9)-C(8)-H(8)	119.9	C(8)-C(9)-H(9)	120.2
C(10)-C(9)-C(8)	119.56(19)	C(10)-C(9)-H(9)	120.2
C(9)-C(10)-H(10)	119.9	C(9)-C(10)-C(11)	120.3(2)
C(11)-C(10)-H(10)	119.9	C(6)-C(11)-H(11)	119.6
C(10)-C(11)-C(6)	120.84(19)	C(10)-C(11)-H(11)	119.6
Si(1)-C(12)-H(12A)	109.5	Si(1)-C(12)-H(12B)	109.5
Si(1)-C(12)-H(12C)	109.5	H(12A)-C(12)-H(12B)	109.5
H(12A)-C(12)-H(12C)	109.5	H(12B)-C(12)-H(12C)	109.5
Si(1)-C(13)-H(13A)	109.5	Si(1)-C(13)-H(13B)	109.5
Si(1)-C(13)-H(13C)	109.5	H(13A)-C(13)-H(13B)	109.5
H(13A)-C(13)-H(13C)	109.5	H(13B)-C(13)-H(13C)	109.5
Si(1)-C(14)-H(14A)	109.5	Si(1)-C(14)-H(14B)	109.5
Si(1)-C(14)-H(14C)	109.5	H(14A)-C(14)-H(14B)	109.5
H(14A)-C(14)-H(14C)	109.5	H(14B)-C(14)-H(14C)	109.5
Si(2)-C(15)-H(15)	106.5	C(16)-C(15)-Si(2)	111.16(14)
C(16)-C(15)-H(15)	106.5	C(16)-C(15)-C(17)	112.46(16)
C(17)-C(15)-Si(2)	113.16(13)	C(17)-C(15)-H(15)	106.5
O(3)-C(16)-C(15)	113.59(18)	O(3)-C(16)-H(16A)	108.8

O(3)-C(16)-H(16B)	108.8	C(15)-C(16)-H(16A)	108.8
C(15)-C(16)-H(16B)	108.8	H(16A)-C(16)-H(16B)	107.7
C(15)-C(17)-H(17A)	108.6	C(15)-C(17)-H(17B)	108.6
H(17A)-C(17)-H(17B)	107.6	C(18)-C(17)-C(15)	114.71(16)
C(18)-C(17)-H(17A)	108.6	C(18)-C(17)-H(17B)	108.6
C(17)-C(18)-H(18)	107.9	C(19)-C(18)-C(17)	111.21(17)
C(19)-C(18)-H(18)	107.9	C(20)-C(18)-C(17)	112.87(15)
C(20)-C(18)-H(18)	107.9	C(20)-C(18)-C(19)	108.94(16)
O(4)-C(19)-C(18)	110.68(16)	O(4)-C(19)-H(19A)	109.5
O(4)-C(19)-H(19B)	109.5	C(18)-C(19)-H(19A)	109.5
C(18)-C(19)-H(19B)	109.5	H(19A)-C(19)-H(19B)	108.1
C(21)-C(20)-C(18)	121.32(18)	C(21)-C(20)-C(25)	117.84(19)
C(25)-C(20)-C(18)	120.83(18)	C(20)-C(21)-H(21)	119.3
C(22)-C(21)-C(20)	121.4(2)	C(22)-C(21)-H(21)	119.3
C(21)-C(22)-H(22)	120.1	C(23)-C(22)-C(21)	119.8(2)
C(23)-C(22)-H(22)	120.1	C(22)-C(23)-H(23)	120.1
C(24)-C(23)-C(22)	119.7(2)	C(24)-C(23)-H(23)	120.1
C(23)-C(24)-H(24)	119.8	C(23)-C(24)-C(25)	120.3(2)
C(25)-C(24)-H(24)	119.8	C(20)-C(25)-H(25)	119.6
C(24)-C(25)-C(20)	120.9(2)	C(24)-C(25)-H(25)	119.6
Si(2)-C(26)-H(26A)	109.5	Si(2)-C(26)-H(26B)	109.5
Si(2)-C(26)-H(26C)	109.5	H(26A)-C(26)-H(26B)	109.5
H(26A)-C(26)-H(26C)	109.5	H(26B)-C(26)-H(26C)	109.5
Si(2)-C(27)-H(27A)	109.5	Si(2)-C(27)-H(27B)	109.5
Si(2)-C(27)-H(27C)	109.5	H(27A)-C(27)-H(27B)	109.5
H(27A)-C(27)-H(27C)	109.5	H(27B)-C(27)-H(27C)	109.5
Si(2)-C(28)-H(28A)	109.5	Si(2)-C(28)-H(28B)	109.5
Si(2)-C(28)-H(28C)	109.5	H(28A)-C(28)-H(28B)	109.5
H(28A)-C(28)-H(28C)	109.5	H(28B)-C(28)-H(28C)	109.5

10. HPLC Traces of the Products

HPLC trace of *rac*-**4a** and (*S*)-**4a**

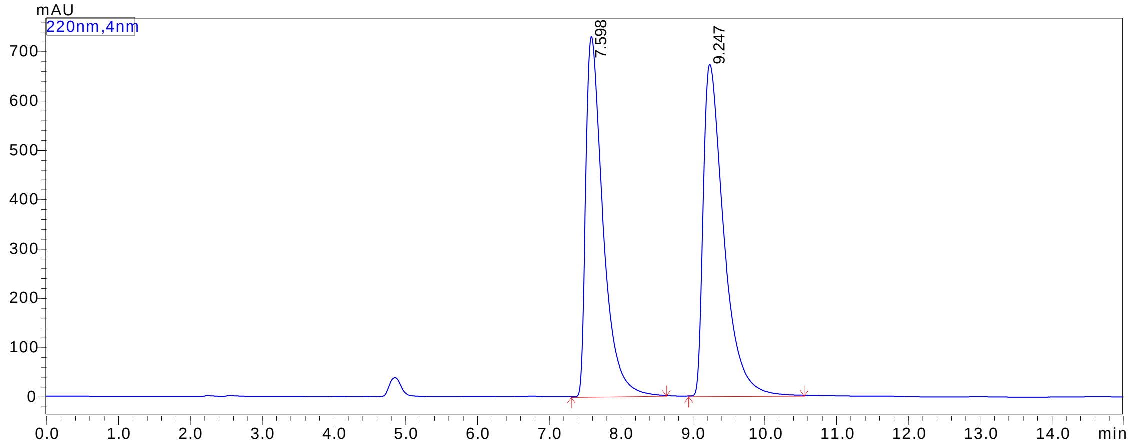

Peak#	Ret. Time	Area%
1	7.598	49.051
2	9.247	50.049
Total		100.000

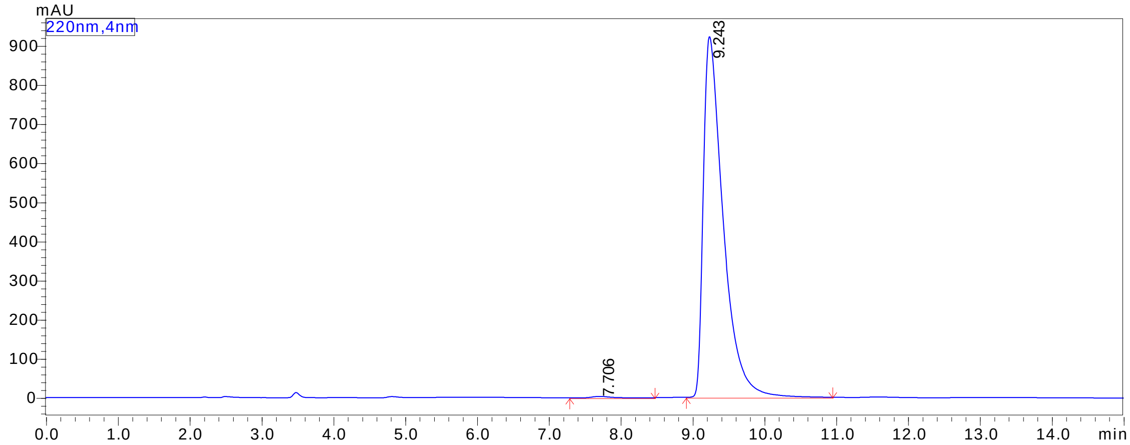

Peak#	Ret. Time	Area%
1	7.706	0.267
2	9.243	99.733
Total		100.000

HPLC trace of *rac*-**4b** and (*S*)-**4b**

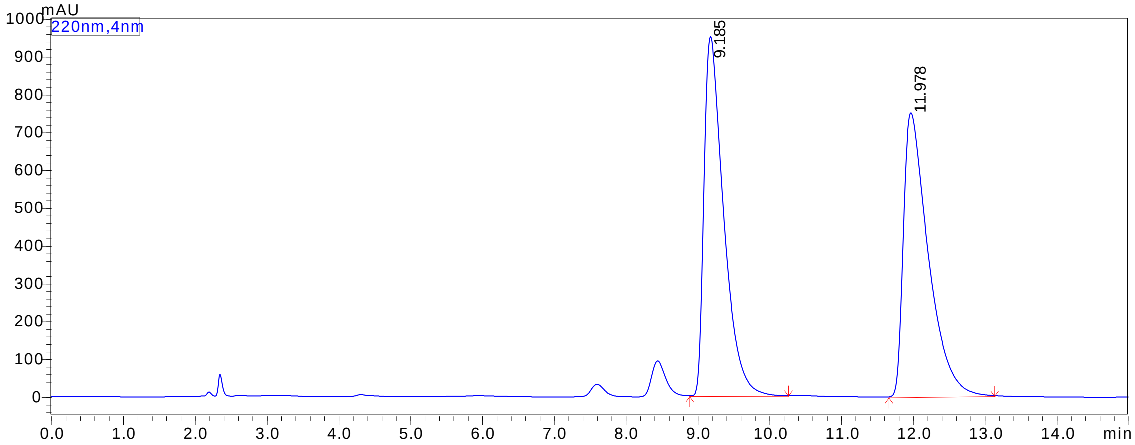

Peak#	Ret. Time	Area%
1	9.185	49.993
2	11.978	50.007
Total		100.000

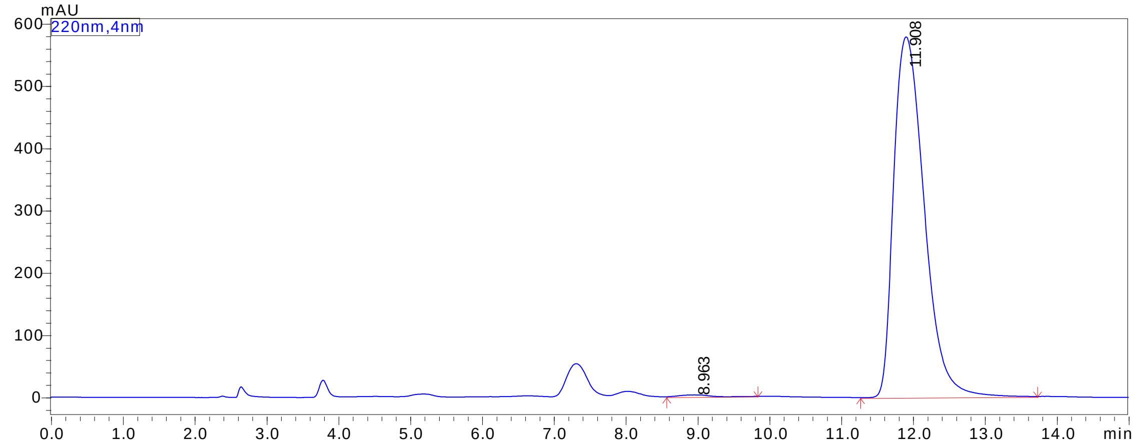

Peak#	Ret. Time	Area%
1	8.963	0.379
2	11.908	99.621
Total		100.000

HPLC trace of *rac*-**4c** and (*S*)-**4c**

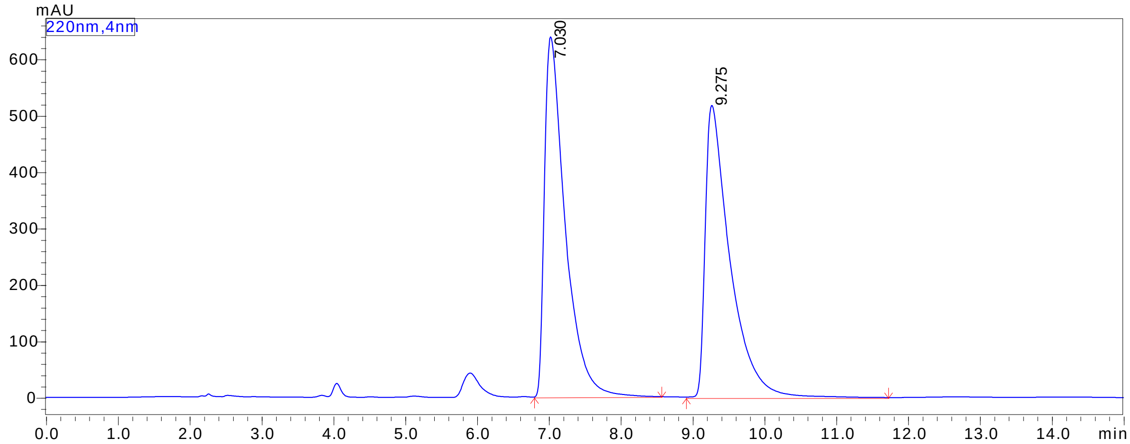

Peak#	Ret. Time	Area%
1	7.030	50.862
2	9.275	49.138
Total		100.000

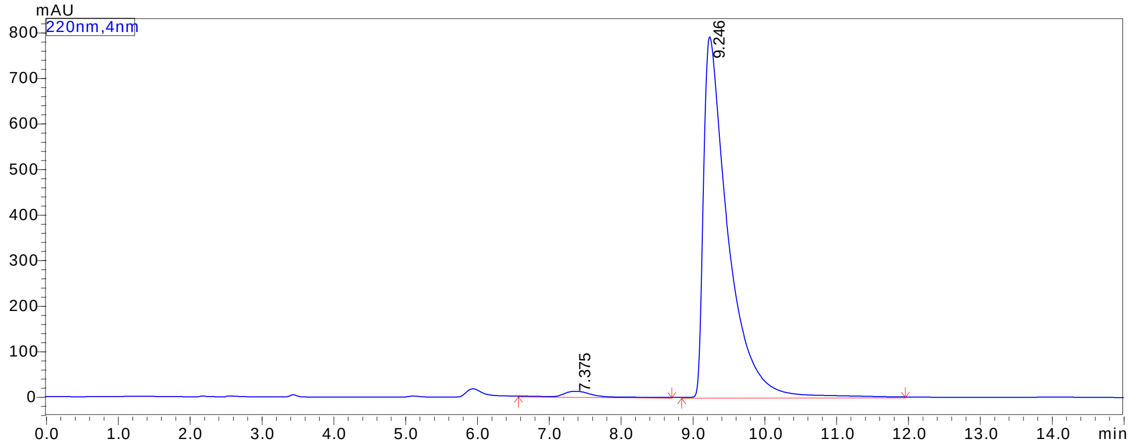

Peak#	Ret. Time	Area%
1	7.375	1.235
2	9.246	98.765
Total		100.000

HPLC trace of *rac*-**4d** and (*S*)-**4d**

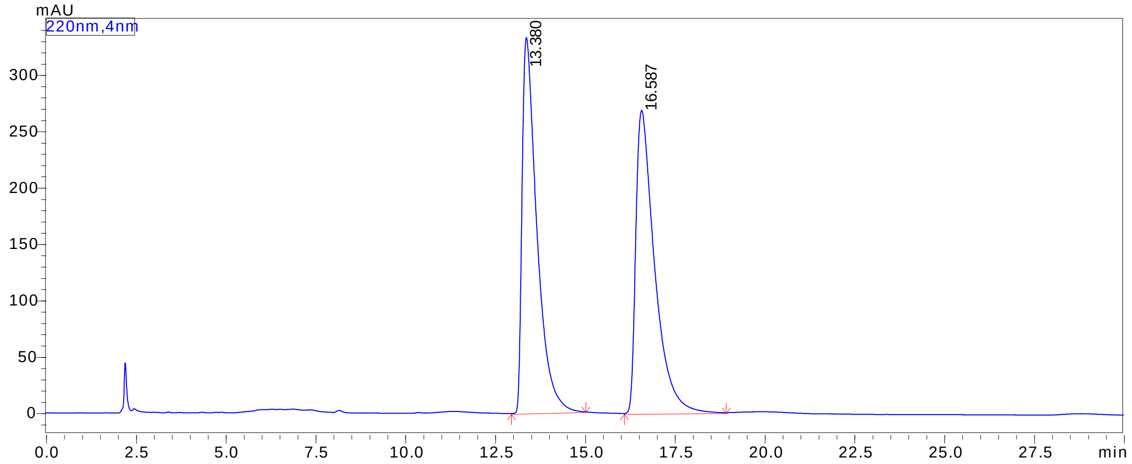

Peak#	Ret. Time	Area%
1	13.380	49.496
2	16.587	50.504
Total		100.000

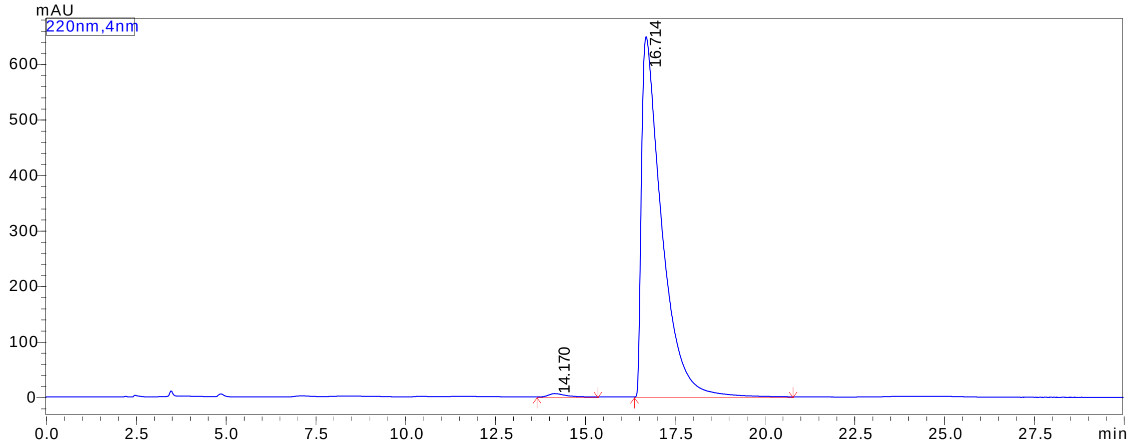

Peak#	Ret. Time	Area%
1	14.170	0.740
2	16.714	99.260
Total		100.000

HPLC trace of *rac*-**4e** and (*S*)-**4e**

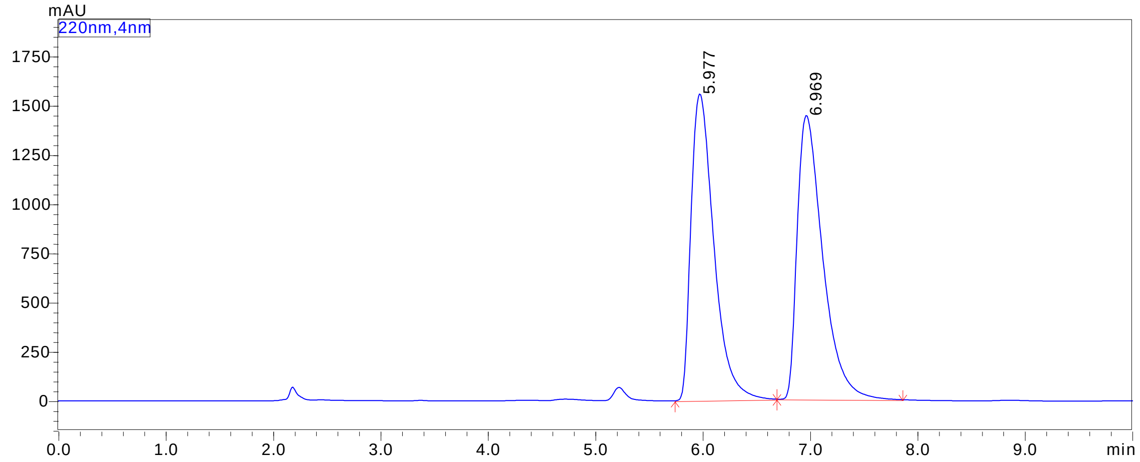

Peak#	Ret. Time	Area%
1	5.977	49.178
2	6.969	50.822
Total		100.000

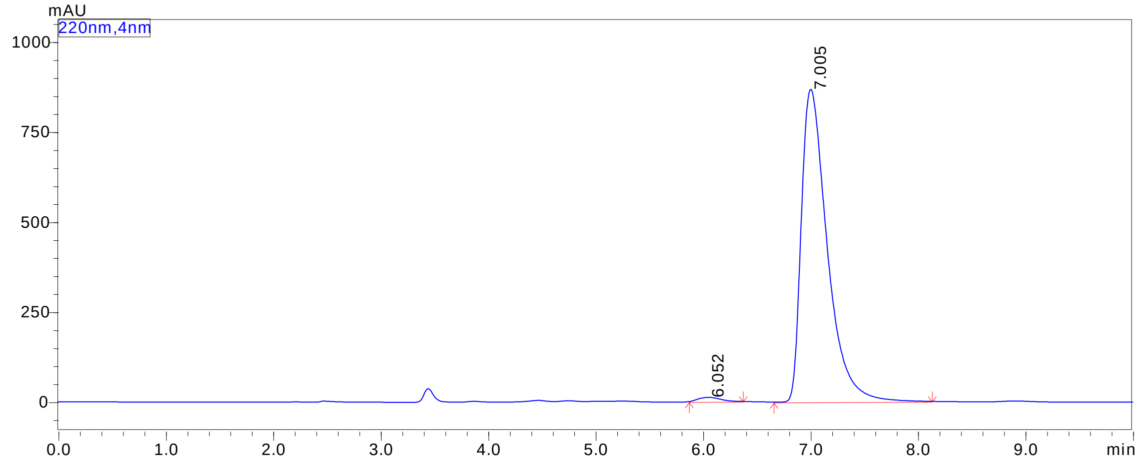

Peak#	Ret. Time	Area%
1	6.052	1.114
2	7.005	98.886
Total		100.000

HPLC trace of *rac*-**4f** and (*S*)-**4f**

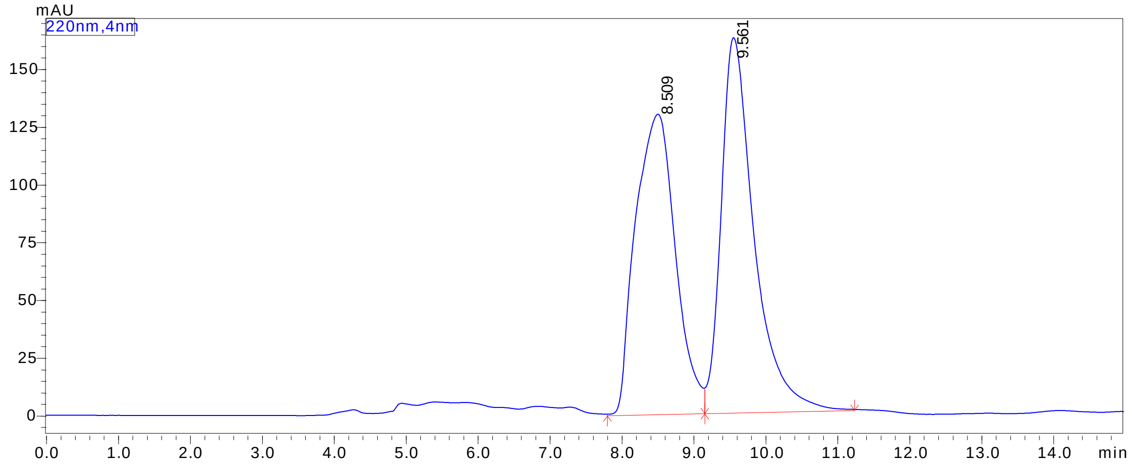

Peak#	Ret. Time	Area%
1	8.509	49.252
2	9.561	50.748
Total		100.000

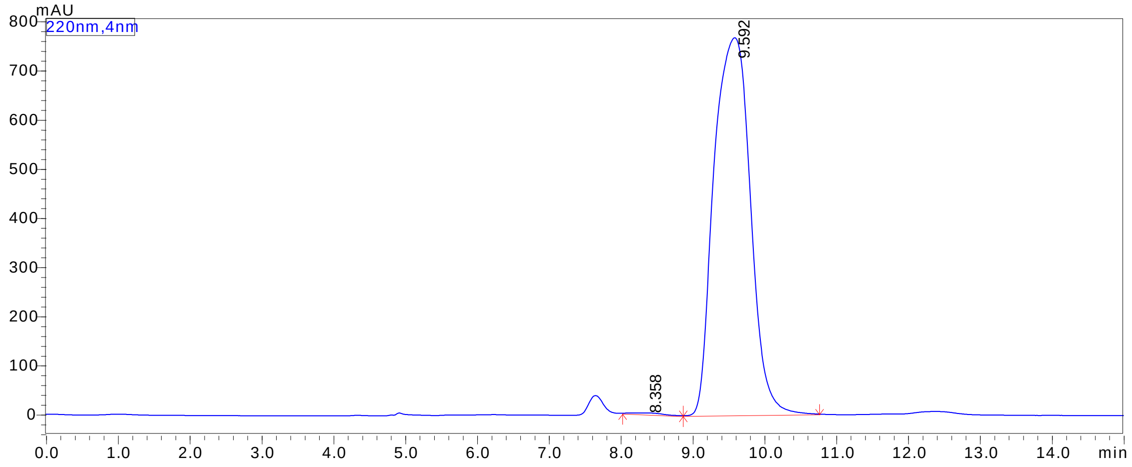

Peak#	Ret. Time	Area%
1	8.358	0.205
2	9.592	99.795
Total		100.000

HPLC trace of *rac*-**4g** and (*S*)-**4g**

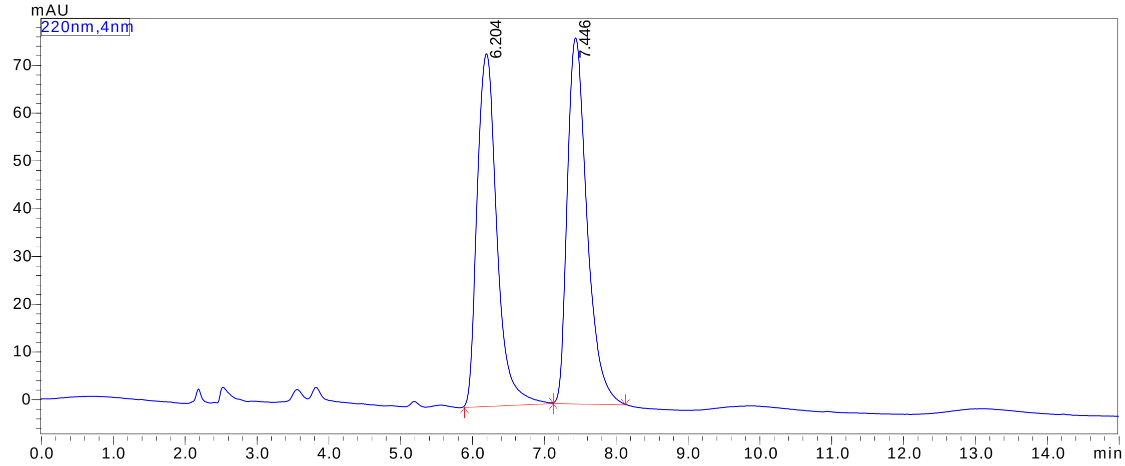

Peak#	Ret. Time	Area%
1	6.204	49.075
2	7.446	50.925
Total		100.000

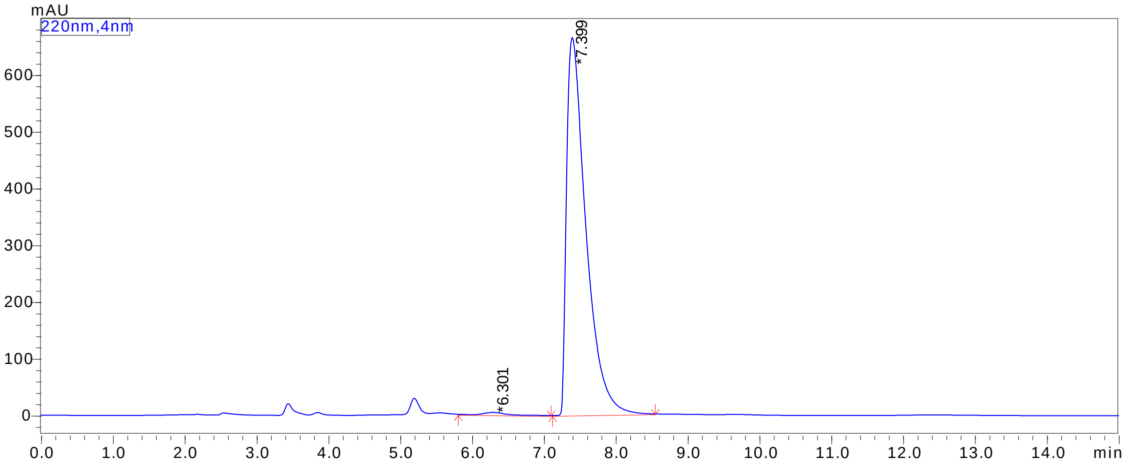

Peak#	Ret. Time	Area%
1	6.301	0.485
2	7.399	99.515
Total		100.000

HPLC trace of *rac*-**4h** and (*S*)-**4h**

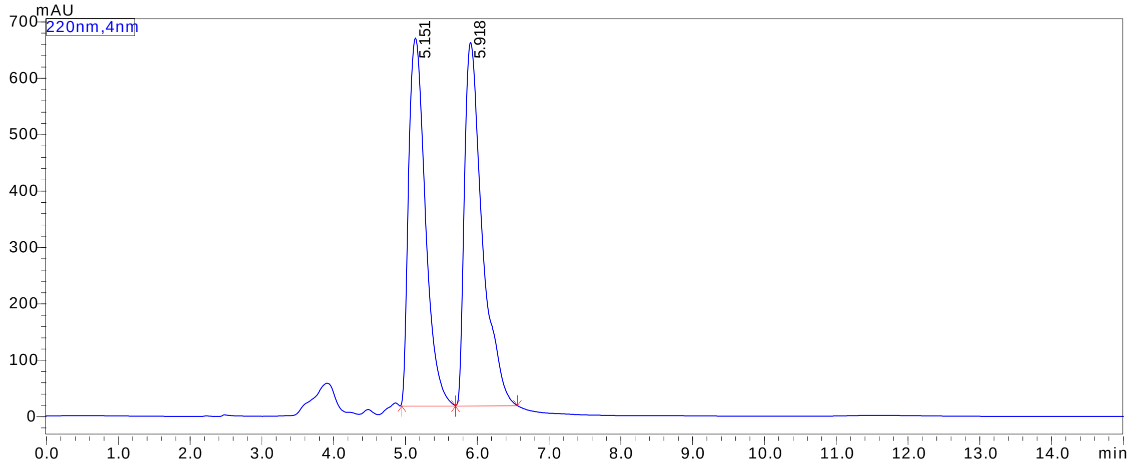

Peak#	Ret. Time	Area%
1	5.151	48.711
2	5.918	51.289
Total		100.000

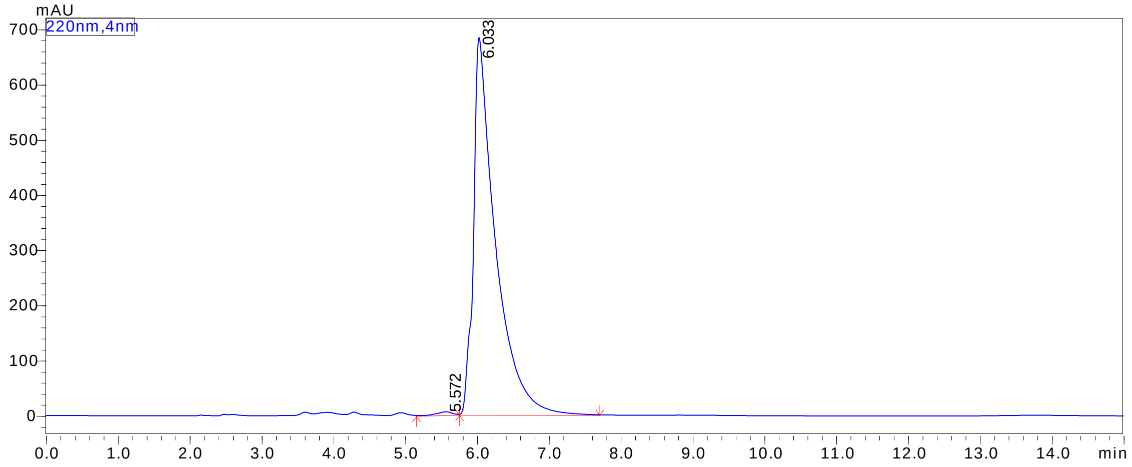

Peak#	Ret. Time	Area%
1	5.572	0.422
2	6.033	99.578
Total		100.000

HPLC trace of *rac*-**4i** and (*R*)-**4i**

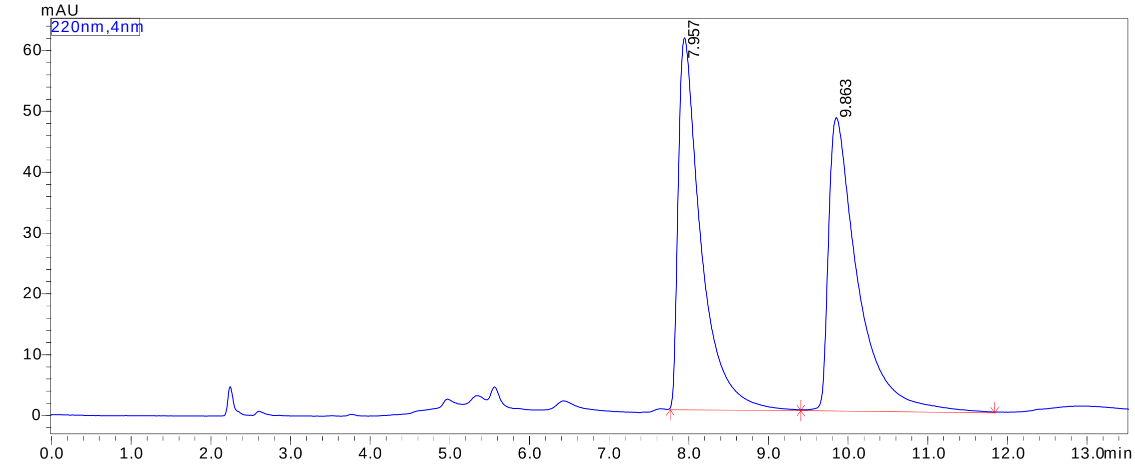

Peak#	Ret. Time	Area%
1	7.957	49.695
2	9.863	50.305
Total		100.000

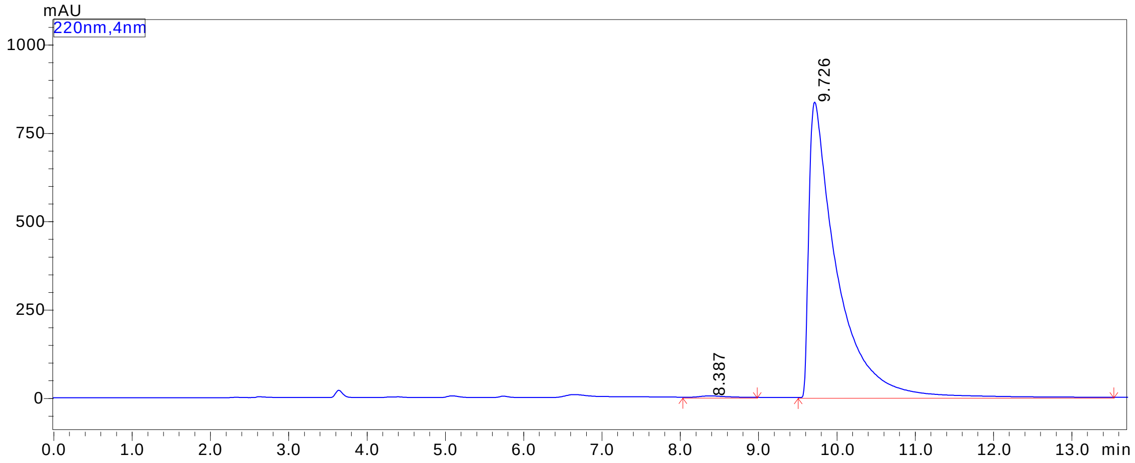

Peak#	Ret. Time	Area%
1	8.387	0.373
2	9.726	99.627
Total		100.000

HPLC trace of *rac*-**4j** and (*S*)-**4j**

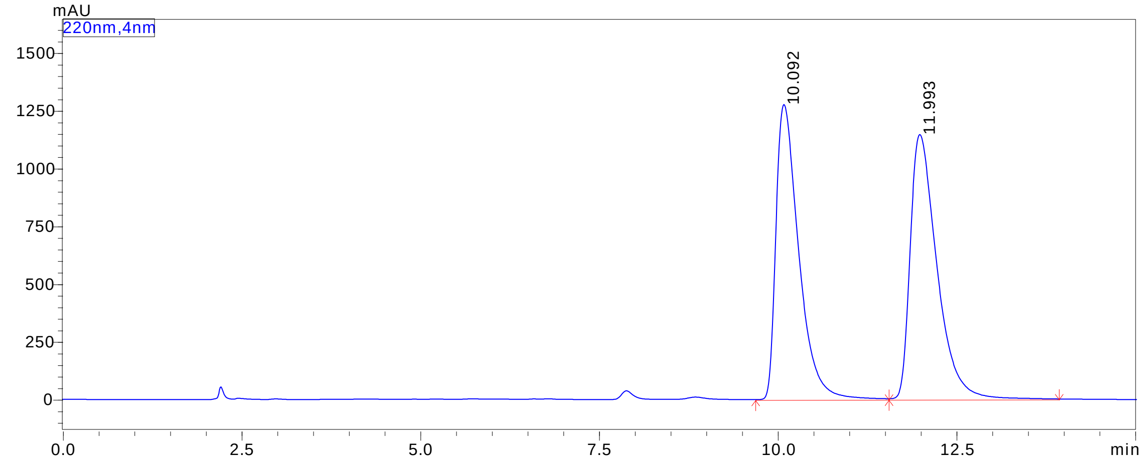

Peak#	Ret. Time	Area%
1	10.092	48.806
2	11.993	51.194
Total		100.000

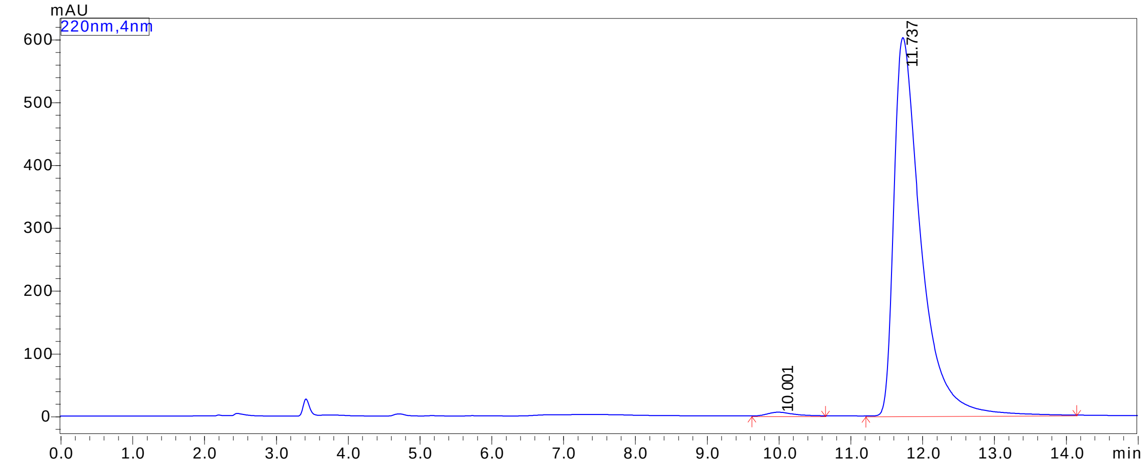

Peak#	Ret. Time	Area%
1	10.001	0.864
2	11.737	99.136
Total		100.000

HPLC trace of *rac*-**4k** and (*R*)-**4k**

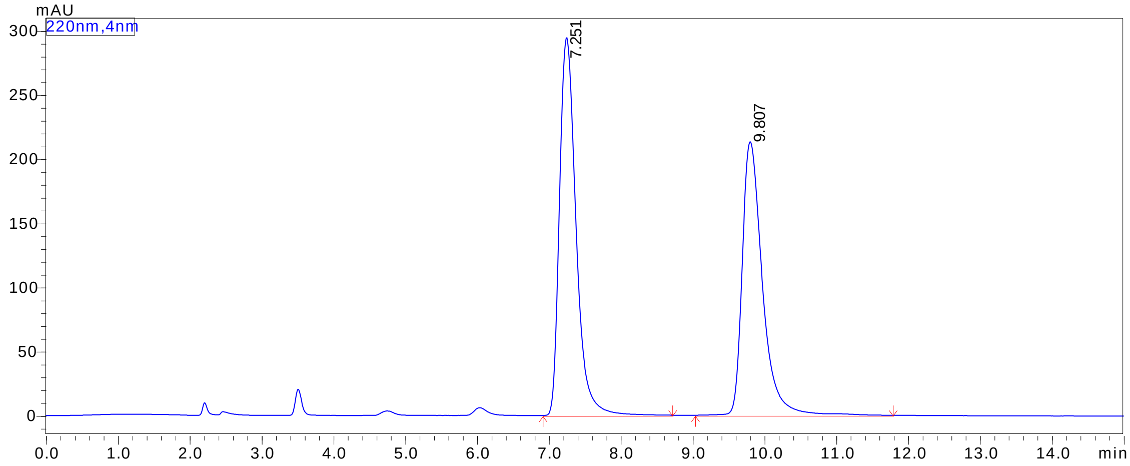

Peak#	Ret. Time	Area%
1	7.251	53.098
2	9.807	46.902
Total		100.000

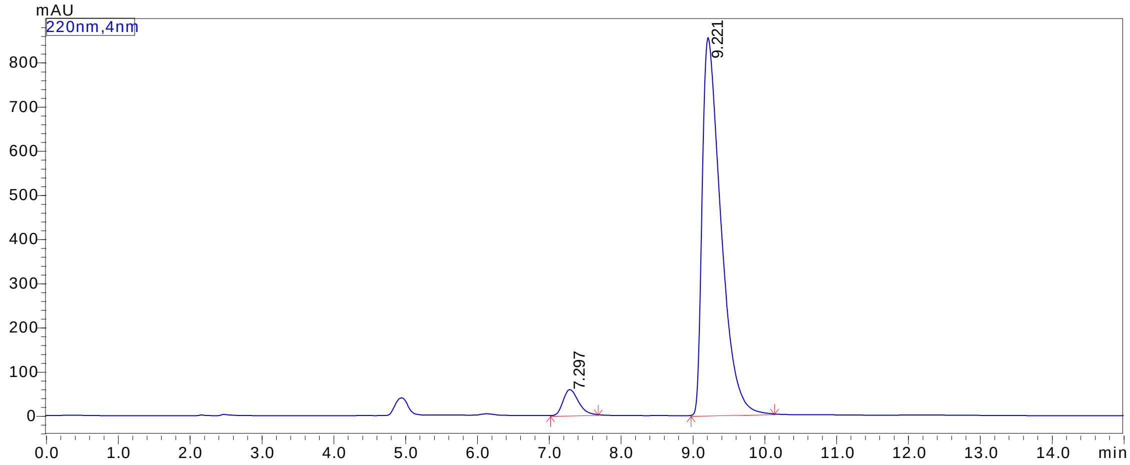

Peak#	Ret. Time	Area%
1	7.297	5.199
2	9.221	94.801
Total		100.000

HPLC trace of *rac*-**4l** and (*R*)-**4l**

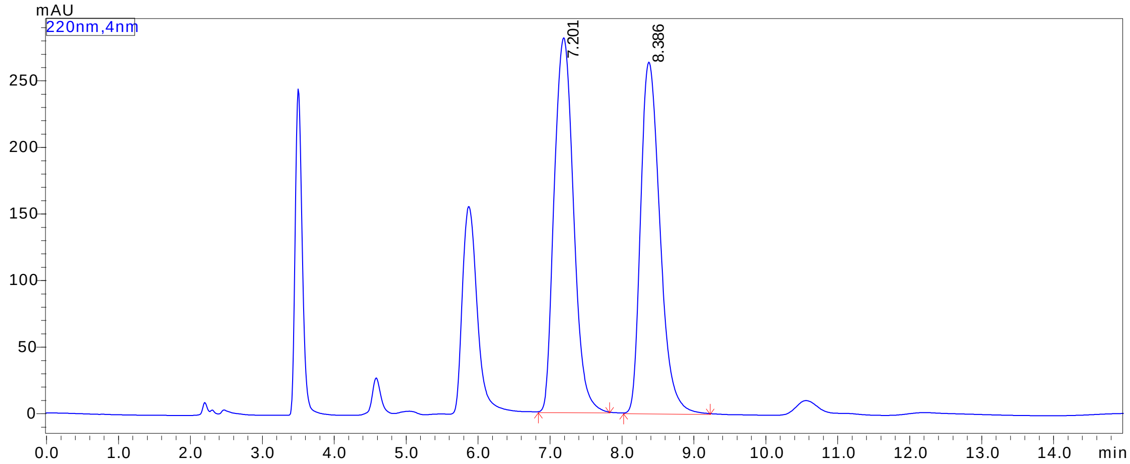

Peak#	Ret. Time	Area%
1	7.201	51.548
2	8.386	48.452
Total		100.000

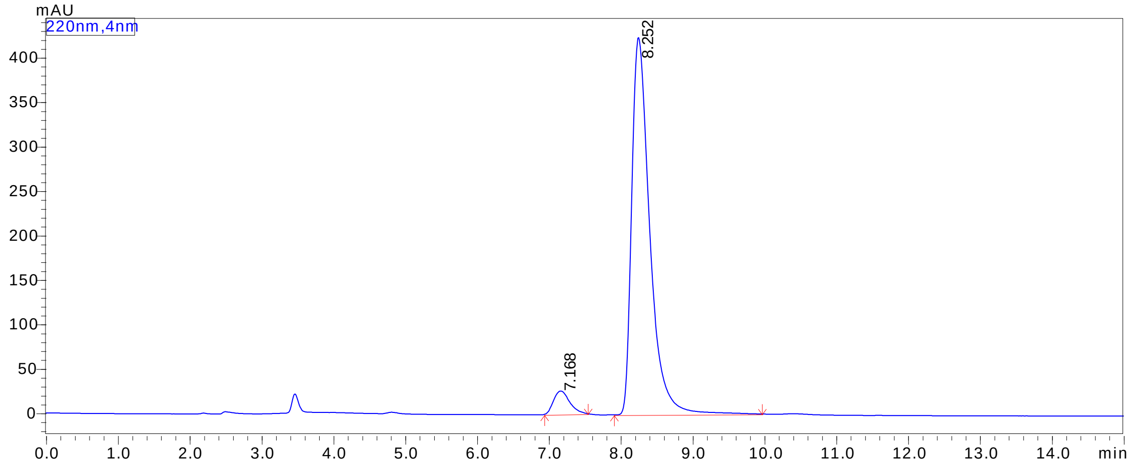

Peak#	Ret. Time	Area%
1	7.168	5.134
2	8.252	94.866
Total		100.000

HPLC trace of *rac*-**4m** and (*R*)-**4m**

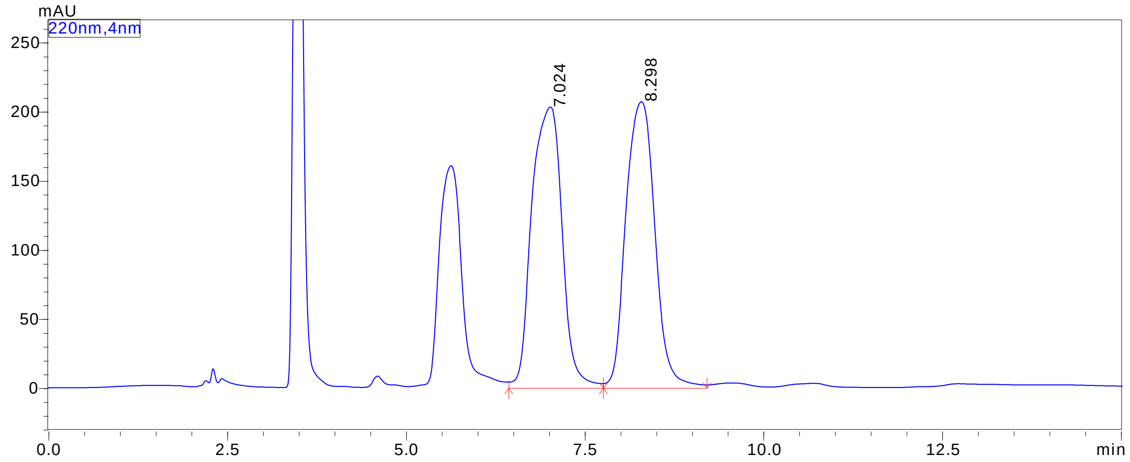

Peak#	Ret. Time	Area%
1	7.024	50.578
2	8.298	49.422
Total		100.000

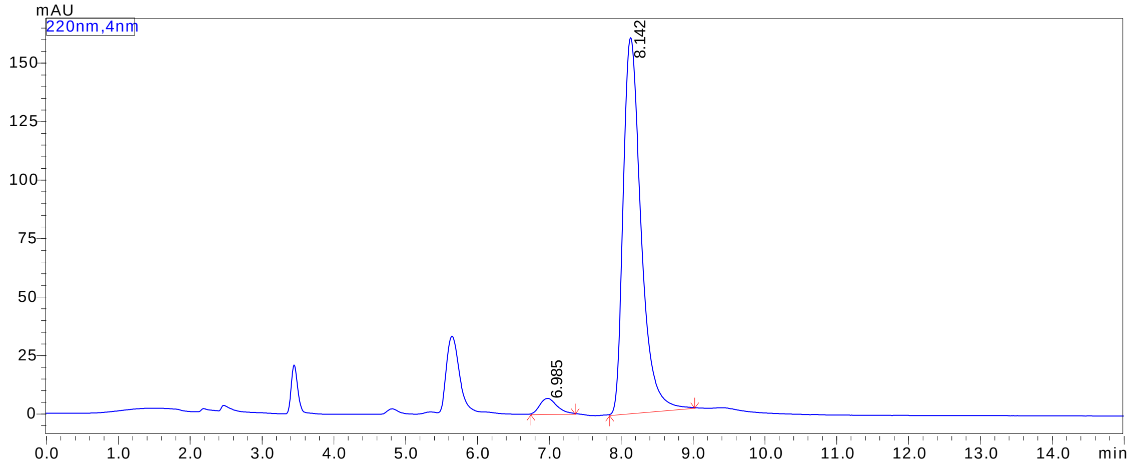

Peak#	Ret. Time	Area%
1	6.985	3.699
2	8.142	96.301
Total		100.000

HPLC trace of *rac*-**4n** and (*R*)-**4n**

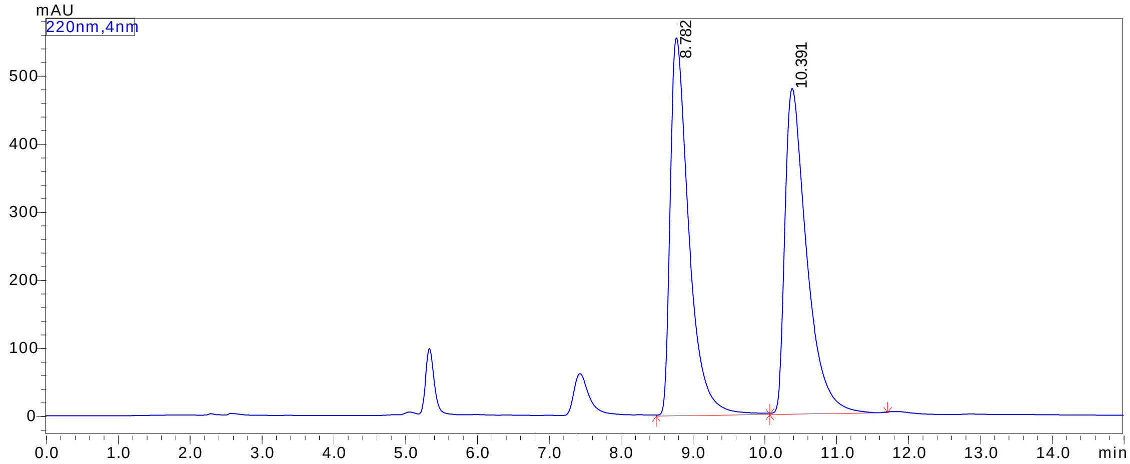

Peak#	Ret. Time	Area%
1	8.782	49.946
2	10.391	50.054
Total		100.000

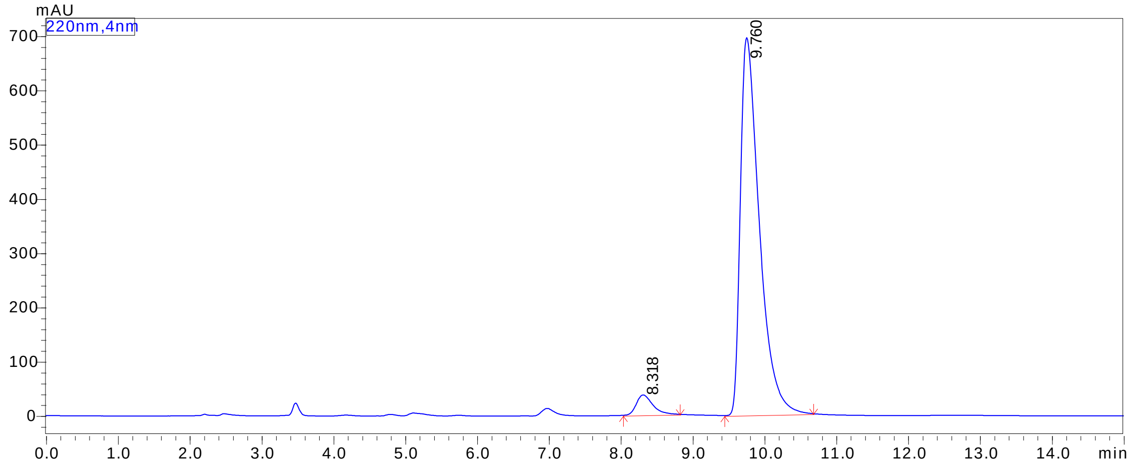

Peak#	Ret. Time	Area%
1	8.318	4.369
2	9.760	95.631
Total		100.000

HPLC trace of *rac*-**4o** and (*R*)-**4o**

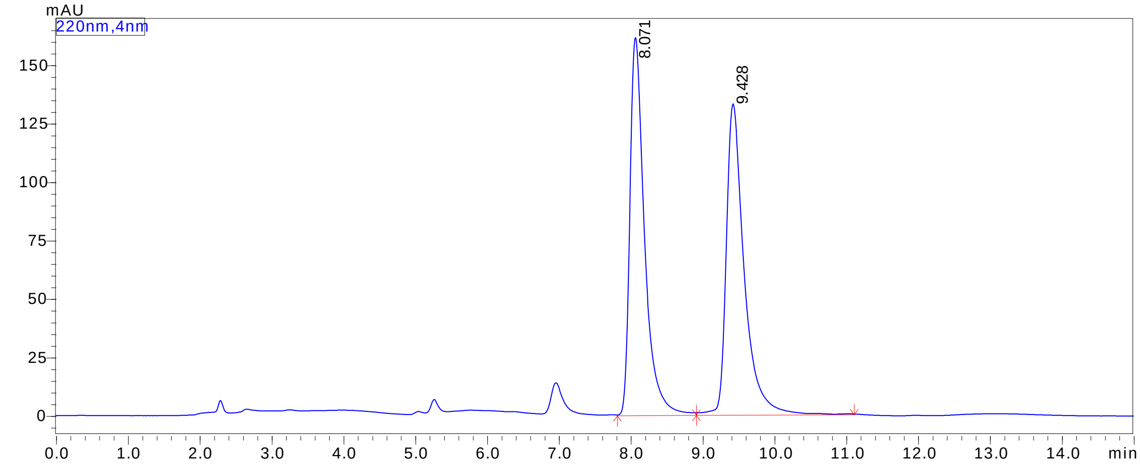

Peak#	Ret. Time	Area%
1	8.071	49.967
2	9.428	50.033
Total		100.000

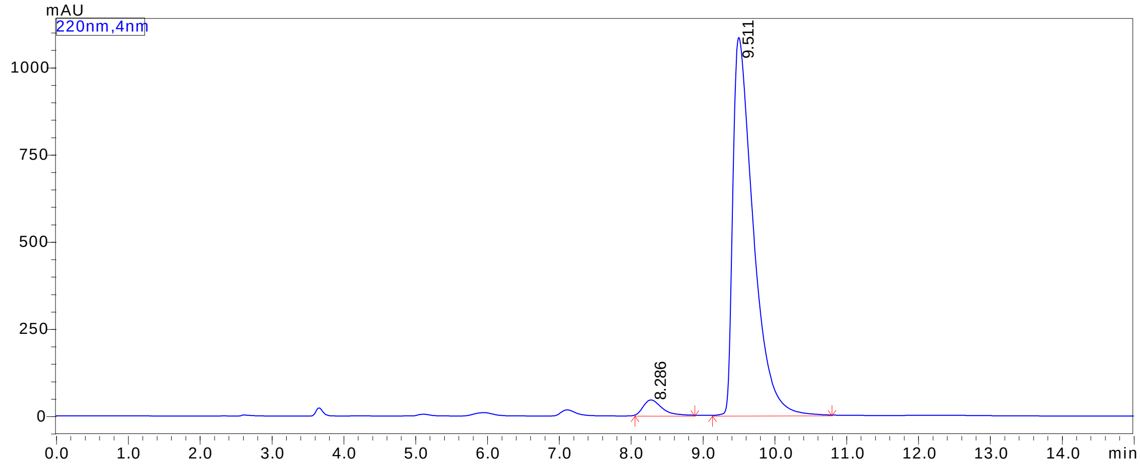

Peak#	Ret. Time	Area%
1	8.286	3.485
2	9.511	96.515
Total		100.000

HPLC trace of *rac*-**4p** and (*R*)-**4p**

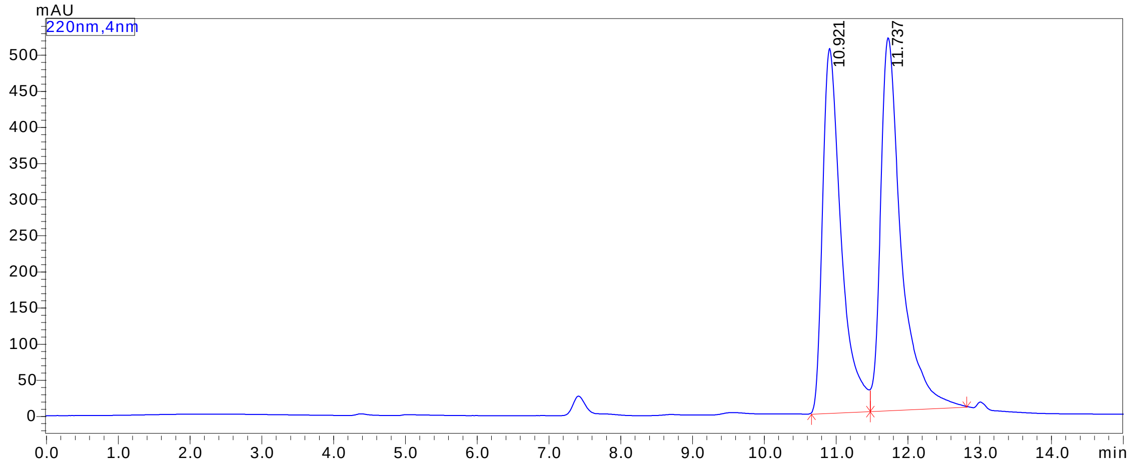

Peak#	Ret. Time	Area%
1	10.921	46.923
2	11.737	53.077
Total		100.000

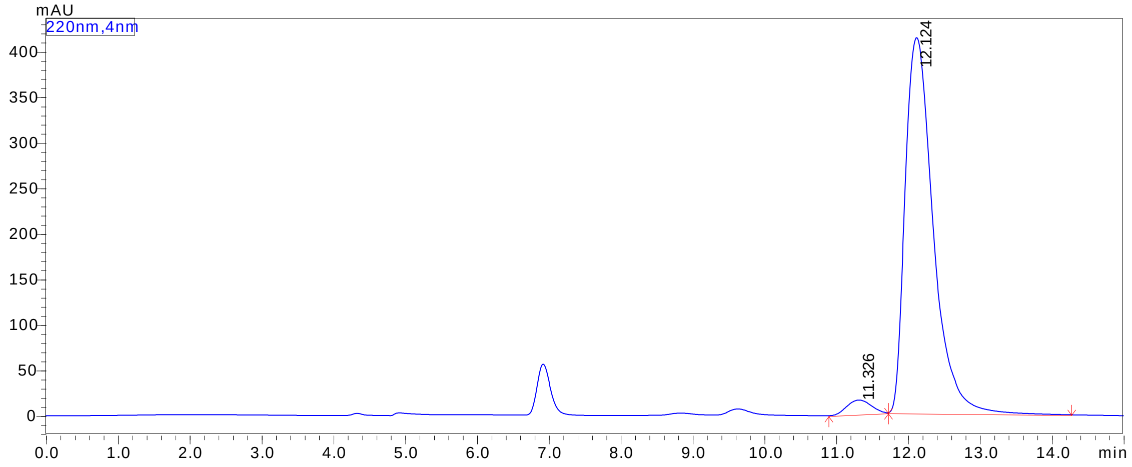

Peak#	Ret. Time	Area%
1	11.326	3.101
2	12.124	96.899
Total		100.000

HPLC trace of *rac*-**4q** and (*R*)-**4q**

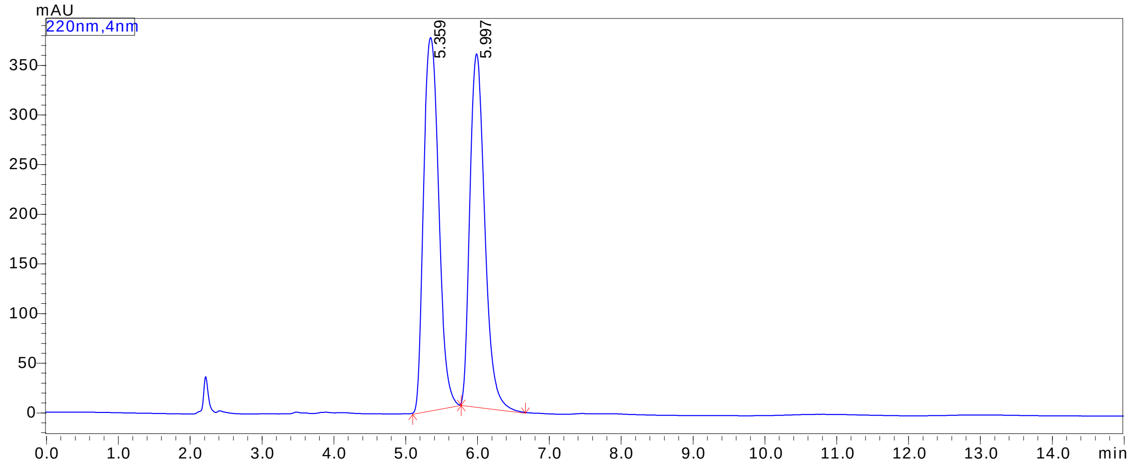

Peak#	Ret. Time	Area%
1	5.359	51.797
2	5.997	48.203
Total		100.000

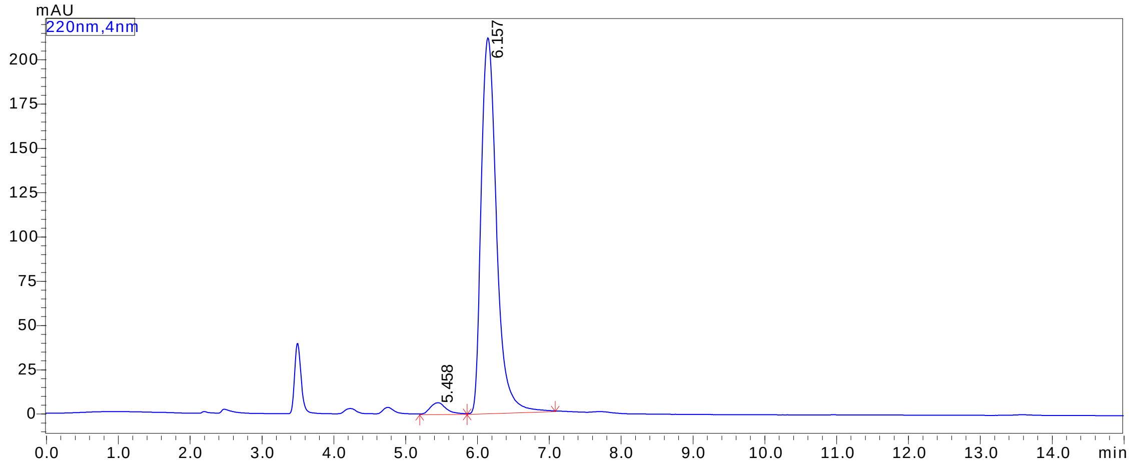

Peak#	Ret. Time	Area%
1	5.458	2.553
2	6.157	97.447
Total		100.000

HPLC trace of *rac*-**4r** and (*R*)-**4r**

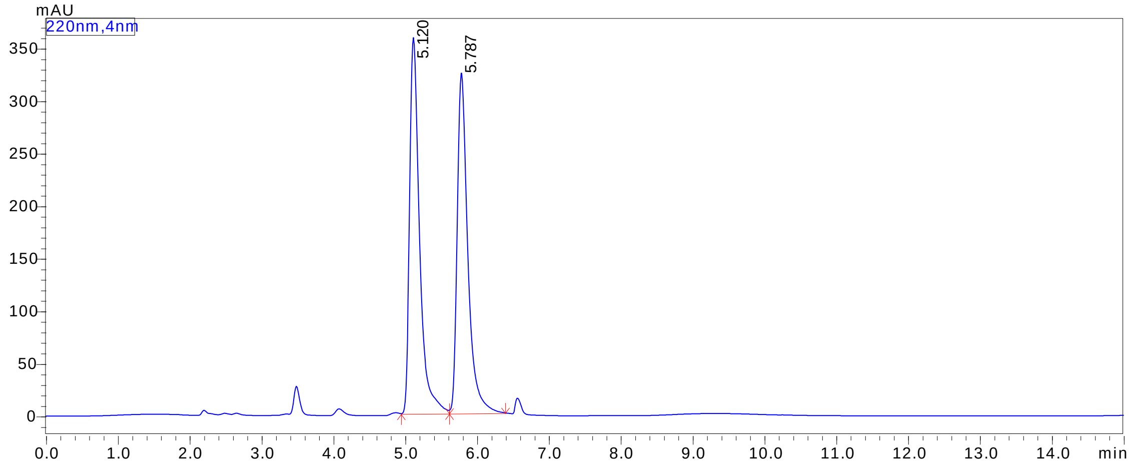

Peak#	Ret. Time	Area%
1	5.120	50.734
2	5.787	49.266
Total		100.000

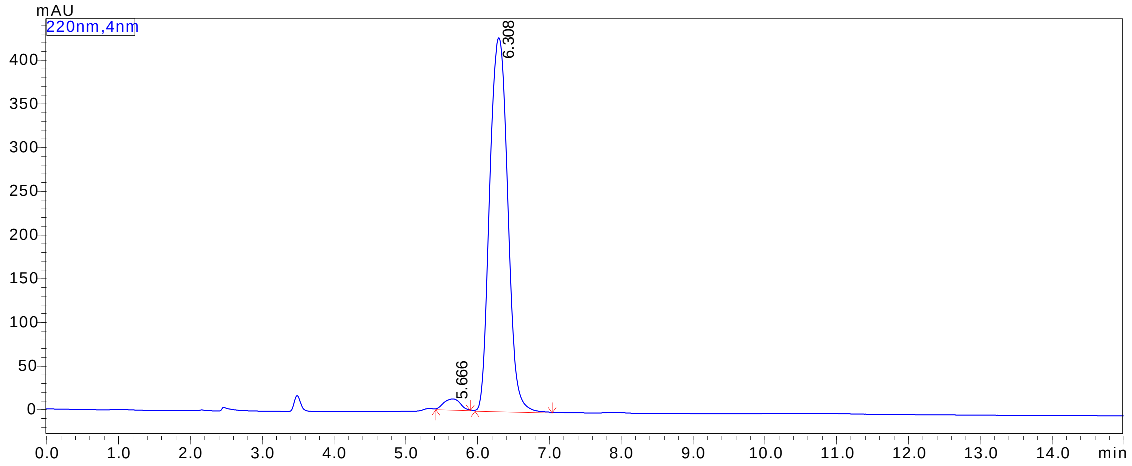

Peak#	Ret. Time	Area%
1	5.666	2.371
2	6.308	97.629
Total		100.000

HPLC trace of *rac*-**4s** and (*R*)-**4s**

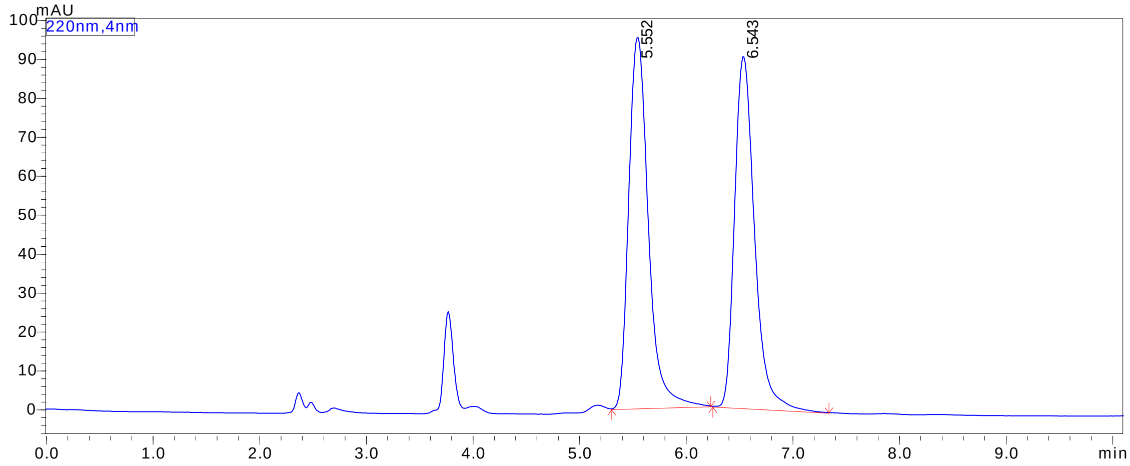

Peak#	Ret. Time	Area%
1	5.552	50.941
2	6.543	49.059
Total		100.000

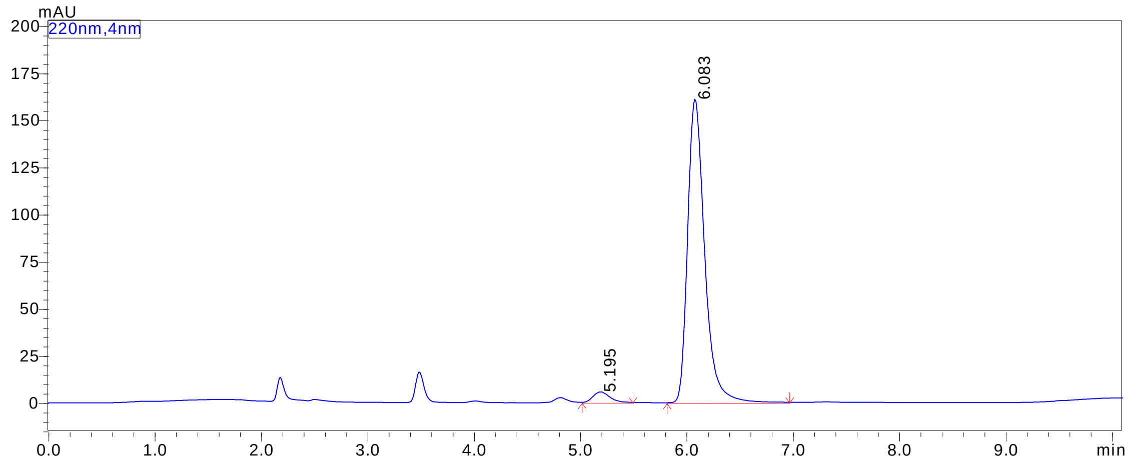

Peak#	Ret. Time	Area%
1	5.195	3.197
2	6.083	96.803
Total		100.000

HPLC trace of *rac*-**4t** and (*R*)-**4t**

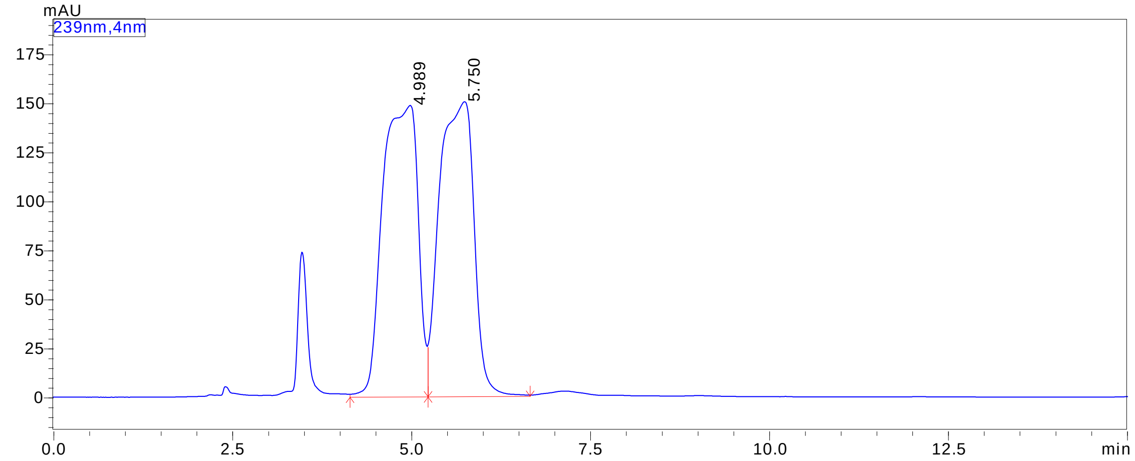

Peak#	Ret. Time	Area%
1	4.989	50.290
2	5.750	49.710
Total		100.000

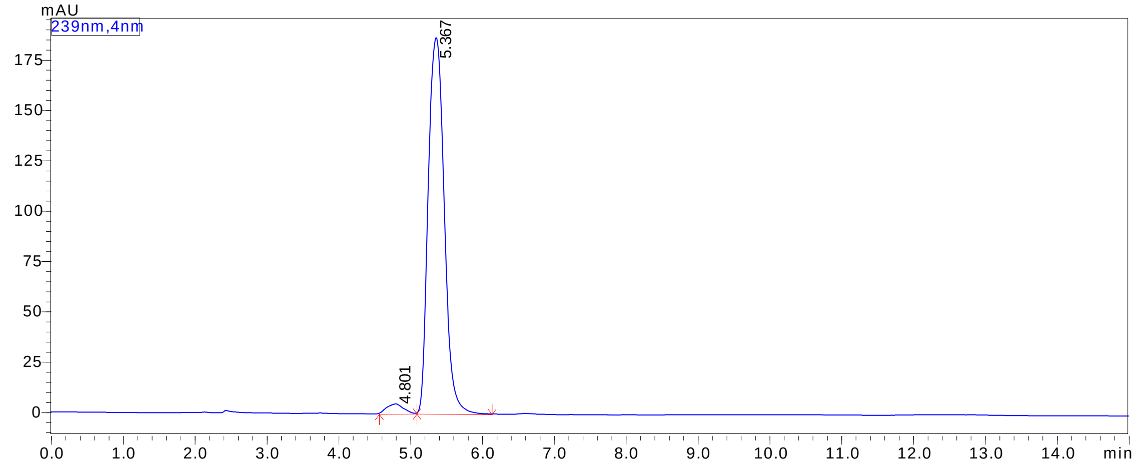

Peak#	Ret. Time	Area%
1	4.801	2.432
2	5.367	97.568
Total		100.000

HPLC trace of *rac*-**4u** and (*R*)-**4u**

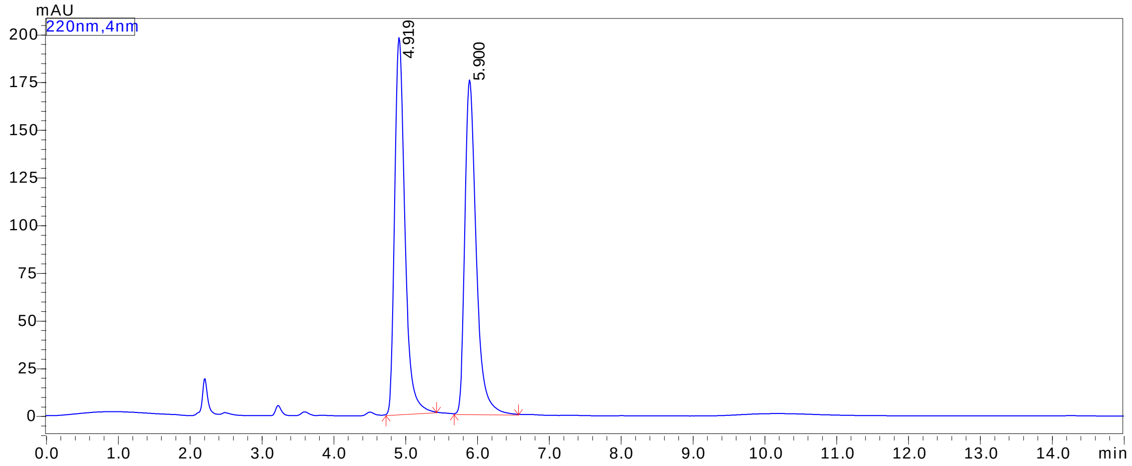

Peak#	Ret. Time	Area%
1	4.919	50.426
2	5.900	49.574
Total		100.000

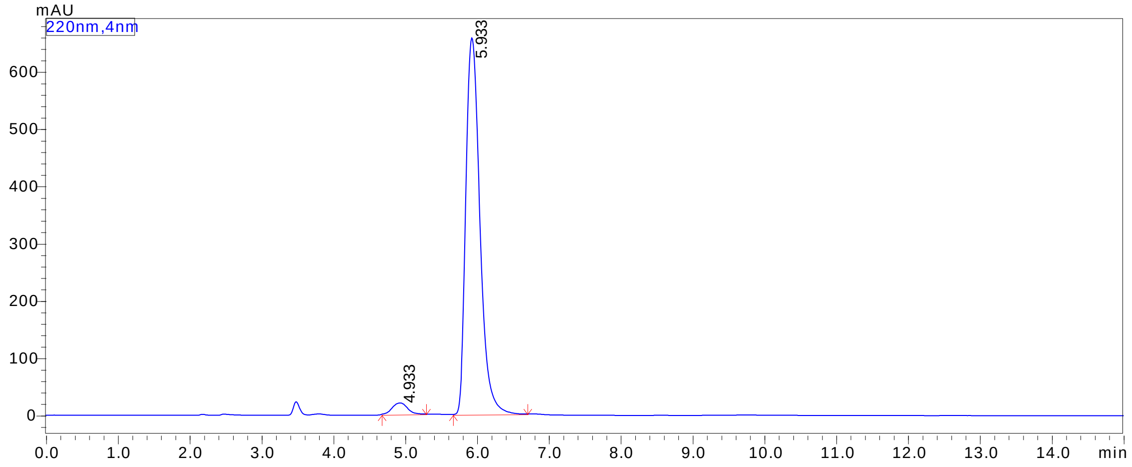

Peak#	Ret. Time	Area%
1	4.933	3.123
2	5.933	96.877
Total		100.000

HPLC trace of *rac*-**4v** and (*R*)-**4v**

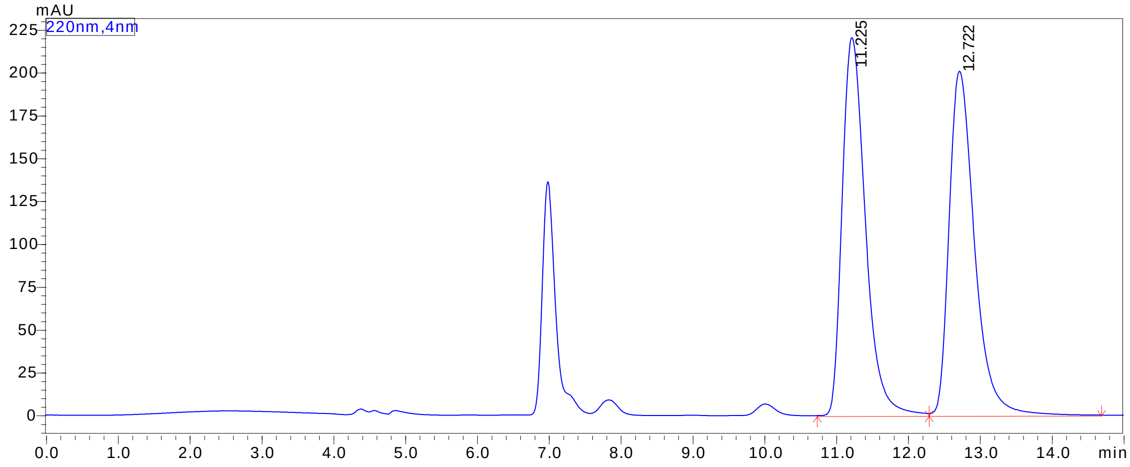

Peak#	Ret. Time	Area%
1	11.225	50.198
2	12.722	49.802
Total		100.000

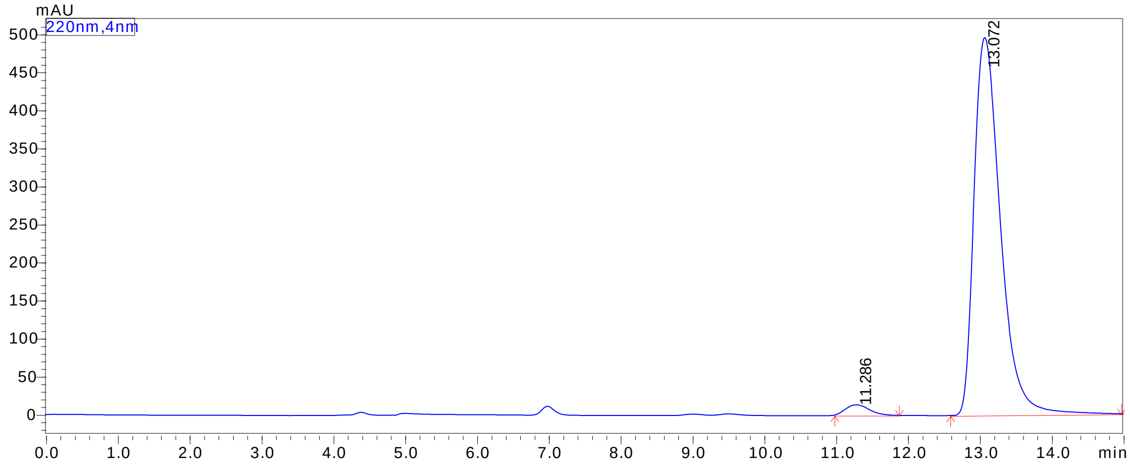

Peak#	Ret. Time	Area%
1	11.286	2.388
2	13.072	97.612
Total		100.000

HPLC trace of *rac*-**4w** and (*R*)-**4w**

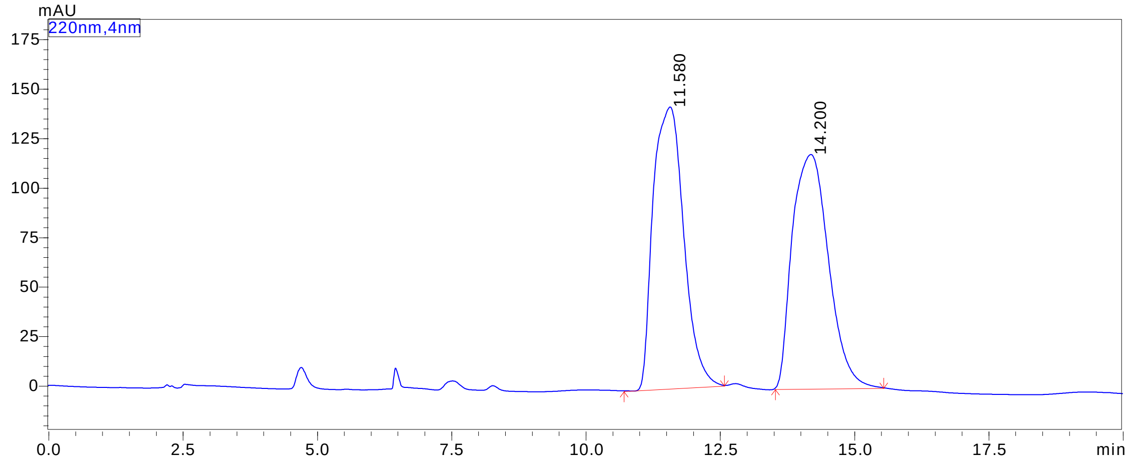

Peak#	Ret. Time	Area%
1	11.580	50.365
2	14.200	49.635
Total		100.000

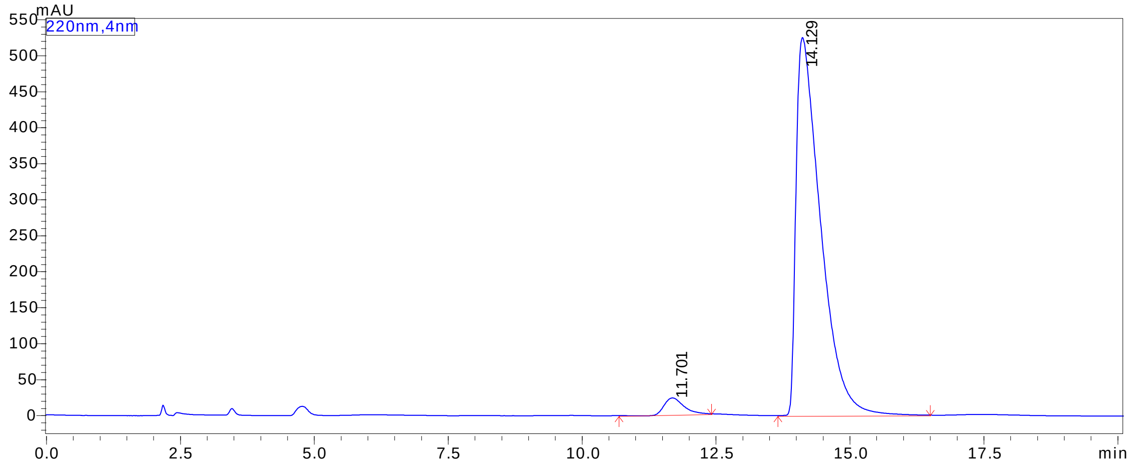

Peak#	Ret. Time	Area%
1	11.701	3.176
2	14.129	96.824
Total		100.000

HPLC trace of *rac*-**4x** and (*R*)-**4x**

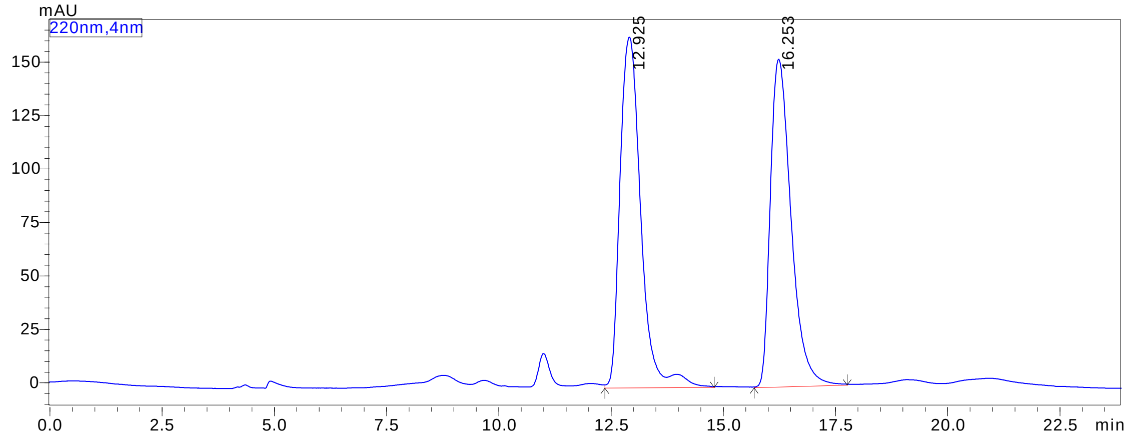

Peak#	Ret. Time	Area%
1	12.925	51.827
2	16.253	48.173
Total		100.000

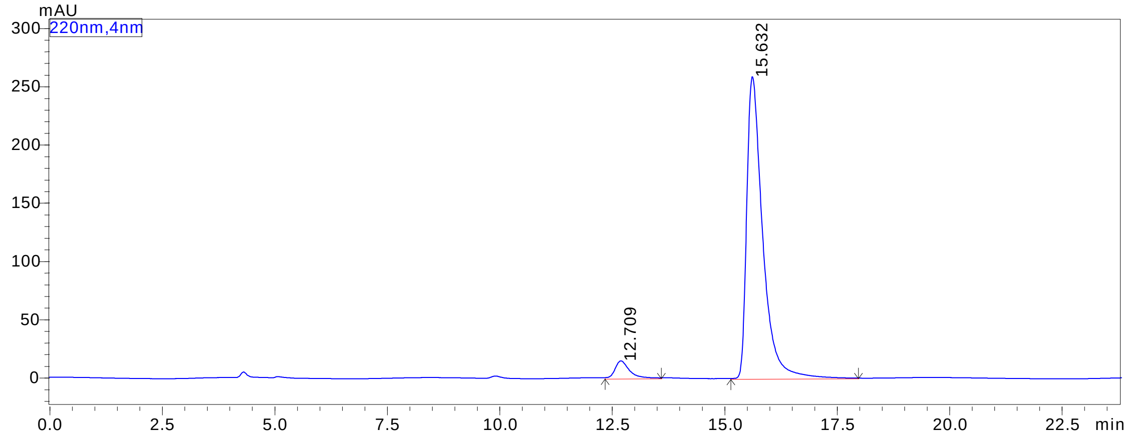

Peak#	Ret. Time	Area%
1	12.709	4.528
2	15.632	95.472
Total		100.000

HPLC trace of *rac*-**4y** and (*R*)-**4y**

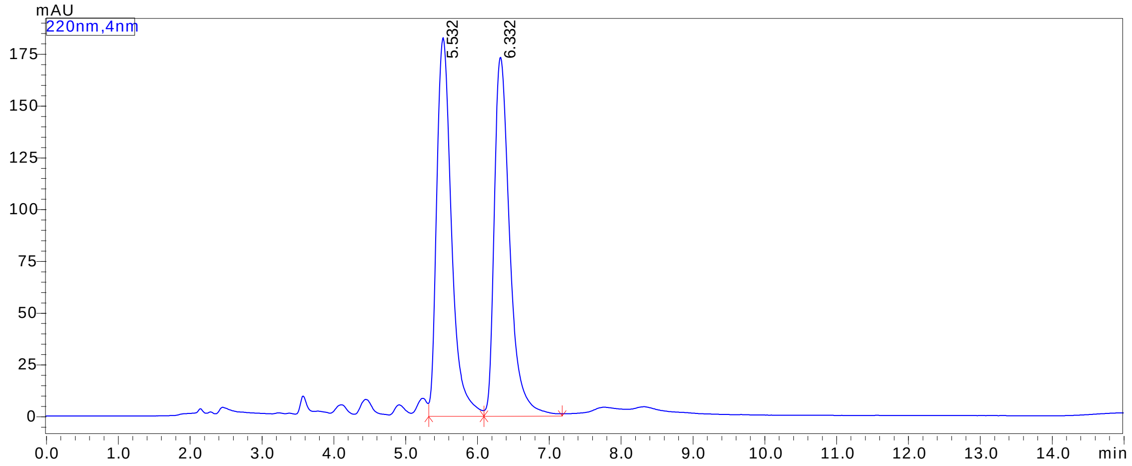

Peak#	Ret. Time	Area%
1	5.532	50.243
2	6.332	49.757
Total		100.000

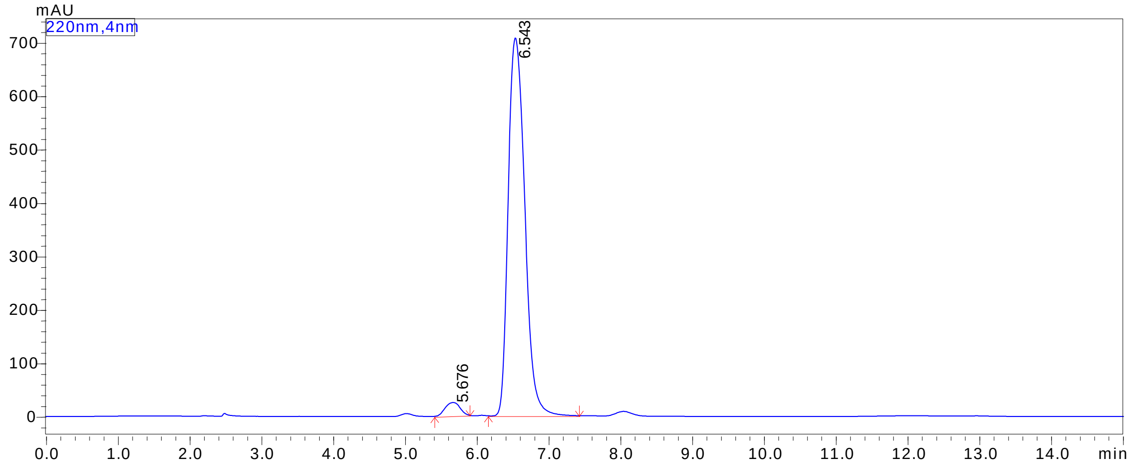

Peak#	Ret. Time	Area%
1	5.676	3.018
2	6.543	96.982
Total		100.000

HPLC trace of *rac*-**4za** and (2*R*,4*S*)-**4za**

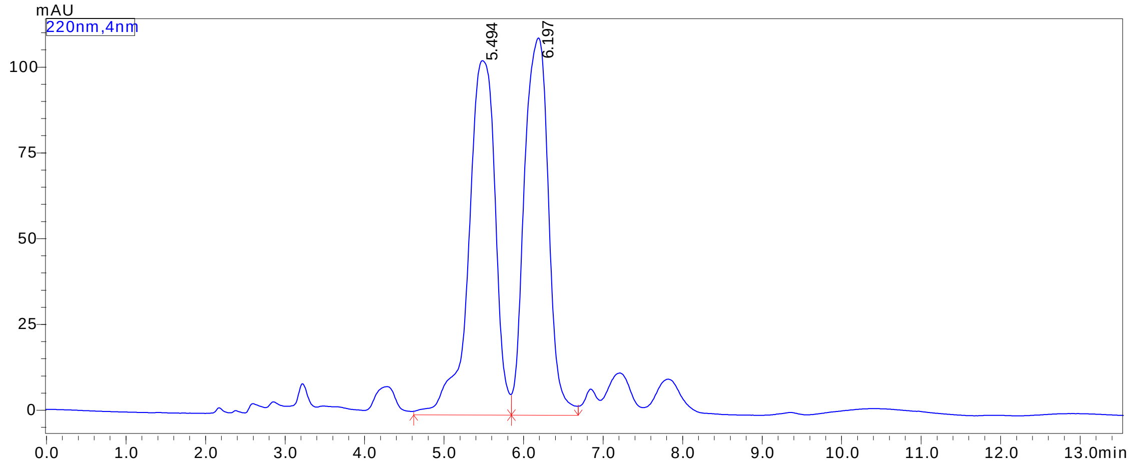

Peak#	Ret. Time	Area%
1	5.494	50.964
2	6.197	49.036
Total		100.000

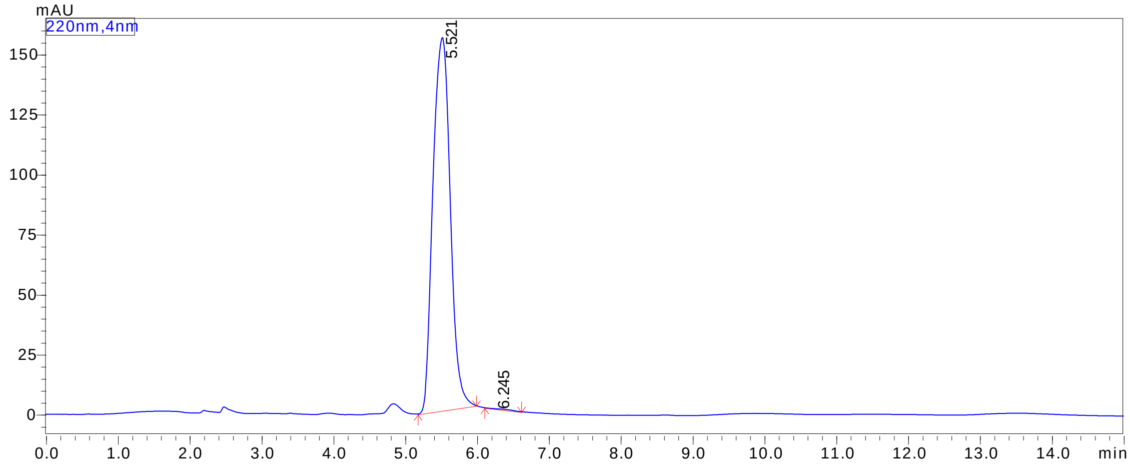

Peak#	Ret. Time	Area%
1	5.870	98.935
2	6.833	1.065
Total		100.000

HPLC trace of *rac*-**4zb** and (2*R*,4*R*)-**4zb**

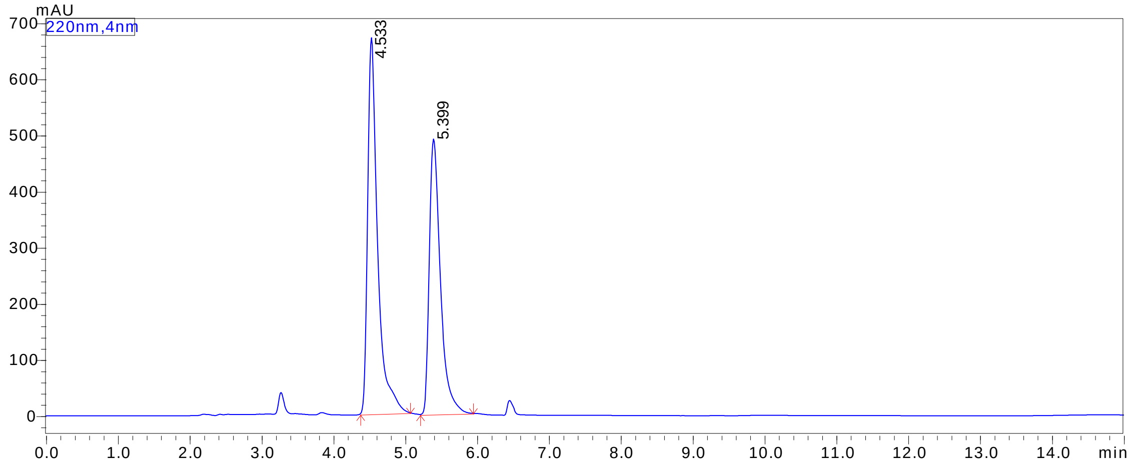

Peak#	Ret. Time	Area%
1	4.533	55.443
2	5.399	44.557
Total		100.000

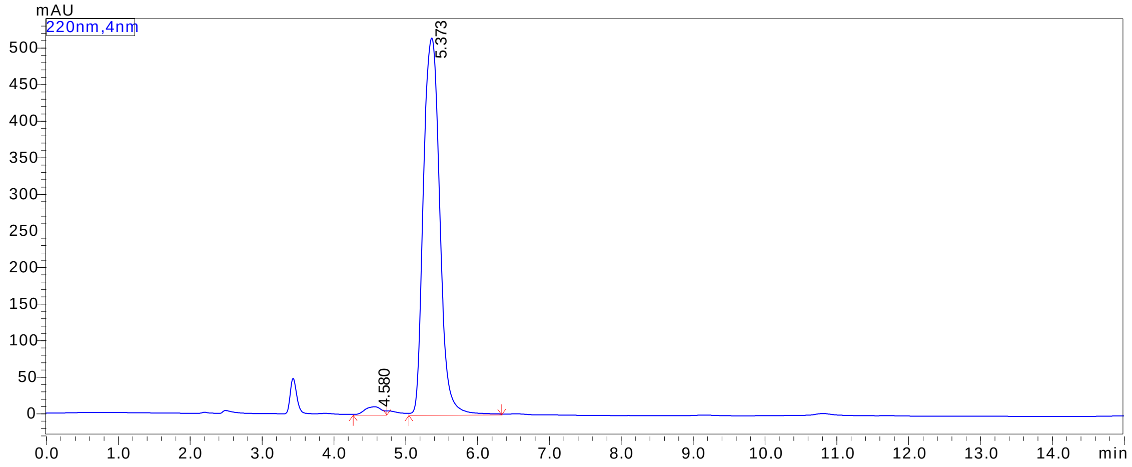

Peak#	Ret. Time	Area%
1	4.580	2.178
2	5.373	97.822
Total		100.000

HPLC trace of *rac*-**5a** and (*S*)-**5a**

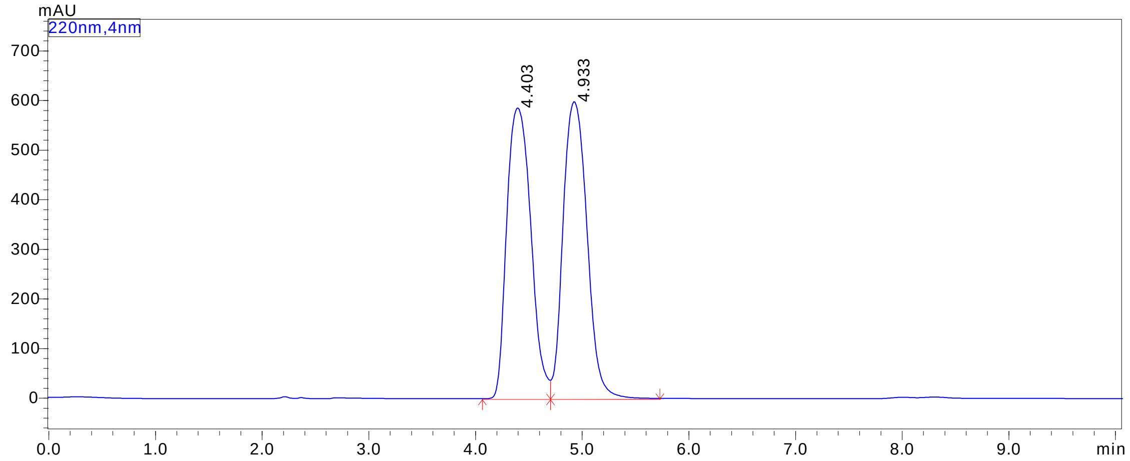

Peak#	Ret. Time	Area%
1	4.403	49.656
2	4.933	50.344
Total		100.000

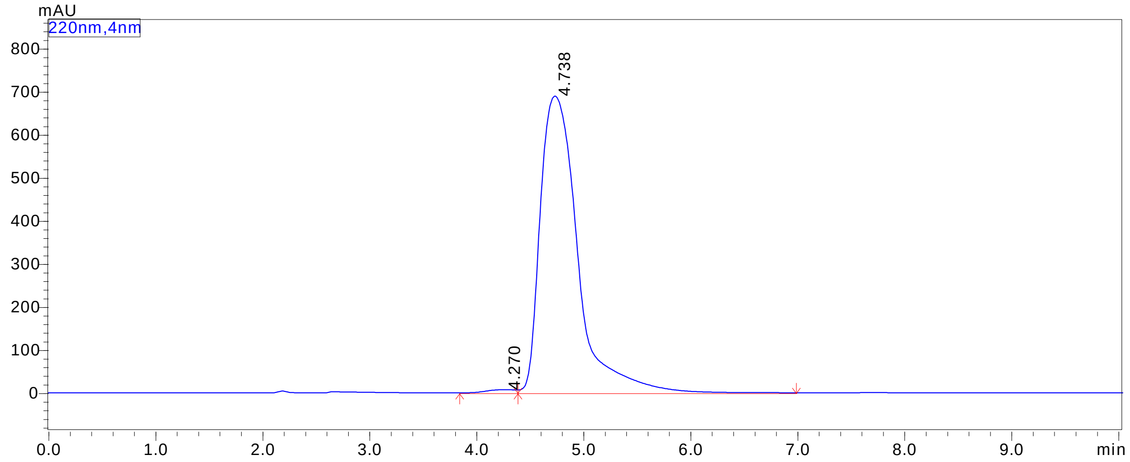

Peak#	Ret. Time	Area%
1	4.270	0.451
2	4.738	99.549
Total		100.000

HPLC trace of *rac*-**5b** and (*S*)-**5b**

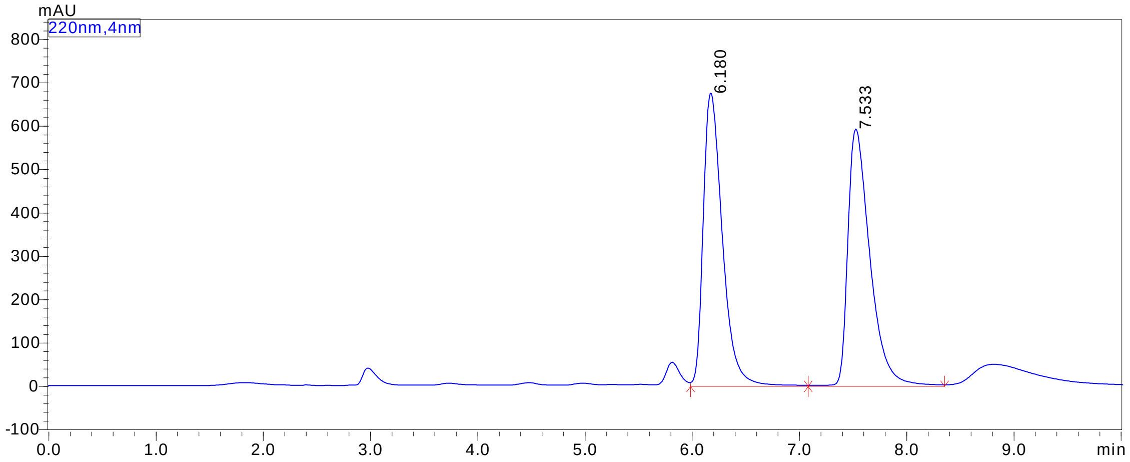

Peak#	Ret. Time	Area%
1	6.180	49.803
2	7.533	50.197
Total		100.000

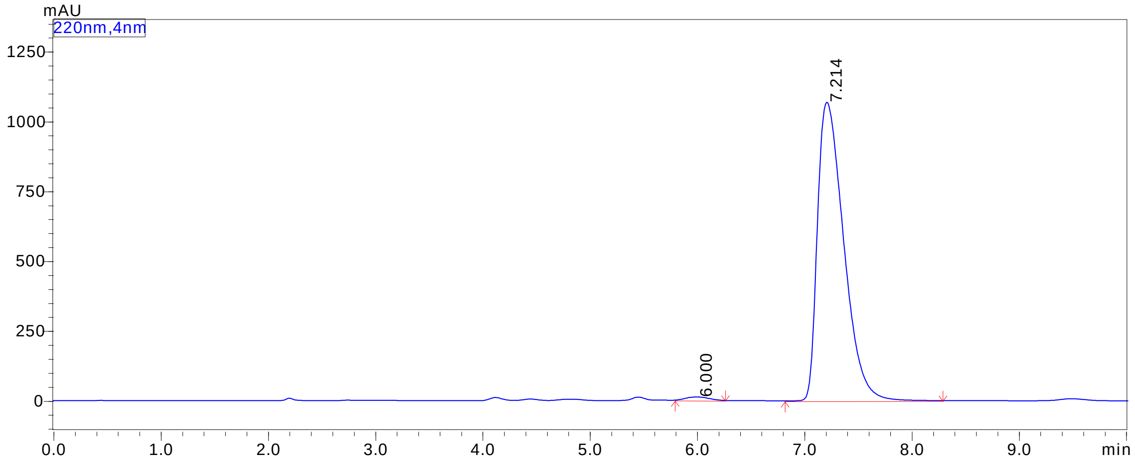

Peak#	Ret. Time	Area%
1	6.000	0.558
2	7.214	99.442
Total		100.000

HPLC trace of *rac*-**6** and (*S*)-**6**

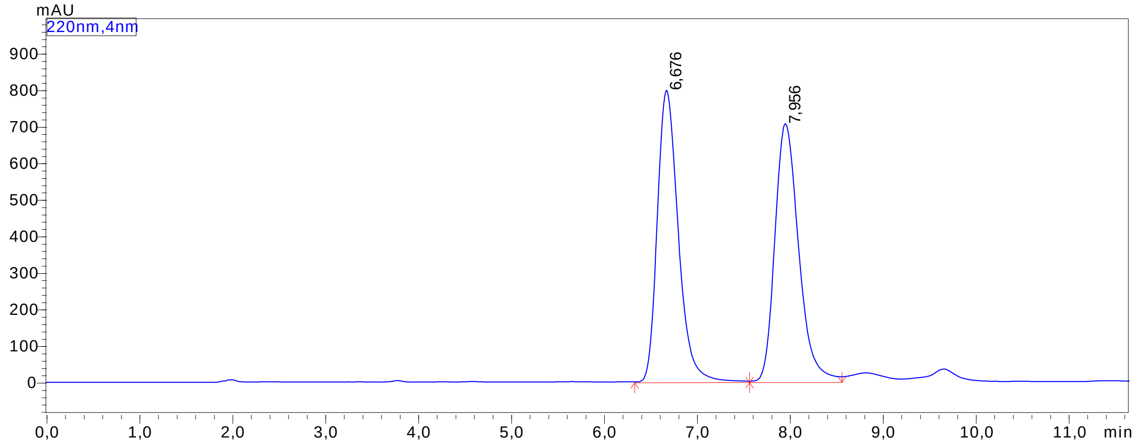

Peak#	Ret. Time	Area%
1	6,676	49,865
2	7,956	50,135
Total		100,000

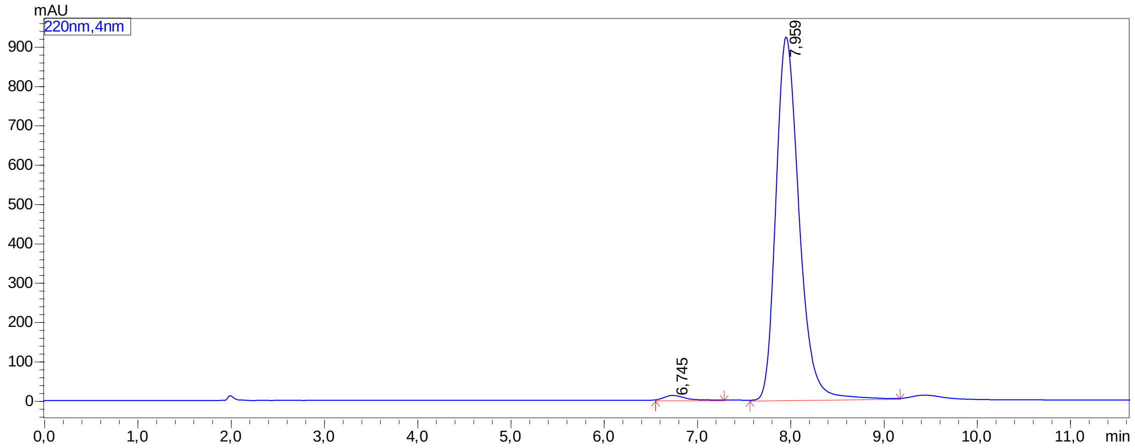

Peak#	Ret. Time	Area%
1	6,745	1,060
2	7,959	98,940
Total		100,000

HPLC trace of *rac*-**7a** and (*R*, 2*S*, 3*R*)-**7a**

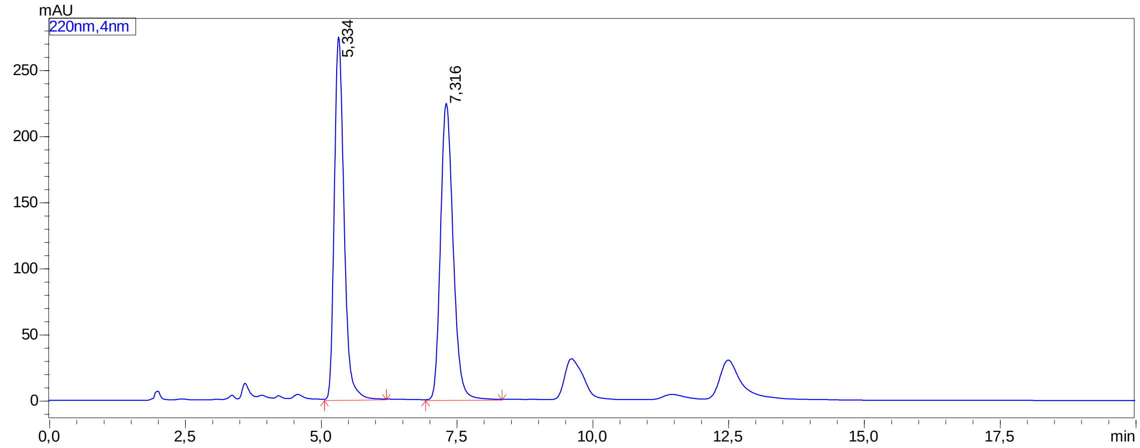

Peak#	Ret. Time	Area%
1	5,334	48,705
2	7,316	51,295
Total		100,000

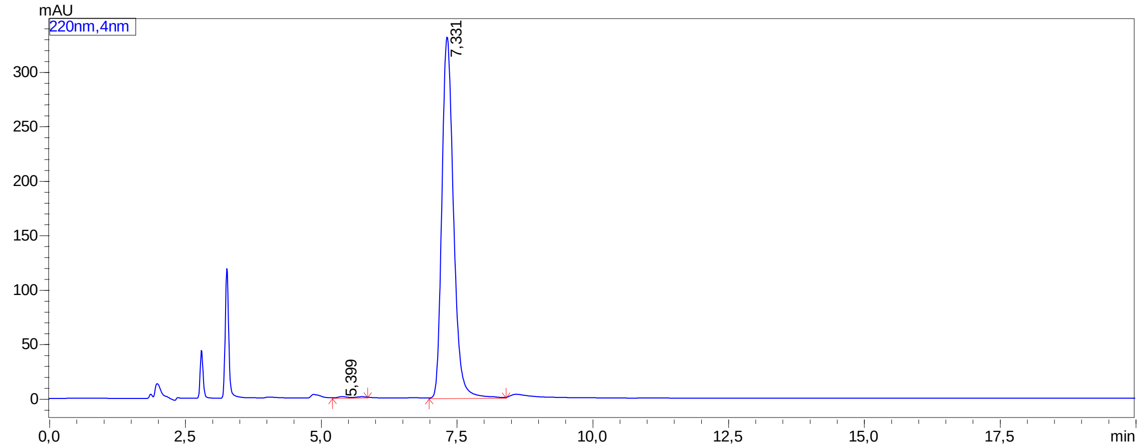

Peak#	Ret. Time	Area%
1	5,399	0,217
2	7,331	99,783
Total		100,000

HPLC trace of *rac*-**7b** and (*R*, 2*R*, 3*S*)-**7b**

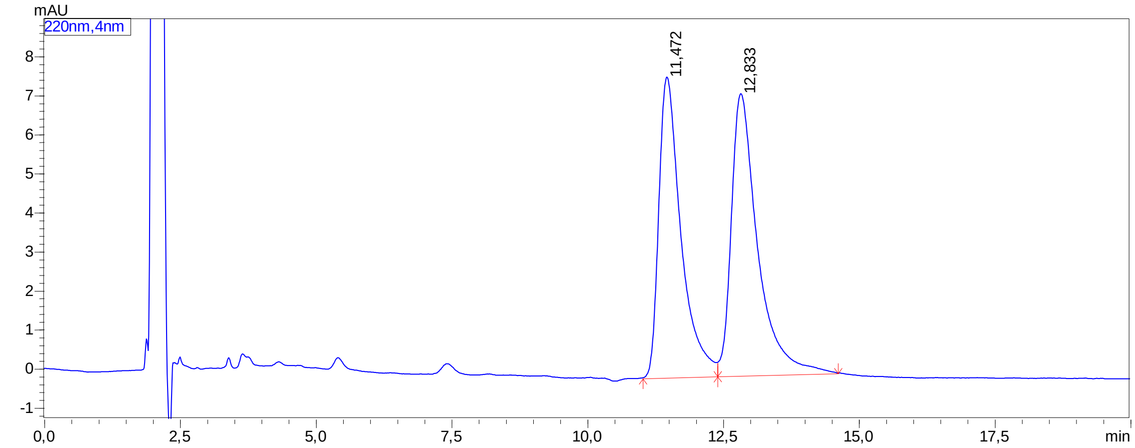

Peak#	Ret. Time	Area%
1	11,472	47,339
2	12,833	52,661
Total		100,000

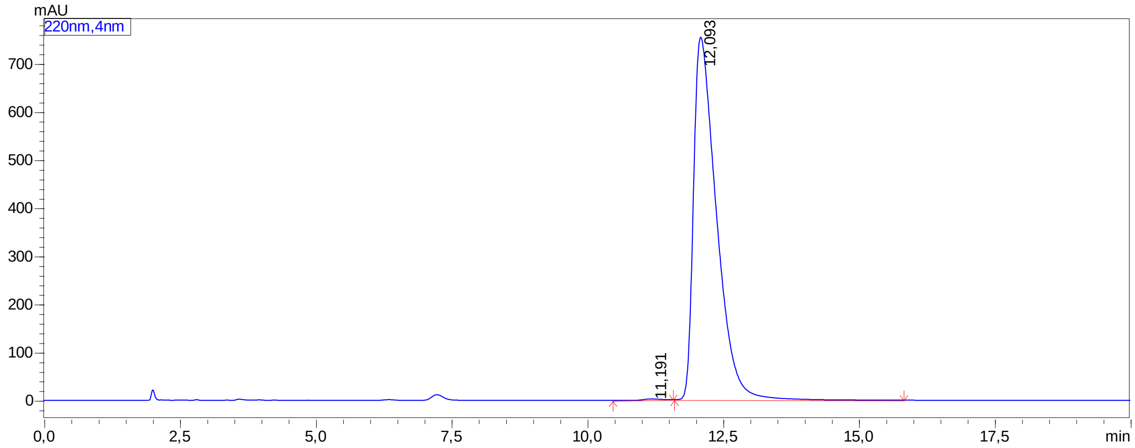

Peak#	Ret. Time	Area%
1	11,191	0,139
2	12,093	99,861
Total		100,000

HPLC trace of *rac*-**8** and (*S*)-**8**

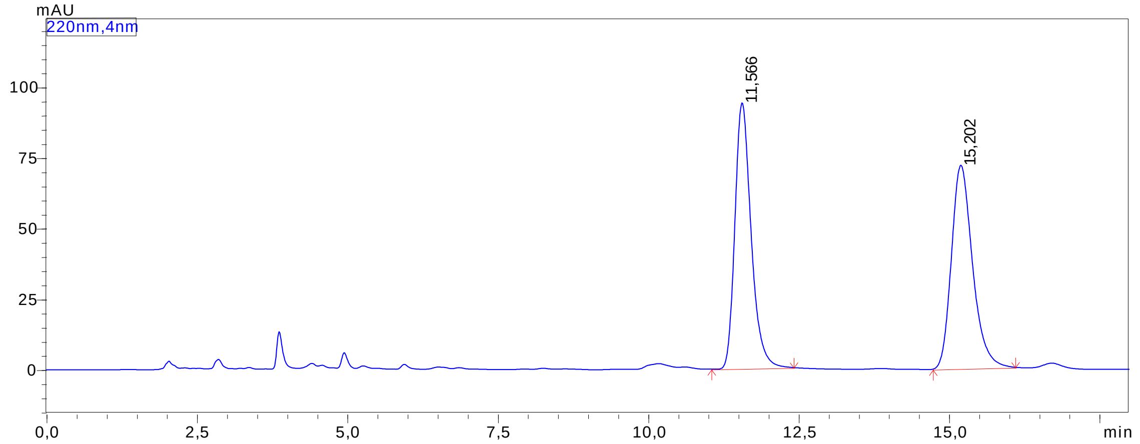

Peak#	Ret. Time	Area%
1	11,566	50,057
2	15,202	49,943
Total		100,000

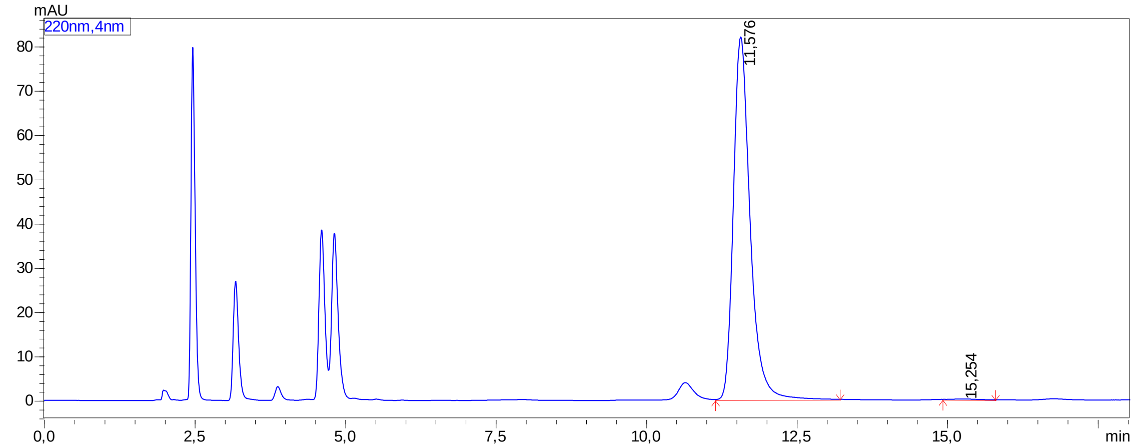

Peak#	Ret. Time	Area%
1	11,576	99,819
2	15,254	0,181
Total		100,000

HPLC trace of *rac*-**9a** and (*S*)-**9a**

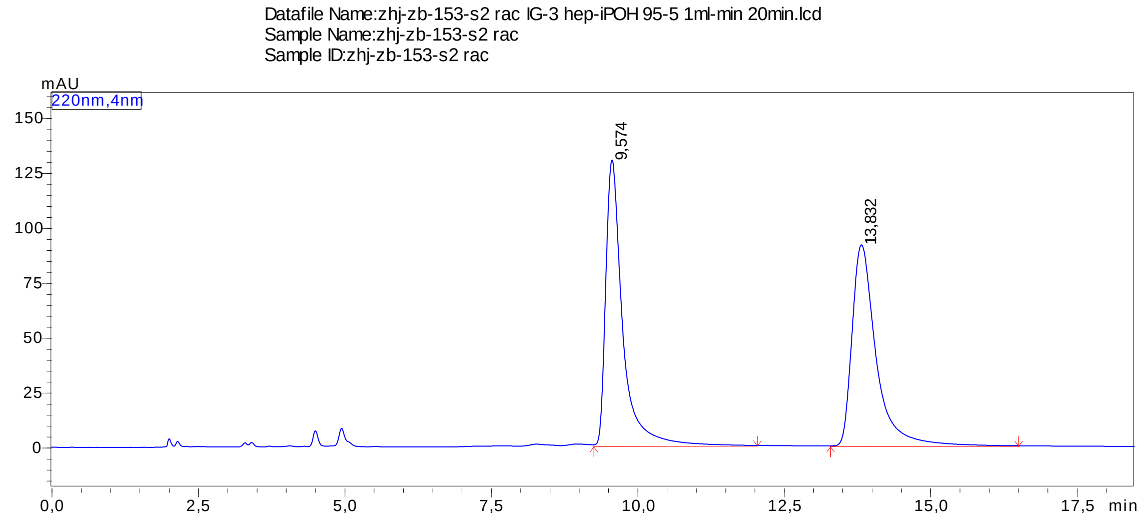

Peak#	Ret. Time	Area%
1	9,574	50,005
2	13,832	49,995
Total		100,000

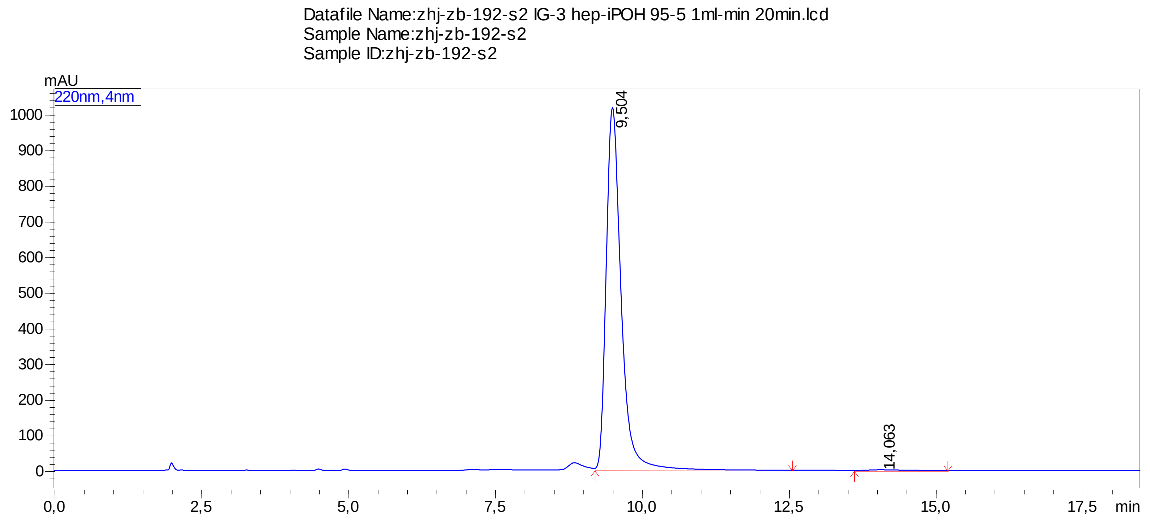

Peak#	Ret. Time	Area%
1	9,504	99,661
2	14,063	0,339
Total		100,000

HPLC trace of *rac*-**9b** and (*S*)-**9b**

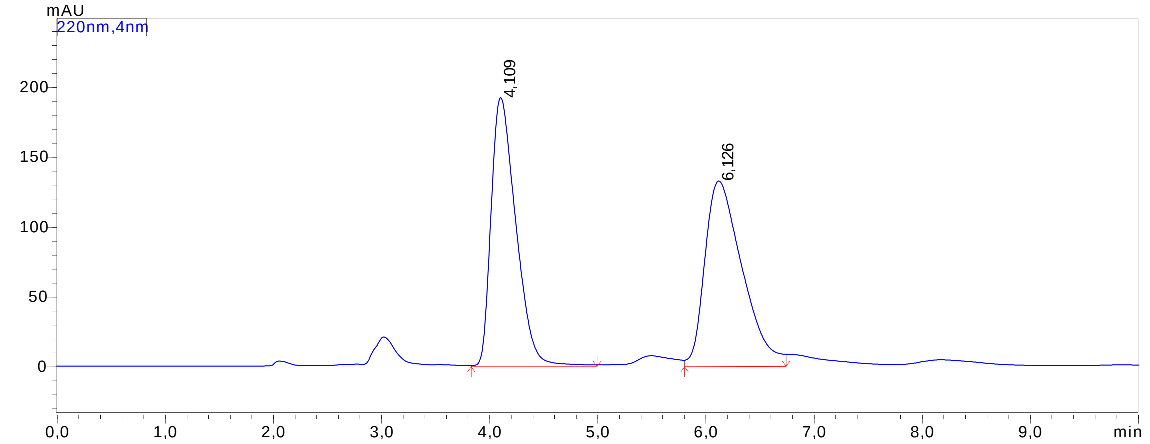

Peak#	Ret. Time	Area%
1	4,109	49,040
2	6,126	50,960
Total		100,000

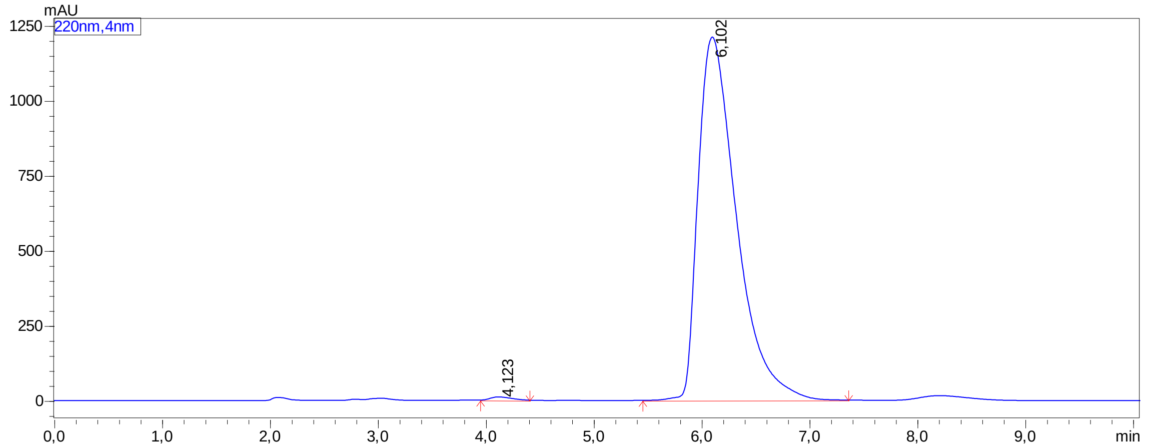

Peak#	Ret. Time	Area%
1	4,123	0,474
2	6,102	99,526
Total		100,000

HPLC trace of *rac*-**10** and (2*S*, 4*S*)-**10**

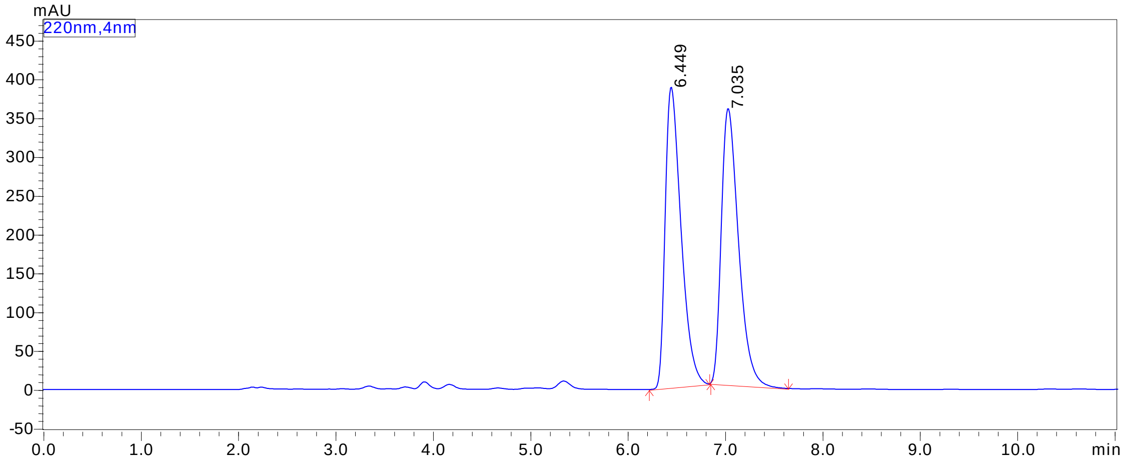

Peak#	Ret. Time	Area%
1	6.449	50.023
2	7.035	49.977
Total		100.000

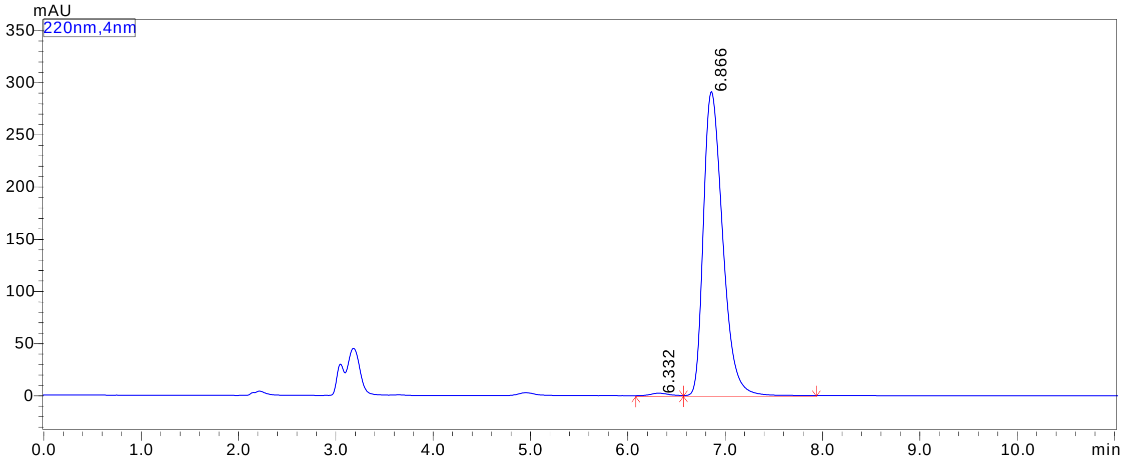

Peak#	Ret. Time	Area%
1	6.332	0.420
2	6.866	99.580
Total		100.000

HPLC trace of *rac*-**11** and (2*S*, 4*S*)-**11**

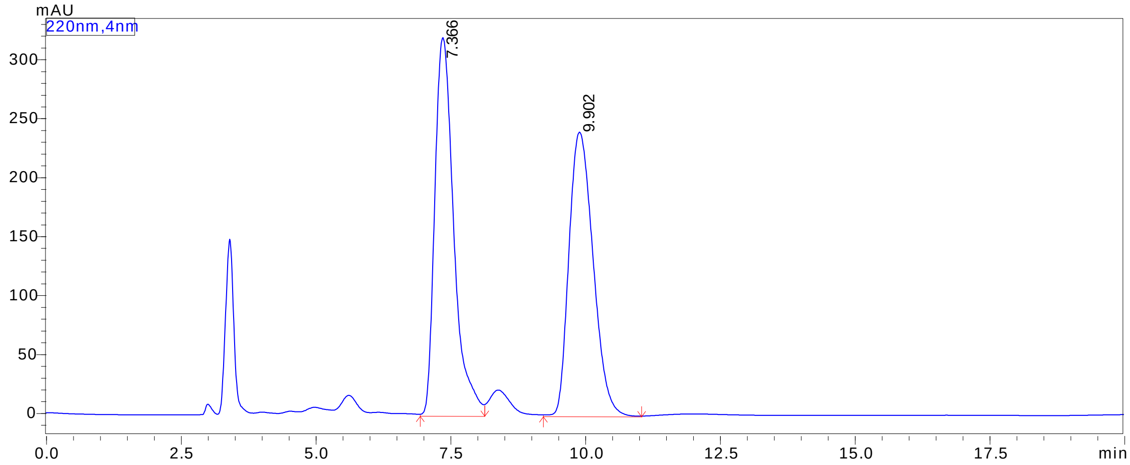

Peak#	Ret. Time	Area%
1	7.366	51.181
2	9.902	48.819
Total		100.000

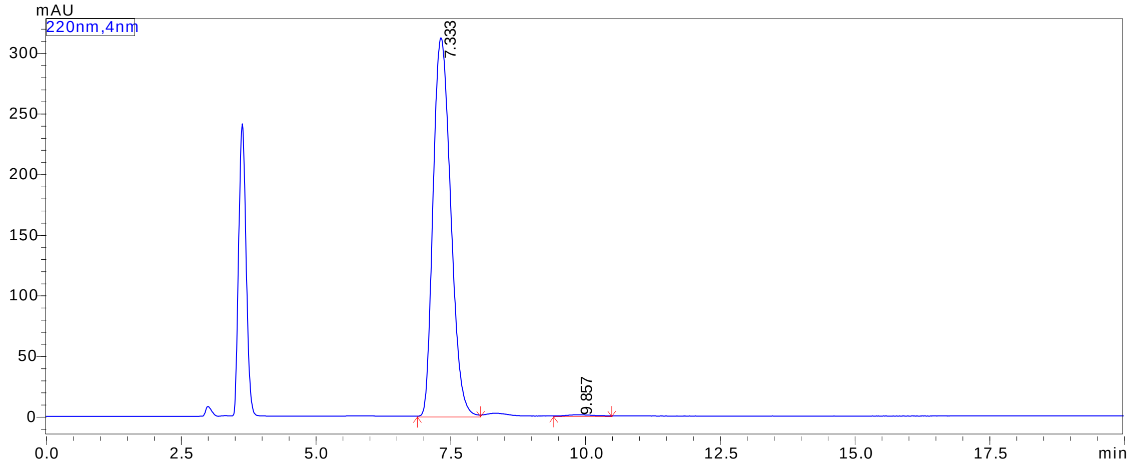

Peak#	Ret. Time	Area%
1	7.333	99.616
2	9.857	0.384
Total		100.000

HPLC trace of *rac*-**12** and (*S*)-**12**

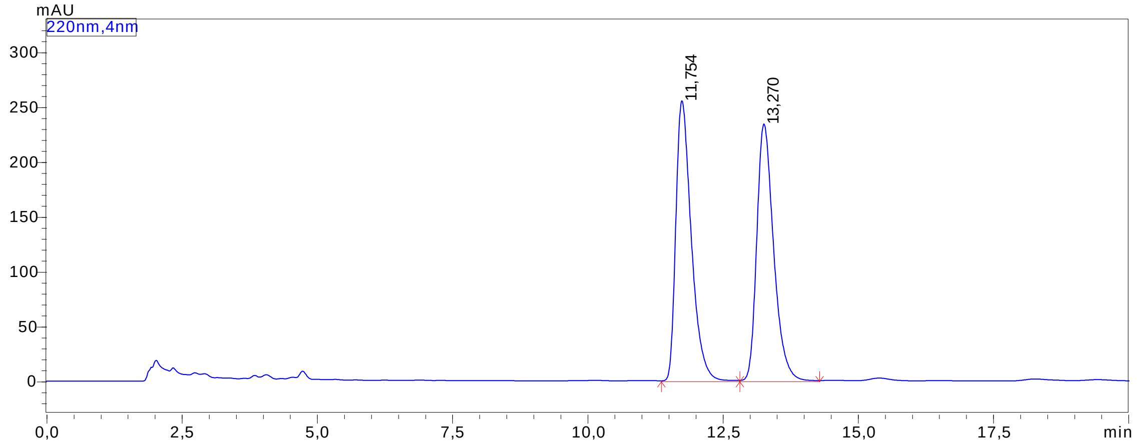

Peak#	Ret. Time	Area%
1	11,754	50,039
2	13,270	49,961
Total		100,000

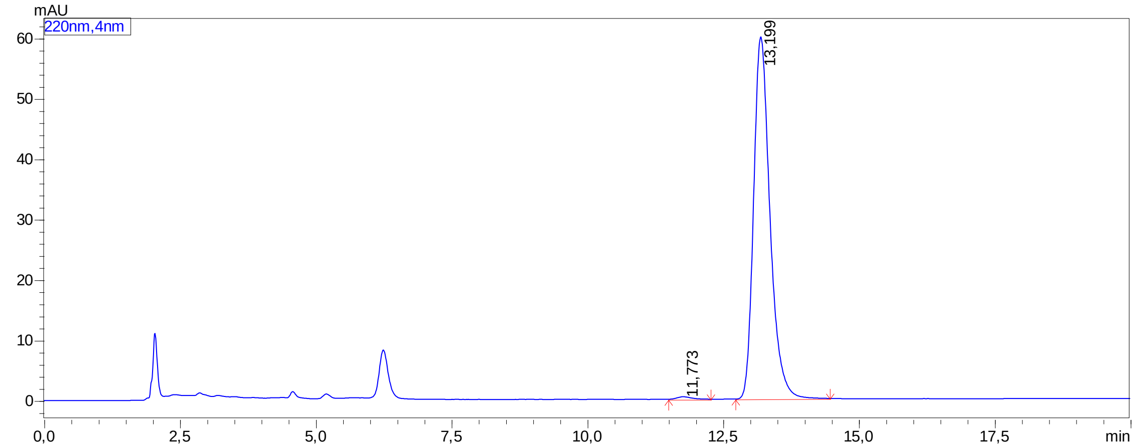

Peak#	Ret. Time	Area%
1	11,773	0,606
2	13,199	99,394
Total		100,000

HPLC trace of *rac*-**13** and (*S*)-**13**

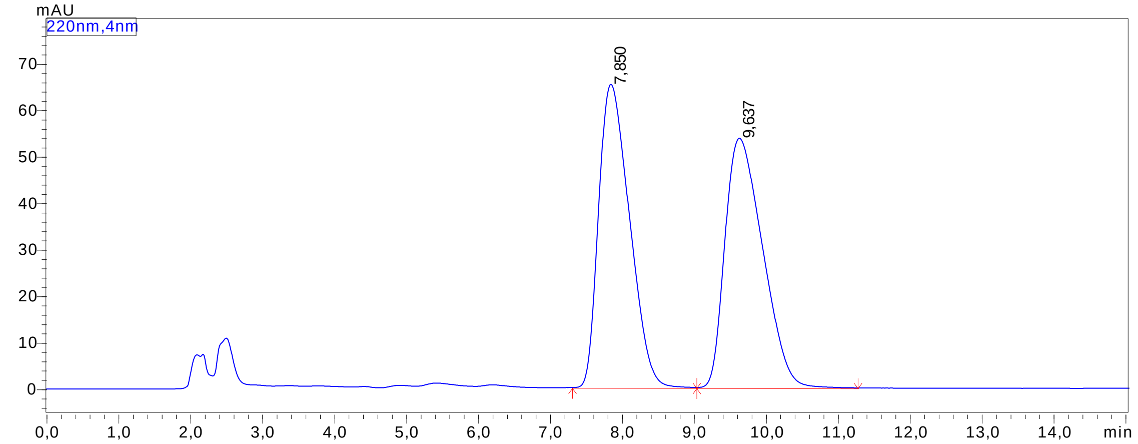

Peak#	Ret. Time	Area%
1	7,850	49,959
2	9,637	50,041
Total		100,000

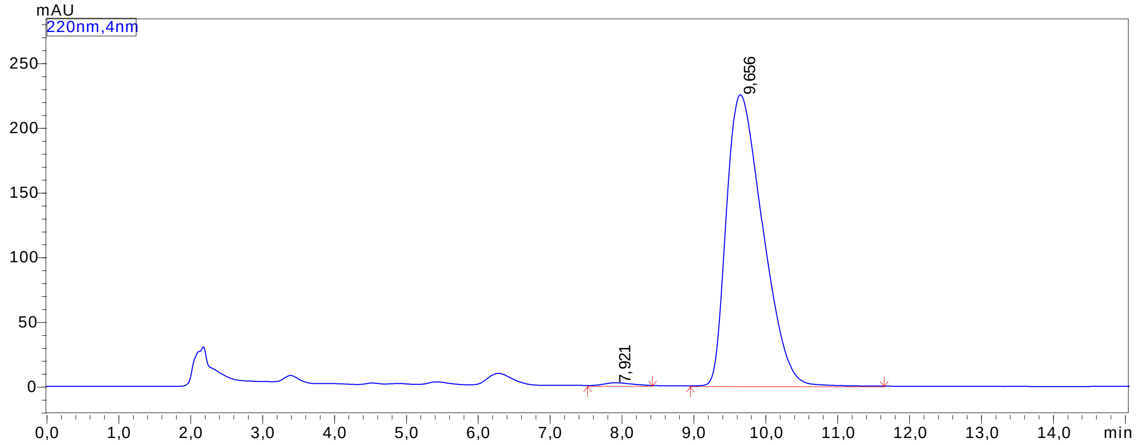

Peak#	Ret. Time	Area%
1	7,921	0,481
2	9,656	99,519
Total		100,000

HPLC trace of *rac*-**14** and (*S*)-**14**

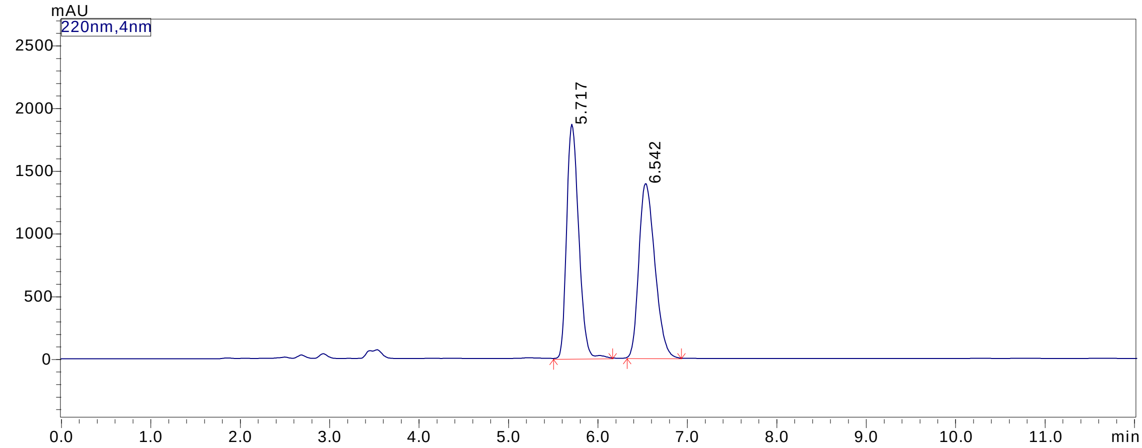

Peak#	Ret. Time	Area%
1	5.717	50.286
2	6.542	49.714
Total		100.000

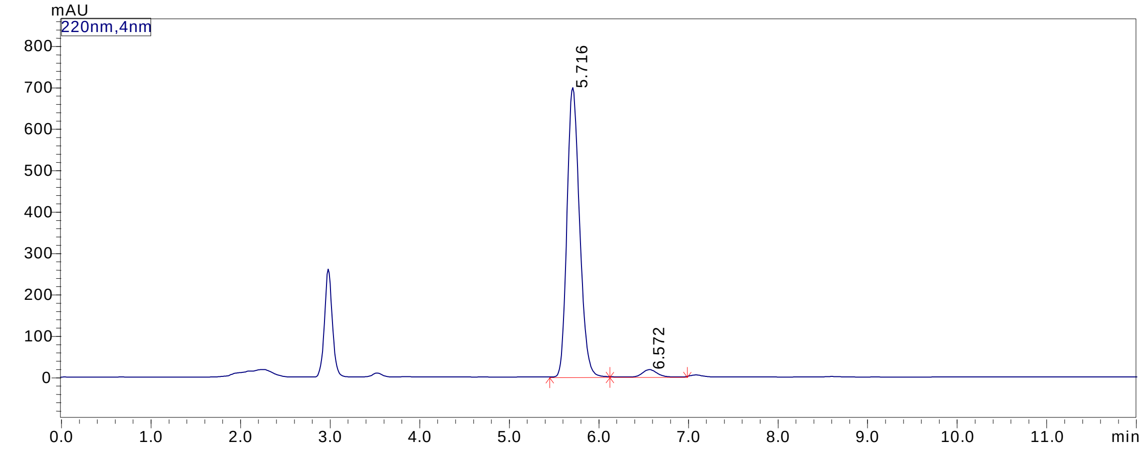

Peak#	Ret. Time	Area%
1	5.716	97.796
2	6.572	2.204
Total		100.000

HPLC trace of *rac*-**15** and (*S*)-**15** after Bz protection

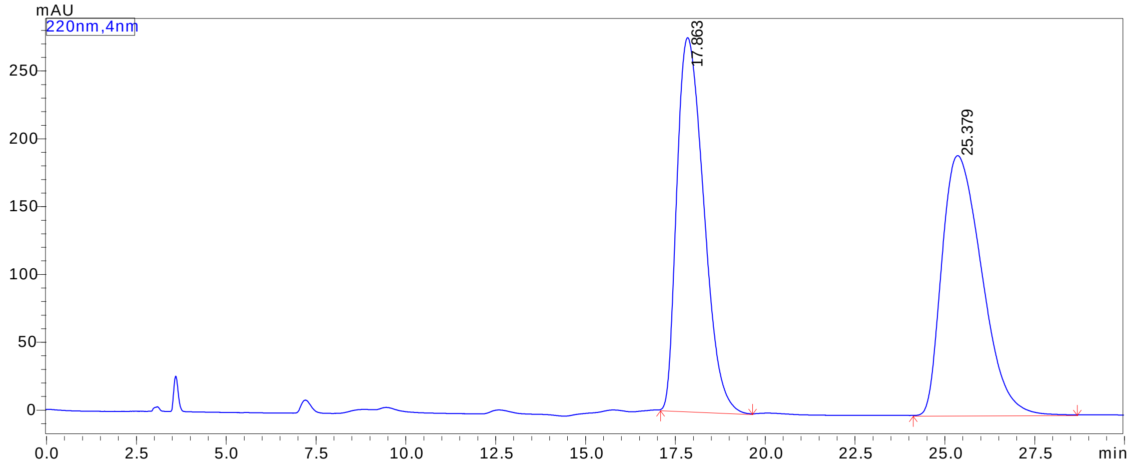

Peak#	Ret. Time	Area%
1	17.863	49.262
2	25.379	50.738
Total		100.000

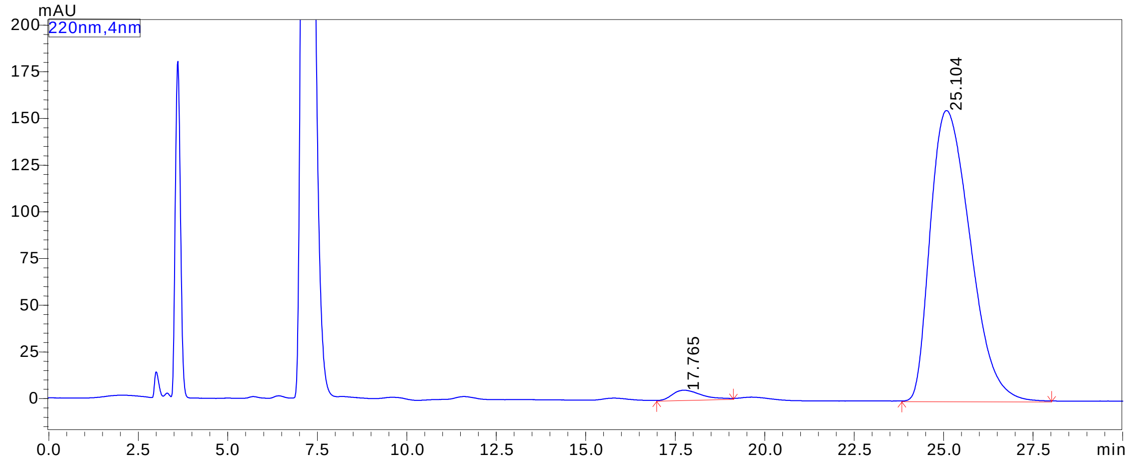

Peak#	Ret. Time	Area%
1	17.765	1.746
2	25.104	98.254
Total		100.000

HPLC trace of *rac*-**15** and (*R*)-**15** after Bz protection

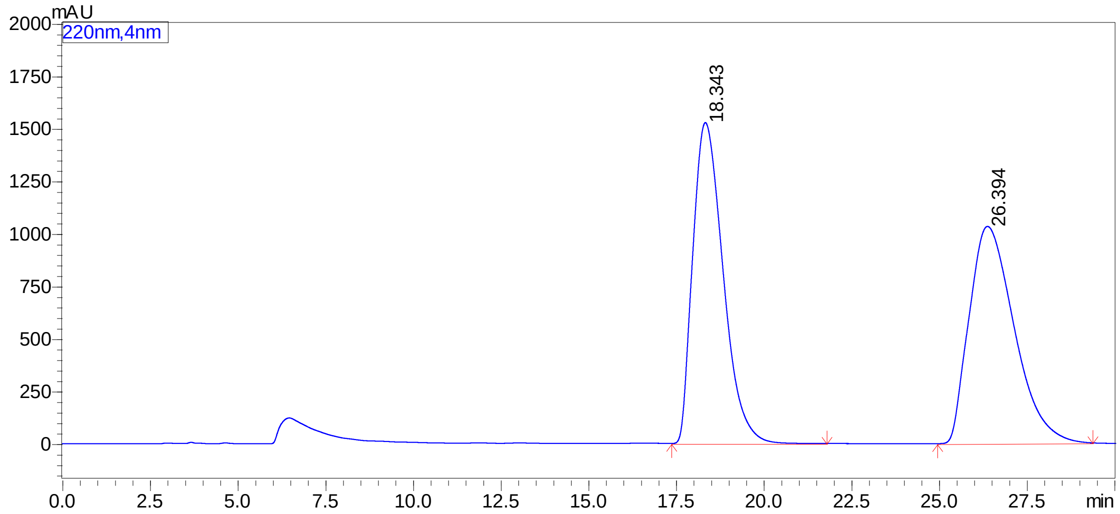

Peak#	Ret. Time	Area%
1	18.343	49.782
2	26.394	50.218
Total		100.000

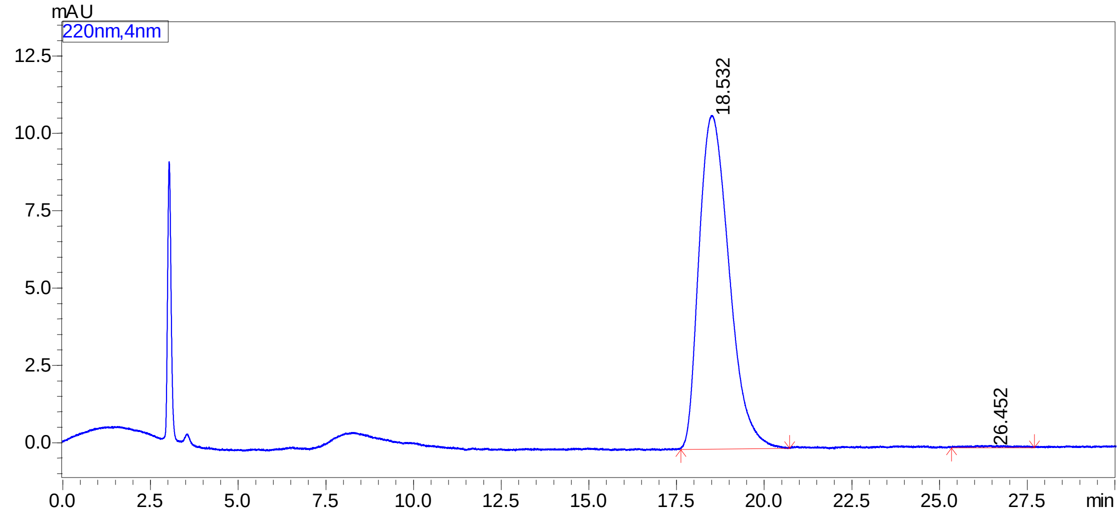

Peak#	Ret. Time	Area%
1	18.532	99.626
2	26.452	0.374
Total		100.000

11. ^{1}H , ^{13}C, ^{31}P and ^{19}F NMR spectra of substrates and products

^{1}H and ^{13}C NMR traces of **1b**

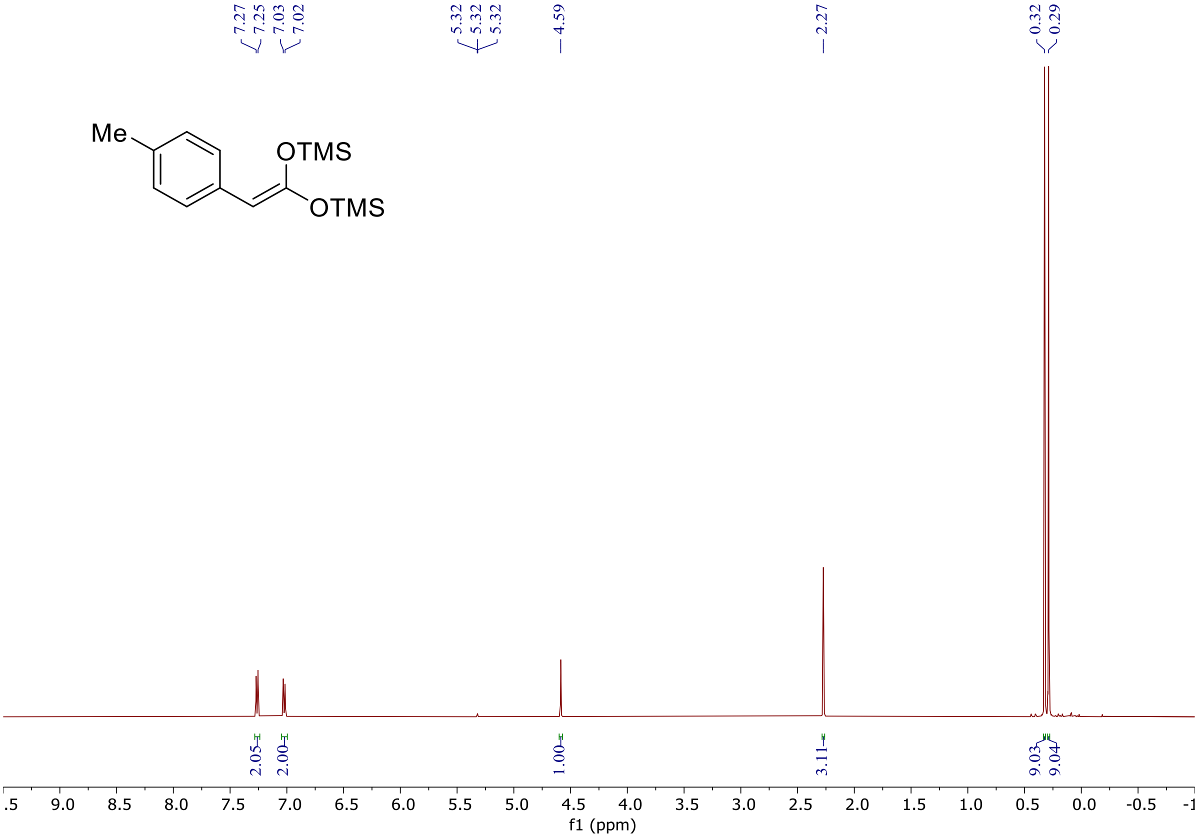

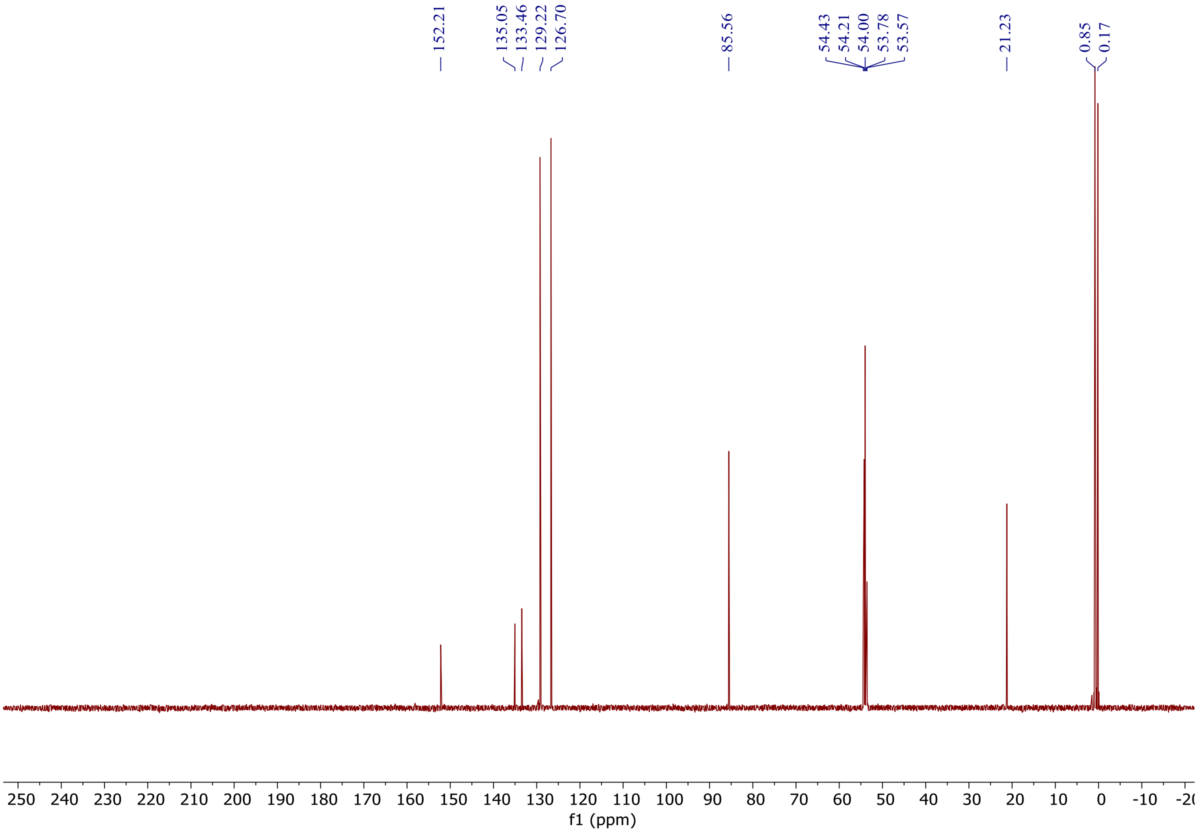

^{1}H and ^{13}C NMR traces of **1c**

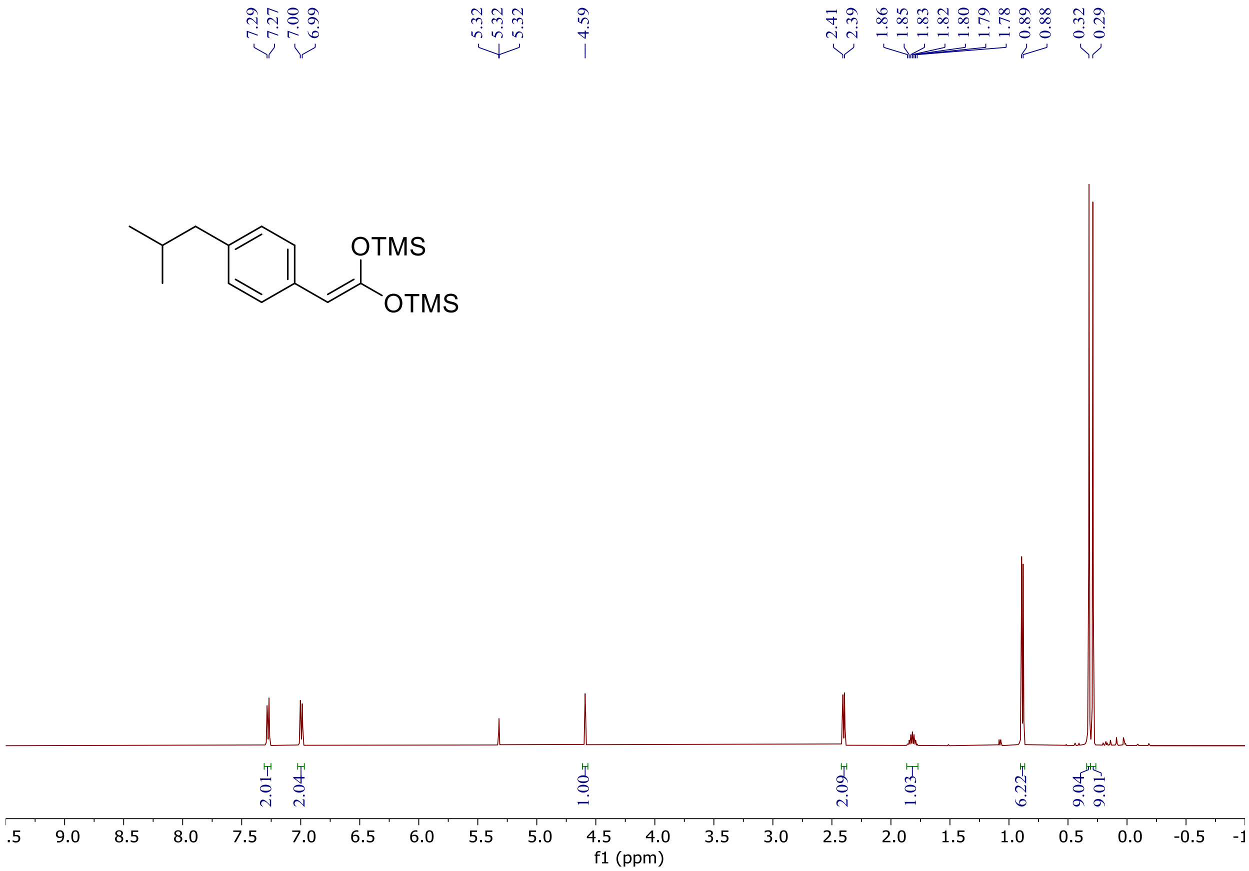

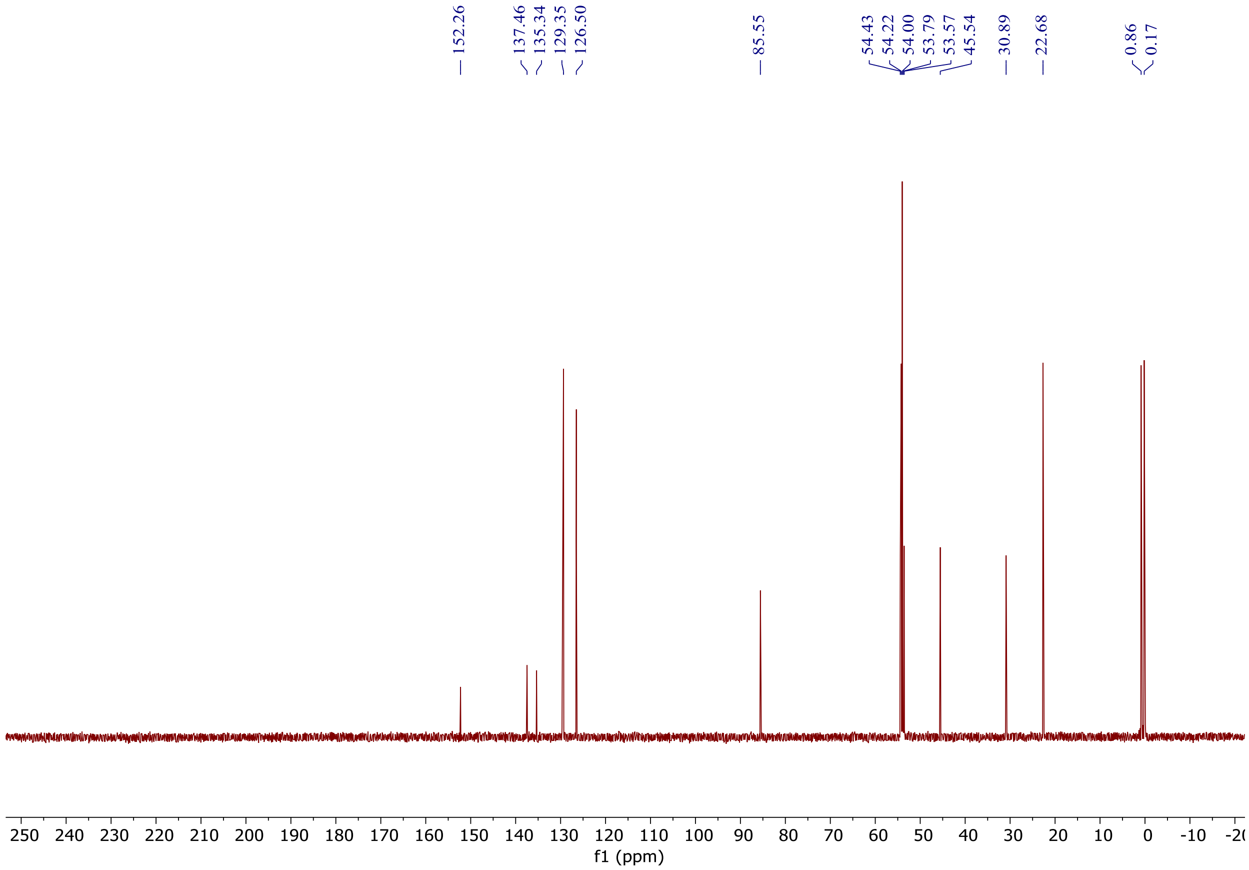

^{1}H and ^{13}C NMR traces of **1d**

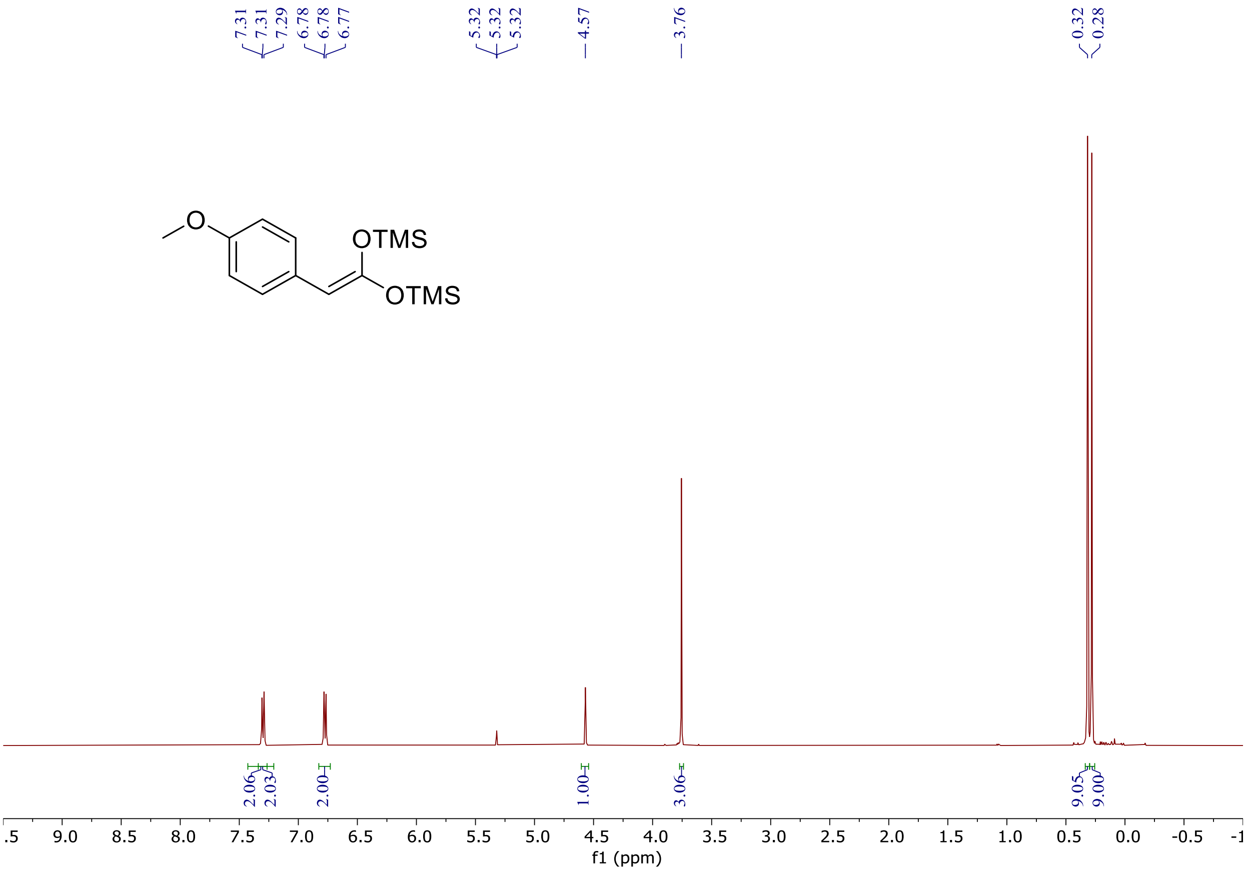

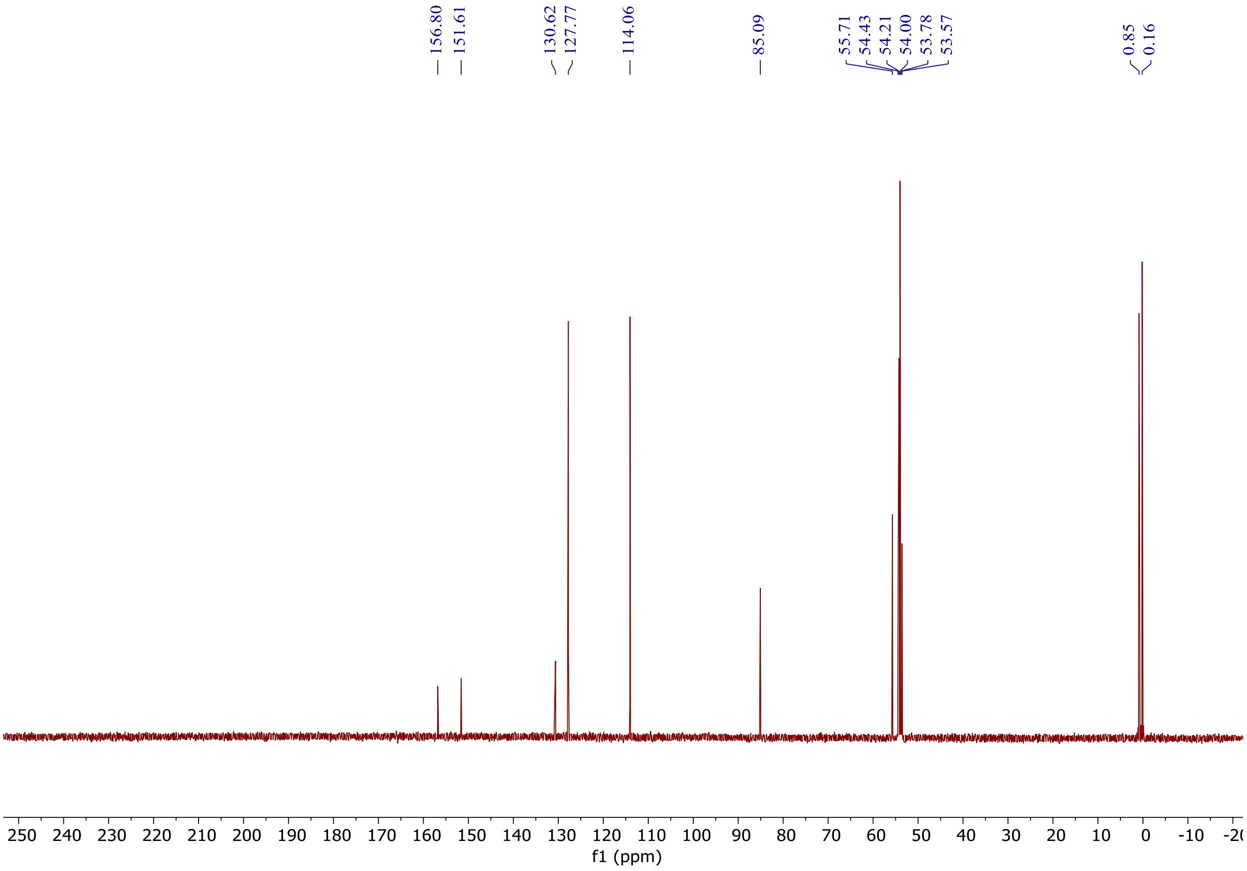

^{1}H, ^{13}C and ^{19}F NMR traces of **1f**

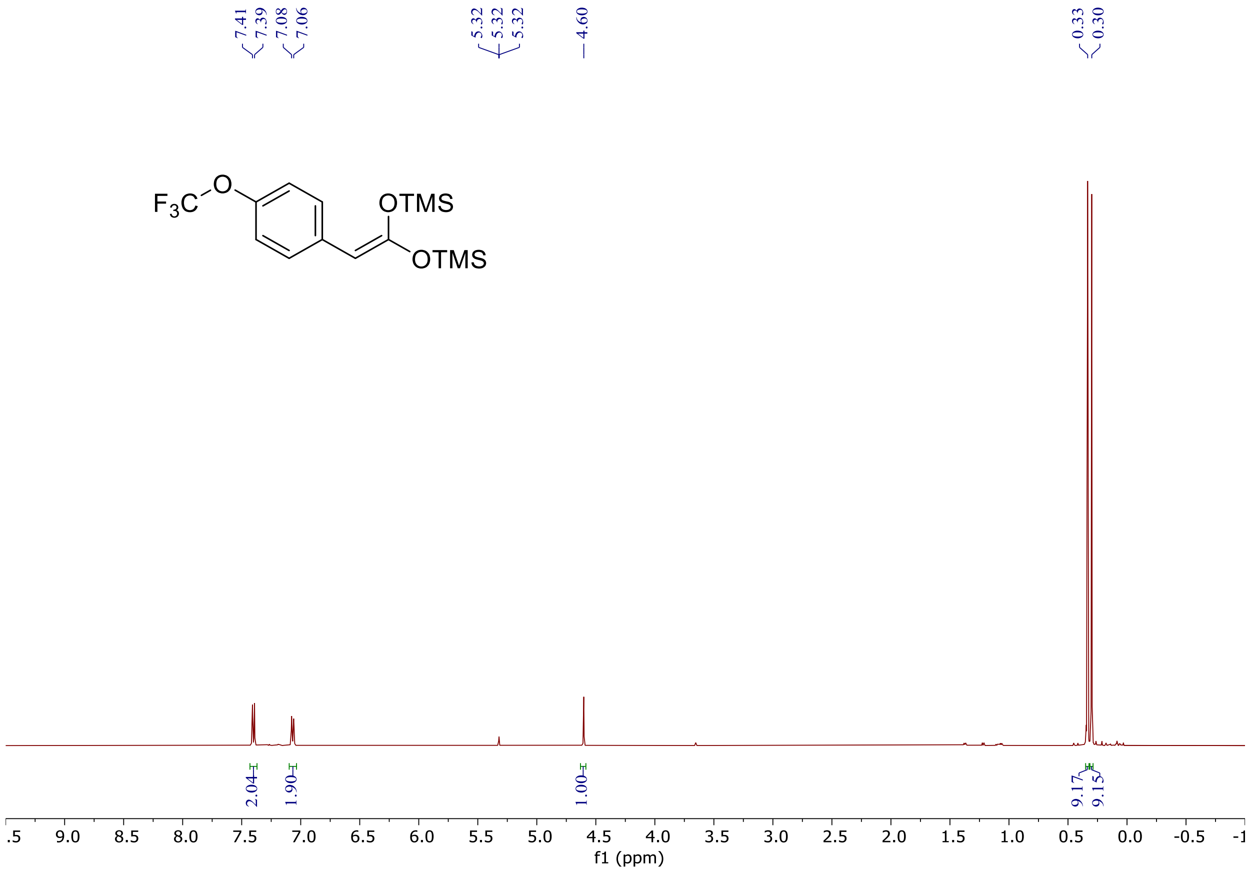

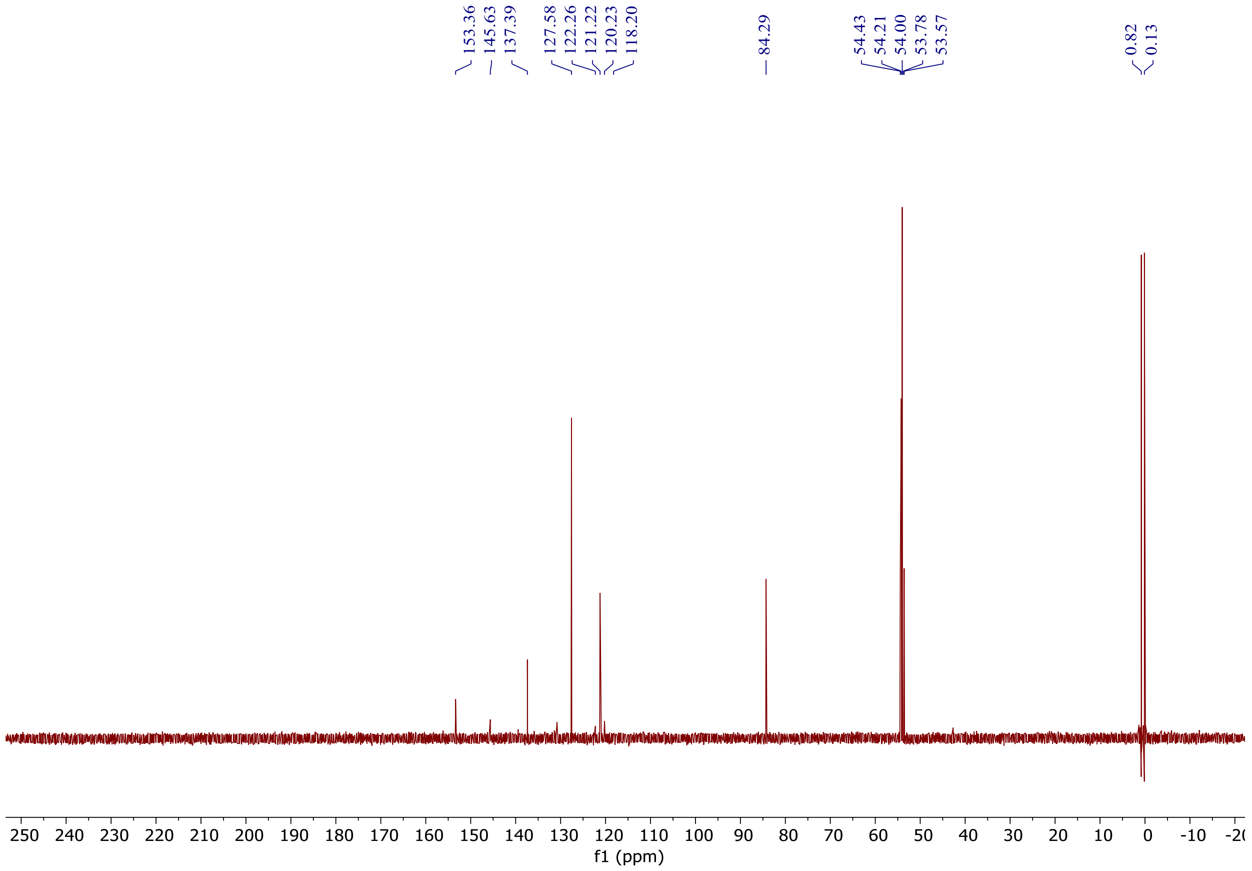

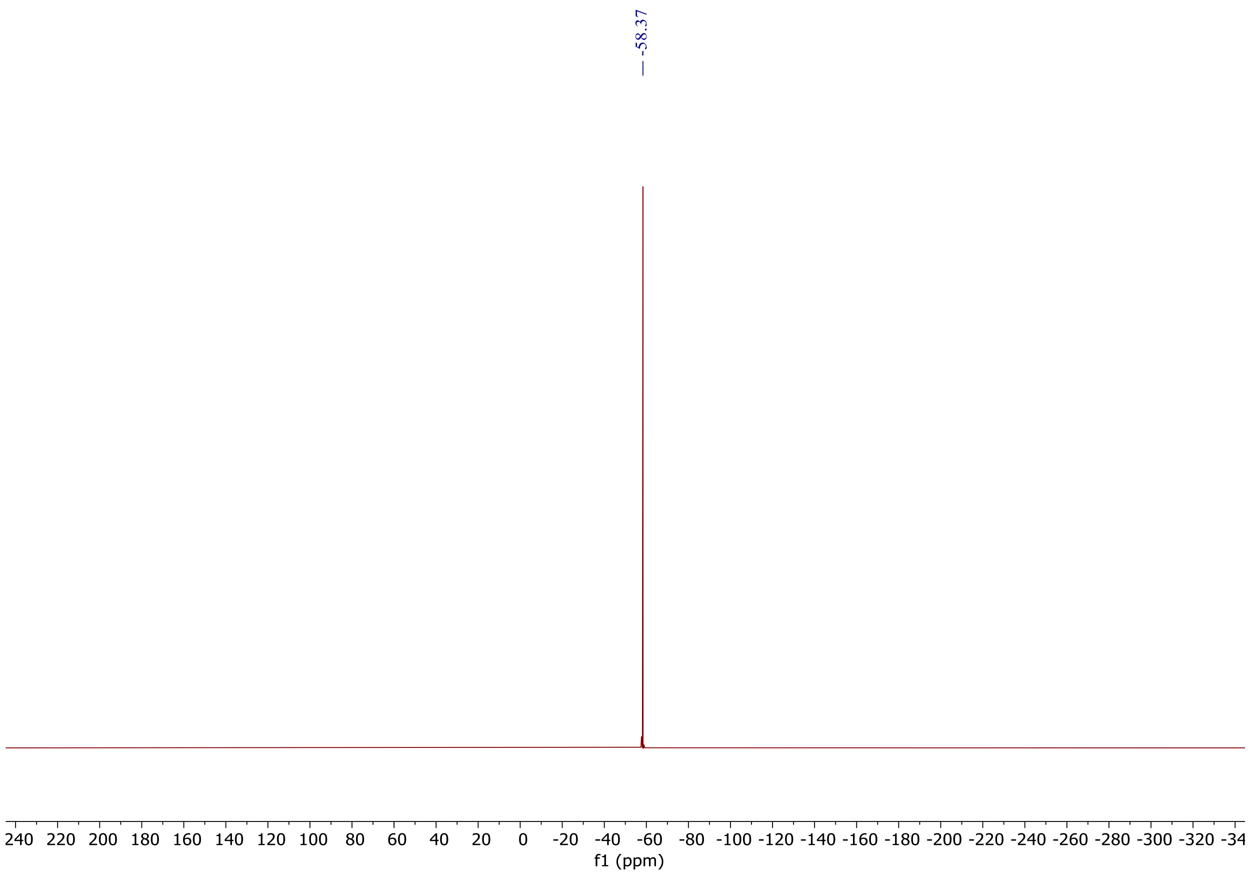
-58.37
240 220 200 180 160 140 120 100 80 60 40 20 0 -20 -40 -60 -80 -100 -120 -140 -160 -180 -200 -220 -240 -260 -280 -300 -320 -34
f1 (ppm)

^{1}H and ^{13}C NMR traces of **1i**

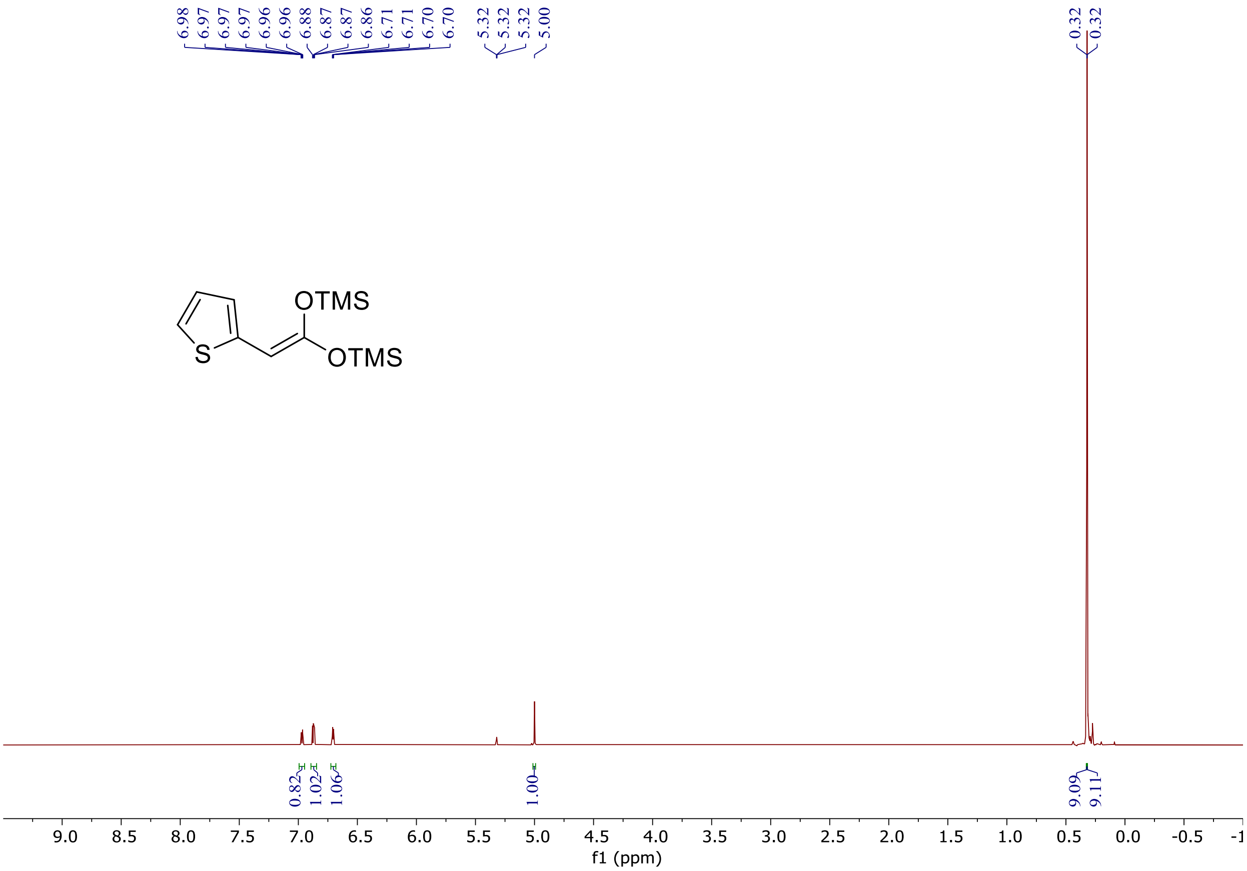

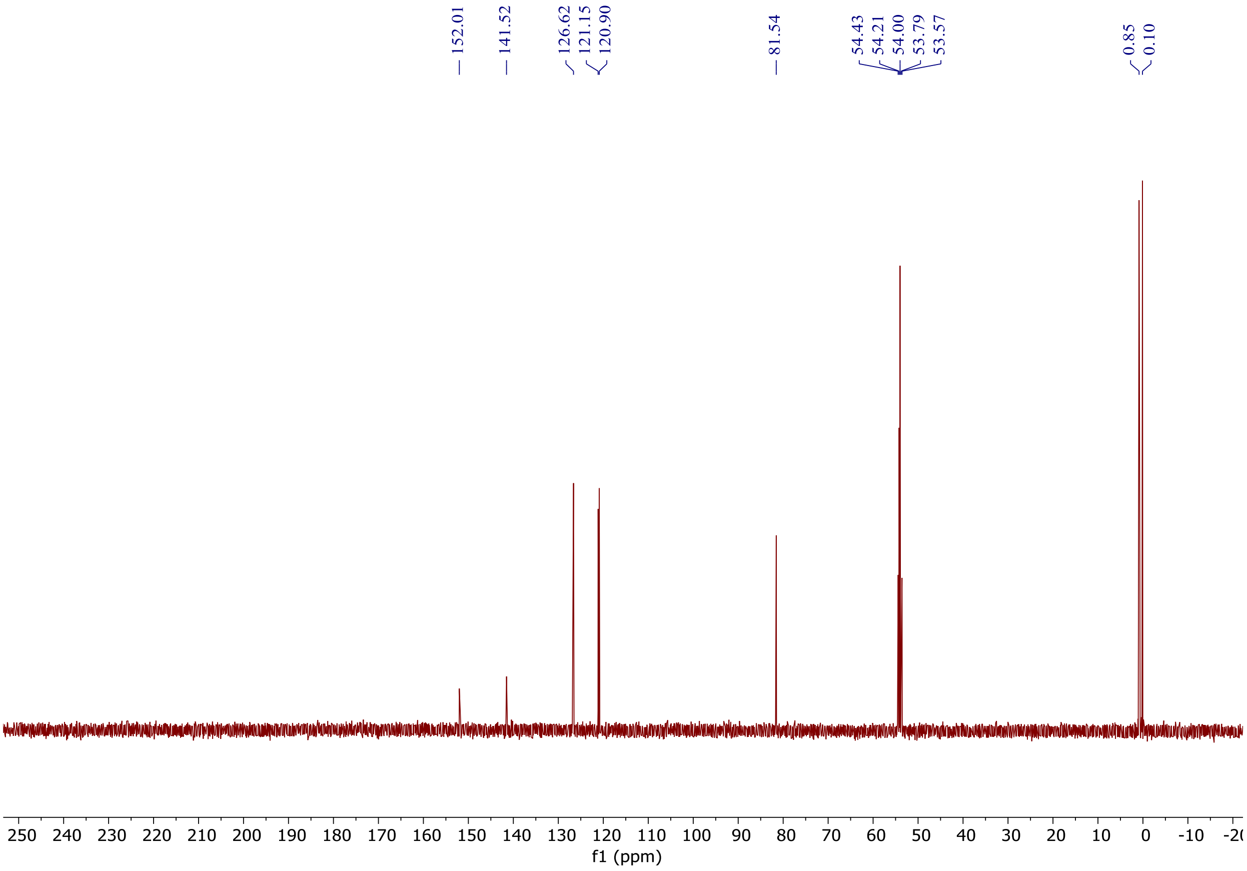

^{1}H and ^{13}C NMR traces of **1j**

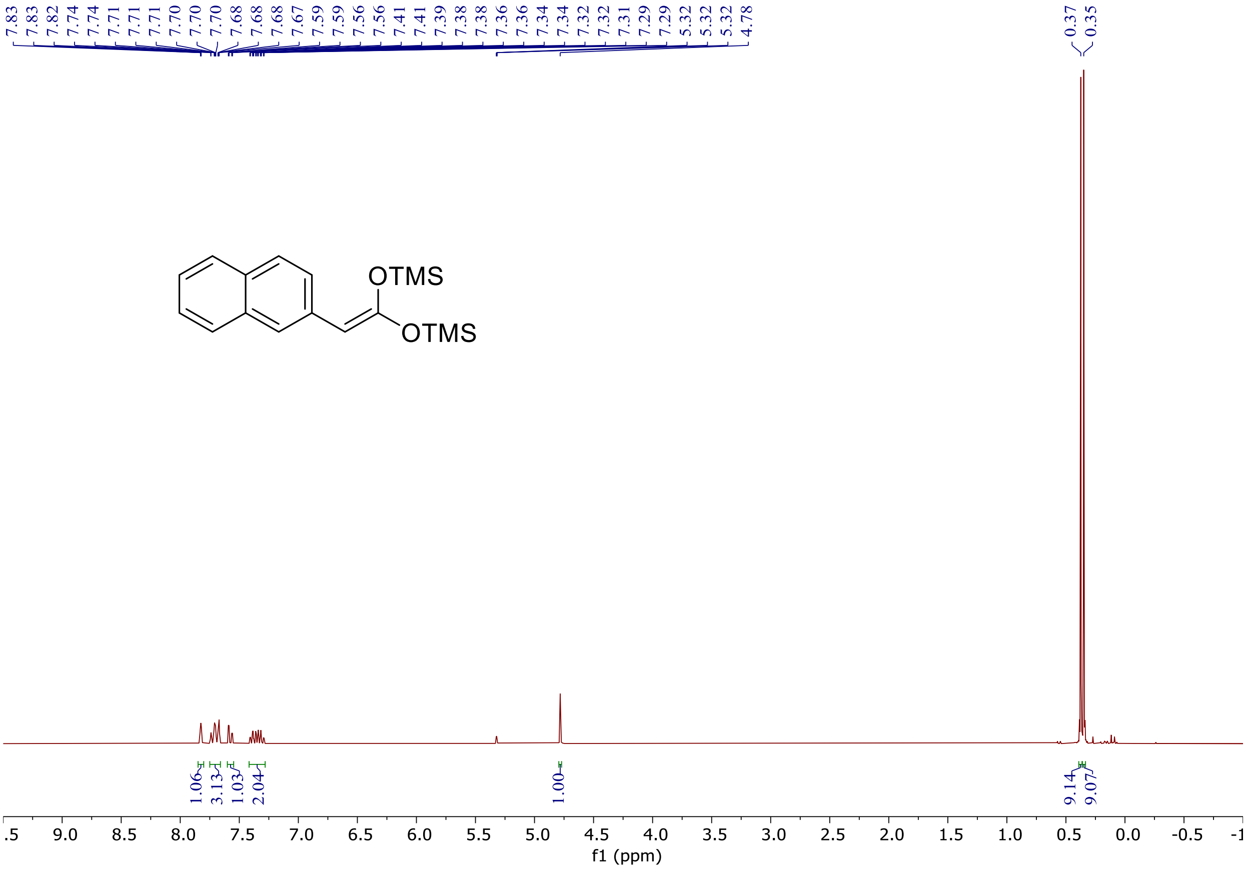

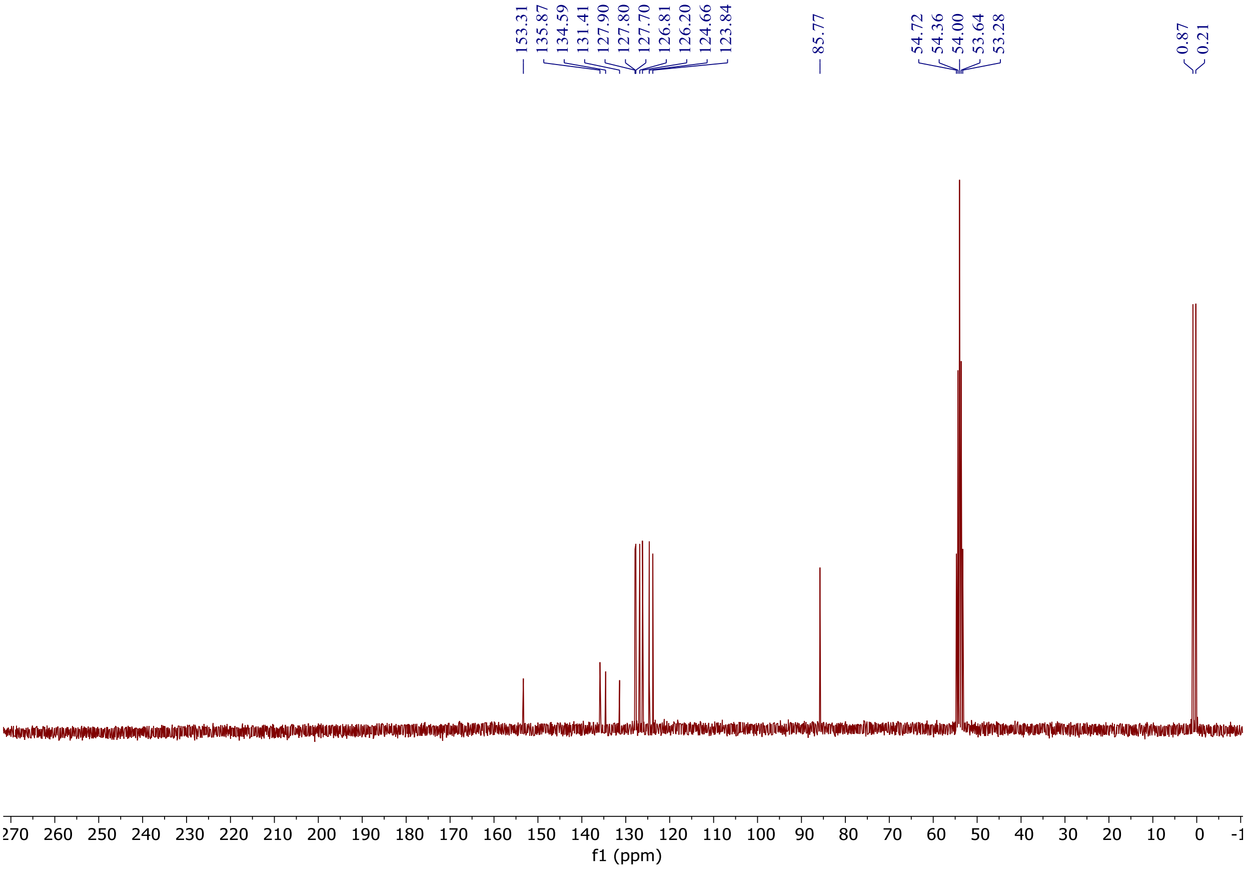

^{1}H and ^{13}C NMR traces of **1n**

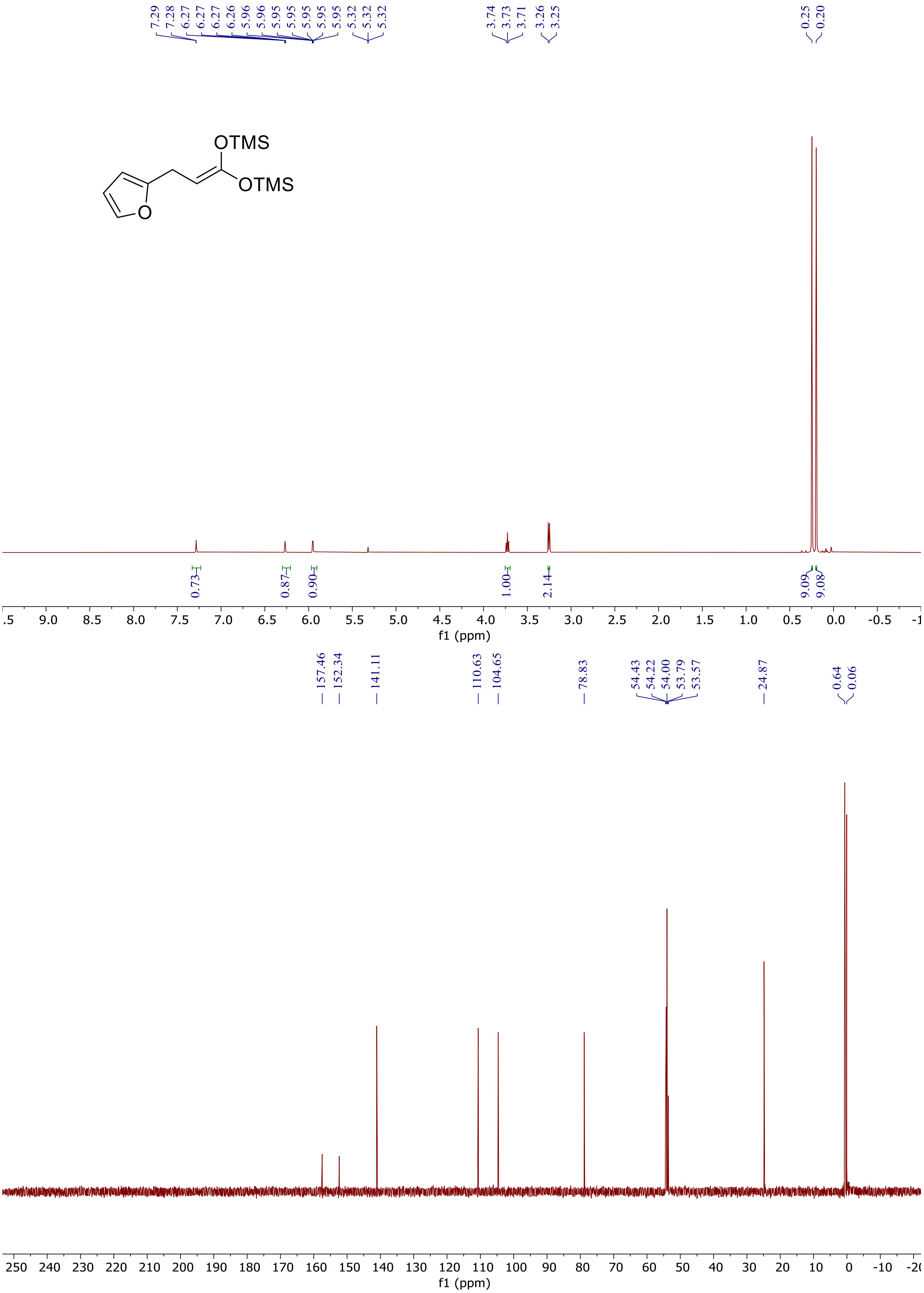

^{1}H and ^{13}C NMR traces of **1y**

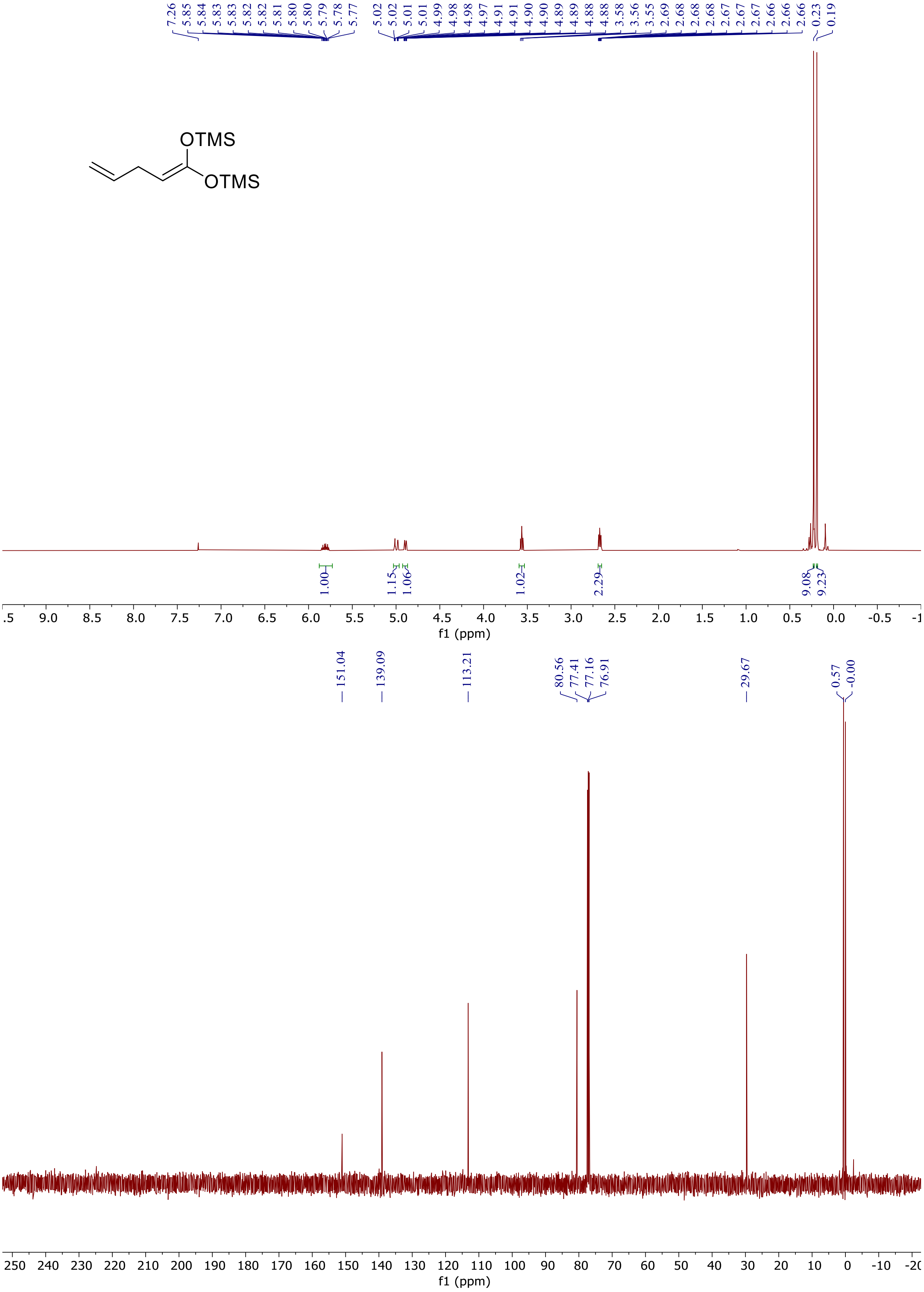

^1H, ^{13}C and ^{19}F NMR traces of **s1b**

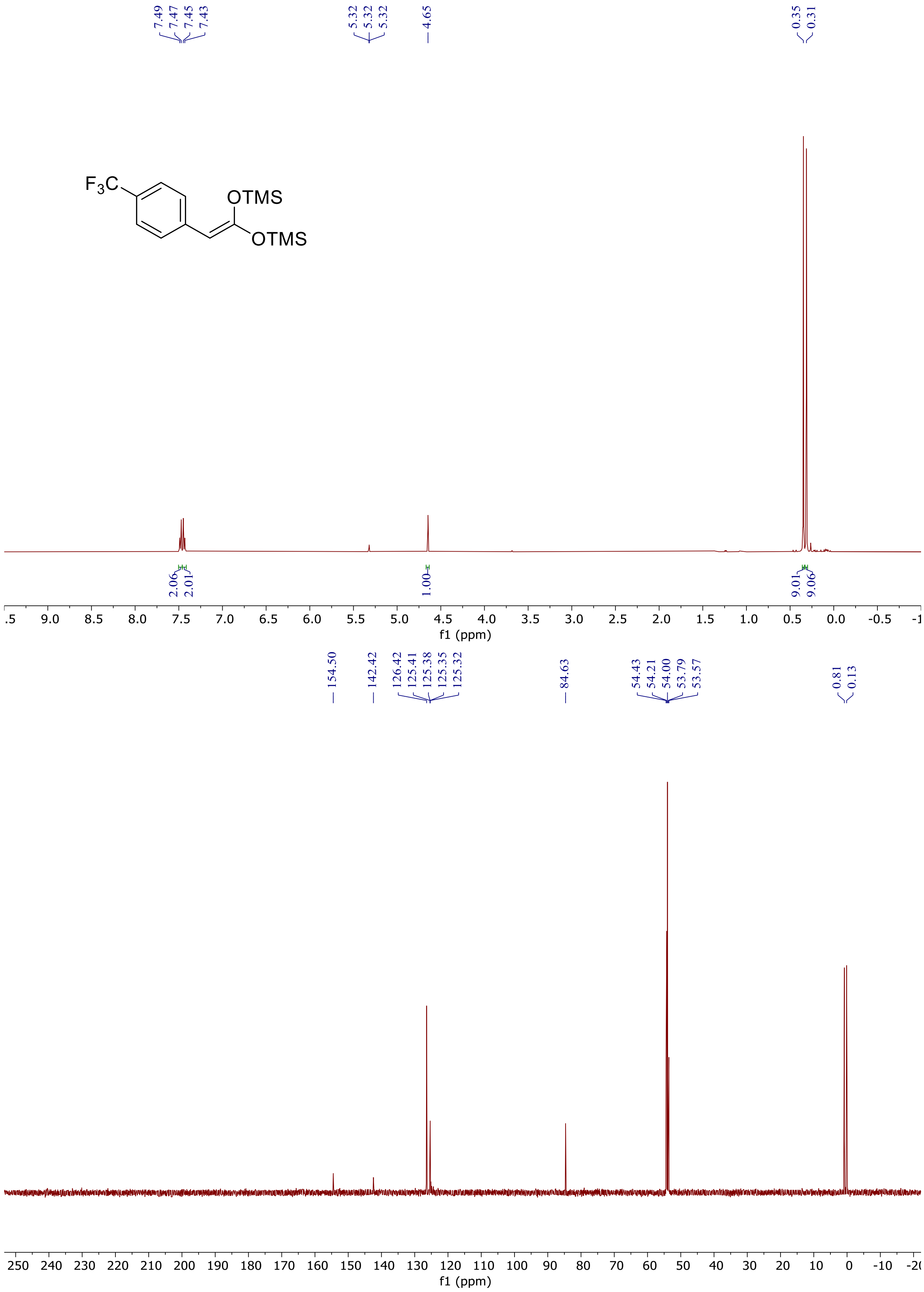

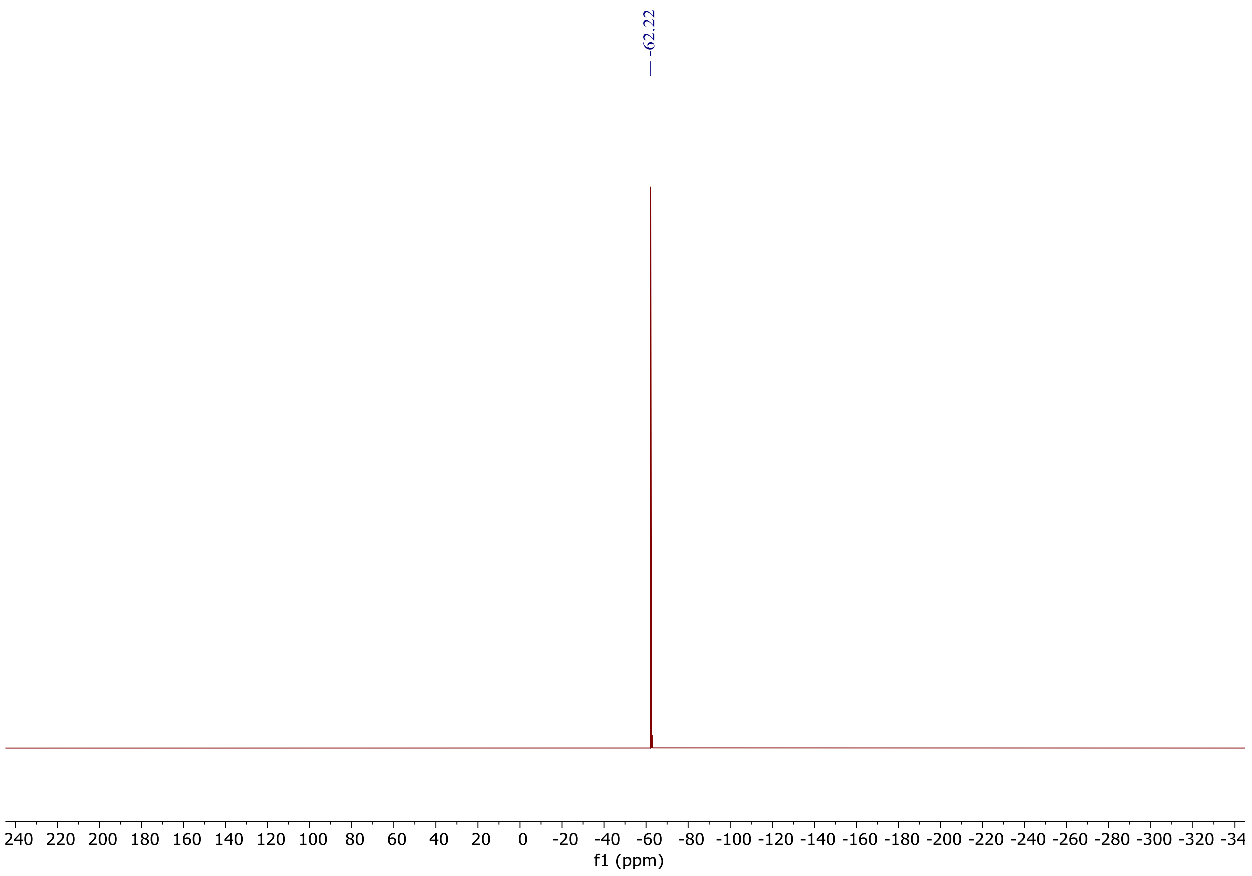
-62.22
240 220 200 180 160 140 120 100 80 60 40 20 0 -20 -40 -60 -80 -100 -120 -140 -160 -180 -200 -220 -240 -260 -280 -300 -320 -34
f1 (ppm)

^{1}H and ^{13}C NMR traces of **diol**

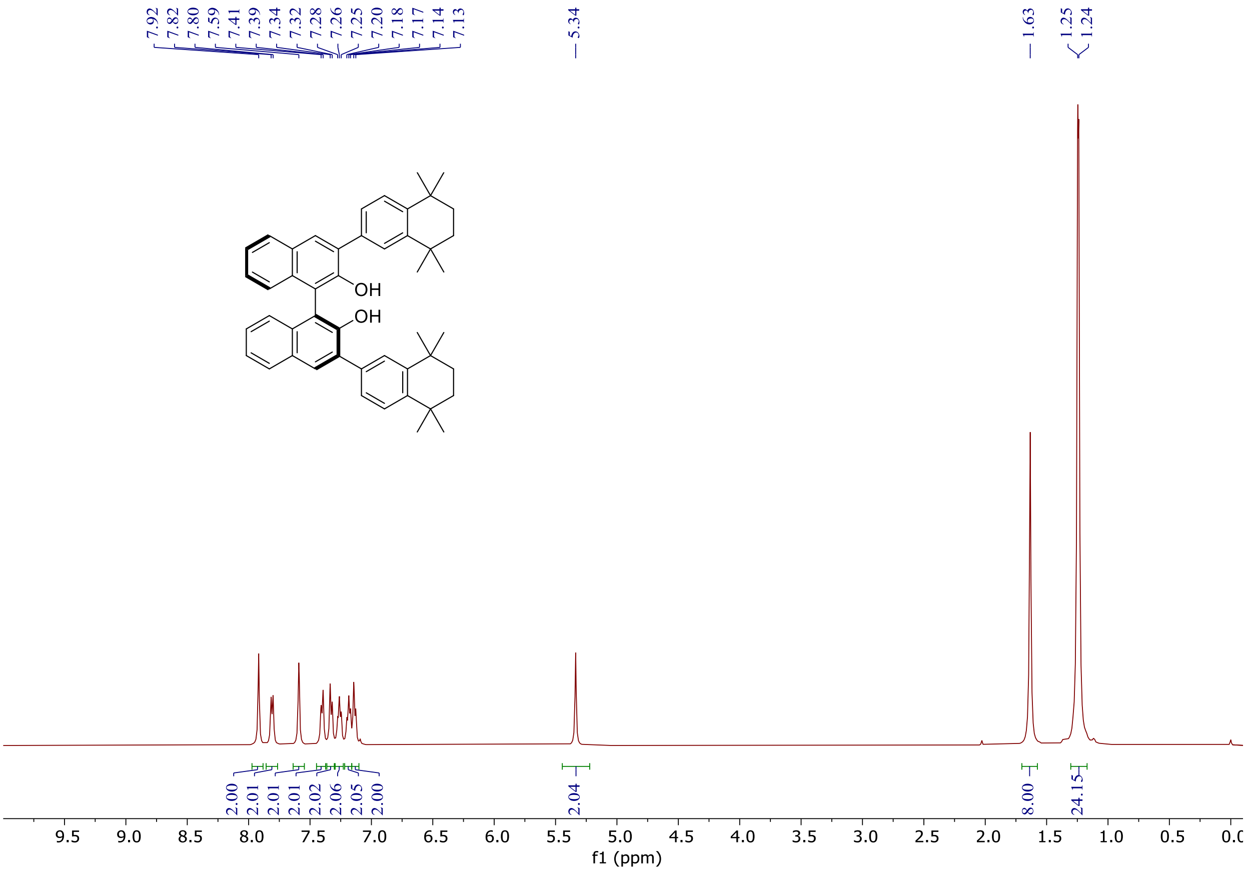

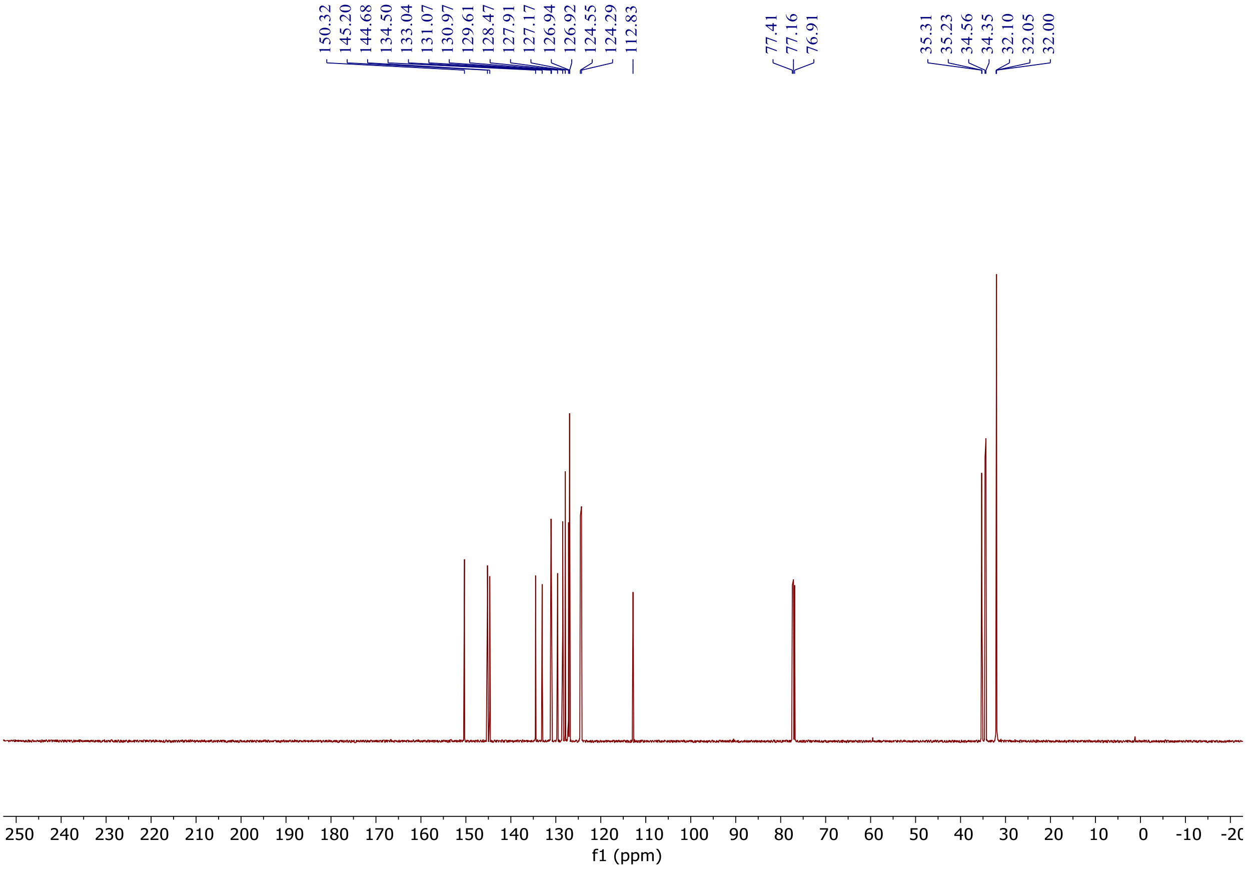

^{1}H and ^{13}C NMR traces of **IDPi 3e**

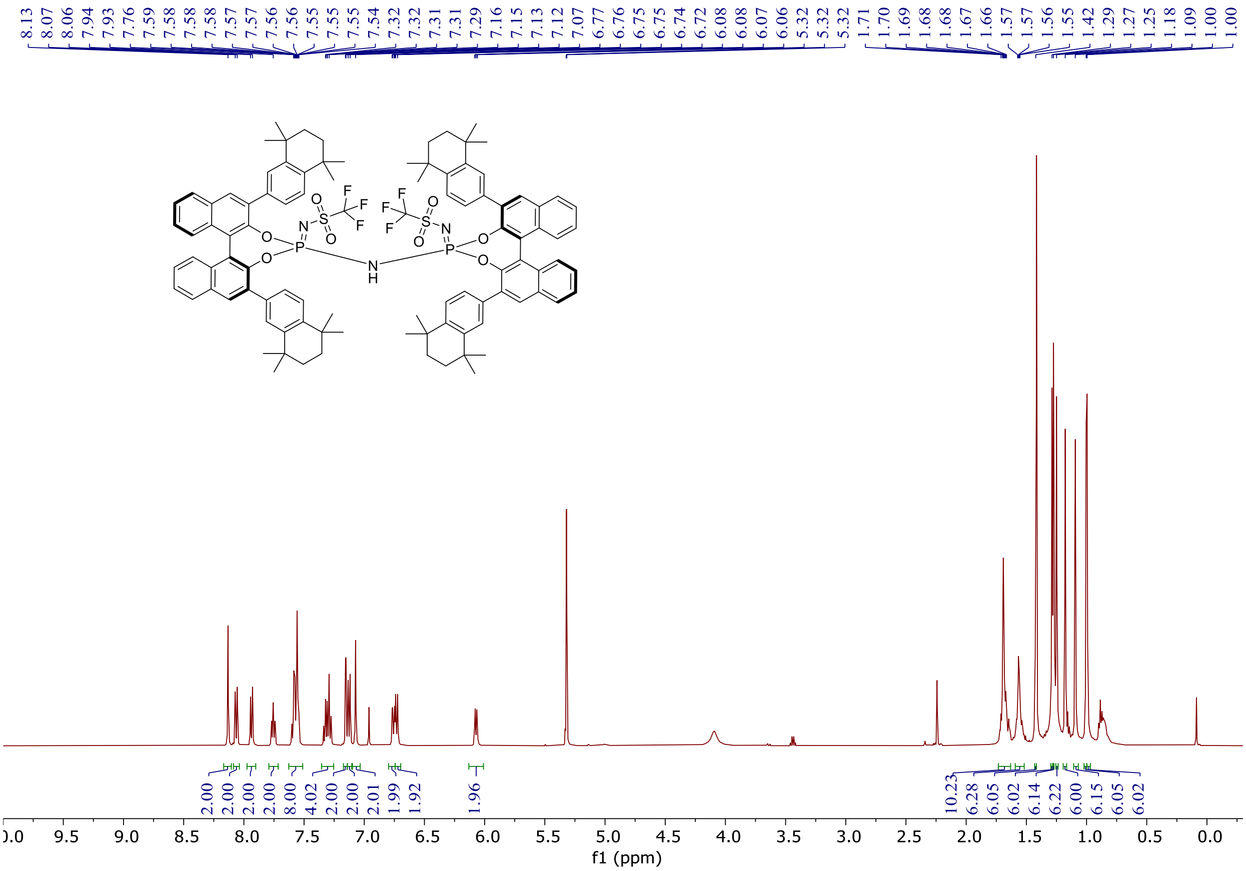

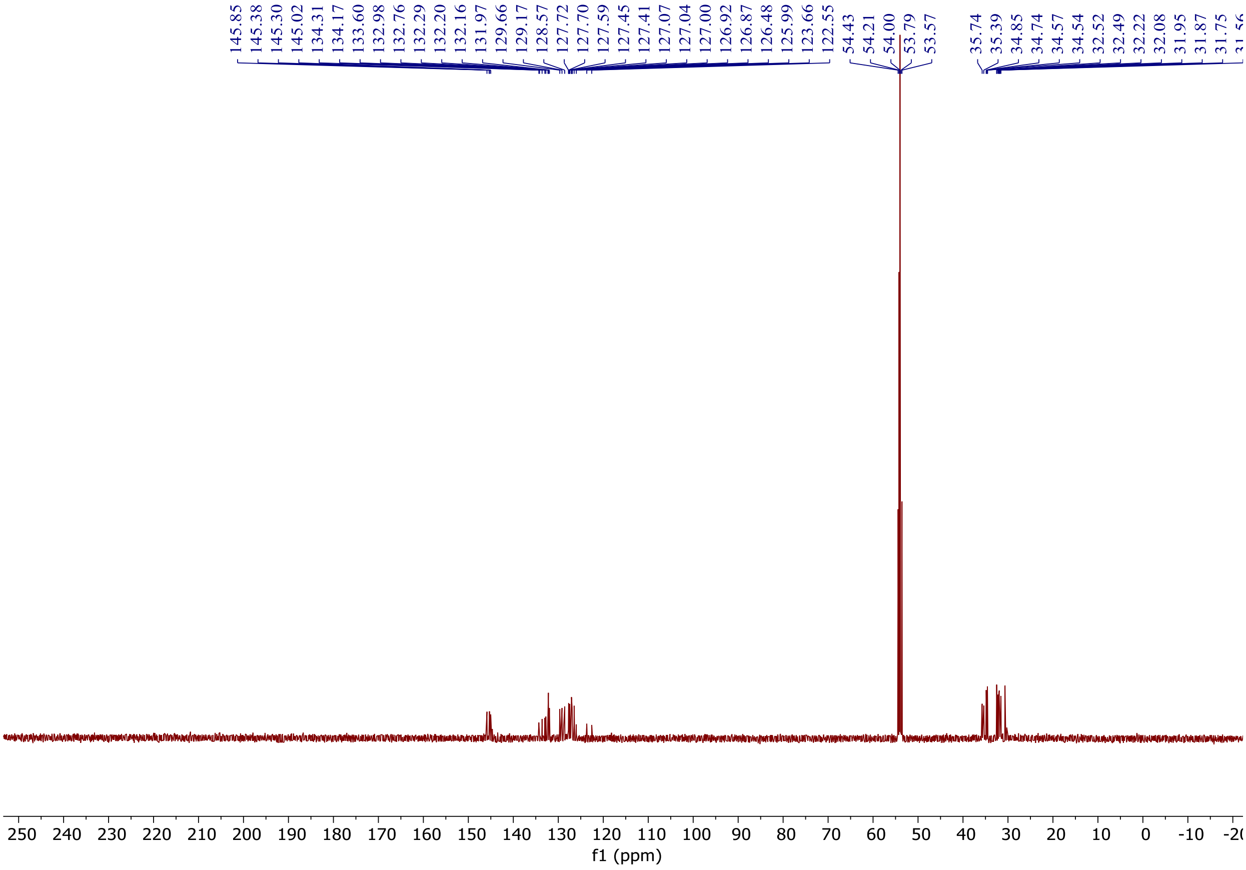

^{19}F and ^{31}P NMR traces of **IDPi 3e**

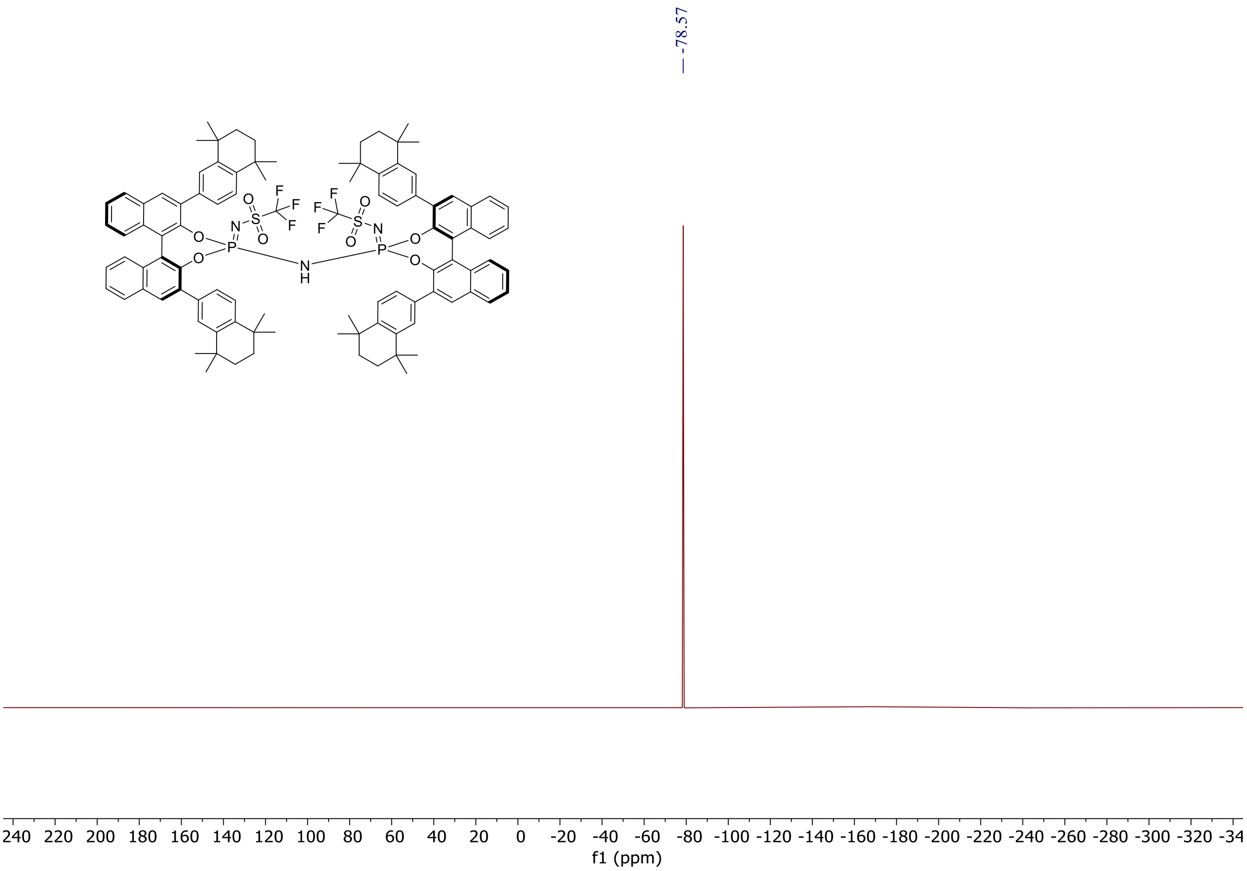

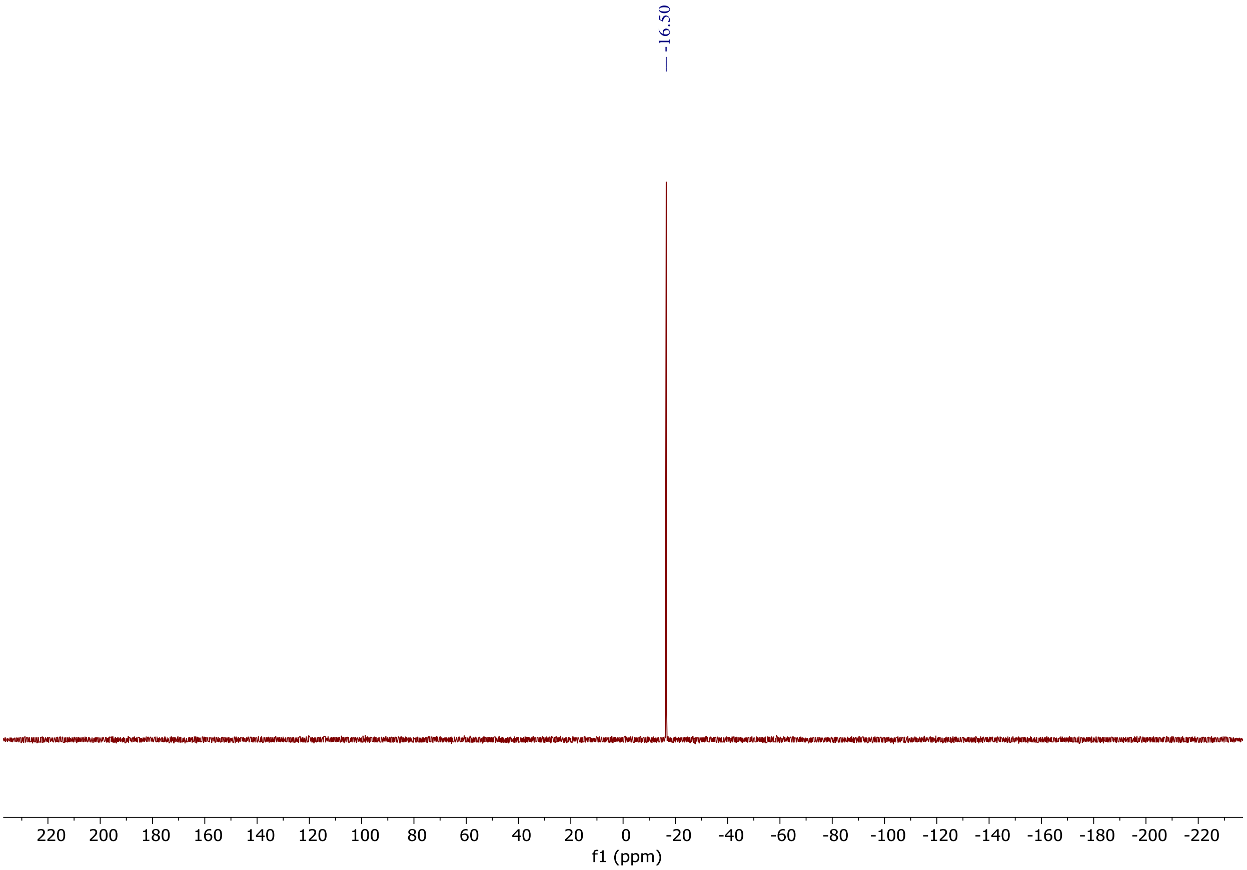

^{1}H and ^{13}C NMR traces of ***i*IDP**

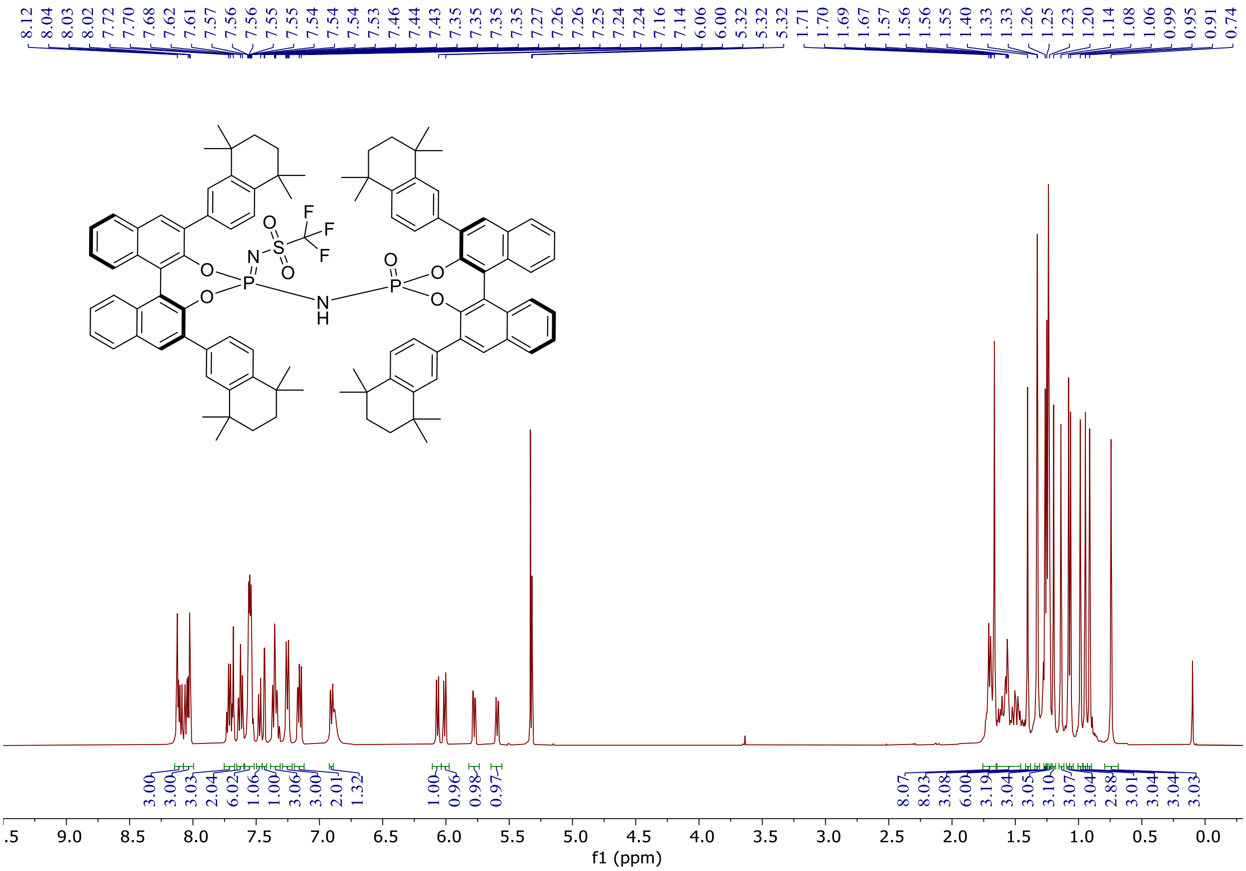

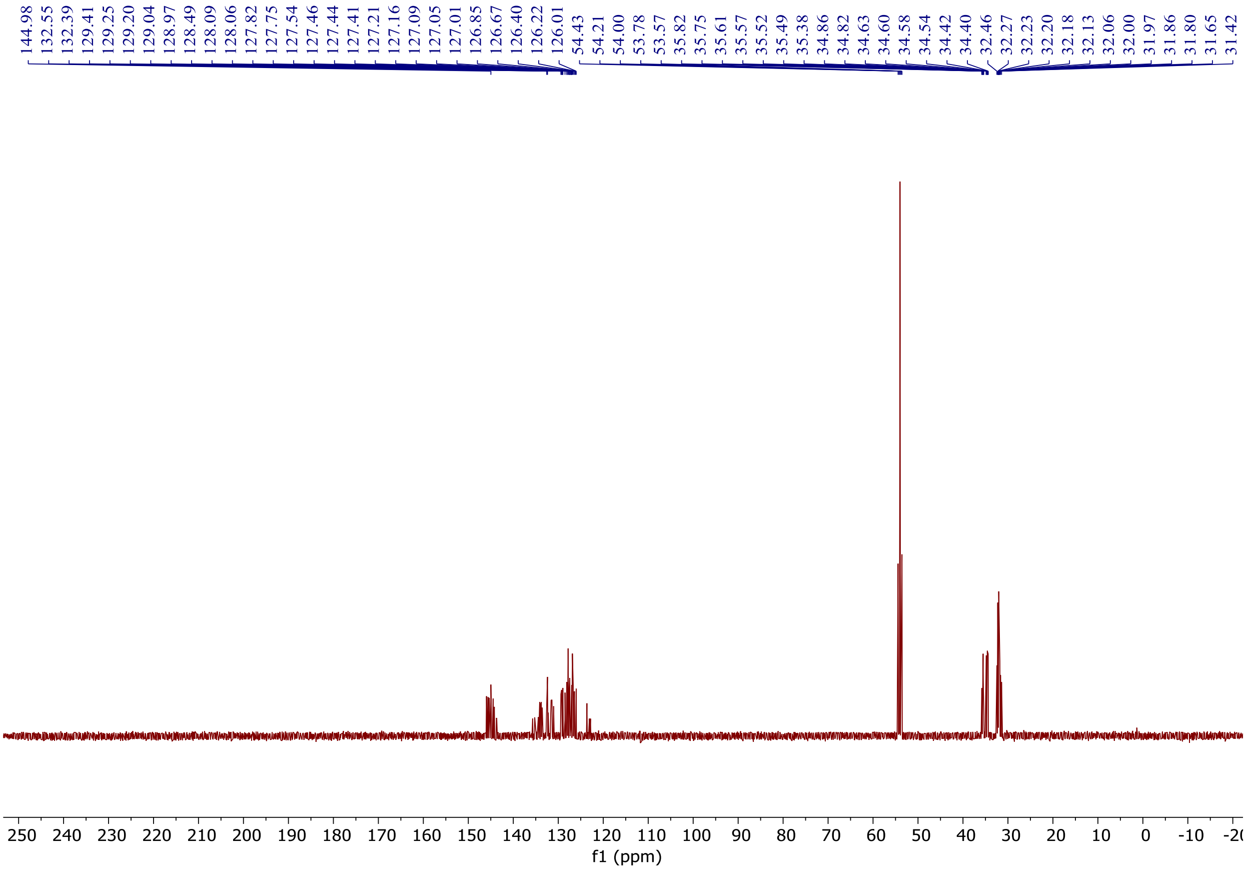

^{19}F and ^{31}P NMR traces of ***i*IDP**

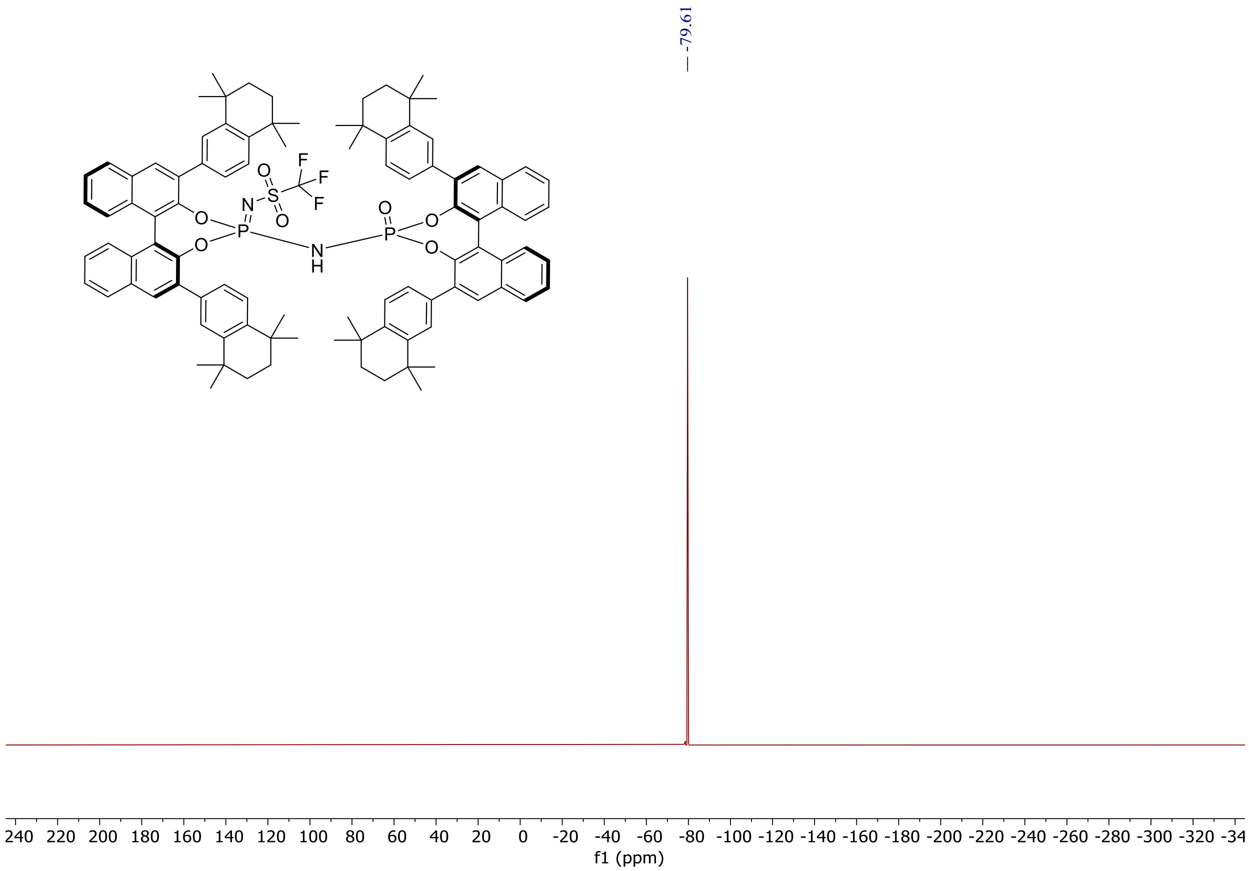

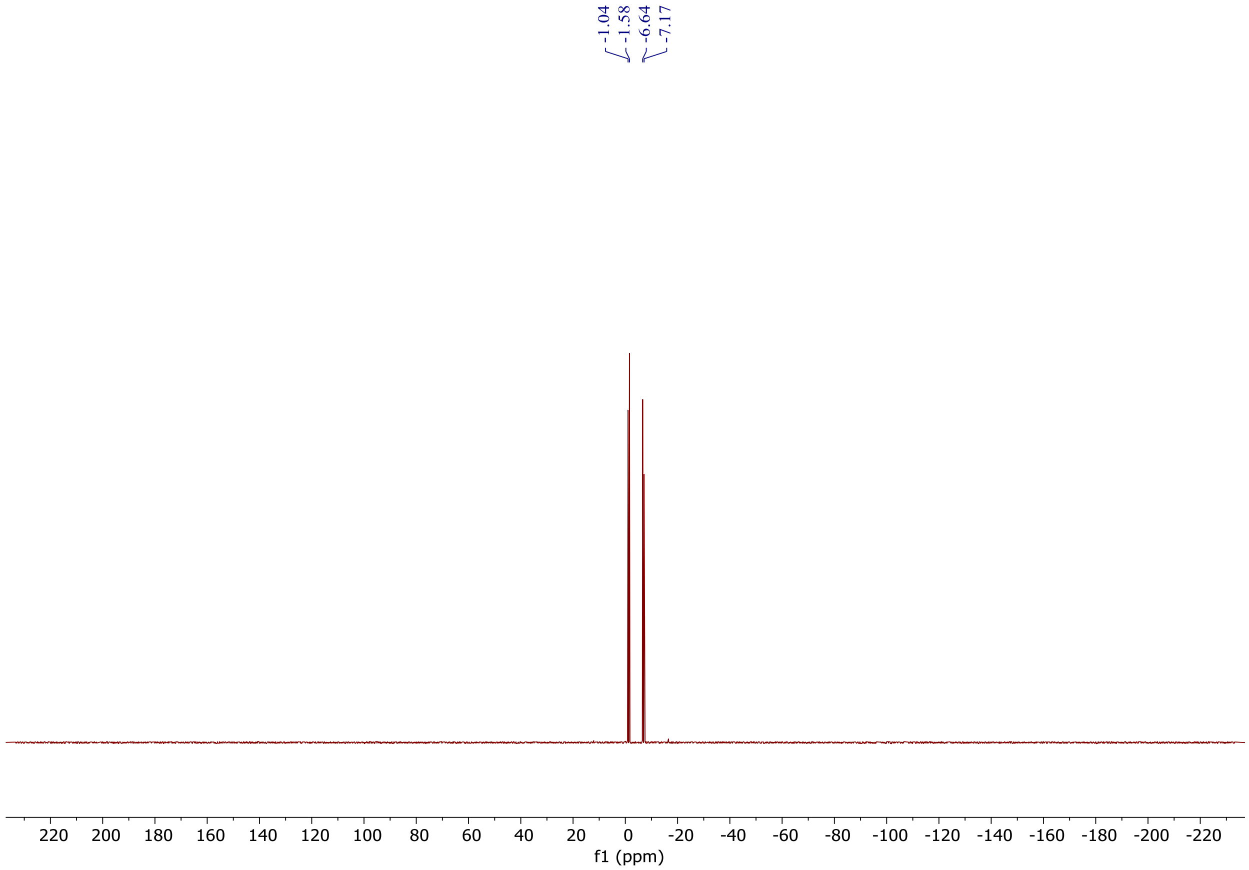

^{1}H and ^{13}C NMR traces of **4a**

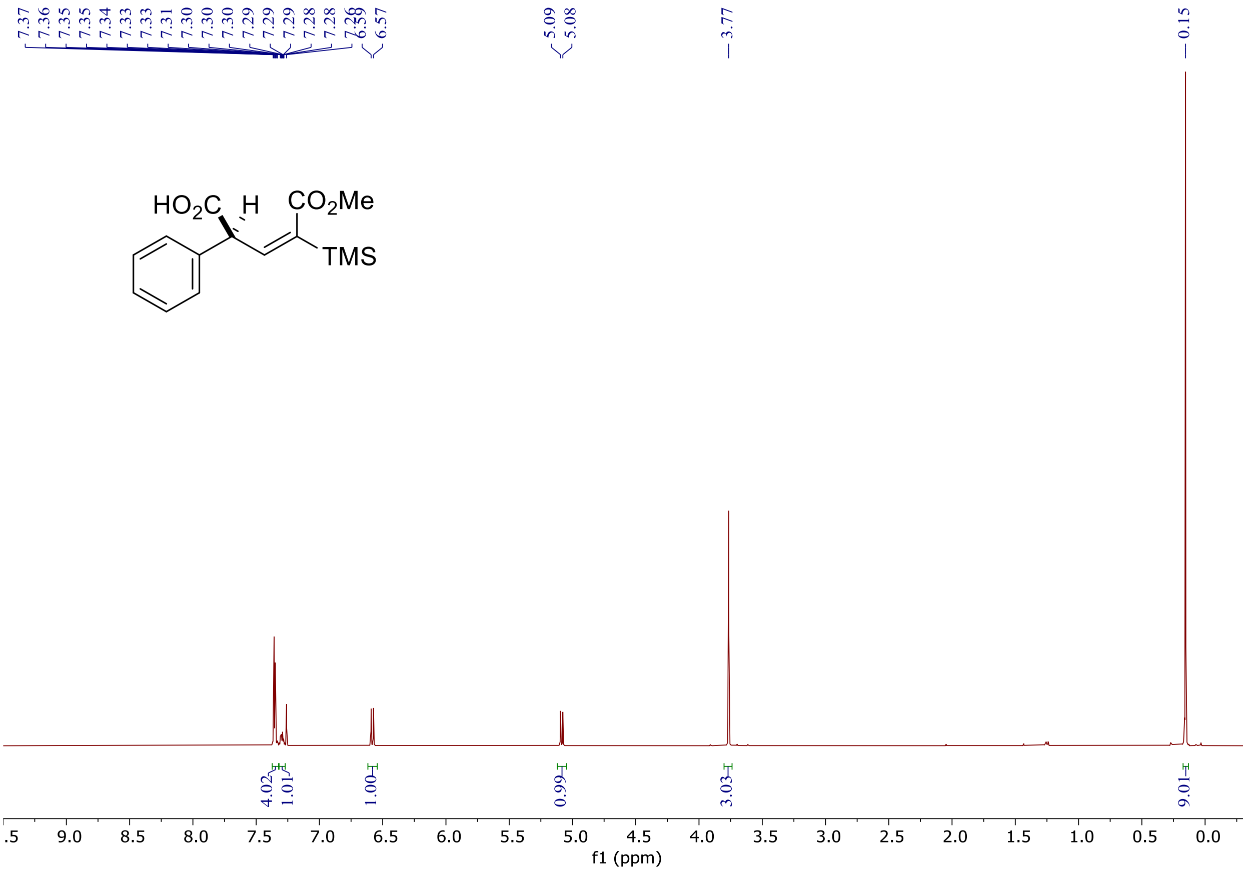

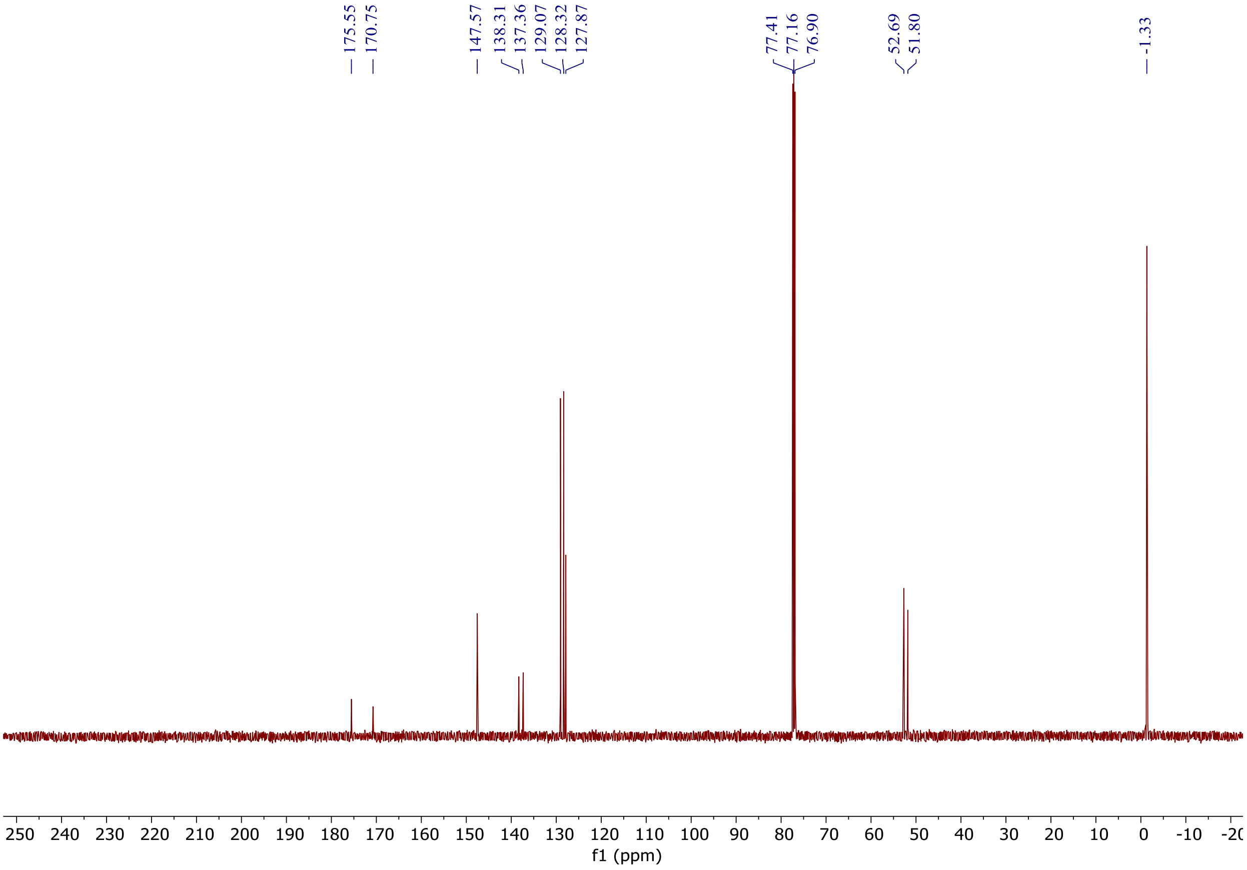

DEPT135 and HSQC traces of **4a**

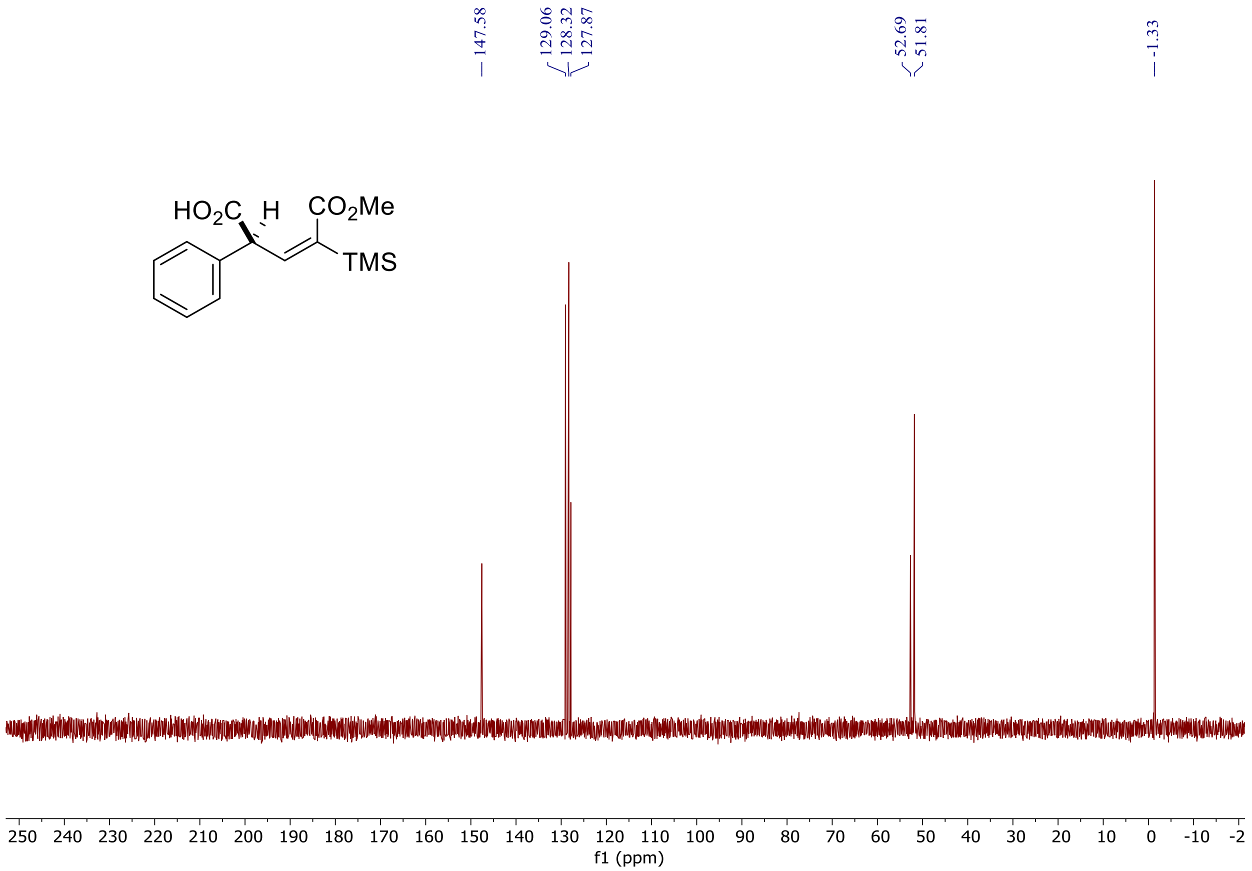

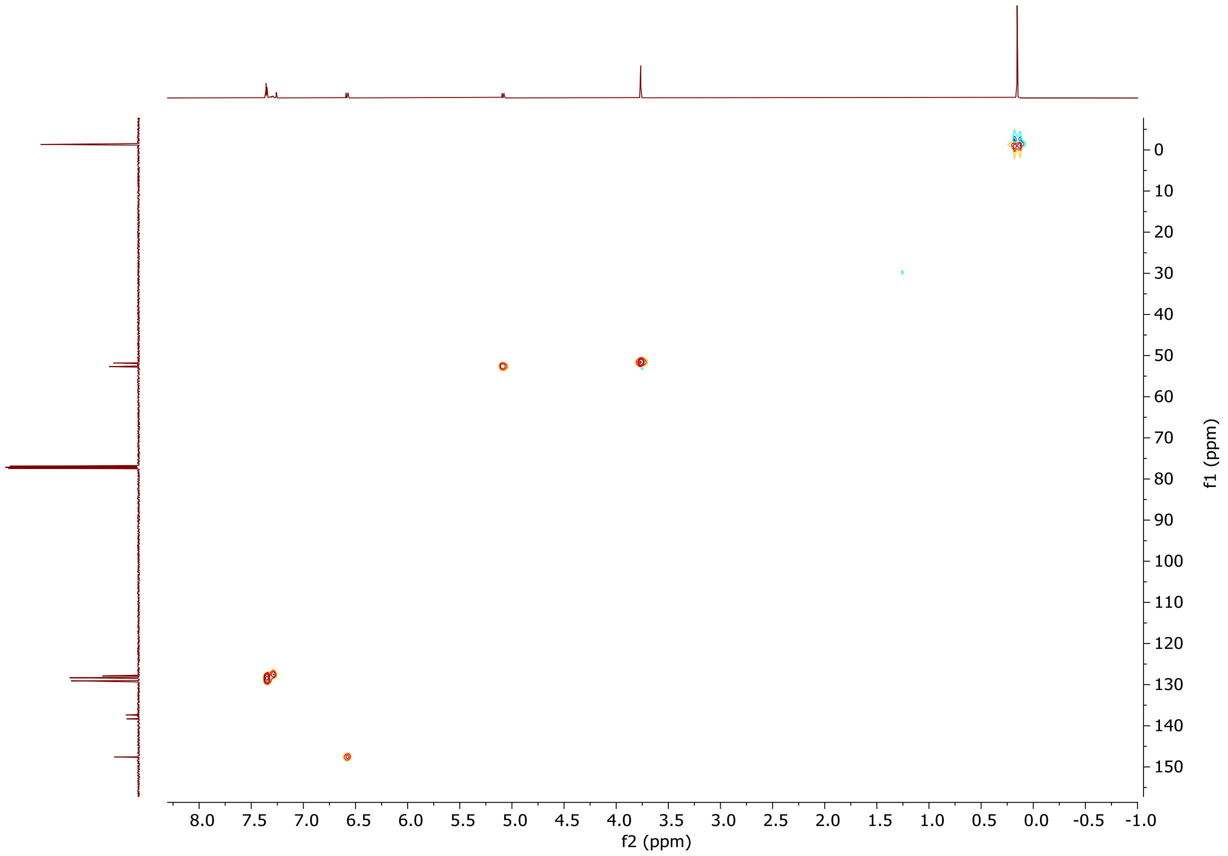

HMBC and NOESY traces of **4a**

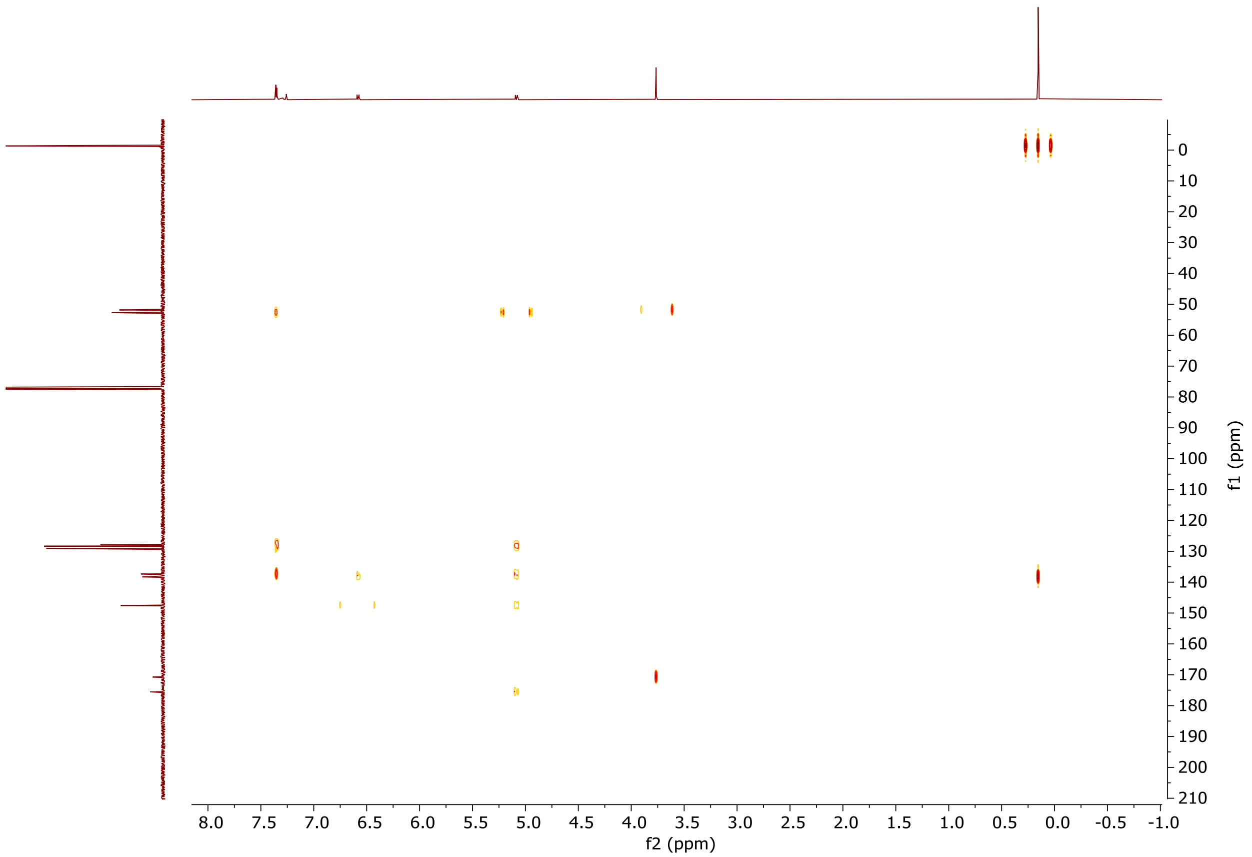

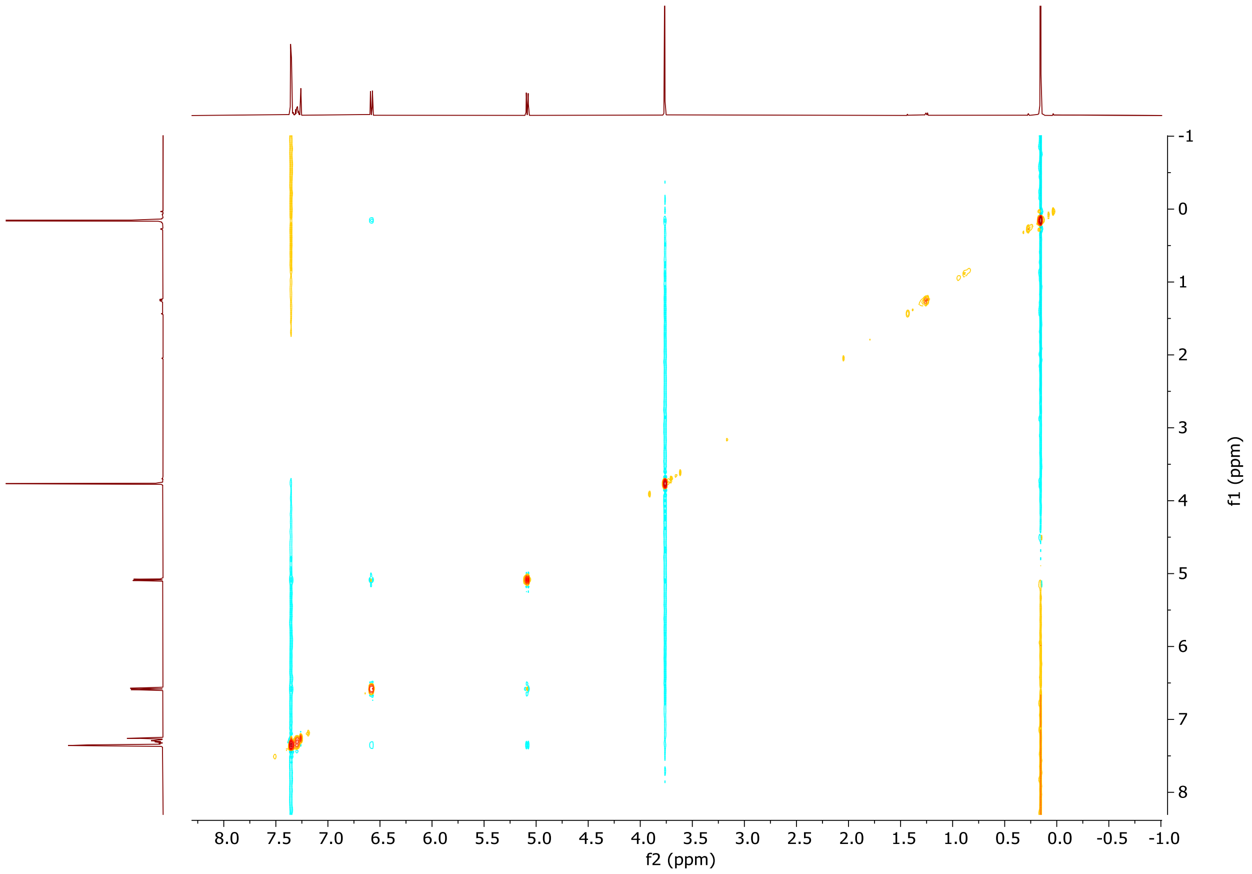

^{1}H and ^{13}C NMR traces of **4b**

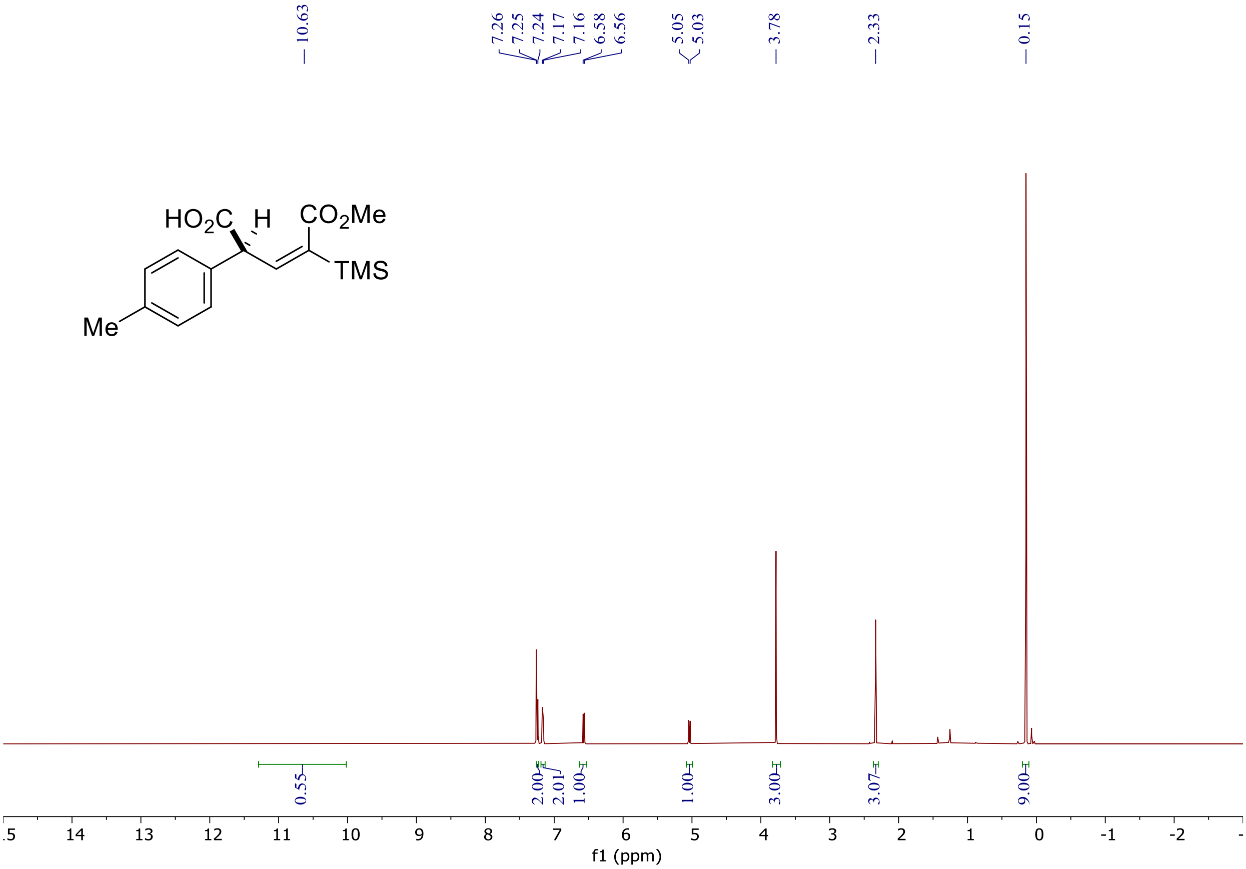

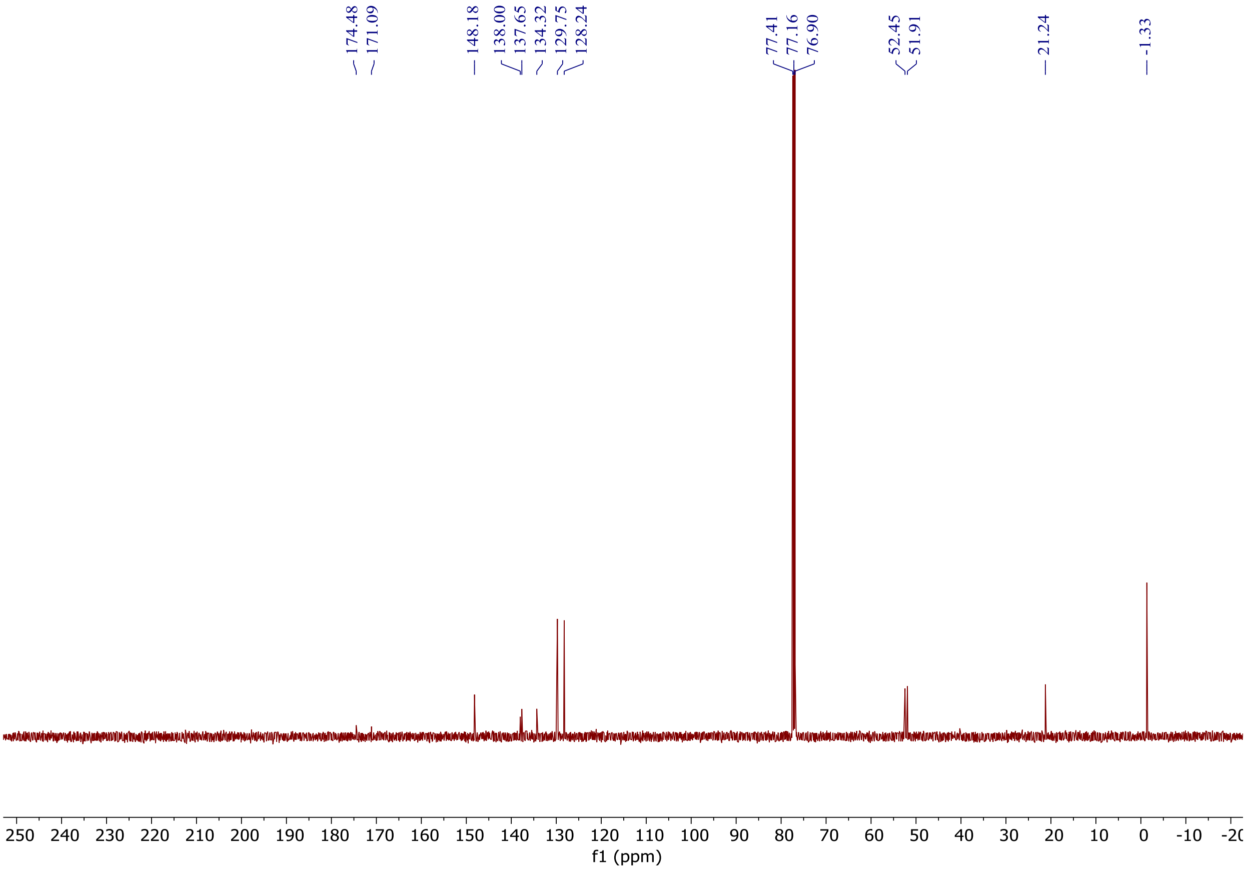

^{1}H and ^{13}C NMR traces of **4c**

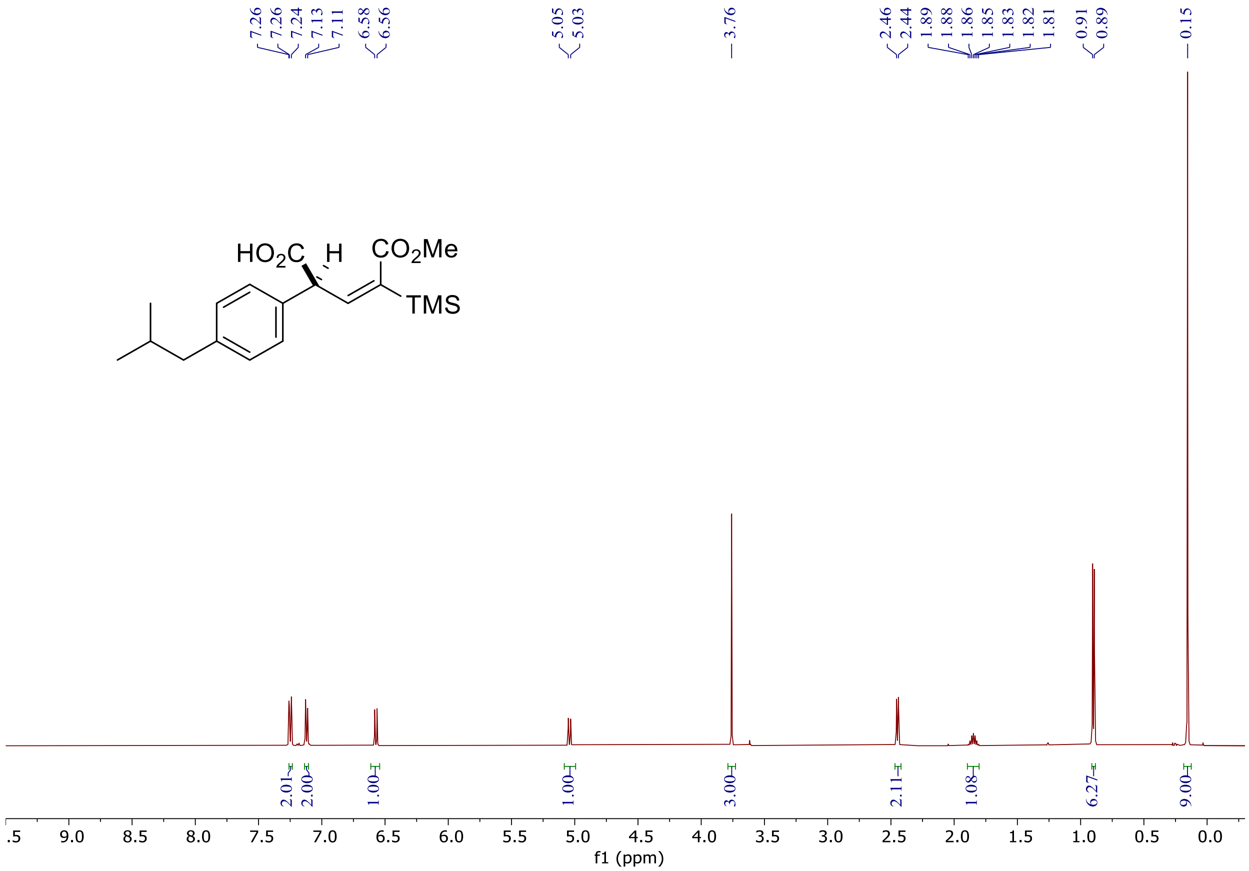

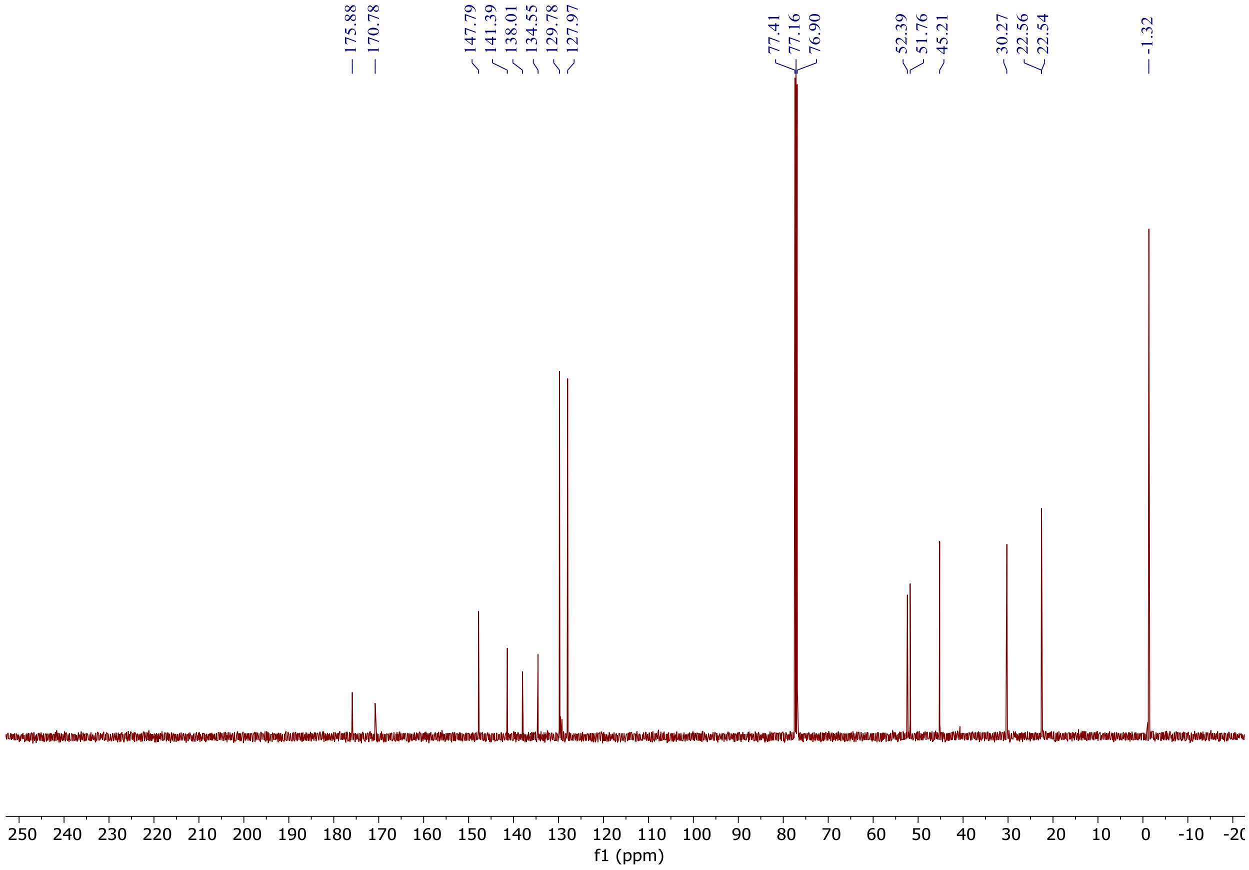

^{1}H and ^{13}C NMR traces of **4d**

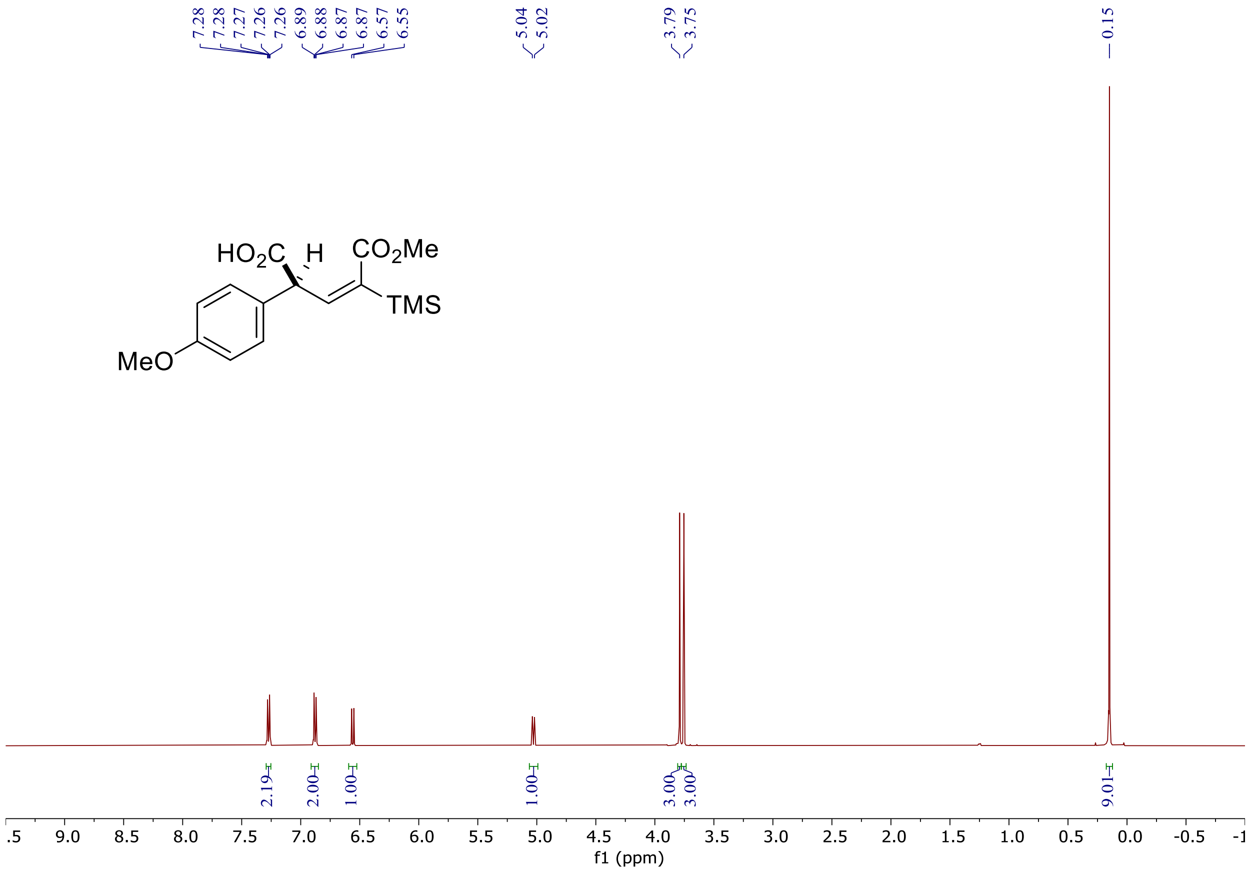

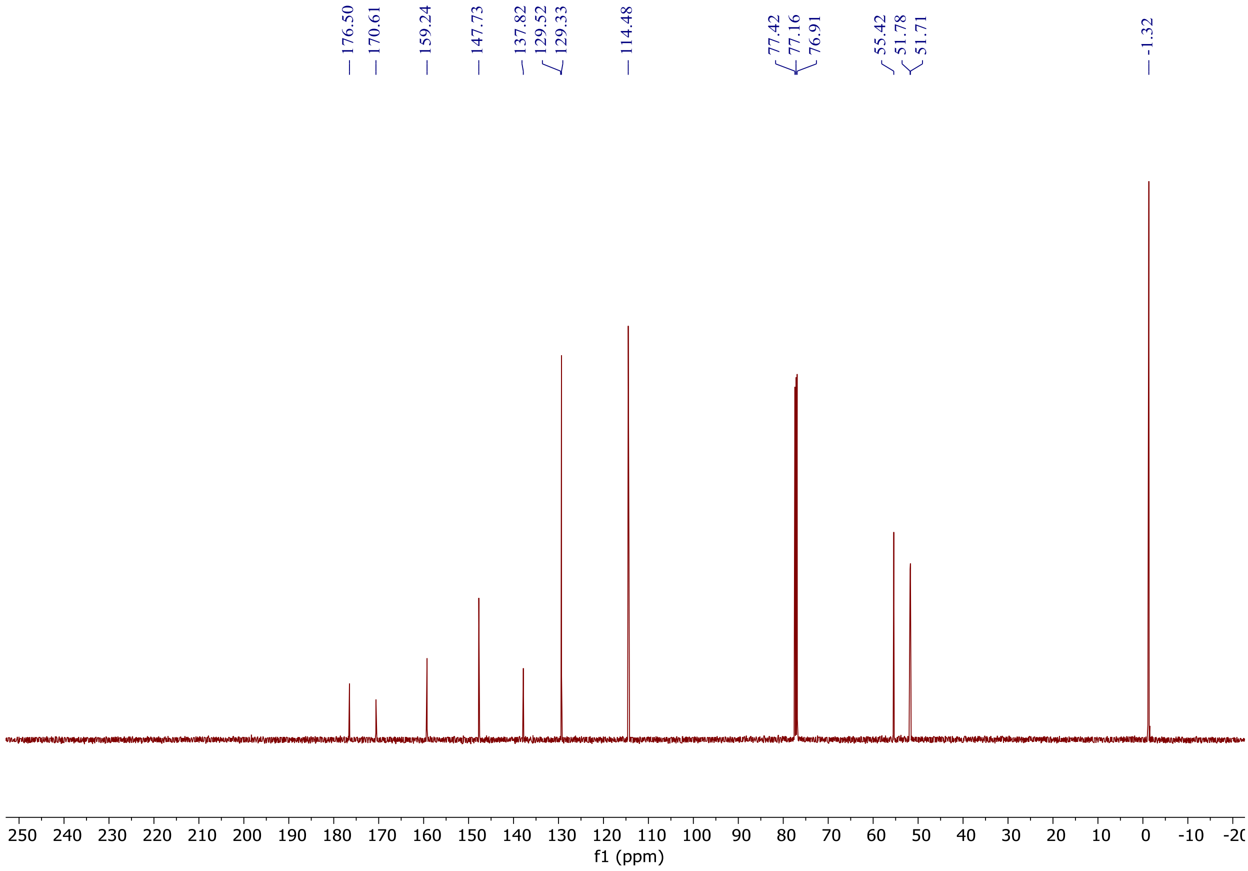

^{1}H and ^{13}C NMR traces of **4e**

^{13}C NMR (126 MHz, Chloroform-*d*) δ 175.5, 170.5, 146.8, 138.8, 136.4, 132.2, 130.0, 122.0, 52.0, 51.9, -1.4.

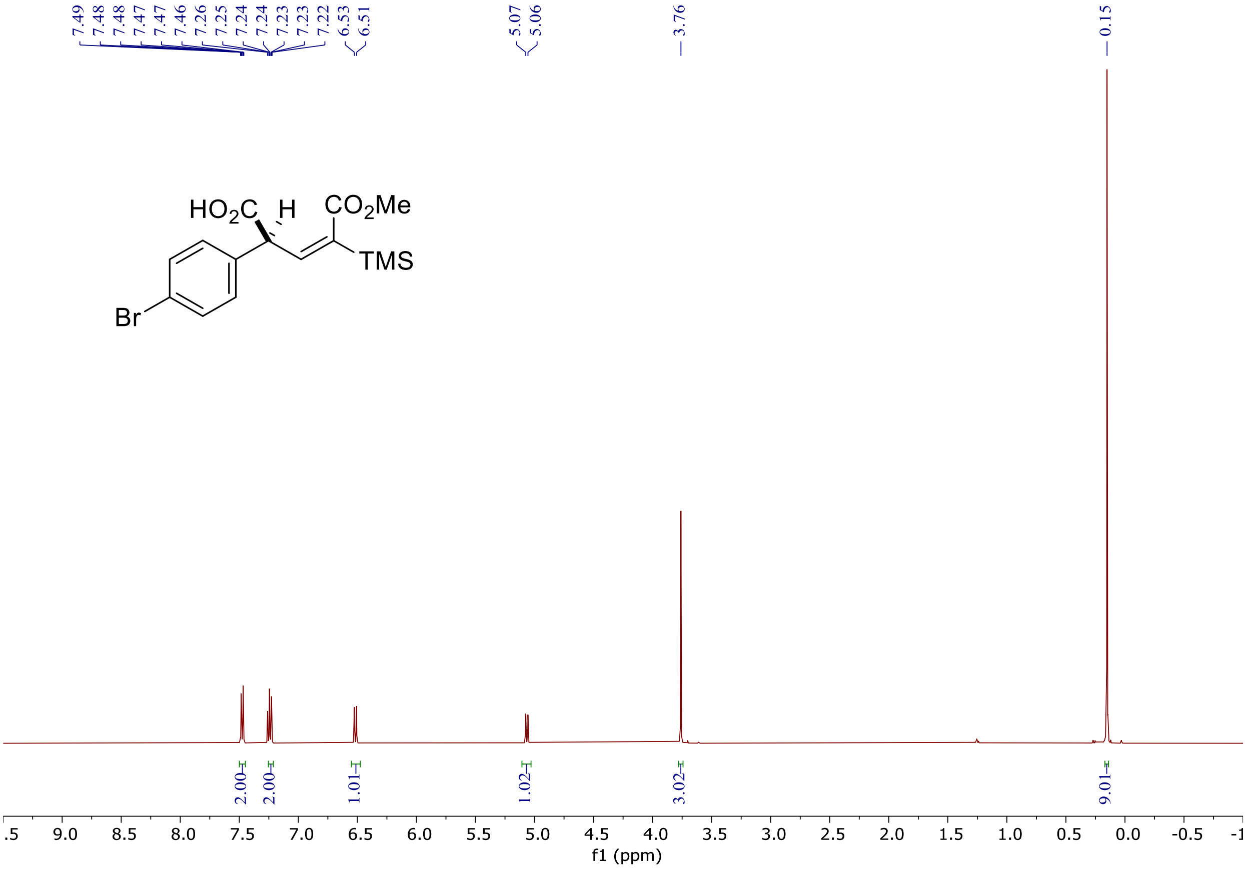

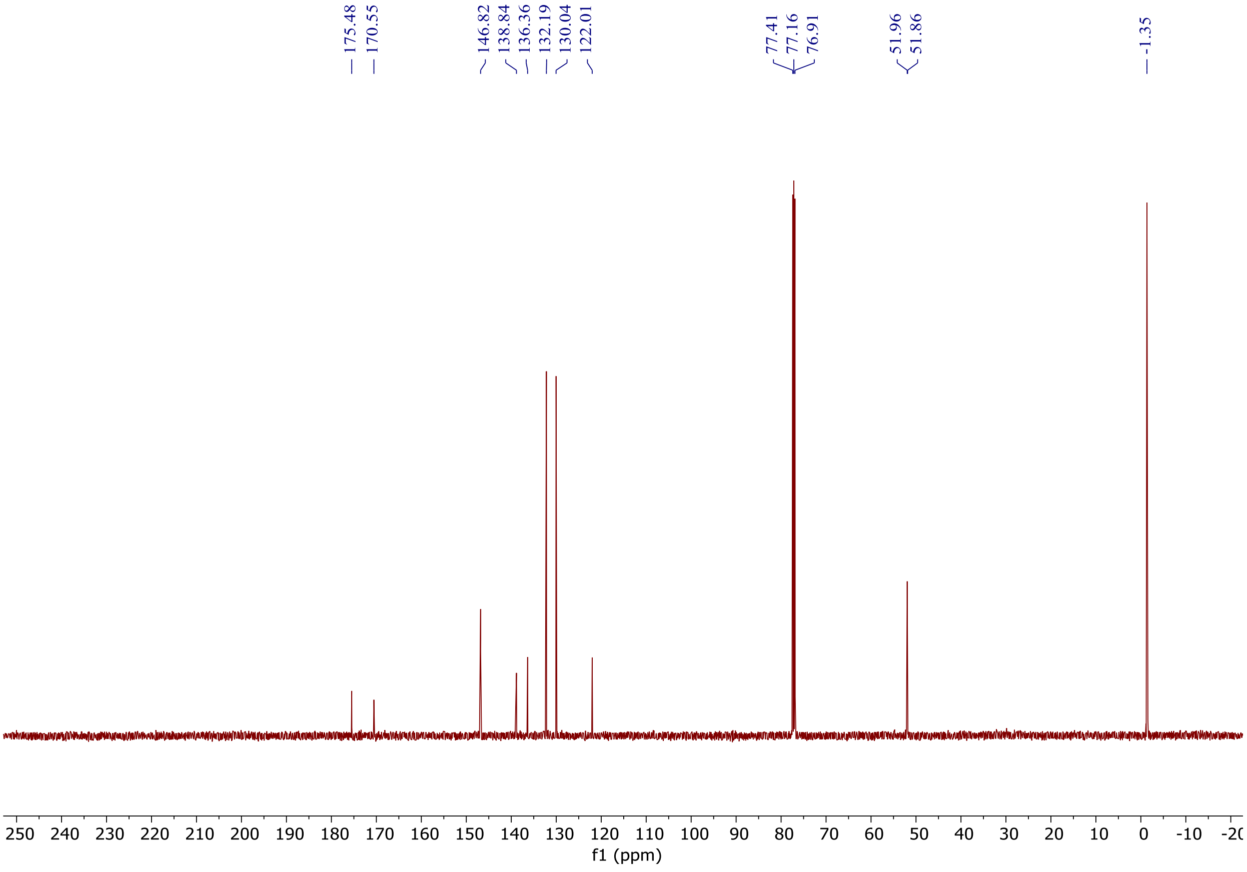

^{1}H, ^{13}C and ^{19}F NMR traces of **4f**

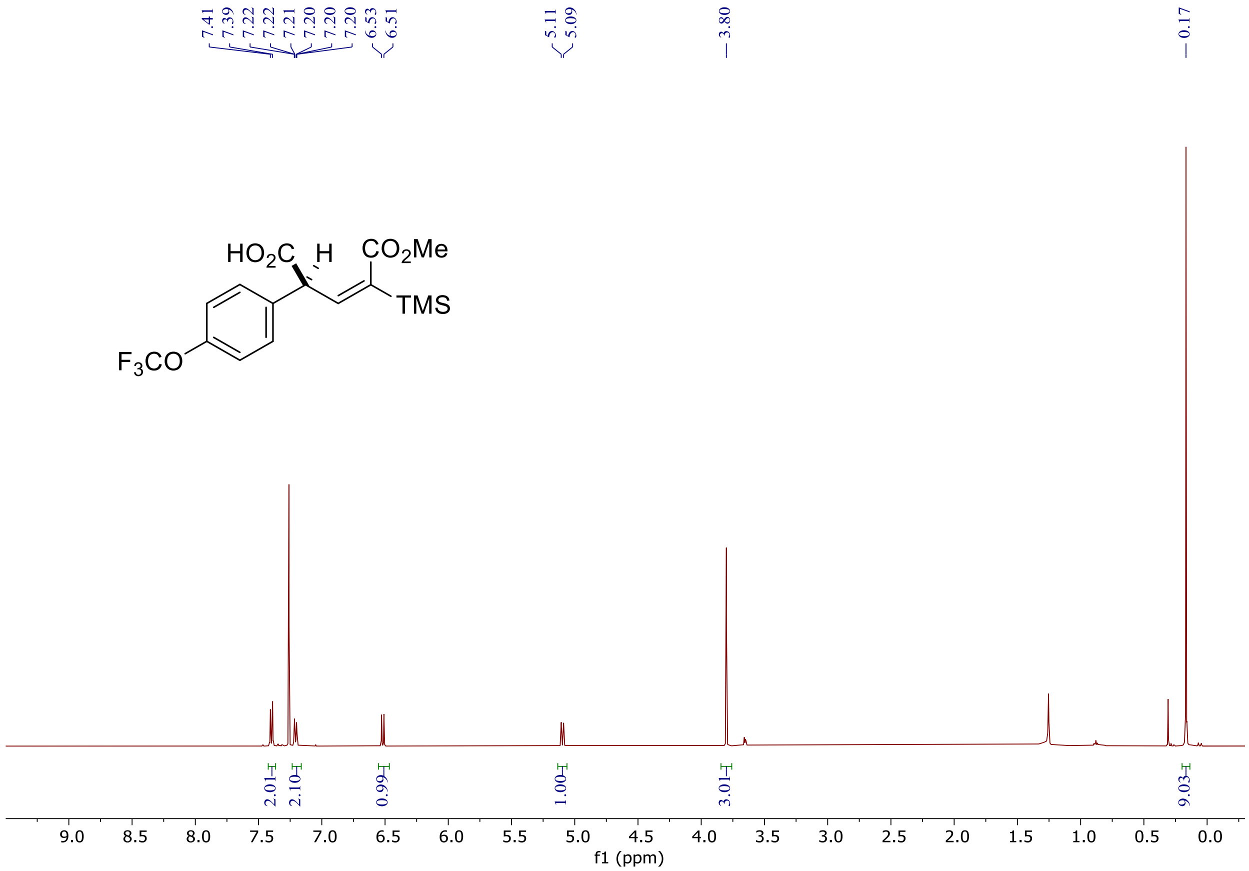

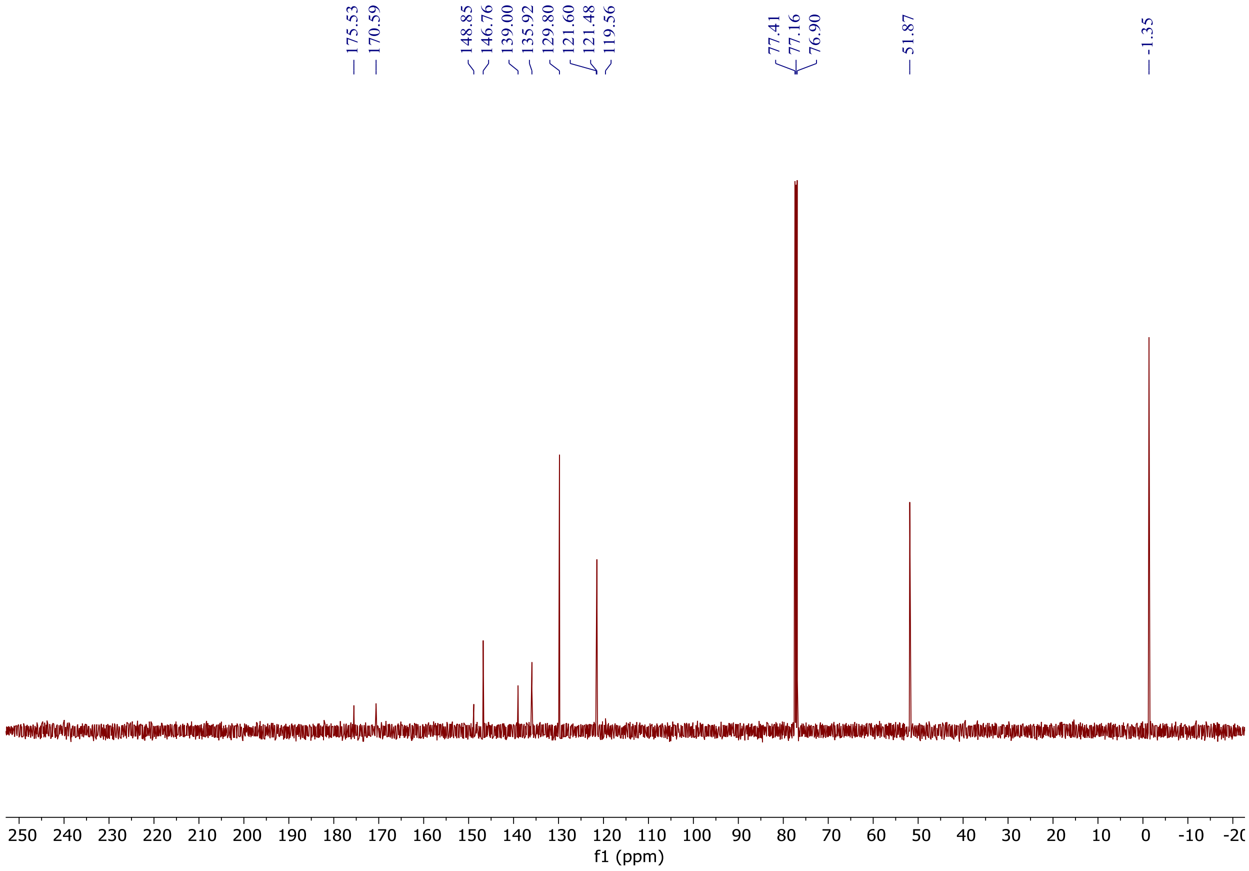

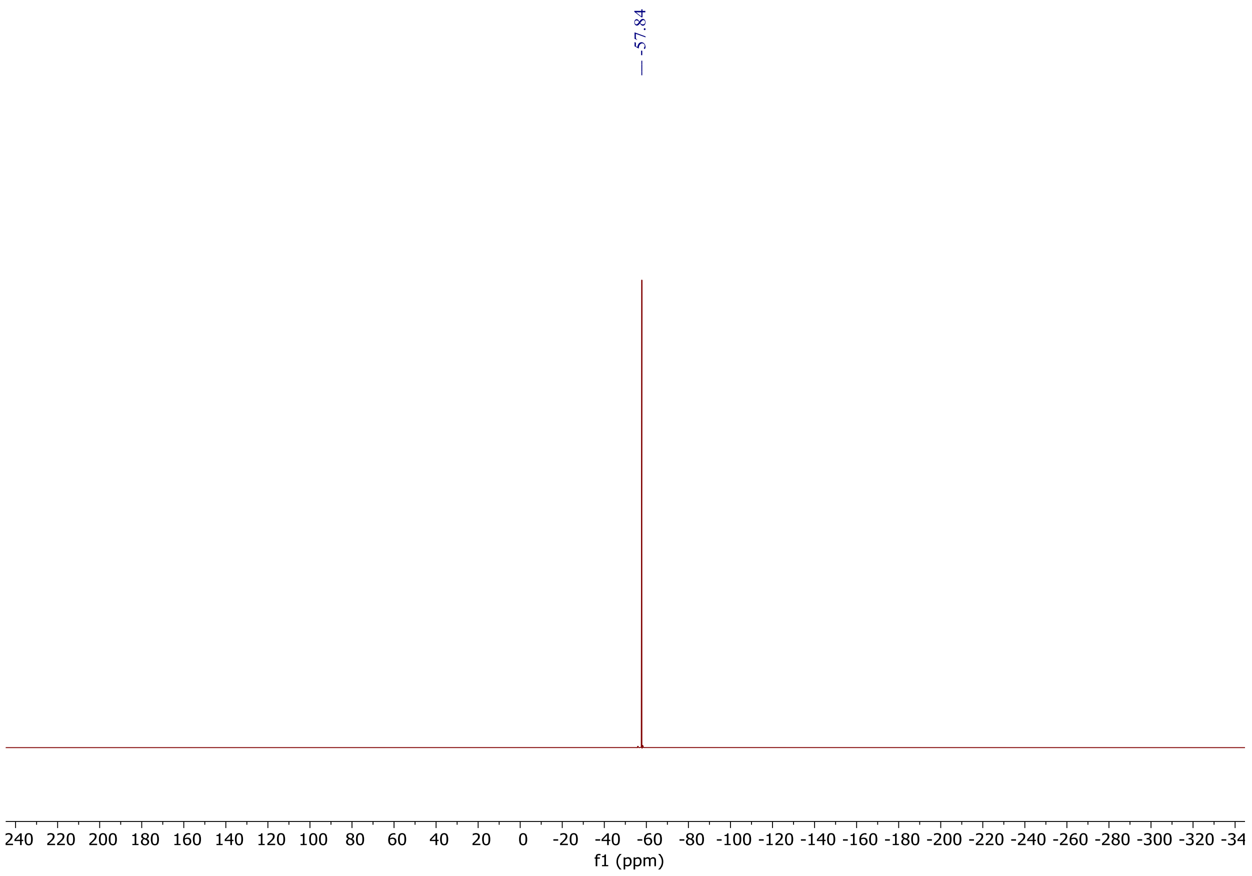

-57.84
240 220 200 180 160 140 120 100 80 60 40 20 0 -20 -40 -60 -80 -100 -120 -140 -160 -180 -200 -220 -240 -260 -280 -300 -320 -34
f1 (ppm)

^{1}H and ^{13}C NMR traces of **4g**

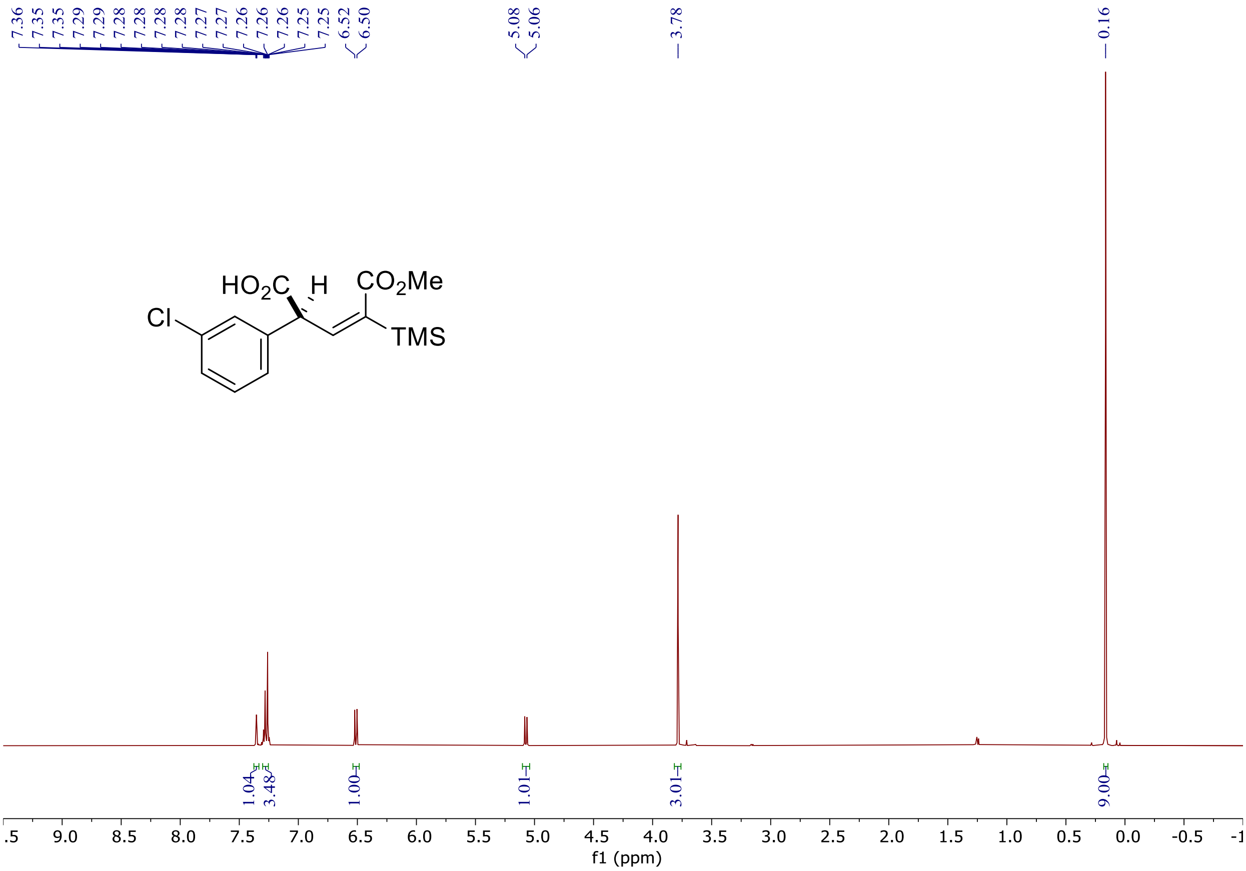

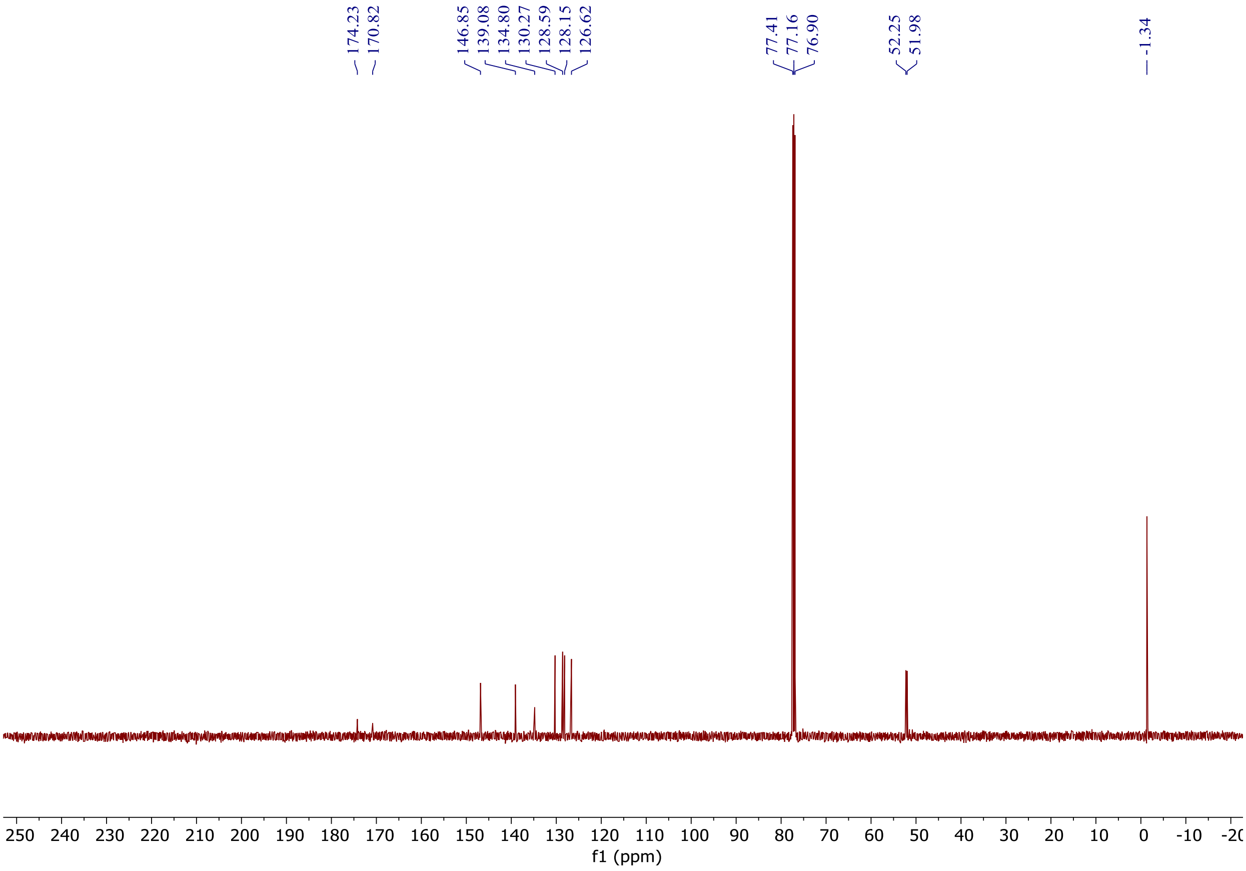

^1H and ^{13}C NMR traces of **4h**

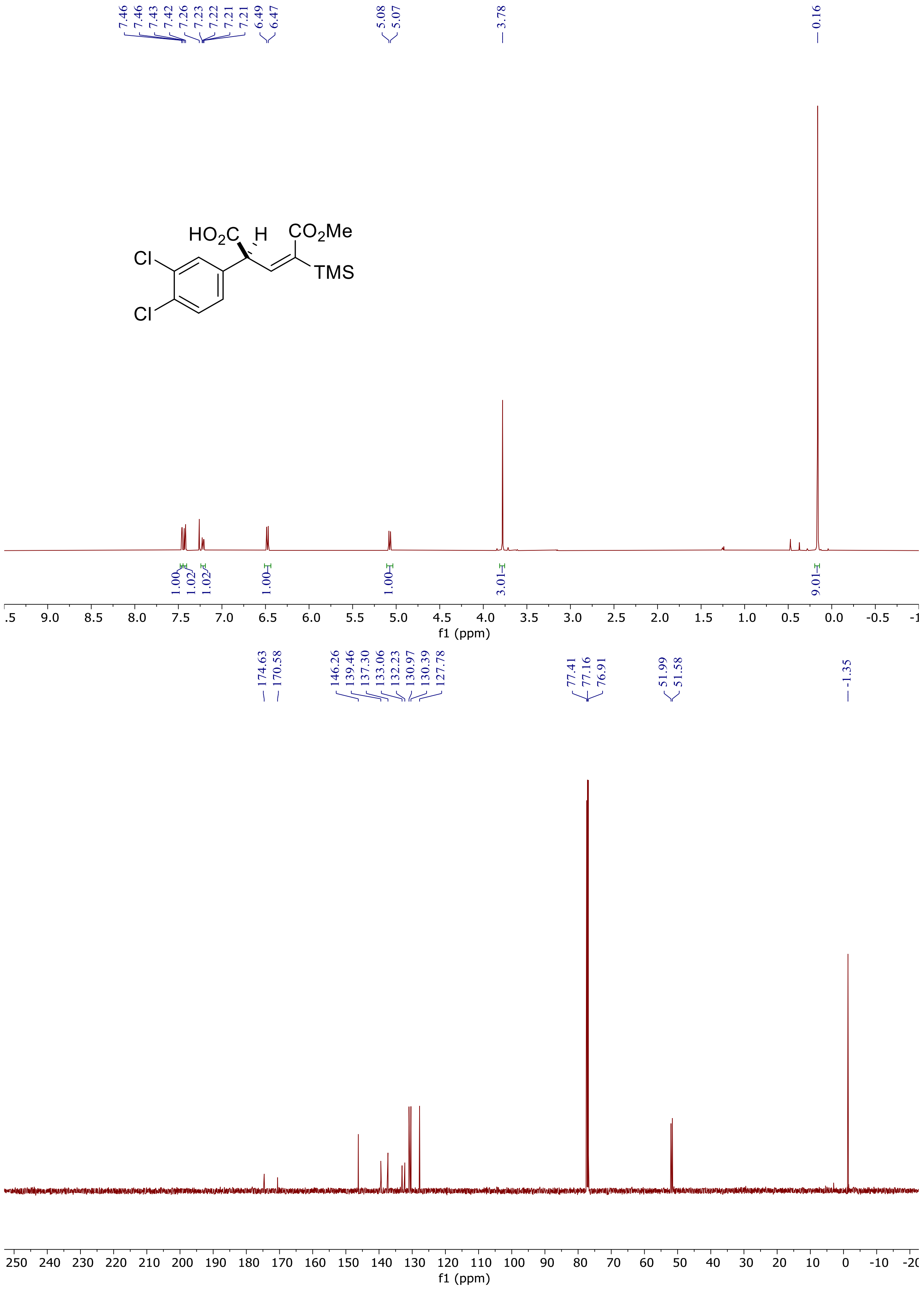

^{1}H and ^{13}C NMR traces of **4i**

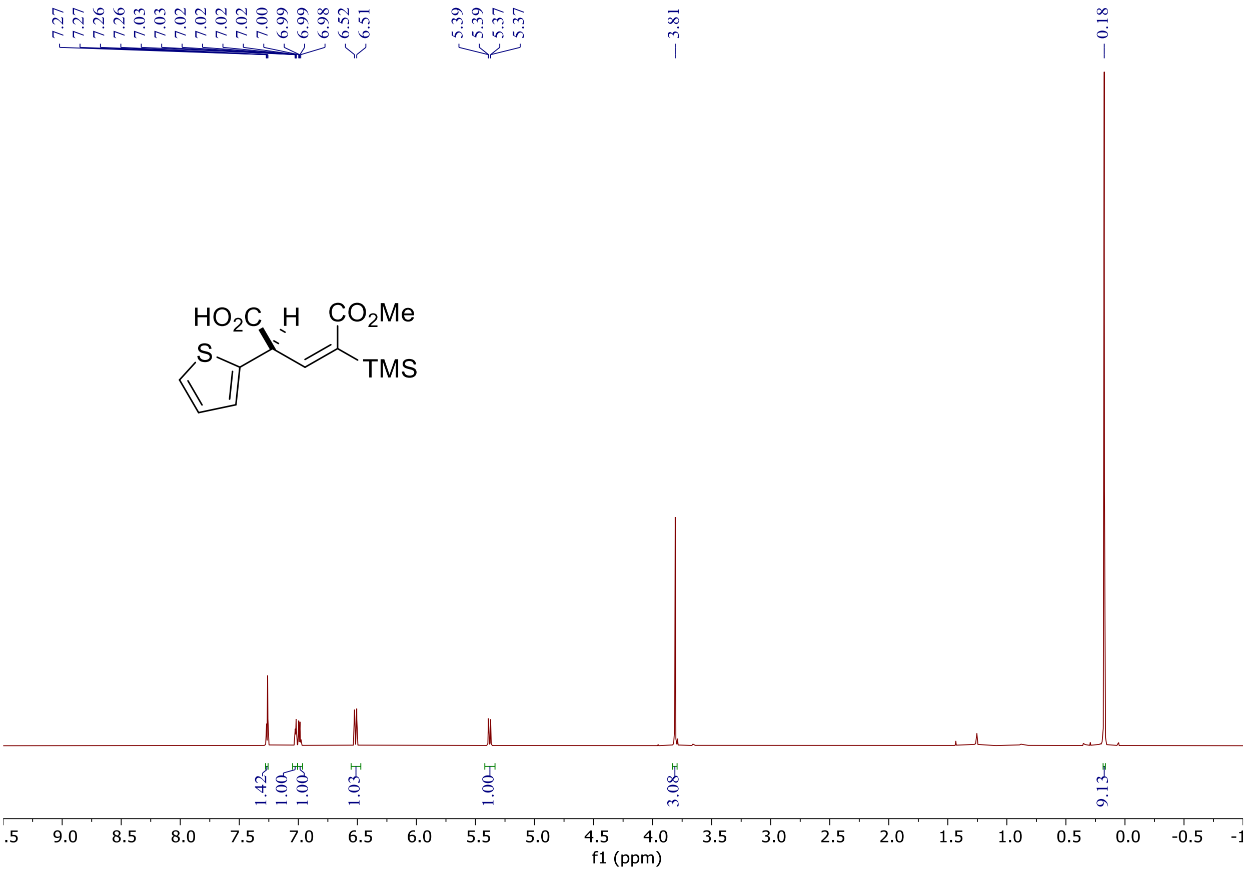

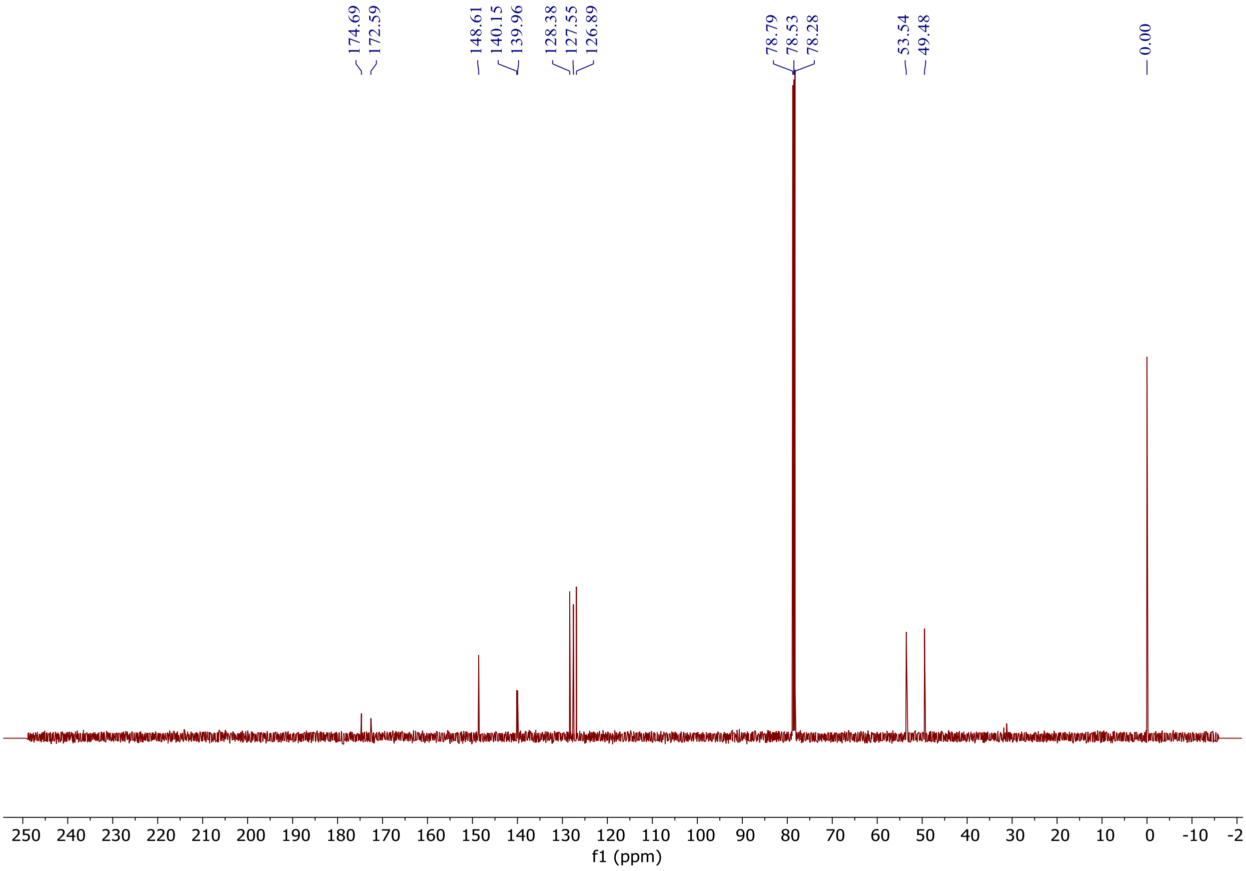

^{1}H and ^{13}C NMR traces of **4j**

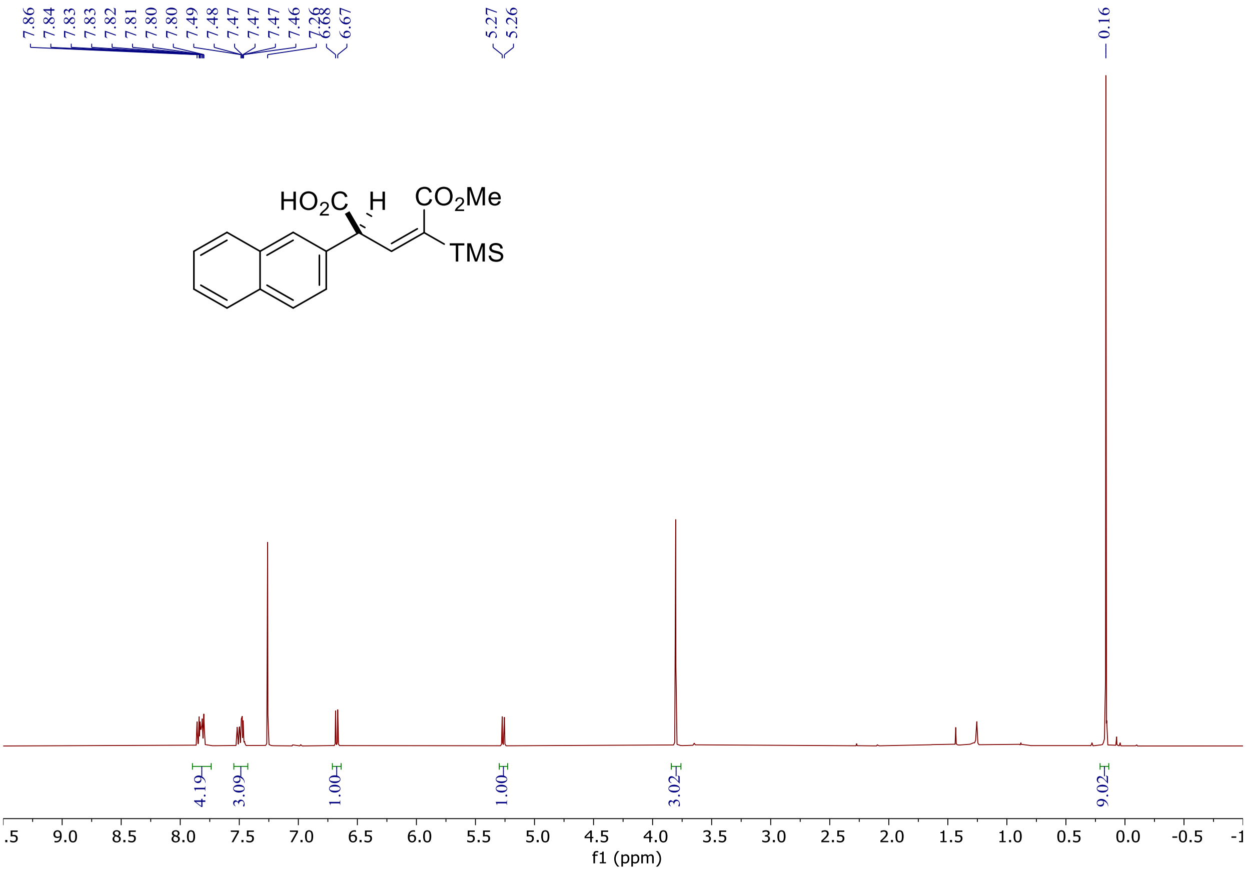

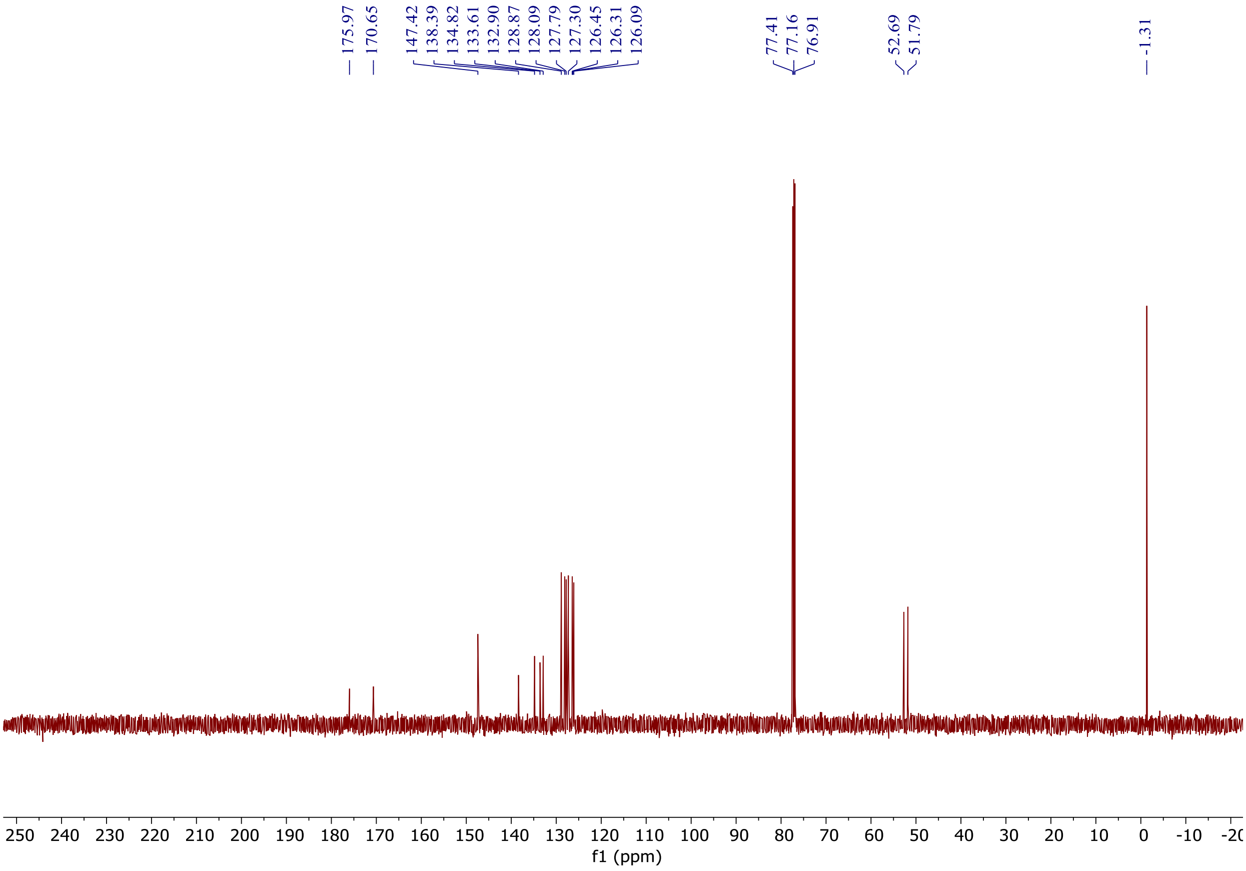

^{1}H and ^{13}C NMR traces of **4k**

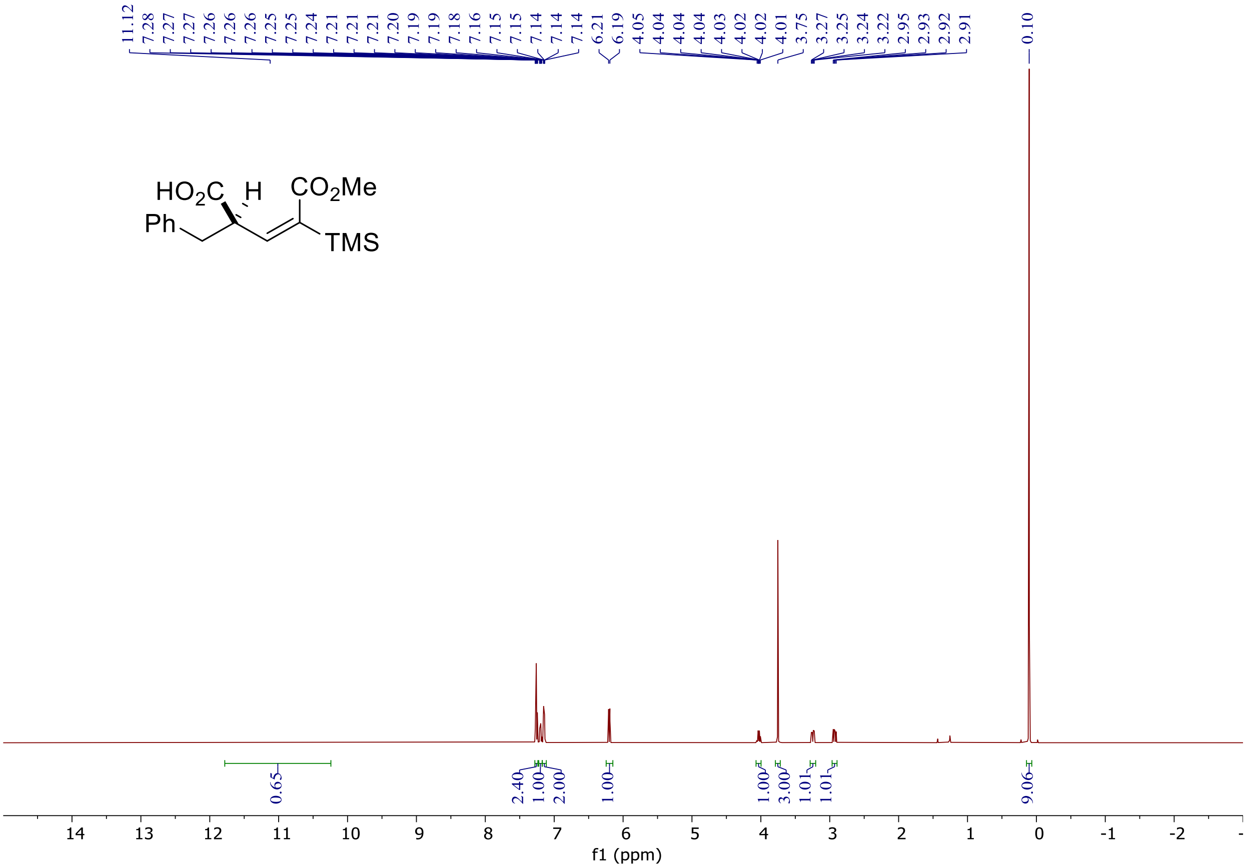

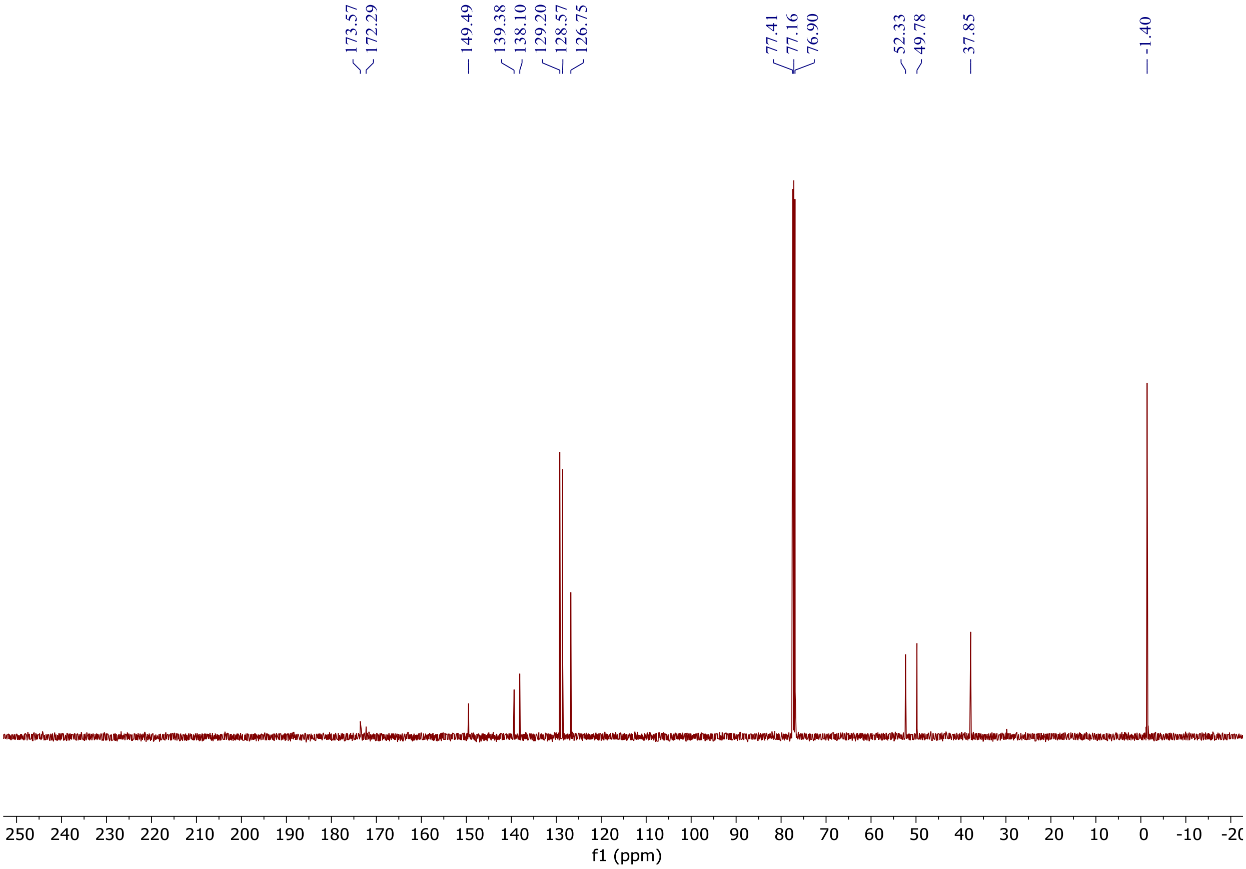

^{1}H and ^{13}C NMR traces of **4l**

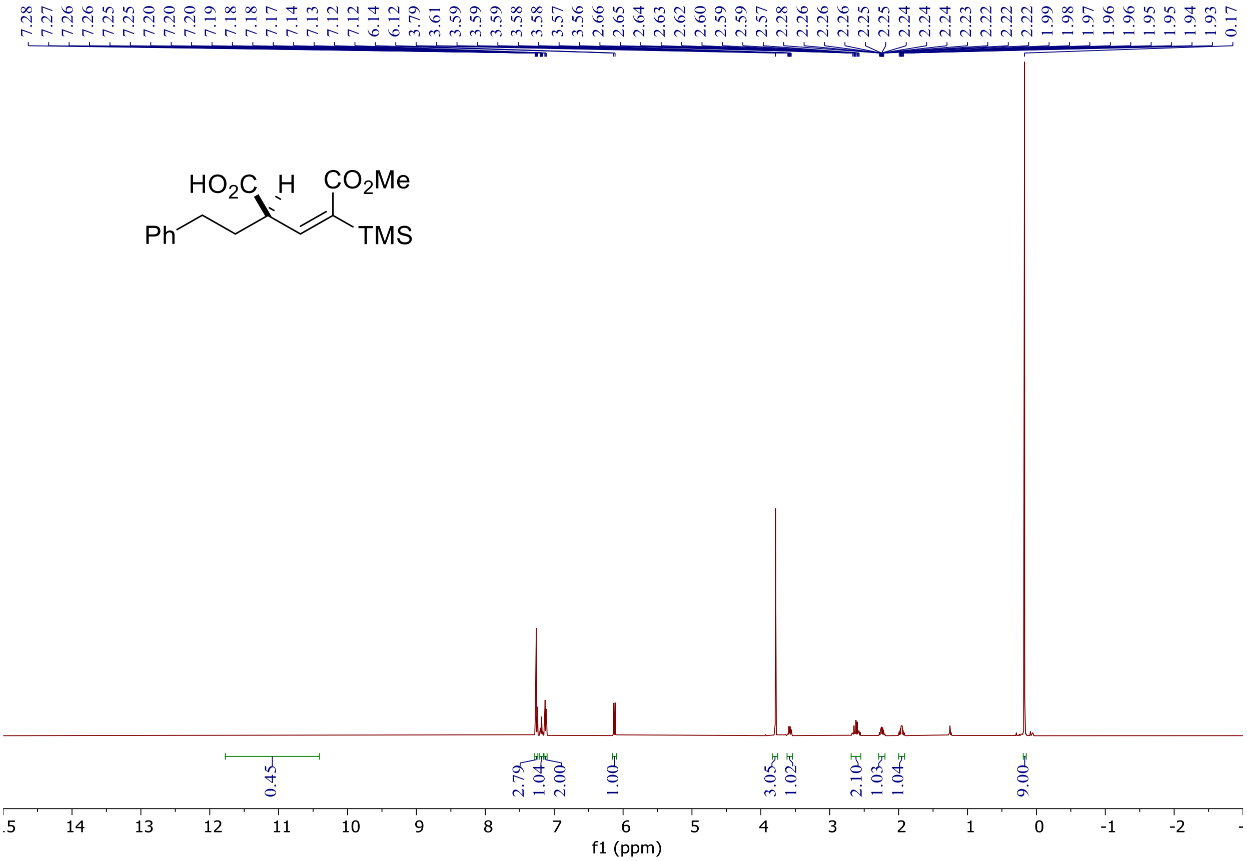

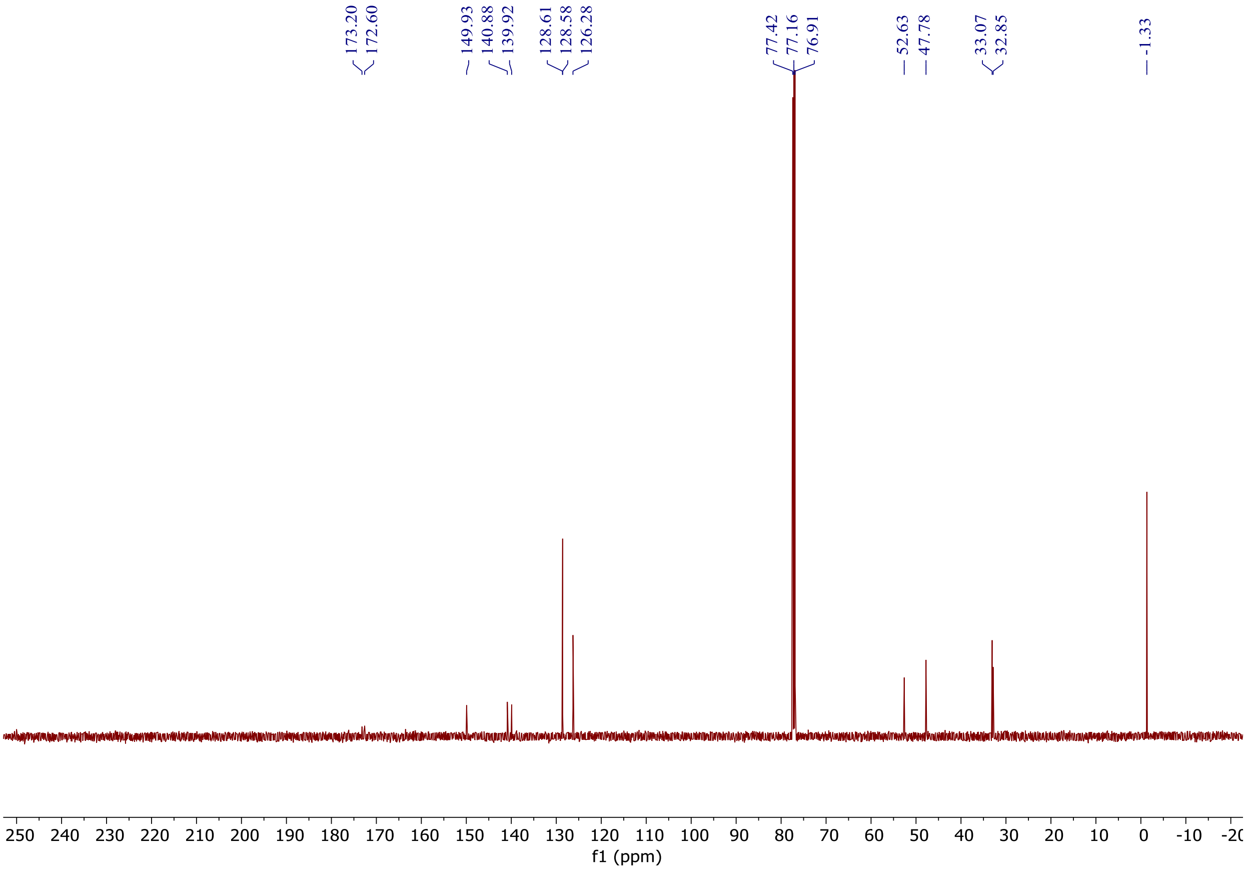

^{1}H and ^{13}C NMR traces of **4m**

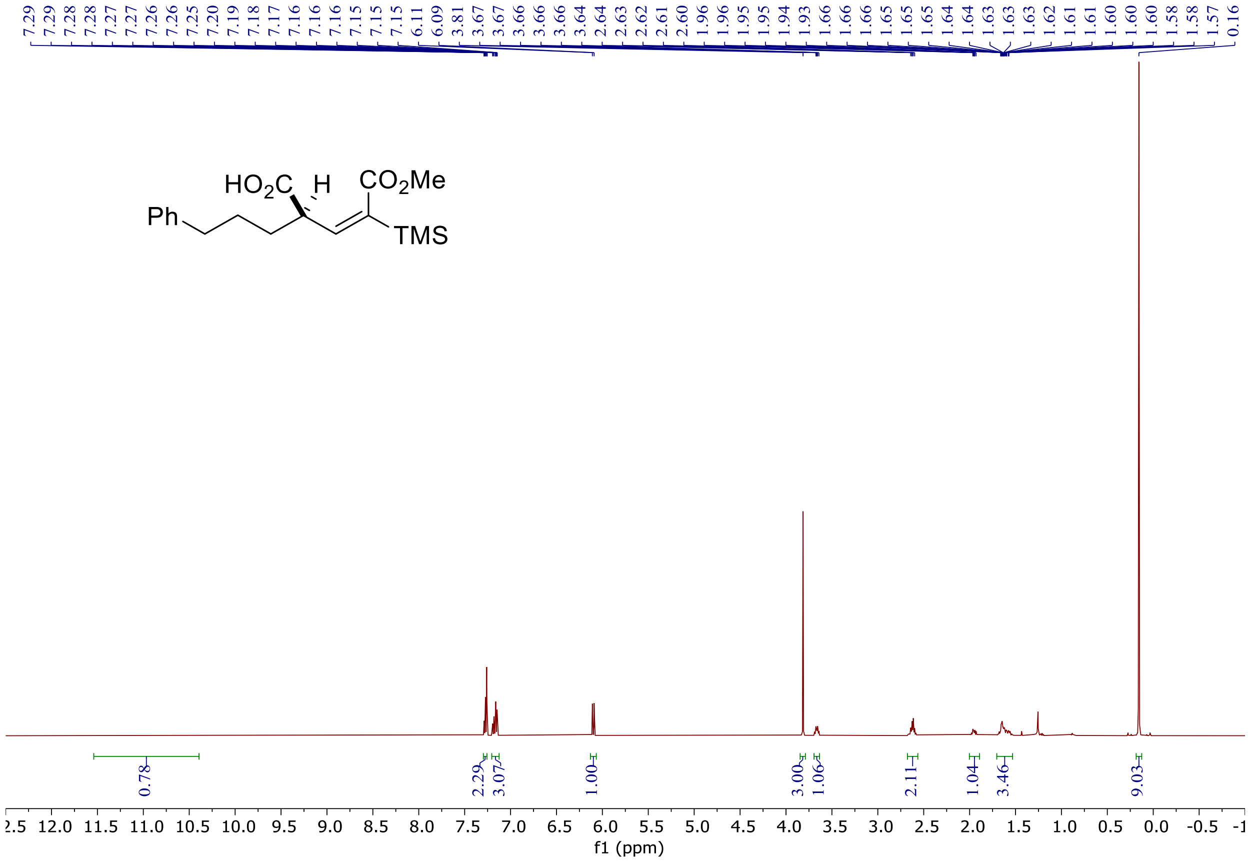

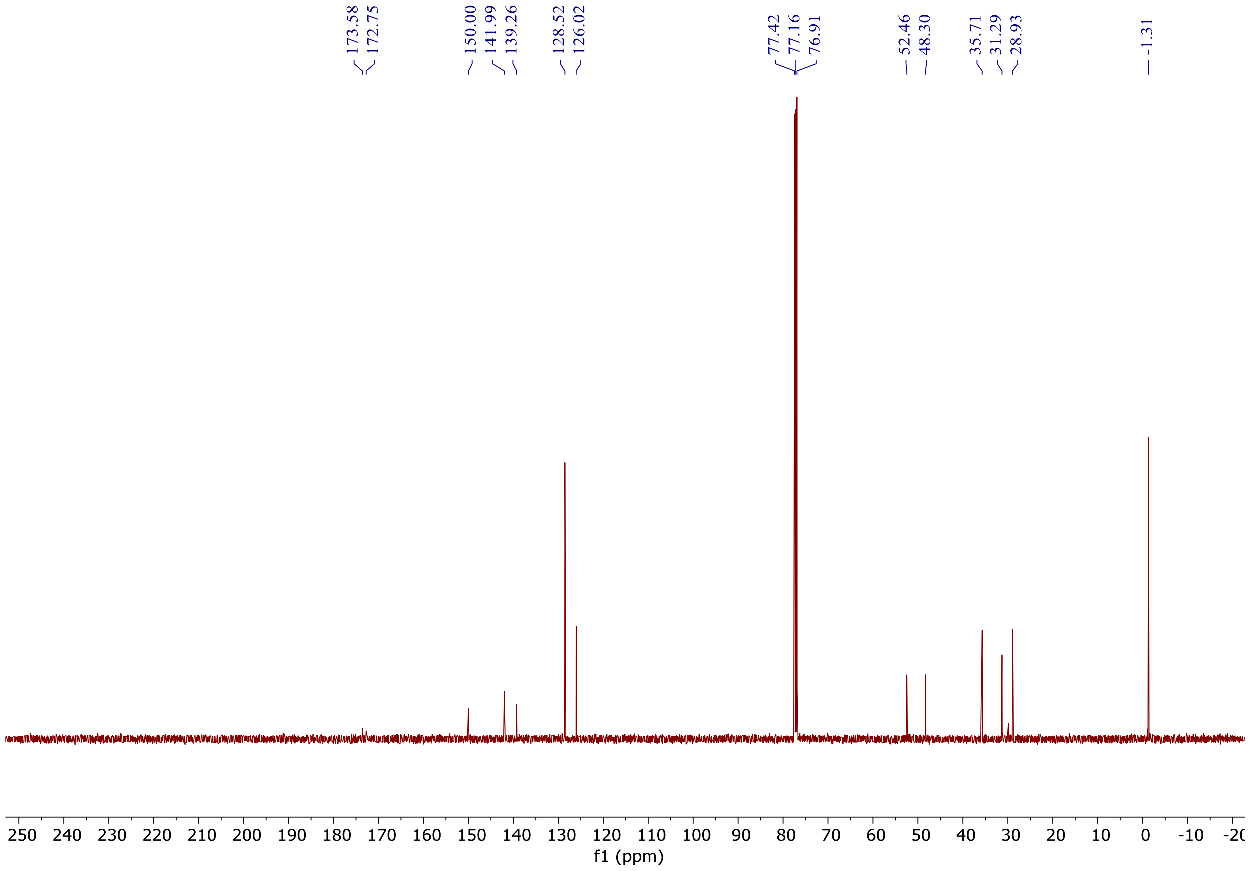

^{1}H and ^{13}C NMR traces of **4n**

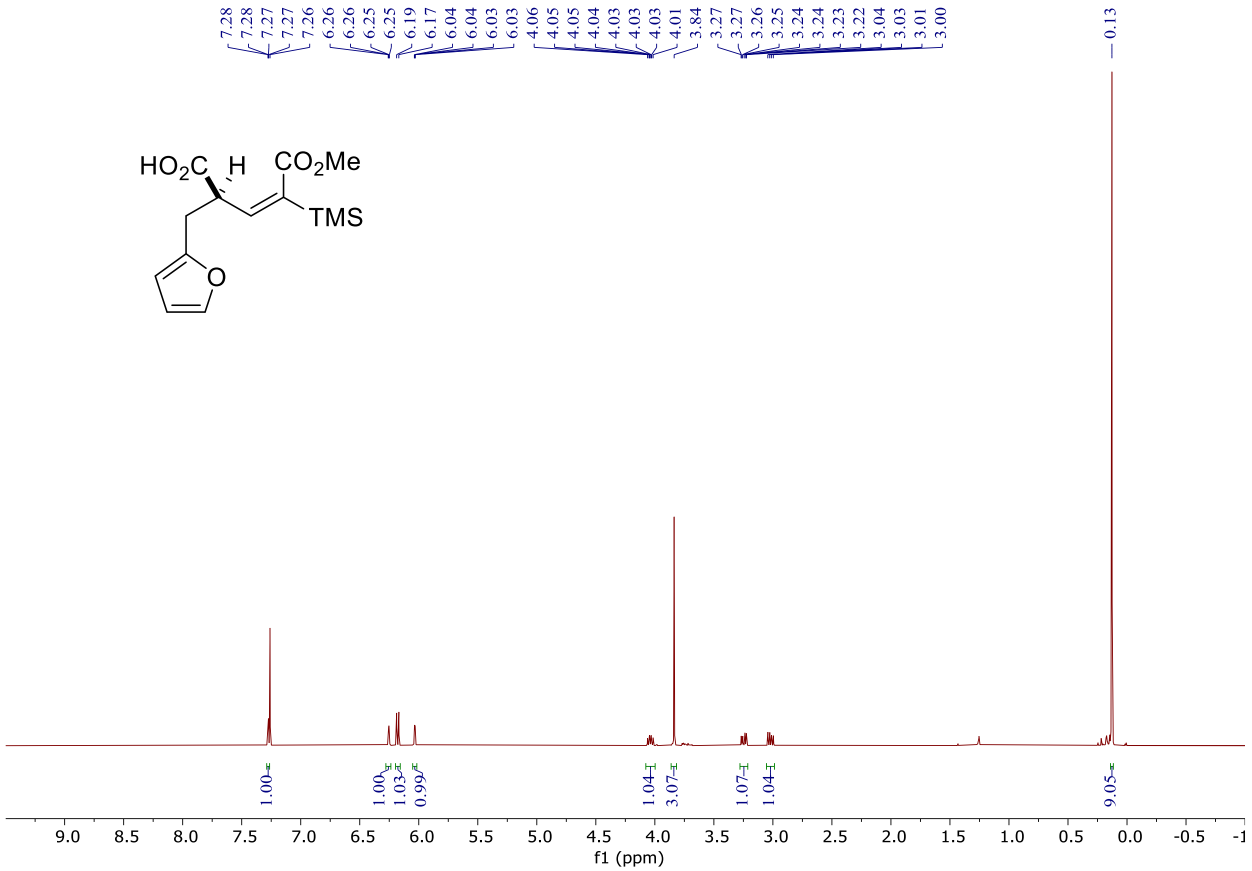

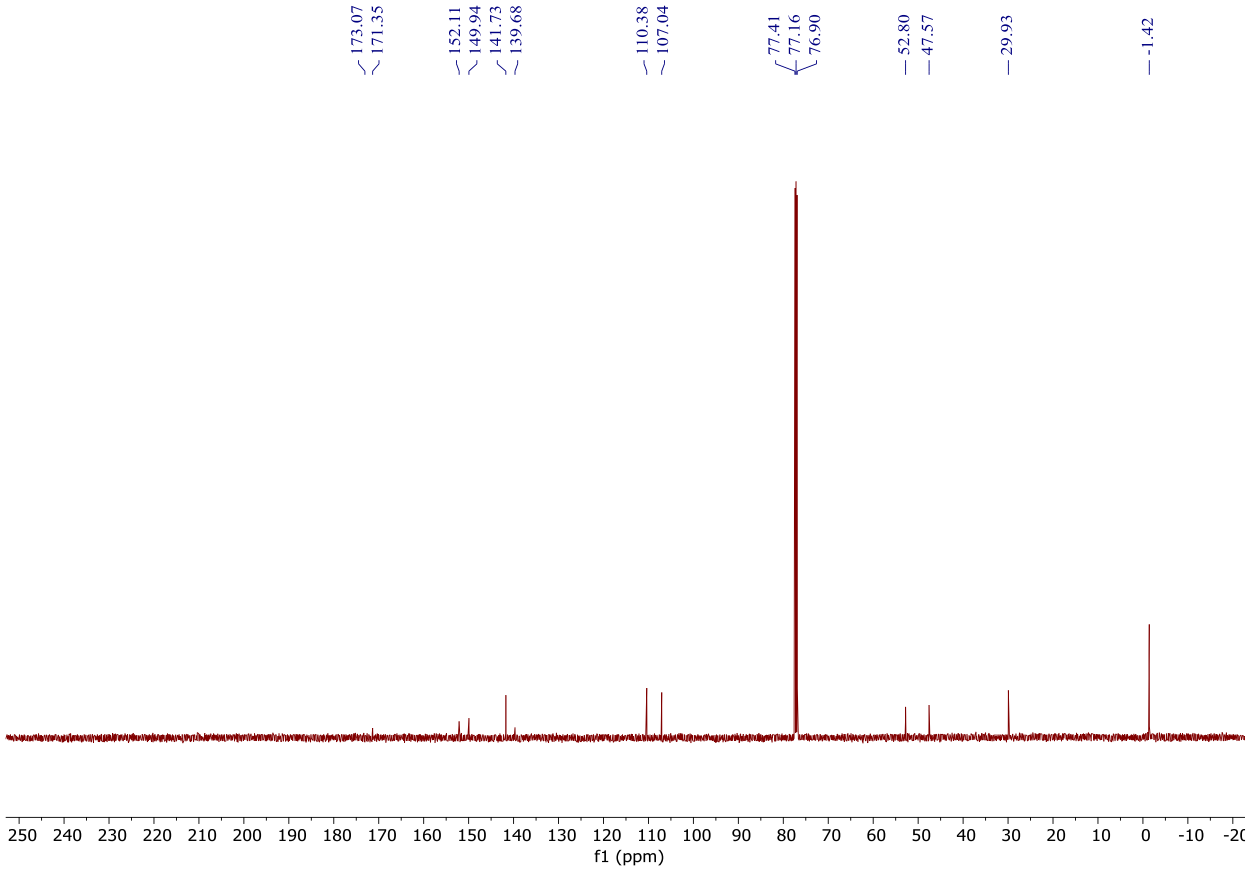

^{1}H and ^{13}C NMR traces of **4o**

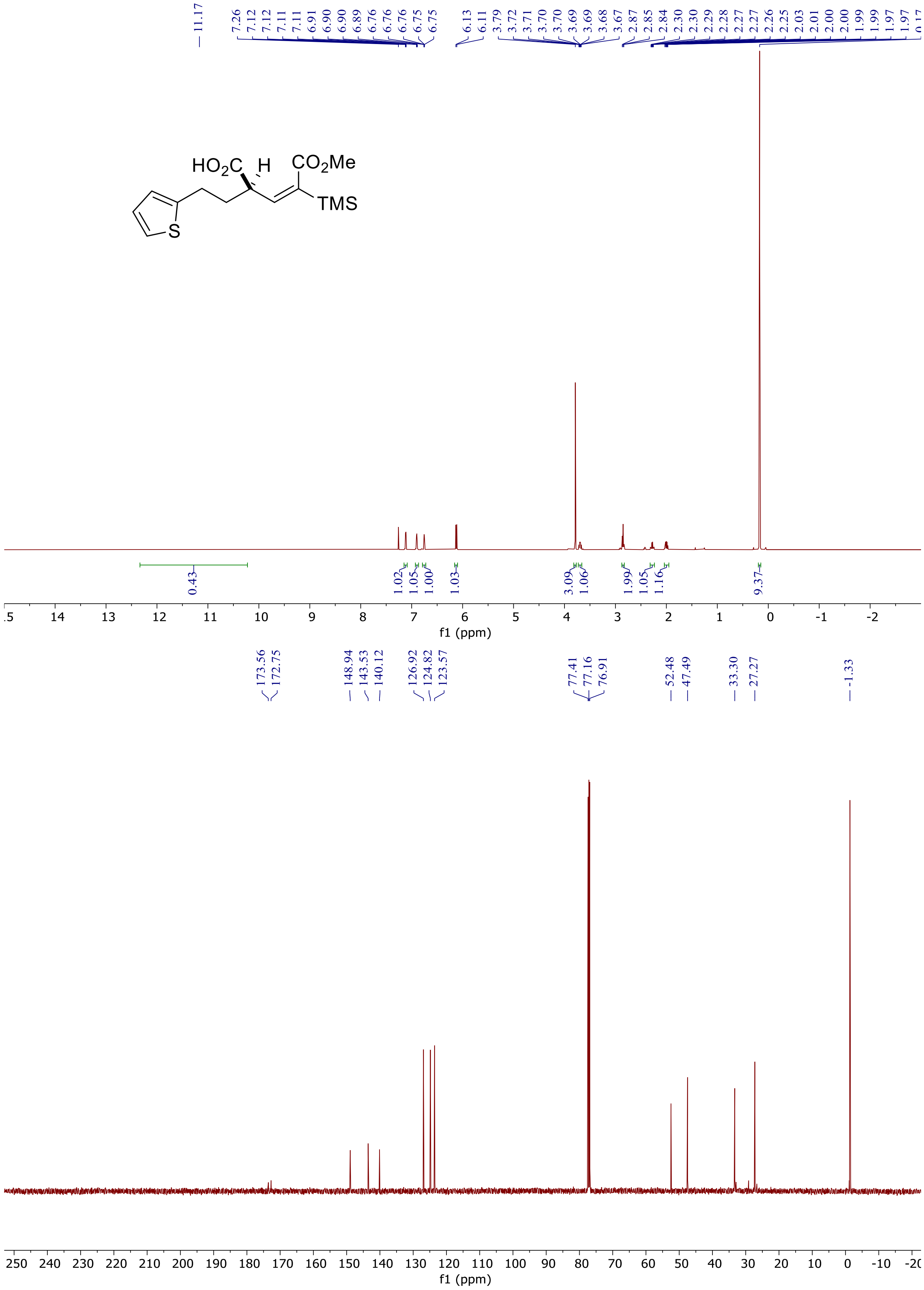

^{1}H and ^{13}C NMR traces of **4p**

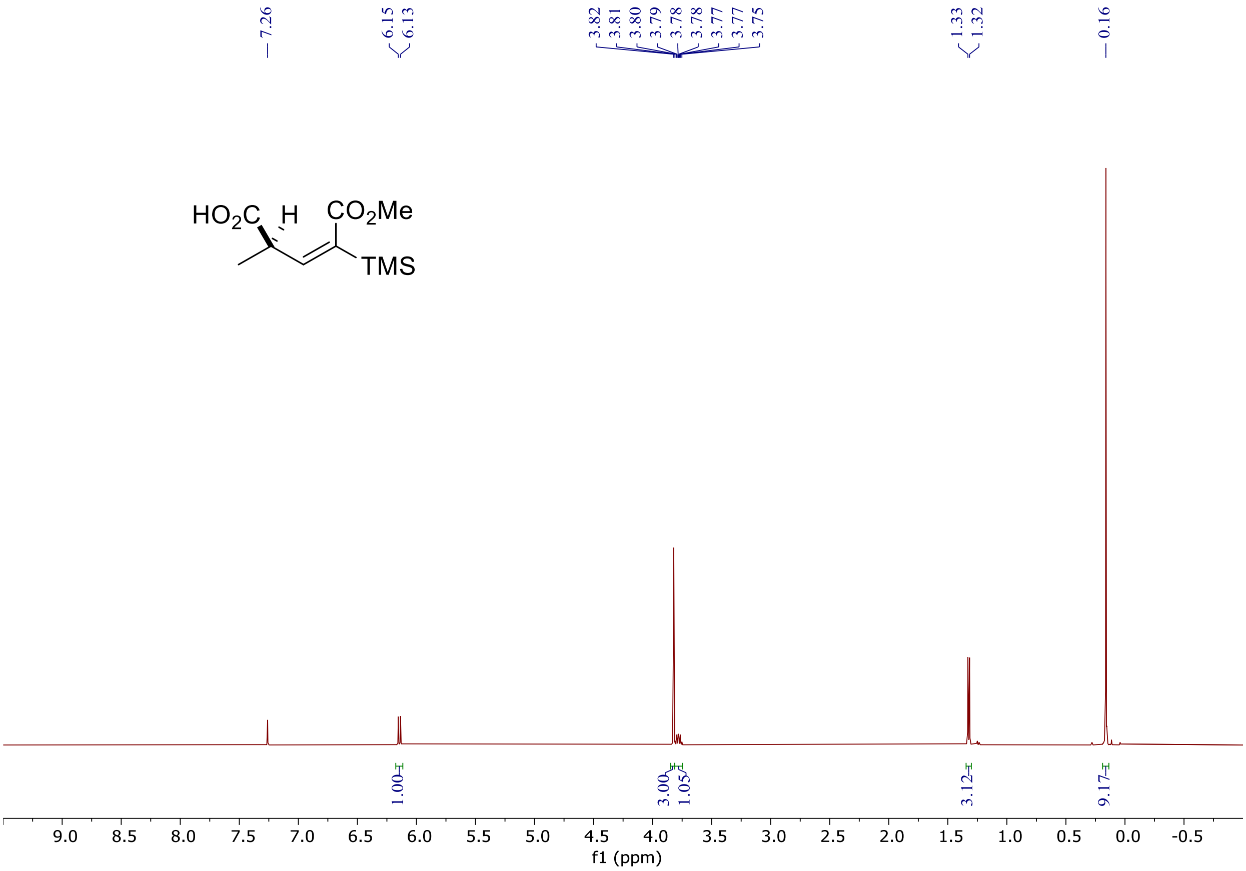

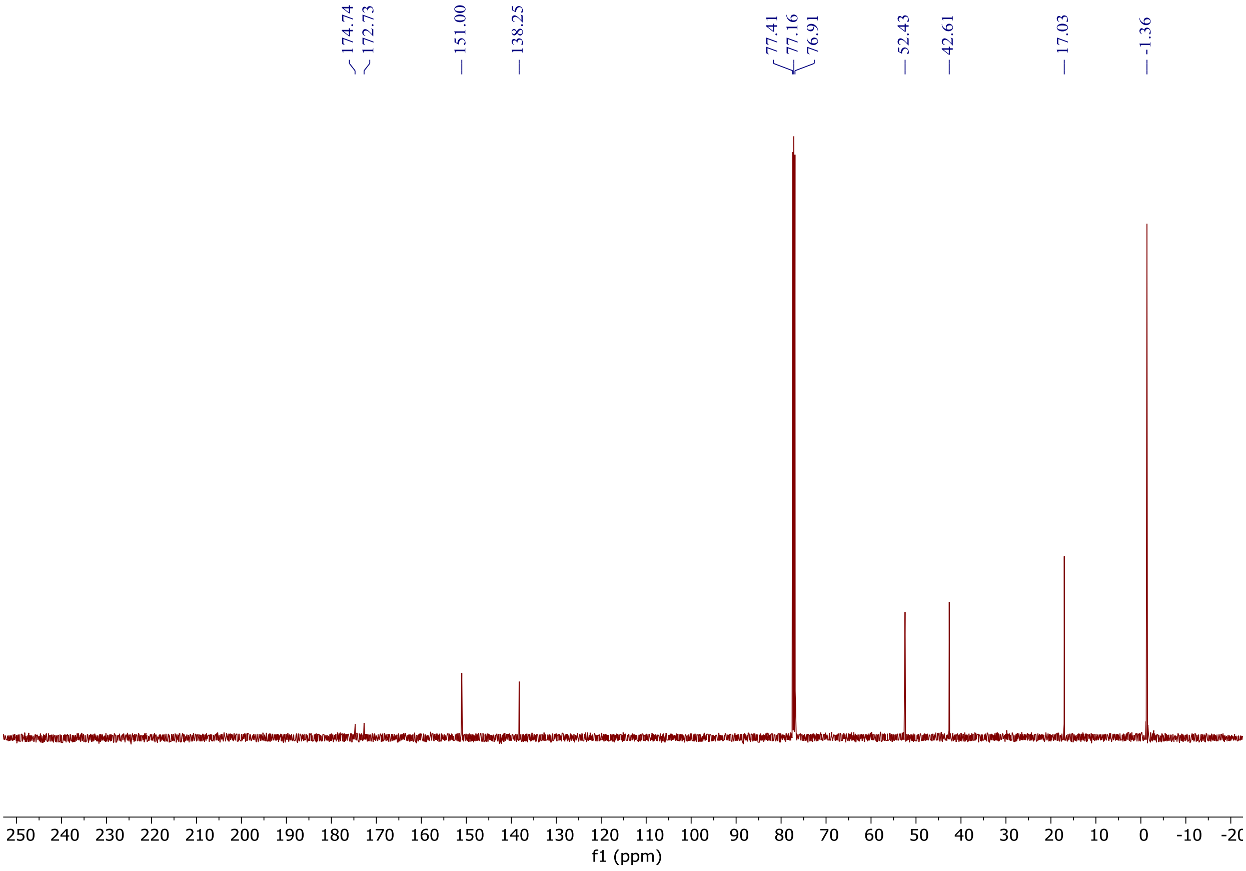

^1H and ^{13}C NMR traces of **4q**

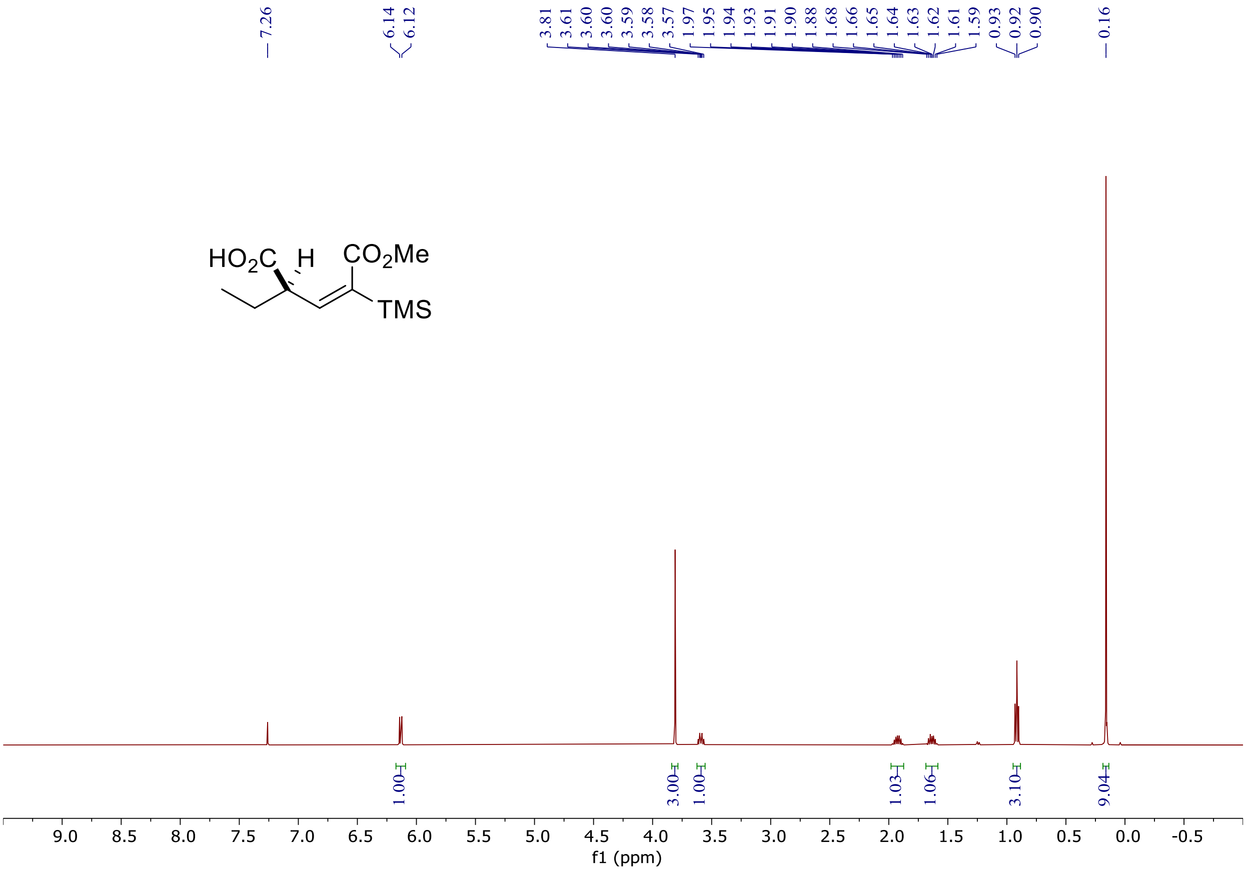

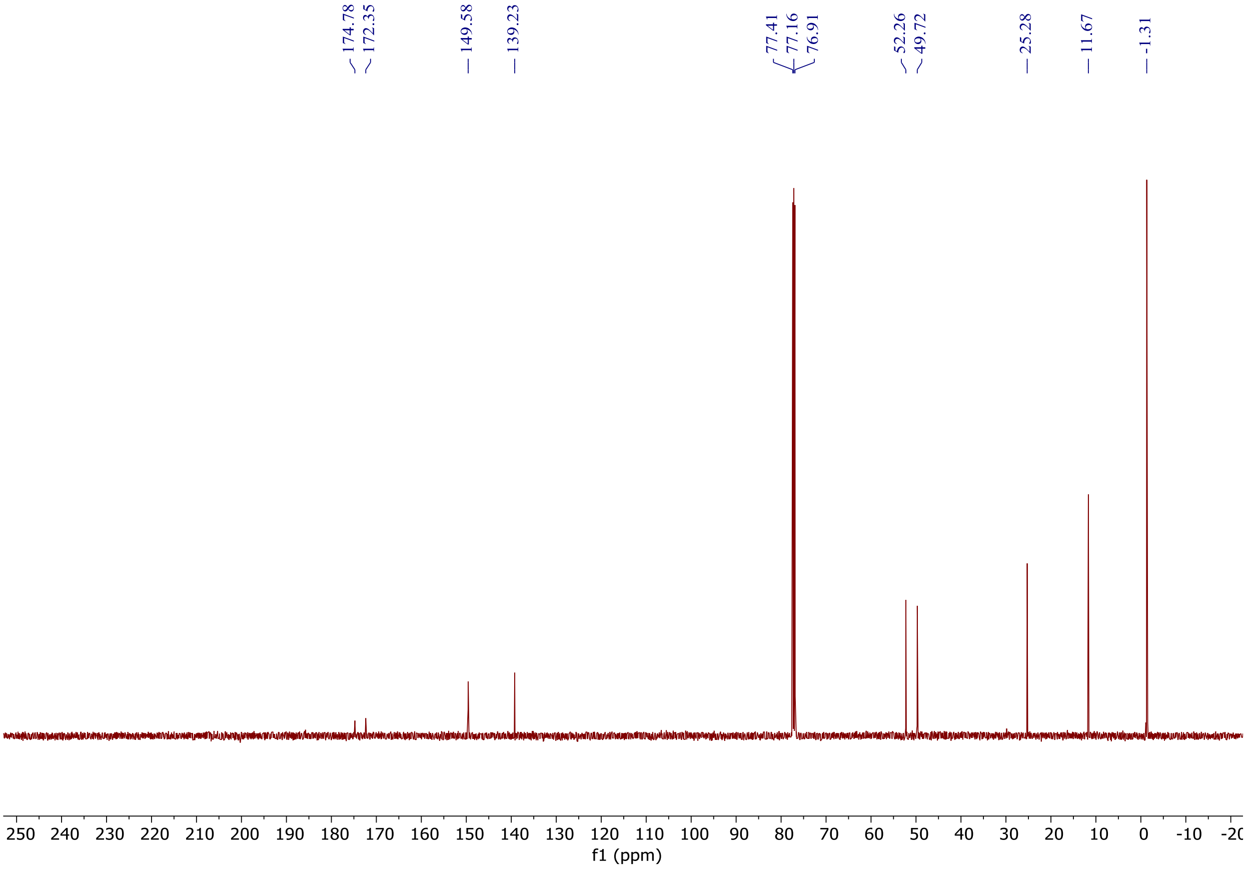

^{1}H and ^{13}C NMR traces of **4r**

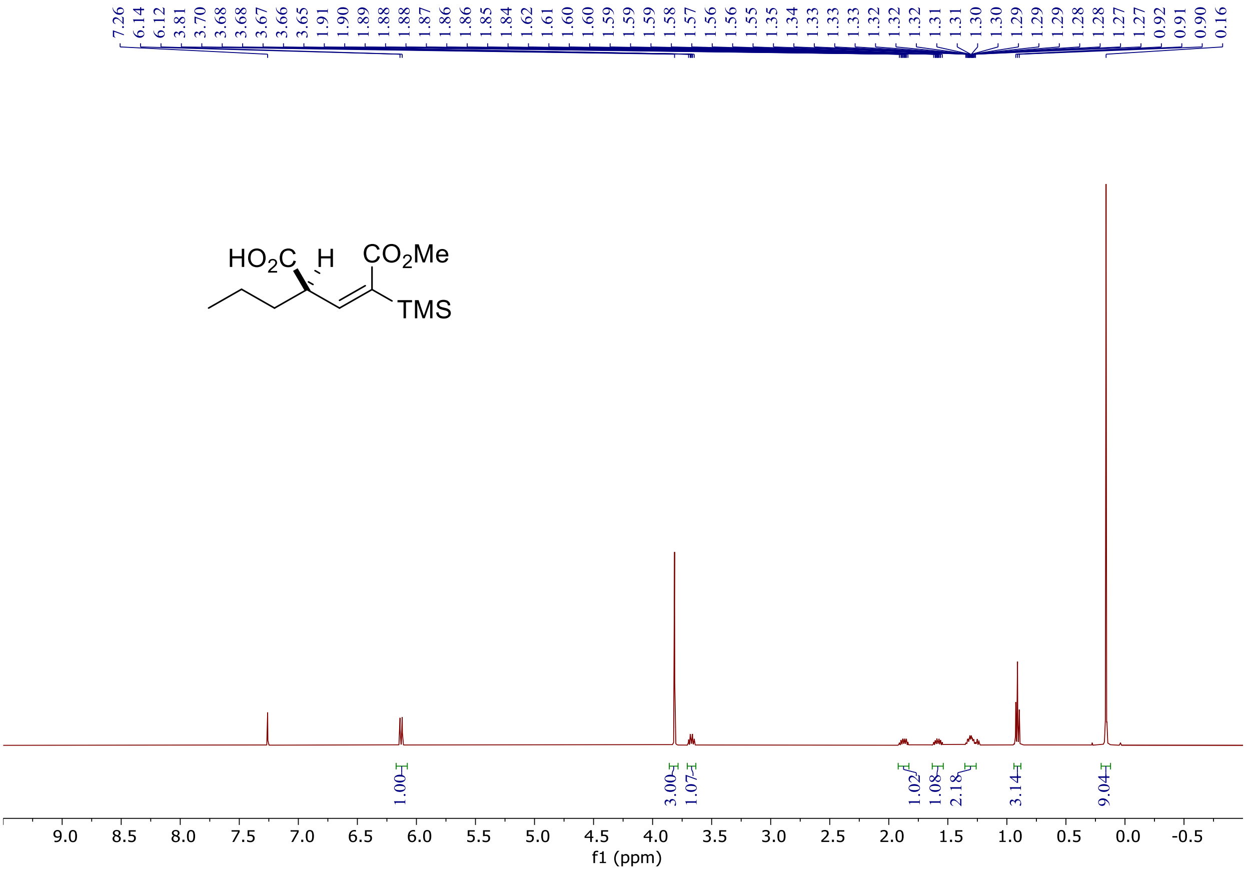

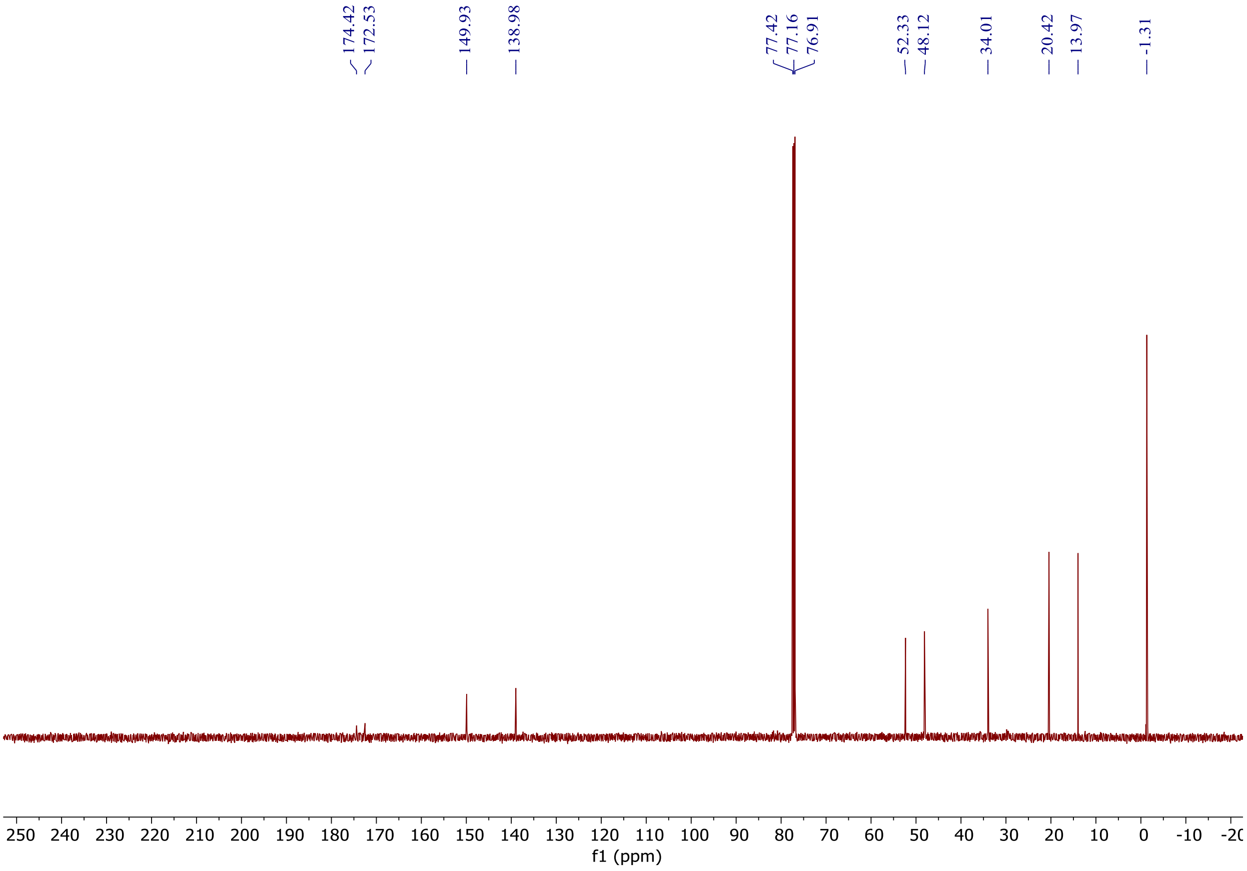

^{1}H and ^{13}C NMR traces of **4s**

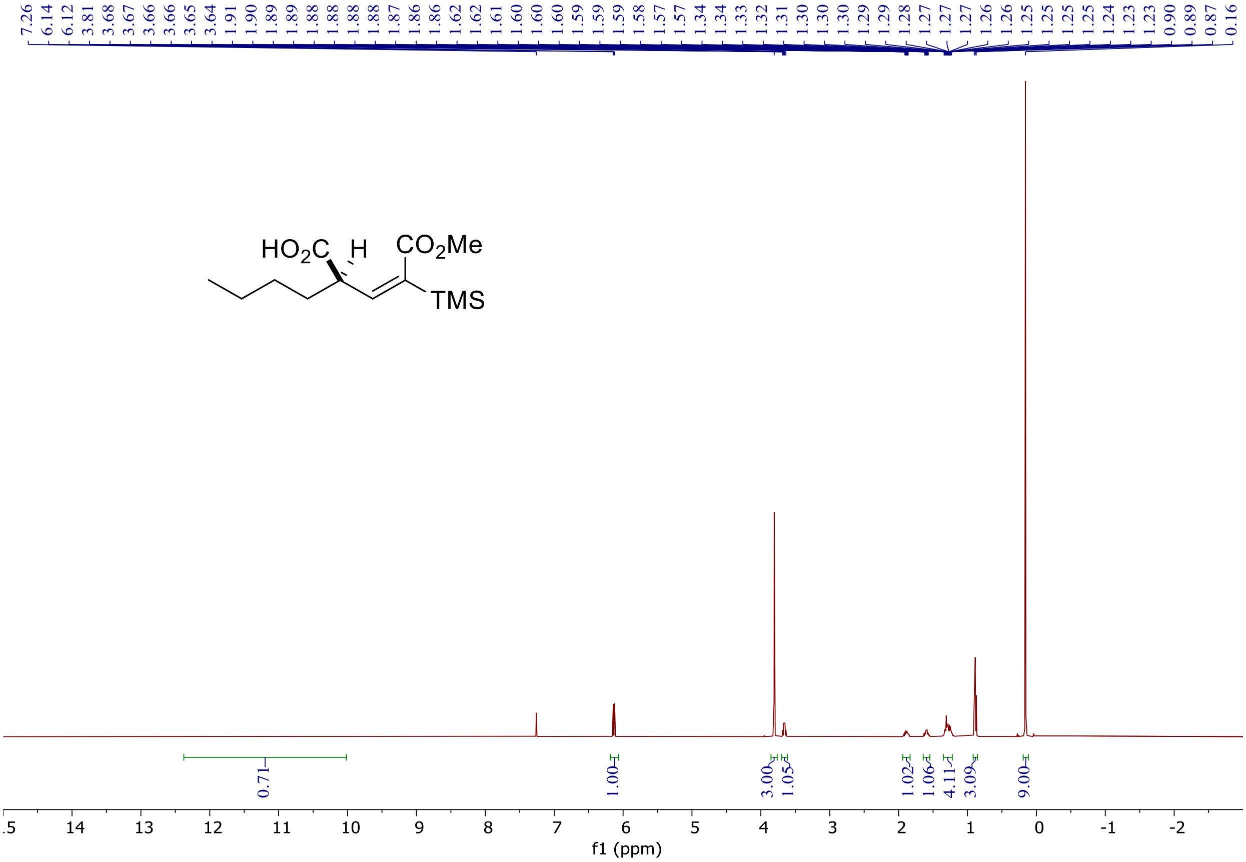

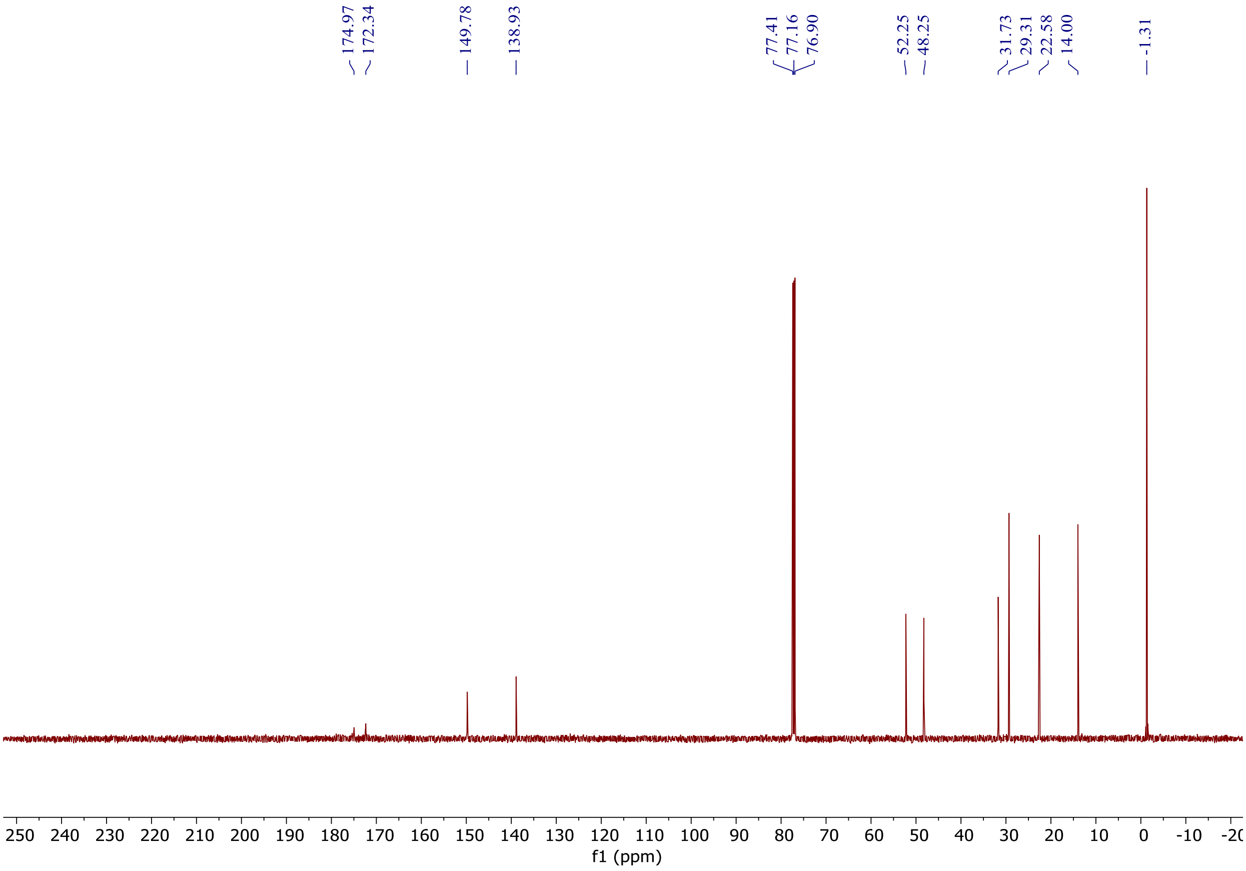

^{1}H and ^{13}C NMR traces of **4t**

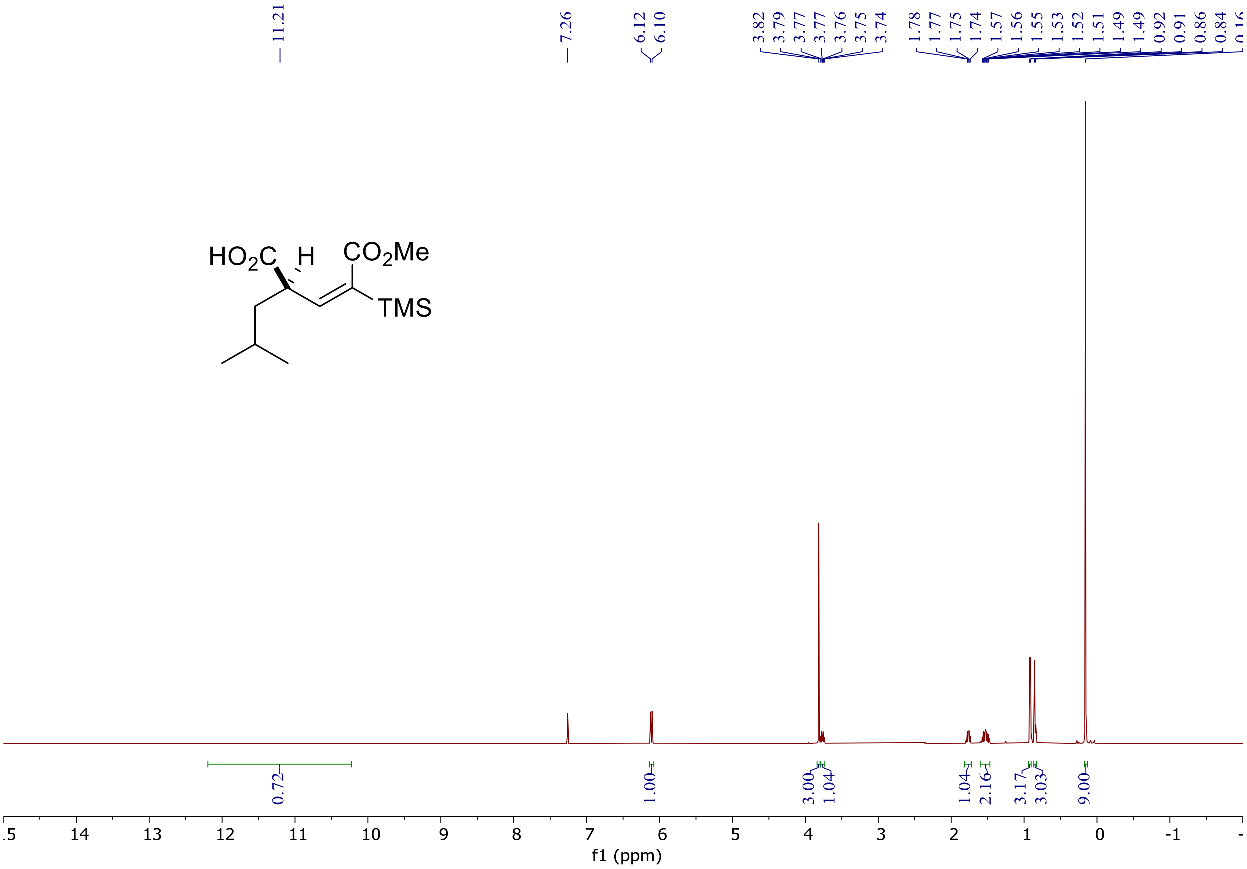

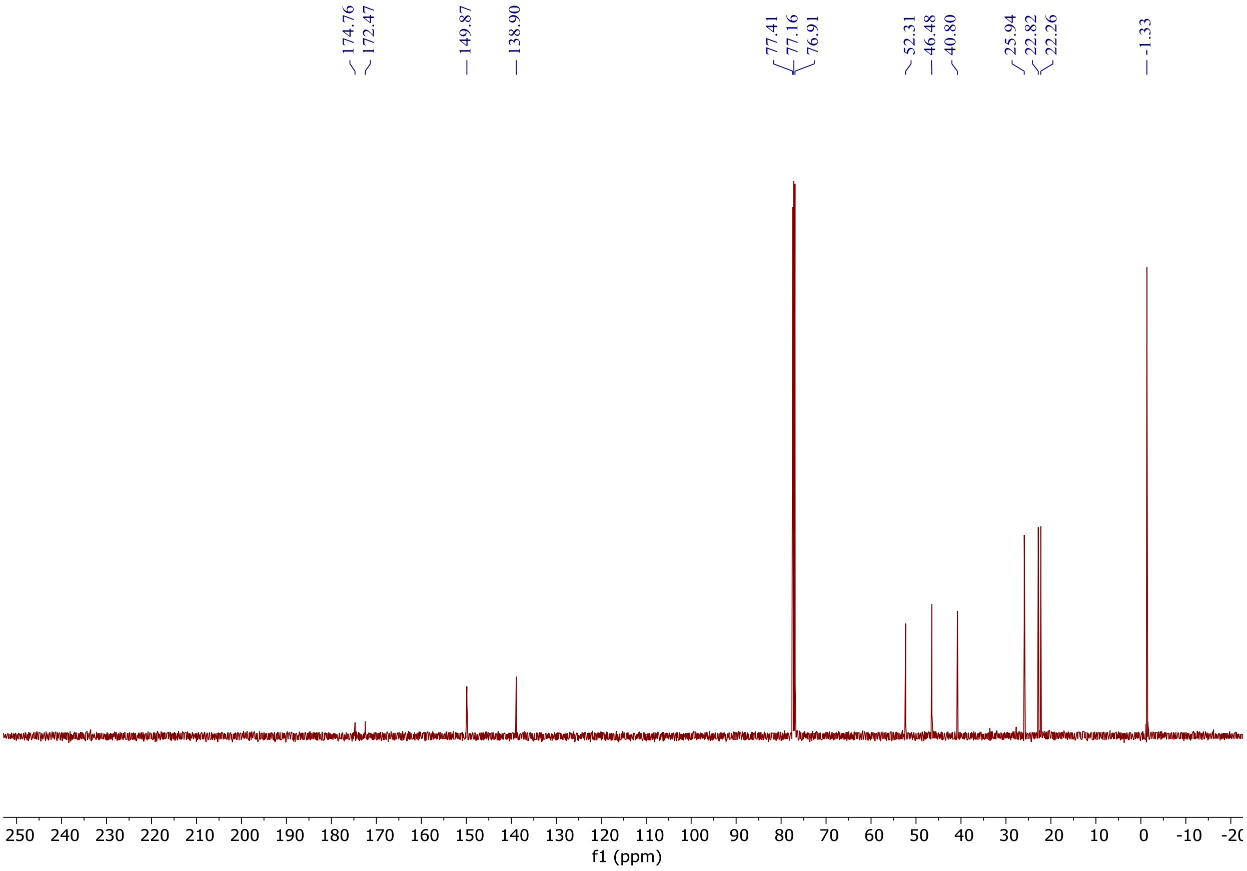

^{1}H and ^{13}C NMR traces of **4u**

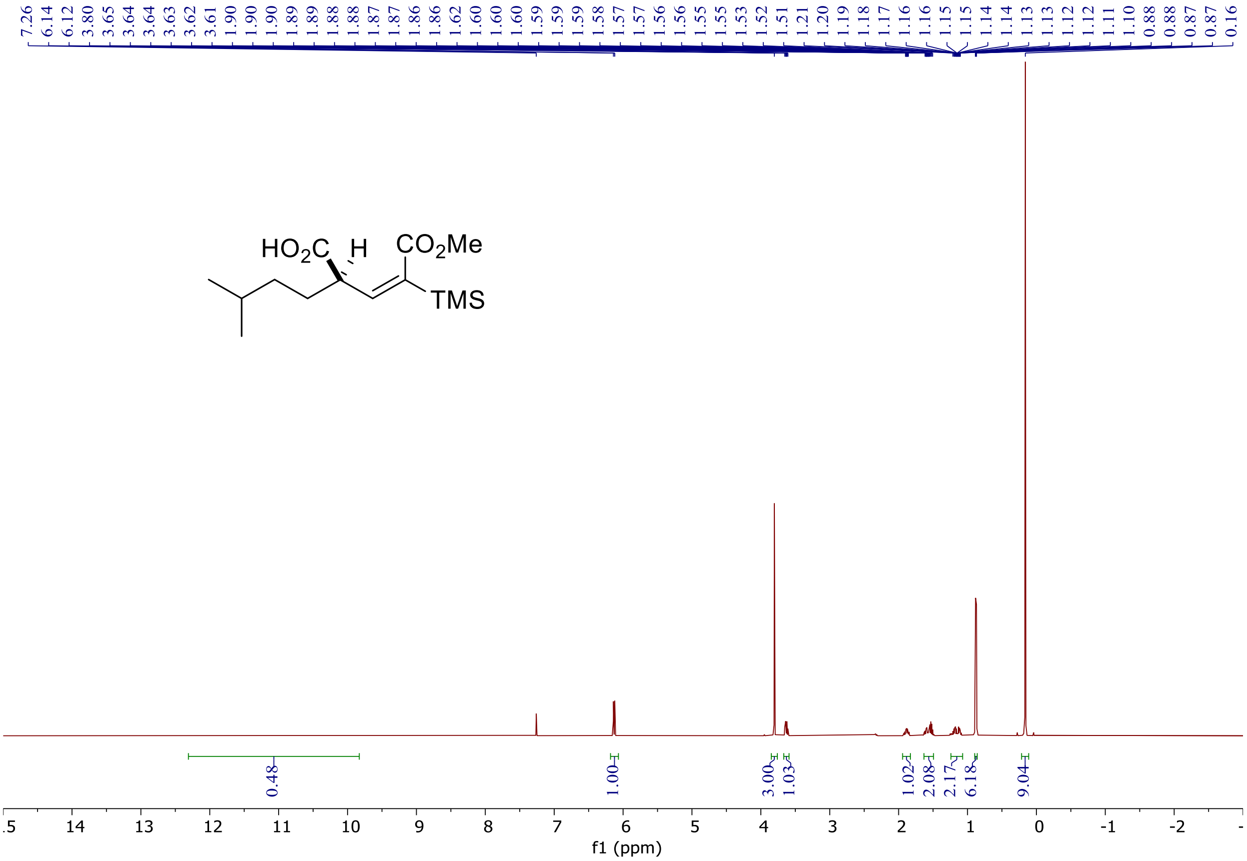

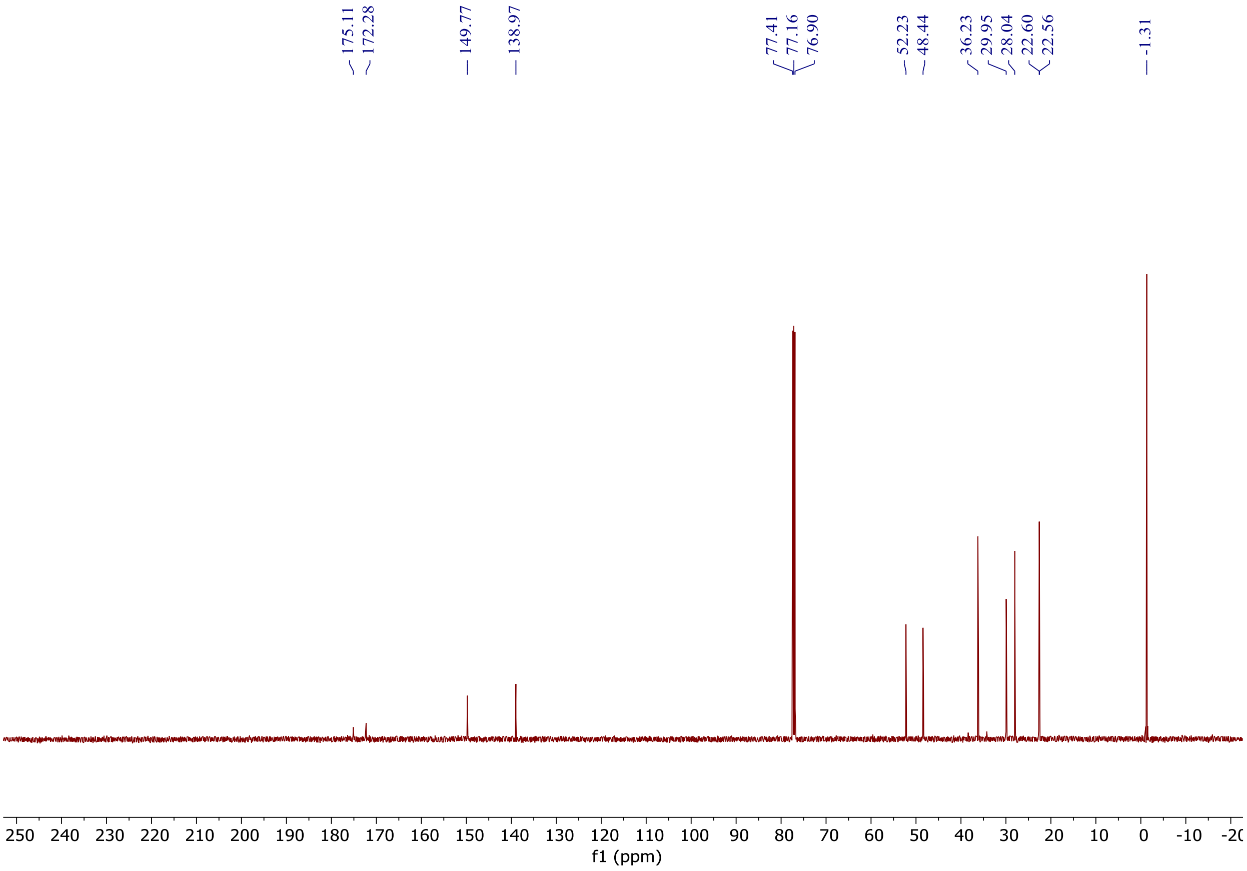

^{1}H and ^{13}C NMR traces of **4v**

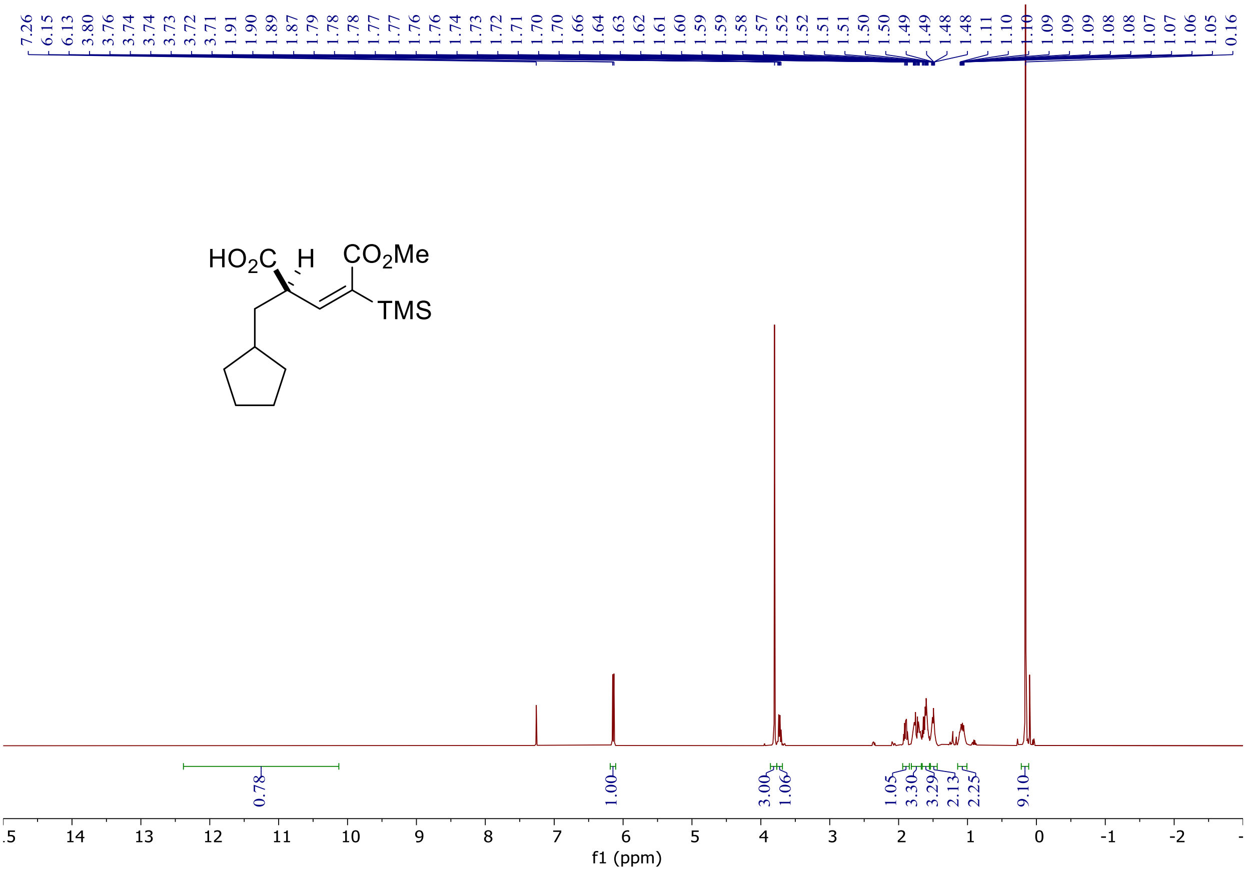

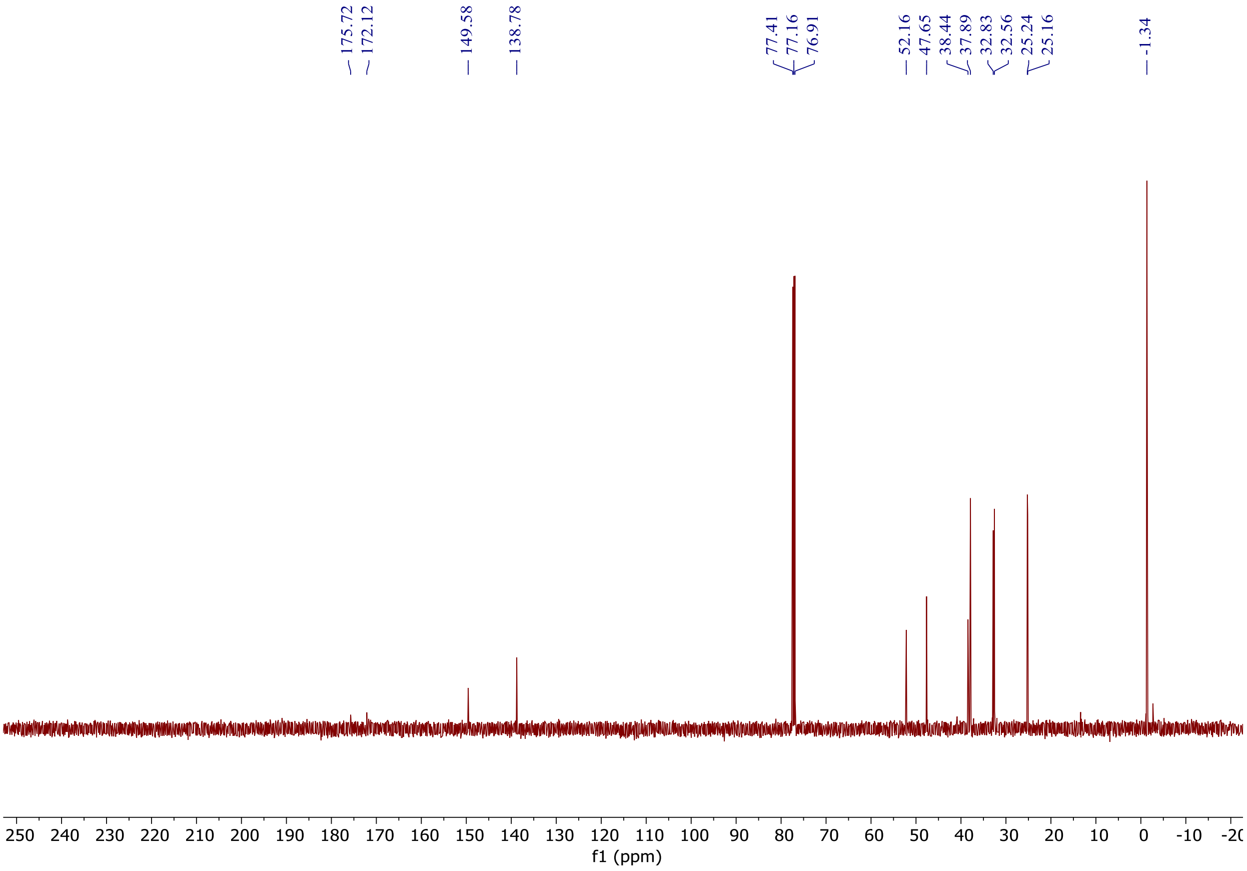

^{1}H and ^{13}C NMR traces of **4w**

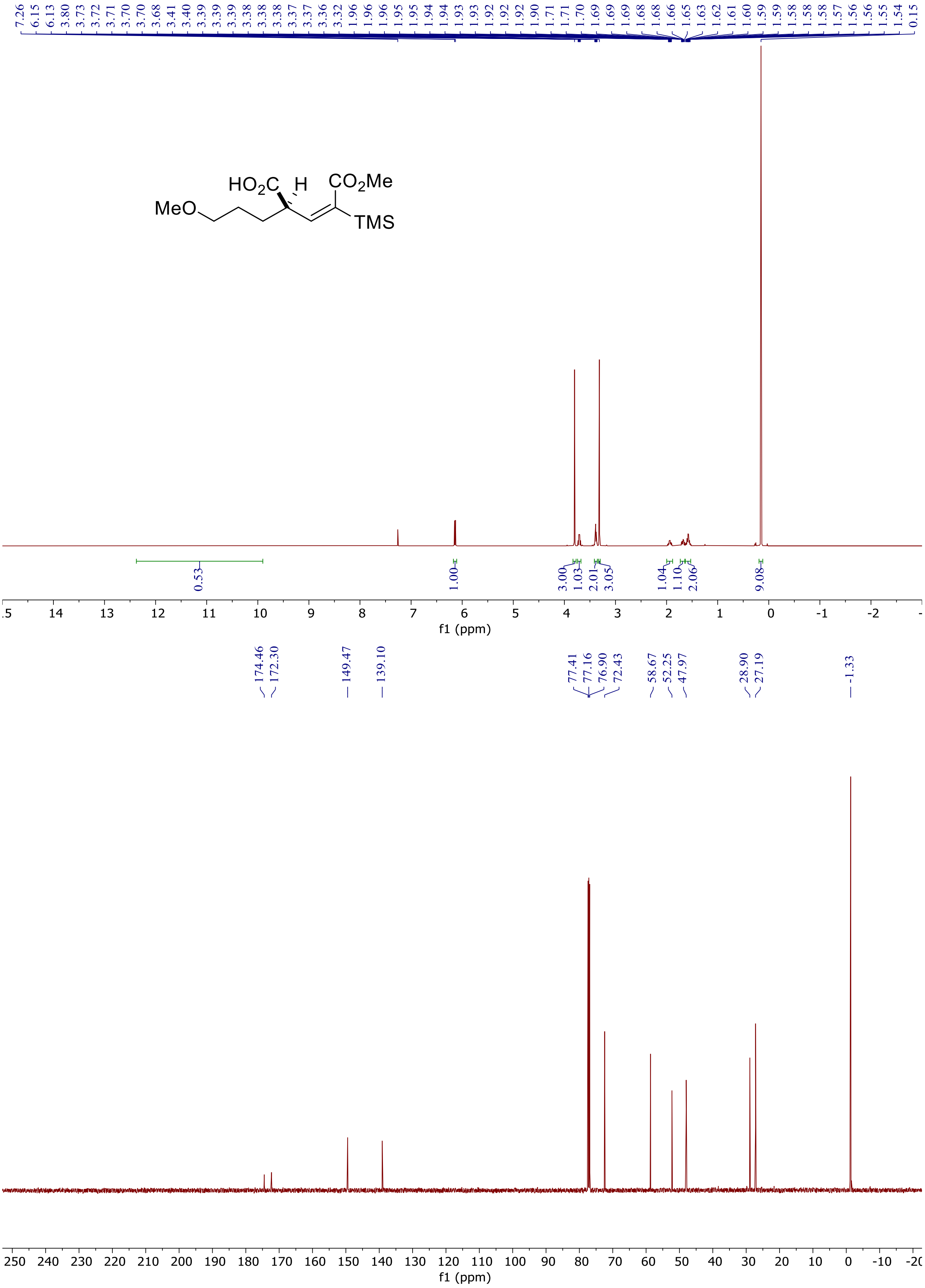

^{1}H and ^{13}C NMR traces of **4x**

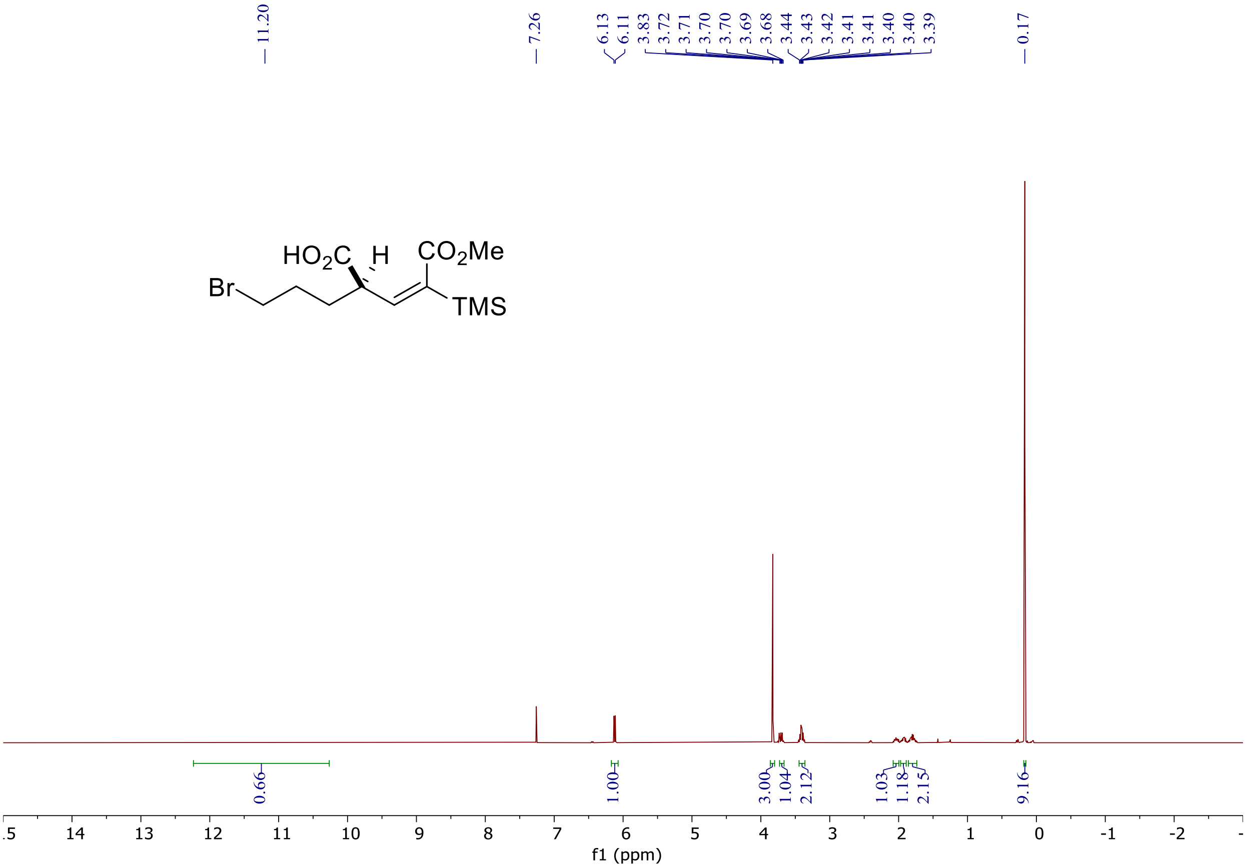

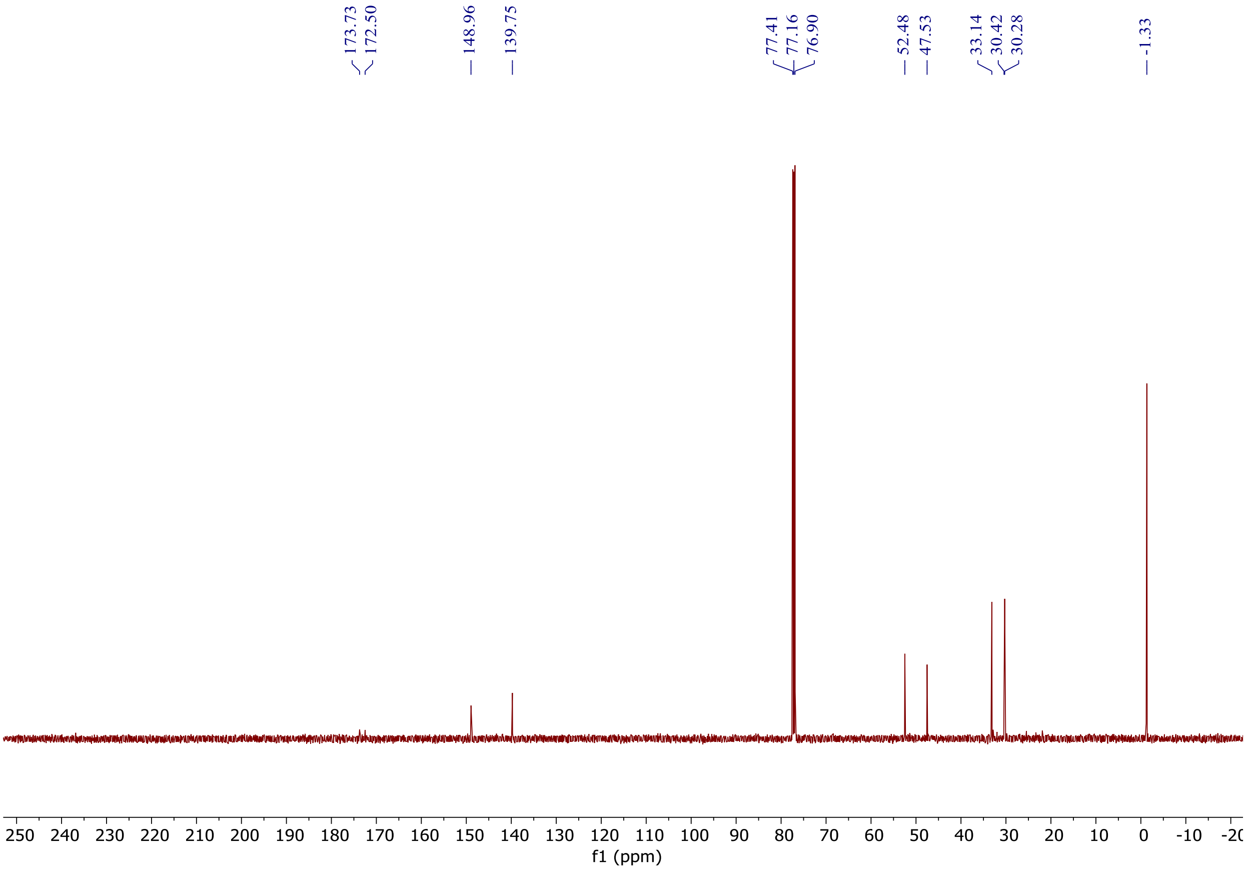

^{1}H and ^{13}C NMR traces of **4y**

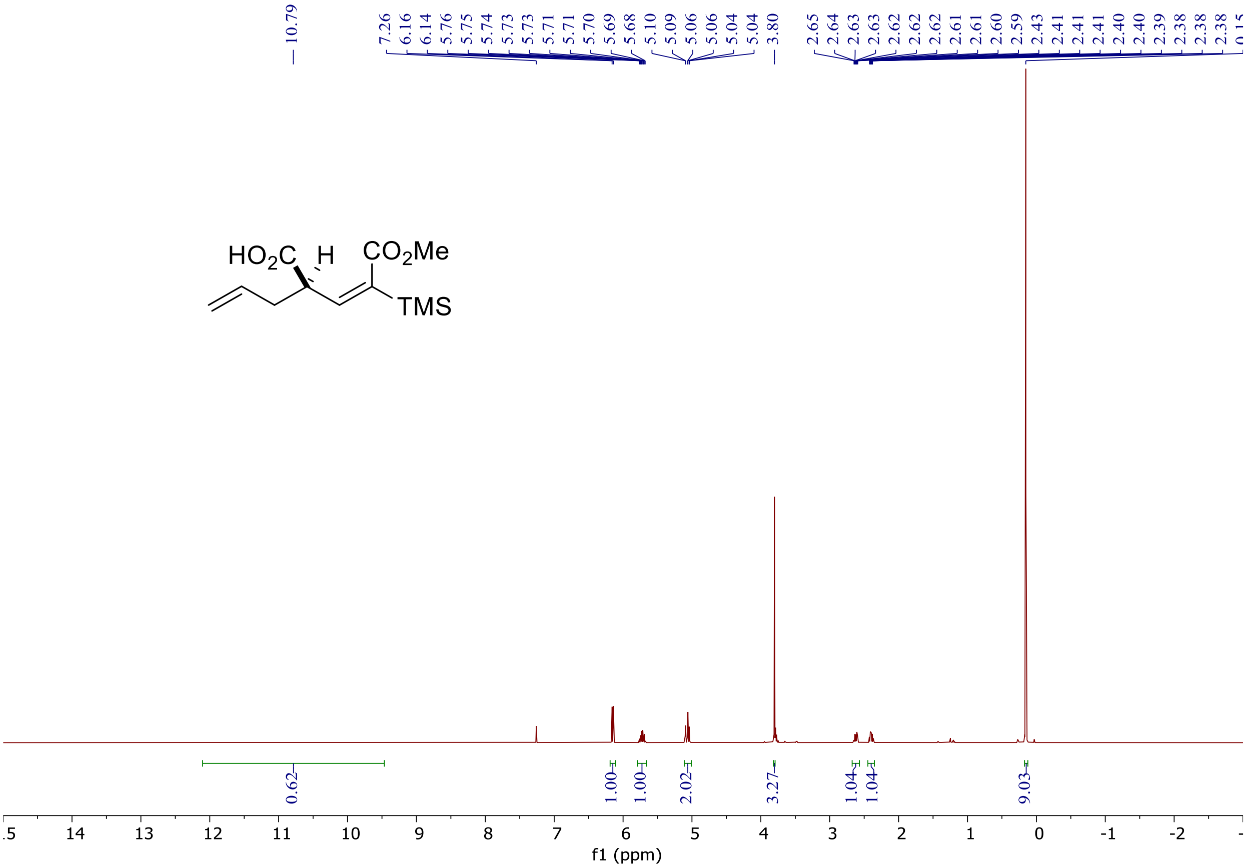

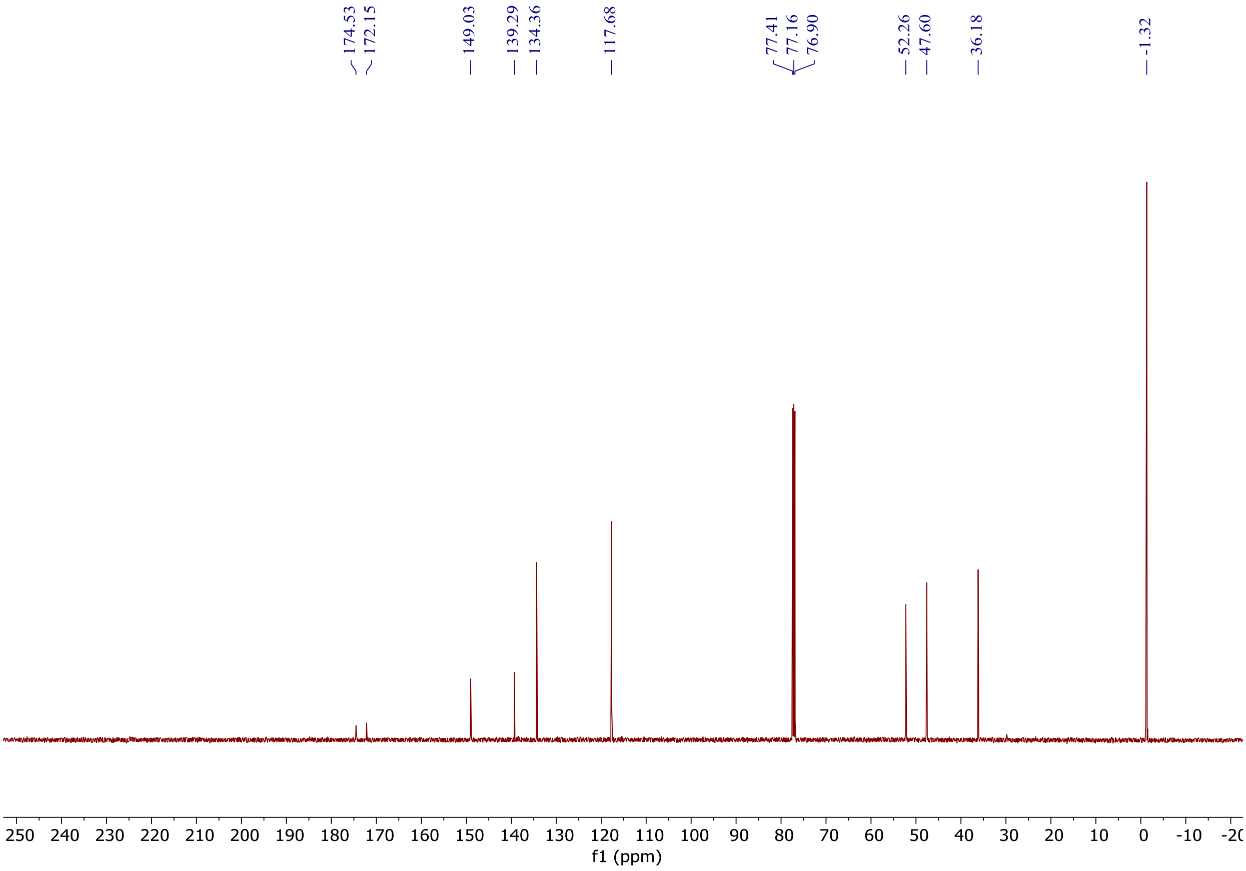

^{1}H and ^{13}C NMR traces of **4za**

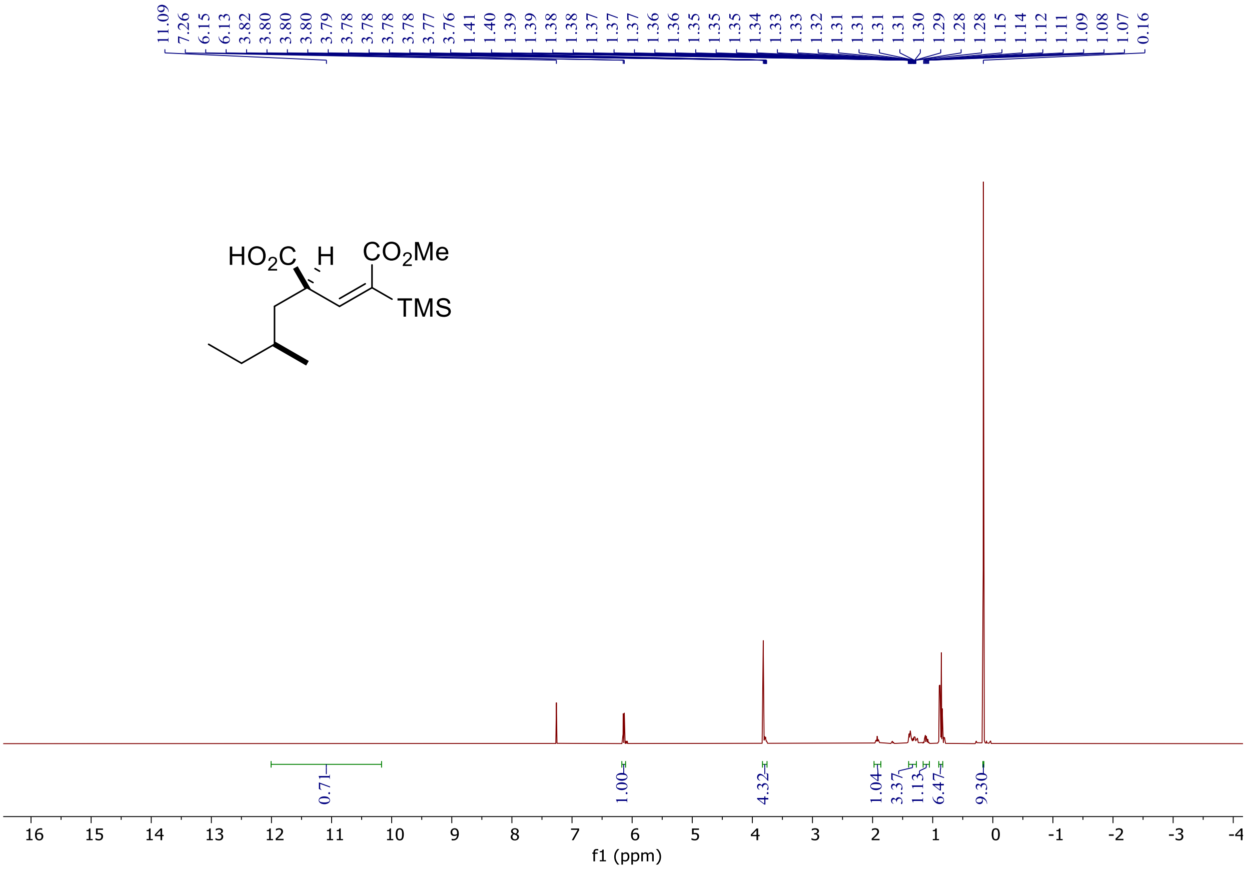

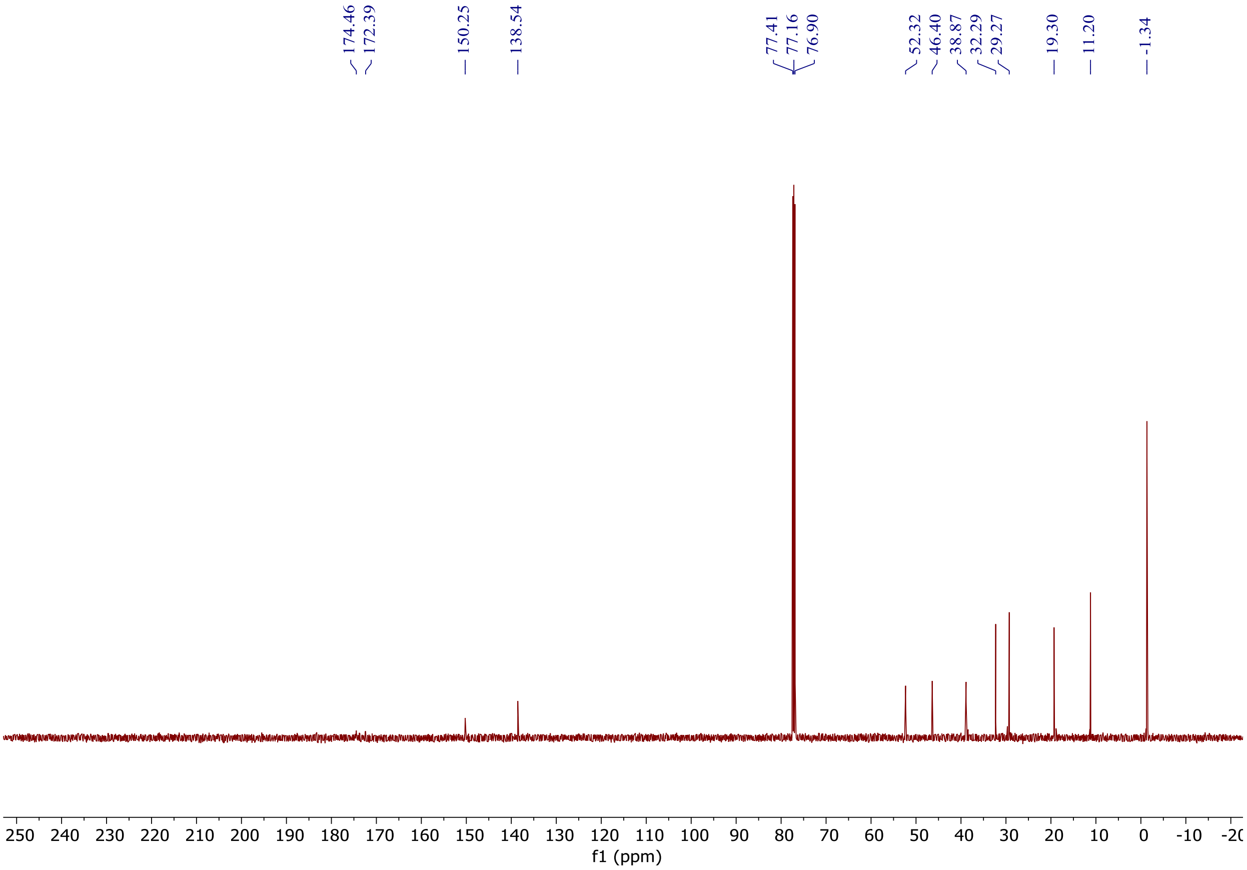

^{1}H and ^{13}C NMR traces of **4zb**

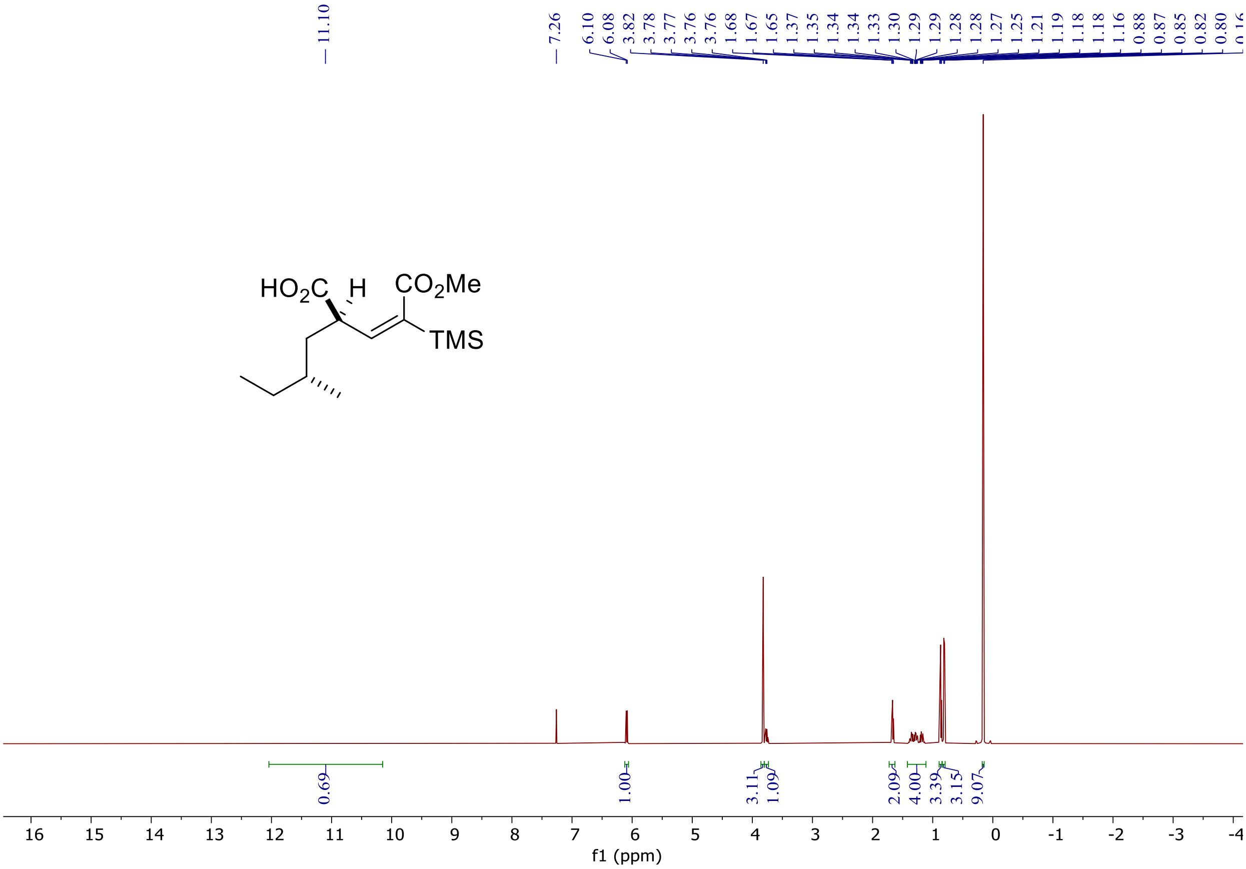

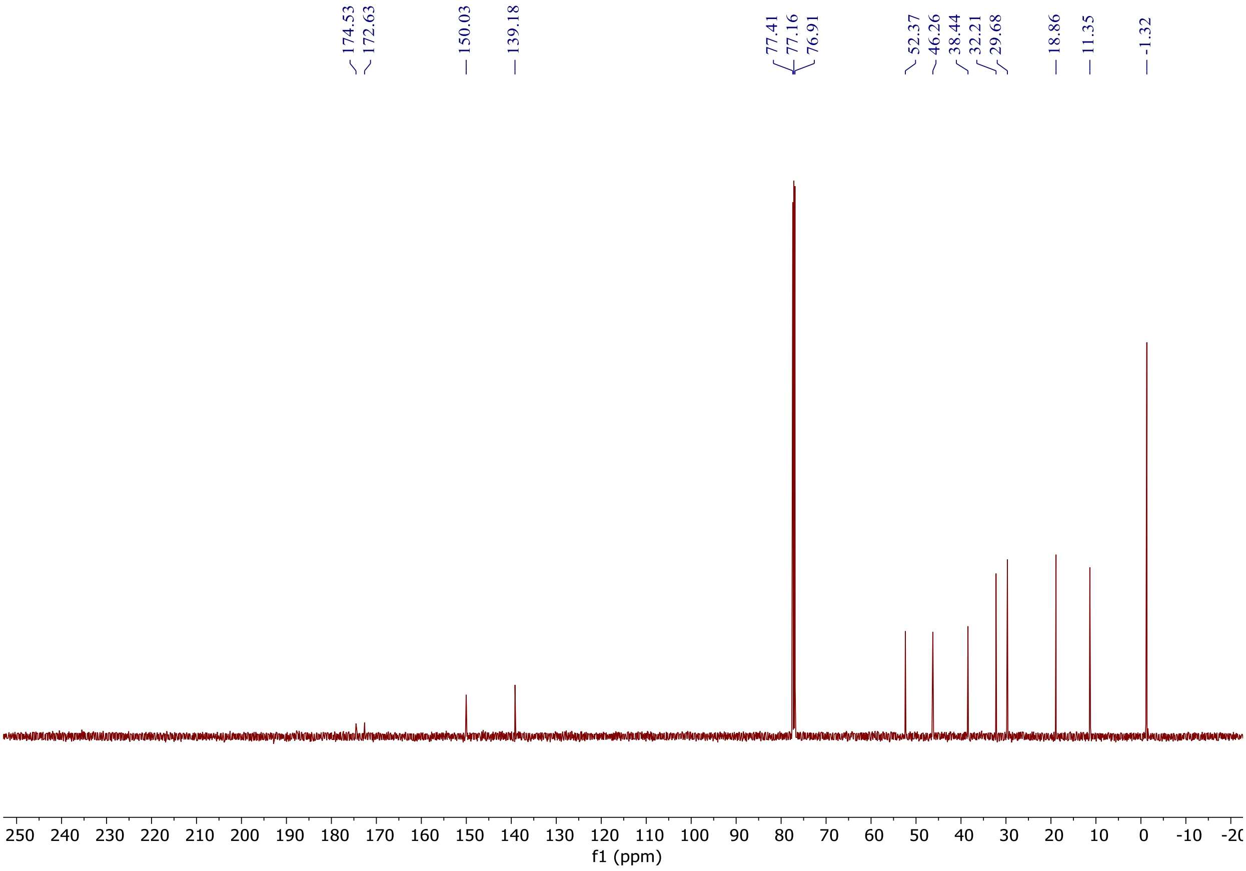

^{1}H and ^{13}C NMR traces of **5a**

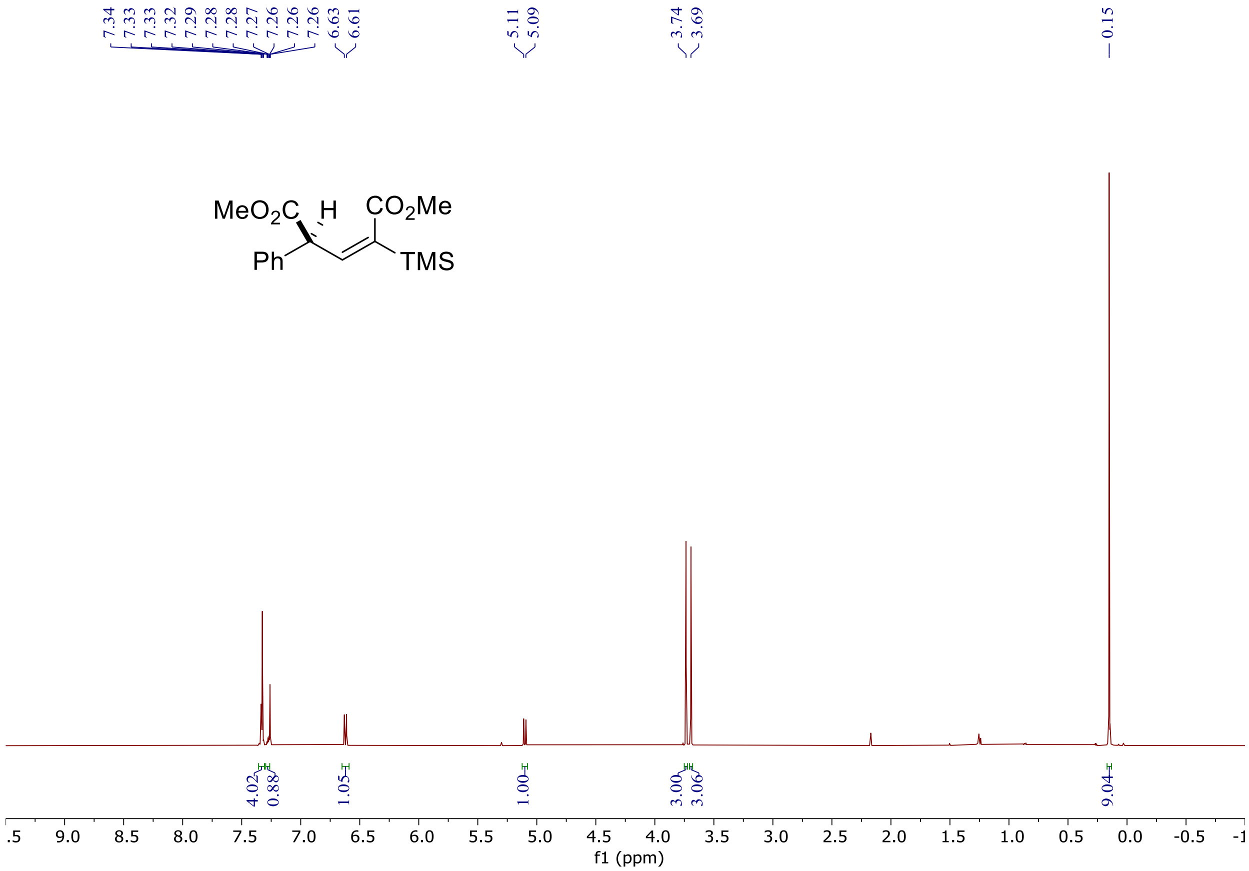

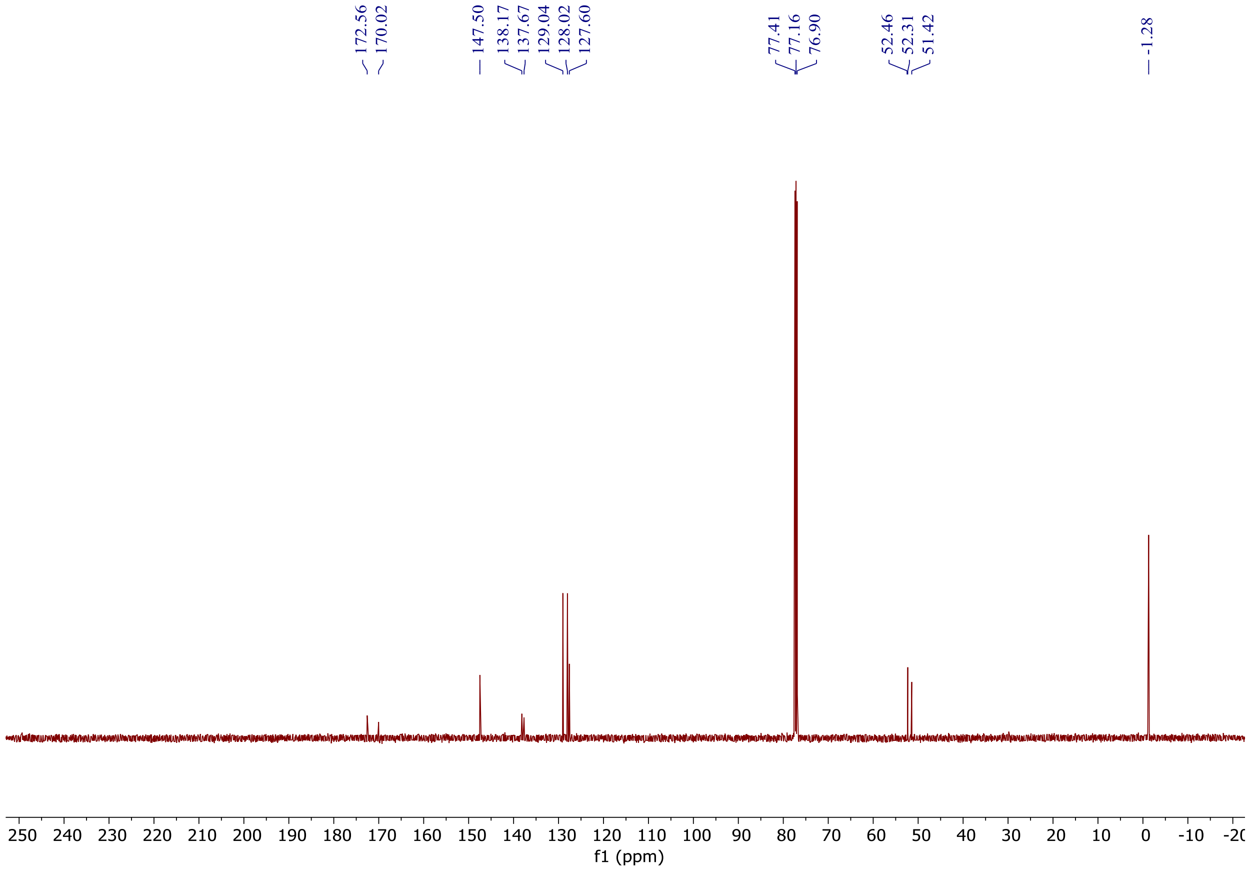

^{1}H and ^{13}C NMR traces of **5b**

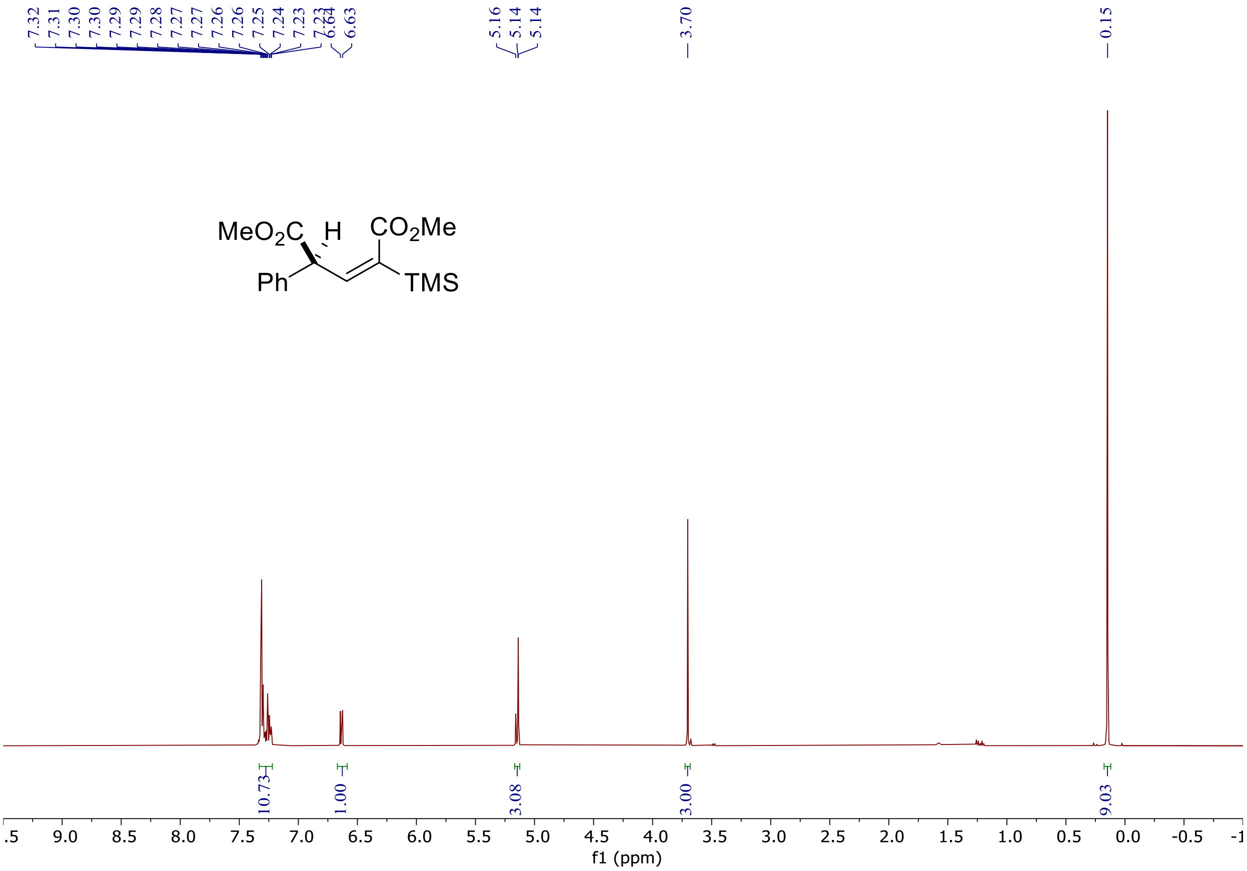

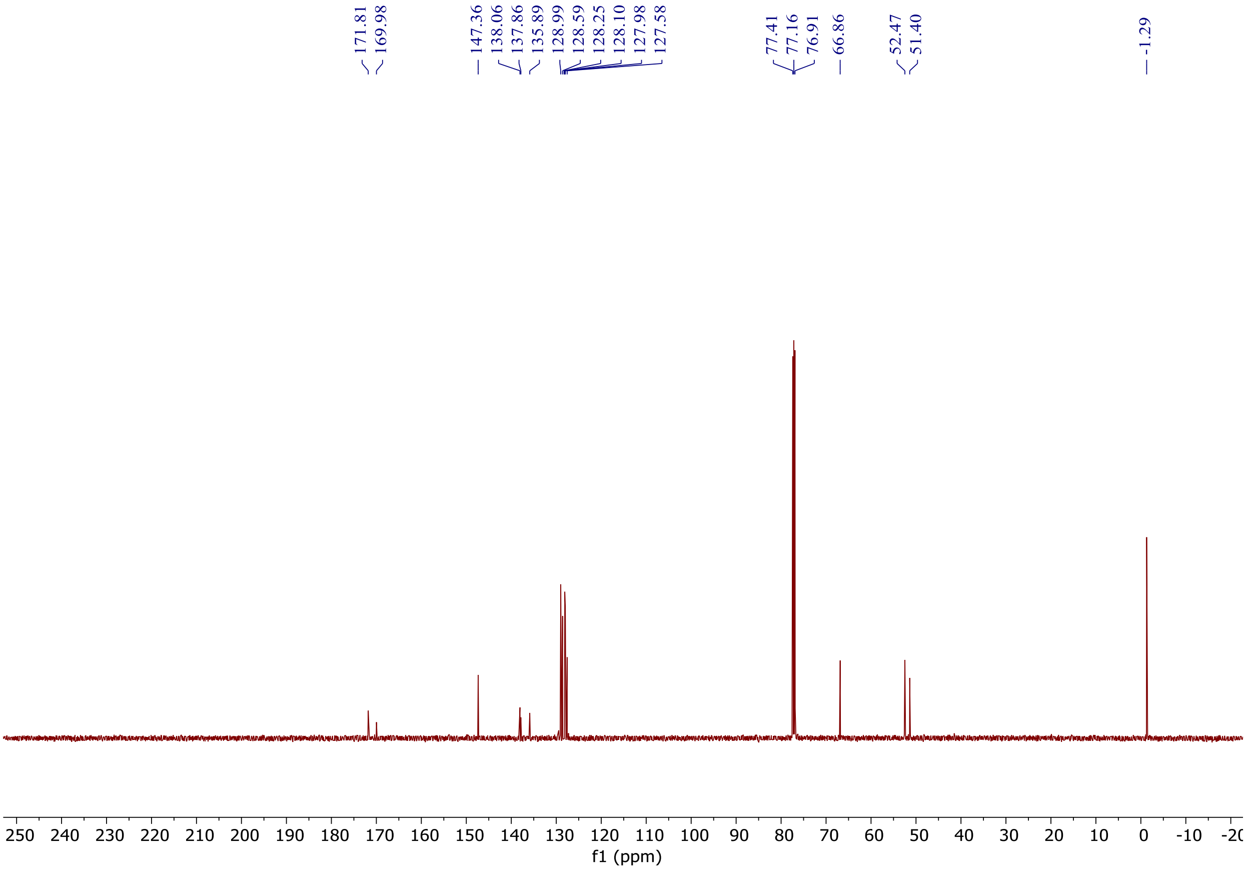

^1H and ^{13}C NMR traces of **6**

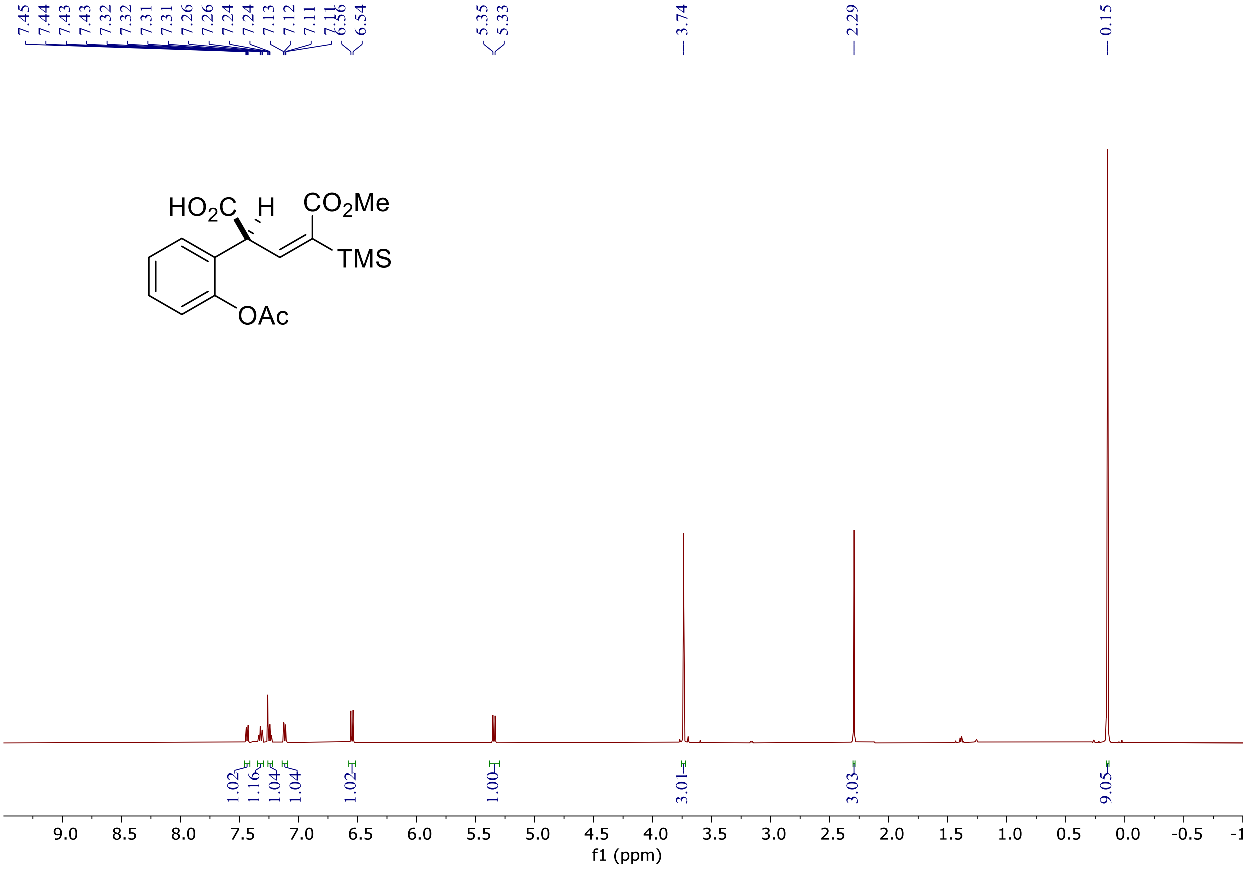

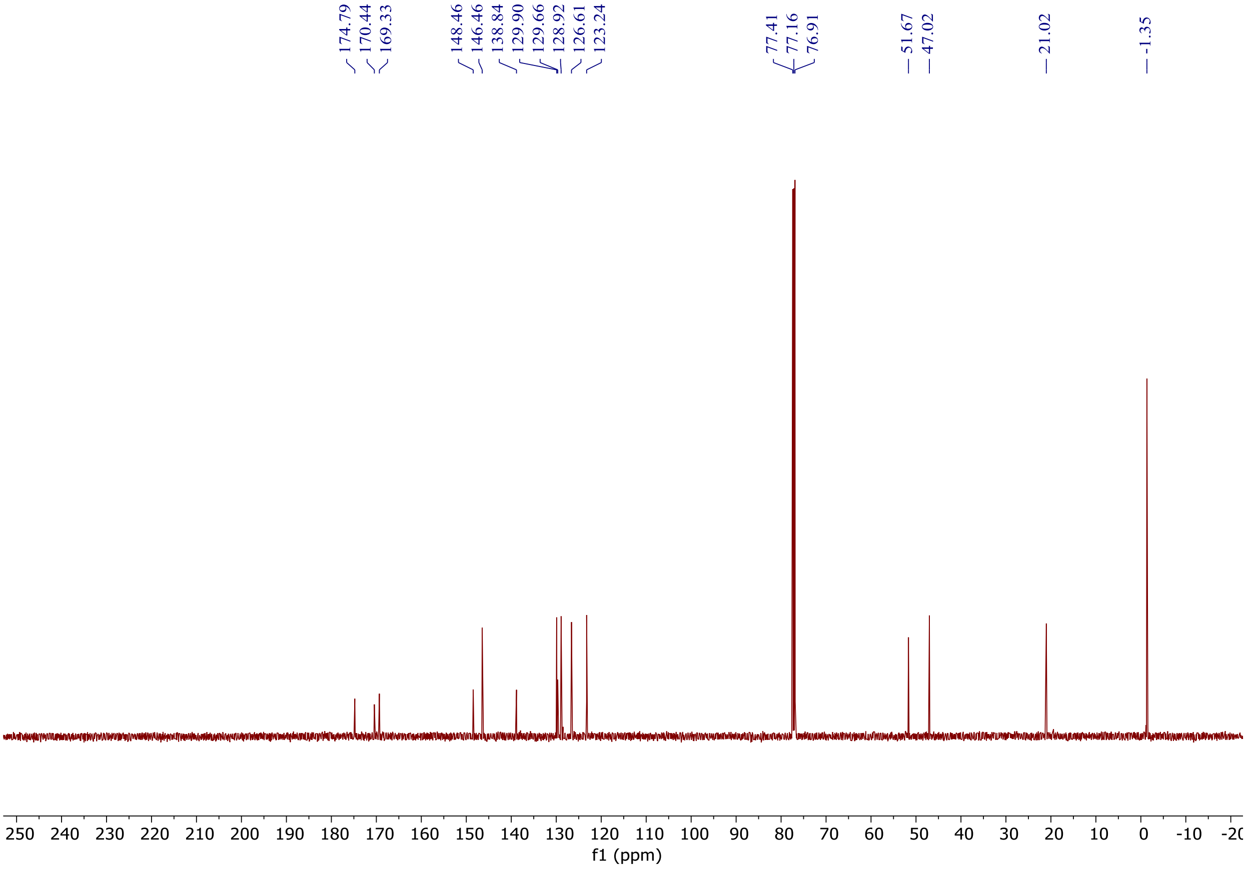

^{1}H and ^{13}C NMR traces of **7a**

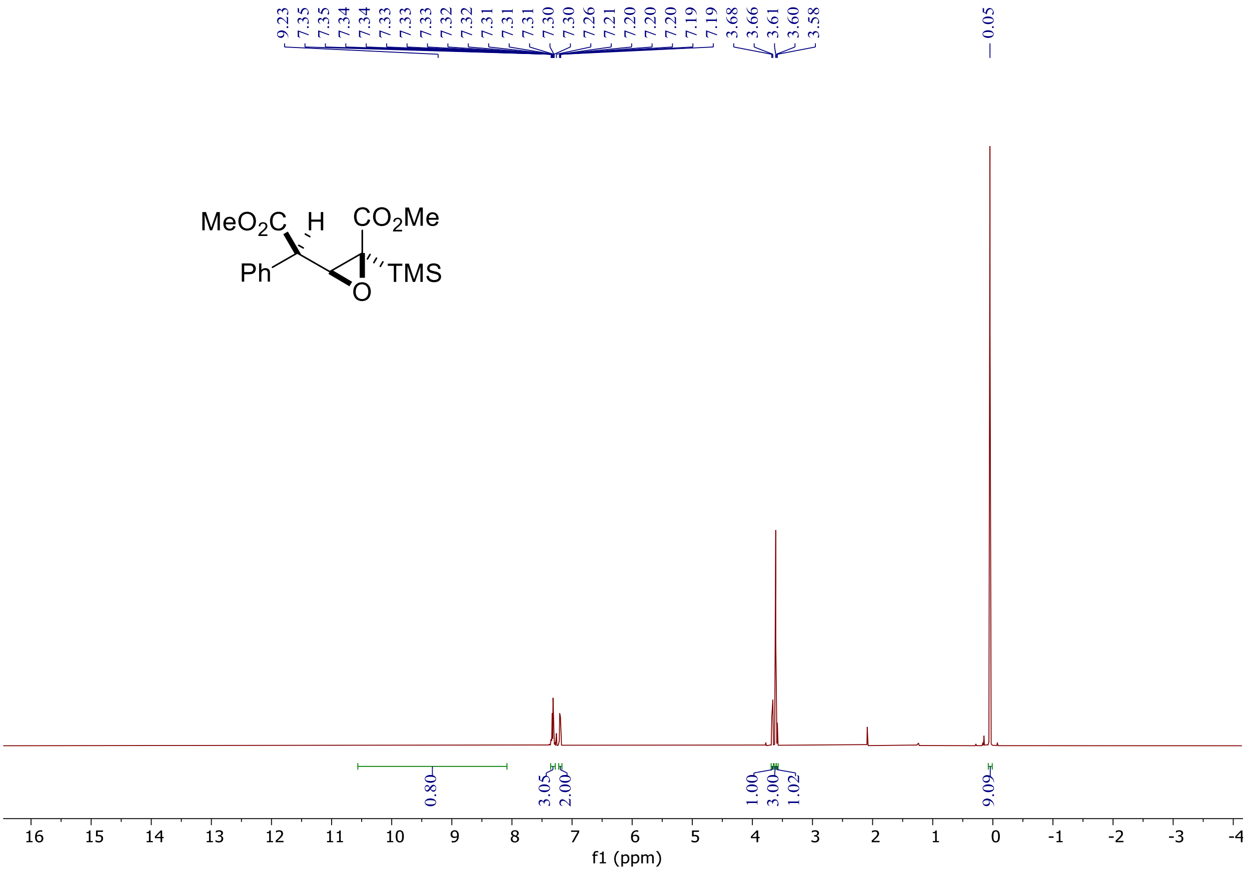

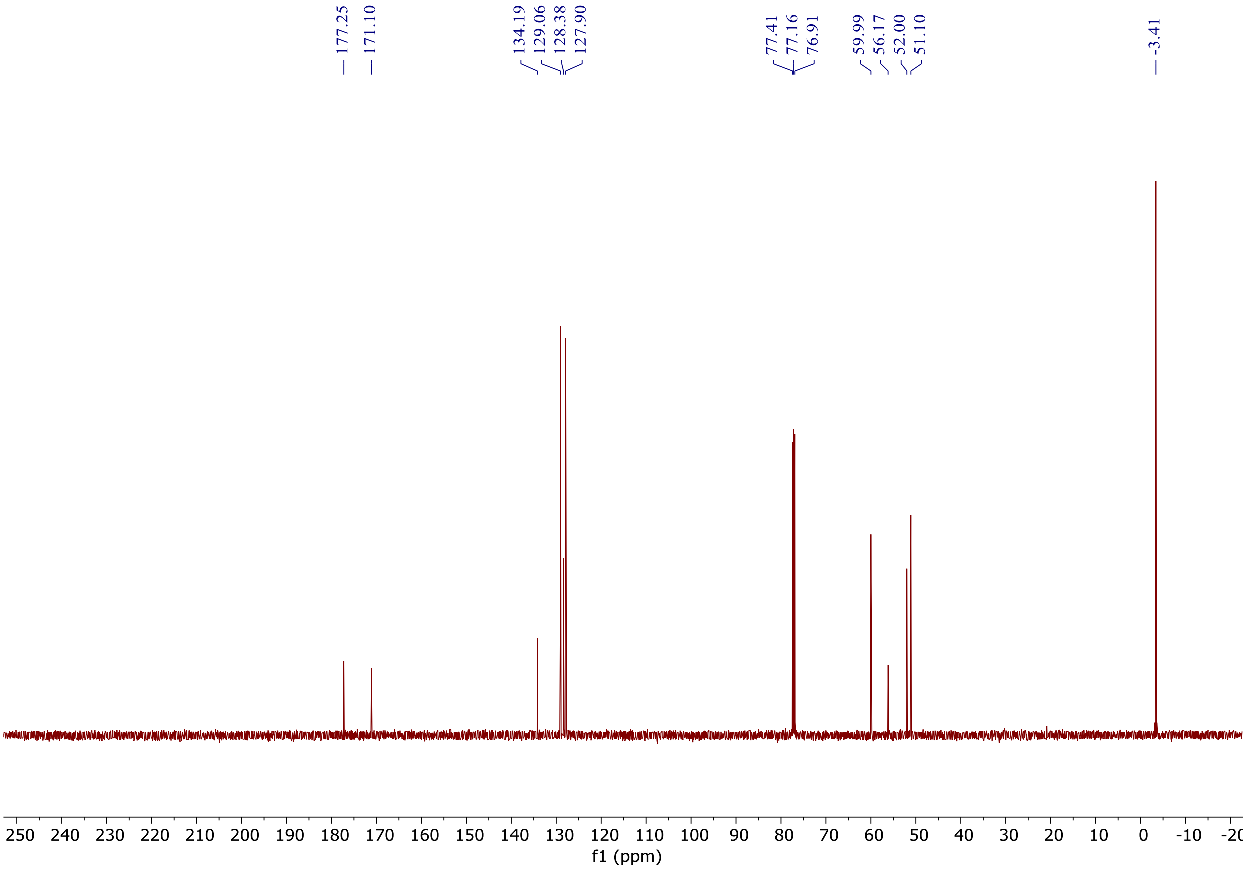

^{1}H and ^{13}C NMR traces of **7b**

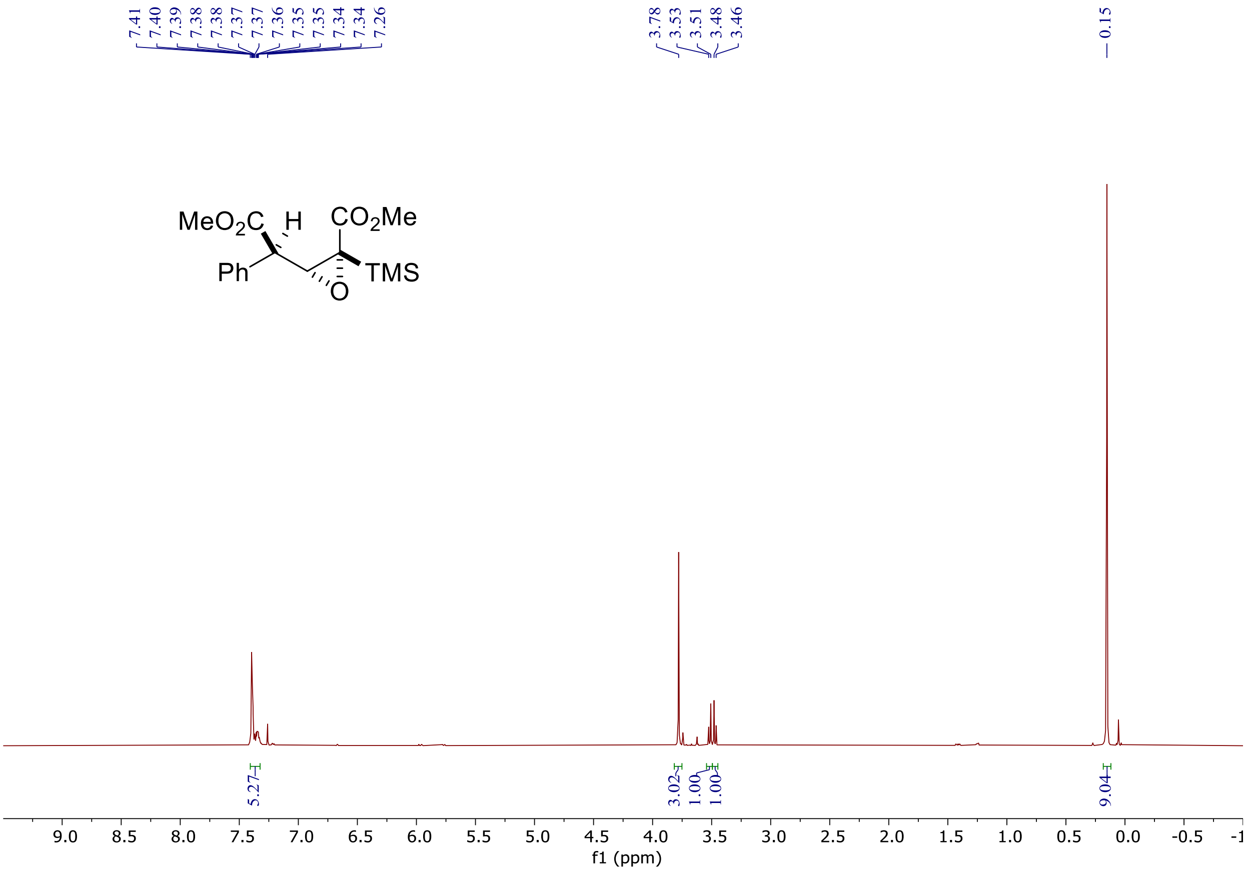

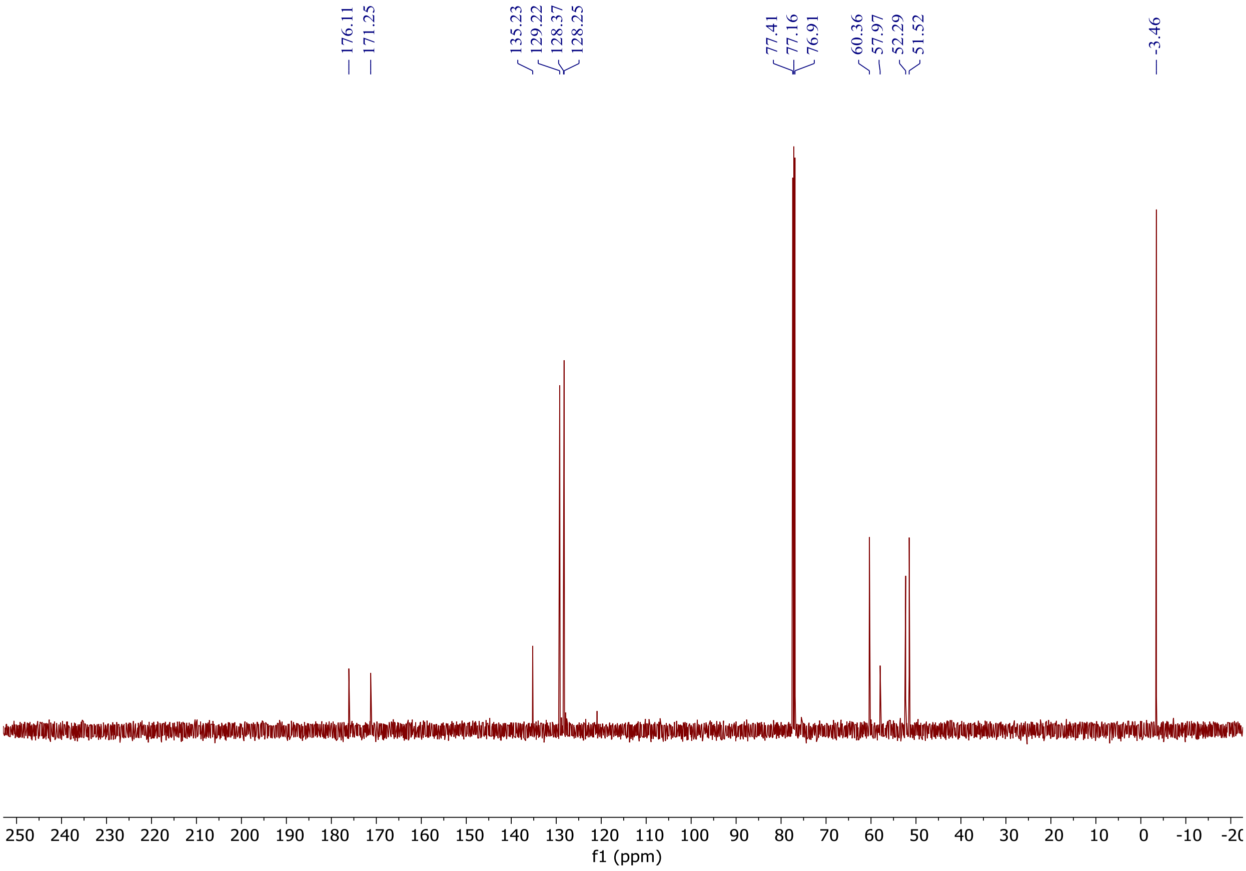

^{1}H and ^{13}C NMR traces of **8**

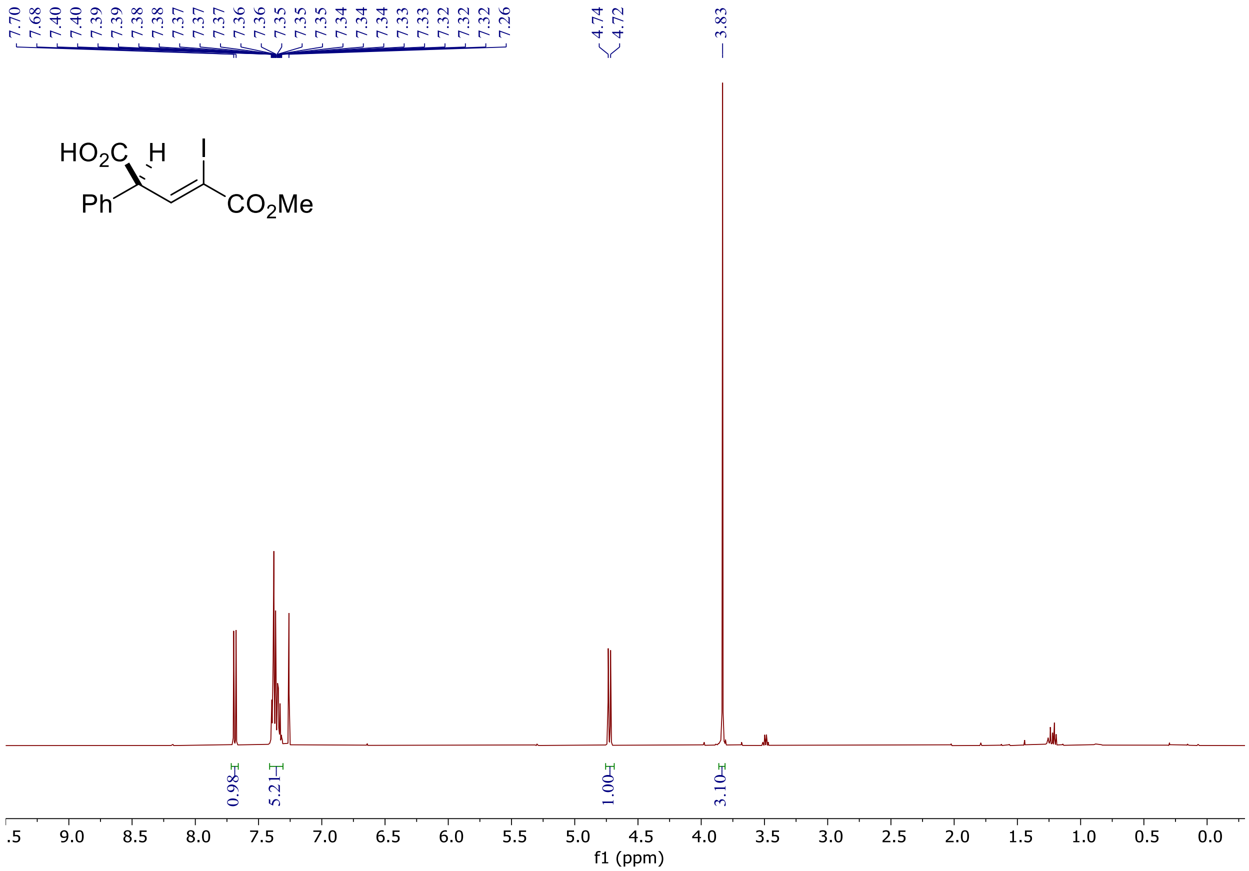

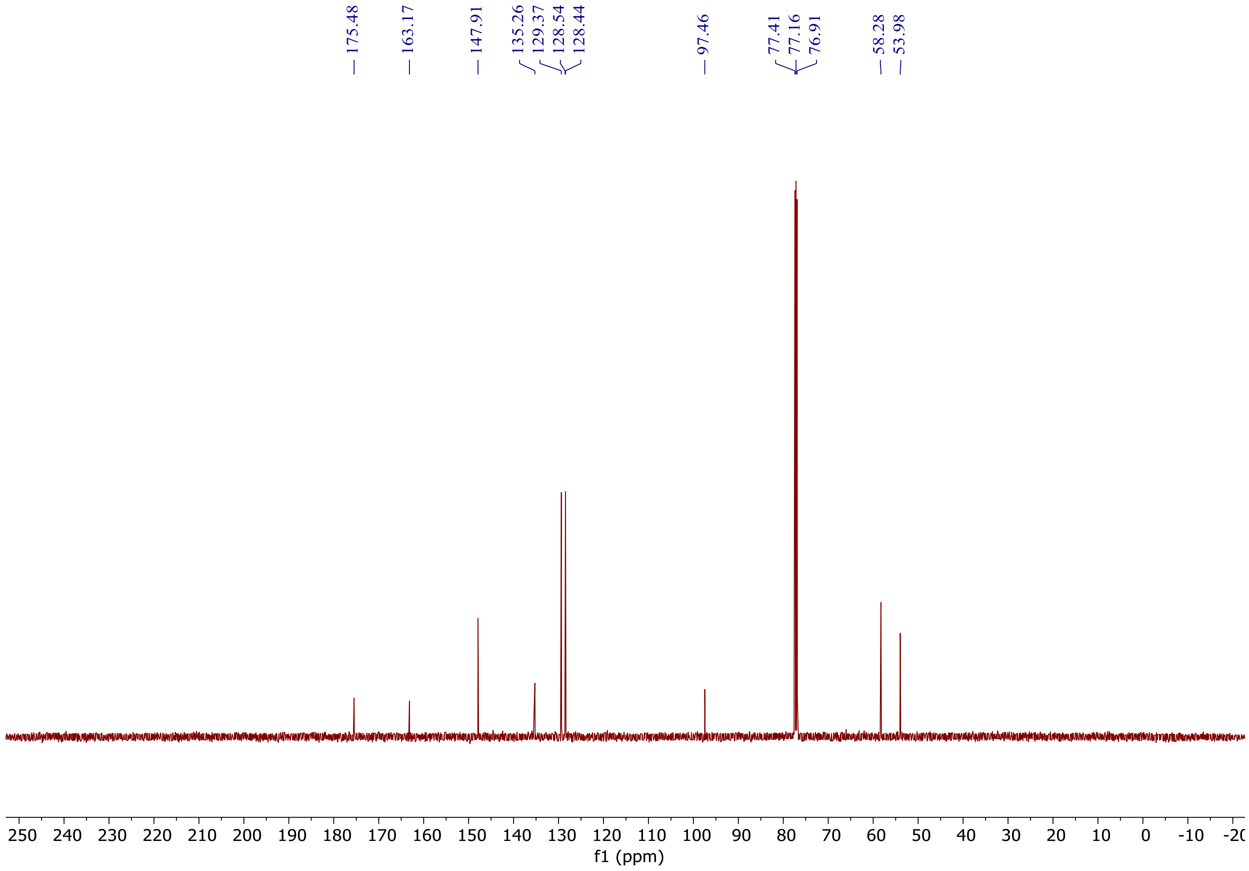

^{1}H and ^{13}C NMR traces of **9a**

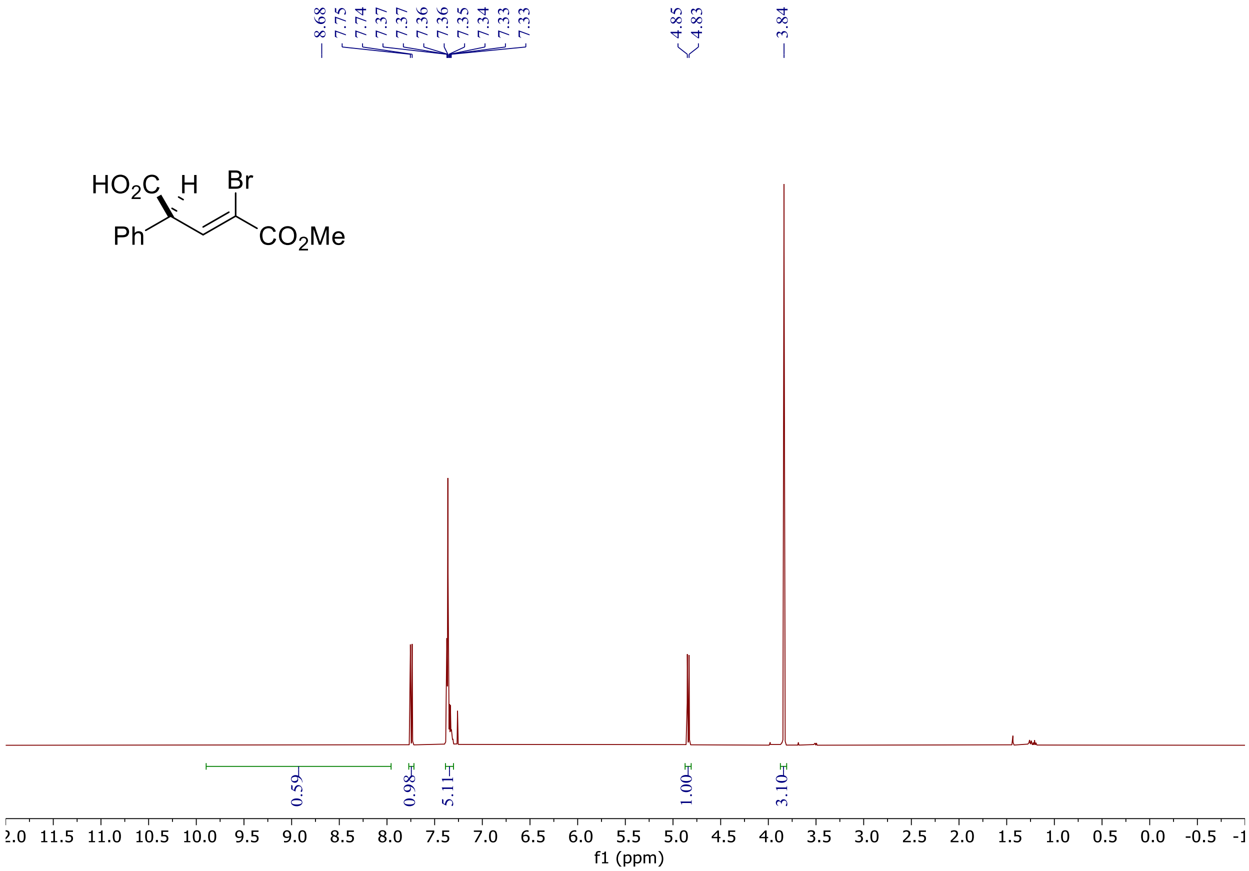

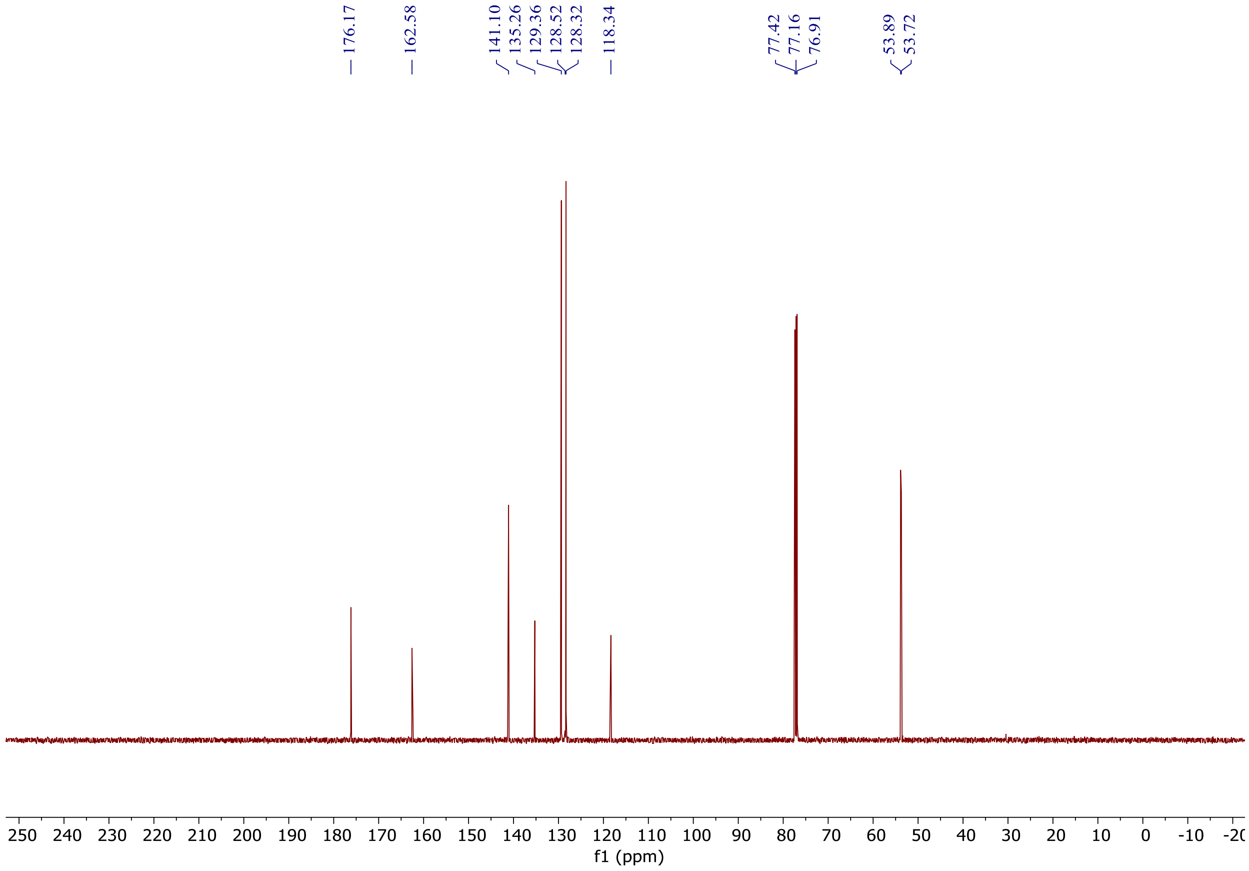

^{1}H and ^{13}C NMR traces of **9b**

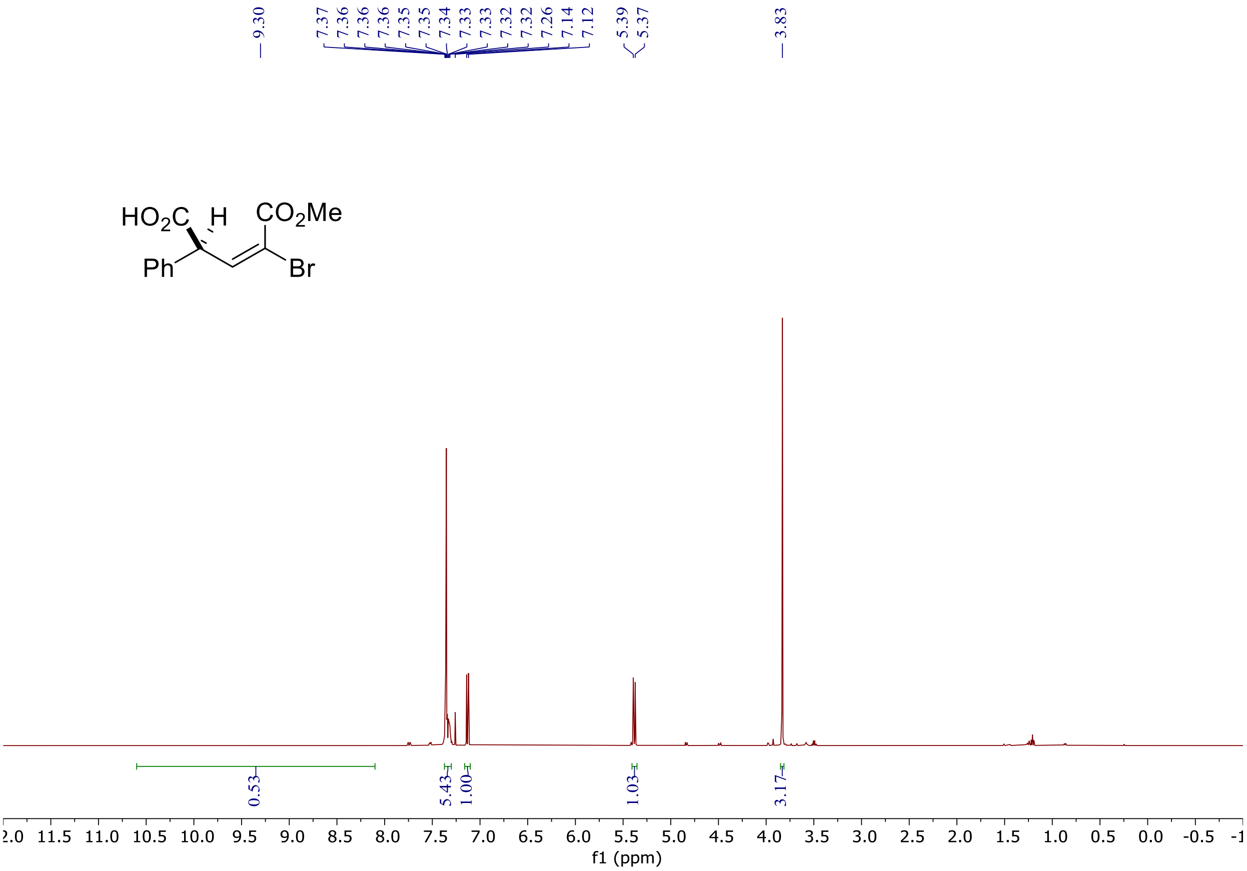

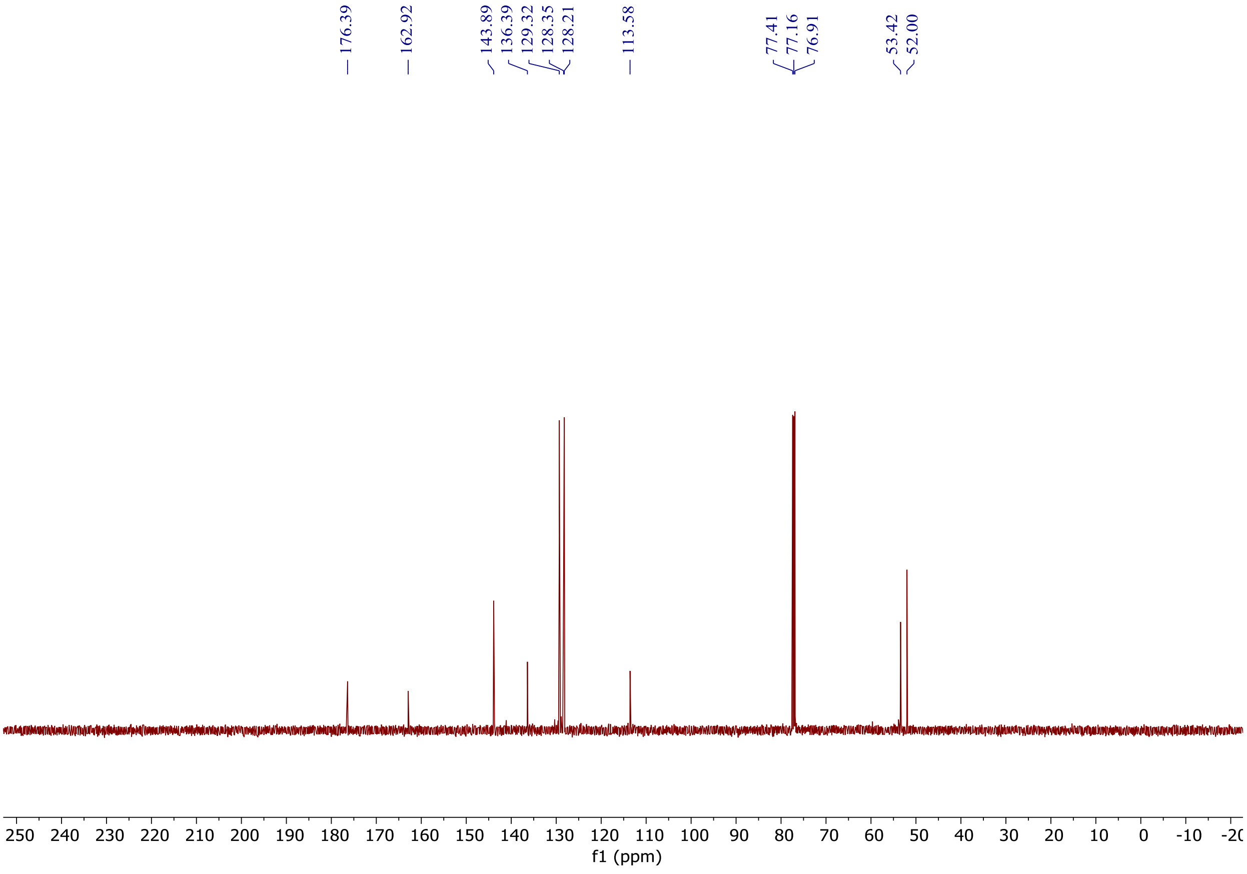

^{1}H and ^{13}C NMR traces of **10**

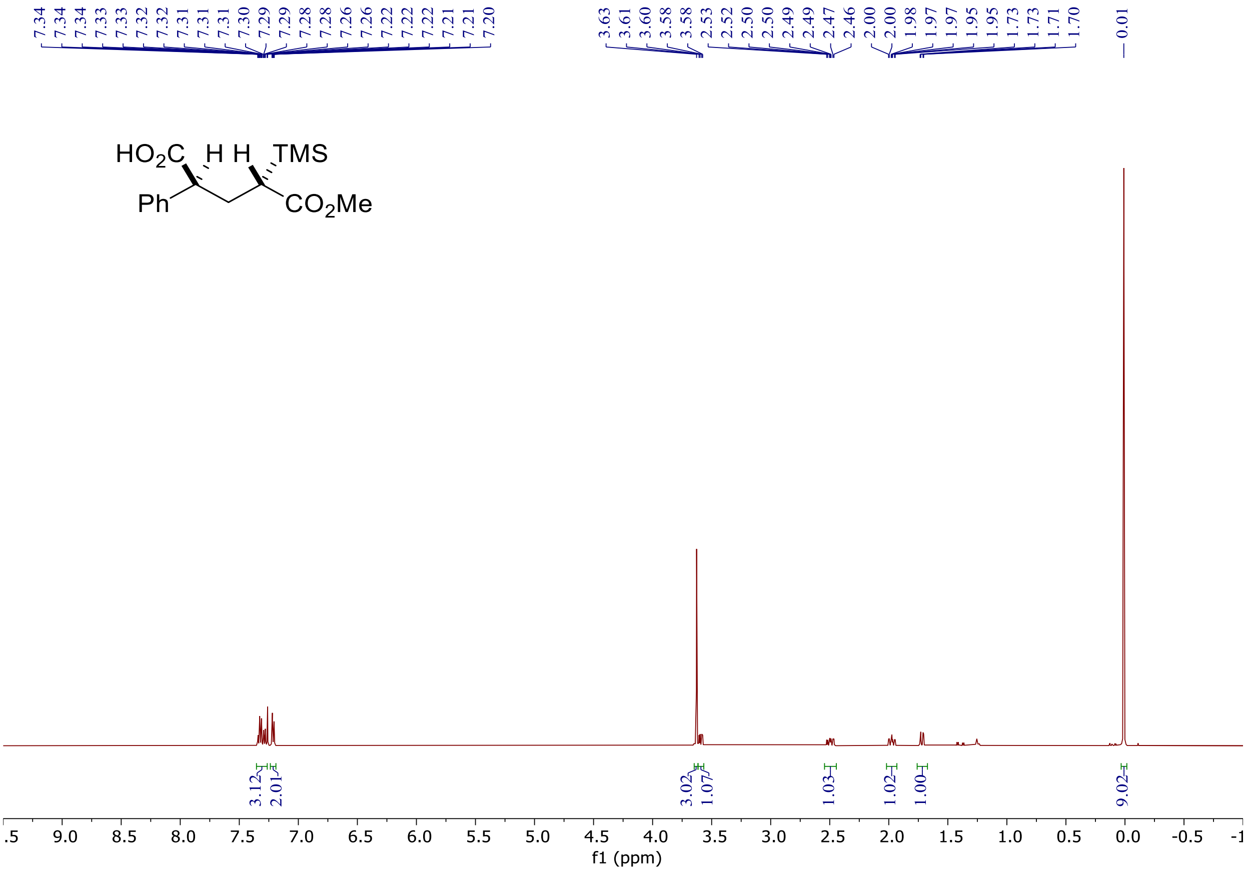

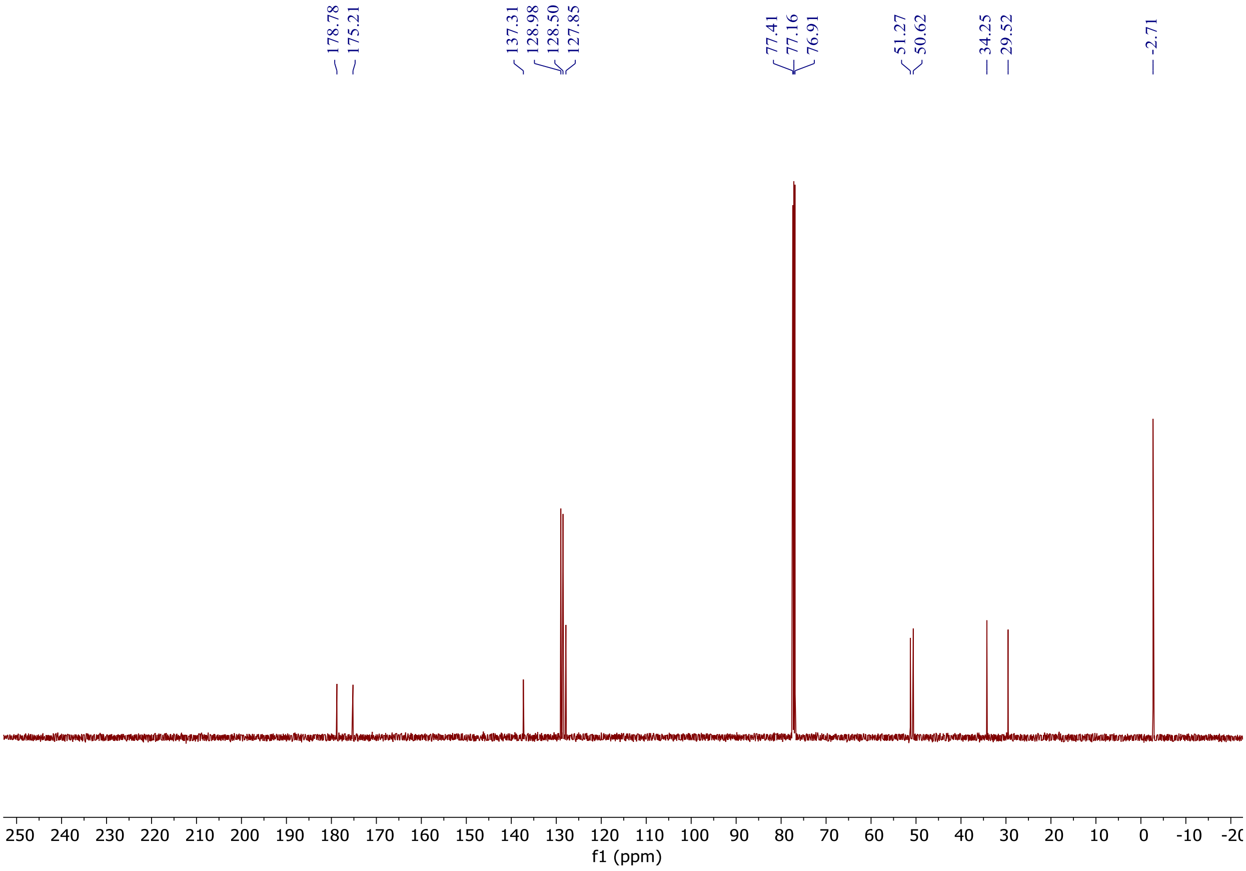

^{1}H and ^{13}C NMR traces of **11**

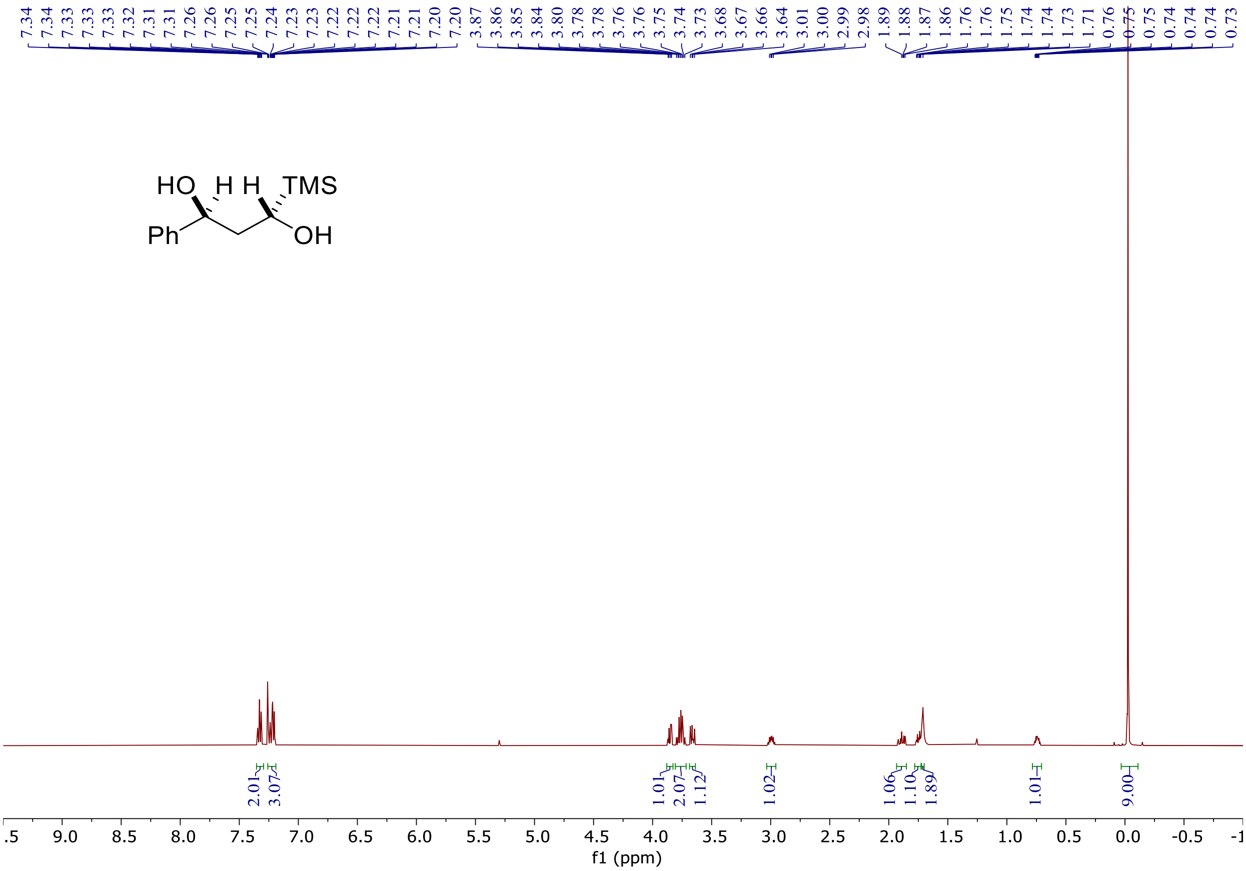

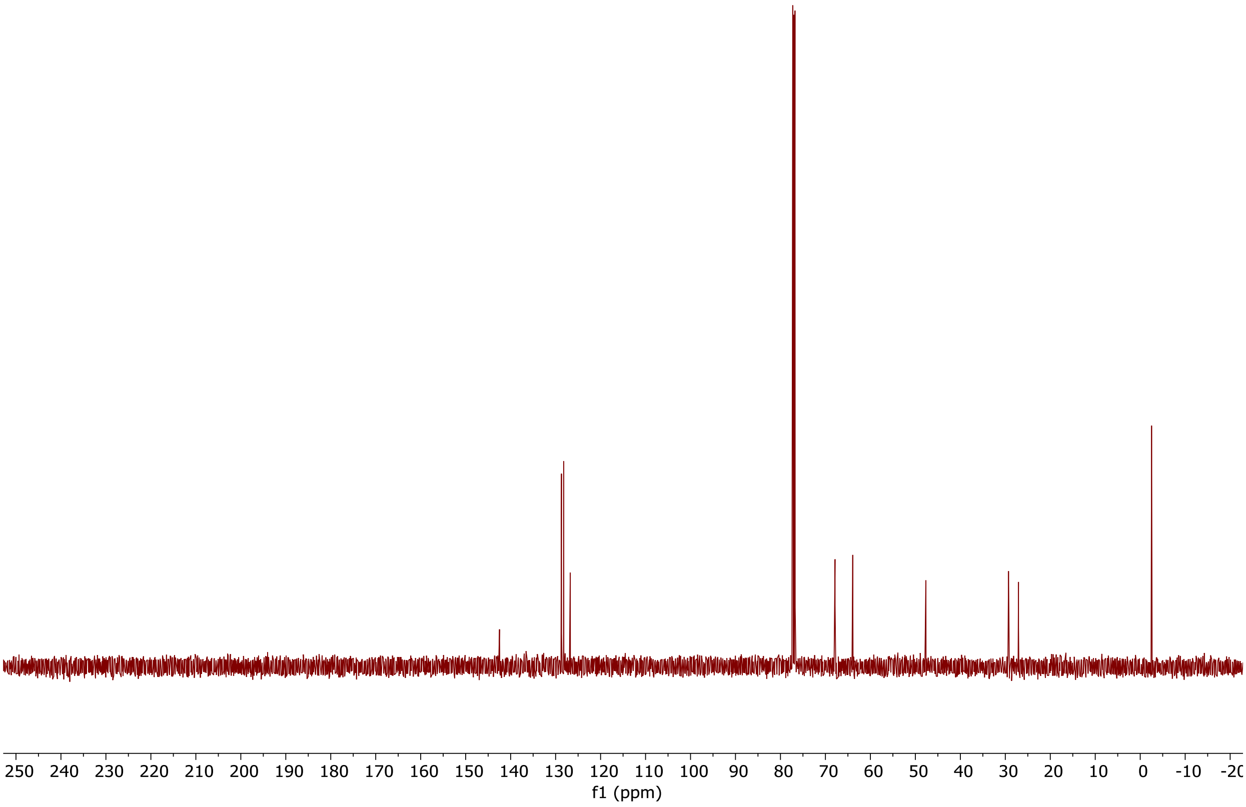

^{1}H and ^{13}C NMR traces of **12**

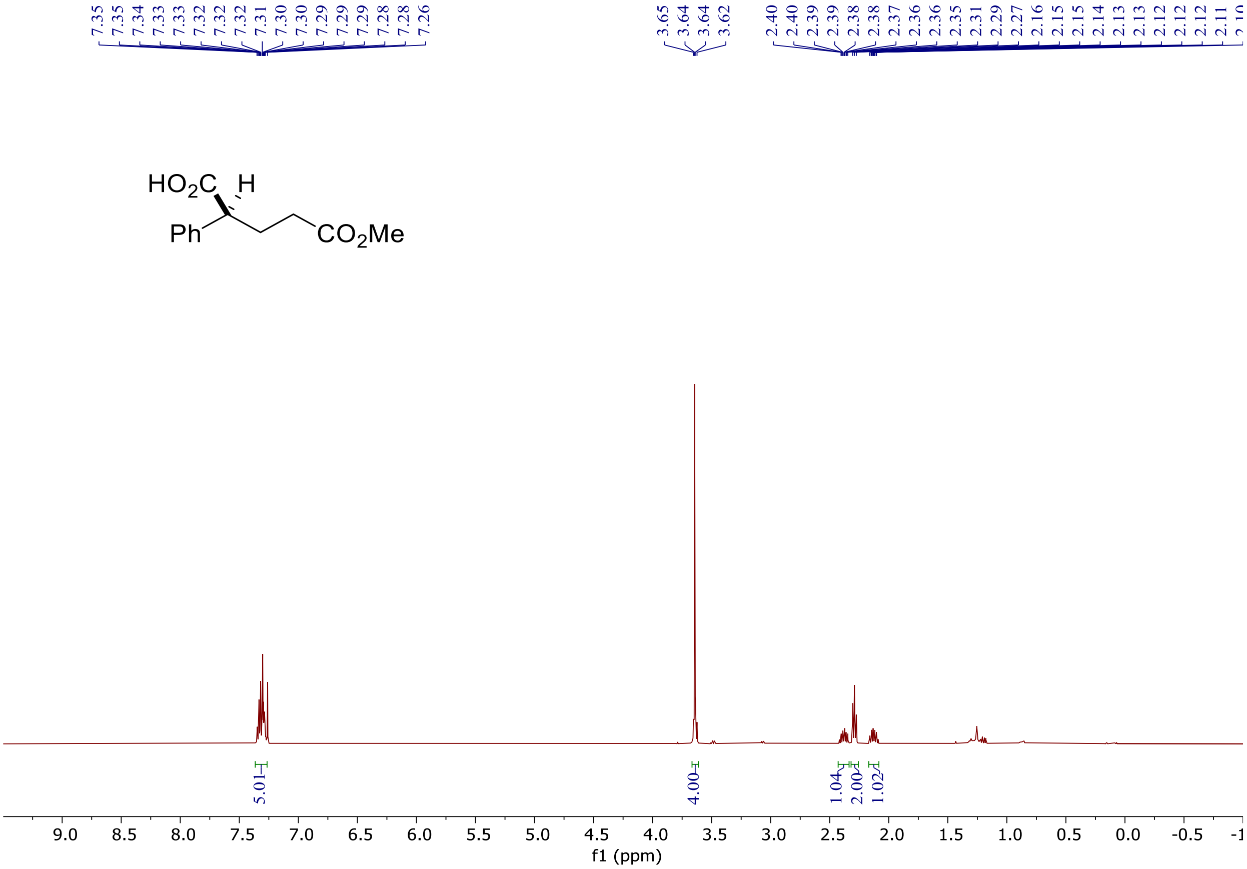

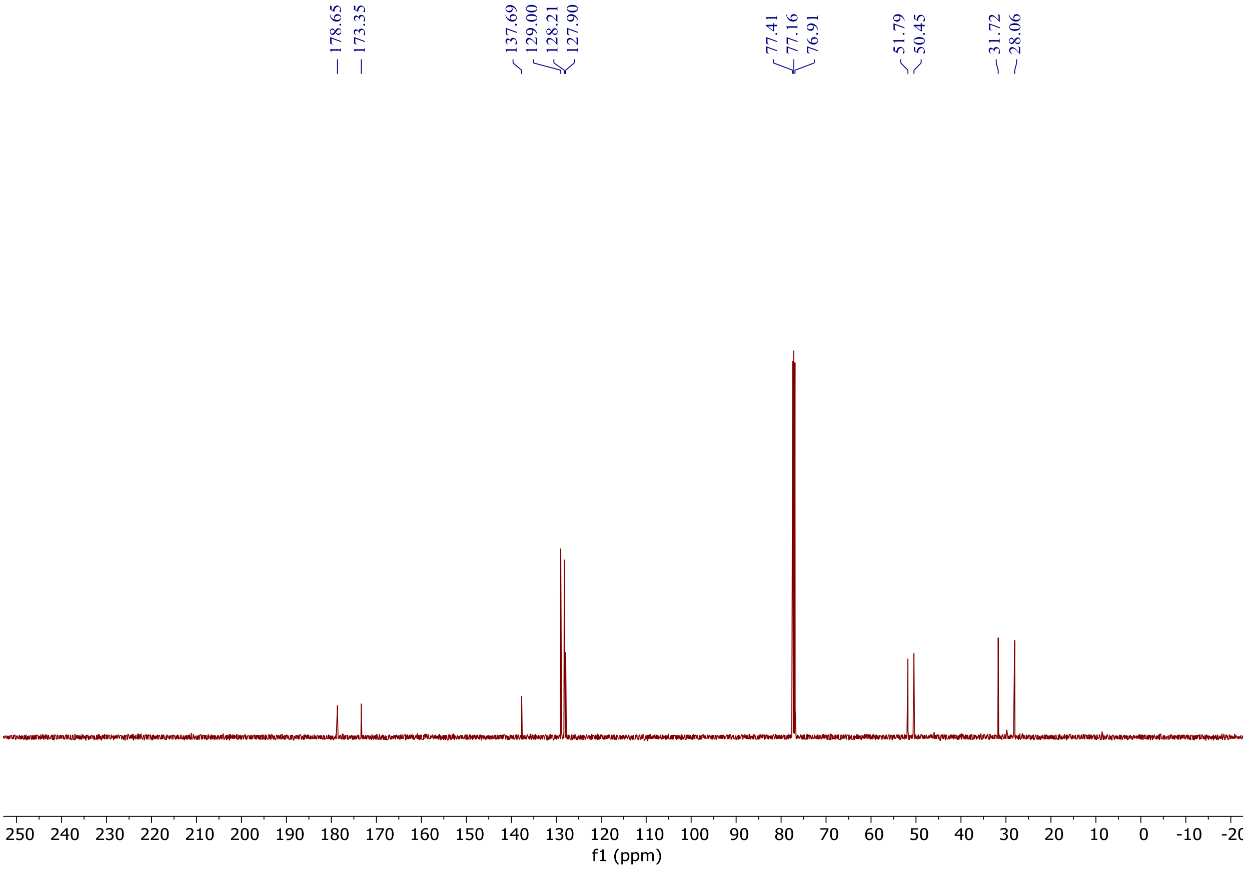

^{1}H and ^{13}C NMR traces of **13**

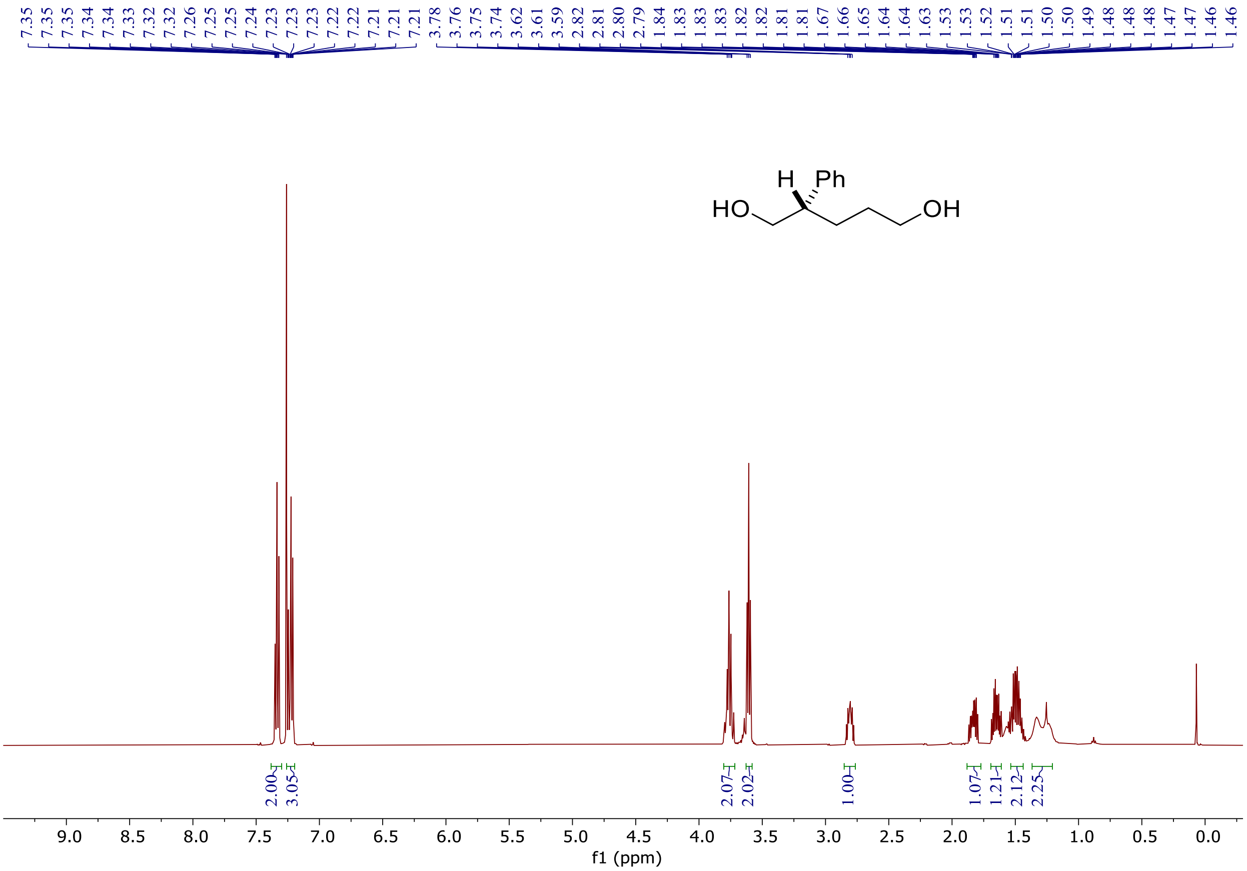

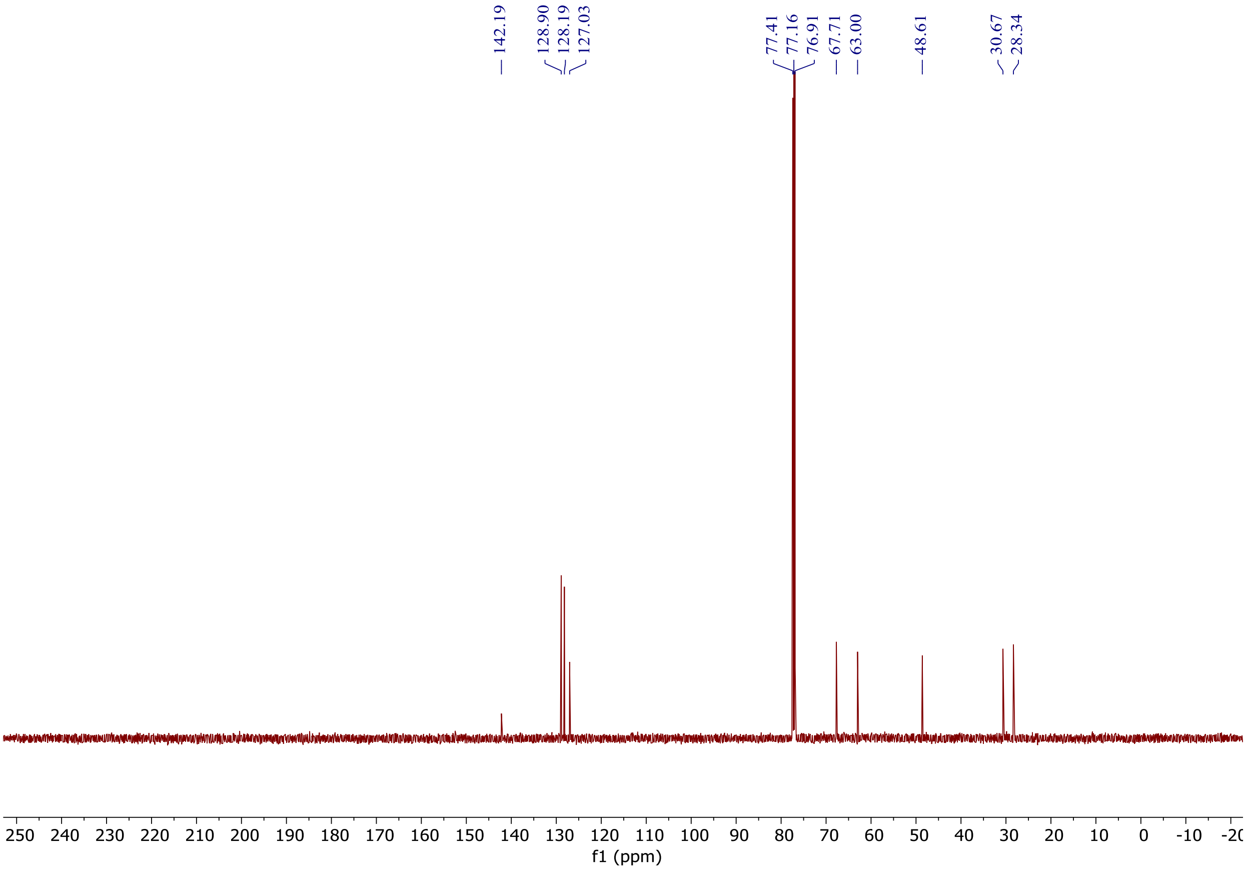

^{1}H and ^{13}C NMR traces of **14**

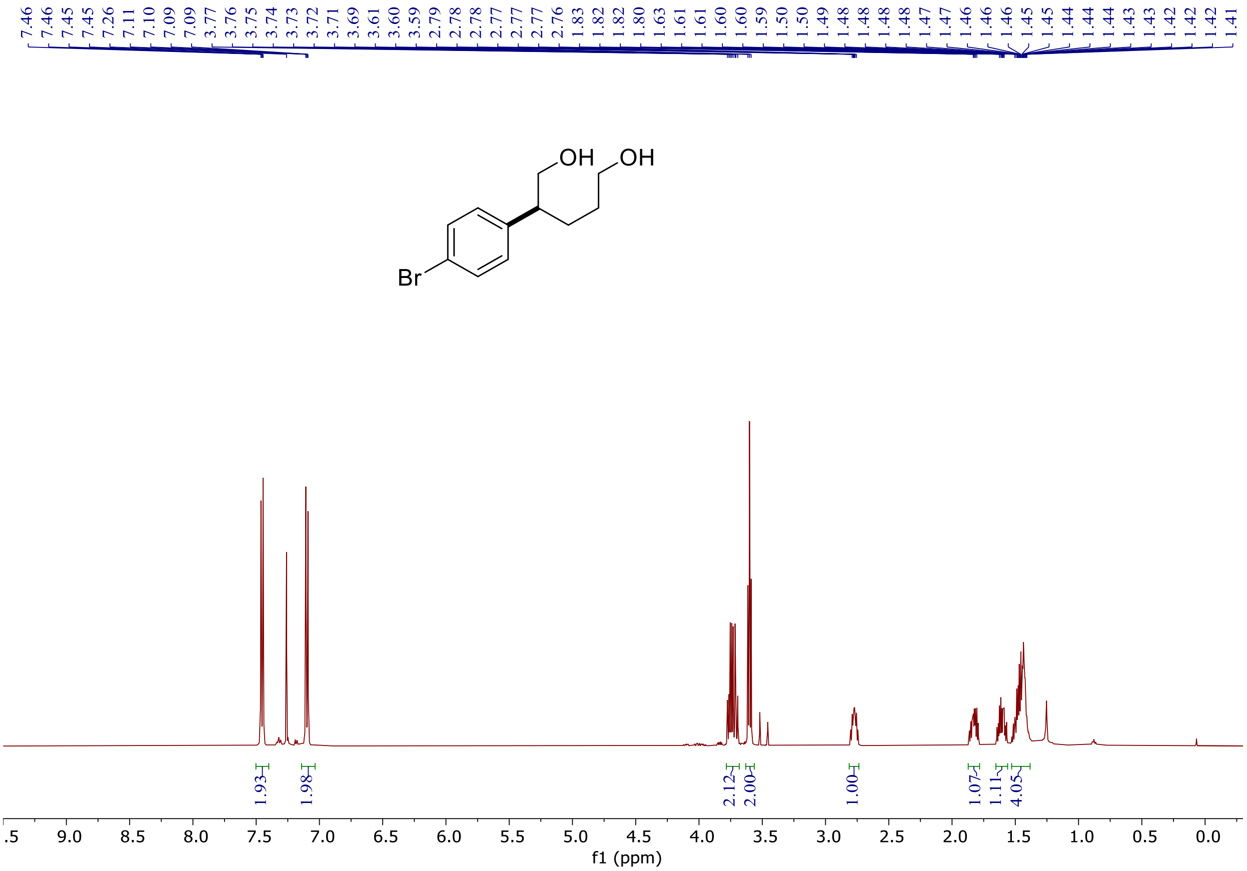

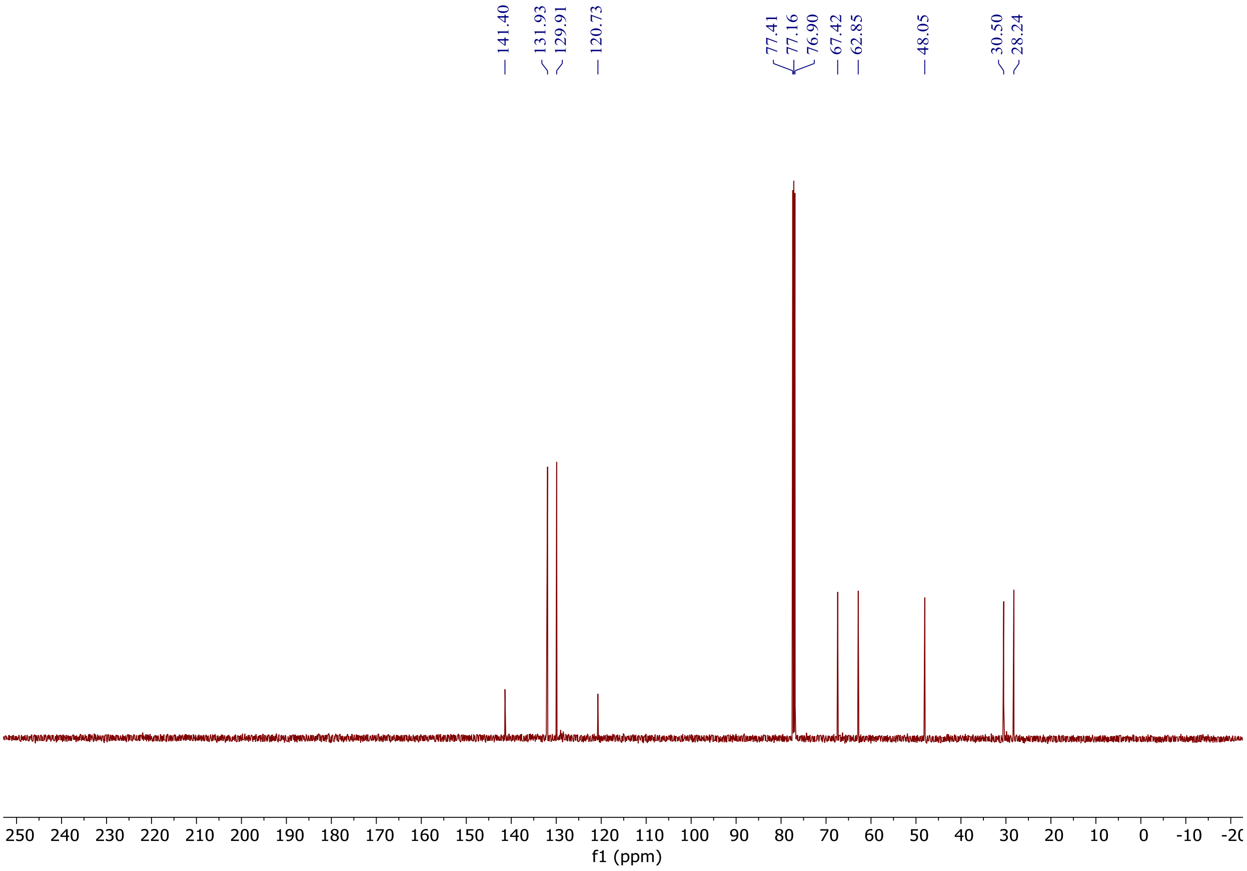

^{1}H and ^{13}C NMR traces of **15**

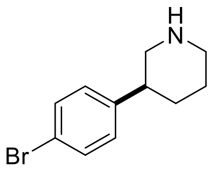

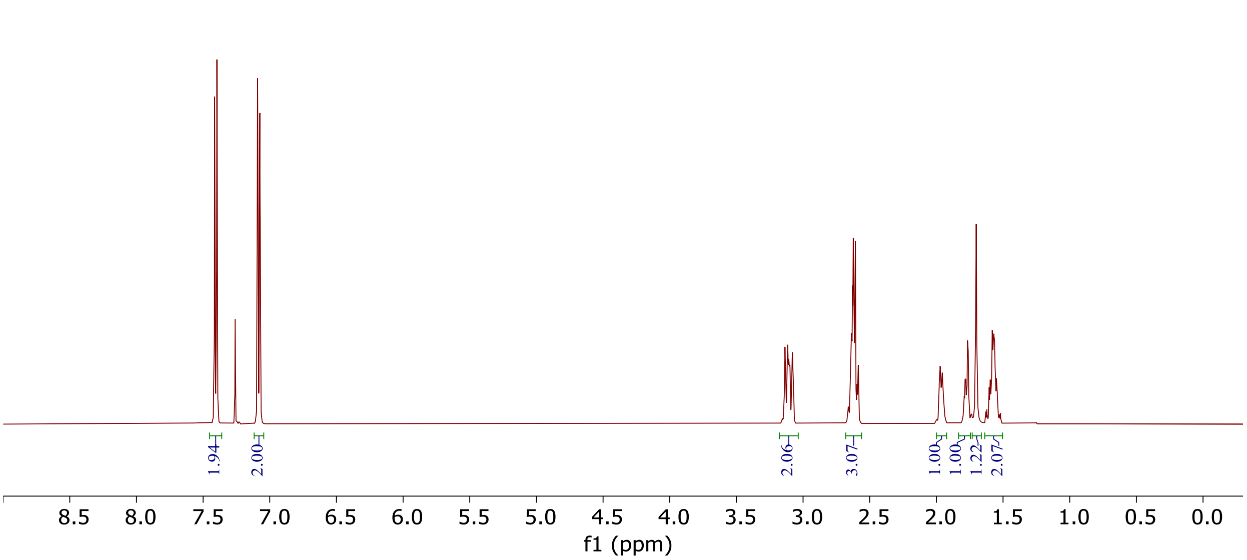

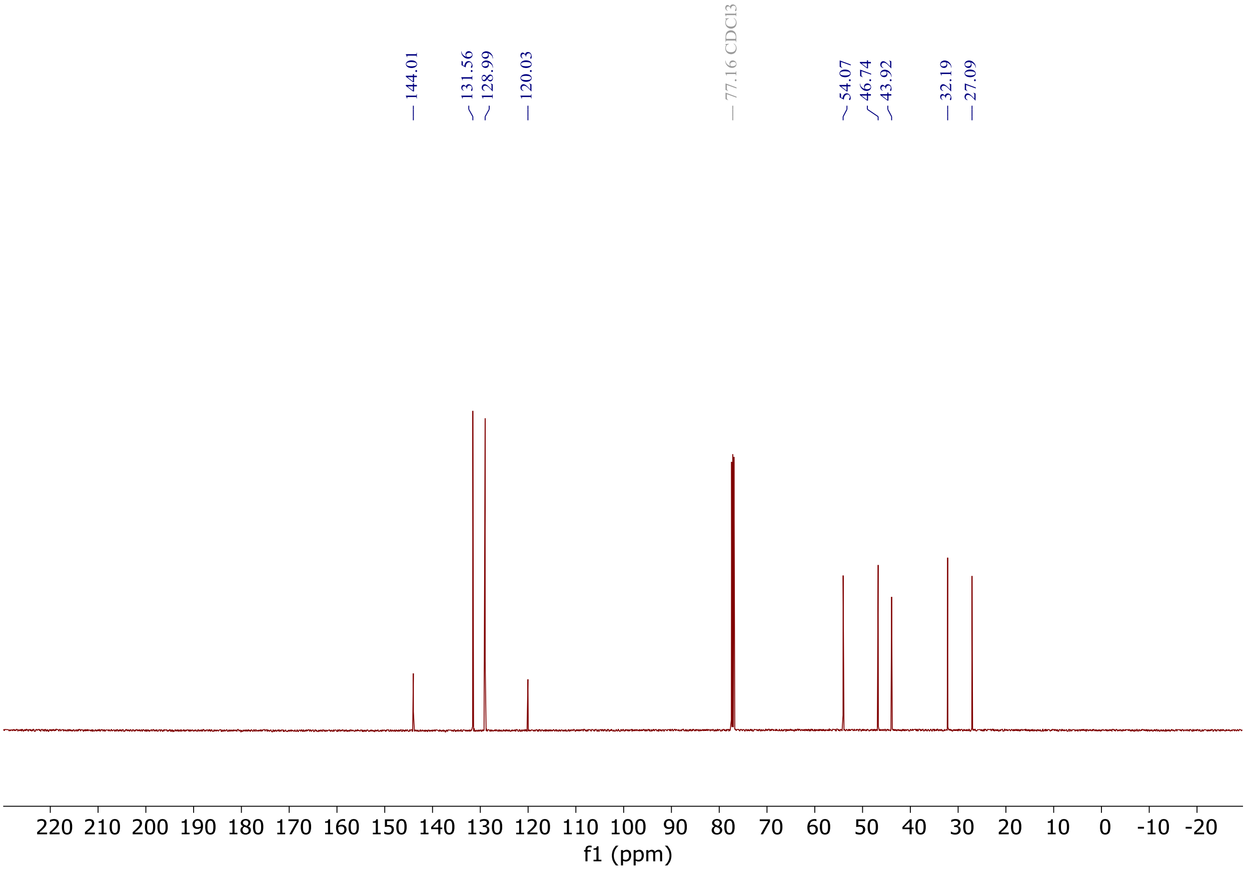

^1H and ^{13}C NMR traces of *p*-Toluenesulfonate of **15** (in DMSO-d_6)

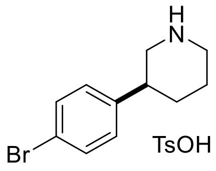

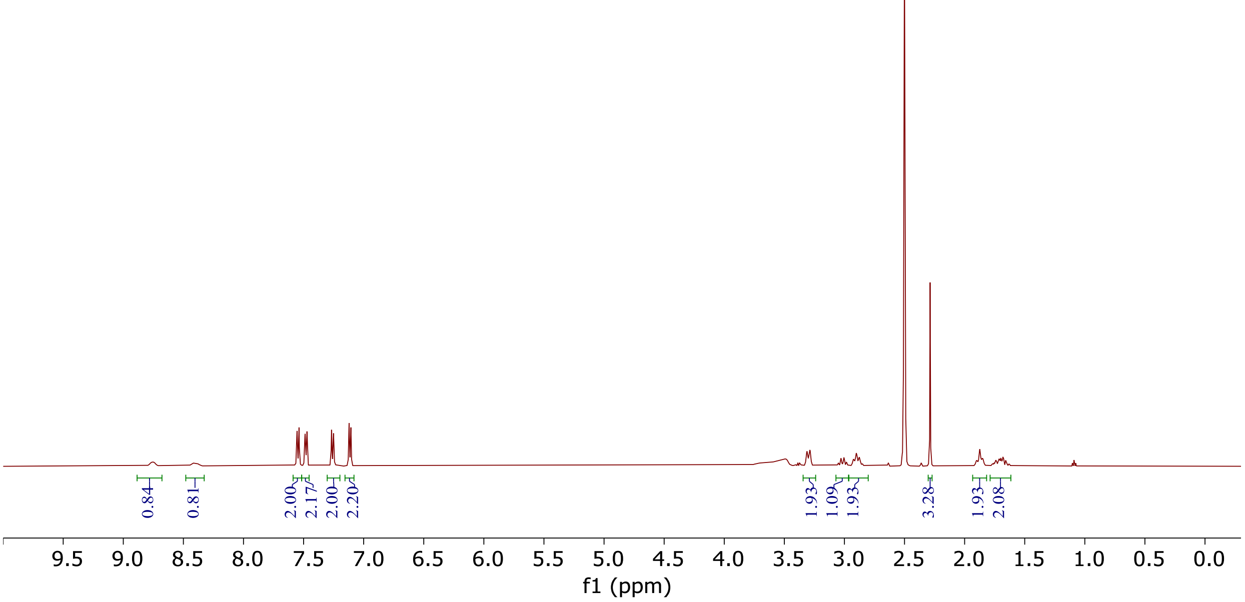

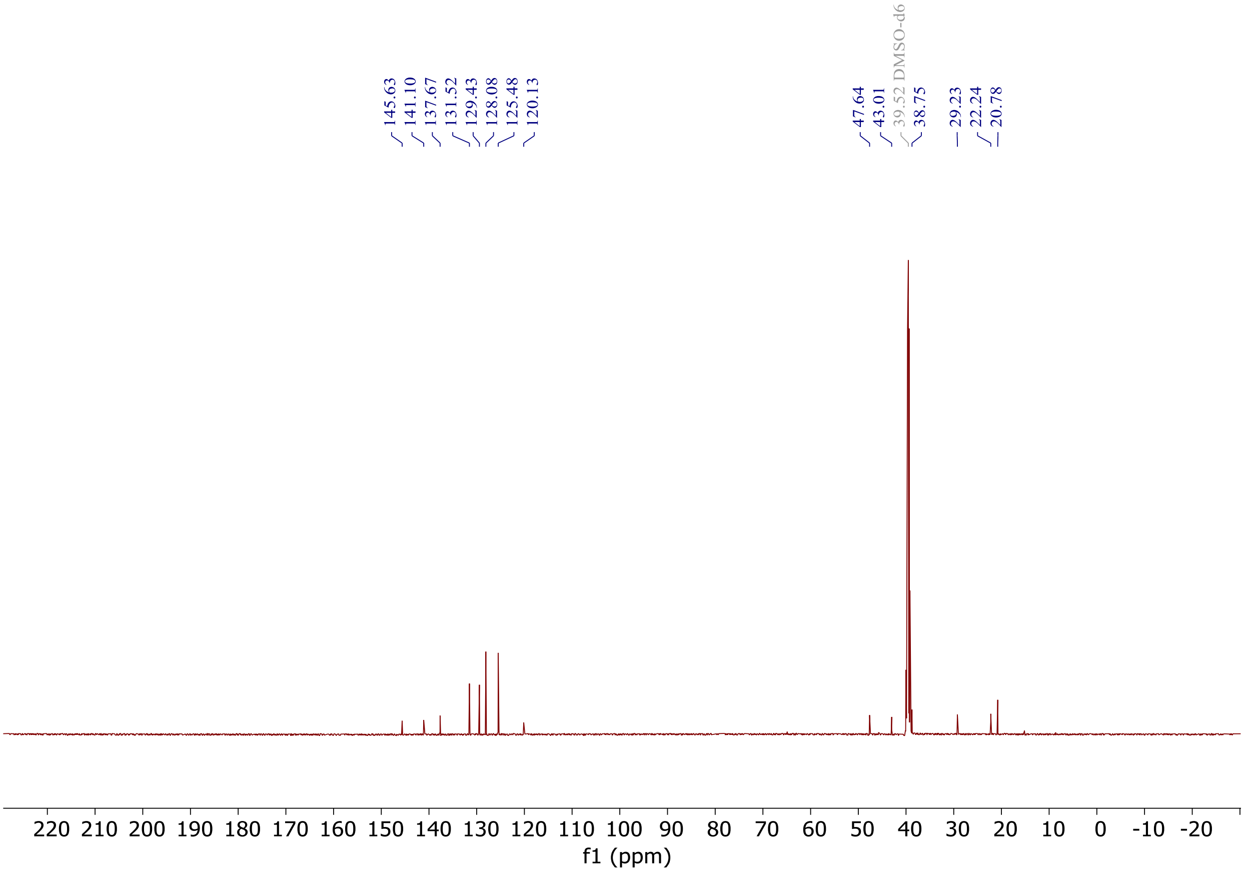

^{1}H and ^{13}C NMR traces of *p*-Toluenesulfonate of **15** (in $CDCl_3$)

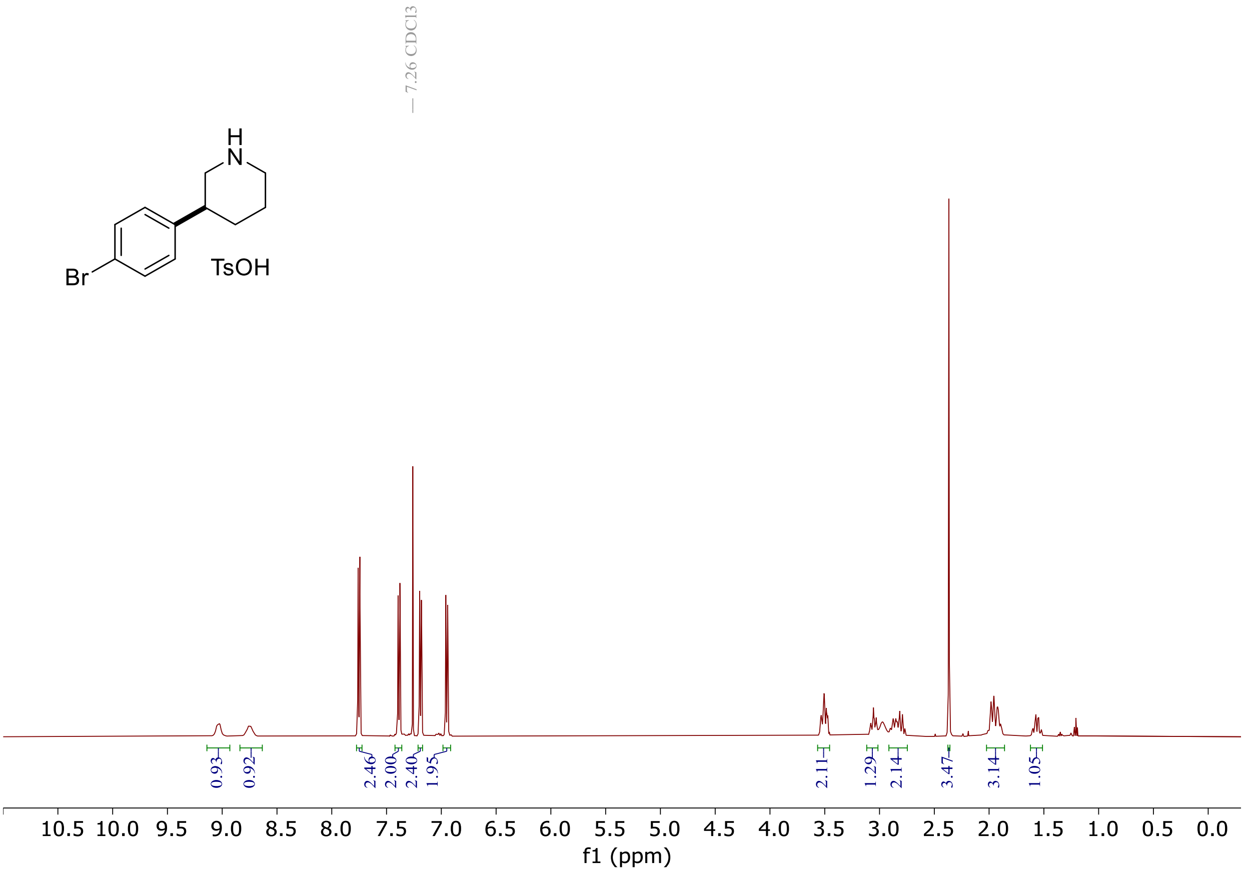

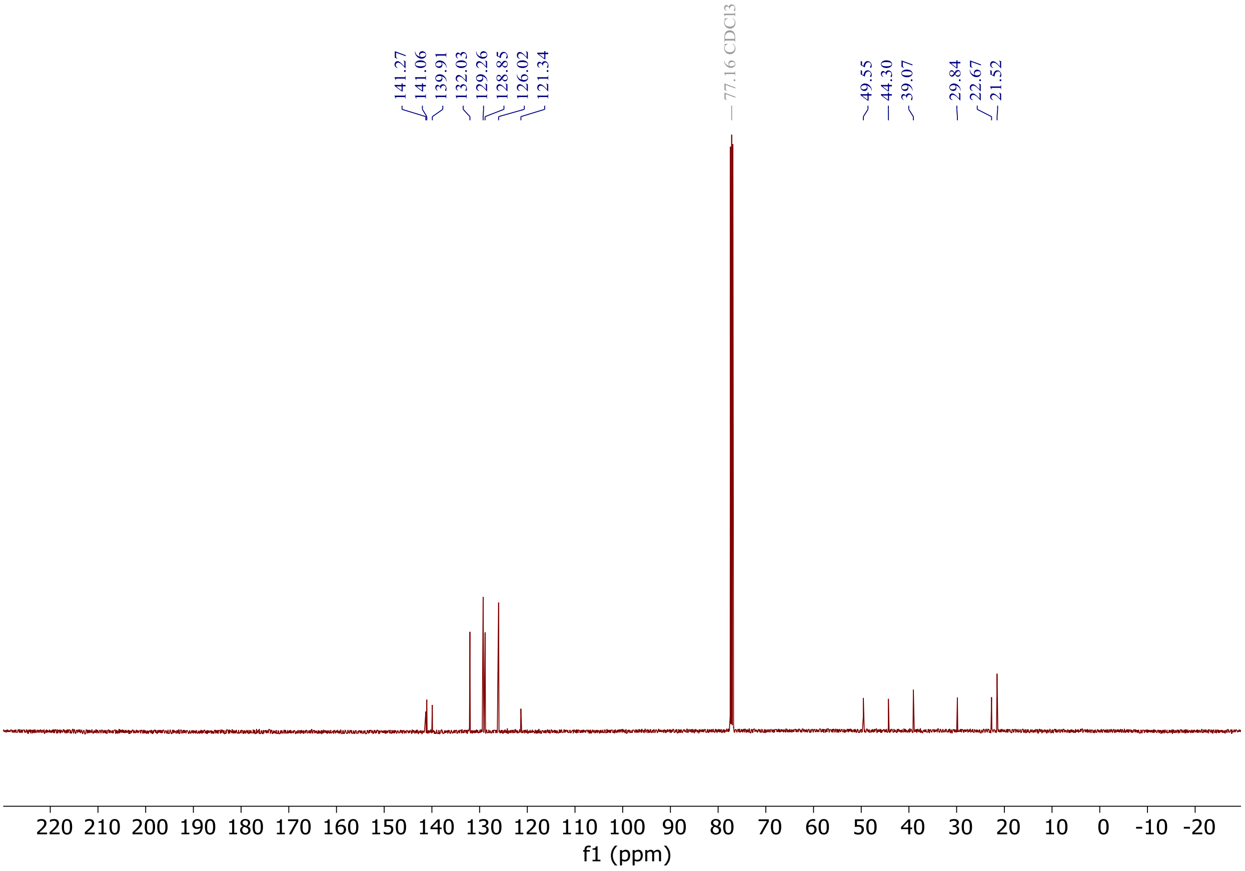

^1H and ^{13}C NMR traces of **s4a**

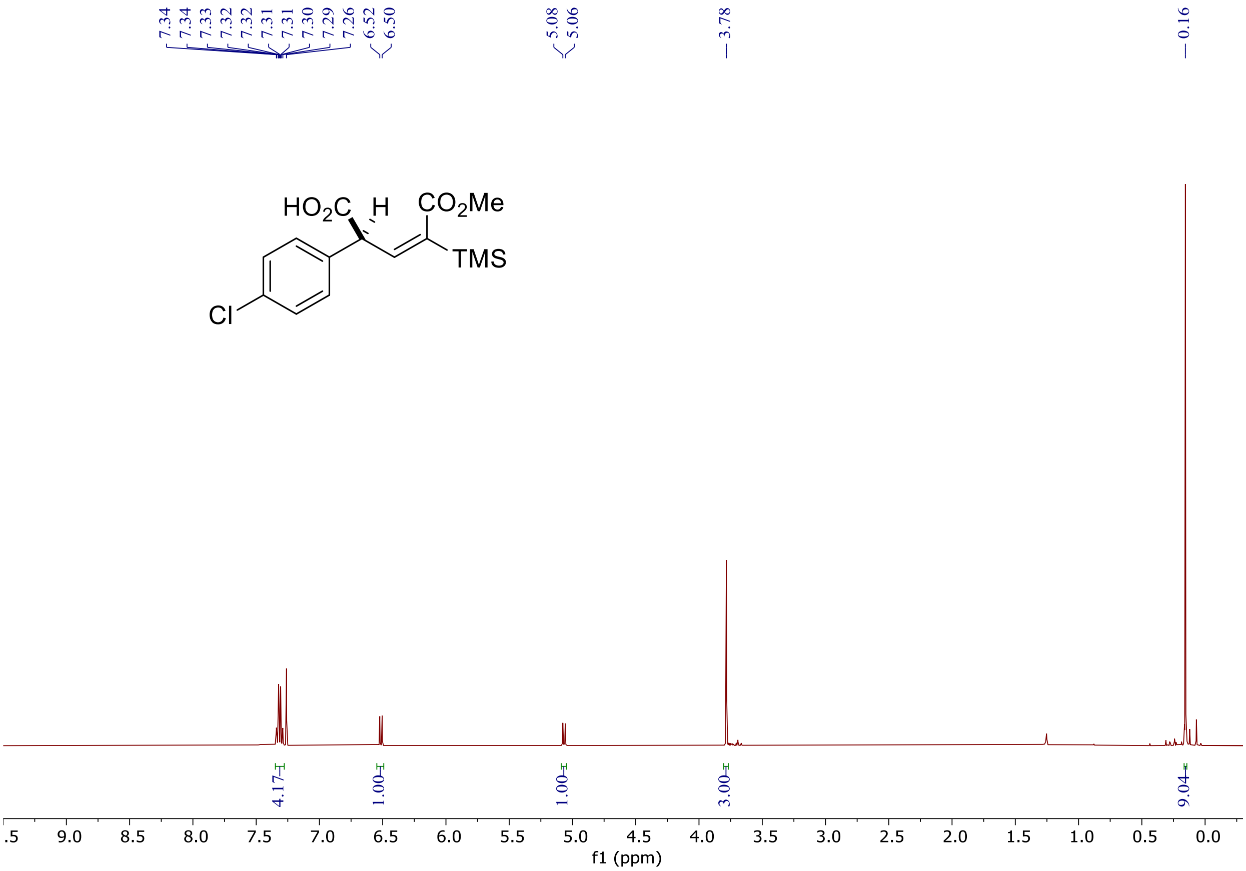

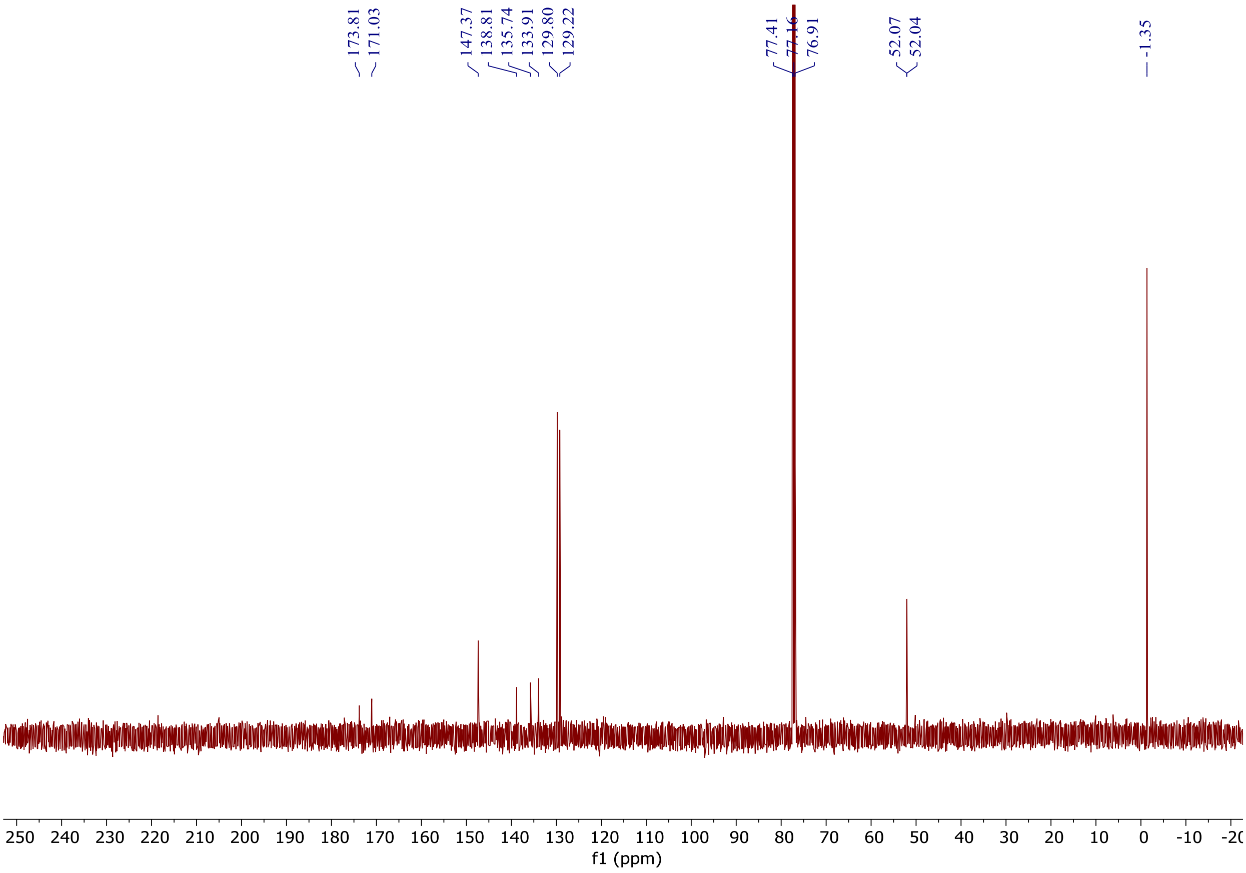

^{1}H, ^{13}C and ^{19}F NMR traces of **s4b**

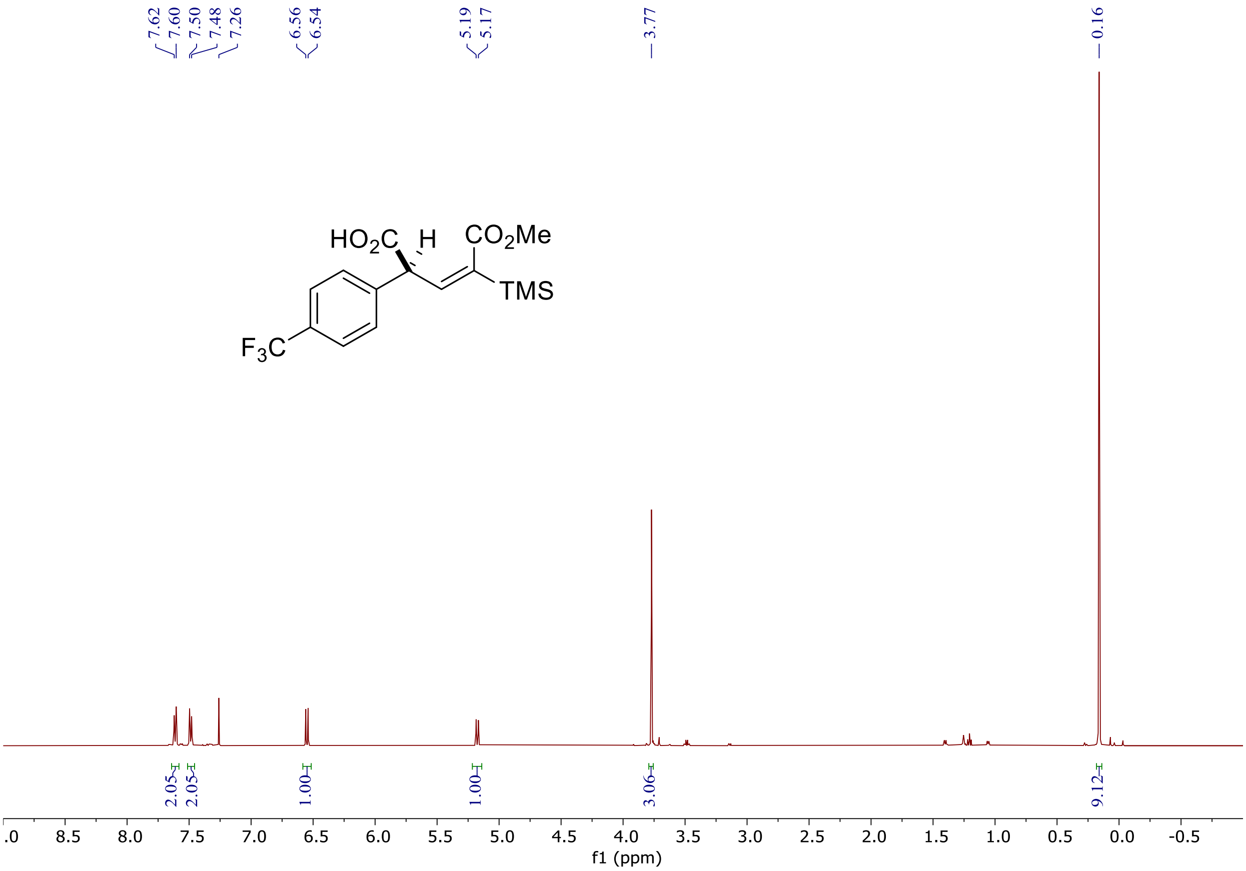

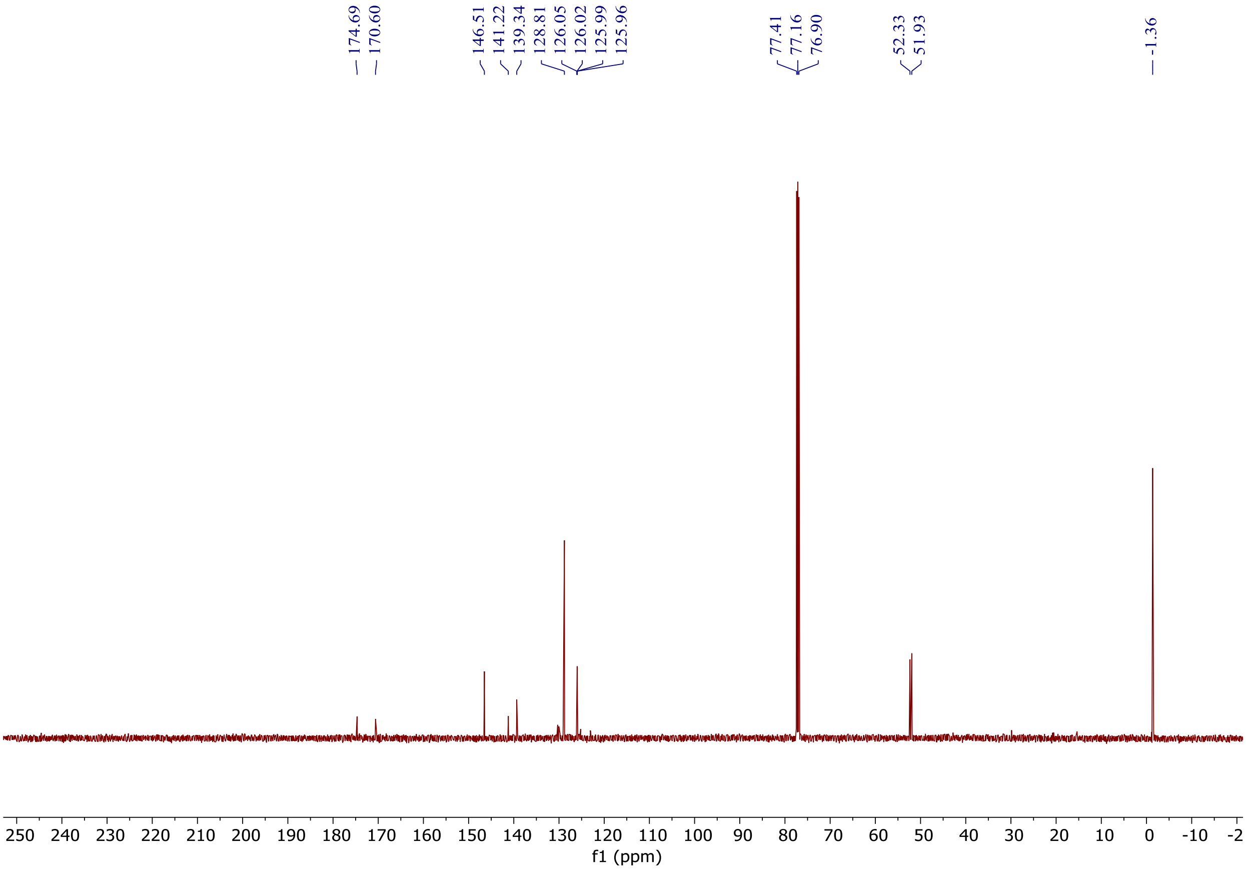

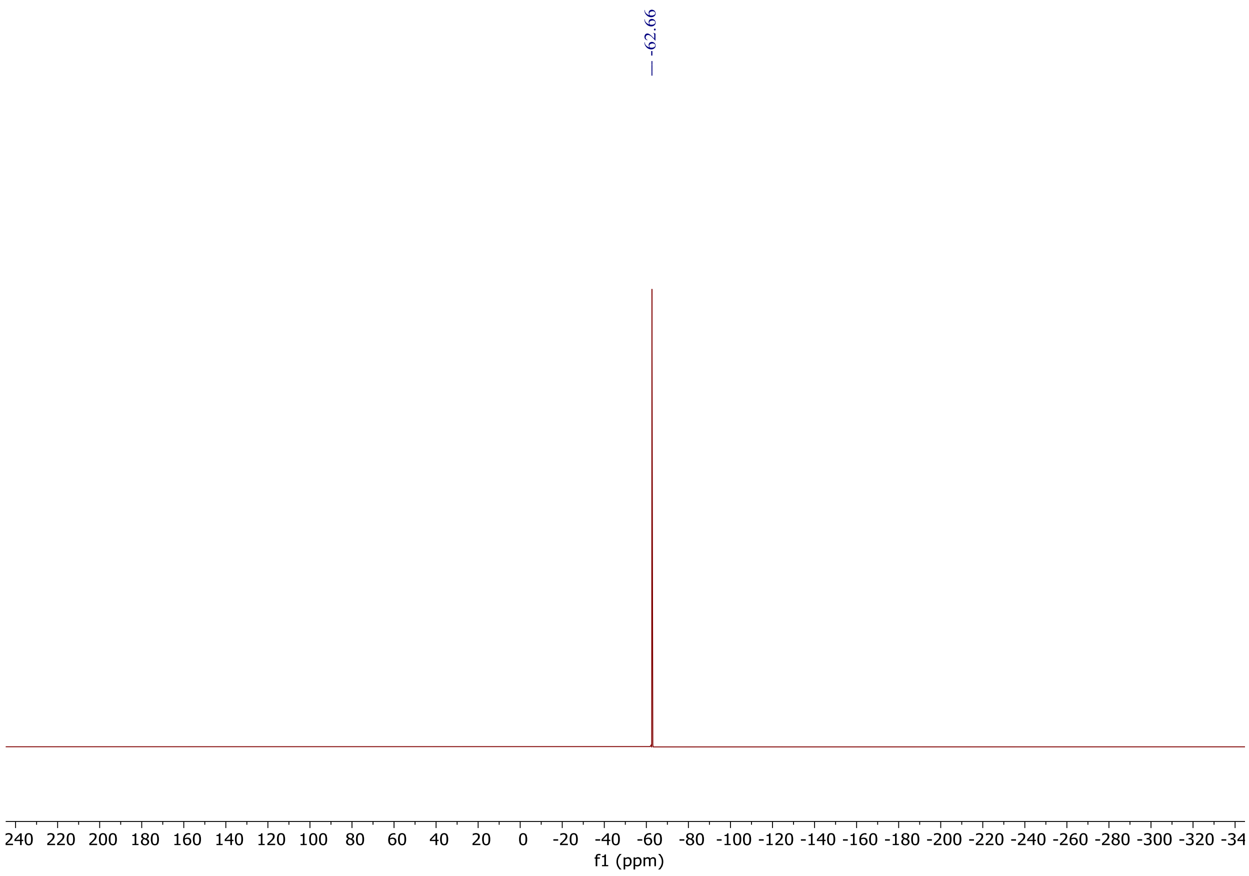
-62.66
240 220 200 180 160 140 120 100 80 60 40 20 0 -20 -40 -60 -80 -100 -120 -140 -160 -180 -200 -220 -240 -260 -280 -300 -320 -34
f1 (ppm)

^{1}H and ^{13}C NMR traces of **s4c**

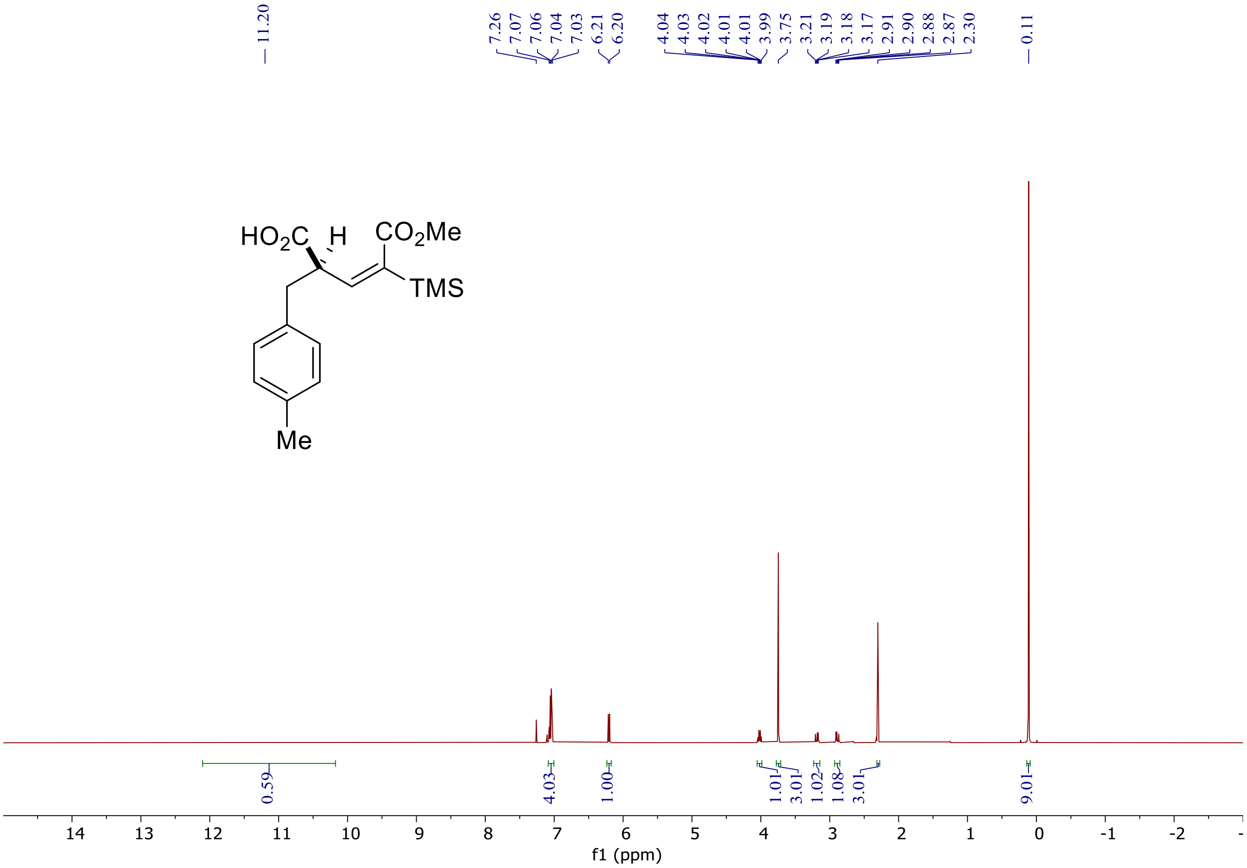

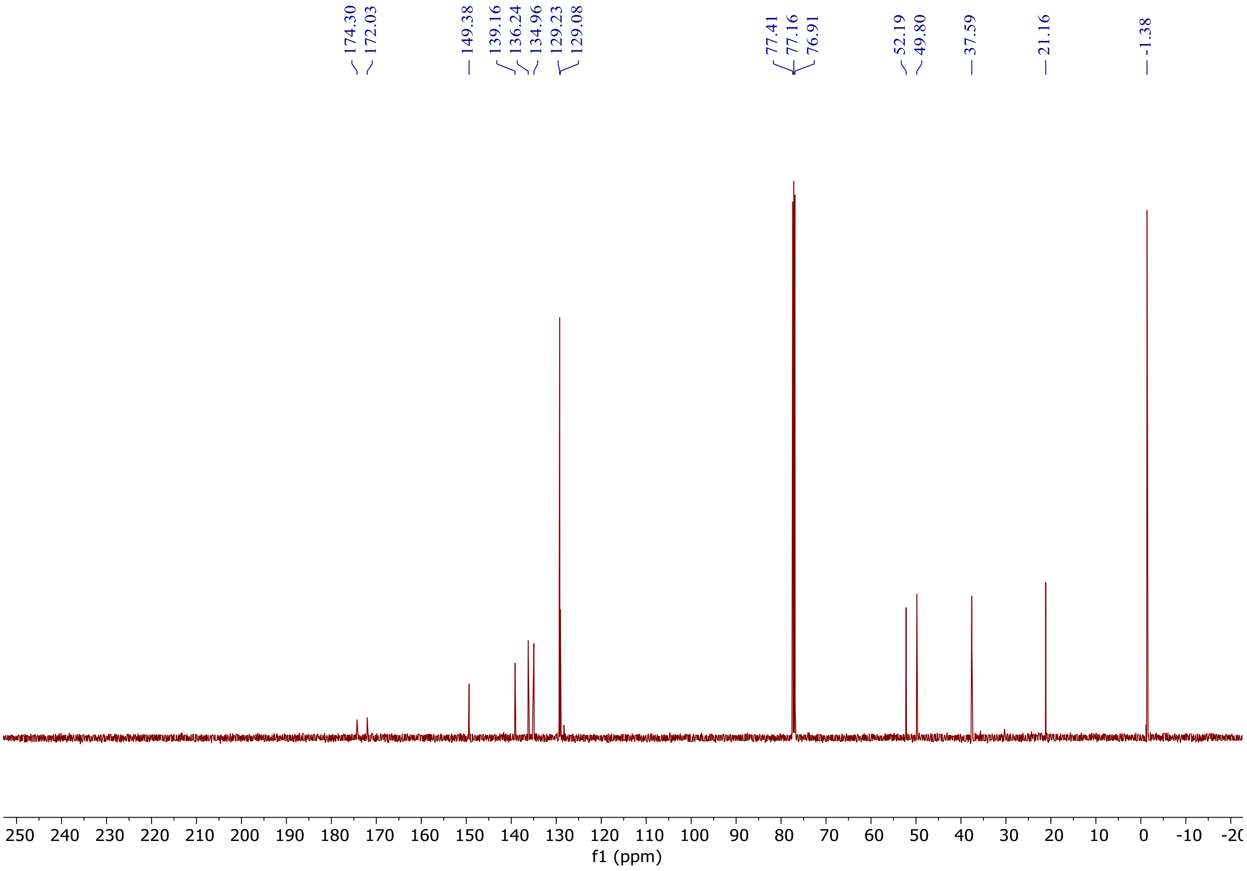

^{1}H, ^{13}C and ^{19}F NMR traces of **s4d**

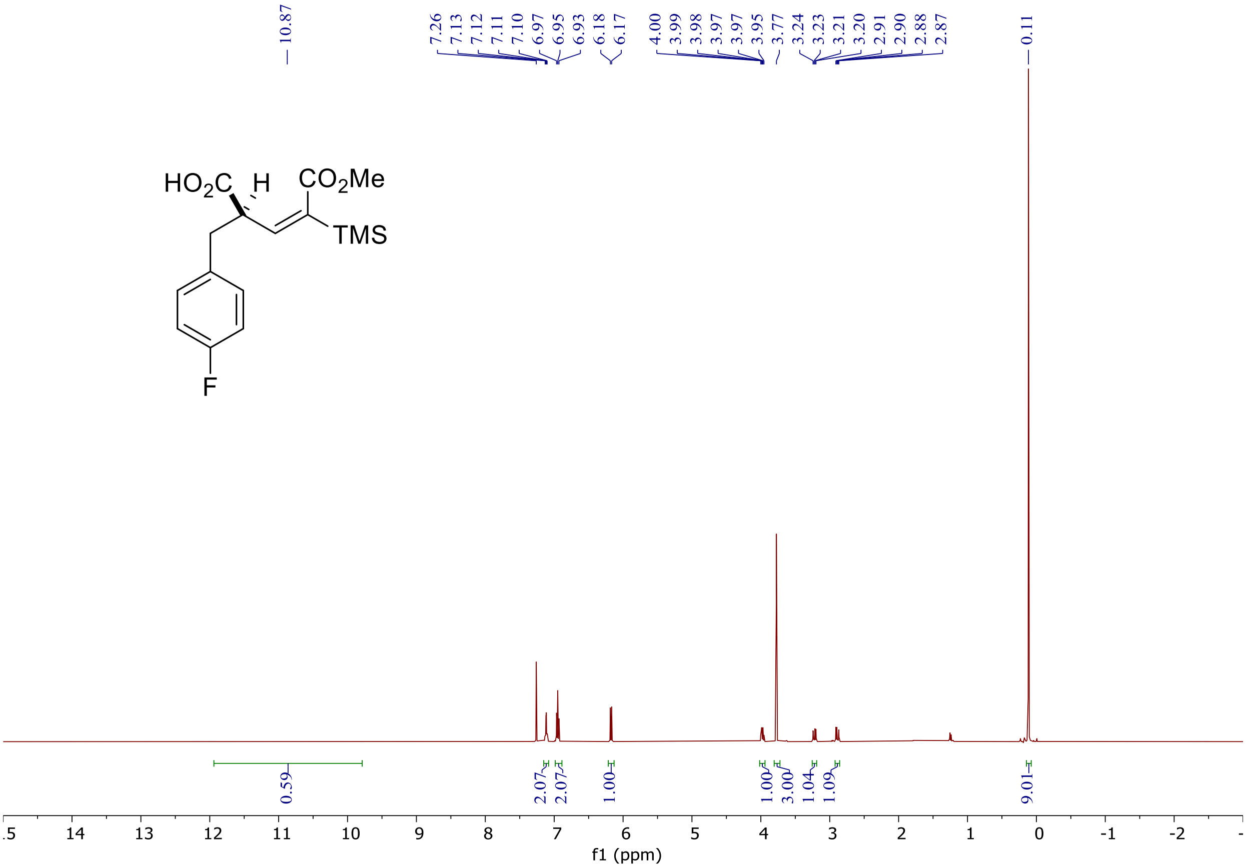

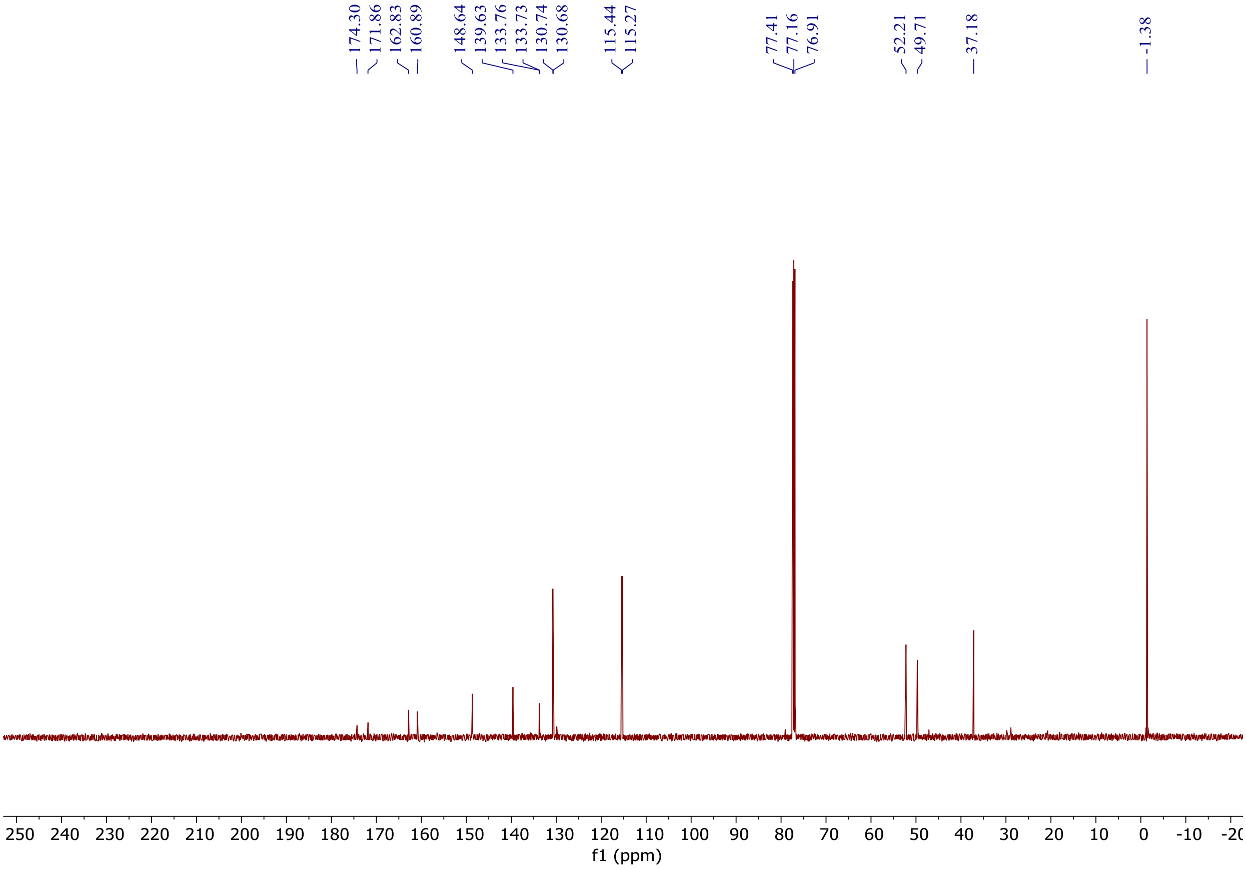

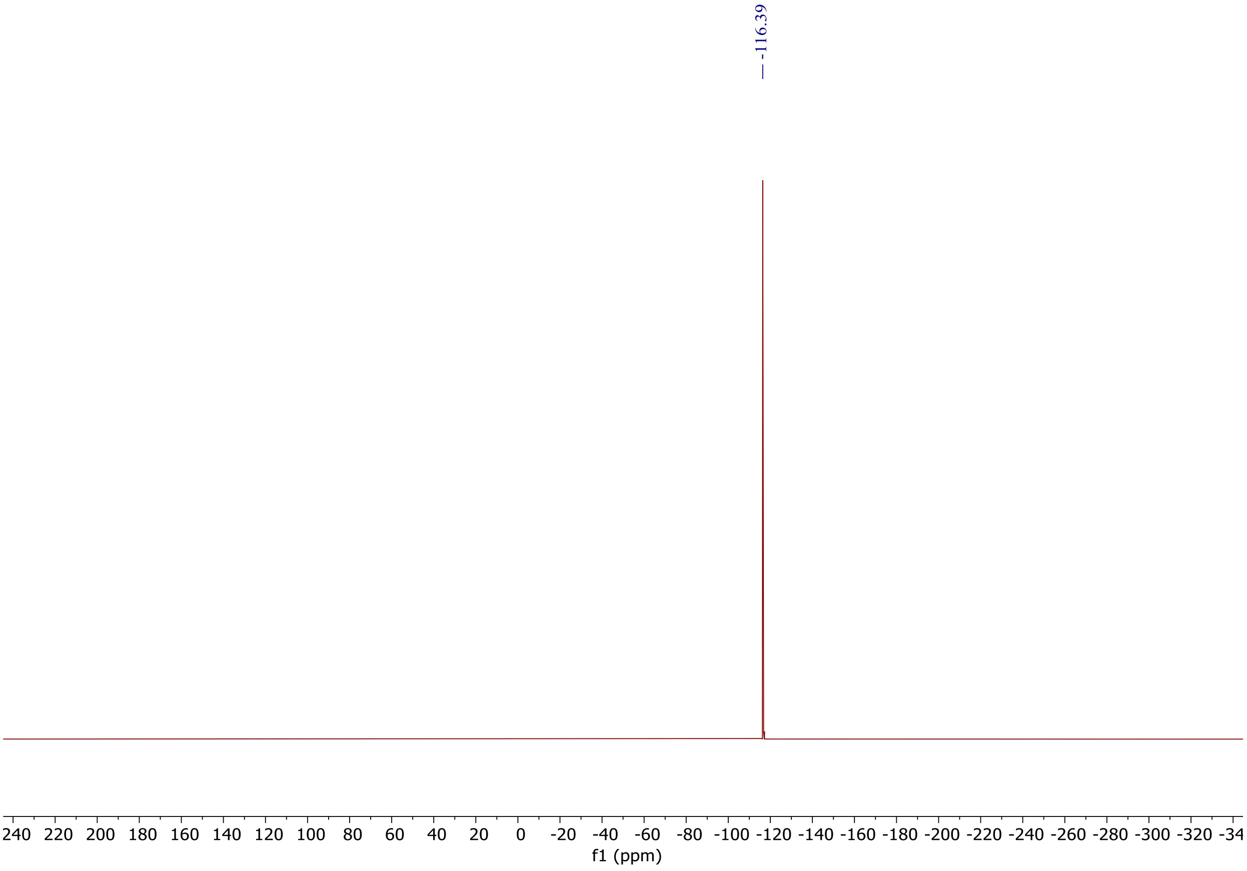
-116.39
240 220 200 180 160 140 120 100 80 60 40 20 0 -20 -40 -60 -80 -100 -120 -140 -160 -180 -200 -220 -240 -260 -280 -300 -320 -34
f1 (ppm)

^{1}H, ^{13}C and ^{19}F NMR traces of **s4e**

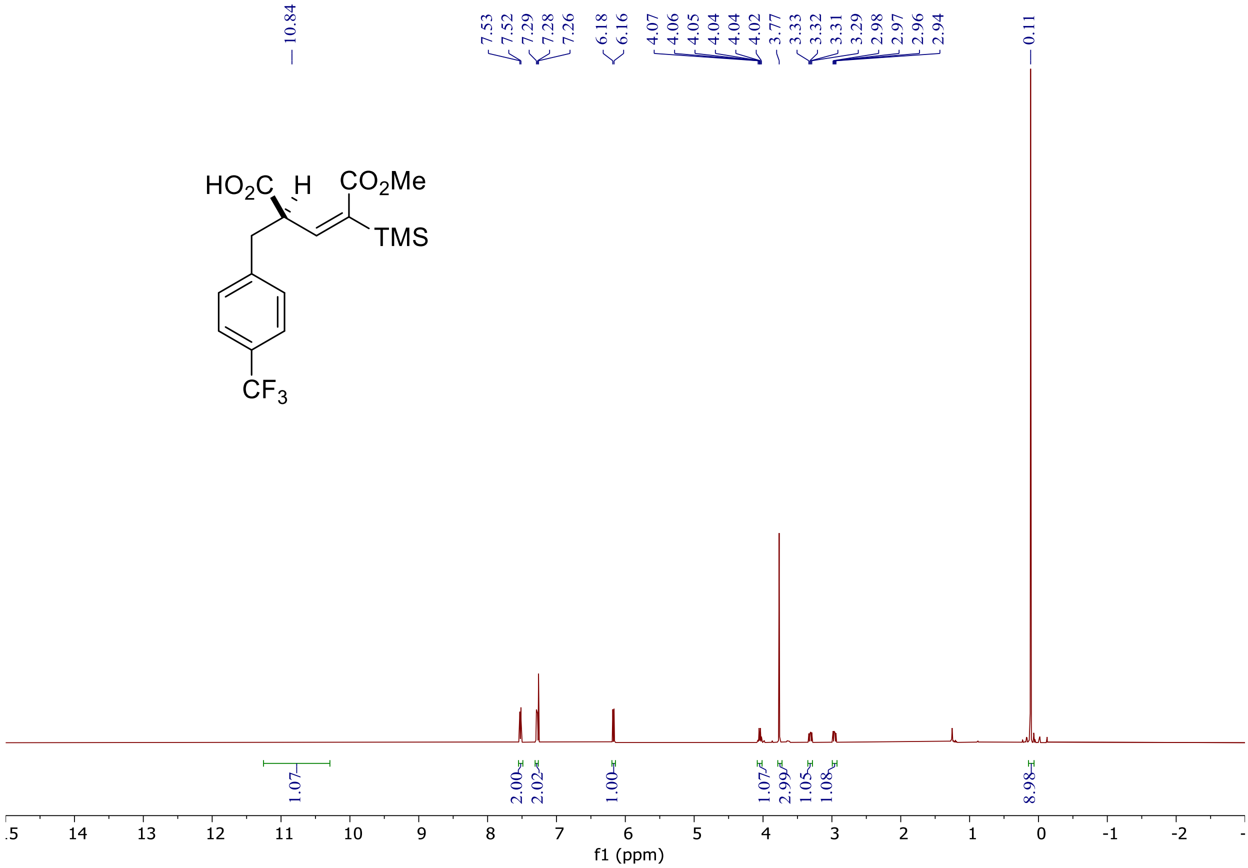

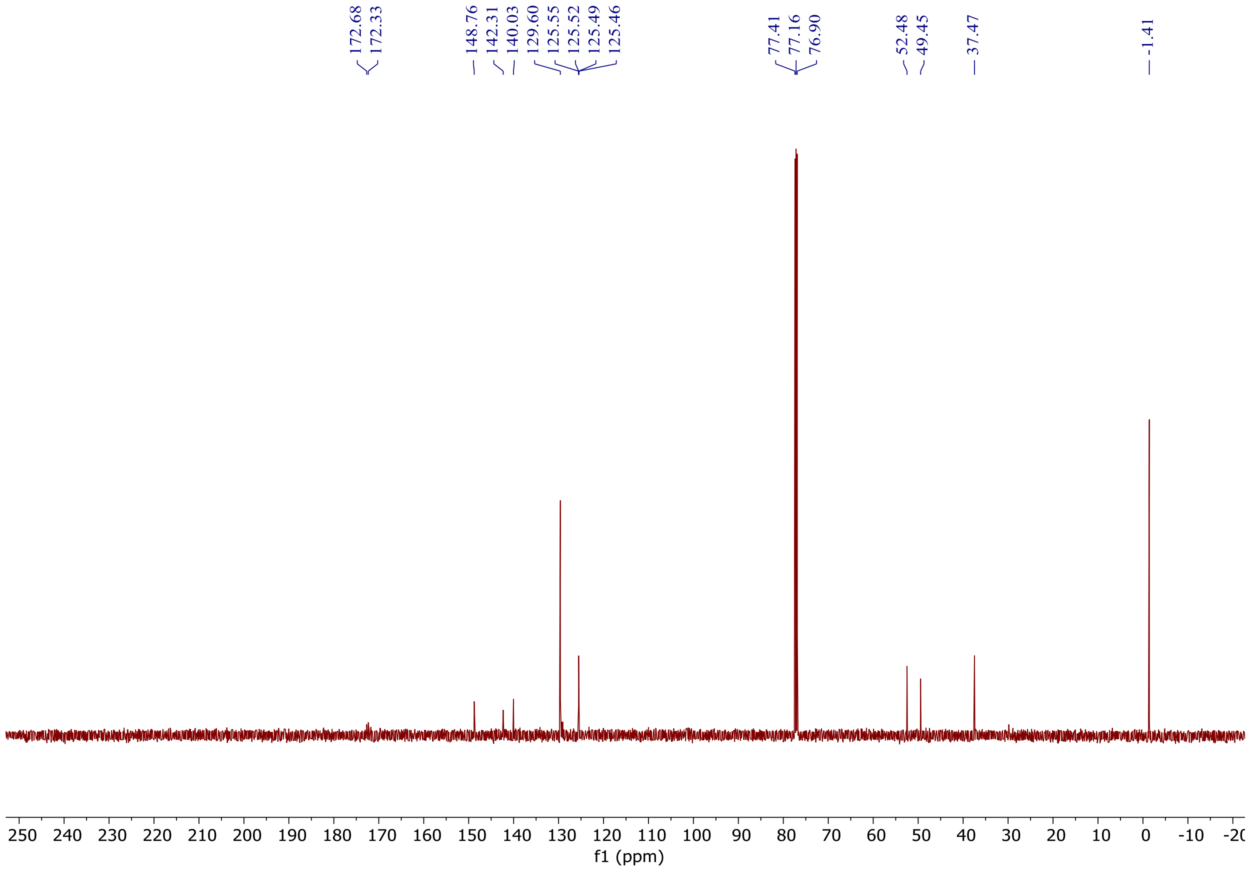

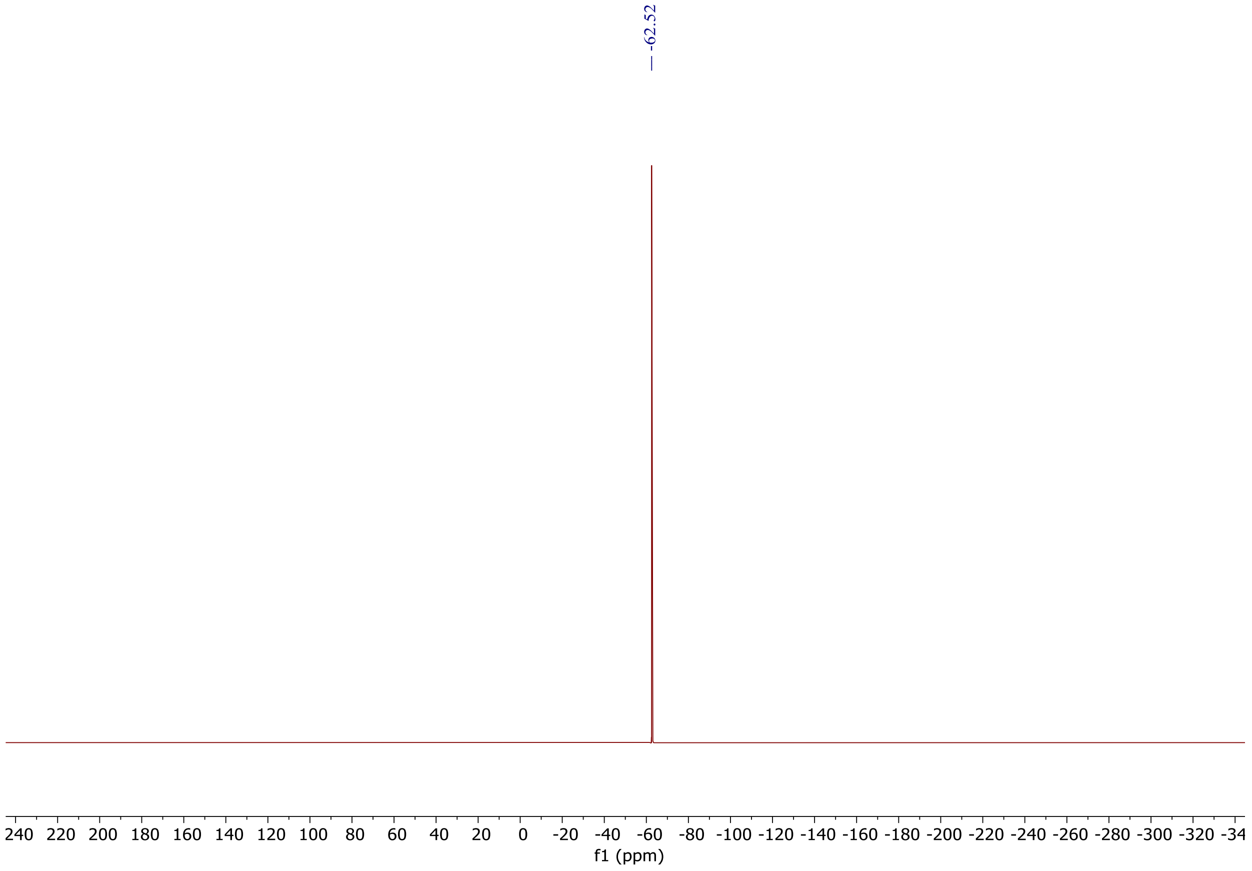
-62.52
240 220 200 180 160 140 120 100 80 60 40 20 0 -20 -40 -60 -80 -100 -120 -140 -160 -180 -200 -220 -240 -260 -280 -300 -320 -34
f1 (ppm)

^{1}H and ^{13}C NMR traces of **s4f**

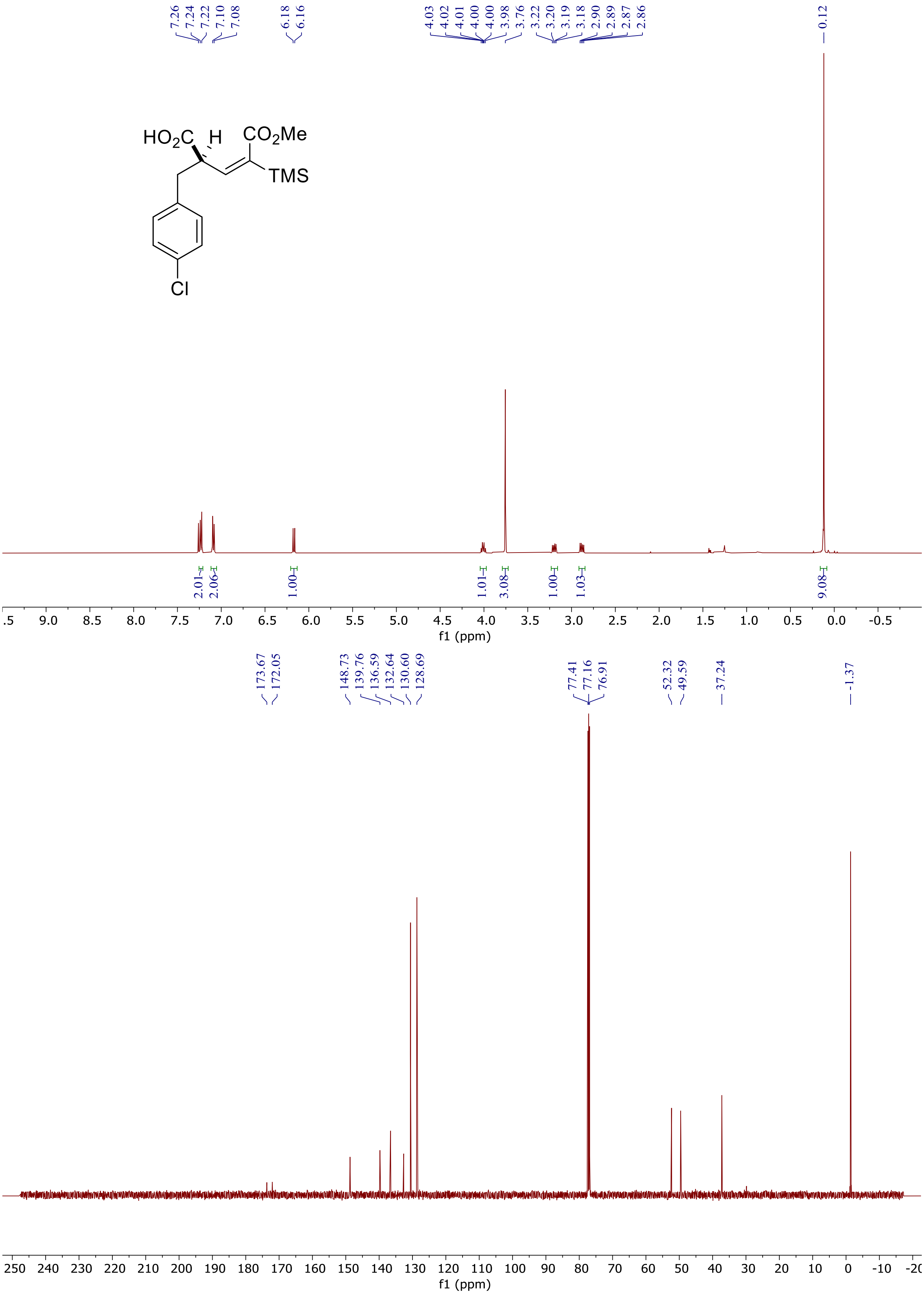

^{1}H and ^{13}C NMR traces of **s4g**

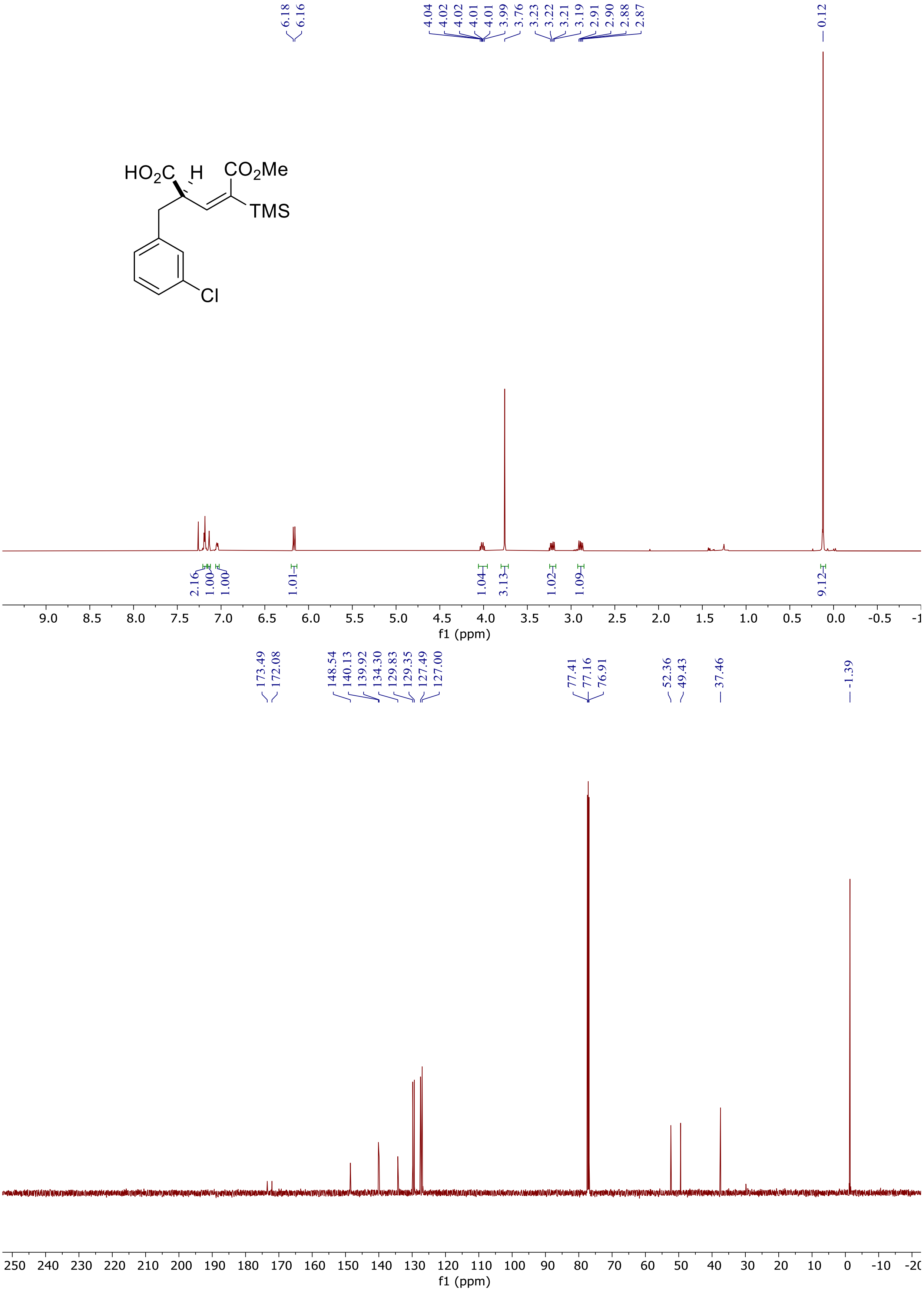

^{1}H and ^{13}C NMR traces of **s4h**

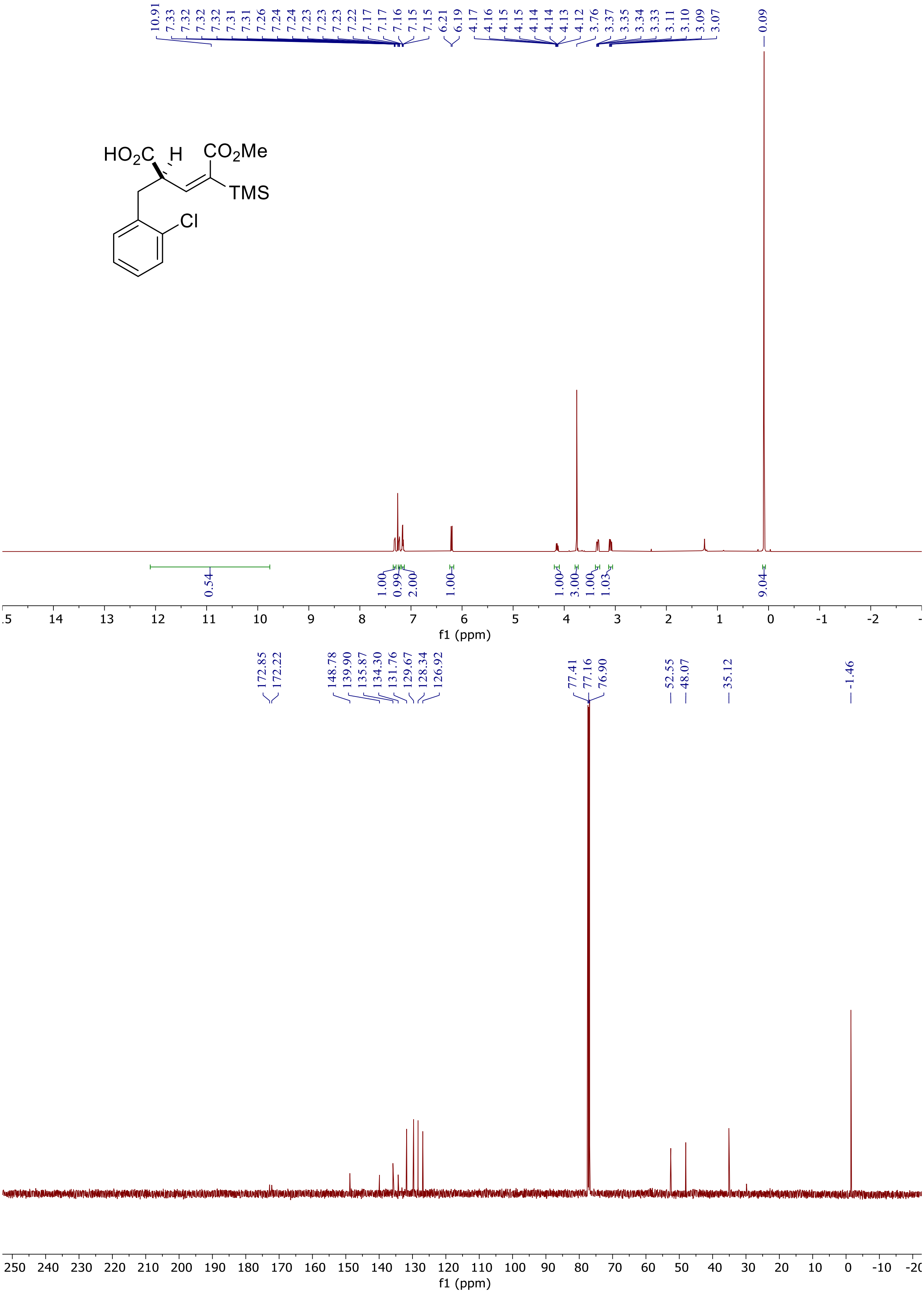

12. Computational Details

All calculations were performed using ORCA 6.0.[8] Preliminary transition state structures were generated using constrained optimizations at the GFN2-xTB[9] level of theory, followed by exploration of the conformational landscape at the same level of theory using GOAT.[10] All resulting conformers were subsequently optimized using constraints at the r2SCAN-3c[11–15] level of theory.

In certain cases, as indicated individually, these conformers were then subjected to a single-point energy calculation at a higher level of theory in order to sort-out high-energy variants (individually mentioned at the XYZ coordinate section).

In the majority of the cases, all conformers were subjected to transition state optimizations, followed by frequency and single-point energy calculations. The Resolution of Identity (RI) approximation[16] in the Split-RI-J[17] variant using a corresponding auxiliary basis set was used.[18,19] Solvent effects were included implicitly using the CPCM[20] or SMD[21] model in methylcyclohexane. Gibbs free energies were calculated using the final single-point energies in combination with thermochemical corrections from the vibrational frequency calculations acquired by using Duarte's otherm.py[22] in a 1 M solution standard state at 213 K.

In order to gain insight about the stereodetermining steps of this transformation, we first dissected the pathway from the starting materials **1p** and **2a** to the silyl ester of product **4p** into two steps: 1) nucleophilic addition and 2) silyl propadienone acetal isomerization. To gauge the energetic profile of the overall transformation, we used the achiral and highly competent anion Tf_2N^-. Upon comparison of Gibbs free energies of the individual steps, we later focused on the computational investigation using chiral enantiopure IDPi **3e**.

12.1. Nucleophilic Addition Catalyzed by $TMSNTf_2$

Based on a Lewis-acidic activation of **2a** by $TMSNTf_2$, we investigated the nucleophilic addition of bis-SKA **1p** leading to both silyl propadienone acetal intermediates **S1a** and **S1b**. Four distinct transition states were identified, two for each diastereomer, clearly indicating the catalytic effect of silylation. Interestingly, in **TS-1** and **TS-2** the formation of the C–C and O–TMS bonds were found to occur simultaneously (Figure S1).

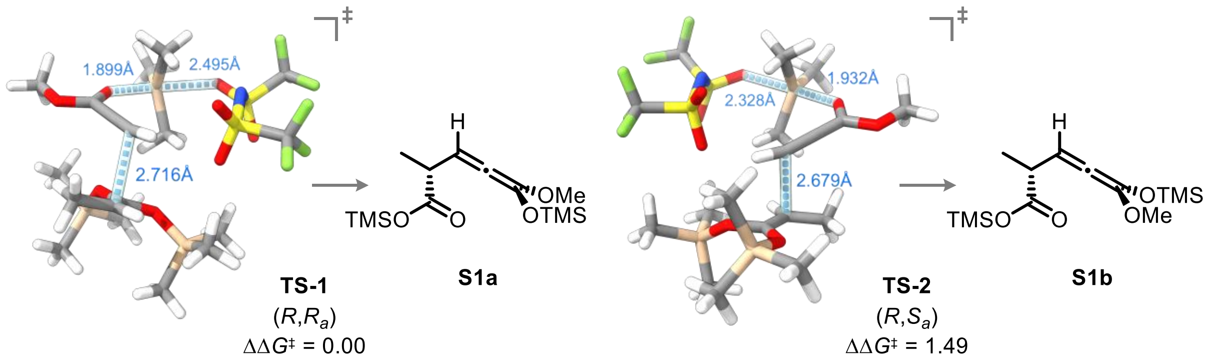

Figure S1. *Comparison of **TS-1** and **TS-2**.*

We applied a Distortion–Interaction Analysis (DIA) to elucidate the key differences, and observed interaction as the dominating factor in the energetic difference between both transition states (see Table S11).

Table S11. *Distortion–Interaction Analysis*[23] *of* ***TS-1*** *and* ***TS-2*** *at the ωB97M-V/def2-QZVP*[24] *level of theory.*

distortion: **–0.89 kcal/mol** ***interaction:*** **2.21 kcal/mol**	complete	propiolate	bis-SKA	TMS^+	Tf_2N^-
**TS-1**	–3627.85328 E_h	–305.23081 E_h	–1085.77777 E_h	–408.94302 E_h	–1827.64636 E_h
**TS-2**	–3627.85118 E_h	–305.22982 E_h	–1085.78000 E_h	–408.94425 E_h	–1827.64531 E_h
(**TS-2**) – (**TS-1**)	1.32 kcal/mol	0.62 kcal/mol	–1.40 kcal/mol	–0.77 kcal/mol	0.66 kcal/mol

Possibly, the *endo*-orientation of the bis-silyl ketene acetal with respect to the electron-deficient propiolate substrate might engage in stabilizing interactions, leading to a computationally observed preference of the (R,R_a)-diastereomer, further aided by the bidentate binding of the anion. Transition states corresponding to a π-Lewis acid-type activation of the triple bond followed by nucleophilic addition of the bis-silyl ketene acetal, which would directly furnish the silyl ester of the final product **4p**, could not be located.

12.2. Silyl Propadienone Acetal Isomerization Catalyzed by $TMSNTf_2$

We subsequently investigated the isomerization of the silyl propadienone acetal to the α-silyl ester. The stereochemical congestion of this molecule required the systematic evaluation of both diastereomers (R,R_a) and (R,S_a) to the corresponding (E)- or (Z)-isomers, resulting in four distinct approaches **TS-3**–**6**. Interestingly, for each mode of stereoselectivity, we were able to locate three different types of transition states, leading to a total of 12 TSs for the isomerization step. More specifically, they differed in the relative stereochemistry of the Tf_2N^- and SKA fragment (apical and equatorial positions of a Si-centered trigonal bipyramid), as well as the *N*- or *O*-coordination of the Tf_2N^- anion. An overview is provided below. The Gibbs free energies are given in relation to **TS-1**.

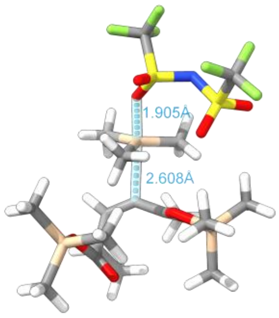

TS-3
(E) from (R,R_a), *O*-TMS, apical
$\Delta\Delta G^‡$ = –22.22

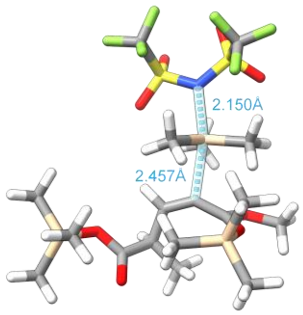

TS-3B
(E) from (R,R_a), *N*-TMS, apical
$\Delta\Delta G^‡$ = –13.64

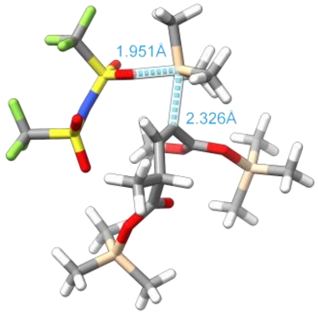

TS-3C
(E) from (R,R_a), *O*-TMS, equatorial
$\Delta\Delta G^‡$ = –9.20

Figure S2. *Comparison of silyl propadienone acetal isomerization transition states (kcal/mol)* ***TS-3***, ***TS-3B***, *and* ***TS-3C***.

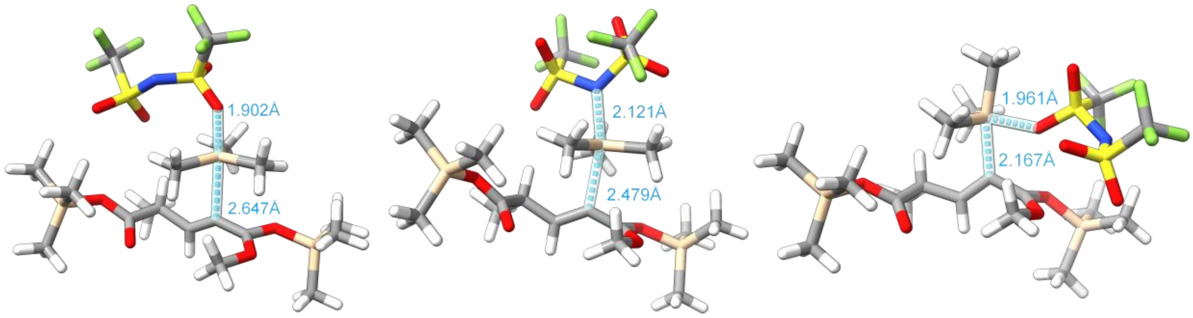

TS-4
(Z) from (R,R_a), O-TMS, apical
$\Delta\Delta G^{\ddagger}$ = –20.81

TS-4B
(Z) from (R,R_a), N-TMS, apical
$\Delta\Delta G^{\ddagger}$ = –15.44

TS-4C
(Z) from (R,R_a), O-TMS, equatorial
$\Delta\Delta G^{\ddagger}$ = –7.51

Figure S3. *Comparison of silyl propadienone acetal isomerization transition states (kcal/mol)* ***TS-4***, ***TS-4B***, *and* ***TS-4C***.

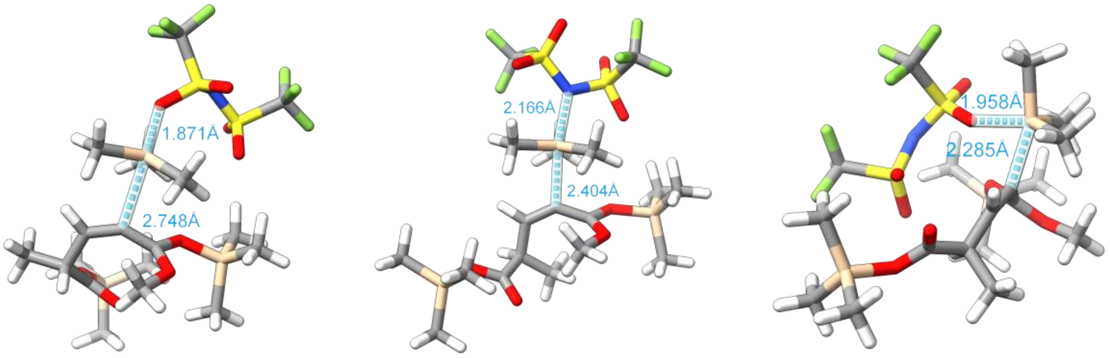

TS-5
(E) from (R,S_a), O-TMS, apical
$\Delta\Delta G^{\ddagger}$ = –21.73

TS-5B
(E) from (R,S_a), N-TMS, apical
$\Delta\Delta G^{\ddagger}$ = –18.30

TS-5C
(E) from (R,S_a), O-TMS, equatorial
$\Delta\Delta G^{\ddagger}$ = –8.69

Figure S4. *Comparison of silyl propadienone acetal isomerization transition states (kcal/mol)* ***TS-5***, ***TS-5B***, *and* ***TS-5C***.

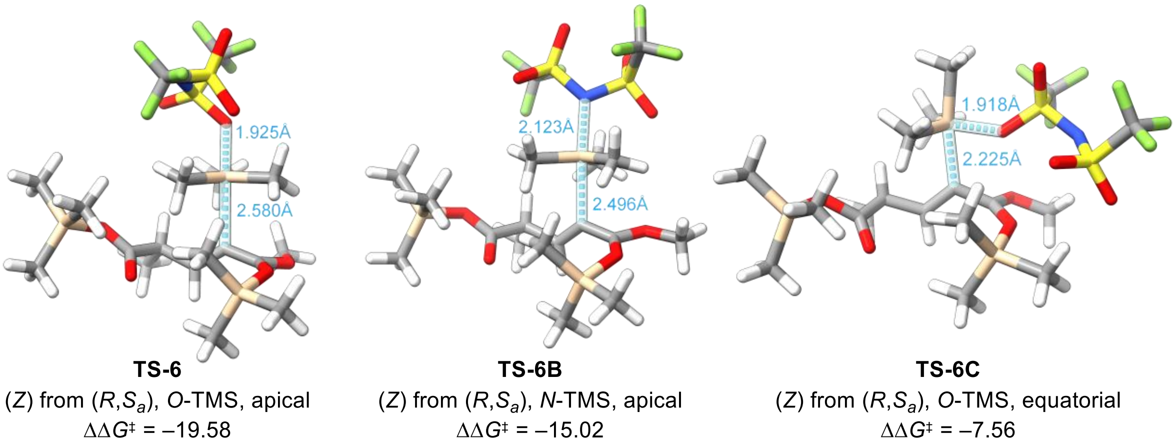

TS-6
(Z) from (R,S_a), O-TMS, apical
$\Delta\Delta G^{\ddagger}$ = –19.58

TS-6B
(Z) from (R,S_a), N-TMS, apical
$\Delta\Delta G^{\ddagger}$ = –15.02

TS-6C
(Z) from (R,S_a), O-TMS, equatorial
$\Delta\Delta G^{\ddagger}$ = –7.56

Figure S5. *Comparison of silyl propadienone acetal isomerization transition states (kcal/mol)* ***TS-6***, ***TS-6B***, *and* ***TS-6C***.

In all cases, the apical transition state with transfer from the oxygen atom of the Tf_2N^- was found to be the lowest in energy. To further elucidate key differences, we again conducted DIA for the four lowest TSs.

Table S12. *Distortion–Interaction Analysis of **TS-3**, **TS-4**, **TS-5**, and **TS-6** at the ωB97M-V/def2-QZVP level of theory.*

overall	**TS-3**	**TS-4**	**TS-5**	**TS-6**
**TS-3**	–3627.89463 E_h	1.75 kcal/mol	–0.03 kcal/mol	–3.02 kcal/mol
**TS-4**	–	–3627.89184 E_h	–1.77 kcal/mol	1.27 kcal/mol
**TS-5**	–	–	–3627.89467 E_h	3.05 kcal/mol
**TS-6**	–	–	–	–3627.88981 E_h
substrate	**TS-3**	**TS-4**	**TS-5**	**TS-6**
**TS-3**	–1391.06187 E_h	1.74 kcal/mol	–0.91 kcal/mol	1.43 kcal/mol
**TS-4**	–	–1391.05911 E_h	–2.64 kcal/mol	–0.31 kcal/mol
**TS-5**	–	–	–1391.06332 E_h	2.34 kcal/mol
**TS-6**	–	–	–	–1391.05959 E_h
TMS^+	**TS-3**	**TS-4**	**TS-5**	**TS-6**
**TS-3**	–408.94399 E_h	0.62 kcal/mol	1.49 kcal/mol	–0.52 kcal/mol
**TS-4**	–	–408.94300 E_h	0.87 kcal/mol	–1.14 kcal/mol
**TS-5**	–	–	–408.94161 E_h	–2.02 kcal/mol
**TS-6**	–	–	–	–408.94482 E_h
NTf_2^-	**TS-3**	**TS-4**	**TS-5**	**TS-6**
**TS-3**	–1827.63574 E_h	0.06 kcal/mol	0.96 kcal/mol	–0.26 kcal/mol
**TS-4**	–	–1827.63565 E_h	0.90 kcal/mol	–0.32 kcal/mol
**TS-5**	–	–	–1827.63422 E_h	–1.21 kcal/mol
**TS-6**	–	–	–	–1827.63615 E_h

Table S13. *Continuation of the Distortion–Interaction Analysis of **TS-3**, **TS-4**, **TS-5**, and **TS-6** at the ωB97M-V/def2-QZVP level of theory.*

distortion	**TS-3**	**TS-4**	**TS-5**	**TS-6**
**TS-3**	–	2.41 kcal/mol	1.54 kcal/mol	0.65 kcal/mol
**TS-4**	–	–	–0.87 kcal/mol	–1.77 kcal/mol
**TS-5**	–	–	–	–0.89 kcal/mol
**TS-6**	–	–	–	–
interaction	**TS-3**	**TS-4**	**TS-5**	**TS-6**
**TS-3**	–	–0.67 kcal/mol	–1.57 kcal/mol	2.37 kcal/mol
**TS-4**	–	–	–0.90 kcal/mol	3.04 kcal/mol
**TS-5**	–	–	–	3.94 kcal/mol
**TS-6**	–	–	–	–

Considering the nucleophilic addition in the previous step to be highly diastereoselective, which is in alignment with the computational data (*cf.* 1.49 kcal/mol in favor of **TS-1** over **TS-2**), a competition between **TS-3** and **TS-4**, which correspond to isomerization from the (R,R_a)-isomer, is plausible. Based on the DIA, it appears as though **TS-4** profits slightly from favorable interactions (–0.67 kcal/mol), whereas there is a clear indication of less distorted substrate (1.74 kcal/mol) and TMS^+ cation (0.62 kcal/mol).

To further delineate these factors, we calculated the second-order perturbative estimates $E(2)$ of selected donor–acceptor interactions in both **TS-3** and **TS-4**.[25]

Table S14. *Second-order perturbation energy estimates E(2) based on NBO analysis of **TS-3** at the ωB97M-V/def2-QZVP level of theory.*

**interacting atoms**	**interaction type**	***E*(2) / (kcal/mol)**	**Σ / (kcal/mol)**
O72–Si32	LP1 → p	26.94	164.86
	LP2 → p	1.30	
	LP3 → p	136.62	

(C2–C3)–Si32	σ → p<br>π → p	1.93<br>39.11	41.04
(C1–C2)–Si32	σ → p<br>π → p	2.96<br>1.40	4.36
(C1–C11)–(C16–O18)	σ → σ*<br>σ → π*	1.99<br>5.23	7.22
O4–(C2–C3)	LP1 → σ*<br>LP2 → π*	9.79<br>64.85	74.64
O5–(C2–C3)	LP1 → σ*<br>LP2 → π*	1.44<br>68.50	69.94
(C1–H10)–(C2–C3)	σ → σ*<br>σ → π*	8.62<br>0.69	9.31
(C1–C11)–(C2–C3)	σ → π*	6.31	6.31

Table S15. *Second-order perturbation energy estimates E(2) based on NBO analysis of* ***TS-4*** *at the ωB97M-V/def2-QZVP level of theory.*

interacting atoms	interaction type	*E*(2) / (kcal/mol)	Σ / (kcal/mol)
O72–Si32	LP1 → p<br>LP2 → p<br>LP3 → p	27.82<br>0.82<br>136.73	165.37
(C2–C3)–Si32	σ → p<br>π → p	1.69<br>40.81	42.50

(C1–C2)–Si32	σ → p<br>π → p	2.94<br>0.09	3.03
(C1–C11)–(C16–O17)	σ → σ*	3.58	3.58
O4–(C2–C3)	LP1 → σ*<br>LP2 → π*	9.09<br>62.45	71.54
O5–(C2–C3)	LP1 → σ*<br>LP2 → π*	1.39<br>63.00	64.39
(C1–H10)–(C2–C3)	σ → π*	9.21	9.21
(C1–C11)–(C2–C3)	σ → σ*	4.34	4.34

In **TS-3**, the silyl propadienone acetal adopts a favorable conformation, leading to significant donation of the σ bond between C1 and C11 into the π* orbital of the neighboring silyl ester. The lack of this interaction in **TS-4** might contribute to the overall higher stability of **TS-3** over **TS-4** (see Fig. S6), which only benefits from donation into the σ* orbital of the anti-periplanar C16–O17 bond.

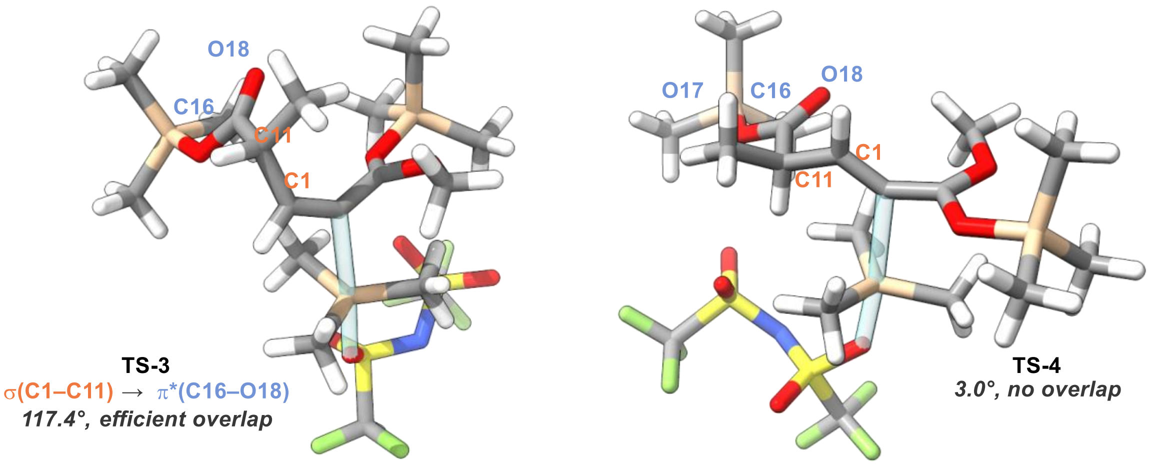

***Figure S6.** Comparison of **TS-3** (left) and **TS-4** demonstrates the kinetic preference of the pre-(*E*)-transition state due to favorable stereoelectronic interactions.*

O-to-*C*-isomerizations of silyl ketene acetals have been shown to be reversible, leading to the need for additional thermodynamic arguments instead of a kinetic preference for the (*E*)-isomer. For this reason, we calculated the final product **4p** and (*Z*)-**4p** at the ωB97M-V/def2-QZVP//r2SCAN-3c level of theory. As expected due to the steric demands of the TMS group, we calculated a thermodynamic preference for the (*E*)-isomer of 2.11 kcal/mol. Consequently, a thermodynamically controlled isomerization of the intermediate silyl propadienone acetal would lead to the same experimentally observed selectivity.

From a stereoelectronic perspective, silylation of the silyl propadienone acetal intermediate requires attack of the TMS^+ fragment to the π orbital. Since either a Lewis acid-induced intramolecular or an uncatalyzed shift would require one of the perpendicularly oriented O-TMS groups to create efficient overlap with the α-position, we predict such a system to be significantly more strained than the intermolecular silylation transition states **TS-3–TS-6**.

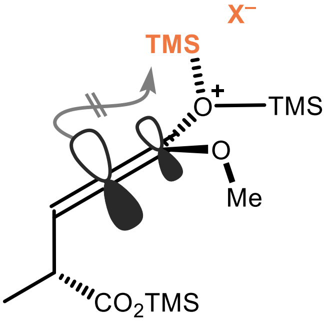

Figure S7. *From a stereoelectronic perspective, intramolecular silylation of the silyl propadienone acetal intermediate requires a system to be significantly more strained.*

12.3. Nucleophilic Addition Catalyzed by IDPi 3e

Based on our model study with $TMSNTf_2$, we predict the nucleophilic addition step to be rate- and enantiodetermining in our catalytic asymmetric reaction with IDPi **3e**. In principle, diastereomers of the intermediate silyl propadienone acetal might interact differently with the enantiopure anion of **3e**, which could influence the observed selectivity *via* kinetic resolution. The excellent enantioselectivities observed in our reaction, combined with the exclusive diastereoselectivities, however, support a highly stereoselective nucleophilic addition followed by rapid downstream isomerization. This is in alignment with the computational data provided below.

Overall, considering the possible formation of both diastereomers of the silyl propadienone acetal, we calculated four distinct transition states for all four possible stereoisomers (R,R_a)- (**TS-1-IDPi**), (S,R_a)- (**TS-1-ent-IDPi**), (R,S_a)- (**TS-2-IDPi**), and (S,S_a)-**4p** (**TS-2-ent-IDPi**) and evaluated the computational stereoselectivity using different methods.

Table S16. *Transition state overview and comparison of different density functionals; structures were obtained at the r2SCAN-3c level of theory.*

method	TS-1-IDPi	TS-1-ent-IDPi	TS-2-IDPi	TS-2-ent-IDPi	er / 213 K
ωB97M-V/def2-QZVP	0.78 kcal/mol	3.75 kcal/mol	**0.00 kcal/mol**	**2.47 kcal/mol**	>99.5:0.5
SMD(MeCy)-ωB97M-V/def2-QZVP	**–0.01 kcal/mol**	2.94 kcal/mol	0.00 kcal/mol	**0.71 kcal/mol**	85:15
CPCM(MeCy)-ωB97M-V/def2-QZVP	0.41 kcal/mol	2.68 kcal/mol	**0.00 kcal/mol**	**1.46 kcal/mol**	97:3

CPCM(MeCy)-ωB97X-V /def2-QZVP	0.27 kcal/mol	2.70 kcal/mol	**0.00 kcal/mol**	**0.96 kcal/mol**	90.5:9.5
CPCM(MeCy)-M06-2X[26]/def2-QZVP	**–0.20 kcal/mol**	3.88 kcal/mol	0.00 kcal/mol	**1.05 kcal/mol**	95:5
CPCM(MeCy)-B3LYP-D4[27,28]/def2-QZVP	**–0.13 kcal/mol**	1.48 kcal/mol	0.00 kcal/mol	**0.50 kcal/mol**	81.5:18.5

Irrespective of the method used, we were able to qualitatively predict the correct enantioselectivity for the reaction. This observation is also conserved for both diastereomers (**TS-1-IDPi** vs. **TS-1-ent-IDPi** and **TS-2-IDPi** vs. **TS-2-ent-IDPi**). Interestingly, in contrast to our results with $TMSNTf_2$, computations now favor the formation of the (R,S_a)-diastereomer of the silyl propadienone acetal, highlighting the confined microenvironment of IDPi **3e**, although to a less pronounced extent (energetic similarity between **TS-1-IDPi** and **TS-2-IDPi**).

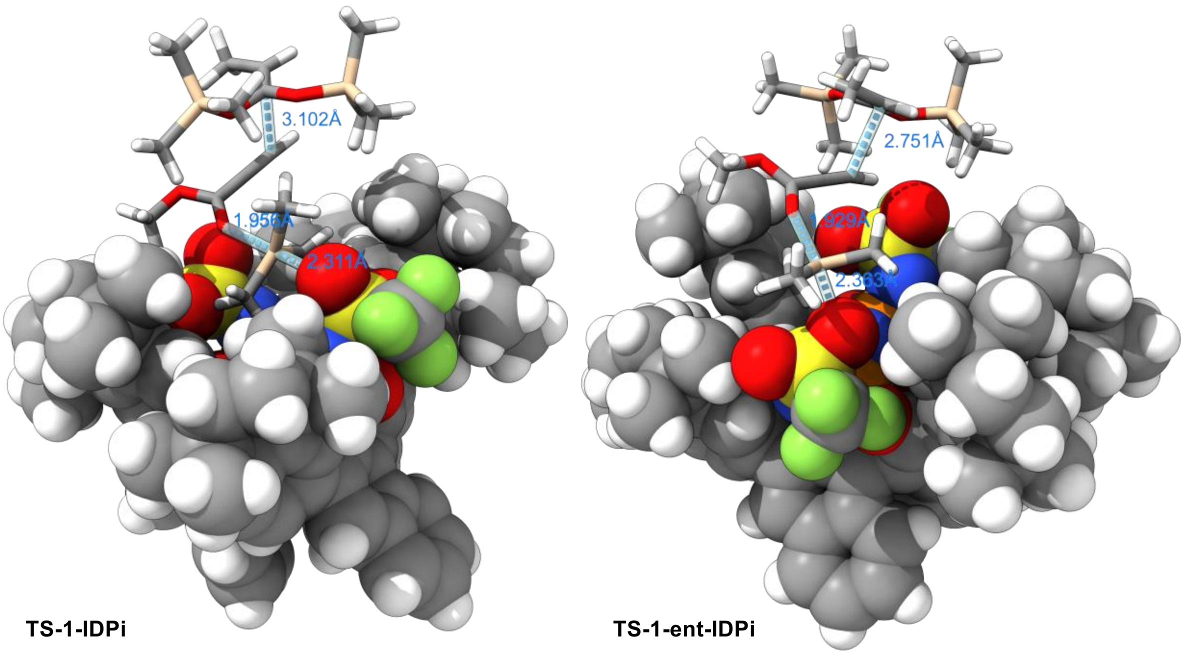

Figure S8. *Structural comparison of* ***TS-1-IDPi*** *and* ***TS-1-ent-IDPi****.*

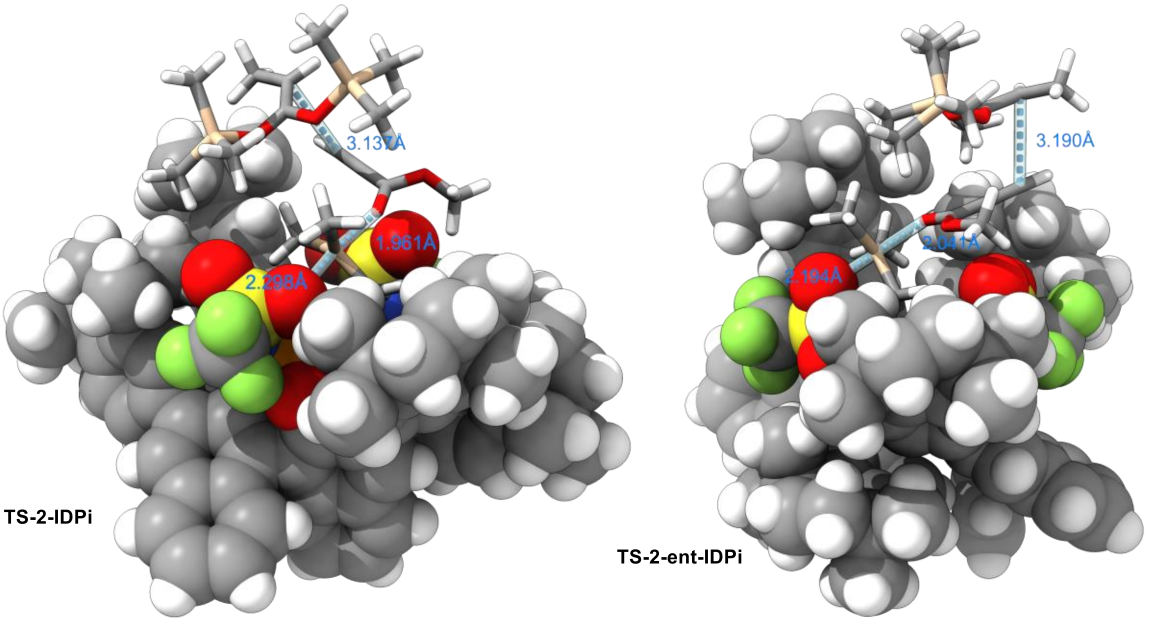

Figure S9. *Structural comparison of* ***TS-2-IDPi*** *and* ***TS-2-ent-IDPi****.*

Again, DIA was performed in order to compare all four transition states. Notably, the degree of silyl transfer slightly differs between the individual transition states. However, this difference is expected to exhibit only a minor influence on the overall structure of the IDPi anion and propiolate (*cf.* **TS-2-IDPi** and **TS-2-IDPi-ent**).

Table S17. *Distortion–Interaction Analysis of four IDPi transition states at the ωB97M-V/def2-QZVP level of theory.*

overall	**TS-1-IDPi**	**TS-1-ent-IDPi**	**TS-2-IDPi**	**TS-2-ent-IDPi**
**TS-1-IDPi**	–8437.58428 E_h	2.94 kcal/mol	–2.72 kcal/mol	1.23 kcal/mol
**TS-1-ent-IDPi**	–	–8437.57960 E_h	–5.66 kcal/mol	–1.71 kcal/mol
**TS-2-IDPi**	–	–	–8437.58862 E_h	3.95 kcal/mol
**TS-2-ent-IDPi**	–	–	–	–8437.58232 E_h

propiolate ***2a***	**TS-1-IDPi**	**TS-1-ent-IDPi**	**TS-2-IDPi**	**TS-2-ent-IDPi**
**TS-1-IDPi**	–305.23676 E_h	3.33 kcal/mol	0.09 kcal/mol	4.36 kcal/mol
**TS-1-ent-IDPi**	–	–305.23145 E_h	–3.24 kcal/mol	1.03 kcal/mol
**TS-2-IDPi**	–	–	–305.23661 E_h	4.27 kcal/mol
**TS-2-ent-IDPi**	–	–	–	–305.22981 E_h

bis-SKA ***1p***	**TS-1-IDPi**	**TS-1-ent-IDPi**	**TS-2-IDPi**	**TS-2-ent-IDPi**
**TS-1-IDPi**	–1085.77991 E_h	–0.41 kcal/mol	0.00 kcal/mol	–1.04 kcal/mol
**TS-1-ent-IDPi**	–	–1085.78056 E_h	0.41 kcal/mol	–0.63 kcal/mol
**TS-2-IDPi**	–	–	–1085.77991 E_h	–1.04 kcal/mol
**TS-2-ent-IDPi**	–	–	–	–1085.78157 E_h
TMS^+	**TS-1-IDPi**	**TS-1-ent-IDPi**	**TS-2-IDPi**	**TS-2-ent-IDPi**
**TS-1-IDPi**	–408.94605 E_h	0.60 kcal/mol	–0.34 kcal/mol	–0.33 kcal/mol
**TS-1-ent-IDPi**	–	–408.94508 E_h	–0.94 kcal/mol	–0.94 kcal/mol
**TS-2-IDPi**	–	–	–408.94658 E_h	0.00 kcal/mol
**TS-2-ent-IDPi**	–	–	–	–408.94658 E_h

Table S18. *Continuation of the Distortion–Interaction Analysis of four IDPi transition states at the ωB97M-V/def2-QZVP level of theory.*

$IDPi^-$ ***3e***	**TS-1-IDPi**	**TS-1-ent-IDPi**	**TS-2-IDPi**	**TS-2-ent-IDPi**
**TS-1-IDPi**	–6637.34771 E_h	–4.21 kcal/mol	–2.38 kcal/mol	1.09 kcal/mol
**TS-1-ent-IDPi**	–	–6637.35441 E_h	1.82 kcal/mol	5.30 kcal/mol
**TS-2-IDPi**	–	–	–6637.35151 E_h	3.48 kcal/mol
**TS-2-ent-IDPi**	–	–	–	–6637.34597 E_h
distortion	**TS-1-IDPi**	**TS-1-ent-IDPi**	**TS-2-IDPi**	**TS-2-ent-IDPi**
**TS-1-IDPi**	–	–0.27 kcal/mol	–2.63 kcal/mol	5.12 kcal/mol
**TS-1-ent-IDPi**	–	–	–2.35 kcal/mol	5.39 kcal/mol
**TS-2-IDPi**	–	–	–	7.75 kcal/mol

**TS-2-ent-IDPi**	–	–	–	–

interaction	**TS-1-IDPi**	**TS-1-ent-IDPi**	**TS-2-IDPi**	**TS-2-ent-IDPi**
**TS-1-IDPi**	–	3.21 kcal/mol	–0.10 kcal/mol	–3.89 kcal/mol
**TS-1-ent-IDPi**	–	–	–3.30 kcal/mol	–7.10 kcal/mol
**TS-2-IDPi**	–	–	–	–3.80 kcal/mol
**TS-2-ent-IDPi**	–	–	–	–

Because of the best alignment with the experimentally observed enantioselectivity (97:3 er, in alignment with computations at the ωB97M-V/def2-QZVP//r2SCAN-3c level of theory by using **TS-2-IDPi** and **TS-2-ent-IDPi**), we chose this method for further evaluation (see Table S17 and S18).

Considering the DIA of **TS-2-IDPi** and **TS-2-ent-IDPi**, a clear dominance of distortion becomes evident. Although **TS-2-ent-IDPi** seems to profit from more interactions (–3.80 kcal/mol), a total of 7.75 kcal/mol of distortion seems to be able to override this preference. This global distortion is furthermore partitioned between the propiolate (4.27 kcal/mol) as well as the anion of IDPi **3e** (3.48 kcal/mol).

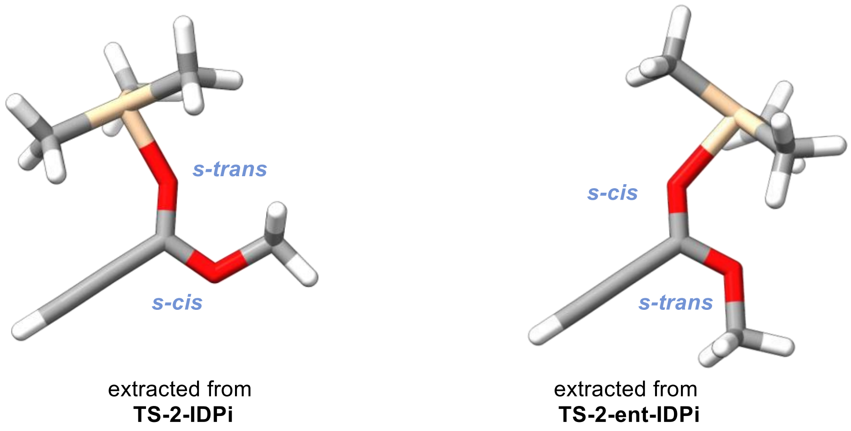

Figure S10. *Structural comparison of the propiolate fragment in* ***TS-2-IDPi*** *and* ***TS-2-ent-IDPi****.*

The alkyl ester functionality in the propiolate substrate is forced into a stereoelectronically unfavorable s-*trans* conformation in the transition state leading to the minor enantiomer. This is ultimately enforced by the s-*cis* conformation of the TMS fragment for steric reasons but might additionally relate to the predominance of favorable interactions in **TS-2-ent-IDPi**, possibly distorting to maximize possible contacts. Interestingly, the methyl ester fragment adopts the more favorable s-*cis* conformation in both **TS-1-IDPi** and **TS-1-ent-IDPi**, again underlining the apparent predominance of interactions resulting from this conformational penalty.

Regarding the differences between the IDPi anion in **TS-2-IDPi** and **TS-2-ent-IDPi**, the reasons for the higher level of distortion are less obvious due to the higher complexity of the overall structure. Nonetheless, based on structural inspection, the anion in **TS-2-IDPi** seems to benefit from π–π interactions of the two BINOL subunits, which creates more degrees of freedom for the structurally demanding 3,3’ substituents. The structural difference between both conformations demonstrates the degree of flexibility that is ultimately responsible for stereodifferentiation.

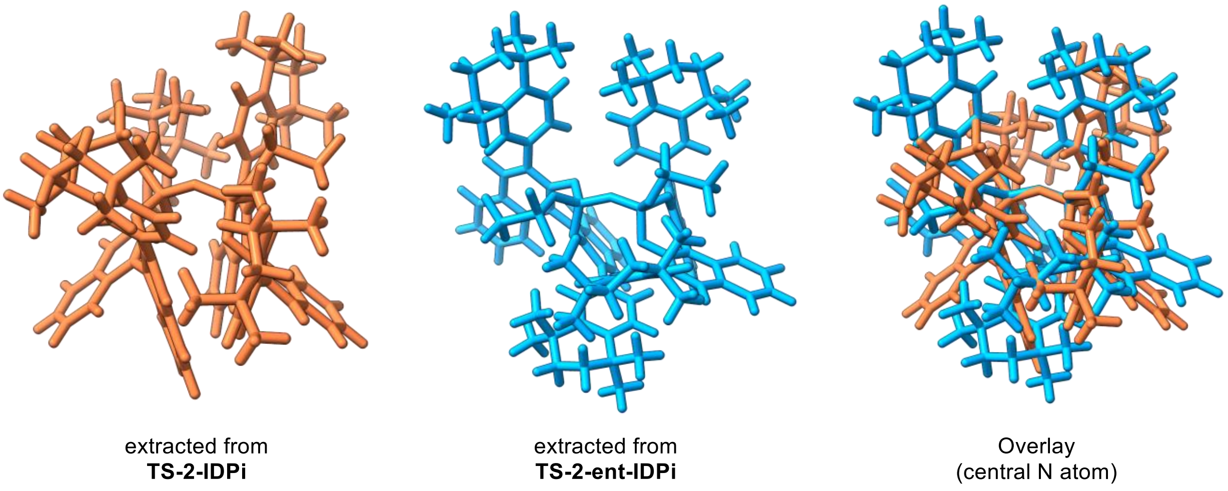

Figure S11. *Structural comparison of the IDPi fragment in* ***TS-2-IDPi*** *and* ***TS-2-ent-IDPi****, as well as overlay of both structures.*

12.4. Thermodynamic Arguments

Besides the kinetic preference of the (*E*)- over the (*Z*)-diastereomer, as clearly shown in the analysis of **TS-3** and **TS-4**, the isomerization of the silyl propadienone acetal might also be reversible, as demonstrated in the literature.[29] As a consequence, the thermodynamically more stable product would enrich selectively. For this reason, we have optimized and compared both diastereomers of the silyl ester of product **4p**. As expected from the configuration of the double bond, we found the (*E*)-diastereomer to be lower in energy by 2.11 kcal/mol.

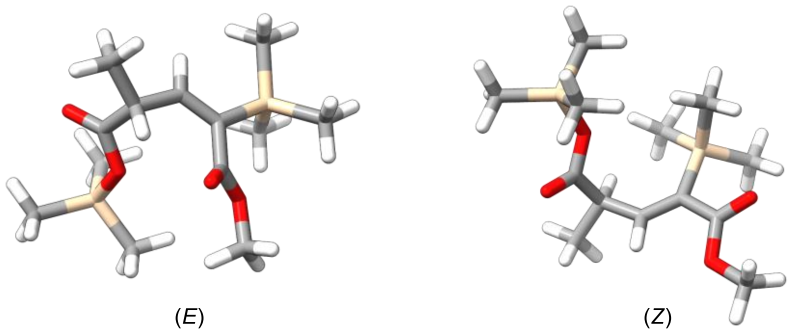

Figure S12. *Structural comparison of the two diastereomers of the silyl ester of product* ***4p****.*

12.5. Activation Barriers

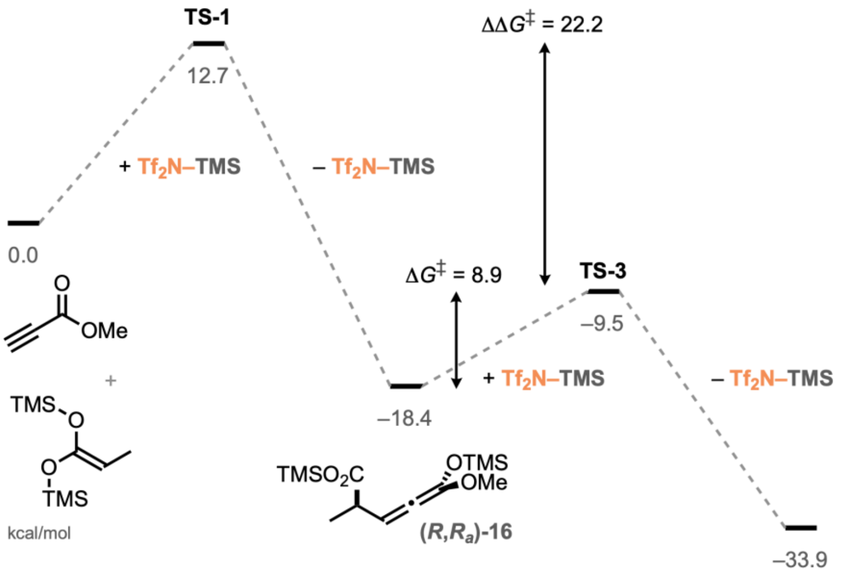

Figure S13. *Obtained reaction barriers for the TMSNTf2-catalyzed reaction, indicating the nucleophilic addition to be the rate-determining step.*

12.6. XYZ Coordinates of Computed Structures

TS-1

36 conformers were generated by GOAT, all of which were used for constrained geometry optimizations followed by transition state optimizations and frequency calculations.

Electronic energy (a. u.) @ CPCM(MeCy)-ωB97M-V/def2-QZVP: –3627.86590806235
Thermochemical corrections (a. u.) @ r2SCAN-3c: 0.485218723108
Imaginary frequencies: one (–44.27 cm^{-1})
Gibbs Free Energy (a. u.): –3627.38068933924

73

C	-1.81786156387159	9.15175162524198	-0.37431098648682
C	-1.83388231329623	9.50668217925968	-1.53545692794456
C	-1.84707135534516	9.81424471439372	-2.89354408746378
O	-2.52166848240718	10.89828694186195	-3.23442730169055
O	-1.23130377733332	9.15334353866248	-3.78399700390222
C	-2.45381152132560	11.30347476092073	-4.62660811844904
H	-3.04737944367940	12.21506236531199	-4.67838132394763
H	-2.87686082622660	10.52658893353139	-5.26697110286793
H	-1.41598385768173	11.49682633055169	-4.90894945166761
C	-4.31475004374103	8.44900129519670	0.43167230914860
C	-4.77671946384849	9.87036989819519	0.38081835875299
H	-5.77806853004923	9.97869682736437	0.81925883526698
H	-4.81431450542069	10.23816374313179	-0.64730028020684

H	-4.10833681204894	10.52678951236845	0.95028340529847
C	-4.26999327801211	7.62160244680408	-0.63846070243060
O	-4.45919056836934	8.09412238478724	-1.88652061405518
O	-3.94663944482610	6.32527045233454	-0.60891403450770
Si	-5.51998030602193	7.49055442061301	-3.05951322885835
C	-7.25500468208081	7.77184740377220	-2.42555115899709
H	-7.43398769501212	8.83106138140105	-2.21195767354999
H	-7.41764348587136	7.20971102565403	-1.49845209194848
H	-8.00530241040348	7.43744000469275	-3.15074577739143
C	-5.23892191889642	5.68573473184666	-3.45025772237063
H	-5.58267613595147	5.03988254089001	-2.63729772391025
H	-4.18077532794477	5.46645347474144	-3.62241079794876
H	-5.79606300605704	5.41302794132155	-4.35503022299886
C	-5.10127047206682	8.56287995082961	-4.53236548369639
H	-4.07658339730799	8.35938808549303	-4.86650386765081
H	-5.17828858901805	9.62446534827557	-4.27239892876742
H	-5.77199807539174	8.36779552327187	-5.37597740389182
Si	-4.28570952650990	5.10881163096354	0.53181130237489
C	-6.15269571622819	4.91606830230482	0.54792656801123
H	-6.63545540904507	5.85979302521268	0.82931849440624
H	-6.45257775506577	4.16402653440115	1.28700016393636
H	-6.55016246634256	4.60373969360857	-0.42357014387729
C	-3.70767088282422	5.51658236255969	2.26299197626780
H	-2.70551601893236	5.95357315054509	2.25713983064879
H	-3.65379042073414	4.58490766484921	2.84004421612919
H	-4.39651351124852	6.18550429448461	2.78945117680644
C	-3.40514684641764	3.63724331347067	-0.18421489577933
H	-2.32554103464861	3.82069957400615	-0.21755911504086
H	-3.74733128558296	3.43459450977667	-1.20503071850718
H	-3.58295762455862	2.73906869402449	0.41759917257988
H	-4.07517617267907	8.01445566530017	1.39660668402899
Si	-0.06700830054665	7.69440214812712	-3.43635120267123
C	1.17814522645923	8.66360120600486	-2.43945370965221
H	2.17015592424669	8.22779498206182	-2.58005706806563
H	0.97360041392930	8.66339994426697	-1.36735460218850
H	1.19790571553915	9.70065260680473	-2.79525794719034
C	-1.37413743812770	6.51733388580303	-2.82600799809332
H	-1.38720642436316	6.37941691115548	-1.74232653291176
H	-1.21179304828021	5.53554260753761	-3.28025916414745
H	-2.36359879288393	6.88300891742210	-3.11702727202000
C	0.42552087665802	7.43550526494943	-5.21283606565434
H	-0.45508445818320	7.23836913222801	-5.83378086834161
H	1.10146589276053	6.58048607059464	-5.29522964916670
H	0.93604279728087	8.31842296589774	-5.61199933501176

O	0.54460162187350	7.76585534465990	1.36648926541346
S	0.38829307454391	6.40750300308031	0.82468641879699
O	-0.97349545337046	5.93194092603766	0.51959266438793
C	0.98493598877935	5.26897031406774	2.22196060438380
F	2.22779126571424	5.59234242093805	2.59078875246926
F	0.96375452144089	3.99310919453671	1.82251727956348
F	0.16659054718642	5.40625458579643	3.27710404063004
N	1.48004285187429	6.22650321358059	-0.35443782592398
S	1.37168187361230	5.10350809020151	-1.51623801905469
C	3.11117127091428	4.35491356740659	-1.39331518015204
F	4.04455342655926	5.29360998781238	-1.57422956430455
F	3.24595426256468	3.41734517027273	-2.33926299273828
F	3.28007744706528	3.79170728401652	-0.19339730230179
O	0.42765051755988	3.99624229084826	-1.33732799316452
O	1.41544036955166	5.77635681579406	-2.84774684421823
H	-1.57858601289769	8.87711295104113	0.62990451498611

TS-1B

12 conformers were generated by GOAT, all of which were used for constrained geometry optimizations followed by transition state optimizations and frequency calculations.

Electronic energy (a. u.) @ CPCM(MeCy)-ωB97M-V/def2-QZVP: –3627.84550899668
Thermochemical corrections (a. u.) @ r2SCAN-3c: 0.48416969712
Imaginary frequencies: one (–272.63 cm^{-1})
Gibbs Free Energy (a. u.): –3627.36133929956

73

C	-3.25839332528509	4.90932357486698	-1.35868980087337
C	-2.39519305333391	5.58832567258847	-0.76330091400365
C	-1.68105113833678	6.67534475870515	-0.25380946401558
O	-2.01724817606671	6.97778704637399	1.03478792303142
O	-0.77904392439123	7.27392125698638	-0.85037524197679
C	-1.19440833384574	7.98711974297738	1.65110967576341
H	-0.14673251261845	7.67029501992948	1.67587387916199
H	-1.58346673067430	8.09356840777595	2.66506812965638
H	-1.26143744591251	8.93510990577671	1.11091998078465
H	-3.48919883893788	3.87976276989206	-1.58421652031983
C	-4.86464657238196	5.70830445718239	-2.27334062220242
C	-5.23483902026015	4.65407723258172	-3.27906737738801
H	-6.06844861691399	4.98280511921681	-3.91033471048227
H	-5.53713163247688	3.72464608782486	-2.78783062863745
H	-4.38320646305853	4.44355210365114	-3.93330948579800
C	-5.55930263135895	5.84265747413807	-1.08256035100047

O	-6.24624922261104	4.82105772498824	-0.58704428575395
O	-5.41282741329919	6.88526340079791	-0.27572875968707
Si	-6.45110903093748	4.46082281619957	1.07893336147176
C	-4.78837540353481	4.47114445843786	1.91712920868550
H	-4.30003919304890	5.44761178926299	1.83357201101265
H	-4.11302866341567	3.73110856400542	1.47573031821419
H	-4.90529482705858	4.23319481627218	2.98137493636628
C	-7.20496573584171	2.75578827709110	0.99986414347580
H	-6.53521783599005	2.05140285658033	0.49539415902283
H	-8.15535460996766	2.76840392810458	0.45526044893350
H	-7.40074722494149	2.37102092210952	2.00693320701696
C	-7.63901215873511	5.67127871377543	1.86439479866706
H	-8.50039920290919	5.86545722420703	1.21627662029407
H	-7.15128004192062	6.62531447888556	2.08432484457759
H	-8.01824212433804	5.26175372123805	2.80819716244846
Si	-5.76869868468967	8.54385743747698	-0.52134100598095
C	-4.80393266754693	9.37514548115114	0.83038955090020
H	-5.00454431640972	10.45205368085416	0.84768404298961
H	-3.73181357908722	9.23491927934830	0.66592656138438
H	-5.06427194602204	8.96150335902324	1.81089098406854
C	-7.62035228427761	8.71801816590725	-0.29729541670850
H	-7.93994875566720	8.50389752586069	0.72686282893866
H	-8.16216400237152	8.04252476125537	-0.96988748915198
H	-7.93207359647198	9.74091671100962	-0.53919637062614
C	-5.30260411755996	9.10880289540570	-2.23189220395325
H	-4.23985095661431	8.96625808879953	-2.44108701040428
H	-5.50759155426831	10.18444639634121	-2.30452210004803
H	-5.89358730720119	8.60670337546470	-3.00572962023330
H	-4.40153921044799	6.61247677658565	-2.65085239312082
Si	1.46279492581839	6.37166721219671	-2.93047475185171
C	2.54484310910208	6.06196148981693	-4.42250788286894
H	3.49970035080346	5.62022883038368	-4.11418909743058
H	2.76448192759603	6.99351526210982	-4.95510019523817
H	2.06823488813249	5.37098051483782	-5.12598631094537
C	0.99516487078600	4.77618042453617	-2.10395144045168
H	0.27375260235155	4.93916997274665	-1.29891946781564
H	1.88920041002661	4.29748705783315	-1.68667820006506
H	0.54977261933322	4.08374647002645	-2.82631020486448
C	2.21379626558577	7.65552732725241	-1.82313123507898
H	1.54872817119130	7.93450308159858	-1.00322771862578
H	2.48362925557824	8.56076640826921	-2.37478111611552
H	3.13447809185196	7.23011863790483	-1.40239153462265
O	-0.01116500853707	6.91244676239354	-3.71240698840525
S	-0.94058676042885	8.16677813443230	-3.68165499295129

O	-2.33842955486470	7.80849029143963	-3.48784552218825
C	-0.74449126079467	8.60901005600111	-5.51981155008156
F	-1.17239614738719	7.58734441015556	-6.26400371810332
F	0.53572433304095	8.85885255294430	-5.80402068522863
F	-1.47901079136647	9.68887572329571	-5.78230108964532
N	-0.21665880871707	9.35741562073311	-2.97522966122974
S	-0.84503425232426	10.25142166989092	-1.72493430543095
C	-0.98437903341495	11.90988596057785	-2.63913959351431
F	-1.43015186230477	12.82871465576063	-1.77549395051264
F	-1.85341001177901	11.80480491411640	-3.65167427178076
F	0.20184606831974	12.29342830911953	-3.11576179953937
O	-2.21776538034758	9.91566002639793	-1.34211051997551
O	0.20070505935498	10.44634999485385	-0.72599922888934

TS-2

35 conformers were generated by GOAT, all of which were used for constrained geometry optimizations followed by transition state optimizations and frequency calculations.

Electronic energy (a. u.) @ CPCM(MeCy)-ωB97M-V/def2-QZVP: –3627.86316609611
Thermochemical corrections (a. u.) @ r2SCAN-3c: 0.484846646536
Imaginary frequencies: one (–75.42 cm^{-1})
Gibbs Free Energy (a. u.): –3627.37831944957

73

C	-3.58793177818416	5.59977696549622	0.72142659872489
C	-2.50093077104309	5.23480256732440	0.31206003973695
C	-1.19102277792586	5.07272973643624	-0.13283726818812
O	-1.00782320749650	4.20520318126250	-1.11753576195458
O	-0.19778927350191	5.68506619674475	0.36104095757046
C	0.35596163808742	4.01364796196988	-1.57037937566879
H	0.75863230108407	4.95191199433561	-1.96009921206082
H	0.28458455214301	3.26501587960542	-2.35798515261249
H	0.98012097220066	3.65629632911606	-0.74767035473579
C	-4.36515467483186	7.50474994879280	-0.99490745444311
C	-3.63592152937373	6.97631874925333	-2.17788291758187
H	-2.62264731178987	6.65031675593859	-1.89429636497291
H	-3.51213049994597	7.74562384913983	-2.95070803804911
H	-4.15116405186539	6.11967737772023	-2.61851931074449
C	-5.58975251664133	7.08408136222327	-0.59069049223109
O	-6.20734129436698	6.04921193376557	-1.16778478989155
O	-6.24620028067541	7.59769299867630	0.46143640847318
Si	-7.56146662448583	5.21444969056611	-0.55242006966327
C	-7.72596313334906	3.80937179739392	-1.76984029747672

H	-7.89623469216848	4.18377783976982	-2.78497620519342
H	-8.56880780730364	3.16136854961094	-1.50477096879187
H	-6.81995893754985	3.19416032802813	-1.78430674405000
C	-9.07566771251354	6.31023045144456	-0.60129476717607
H	-9.16258166679304	6.81592619818415	-1.56938937587714
H	-9.04820161339506	7.07164625279717	0.18311472627459
H	-9.98166716162248	5.70978646489409	-0.45686908363363
C	-7.20324453842090	4.58677320044479	1.17020980383768
H	-7.02915029840888	5.40408723215639	1.87754735708879
H	-6.32804782836247	3.92748803522571	1.18342868962454
H	-8.05432903925720	4.00264050777252	1.54039793750020
Si	-6.53967515907683	9.24298656942176	0.82289804679079
C	-7.60393193256162	9.14377015458038	2.34600064815680
H	-7.03256024819620	8.70927195500584	3.17210068012055
H	-8.49432907474218	8.52786349674798	2.18229484548595
H	-7.93573161084546	10.14233723872238	2.65140175351787
C	-7.44829083378631	9.93285422635928	-0.66256989581640
H	-7.64486623059952	11.00308830822248	-0.53209984992763
H	-8.40736088262251	9.43028441458104	-0.82543334973501
H	-6.84524997464739	9.81679511742127	-1.57088299802557
C	-4.98852785773737	10.23267446200295	1.13755263168259
H	-4.43747598835208	10.45856154807155	0.21887756068181
H	-4.32149461305131	9.73753211312101	1.84931041292644
H	-5.28130278916293	11.19264211037057	1.58209459398196
H	-3.93815423185768	8.33667589758774	-0.44662968412922
Si	-0.27187747042385	6.77222325017293	1.95601691738159
C	1.53839046871043	7.20808337111942	1.88796537049962
H	1.80304212155551	7.89789158239822	2.69262027123363
H	1.78039208479787	7.68415330158526	0.93158789010290
H	2.15691071191094	6.30952008716984	1.98536554055746
C	-1.40842290009850	8.07553789738146	1.23861817098565
H	-1.01571479407242	9.06296534194488	1.49833764135881
H	-2.42768486288346	8.01380827832982	1.62876584249394
H	-1.45554542465587	7.99159057875515	0.14788056482686
C	-0.68142961725330	5.31272713096170	3.05106764438070
H	-1.74923493165663	5.09186025936644	3.10991772837869
H	-0.33316743551006	5.49819232223587	4.06906057243769
H	-0.16532596416641	4.42817865570625	2.65943185257528
O	-0.45004646189058	8.06187098717978	3.88621954496389
S	-1.75087656648709	8.43365976791572	4.53588383219501
O	-2.44100023720209	9.62460569137771	4.03238500643996
C	-1.18419944044880	8.84121798661365	6.30048603479219
F	-0.38277045567585	9.91160645526066	6.26006345599721
F	-0.51909497238703	7.81551004817712	6.83616448059697

F	-2.24972932828945	9.12061220284889	7.05620338607507
N	-2.59967139980617	7.09317537855543	4.81345199394787
S	-4.10244130869787	6.83453709657049	4.26219982944539
C	-5.21711186285141	7.47993360640355	5.65624002534845
F	-6.49759748286102	7.21121973151132	5.35520892432042
F	-5.07321314430371	8.80356141760734	5.78828397896286
F	-4.91044622213674	6.88732091848298	6.81284674356998
O	-4.49368070712062	7.62248016482885	3.07784824328196
O	-4.32388913360425	5.38511003409184	4.24379575019489
H	-4.46949025254053	5.76759848529537	1.30330885990931

TS-2B

15 conformers were generated by GOAT, all of which were used for constrained geometry optimizations followed by transition state optimizations and frequency calculations.

Electronic energy (a. u.) @ CPCM(MeCy)-ωB97M-V/def2-QZVP: –3627.83872298963
Thermochemical corrections (a. u.) @ r2SCAN-3c: 0.482661260917
Imaginary frequencies: one (–249.02 cm^{-1})
Gibbs Free Energy (a. u.): –3627.35606172871

73

C	-3.51197457413165	4.43260891374309	-1.03210324829421
C	-2.35800665451263	4.76494106014847	-1.35947176983051
C	-1.31779536583032	5.61768029412817	-1.77364390266262
O	-0.79605984049542	5.44162397154374	-3.02090122092743
O	-0.80848991386661	6.47820179518135	-1.05767713360160
C	-1.38034883624399	4.43199436308611	-3.86314493648772
H	-1.31719601837829	3.44821960984503	-3.38536118624377
H	-0.79499302806704	4.44696447587997	-4.78364720834632
H	-2.42450575535151	4.67255965018996	-4.08496698177186
H	-4.10519097716671	3.64363676196074	-0.60362341737696
C	-5.12724095928689	5.68530622101407	-1.56728599890557
C	-5.77656323593393	4.80981670612581	-2.60450800242014
H	-6.08848213710693	3.84893095616273	-2.18487953196450
H	-5.07422350706311	4.62215605003149	-3.42091681165695
H	-6.66511235183691	5.29210648409913	-3.03093239620435
C	-5.64883540314484	5.80629595002665	-0.30175608730677
O	-6.46929557086940	4.86673606060557	0.16486042564700
O	-5.32976626967061	6.73666027188268	0.59230227764445
Si	-7.14262345814927	4.78698872997320	1.73347939625676
C	-8.10702375462091	3.19242199749334	1.65004215904142
H	-7.44486283008265	2.33866739310195	1.47005765836242
H	-8.84569314684342	3.22201617109107	0.84203258347822

H	-8.64168142767970	3.01024155518413	2.58885098254119
C	-8.26615296088328	6.25608739511319	1.99169377048485
H	-8.79901148002628	6.16742355429755	2.94565559487286
H	-9.01631227173404	6.32093218967267	1.19601407615805
H	-7.69967407347388	7.19215297384974	2.00476196183937
C	-5.79131177560387	4.69510833760794	3.02090799117767
H	-4.99804471761496	4.00479549661966	2.71292528347671
H	-6.19790613679164	4.33205351182816	3.97216943123109
H	-5.33694365676293	5.67420374827886	3.19626965743226
Si	-4.10875137805218	7.92171629503220	0.78619890342178
C	-4.98900953372009	9.16307297333989	1.87294678165522
H	-4.31337595213450	9.98077878749718	2.14823246019288
H	-5.34326384675606	8.69899303066635	2.80018630288583
H	-5.85228570008836	9.60116377752324	1.36001652940387
C	-3.58693651419543	8.69429319718405	-0.82706026542470
H	-4.43428769443414	8.96562079157599	-1.46541671238838
H	-2.91108617297217	8.04775881791770	-1.39444162516682
H	-3.03850754335052	9.61775385731613	-0.60169567151234
C	-2.69039993948294	7.11586884501125	1.68100424140220
H	-2.21576554227800	6.35380185909641	1.05561912848307
H	-3.01409353631715	6.66322783727584	2.62441952776991
H	-1.92405987235563	7.86650526663672	1.91030322520618
H	-4.55156126735904	6.52589209128773	-1.92953800975928
Si	1.29832763544207	8.83535388506886	-2.51083377736382
C	2.17310387893835	7.21547000607803	-2.30672088552507
H	3.04535595639303	7.35486668137355	-1.65616824261872
H	2.52314208138577	6.83249764441691	-3.27024933162535
H	1.50413627691960	6.48205824181997	-1.84964726537810
C	0.46310248604760	9.50075523897505	-0.99428374023620
H	-0.21467634984451	8.74552214532689	-0.58636362794785
H	-0.10249998975566	10.40958598677003	-1.22574685227134
H	1.21183203102049	9.74949861122400	-0.23307162063868
C	2.39522998551570	10.10385736479609	-3.33312838421459
H	2.82983339740489	9.72747224364730	-4.26505673304797
H	3.22742091123285	10.33861577918631	-2.65805154672809
H	1.86864005759664	11.04001374289856	-3.54448802490322
O	-0.08846377373870	8.60904225411854	-3.58491264088062
S	-0.10690181559133	8.19294854809757	-5.09261095852029
O	1.08955113312076	7.49746800764897	-5.54757925628029
C	-0.07528742936406	9.93924719961926	-5.95293654589042
F	1.16782543292034	10.16203046528950	-6.38411692265401
F	-0.42857879573971	10.90340635421465	-5.11064908470706
F	-0.90268315695721	9.91276674237549	-6.99263152237319
N	-1.42926140855820	7.43935019030056	-5.45331290336191

S	-2.90344119697831	7.97823812749154	-4.90274634721989
C	-3.87938593453114	7.65959091886404	-6.50175815730783
F	-5.15763685236356	7.96978765237589	-6.26117014452744
F	-3.79561951559543	6.37287641536074	-6.84617005598624
F	-3.42194227988925	8.41832149219209	-7.49767607980514
O	-3.46050536170407	7.03348369114667	-3.93374176788155
O	-2.94440084816509	9.42207429268882	-4.65071577715209

TS-3

89 conformers were generated by GOAT, all of which were used for constrained geometry optimizations followed by transition state optimizations and frequency calculations.

Electronic energy (a. u.) @ CPCM(MeCy)-ωB97M-V/def2-QZVP: –3627.90512588146
Thermochemical corrections (a. u.) @ r2SCAN-3c: 0.489025679174
Imaginary frequencies: one (–112.48 cm^{-1})
Gibbs Free Energy (a. u.): –3627.41610020229

73

C	-2.46901935014189	6.42379818501203	0.87003896540866
C	-2.63225613774325	6.06133954041810	-0.38966955366039
C	-3.64354953556543	5.98267398438505	-1.31025809701862
O	-3.85071752011625	6.88358550383875	-2.28170074052548
O	-4.34831804657855	4.88902036324603	-1.45400507605487
C	-3.10118657991663	8.11289916141819	-2.21956788975732
H	-2.04522875917476	7.90173304131133	-2.02392737256105
H	-3.49639860027096	8.75763830253960	-1.43057154374668
H	-3.22974842968771	8.58178416037143	-3.19549103943112
H	-1.50851704500416	6.26526601811996	1.36054778755835
C	-3.58815770387884	6.93816686277478	1.77662656654567
C	-4.41705653974401	8.06853489264556	1.18165871445487
H	-4.91326403762630	7.74392722138780	0.26212856679402
H	-3.77850238474360	8.93050023357689	0.96631890074462
H	-5.20192760185287	8.37675584969460	1.87761325653840
C	-4.46993756001558	5.74184719379412	2.12146058219978
O	-3.79419552675425	4.79204944565878	2.78355751152011
O	-5.63925420524904	5.64753692248485	1.81295860450192
Si	-4.53927321590428	3.33592125824224	3.32327356491458
C	-5.76552261144101	3.78578734223107	4.65735630007322
H	-6.20600757396929	2.88380860162645	5.09752860475689
H	-6.57726027384290	4.39373004822924	4.24557818444053
H	-5.28572640929113	4.35069320159588	5.46401132812079
C	-3.08028438124311	2.38756079266527	3.99492553209033
H	-2.35391437039915	2.17896496819357	3.20164653832230

H	-3.39914870516398	1.42758866729689	4.41600864871284
H	-2.56886352678729	2.94866900405701	4.78425363651778
C	-5.34534229093020	2.44242708165931	1.89899454690906
H	-4.65810717061949	2.33308270687912	1.05305975884147
H	-6.23061991442689	2.98570999836274	1.55777630234127
H	-5.65172422915731	1.43653250772516	2.20986555868540
Si	-0.94744299683827	4.28104602219286	-1.27989445254961
C	-2.01146181921205	3.11654197447967	-0.26711937053328
H	-2.68435949385830	2.55091790416746	-0.91776297015751
H	-2.60262837386475	3.63880238182046	0.48488253008160
H	-1.34963786566985	2.39836357748641	0.22949332927473
C	0.40947201753851	5.23785728758057	-0.41270196819201
H	0.60781708833899	4.82422406184325	0.58171817032715
H	0.15833996616382	6.29579226254226	-0.30588263668762
H	1.33292794261543	5.15850211314955	-0.99585502053752
C	-1.34717560759204	4.89222022326557	-3.00719714520122
H	-1.22571193466017	5.97868184157883	-3.05132956238522
H	-2.36733975753258	4.65265927193029	-3.31842654180771
H	-0.66698787402625	4.45044132179869	-3.74090127925868
H	-3.12646325602733	7.26794174242472	2.71649085293377
Si	-5.72067861185185	4.59473708180190	-2.46605289097398
C	-6.18790825146251	2.86094402492475	-1.99019667391298
H	-7.05573821216639	2.52767303181912	-2.57072742466698
H	-6.44196118770629	2.79125492275355	-0.92807551445458
H	-5.36061653127778	2.17429964957518	-2.19873238095486
C	-5.24281714626824	4.71109043478803	-4.26313196135654
H	-4.88017450308390	5.70731684978024	-4.53148065335773
H	-6.11990066249700	4.49241904365757	-4.88482457526238
H	-4.47171211212620	3.97645687709381	-4.51962626042822
C	-7.00073605038165	5.85491143634268	-1.95552335641197
H	-6.72718740537796	6.86403899051904	-2.27997543187982
H	-7.11015529502055	5.86069368243129	-0.86454684131757
H	-7.97668023171478	5.61470862275310	-2.39254597251475
O	-2.98893945450832	1.85700741474967	-3.24499399311890
S	-2.09306311868650	1.67315271741038	-4.39783006367683
O	-2.30022380170279	2.43960551817664	-5.62390396403542
C	-2.28573500569465	-0.14744184892091	-4.90717280835337
F	-1.49375863192795	-0.41938667229383	-5.94692523538275
F	-3.56021288793349	-0.35884894769723	-5.25651068463734
F	-1.96962426920587	-0.95115257215363	-3.88971916891832
N	-0.49247797469262	1.75943729250047	-4.01823916469070
S	0.02744961873778	1.57406796766515	-2.52819504370792
C	1.83598075953760	1.13762238230788	-2.90675326590208
F	2.47039221751409	0.97013270229502	-1.74412278023936

F	2.42790653357481	2.10569379608508	-3.60157372381946
F	1.86580010911393	0.00065931920631	-3.59792806486301
O	0.27500711766581	2.92504432188667	-1.82508063479866
O	-0.52799281164617	0.54011688471989	-1.65714205469250

TS-3B

43 conformers were generated by GOAT, all of which were used for constrained geometry optimizations followed by transition state optimizations and frequency calculations.

Electronic energy (a. u.) @ CPCM(MeCy)-ωB97M-V/def2-QZVP: –3627.89315551822
Thermochemical corrections (a. u.) @ r2SCAN-3c: 0.490728269012
Imaginary frequencies: one (–149.56 cm^{-1})
Gibbs Free Energy (a. u.): –3627.40242724921

73

C	-3.91240688350707	3.97756180263815	1.00661001329588
C	-2.64893847552714	4.00819439742134	0.62140906553584
C	-1.58767685875420	4.88003332341976	0.67198780304677
O	-0.75659853832439	4.80061601068117	1.71056546172052
O	-1.18872211491770	5.67573052496156	-0.29562495877073
C	0.52831417063984	5.46138376917182	1.62258591974902
H	1.09937396226823	5.07554773893410	0.77414732679759
H	1.02909569965766	5.21616796127484	2.55882959019016
H	0.39944484665897	6.54281064658076	1.52927637150108
H	-4.50309383949844	3.07715340969115	0.83705920755729
C	-4.68468825828015	5.08504814581642	1.70411568580994
C	-3.87409953334809	6.31514864918511	2.07156435225758
H	-4.50706493908219	7.05239882343261	2.57154238717184
H	-3.46014599360171	6.79773251663807	1.18085673929144
H	-3.05303162735081	6.04285456165366	2.74061379638353
C	-5.89364594414555	5.43961090975363	0.84307083989727
O	-6.69141962573460	4.38490883668538	0.63575479654475
O	-6.10091148986615	6.54425724178406	0.38502499777665
Si	-8.04041715884814	4.43651564864539	-0.43222360468917
C	-7.41169776191484	4.83418386086548	-2.14485174454052
H	-8.22928603960893	4.79257008563254	-2.87376736510506
H	-6.65064474413642	4.11060058443513	-2.45835784880323
H	-6.97244867887535	5.83564635945612	-2.17887298809864
C	-9.28094077826878	5.68152112867508	0.19281582884010
H	-9.53455706042101	5.48893572250928	1.24114349543436
H	-10.20842354716971	5.62209138311985	-0.38859209156491
H	-8.88907457835918	6.69965412956670	0.11587655152616
C	-8.68956696673688	2.69252790002063	-0.32256329539227

H	-9.00116397528925	2.44957321098414	0.69893628546781
H	-7.92621042388829	1.96624343544953	-0.62201627306726
H	-9.55701101439690	2.55788720372290	-0.97839803483541
Si	-1.79321164890231	1.78789983803601	0.00807713456720
C	-3.02180940468008	1.94509194149380	-1.41267038228819
H	-4.06089010730105	1.99104989343080	-1.08410463942688
H	-2.92287953801783	1.09963711319401	-2.09862583154053
H	-2.79184026131355	2.86400160623997	-1.96029190419953
C	-1.96850221018549	1.50426053388405	1.86155973632549
H	-3.01245172847546	1.38359907900105	2.15907149998289
H	-1.55388663080643	2.36125824369780	2.39907559845352
H	-1.41697446006880	0.60587730613576	2.15683119940561
C	-0.09414464337551	2.41433714742765	-0.49674251993105
H	-0.17766573836996	3.42918373105854	-0.89572295369689
H	0.37552475731715	1.80645394160925	-1.26870988944670
H	0.58309747308422	2.42305304924549	0.36186709873366
H	-5.10284852802944	4.62634452730014	2.61494007259121
Si	-1.82162685445502	6.16746226840957	-1.82524027457692
C	-1.34277026812488	7.96775801725321	-1.86270007209846
H	-1.85035386735142	8.52732204810761	-1.06944622356740
H	-1.62260554684753	8.42063236319748	-2.82044915866820
H	-0.26272726717214	8.09561293611933	-1.73414389310957
C	-3.66132088277132	5.94341130893905	-1.95712896420491
H	-4.20955275844311	6.51676682379358	-1.20284757224593
H	-3.96634539884969	4.89694478270843	-1.87557527965805
H	-3.97647542646283	6.31067195309624	-2.94254755964392
C	-0.88136258366624	5.18329005647630	-3.09935586660691
H	-1.10696206318025	5.57040792042447	-4.10024631129761
H	-1.14410206566839	4.12098431954463	-3.09020080301322
H	0.20024957039985	5.26621955682111	-2.94744134599783
O	0.94170303380703	-0.23680432876423	0.15945277833702
S	-0.07752284246206	-1.10367336762719	-0.42983173751401
O	-0.14854623198682	-2.52734602742958	-0.12031335463419
C	0.24584925445416	-1.04495544859851	-2.31622601536176
F	-0.42646063643267	-2.02001806994100	-2.92976587723822
F	1.55430079943359	-1.21449255424102	-2.52493188469506
F	-0.12924320825187	0.13097262136667	-2.84719456309957
N	-1.56205463946541	-0.32211919304653	-0.33542380876165
S	-2.91122177115967	-1.21258859314437	0.14537582321220
C	-3.42461705224829	-2.23355974502124	-1.38789215645857
F	-4.67761034458510	-2.65247467322259	-1.18348805289362
F	-2.63456361153449	-3.28617910763785	-1.56704733795833
F	-3.39760542880982	-1.46232703263060	-2.48468090566340
O	-2.65190124406476	-2.18019825730848	1.20314647994069

O	-3.98578982699575	-0.22199844723876	0.27511138508248

TS-3C

71 conformers were generated by GOAT, all of which were used for constrained geometry optimizations followed by transition state optimizations and frequency calculations.

Electronic energy (a. u.) @ CPCM(MeCy)-ωB97M-V/def2-QZVP: –3627.88599985569
Thermochemical corrections (a. u.) @ r2SCAN-3c: 0.490644204912
Imaginary frequencies: one (–103.32 cm^{-1})
Gibbs Free Energy (a. u.): –3627.39535565078

73

C	-1.49433185339433	2.05202307602005	-0.44727374179704
C	-1.82568954134650	2.92197879569050	-1.39755121082290
C	-3.09864945286139	3.45499499232051	-1.66403188481158
O	-3.42421298614600	4.72239545587253	-1.52440148030304
O	-3.97805225559910	2.74926725773842	-2.32233601045904
C	-2.54464560608470	5.56072913964651	-0.72619694458712
H	-1.65491304125817	5.81328749656420	-1.30725246578851
H	-2.26903680085548	5.04802390771168	0.19605364284043
H	-3.12688102530206	6.45742586801025	-0.51382123350635
H	-0.47241960450560	1.67461189565489	-0.42445665548234
C	-2.37479145181811	1.41201112489397	0.62493949697237
C	-1.55228390017142	0.52172633600764	1.55338270949184
H	-0.83146459364451	1.11404921922553	2.12186540087712
H	-0.99936387008336	-0.21540495677514	0.96233202867694
H	-2.19399508535539	-0.01189928656292	2.25846143334125
C	-3.24700265925380	2.42473413052474	1.34262941904813
O	-3.14995944505827	2.40826249645371	2.66470139237170
O	-4.00425399053830	3.17060481171990	0.73830171572529
Si	-3.98846486517218	3.51312076048136	3.68364633174312
C	-5.79905954397235	3.05044934370250	3.64527145771253
H	-6.22311567408796	3.22745165418866	2.65163333993762
H	-6.36654588233704	3.64838542149502	4.36776083736960
H	-5.94328813661329	1.99472709628980	3.89957806045808
C	-3.70169535720780	5.27686275455626	3.14969345172359
H	-2.63493590585211	5.46537842947018	2.99264232692245
H	-4.05829011911127	5.96173758840335	3.92844155320450
H	-4.23627345218830	5.50007339761190	2.22211126571688
C	-3.20785227528499	3.16335276428035	5.34008866393939
H	-3.65741453643784	3.78028156541920	6.12602578648564
H	-2.13507640577891	3.38324532383378	5.30844038276157
H	-3.32935852902830	2.11255917315073	5.62370742207357

Si	-0.38888015318032	3.04739108802996	-3.22234258759719
C	1.17519932611520	3.13464990207225	-4.30997035606919
H	1.96099359441917	2.44760399860675	-3.97643205759692
H	1.60046555322302	4.14416592401726	-4.34040018901074
H	0.90478094380899	2.85965984712088	-5.33781696844077
C	-0.87253893908025	1.23907685780186	-3.47432344148811
H	-1.94091181399704	1.05911627705220	-3.33149863534096
H	-0.33405301470885	0.60953575612157	-2.75639989738346
H	-0.57142811523376	0.92401628455993	-4.48015722773187
C	-1.43508193396258	4.37965405005809	-4.05563732398364
H	-2.51098730659827	4.19960122274577	-4.00348266424906
H	-1.14453220769610	4.43357977129285	-5.11043892893916
H	-1.22995332877405	5.36391498964734	-3.62178425521402
H	-3.10170554634876	0.78645505044425	0.07953159854371
Si	-5.58290016137427	3.21274307346086	-2.78463290295883
C	-6.23078070930778	1.60569227913035	-3.46830754426164
H	-7.25383147044788	1.72691031655731	-3.84137215918969
H	-6.24390596141187	0.82737745553276	-2.69806549565381
H	-5.61023217881404	1.25117081363255	-4.29817163755727
C	-5.47005460606415	4.52735283009163	-4.10355484159163
H	-4.99972492943035	5.44073663245519	-3.72759371227781
H	-6.47607488808525	4.78553893924671	-4.45538934133078
H	-4.89773839661773	4.17709819436351	-4.96927931525021
C	-6.52271579295950	3.76362162699263	-1.27543273657421
H	-7.58123890925883	3.89841383786434	-1.52815052327277
H	-6.13828141248006	4.70688516357095	-0.87817629183541
H	-6.44937712341953	3.01577360541209	-0.47990701868116
O	-0.72865859776776	4.05717852178420	2.07832217478770
S	0.61650184400424	4.00244533723327	1.49279146698241
O	1.25149956445759	2.70853049292395	1.22312897375159
C	1.72666216964704	4.87234187506611	2.76405136416235
F	1.75717577410258	4.12514950146460	3.87333258406623
F	1.24431883892479	6.08058712626510	3.06260764054023
F	2.96814494990024	4.99766504577411	2.28473256561974
N	0.71043288715267	5.12091582693182	0.28376023910960
S	1.19355358264141	4.80394216748268	-1.18903748210605
C	3.09902809524367	4.66081266524596	-1.19539676663269
F	3.49781197592581	3.62022287662262	-0.46963415550993
F	3.59854483123619	5.78645505813553	-0.68176147063520
F	3.52408219159936	4.51927509961317	-2.45161015677200
O	0.79303604345161	3.42725573312630	-1.71681995091881
O	0.88202317954833	5.92922987552156	-2.06970705468797

TS-4

53 conformers were generated by GOAT, all of which were used for constrained geometry optimizations followed by transition state optimizations and frequency calculations.

Electronic energy (a. u.) @ CPCM(MeCy)-ωB97M-V/def2-QZVP: –3627.9022457288
Thermochemical corrections (a. u.) @ r2SCAN-3c: 0.488391025121
Imaginary frequencies: one (–144.51 cm^{-1})
Gibbs Free Energy (a. u.): –3627.41385470368

73

C	-1.95586275772396	5.99770488256739	-0.39528174634743
C	-1.34400035818659	6.56732040731107	-1.41992486129643
C	-0.08009568802283	6.95798100541575	-1.73960939765263
O	0.41666817766934	8.18206656954892	-1.49265068928649
O	0.71957782845187	6.22350067689153	-2.48900679366928
C	-0.37947422656745	9.05864099075691	-0.65958153001378
H	-1.25691775479661	9.39710744590867	-1.21828474169318
H	0.27140385991141	9.90184900029418	-0.42733904182973
H	-0.70542849394076	8.54854952516352	0.24936841837931
H	-1.37065028917473	5.71004565467957	0.48467230241568
C	-3.42386170759311	5.70040274501289	-0.27846897532372
C	-3.67309654850378	4.32048244140608	0.35155795975912
H	-4.74323631482421	4.10600522253076	0.38700413532460
H	-3.27059735216445	4.27849165820615	1.36993034683054
H	-3.18035054391777	3.54679771445958	-0.24490640949744
C	-4.11037471197539	6.79408898919494	0.53113962771118
O	-5.43085354824493	6.61431144380051	0.59320213973173
O	-3.52699770866760	7.72871698808798	1.04800672913711
Si	-6.50123720822579	7.75919165216889	1.30733848044207
C	-6.06015083791045	7.95990773568416	3.11312718955742
H	-6.81584261679961	8.56896192592125	3.62320274028705
H	-5.08694687481763	8.44424580892328	3.23128615467887
H	-6.02480933140568	6.98748329781602	3.61713528773121
C	-8.16146015929462	6.93910386643749	1.10482652627980
H	-8.95625009459406	7.56483488782623	1.52666132751288
H	-8.18788685008406	5.97149822749868	1.61778707951870
H	-8.38121733484852	6.77616566860993	0.04512359130418
C	-6.37541084960520	9.35477951258926	0.35042842177074
H	-7.09315016789379	10.08921254612488	0.73427060616103
H	-6.60505866271596	9.18125067734345	-0.70636205768736
H	-5.37160987588832	9.78327967463749	0.43038816758815
Si	-2.74623414984132	7.07459860134284	-3.60730278415067
C	-3.80778978794908	8.13915179301047	-2.48449310592468
H	-3.28742649386807	8.44857018609418	-1.57752064320728

H	-4.72510558703640	7.63124146207922	-2.17509947442062
H	-4.10915685269155	9.02805346703553	-3.05104544820168
C	-2.72813161492280	5.20476034123571	-3.73910776859376
H	-1.91106345598527	4.75709896194986	-3.17195758971452
H	-2.61420507107439	4.94503571595367	-4.79780787218586
H	-3.67525663653595	4.77058382767791	-3.40353011389825
C	-1.30606636275985	7.97296591988248	-4.40237196523828
H	-0.80871947851478	8.66427999910013	-3.71584270181732
H	-1.65891659036307	8.54845958837435	-5.26340549051694
H	-0.56105790521052	7.25459718627944	-4.75813964251048
H	-3.90237142004814	5.70816662318473	-1.26370658249480
Si	2.35695647560868	6.52385167688248	-2.91957719370831
C	3.35081994828872	6.67267680456552	-1.34665960805173
H	3.10682583088700	7.58753975176272	-0.79825313225895
H	4.42259453291151	6.69336865041675	-1.57576189257056
H	3.16910647901083	5.81763929656541	-0.68638507081869
C	2.49688149280852	8.04645575153348	-3.98913119911921
H	2.19318125463880	8.94883437293189	-3.45110489430255
H	1.88379467687855	7.96387715120388	-4.89236184002884
H	3.53868364097192	8.17482277687141	-4.30723625396651
C	2.77647533366611	4.97862896786510	-3.87065748966707
H	3.81306140362635	5.00820925850663	-4.22420045334783
H	2.12671992983877	4.86815276675529	-4.74546949938387
H	2.65708198478732	4.08610941734790	-3.24767179417079
O	-6.03096522170274	5.33637155999856	-2.83321528412584
S	-6.96555649285438	6.36899199641916	-3.30237776251621
O	-7.57407989871251	7.30752266619356	-2.35963551284643
C	-8.42999894395659	5.43710198864839	-4.07560351621577
F	-9.03420806862036	4.72603867861164	-3.11714130540292
F	-8.00544787068821	4.61434890594989	-5.03568234097909
F	-9.30103682229394	6.30754581426892	-4.59255450243388
N	-6.39874037080496	7.25598581622869	-4.56969993436433
S	-5.23028261791191	6.72562114060033	-5.50554183613589
C	-5.57583166533943	7.79999491688371	-7.03178392777229
F	-5.57476231101643	9.09318321540811	-6.71842142142280
F	-6.75994858922862	7.45573745269895	-7.53276590380574
F	-4.62243732225708	7.55957967154867	-7.93520085280848
O	-3.86128280544708	7.32844475864123	-5.12692366672343
O	-5.17063356240075	5.32658224425901	-5.92469175997769

TS-4B

35 conformers were generated by GOAT, all of which were used for constrained geometry optimizations followed by transition state optimizations and frequency calculations.

Electronic energy (a. u.) @ CPCM(MeCy)-ωB97M-V/def2-QZVP: –3627.89542933385
Thermochemical corrections (a. u.) @ r2SCAN-3c: 0.4901395807
Imaginary frequencies: one (–173.31 cm^{-1})
Gibbs Free Energy (a. u.): –3627.40528975315

73

C	-4.42030459467811	5.31164656426788	-1.83976839380851
C	-3.27676490024212	5.57207854572354	-2.45879010881327
C	-2.37832974544945	4.76847152913635	-3.12025261420849
O	-1.31488020352004	4.20253823263494	-2.54090495779227
O	-2.38724323032329	4.63972569851672	-4.42436540993116
C	-1.19620504794472	4.32950712624255	-1.10010809295310
H	-0.38086364076451	3.66162944629521	-0.82256098896082
H	-2.12754089636411	4.04636791142641	-0.60504607027503
H	-0.94578448129665	5.36464101349893	-0.85119895805222
H	-4.79478211836462	4.28390461774754	-1.80566026644191
C	-5.28902966908526	6.30702668776400	-1.12452782854621
C	-6.78194204642980	6.04230194163214	-1.38668667419537
H	-7.06926991543521	5.04835304245915	-1.02599888584196
H	-6.98354450056555	6.08881014106857	-2.46105998603185
H	-7.39857913618934	6.79065630785097	-0.88352138058288
C	-5.01017884625650	6.25103767023865	0.37074927323953
O	-5.57790375177730	7.27104316949925	1.02287626896512
O	-4.35002250723490	5.38612697165052	0.91422758048923
Si	-5.30342381756486	7.57582911621670	2.69268802051007
C	-5.98084479347827	6.15695099678718	3.69906626367045
H	-7.02867267479822	5.96170995320209	3.44529015244110
H	-5.93658423726955	6.39002039408097	4.76934411332155
H	-5.40775864878579	5.24269848147351	3.52025646643488
C	-6.27145708288547	9.14563860613914	2.96488825454470
H	-5.91134922039439	9.94428263659771	2.30730712333120
H	-6.17423980191851	9.49101037130702	4.00022933083558
H	-7.33675718313829	8.99523141339545	2.75951775913363
C	-3.47449916588593	7.83996818880015	2.95682358516077
H	-2.90852611501899	6.93039153959092	2.73521827567178
H	-3.27718245643393	8.12180879692263	3.99775541993673
H	-3.09865312095617	8.64635995625043	2.31751321233101
Si	-2.50683448242228	7.91440790143445	-2.71309096895960
C	-2.52424888615255	7.99341076831802	-0.82253883099928
H	-1.62014050233593	8.53814883587411	-0.52475843753093

H	-2.50434675020310	7.00745658397920	-0.35548079229871
H	-3.38195596672592	8.54184658568433	-0.42772744556228
C	-3.79920643940406	7.85541491028901	-4.08486646121550
H	-4.82396076889961	7.79109901789465	-3.71708213228001
H	-3.59924726365322	6.97280452661349	-4.69982985071820
H	-3.71153274701009	8.74632435577467	-4.71437053393922
C	-0.79058156993374	7.50614406190508	-3.35489813826069
H	-0.53959338218080	6.45571521325878	-3.18488636270357
H	-0.02078160446794	8.10414926229078	-2.86551650649190
H	-0.72113383205892	7.70666671792758	-4.42737920391293
H	-5.06313674481693	7.32893212832465	-1.44730123612652
Si	-1.31035950790513	3.75461503986977	-5.44559972104337
C	-2.06031806275675	4.07140943500845	-7.11879959316760
H	-1.49087683516385	3.56019240651721	-7.90287087666863
H	-2.06347932449532	5.14185474957497	-7.35077152043316
H	-3.09391511257523	3.71272635490891	-7.16397404128222
C	-1.40829344646629	1.95738695681803	-4.95465630748749
H	-0.87040915406431	1.33835808915053	-5.68209601060842
H	-2.44756501970282	1.61198346088986	-4.93038142939482
H	-0.96441519641188	1.78112995289558	-3.97032117161885
C	0.41703875269390	4.44177006618819	-5.31558641088265
H	0.43789376808112	5.51714897543558	-5.51961981659731
H	1.05980932081263	3.95127510611630	-6.05672392312303
H	0.85335672077358	4.27221181034571	-4.32698122704158
O	-0.29584543069531	10.29079617389311	-4.19110366529525
S	-1.12073082794755	11.00955312547286	-3.22392725009645
O	-1.57247453163465	12.37901990348958	-3.46603145554692
C	-0.04078149590289	11.12336564608161	-1.65426127310668
F	0.93987003308459	11.99940167937499	-1.88412445074561
F	0.50473664897995	9.92538531735246	-1.37736143116242
F	-0.75530056022119	11.51987720144885	-0.60437653460338
N	-2.40280351979382	10.03148909519039	-2.77683771713024
S	-3.80320210516571	10.83387100706748	-2.25801972014814
C	-4.49549884680989	11.75063436597021	-3.82415732871851
F	-3.91053847400949	11.31224459493193	-4.94292894166855
F	-4.33173799946659	13.05972888979394	-3.70769180832383
F	-5.80354576697587	11.47786479167642	-3.88853428601297
O	-4.77933035125491	9.76589832310006	-2.00752648959658
O	-3.56103518147529	11.86671953921226	-1.25741920060385

TS-4C

32 conformers were generated by GOAT, all of which were used for constrained geometry optimizations followed by transition state optimizations and frequency calculations.

Electronic energy (a. u.) @ CPCM(MeCy)-ωB97M-V/def2-QZVP: –3627.88182329382
Thermochemical corrections (a. u.) @ r2SCAN-3c: 0.489169744389
Imaginary frequencies: one (–76.93 cm^{-1})
Gibbs Free Energy (a. u.): –3627.39265354943

73

C	-4.19266689063277	5.52802475028836	-1.87269589957212
C	-3.57519149925867	6.51992287417597	-2.52531561926461
C	-2.47527263288950	6.15451194230494	-3.35733320925730
O	-1.24431342342204	6.05059873144668	-2.92597339714586
O	-2.63558583016893	5.92112197003207	-4.61763318691480
C	-0.97986644788627	6.26562342615646	-1.50159691528306
H	-1.50468230072463	5.51164722395533	-0.91177630292491
H	-1.30150914021444	7.27060497489394	-1.22545843730903
H	0.10200781560439	6.17939352332210	-1.41446543643236
H	-3.85770462685561	4.49228851106191	-1.98986987456555
C	-5.33514510034422	5.66956347960635	-0.90793885194719
C	-6.46085435794252	4.66738867315828	-1.22612298782376
H	-7.28879597576407	4.77955553416437	-0.52137176937869
H	-6.08750715189184	3.63907384309732	-1.16785323400302
H	-6.83630020700158	4.83720545378600	-2.23953660496860
C	-4.83467999886075	5.42444656845010	0.50843532990706
O	-5.71776376630977	5.82272216248932	1.43196016442039
O	-3.76439519753527	4.91467985348644	0.77803938434818
Si	-5.36324483367419	5.75327807792850	3.11560534522736
C	-6.89911900521105	6.50511418853467	3.86291368307512
H	-7.79158050251080	5.92790726944211	3.59821020524969
H	-7.04572364263611	7.53274262278223	3.51355674805592
H	-6.82783639690415	6.52930923447417	4.95607576672844
C	-3.85267105260693	6.79427305368817	3.45384585324103
H	-3.70496536174739	6.91619762569026	4.53319075293483
H	-3.96163231560362	7.79372968189524	3.01773870756960
H	-2.95373000788573	6.33448016351407	3.03357660597799
C	-5.15630753481679	3.97132992930755	3.63012265712680
H	-5.07331834459505	3.89551155750586	4.72063160621265
H	-4.25719391464624	3.53498203189082	3.18582346392809
H	-6.01954545719616	3.37117183748755	3.32151612644131
Si	-4.19522958140600	8.59021051229193	-2.36278406730531
C	-4.45222362335910	10.48137240220044	-2.52256704428122
H	-3.63657187857149	11.07492681535453	-2.10195489063039

H	-5.36203503017855	10.71629450886788	-1.95356988500274
H	-4.62123887800447	10.82570476188297	-3.54865419237499
C	-5.81669828109665	8.10837406279997	-3.24220908807135
H	-5.62713437585212	8.25454921420686	-4.31519269154982
H	-6.62063147277677	8.80140834352274	-2.97034552590561
H	-6.15107368312675	7.07811351177511	-3.11674173541415
C	-3.89276163469609	8.58503628616906	-0.48455409011027
H	-3.62765328389264	7.62776699280525	-0.03249029087212
H	-4.76972658404951	8.99770571012011	0.03049297948318
H	-3.06872102320515	9.28607571438710	-0.29956279544294
H	-5.75157681073731	6.68100242650298	-0.92765029597266
Si	-1.56485745354810	5.07386300026771	-5.71360444012209
C	-1.32184993651341	3.39282055085350	-4.93918659774514
H	-0.72557745790610	3.45285212954991	-4.02323449708449
H	-0.79226744904098	2.73260649705359	-5.63569127333820
H	-2.28180300408801	2.92224009628347	-4.70015595393516
C	0.03061689096167	5.97591106548938	-5.98687468155626
H	0.61203740925417	6.08862850193278	-5.06771208872231
H	-0.12657842347734	6.97515100481603	-6.40157035535887
H	0.63077141230089	5.40262241720816	-6.70537102897184
C	-2.62634276763429	4.99991021944186	-7.24049682008126
H	-2.11480572781563	4.44424207555743	-8.03439404065885
H	-2.83983836273084	6.00412515767752	-7.62094983881255
H	-3.58032115411112	4.50203963336390	-7.03868869565961
O	0.31898713595424	9.20650177465748	-1.88357271870832
S	0.87300115314536	8.90383876722382	-3.21057685829471
O	1.66502841085378	7.69266452622003	-3.43075579997892
C	2.04528768435099	10.33930348977050	-3.62832164681230
F	1.38869577464985	11.49911148938217	-3.58148778895969
F	2.55889909277094	10.17050570565535	-4.85034116745397
F	3.03817639761179	10.35557385492297	-2.73253789963767
N	-0.23564556953846	9.02145320169114	-4.42862293257748
S	-1.63158769857953	9.72113553522560	-4.12377768888200
C	-2.30471845954335	9.71759848357662	-5.90074198595158
F	-3.54922246769536	10.20236143311517	-5.87104496808684
F	-2.33498790381828	8.47844164446369	-6.40177567477983
F	-1.54387596926874	10.49111431996674	-6.66905434673020
O	-2.55682454908688	8.69680258036618	-3.43469616926473
O	-1.68973575813175	11.09439880223928	-3.63527909309740

TS-5

77 conformers were generated by GOAT, all of which were used for constrained geometry optimizations followed by transition state optimizations and frequency calculations.

Electronic energy (a. u.) @ CPCM(MeCy)-ωB97M-V/def2-QZVP: –3627.9041650929
Thermochemical corrections (a. u.) @ r2SCAN-3c: 0.488850519029
Imaginary frequencies: one (–78.21 cm^{-1})
Gibbs Free Energy (a. u.): –3627.39453435320

73

C	-3.66907863730459	1.80859649842051	0.04820863048249
C	-3.24140370581224	2.13746823238553	-1.15378765810369
C	-3.10244474104501	3.20922486964655	-1.98657862199472
O	-4.09503228347630	3.68179471394287	-2.75739621426280
O	-1.93258317830267	3.72416714340930	-2.28858714427898
C	-5.43830806048667	3.34143008966066	-2.37028605740584
H	-5.65893863622065	3.77631643233539	-1.39095444078146
H	-5.56293032877034	2.25400280849675	-2.33964551531505
H	-6.08132445885124	3.77771899447116	-3.13518255326813
H	-3.69162662253920	0.75792814376089	0.34722825900562
C	-4.16937819512774	2.74253782582168	1.15878544813347
C	-4.12794440625557	2.04275596799915	2.51563802768854
H	-3.10970122014743	1.73111913527019	2.76268396592288
H	-4.76732031456688	1.15408300627844	2.49447562595355
H	-4.48337347801680	2.70199225809175	3.31232168619459
C	-3.40019823218514	4.04511524604234	1.10423913090480
O	-2.43597364301424	4.13279348727871	2.02529481803855
O	-3.62124384464932	4.90574511372366	0.27161005498449
Si	-1.35771845885991	5.46464545635374	2.15213785006620
C	-2.33698133809499	7.02401515026159	2.45588205063557
H	-2.95033940665129	7.27873577837364	1.58681683532603
H	-1.66253283064378	7.86370193952897	2.66062052202978
H	-2.99801693208259	6.91175585928450	3.32241305441963
C	-0.33175470553788	5.54319188800864	0.59891142844230
H	-0.94968442117168	5.84811135116051	-0.24945844204454
H	0.10427717249133	4.56553719509597	0.36468672491183
H	0.48777574897035	6.26304393461155	0.70660402195374
C	-0.33390144706567	4.99463990689073	3.63910858025351
H	0.19578291218996	4.05093981046838	3.47051859960096
H	-0.96022770752608	4.87659725736021	4.52988668210806
H	0.41480616298499	5.76503852013328	3.85494112900024
Si	-1.94692547009980	0.13395804043202	-2.51853251139311
C	-0.52678126202353	0.90940732399432	-1.57568268253408
H	0.37456271364737	0.29416945355295	-1.66193849535662

H	-0.79301341885228	0.99446025196634	-0.51865529496362
H	-0.29706904291601	1.91293554027589	-1.93979544692007
C	-3.04126335683109	-1.06587321847674	-1.58554891430810
H	-4.02248706948773	-0.63057741056997	-1.37964926940490
H	-2.58791474889472	-1.36890554594679	-0.63600846202189
H	-3.18894563762411	-1.96666983102052	-2.18993285648697
C	-2.76212956358467	0.87421001969231	-4.03070211528827
H	-3.83848861338969	0.97005251424503	-3.85675507547056
H	-2.60909813998900	0.23800363495672	-4.90746493247583
H	-2.36096608522143	1.86092581189543	-4.26719644625350
H	-5.20963168445898	3.00401498540685	0.91483070045749
Si	-1.58341720438273	5.01575279547308	-3.38271981737361
C	-2.54992091866235	6.51498305310261	-2.82182133005629
H	-2.07863255759533	7.43325232143810	-3.19105116619930
H	-2.60300097297035	6.57119448570061	-1.72965967457650
H	-3.57654383204392	6.48393507583386	-3.19949566787062
C	-2.00936039575486	4.53712732906360	-5.13462974160617
H	-1.39692973628977	3.69768653320045	-5.47843882708485
H	-1.80092063094246	5.39048045768063	-5.79245735909789
H	-3.06611771746348	4.27811644065840	-5.24806322720474
C	0.25638424301117	5.20305544493142	-3.17902040545167
H	0.76360592563193	4.26010863606536	-3.41135399004324
H	0.53459683153201	5.50188628332726	-2.16414172225421
H	0.63499694720655	5.96345059455639	-3.87195724095041
O	-0.97321404154429	-1.23540820630061	-3.34183135690726
S	0.06314543293150	-1.22543061365509	-4.49083803419663
O	-0.54572315419267	-1.13838135142459	-5.81671524695274
C	0.74619865998988	-2.97955735440497	-4.24288418126324
F	1.24056388128276	-3.13055218447150	-3.01777167316223
F	1.69790367628280	-3.19633462817174	-5.14718977735663
F	-0.25415620411798	-3.84239084779323	-4.43170227470717
N	1.38029510574555	-0.41340251269863	-4.13540047721278
S	1.49971653596184	1.19340908158017	-4.49426068802248
C	2.41656553590575	1.16156310176695	-6.15832324666333
F	3.60941676133036	0.58069971076543	-6.01111032375499
F	2.58797461246311	2.42384652790859	-6.56944755782941
F	1.70953095400375	0.49419006133682	-7.07161763089898
O	0.19806263605049	1.82332230326653	-4.76757292604285
O	2.43896022254269	1.79710182305777	-3.55378907218056

TS-5B

58 conformers were generated by GOAT, all of which were used for constrained geometry optimizations followed by transition state optimizations and frequency calculations.

Electronic energy (a. u.) @ CPCM(MeCy)-ωB97M-V/def2-QZVP: –3627.90028767951
Thermochemical corrections (a. u.) @ r2SCAN-3c: 0.490436681711
Imaginary frequencies: one (–164.76 cm^{-1})
Gibbs Free Energy (a. u.): –3627.40985099780

73

C	-1.90935244668125	2.64871601497806	0.03100316384856
C	-1.68202489797059	3.08304092212361	-1.19860390048939
C	-1.70387835866052	4.31783895412236	-1.82620265868308
O	-2.78492813247818	4.84664037227238	-2.39440539639084
O	-0.59923001846009	4.95255423757285	-2.10208759025991
C	-4.07191163067356	4.24772823983792	-2.09349128932373
H	-4.77204607556077	4.72508304100151	-2.77878273093324
H	-4.34624787003626	4.44643721937411	-1.05382484351247
H	-4.03202306183078	3.16913669940057	-2.26760278330021
H	-1.85392152487792	1.58076566009698	0.24255419007269
C	-2.26064379391278	3.49698560603621	1.24786636034939
C	-1.98976196633014	4.98461018228611	1.10016649807571
H	-2.22024546314884	5.50781476560856	2.03241918377083
H	-2.61308981040083	5.43257181569634	0.32160180083545
H	-0.93749002473859	5.15135605288697	0.85218686285497
C	-3.72940135691097	3.20858431093608	1.53192709104933
O	-3.91909468506321	1.97822909005678	2.02212088621323
O	-4.63164056733585	3.98377791996688	1.28236893197914
Si	-5.49002559517288	1.29125924064703	2.19404822834360
C	-5.09749751790286	-0.43210242469458	2.78748286106050
H	-4.54657628227959	-0.40956956016470	3.73383789484372
H	-4.48864478803083	-0.97329255581173	2.05527229819001
H	-6.01644287576590	-1.00660522687458	2.94898972428099
C	-6.30343006554318	1.27135597317021	0.51305689953902
H	-6.54666968698082	2.28443513757587	0.17972334332018
H	-7.23123221976510	0.68838682506681	0.54158415645669
H	-5.64604572024114	0.80700924579901	-0.23113166836732
C	-6.45479658823055	2.26108937388451	3.46116253508204
H	-5.89432627557166	2.34666761812819	4.39863040475437
H	-7.40311743916325	1.75959564027011	3.68671685274669
H	-6.67533651751975	3.26944746402265	3.09943253411046
Si	-0.59854982659107	1.58878418074212	-2.73955491522509
C	-1.60241518143585	2.36607719463278	-4.13100791426066
H	-1.35274082683214	3.40830128770743	-4.32829011588033

H	-2.66091869610676	2.30504101427795	-3.85555844228127
H	-1.46898197634168	1.80819495777458	-5.06051193860096
C	0.93214481600968	2.13478820543420	-1.77339009770377
H	1.08446766309215	3.21349925287115	-1.82707164300884
H	1.84216964008937	1.64092624191517	-2.12292342623211
H	0.77345578124909	1.86716328321398	-0.72392171738232
C	-1.53322279613646	0.11534684013187	-2.05498531629178
H	-1.79375296069690	-0.57845113318008	-2.85640771842977
H	-2.46256953810372	0.47059039848423	-1.59877770557596
H	-0.96837160527567	-0.44276259809175	-1.30324986882919
H	-1.68797628423256	3.08654173260747	2.09105055318589
Si	-0.28352125898980	6.35082517340239	-3.07463651157250
C	1.56517112929180	6.46578054653858	-2.92948821356049
H	1.88199872878621	6.57069743691152	-1.88652945142394
H	1.94799956826561	7.32736746875788	-3.48763608448158
H	2.02989712229120	5.56308187886800	-3.34231627397221
C	-1.17626024794500	7.77992311273564	-2.27251559840372
H	-0.89538551616117	8.72234475615804	-2.75674307180829
H	-0.91560649661504	7.86206863554101	-1.21152601319080
H	-2.26253459788948	7.67264546196490	-2.35181196628044
C	-0.81897482286200	6.05149046502168	-4.83230460098486
H	-0.25769175818519	5.22097487292235	-5.27425998655108
H	-0.60590330966838	6.94868699977079	-5.42666276713490
H	-1.88962181674661	5.84251595151458	-4.91643638339194
O	1.22820405625928	2.96406392020969	-4.63900818504345
S	1.68184839813274	1.61677712986927	-5.01537600235084
O	3.09757737779978	1.27109035899145	-4.94888337917356
C	1.17650389005730	1.44284417659426	-6.87961803700395
F	1.05132621067984	2.68213142109318	-7.37425503430043
F	0.00477169652662	0.81239904458801	-7.00672214246065
F	2.11394259188745	0.79950687155983	-7.55924266053044
N	0.65959173515341	0.56885371675276	-4.17811511381347
S	0.91528578530602	-1.06528393197088	-4.40736036545033
C	2.05644309428571	-1.55202350776136	-2.96273098415139
F	3.24578223011419	-0.96525524697513	-3.06793000931152
F	2.21285659458784	-2.87883279741463	-2.97247322359266
F	1.49306529630955	-1.18819144249137	-1.79742872886546
O	-0.33072472904652	-1.77729541957619	-4.13287407345201
O	1.68477408157870	-1.31424577732758	-5.62569470952591

TS-5C

43 conformers were generated by GOAT, all of which were used for constrained geometry optimizations followed by transition state optimizations and frequency calculations.

Electronic energy (a. u.) @ CPCM(MeCy)-ωB97M-V/def2-QZVP: –3627.88399297085
Thermochemical corrections (a. u.) @ r2SCAN-3c: 0.489458617646
Imaginary frequencies: one (–136.90 cm^{-1})
Gibbs Free Energy (a. u.): –3627.39453435320

73

C	-3.14391245179762	2.70438819680309	-0.03178070580332
C	-2.74688033589186	2.93089794359125	-1.27965740484653
C	-2.83770444582652	4.05657403443787	-2.09416658022411
O	-3.88518555859900	4.29854717960733	-2.88831188638266
O	-1.83186696532151	4.84413953204593	-2.31188498947789
C	-4.99228936617078	3.36917291697611	-2.87344620290620
H	-4.63373809940347	2.36250325997755	-3.10505427207137
H	-5.67123736243224	3.72660246113903	-3.64801995800509
H	-5.48631808520804	3.36685864086092	-1.90111957841867
H	-2.99552027527596	1.72033414059912	0.41161060187320
C	-3.73242550383699	3.71748508850828	0.91753287351586
C	-5.27217430938527	3.61759714821046	0.99533884442049
H	-5.58014788861328	2.58814305729309	1.20317345276361
H	-5.72912001448712	3.94203722583595	0.05674551109853
H	-5.65501832463828	4.26467310961734	1.79124286810712
C	-3.23022818026719	3.45360064516024	2.33297094505606
O	-3.08811424443408	4.57858226130421	3.03966270636670
O	-3.07016019449001	2.33998485129743	2.78701917647998
Si	-2.59194433222294	4.55309632340641	4.68587992721536
C	-0.94958321221977	3.69155526666600	4.86531913642321
H	-0.58579188874653	3.78760479049894	5.89544789349828
H	-0.20219760426369	4.12993845515948	4.19724228954764
H	-1.03686421195586	2.62891449140406	4.62302384605291
C	-3.94009267729763	3.73515789789289	5.69006650433445
H	-4.90990770195679	4.21313156208087	5.51221272206323
H	-3.72302732889592	3.81187990651027	6.76185674344550
H	-4.02555705162858	2.67501818079880	5.43314386725800
C	-2.46298029110327	6.37537155430891	5.07142502554779
H	-3.42591547598485	6.87915434567729	4.93456527368231
H	-1.73269451727521	6.85962849616010	4.41432603632319
H	-2.14336858382637	6.53478769677997	6.10738057527653
Si	-1.40709831831087	1.37369388493565	-2.27982754423419
C	0.06020087079607	0.18437298179290	-2.51414204295848
H	0.37816401160680	-0.27737422109052	-1.57263620697955

H	0.92997753526615	0.68188469070448	-2.95576516493967
H	-0.24085111338216	-0.62426856862997	-3.19192578319650
C	-2.70083130595922	0.09539797800780	-1.73543045506221
H	-3.73419729647396	0.44676064701260	-1.73914813477645
H	-2.47292123930455	-0.27118237378104	-0.72774874157817
H	-2.59994226471631	-0.76389513596089	-2.40909044821903
C	-1.57276725891499	2.09294218881415	-4.01202944805524
H	-1.40245717355260	1.28096663177769	-4.72802109897111
H	-0.77704208568444	2.82863973903223	-4.17542540264417
H	-2.52962459424762	2.56529523980747	-4.24929938718550
H	-3.44515901115679	4.73332532779692	0.62554937759033
Si	-1.77017561514945	6.30978801234337	-3.25458779558981
C	-0.03418972401322	6.91517891002278	-3.02438548737149
H	0.15468266099025	7.18852210584208	-1.98257654224997
H	0.12804100922145	7.79885233128619	-3.65329889236526
H	0.69654182824638	6.15147536433611	-3.30923618727636
C	-3.03887329609864	7.45781680740962	-2.51624324034545
H	-2.95434920248919	8.45289612718434	-2.96821004582633
H	-2.86284563655382	7.56232509771121	-1.43997712700758
H	-4.06159950112516	7.10235807880383	-2.67347361942167
C	-2.10714874810633	5.87655693967365	-5.03955088144079
H	-1.94060707546235	6.76120222533857	-5.66594705488302
H	-3.13331152795443	5.53525025889831	-5.20047007230174
H	-1.42403400480898	5.09469120027645	-5.38860394734489
O	-1.10335008371742	6.12597310319409	0.35102346849554
S	-0.12466124712075	5.07990937233202	0.66825977171793
O	-0.52661406077498	3.90921992619715	1.45562649145716
C	1.20721492613680	5.96437027377502	1.69045714624405
F	2.21388140355316	5.13012724606731	1.96722912512871
F	0.65216098064370	6.37319634404537	2.84075105616090
F	1.68018801078299	7.02627566789501	1.03440499827319
N	0.79600746932198	4.76306009567833	-0.65999314256987
S	0.94094840581832	3.34070340773862	-1.33028768016197
C	2.33245725489068	2.44078159951481	-0.38047984162027
F	1.95347288166702	2.20602012167073	0.87400204429243
F	3.41323077094118	3.22436496532903	-0.38338683099705
F	2.62507942217723	1.28589090821313	-0.98178590475486
O	-0.25650931979690	2.42261394248464	-1.09282295148616
O	1.41044777510468	3.45548987270012	-2.70977164177322

TS-6

39 conformers were generated by GOAT, all of which were used for constrained geometry optimizations followed by transition state optimizations and frequency calculations.

Electronic energy (a. u.) @ CPCM(MeCy)-ωB97M-V/def2-QZVP: –3627.90175529979
Thermochemical corrections (a. u.) @ r2SCAN-3c: 0.489870091979
Imaginary frequencies: one (–143.17 cm^{-1})
Gibbs Free Energy (a. u.): –3627.41188520781

73

C	-2.88022960410165	4.83661329947176	-3.03404573141950
C	-1.73197385590671	5.44445160565737	-2.75616761021572
C	-0.48019950566917	4.95017456986754	-2.44489656878156
O	0.52651111234291	4.86887927043080	-3.31246833138087
O	-0.04498071910577	4.68869913190308	-1.23833720849657
C	0.22941897203009	5.12718485004534	-4.69960174899506
H	1.16111583424231	4.93325061466037	-5.23080979592457
H	-0.07685914054851	6.16850214323107	-4.83175487159828
H	-0.56167735628082	4.46222622580613	-5.05635608513253
H	-2.92719680360325	3.74390838679626	-3.07627092590477
C	-4.20369394834717	5.50839330419450	-3.27852159593246
C	-4.94742678760174	4.87398489132237	-4.46713702400066
H	-5.90493461993592	5.37349827934949	-4.63585178192737
H	-5.13457480168273	3.81073264799900	-4.28171961631023
H	-4.34376073994984	4.96294085496161	-5.37555415730534
C	-5.06997918194965	5.40310373143304	-2.03035326121796
O	-6.07720194547196	6.27914800786824	-2.04149230337236
O	-4.87285947842424	4.60924434368682	-1.12920621058709
Si	-7.11854508214789	6.51308702295161	-0.68684085914830
C	-8.10226858966581	4.95287658700325	-0.40178349072632
H	-8.61252352134598	4.63745191886275	-1.31865664653224
H	-8.86894966726878	5.11695724293662	0.36441447308936
H	-7.45353849898086	4.13661794422629	-0.07102200138062
C	-8.19534205370703	7.91969537279628	-1.26702352673140
H	-8.92403184564367	8.19408075393462	-0.49607082910660
H	-8.75011591548509	7.64958213682926	-2.17194090317579
H	-7.59365039484114	8.80718526169841	-1.49101767955957
C	-6.08261687083558	7.00025143648177	0.78647815505196
H	-6.72793201151382	7.27658199598238	1.62858218965172
H	-5.45163830906273	7.86567410783948	0.55522180035055
H	-5.43623226258527	6.17800831377561	1.10738832309440
Si	-1.64860092656972	8.02143421322792	-2.66184396311266
C	0.14065127234315	7.91783901552167	-2.12225500913142
H	0.51556057031645	8.91961976624984	-1.90066352847418

H	0.78185015774331	7.46549194135786	-2.88499371720081
H	0.23797239618147	7.32122882254498	-1.21035732942668
C	-2.99502893523131	7.91922785404321	-1.36508230679569
H	-3.91329807557233	8.39599742996924	-1.72789515711333
H	-2.65624377399878	8.46501186125532	-0.47841691735906
H	-3.22859146366238	6.89993473044296	-1.06448171054174
C	-2.08997015100040	8.00985327903140	-4.49331417453542
H	-2.98835971050290	8.62145998884439	-4.63820763875697
H	-2.26266247271196	7.02062145890899	-4.91833314716141
H	-1.28166281726740	8.50047048521592	-5.04950974954972
H	-4.06830794186526	6.57456192676626	-3.48054582027197
Si	-0.85443743230327	4.07516199856617	0.15908667143904
C	-1.90916403846038	5.38948208083143	0.94863558048886
H	-1.44628557670937	6.38124037199815	0.90821279029650
H	-2.05702197539888	5.13872369946990	2.00617440868721
H	-2.89536044753968	5.43778362640218	0.47851736760158
C	0.60555179330040	3.62500855883688	1.22440648396946
H	1.24742382026683	2.88940853982798	0.72844136042001
H	0.27140588153693	3.19719141483654	2.17615736134642
H	1.21377930211658	4.50792371761285	1.44905896010441
C	-1.85620856098951	2.59155683938880	-0.36041412118832
H	-2.11857076172404	1.99787319222703	0.52344433897685
H	-1.29538699019818	1.94048745639178	-1.04005737124380
H	-2.79115714691145	2.89916383809349	-0.84003887753052
O	-0.93806746319894	10.17868434671170	0.19842979931839
S	-1.75562137058757	11.39968432684706	0.18408735107907
O	-1.12573568001979	12.70902464076492	0.34335045859546
C	-2.96702594950112	11.21995835280821	1.63639067662781
F	-2.24581651561008	11.19966573323133	2.76311252804600
F	-3.65262120187048	10.07275202582650	1.54016294648088
F	-3.82150472415854	12.24140171383930	1.67997808231570
N	-2.93725975995627	11.41256182302663	-0.98616788607828
S	-2.68216667963226	10.99070294224336	-2.47856901595264
C	-1.82877353374930	12.43875711086612	-3.36873279268287
F	-2.62472509465151	13.50512840274686	-3.31874887820145
F	-1.60939294052557	12.10731067752515	-4.64581028559720
F	-0.66741275011158	12.71520577166533	-2.77614592594328
O	-1.57742535029085	9.94453688356208	-2.68068579001797
O	-3.93491938792425	10.75007088311385	-3.19217023782262

TS-6B

36 conformers were generated by GOAT, all of which were used for constrained geometry optimizations followed by transition state optimizations and frequency calculations.

Electronic energy (a. u.) @ CPCM(MeCy)-ωB97M-V/def2-QZVP: –3627.89569284167
Thermochemical corrections (a. u.) @ r2SCAN-3c: 0.491073846773
Imaginary frequencies: one (–170.08 cm^{-1})
Gibbs Free Energy (a. u.): –3627.40461899490

73

C	-2.47027157846683	4.66099667471844	-1.88319284189661
C	-2.16654211924865	5.82033010220908	-2.45723732771445
C	-0.97566361138892	6.23387805849292	-3.03236959161091
O	-0.87326170714456	6.21289019696227	-4.35471834725514
O	0.01674618799587	6.81227945883909	-2.39944972190149
C	0.26066330039709	6.86649244248385	-4.97547046919322
H	0.08907163109738	6.75917127723588	-6.04590082243933
H	1.19192500980652	6.37464681591341	-4.68291367979072
H	0.29342623885593	7.92280675694526	-4.69737289656291
H	-1.73055607901323	3.85487863992129	-1.84971005119619
C	-3.78923938999988	4.30611130953305	-1.25234545120089
C	-4.16980916137857	2.84184314045712	-1.53544535368487
H	-5.14017258770625	2.60171578587940	-1.09376234878053
H	-3.41774283349222	2.16117653512398	-1.12177805452935
H	-4.22568997646957	2.67504259536647	-2.61541335726561
C	-3.73916083874649	4.53032933099024	0.25074975822367
O	-4.96013100800710	4.57645062154227	0.79128321151603
O	-2.71198039321624	4.64561247920072	0.89366237775554
Si	-5.22910199233313	4.98410596243302	2.44271753742092
C	-4.48031066238665	3.66623005079217	3.53149353628003
H	-4.84748661513351	2.67187391809390	3.25429672598235
H	-4.74829866407755	3.83793180724247	4.58052776459518
H	-3.38956397436750	3.66510278595370	3.45000536209320
C	-7.09087791919068	4.98980147092672	2.54016927223794
H	-7.42851155221231	5.23571229947452	3.55314609782698
H	-7.50403136987566	4.00990350646313	2.27781904688322
H	-7.51700894315667	5.73075236454081	1.85519208913308
C	-4.51435584688176	6.67955633602278	2.75379722235006
H	-4.79575807696105	7.03669809984770	3.75122735202147
H	-4.89362512985676	7.40202819068347	2.02219869657449
H	-3.42220340500985	6.66529299233455	2.69046886079121
Si	-3.86009364388005	7.57567892964021	-2.98637438320702
C	-4.39356194933261	7.54534469099523	-1.18614299415049
H	-5.34480280590385	7.04755258741314	-0.98885951421137

H	-4.48816511117550	8.57720276732696	-0.83711303982406
H	-3.61240384688726	7.05897167597972	-0.59865846203196
C	-4.34411395798365	6.26965370560723	-4.26177618387965
H	-3.50733754919400	6.10602961625725	-4.94646380976294
H	-5.21611949251677	6.56004539050358	-4.85317804937785
H	-4.56200578614515	5.31584956090002	-3.77552404946990
C	-2.47822572127679	8.83368703812707	-3.19241195601072
H	-1.79714737487376	8.76519412306069	-2.33860777988433
H	-2.88991147373883	9.84636264467334	-3.21119999232919
H	-1.91501811627124	8.69202120826474	-4.11604426940751
H	-4.58513146620559	4.95135788928967	-1.63562529113160
Si	0.46269298142786	6.74820445920561	-0.72171644117093
C	0.71123763952781	4.96308288894470	-0.25521997948136
H	1.33594978241272	4.90271405229119	0.64400588911849
H	1.21757878964456	4.40044948453117	-1.04714928989912
H	-0.24759660475312	4.48660941320019	-0.02727821723460
C	-0.81600083343929	7.57974300350384	0.34141027477009
H	-1.69858163903797	6.94536740740132	0.45890471304728
H	-1.12007314837882	8.55900790695470	-0.04097238158925
H	-0.38744215424001	7.73394358117606	1.33984033184325
C	2.05952849723589	7.70792207852273	-0.77593992099585
H	2.50165518759114	7.77257061965408	0.22445570676786
H	1.89493081882303	8.72983729804566	-1.13440720310149
H	2.79204789523821	7.23005117184977	-1.43520253504791
O	-3.90317400600369	8.92433812813477	-5.69151496347928
S	-5.23189808092045	9.38424466336436	-5.27733336956219
O	-6.40268885797886	9.09974696043058	-6.09923786164707
C	-5.08183466096412	11.31571753174254	-5.19241821883817
F	-4.04436992698250	11.66741218063379	-5.96175915274153
F	-4.84566078405582	11.72527874180351	-3.94177753745551
F	-6.18118191936383	11.89023226654412	-5.65778098607007
N	-5.37831579777896	8.90071921617982	-3.65518012479608
S	-6.84951004670732	9.24917191698627	-2.93045692187548
C	-7.87476031887780	7.66208493919677	-3.18737262886416
F	-7.23166214314925	6.61285518435277	-2.64105290712115
F	-8.08136203434282	7.41374417180607	-4.47675940575664
F	-9.04984097545920	7.81573546643439	-2.57023846617691
O	-6.67170942130175	9.32048578971061	-1.48272992786148
O	-7.55008687224281	10.30400563507487	-3.66074332334991

TS-6C

58 conformers were generated by GOAT, all of which were used for constrained geometry optimizations followed by transition state optimizations and frequency calculations.

Electronic energy (a. u.) @ CPCM(MeCy)-ωB97M-V/def2-QZVP: –3627.88294361776
Thermochemical corrections (a. u.) @ r2SCAN-3c: 0.490208294821
Imaginary frequencies: one (–61.16 cm^{-1})
Gibbs Free Energy (a. u.): –3627.39273532294

73

C	-3.97654943291585	5.31421388584184	-1.92914896794426
C	-3.43979265923414	6.37848359421921	-2.53725849693857
C	-2.31850227412345	6.17320379838849	-3.39681818876575
O	-2.55693802853995	6.07017748058258	-4.68134224251397
O	-1.08011688261663	6.14262390398842	-3.03481597735310
C	-1.42727236306218	5.91799577465782	-5.59265158508559
H	-1.88219020794589	5.85519714982139	-6.57989355870577
H	-0.88502639680260	4.99961037127810	-5.35572200157122
H	-0.76422854435623	6.78119315502615	-5.52067148340897
H	-3.56168441071547	4.31451049685596	-2.08852087169550
C	-5.16713080014903	5.31743107157335	-1.00911527446886
C	-6.15531058453021	4.19721231979031	-1.38759882821736
H	-7.02354386693992	4.20527439202298	-0.72310337682475
H	-5.66914797619623	3.21796750074566	-1.31912819775070
H	-6.50007095036569	4.33627157719643	-2.41661623114936
C	-4.71960110621862	5.10566987994515	0.42838655657567
O	-5.64617693660042	5.49229568371489	1.31286682934087
O	-3.64967985158109	4.62272110400215	0.74850913985826
Si	-5.33621383378301	5.47567022022975	3.00720914961547
C	-3.84753166532964	6.54693454583659	3.35286329085617
H	-3.75566561799257	6.74095244606496	4.42783613175785
H	-3.93814836883989	7.51481819092024	2.84647029015643
H	-2.92663981526594	6.06692404242147	3.00956751731150
C	-5.11802307524003	3.71234174041038	3.57720168239210
H	-4.20808228620092	3.27451117465581	3.15690170506311
H	-5.97017166417397	3.09349747912621	3.27490544996718
H	-5.04825800986540	3.66858284808071	4.67039764281459
C	-6.89967337701982	6.22928239960771	3.69229794529503
H	-6.85998694913006	6.27965895567641	4.78616989343796
H	-7.77885443466812	5.63747904179158	3.41578680962311
H	-7.04530999917837	7.24700135381927	3.31461160206481
Si	-4.35016231021159	8.40471905312510	-2.41202346577292
C	-4.84916740294901	10.25697049578109	-2.46699160328697
H	-5.08198762025819	10.64796856183540	-3.46156003050072

H	-4.10592372485035	10.92107586917576	-2.01577602660000
H	-5.76799783124886	10.33865413429631	-1.87071932504782
C	-5.82791843021599	7.72528598677937	-3.39429888897732
H	-5.56807619742245	7.81662387639792	-4.45752113326708
H	-6.71407377844741	8.34798892551362	-3.22924272522666
H	-6.07234126470902	6.67652023422667	-3.22124686176437
C	-4.17112928351331	8.36292471968635	-0.51817381885048
H	-3.43961769867399	9.13401595403439	-0.24719535133660
H	-3.84896001477299	7.42427199869726	-0.07108749967245
H	-5.12533924922228	8.66768473678801	-0.06936597013851
H	-5.69374649145940	6.27458719698970	-1.04439518906327
Si	-0.29220377044794	5.80223210002567	-1.50099671615960
C	1.50077163394585	5.99642580662755	-1.94261631807439
H	1.76672070547863	7.04447232155661	-2.09937895508760
H	1.75314420463467	5.44573667588790	-2.85463377939859
H	2.11733204313320	5.60072297591754	-1.12664948536868
C	-0.70854935571037	4.02747537981910	-1.10836645909906
H	-0.00886608545746	3.65762568971588	-0.34885796541558
H	-0.60611043050438	3.38160683953380	-1.98763908582846
H	-1.71875759988802	3.93285495947401	-0.69889606717630
C	-0.86278981225439	6.98601216809042	-0.19178682059821
H	-0.11500565807848	7.00835209193325	0.61049683982763
H	-1.81563282435332	6.67001734242080	0.23945389578956
H	-0.95359765484526	8.00139445742435	-0.58794334162576
O	0.24544639994615	9.21484052262264	-2.55381915827725
S	0.54118045915420	9.18289200567004	-3.99348302711042
O	1.38463691601936	8.13291956276847	-4.56598706524989
C	1.45893395278411	10.80511785304172	-4.36488101530885
F	1.74260208865082	10.88290284598736	-5.66725110877655
F	2.59812097605178	10.81607264611070	-3.66345880517082
F	0.71254430188420	11.85209511683326	-4.01097515984160
N	-0.79304394731945	9.31184268058331	-4.95012075572465
S	-2.16993129534955	9.83401464731072	-4.34724282853485
C	-3.14665893340069	9.73406385080361	-5.98225253213828
F	-3.09103393985726	8.50107869990923	-6.48899438614929
F	-2.63346011825985	10.60153290806928	-6.84886827012107
F	-4.42173239598619	10.04819806720955	-5.74162127284901
O	-2.79168482689178	8.71027251432235	-3.48680538398328
O	-2.28889136519210	11.19041796652858	-3.82501341984439

TS-1-IDPi

38 conformers were generated by GOAT, all of which were used for constrained geometry optimizations followed by transition state optimizations and frequency calculations.

Electronic energy (a. u.) @ CPCM(MeCy)-ωB97M-V/def2-QZVP: –8437.60868109445
Thermochemical corrections (a. u.) @ r2SCAN-3c: 2.121328864117
Imaginary frequencies: one (–47.61 cm^{-1})
Gibbs Free Energy (a. u.): –8435.48735223033

273

C	-3.31283494990119	4.49340400006553	-5.69275648483427
C	-3.06226761364114	3.95032115453359	-4.64725493650395
C	-2.76955059972712	3.34520670762728	-3.40118737718658
O	-3.61147173905761	2.40243427923943	-3.05574236477455
O	-1.81368137637390	3.66388204228168	-2.66404835775881
C	-3.27614194738025	1.64065237562496	-1.85675977077730
H	-4.01191223529909	0.83827955719885	-1.82899339556023
H	-3.36370690460684	2.28369716814764	-0.97737300019928
H	-2.25917996685451	1.25299609671376	-1.94801674468799
C	-5.78383767076083	6.30908576370501	-5.22651946349006
C	-6.60999097404309	5.06218741296684	-5.28034608676002
H	-6.44794647973624	4.51264140572490	-6.21579361894167
H	-7.68363490936993	5.28667324389422	-5.22634962318183
H	-6.36027859063296	4.39276320174906	-4.45384877997604
C	-5.02217574176963	6.66125008288831	-4.17803316487480
O	-4.87662977956662	5.86983537825192	-3.09147871810227
O	-4.28930152040586	7.79277749494202	-4.07141786038333
Si	-5.41775280171635	6.26213361677652	-1.53722295972967
C	-7.28334815052031	6.37163055524387	-1.61086661289879
H	-7.59278236901146	7.13037414003095	-2.33917303419934
H	-7.70731529916813	6.64936187235720	-0.63930238787267
H	-7.72529719754664	5.41670073585156	-1.91499230486685
C	-4.68463745829954	7.87506881522534	-0.94354510865880
H	-5.09669780680579	8.72276850739600	-1.50012419393117
H	-3.59801892721468	7.88865128421605	-1.07447870307078
H	-4.90615556902638	8.02638907148227	0.11972789722106
C	-4.83146812160558	4.81289005702576	-0.51696536669233
H	-3.73887619258204	4.72886022609767	-0.54929506452519
H	-5.26218730756823	3.87985978980661	-0.89684892699537
H	-5.12786190279318	4.91912306479656	0.53229254635858
Si	-3.75558393928445	8.89822527233374	-5.22526199311783
C	-5.19045290456367	9.93518333716459	-5.83573257288271
H	-5.72565717967241	10.38706927103527	-4.99321561292798
H	-5.91336164475548	9.35961552489351	-6.42201716128255

H	-4.82236218393236	10.74977226911548	-6.47053326243470
C	-2.90726617845730	8.00090631083003	-6.63183671809500
H	-2.37959657021629	8.72507041037523	-7.26382632998783
H	-3.60710145711564	7.45551735579806	-7.27321412849408
H	-2.15862015452349	7.29628205214247	-6.25150762852716
C	-2.54040343850629	9.94316382781407	-4.27339254827683
H	-3.01196508682029	10.38813534820087	-3.39044658377517
H	-2.14983307120654	10.75736666015973	-4.89378260740747
H	-1.69142583383066	9.34055100752582	-3.93351311567061
H	-5.83127403603017	6.99481920551489	-6.06737482324672
Si	-0.27522629376546	4.85022125428541	-2.89418912444960
C	-1.31745808488730	6.36501964748879	-2.55199656476042
H	-0.72914764268856	7.28267490178864	-2.60372520232484
H	-1.73738417687106	6.28329194612983	-1.54249839650995
H	-2.15798582202803	6.44176440064133	-3.24928462480625
C	0.58011770185794	4.05034772810653	-1.45236143502572
H	0.37981157114105	2.97594266049275	-1.41871885562107
H	0.20938594879697	4.49916635363245	-0.52429090187272
H	1.65921457629209	4.19886181646867	-1.47951594523985
C	0.14327490818551	4.39513944311414	-4.65208501800698
H	-0.44895269823108	3.55184903143042	-5.01519114048411
H	1.19506252352397	4.11358419985289	-4.74144968560572
H	-0.02408787725850	5.25524030452588	-5.30767464419912
H	-3.50074550209545	4.95295773734872	-6.63350990465703
C	2.70016384781498	1.22057103169308	-11.14391399050406
C	1.19753569775152	1.42793562545239	-11.39508246824027
H	1.07684781264869	2.17905466070624	-12.18742282934065
C	0.44383963391915	1.85121712356283	-10.14666158224284
H	-0.61276320093360	2.03439376373239	-10.38482726500604
H	0.85068453757103	2.79901421564612	-9.77017234401344
C	0.52476848041097	0.80701930175330	-9.02389503030617
C	-0.12442411437994	1.39613049327927	-7.76438177156888
H	-1.09398086795805	1.83789160155299	-8.02474605425459
H	0.49710597094915	2.17133669758829	-7.30771193691923
H	-0.31827940371957	0.64364495534860	-6.99860257534448
C	-0.26205027937092	-0.46360922357472	-9.41186995986818
H	-1.31259089569395	-0.21434037234799	-9.60710753274064
H	0.15296238618522	-0.94936966547415	-10.29997410505021
H	-0.22855040427444	-1.18916157718243	-8.59237966714966
C	1.98049560386841	0.39913200282394	-8.79932392761991
C	2.34305528745640	-0.16407363160891	-7.56936828533625
H	1.61401816720829	-0.21211131271040	-6.76931068190604
C	3.61020681264794	-0.69270342578397	-7.34261694364861
C	4.53584538340547	-0.66681081709760	-8.38622777704185

C	4.20915036245384	-0.06141963154998	-9.58766551067894
H	4.96410078459340	-0.01805670024527	-10.36765495558127
H	5.51871711755042	-1.10345072482837	-8.23531773465475
C	3.98442012302976	-1.38976384992770	-6.08584360511317
C	4.17398360267345	-0.73566972080786	-4.84172414504795
O	3.95763949962163	0.64328698664175	-4.78991366808182
P	2.78900229325604	1.25576346077638	-3.84320666160971
N	3.12817532022979	2.77932954546694	-3.61132795732429
P	4.26550817888017	3.80328689026139	-3.23815620822772
N	3.72824731034512	5.20289023321099	-2.63670926301685
S	2.84636724791240	6.19770988223183	-3.51498531292071
O	1.46738643346554	6.36662777080330	-2.95074283106562
C	3.62448737004413	7.87319322370519	-3.09555424909819
F	4.93831050240197	7.84048508736184	-3.33419107072061
F	3.06256678572747	8.80714697682740	-3.87500981853403
F	3.41838833757396	8.20245409527860	-1.81530336843021
O	2.95130861569292	6.08540678202311	-4.97476488855667
O	5.24647834886411	4.23017620732250	-4.44788804082267
C	5.92367493746803	3.20962900690067	-5.10744419368541
C	5.55509229687054	2.95869194793978	-6.44623781011130
C	4.55796649866462	3.84508875048956	-7.09686437791811
C	4.96879653556671	5.09561726632325	-7.54215410340998
C	4.08878182928922	6.01789895028045	-8.11428211950941
C	4.64129850129297	7.37976241994182	-8.53385752447783
C	3.62581687593877	8.11609594672809	-9.42183524438985
C	2.21698785104212	8.05476281689032	-8.85800458021730
H	1.53235764566727	8.66233356008031	-9.46526301470582
H	2.20647461241563	8.48820747095883	-7.84913443054995
C	1.67296293109762	6.61873253307476	-8.78694599417034
C	0.41754928904203	6.65058164625228	-7.89942534732971
H	-0.27460322749161	7.41540898044824	-8.27197494015030
H	0.68860941591692	6.89706234323608	-6.86695691698758
H	-0.11874114253328	5.69587322770004	-7.89644952367954
C	1.27476572355008	6.13104213982623	-10.19305274904138
H	0.84712589609686	5.12380172477782	-10.14577841398618
H	0.52450376685913	6.79999422460174	-10.63291704093510
H	2.13809802412151	6.08674873580894	-10.86409702169125
H	3.94771695706219	9.15968016002186	-9.53770788072483
H	3.64306151633022	7.67346998210628	-10.42708813580450
C	4.93367570129994	8.20165412418379	-7.26250993609128
H	5.33885960876664	9.18590272407727	-7.52867434158503
H	5.66436249848489	7.68687651712232	-6.63031424697937
H	4.03279962664172	8.34124373675975	-6.65856255673835
C	5.94200808545717	7.23152258083377	-9.34167836769958

H	6.24622456792177	8.21095038109394	-9.73005162587169
H	5.79724957024187	6.55449024707795	-10.19074236030268
H	6.76925722124287	6.84862760751241	-8.73744770381045
C	2.72988853364975	5.67472227175950	-8.21234110860355
C	2.33190834240594	4.40600004161296	-7.77686661302799
H	1.28486081269990	4.12847979841420	-7.84540744981830
H	6.01507949449622	5.36042552915027	-7.41731055869843
C	3.21766439803098	3.49259749369886	-7.22809869786630
H	2.87070682311545	2.51933377964740	-6.89297292881940
C	6.21829987929638	1.95886956517836	-7.11151707547468
H	5.97115598331877	1.75559113733345	-8.14953879756491
C	7.20792648407557	1.18088948906723	-6.46622380023368
C	7.57668434622241	1.47158719855960	-5.11449285134666
C	8.55431996327094	0.65941475777609	-4.49476952007558
H	8.81255470702770	0.84227712328389	-3.45795366560297
C	9.16417098172002	-0.36166477258311	-5.18245602226937
C	8.83447221278818	-0.62011069135073	-6.52727765745008
H	9.33657452860248	-1.41919628833871	-7.06477236650957
H	9.90616788689363	-0.97704877481203	-4.68150435844622
C	7.87361872622383	0.13600057540260	-7.15120122092642
H	7.61178787088128	-0.05022212756834	-8.18926508328685
C	6.95139296458038	2.57405052440887	-4.44677041312671
C	7.44790044324238	3.11153958419679	-3.15742025049834
C	6.61920888198539	3.37225413519718	-2.07463458173855
C	7.11716352174664	3.76744931369426	-0.80544505803347
C	6.27742901482218	3.98739086849505	0.39834489036466
C	5.34018215254399	5.01188735760035	0.47809329899557
H	5.14928057313972	5.60592966678590	-0.41085331634524
C	4.68166432702949	5.33111781171988	1.67614186107361
C	3.79372131861549	6.57689744104951	1.69682081305893
C	4.59723180122888	7.78712022344164	1.18505947847175
H	3.98785675784778	8.69557114551947	1.26434672055854
H	4.89626882696294	7.67842938255157	0.14081040844961
H	5.50284995117687	7.92544183955275	1.78587588509717
C	2.56942234871147	6.35822240275403	0.79159478666366
H	1.91335700077932	5.57951261769115	1.19317452796443
H	1.98826233418916	7.28397636079031	0.70359124453076
H	2.86332729963809	6.04706445010512	-0.21392359520102
C	3.33556170471830	6.90350335516243	3.12943092040464
H	2.48934965174105	7.60075054969248	3.07521755006686
H	4.13895783912329	7.43869916559695	3.65207676093233
C	2.96031815387576	5.66426124986706	3.92167712265972
H	2.54986337673319	5.94040914865843	4.90201608179433
H	2.16643095344684	5.11288734366786	3.39836889012249

C	4.16064897080843	4.72741190449201	4.13022347158551
C	5.12977259359320	5.31982926940730	5.17184429526652
H	5.97597185063891	4.64468593361940	5.33562688246370
H	5.53784844130116	6.28226643210067	4.84916352544250
H	4.61741601732002	5.46299217643573	6.13100109862127
C	3.62611080535267	3.38630654046334	4.66465828886333
H	4.42907414788076	2.71839707620193	4.99093201245183
H	2.98049468828937	3.56848670259886	5.53242337704719
H	3.04118723393636	2.86466898245385	3.89985791762072
C	4.89874708777232	4.52562489492229	2.80728709797369
C	5.84938118692340	3.50057025689734	2.71937891202230
H	6.05468813969228	2.88765179722481	3.59221568494656
C	6.54831050069528	3.24847201436553	1.55389356994294
H	7.30314797624587	2.46795752822255	1.52189431920681
C	8.47830684961090	3.92636275865293	-0.66747535087881
C	9.36054030159327	3.80541195587026	-1.75774212501427
C	10.74111740969645	4.08765111012504	-1.61630432153179
C	11.57842970173959	4.05756410027599	-2.70197734637044
C	11.05629758362051	3.76403869630222	-3.97801733574141
C	9.72608041053919	3.46188126771583	-4.14133053576336
H	9.34450794591768	3.25251491287200	-5.13367742645707
H	11.71005348081733	3.78507060668164	-4.84503744547470
H	12.63446392154665	4.28331666938128	-2.58695307792458
H	11.11880845640850	4.34920260329181	-0.63105628943484
H	8.87455278649878	4.21470718252793	0.30295786772029
C	8.84067185372449	3.43950037835998	-3.03456520086374
O	5.24794755113998	3.11666408172207	-2.15064581556048
N	1.36506217136431	1.01959343030031	-4.54414262462206
S	-0.00106320010926	0.63731242025789	-3.82563832193360
O	-0.04310095617032	0.80878298998387	-2.36035729524120
O	-1.15167019529826	1.14168050755195	-4.59897594452288
C	-0.10278852149018	-1.24277277321212	-4.06444229870429
F	-0.08973148524231	-1.56009067258700	-5.36876760089549
F	-1.24603267797277	-1.69726797763120	-3.52609874770908
F	0.92655255472793	-1.85609705922542	-3.46519291508120
O	2.80715477436243	0.32198335942851	-2.51975396149079
C	4.00005393026168	0.09065461889921	-1.84842753762123
C	4.08303819315431	0.54594183149208	-0.51276658738808
C	2.86458340843250	0.98444294544401	0.20687507676457
C	2.74159498985851	2.24251561874334	0.79241729573075
C	1.58544538262279	2.54750853453367	1.49306899029129
H	1.48813089310205	3.54450805811444	1.91062763303896
C	0.54511680105927	1.62908495867329	1.67432736877504
C	-0.70404258528105	2.07203663268706	2.43808399195173

C	-1.24441461342171	3.40023059288168	1.87959536290562
H	-0.55380954346928	4.23469800707937	2.03989628432439
H	-2.18831695575353	3.65220493216104	2.37900930621218
H	-1.43293929479037	3.31612294040549	0.80371490037529
C	-0.35637927923088	2.26976552424149	3.92585352184933
H	-1.24312785966419	2.59022457850247	4.48661002496300
H	0.02558630012157	1.34912722393666	4.37770224560485
H	0.41643159896503	3.03645918482530	4.04600345824822
C	-1.81742593681119	1.02275534733022	2.28629175436456
H	-2.62065671951732	1.24927286170344	3.00014412430534
H	-2.25711610384757	1.11490735121401	1.28276968082910
C	-1.30387351995667	-0.39224931854652	2.48459111858254
H	-0.82396776235992	-0.47200713174462	3.46928845051971
H	-2.13443248788190	-1.11035775240428	2.48568601185690
C	-0.29505720499825	-0.80081313895166	1.39836367287563
C	-1.03009077990749	-1.15150961969649	0.09324087030664
H	-0.32162036407888	-1.41495748832170	-0.69687039023699
H	-1.61213468149637	-0.30486342274800	-0.28025444236671
H	-1.70441735082274	-2.00147084665908	0.25506746953498
C	0.43947548921914	-2.05897790552334	1.89732602943117
H	1.08223274576711	-2.49705686776605	1.12836377889474
H	1.05947605054159	-1.82833768894417	2.77040848667094
H	-0.29478257438774	-2.82060404783355	2.18639093248477
C	0.70207366664985	0.33356353952147	1.15019704565164
H	3.52884753539923	2.98266884211068	0.67750966677522
C	1.83980879898696	0.05897232983199	0.38823185796868
H	1.95348307877448	-0.92307626870653	-0.06266132442673
C	5.28967843956736	0.40995565942372	0.12956522759625
H	5.37901387908174	0.73982532265591	1.16069539974863
C	6.39968143718902	-0.19336292825426	-0.50469532840447
C	6.24472842863949	-0.76943010300634	-1.80572237066014
C	7.35335894853045	-1.42779941049656	-2.38684900373916
H	7.25687417170170	-1.84335264490767	-3.38358651103120
C	8.54853272171258	-1.52484010195475	-1.71567113706011
C	8.69977446173379	-0.96426037628722	-0.43216350365963
H	9.64932302711872	-1.04993908952707	0.08793862672611
H	9.38777659342547	-2.03105473636647	-2.18427273837959
C	7.64497089057006	-0.31251965157756	0.15746245066814
H	7.74695937659340	0.11254198071772	1.15257996382563
C	4.98220660340974	-0.65122652232924	-2.46919727237624
C	4.67124886875026	-1.37960950456339	-3.72150573590142
C	4.82224421683330	-2.80368172371157	-3.76697003605696
C	4.63261117180881	-3.48031226592756	-5.00931850540839
C	4.80398464204930	-4.88445748588230	-5.07246506217839

H	4.66750655476984	-5.38131680849233	-6.02971010974382
C	5.11370193999159	-5.60605806931241	-3.94778723319358
C	5.24774977533242	-4.94756758330977	-2.70837112942378
H	5.45593632344787	-5.52461502863577	-1.81212006913428
H	5.23537752647616	-6.68375106417314	-4.00444530455548
C	5.10775566788381	-3.58386072561653	-2.61873272345544
H	5.20318088910123	-3.09682004282023	-1.65491259086410
C	4.25251275089871	-2.73940496841049	-6.14605505677067
H	4.12859873911530	-3.25357059997009	-7.09597188632162
H	0.76668736099997	0.49778959250480	-11.78817152232035
C	3.40812550345776	2.58981577457888	-11.06010519117829
H	3.02277846701869	3.19532633080432	-10.23404348181873
H	4.48288632574765	2.46014893146932	-10.89170191174349
H	3.27206857453251	3.14379085326007	-11.99733726534491
C	3.26801668564411	0.44244202745365	-12.34304111836177
H	2.96447099585112	0.93565884453431	-13.27410787188610
H	4.36125810168491	0.40295111178565	-12.33961323507891
H	2.88752526804119	-0.58472527181975	-12.35929573702697
C	2.94398226663859	0.48765304676976	-9.82282870223479

TS-1-ent-IDPi

38 conformers were generated by GOAT, which were subjected to a preliminary single point screening at the ωB97M-V/def2-TZVPP level of theory. All 15 conformers below a threshold of 5.00 kcal/mol relative to the lowest conformer were then subjected to constrained geometry optimizations, transition state optimizations, and frequency calculations.

Electronic energy (a. u.) @ CPCM(MeCy)-ωB97M-V/def2-QZVP: –8437.60512784468
Thermochemical corrections (a. u.) @ r2SCAN-3c: 2.121383630452
Imaginary frequencies: one (–62.78 cm^{-1})
Gibbs Free Energy (a. u.): –8435.48374421423

273

C	7.37910847617011	1.73685384104577	-5.99813779500816
C	7.66758957781763	0.39113350129597	-5.69373367420662
C	7.81595984818723	2.73197233791977	-5.16146085084306
C	8.41656401521828	0.06535239367978	-4.58901399503724
C	8.91643565191898	1.07043934189585	-3.72431137269444
C	8.57122223518178	2.42678776816599	-4.00288885014684
C	8.96132076435383	3.43740293145006	-3.10250608946842
C	9.60358313644828	3.15800908330400	-1.91576379261346
C	9.93418275150991	1.80278488200141	-1.65679704449190
C	9.70125662354445	0.77997413042472	-2.55906098077636
H	6.79377483331550	1.97965804996981	-6.88011582603996

H	7.28639171260722	-0.39913482668844	-6.33388546907763
H	7.57438875455781	3.77244835060245	-5.36346597791816
H	8.61408054611855	-0.97649694991612	-4.36558682223725
H	8.70527460085109	4.47006681840216	-3.32577396563372
C	10.16755490230269	-0.60740015869635	-2.33029071959517
C	10.86207427673988	-1.34105406575650	-3.34600466008525
C	9.81177594580834	-1.29210897457109	-1.18719684763044
C	10.97619027998296	-2.76096036924791	-3.22479502678646
C	10.39287950979843	-3.41392112115035	-2.11442001577441
C	9.83997207992931	-2.70303200065779	-1.07923301426881
H	10.40230060583619	-4.49865335675111	-2.06477347661183
C	11.64019532045447	-3.48911888698801	-4.24052490796527
C	12.18974768205671	-2.84887011189001	-5.32396310333277
C	12.08892869272074	-1.44760560268411	-5.43628252451542
C	11.43474232077289	-0.71292306280931	-4.47647448561498
H	12.53304611142692	-0.94279607130846	-6.28964129423954
H	11.36871123523928	0.36567276080419	-4.56861022247602
H	11.70841048334442	-4.57007582563885	-4.14376056964202
H	12.70301489324046	-3.41795730479879	-6.09348513567246
C	9.97503781590761	4.29707927396368	-1.04005038957813
C	9.48804631450159	4.45944492585580	0.25485308183031
C	10.76107714749683	5.31455919437925	-1.58456050848400
C	11.08329411526191	6.42460396714170	-0.82409140704421
C	10.60716793598817	6.59495018199360	0.48047479683465
C	9.77034003169940	5.59800581873293	1.01947050296051
H	8.84423948903289	3.69009341526528	0.66490180347274
H	11.13666209054174	5.21289445030129	-2.59885859658024
H	11.72525066175346	7.18380132034298	-1.26200512479487
C	9.06736960778482	5.77991459400181	2.36602427465284
C	11.07658735079561	7.81636968668863	1.27363206433173
C	10.19159851912188	8.05114592761123	2.50816612497262
C	9.86109744721015	6.76198943572144	3.23854072063603
H	9.26332805628809	8.54865160779596	2.19774630422740
H	10.70674356110849	8.75207997990121	3.17877223197487
H	9.28384477306028	6.97617245793010	4.14837554437979
H	10.78709319824932	6.26805503137428	3.56116431513423
C	8.92296297837656	4.46665690598921	3.14695347430962
H	8.23929791053129	3.76076856528937	2.67135042361695
H	8.52580019805853	4.68469253965010	4.14654003033545
H	9.89158002270221	3.97574965582685	3.26567595439066
C	7.65173973769097	6.32453442517458	2.08271776416975
H	7.67420152639261	7.24035292740234	1.48419495252038
H	7.13274480459662	6.53431766858205	3.02641825129190
H	7.06911498364810	5.58053393422741	1.52826643249578

C	12.52863766629944	7.55252716607954	1.72012647631444
H	13.17969384875019	7.40904220451977	0.85036463622942
H	12.60791953316706	6.64958260234973	2.33404360502965
H	12.90935287707139	8.40653820299917	2.29535006713006
C	11.04408663843387	9.09900874793912	0.42555300644752
H	10.05489901871231	9.24851896297578	-0.02066340819304
H	11.78525818596846	9.09028046682821	-0.37900799143212
H	11.26720093781325	9.96285817165619	1.06295059819840
C	7.87686288054108	-3.93582751467981	-0.24500526028808
H	7.54932808075129	-3.95326265948978	-1.28088217828254
C	7.03513058694650	-4.44074944023235	0.75205637174516
C	5.67835519122700	-5.00351105050752	0.32959496037477
C	4.79305826088418	-3.83989162462362	-0.15838970765089
H	4.66250853030662	-3.08180540226666	0.61993846558511
H	5.24558996545760	-3.33828029492997	-1.01969055074008
H	3.80582896025626	-4.21238477191024	-0.45878355930919
C	5.84124151598936	-6.02357911600789	-0.81069248554715
H	4.87545588053914	-6.49721835713126	-1.02467155038413
H	6.19262416050860	-5.56056081324281	-1.73742334498278
H	6.55488714026586	-6.80610501633232	-0.52960324512421
C	4.99912821095465	-5.72127576588246	1.50683870987741
H	5.45004825989602	-6.71526261811380	1.62996126917409
H	3.94415560130877	-5.89149522027916	1.25449391677522
C	5.11899148929510	-4.94439296804135	2.80655832679473
H	4.54478070564221	-5.43526275109346	3.60403098924999
H	4.68612407151542	-3.94176594739742	2.68294349455723
C	6.57942758532388	-4.79763256828503	3.26310924095704
C	6.60670745586773	-3.76293279310049	4.40268940409771
H	5.85505946927745	-4.02614884101041	5.15730895297806
H	7.57810070923781	-3.73292246937884	4.90759321151151
H	6.38555006858402	-2.75816729129323	4.02578229707396
C	7.11107232823661	-6.13696147486014	3.80724745766871
H	8.13933049980642	-6.02234585994856	4.16629773869366
H	6.49305449233076	-6.48228382459853	4.64566166089806
H	7.11953833943439	-6.91577213224975	3.03851675317956
C	7.44964224012738	-4.34504812217762	2.08990386570488
C	8.71656931095749	-3.81028387745392	2.35568859093352
H	9.06264402434833	-3.76209843848406	3.38232429400371
C	9.53570145190445	-3.30519181293102	1.36081388922326
H	10.50123552663155	-2.87294155222051	1.60681373928132
C	9.10558439839678	-3.34920544032087	0.03589422230766
O	10.62037630426916	1.50804280856672	-0.47473528246092
O	9.20983862248960	-0.61974708560510	-0.13963366011771
H	13.73427059850465	3.94949628233545	-5.30007585742130

H	13.96473620212566	1.47845186027535	-5.49414145060767
C	13.76556602420893	3.33760351772368	-4.40329475518242
C	13.90561827296277	1.93940141888186	-4.51212470123899
H	12.67621513109568	-9.03627700140967	0.28510377743900
H	13.55283210588030	4.99765371880650	-3.06700008366720
C	13.66240683027566	3.92068306172435	-3.16459983388512
H	14.52566718313983	-5.76262615901194	3.48659791349577
C	13.96683146335598	1.15192611845181	-3.38764019647392
H	14.06854462522541	0.07597642754150	-3.48246078511461
C	11.60841075381950	-8.83128996602593	0.15150529971603
H	11.41372136922513	-8.71024166947701	-0.91817051231866
H	11.04606901505353	-9.71139037274440	0.48544797432374
H	12.17795411388427	-8.55678958972312	2.63983393008634
H	13.64636412682339	-6.75399340638921	4.66867882335742
C	13.69415569512556	3.13681512335785	-1.98639254866758
C	13.87363852142302	1.72332986614449	-2.09780601859241
C	13.70176457996694	-6.47326203014290	3.60942105809865
H	13.54683999588527	4.30091644479233	7.84024635372995
H	13.49740188828600	4.80656628558437	-0.62186236955405
H	13.94703578926378	-7.36514454080913	3.02492660192244
H	13.25567172126417	-4.74297365210782	-2.06333906902322
H	12.00285044596502	-6.75764085977651	-1.44118887934015
C	13.58304876617183	3.72678284830419	-0.70738490921096
H	16.98083973399610	4.08209341176334	4.67702970422997
C	13.03875335903575	-4.94818107283114	-1.01848216964860
C	12.33665176680063	-6.08376672310382	-0.65710635828364
O	12.60207686669017	-1.27889065923097	0.32994131144916
C	13.81366099724581	-1.50760504838421	-0.33071745939312
C	13.43549543662560	-4.03042379254374	-0.04403447747471
C	12.04363827175787	-6.38202365380217	0.67726833985898
C	13.95579243508732	0.93938888393365	-0.90344305898744
C	11.25213229125188	-7.99492396207452	2.46051642620831
C	14.22716621775686	-2.85600530370128	-0.48405327729632
C	11.17550216651649	-7.60696010822683	0.97514679942721
H	17.30409194205887	4.32120305202526	2.96196547683086
C	14.47777948007958	-0.44598709494876	-0.92038744029538
C	13.16060198173040	-4.32001966999818	1.28970617595423
C	12.49772907041777	-5.49411748643820	1.67217177525527
H	14.04610028534469	3.11067316289015	6.61821388264726
H	13.49547426223429	-3.62229180707059	2.05074666923581
C	13.39101983117275	3.96760434784206	6.80700701883668
H	10.42669427735031	-8.68387246062052	2.68517028615919
C	13.53363115600620	2.96264932730371	0.43428413096248
H	12.35510890542934	3.62549314635036	6.72582598125068

C	17.12565202465037	4.84545124622756	3.90644316111685
H	15.77599037012760	4.80176998342091	6.36490652624241
H	11.64669549888852	2.70080446085470	2.43044326771063
C	12.37538008741851	-5.82675427415348	3.15856135951628
C	13.66090274010702	1.56159460682030	0.28906505795693
O	13.63443612052241	0.79286149053153	1.44839299166864
C	15.39267185705891	-3.09036913164291	-1.18085995942546
H	18.02452160640204	5.42392112276931	4.15279003243465
C	13.50791526256551	3.57529138285456	1.78105465266396
C	12.49132222003031	3.32609359825337	2.70158560280933
C	15.75252796642831	-0.69739096038114	-1.53021325179546
H	15.71958640494028	-4.11815533208773	-1.31701574254504
H	11.78002078903765	3.65311215007499	4.67366639635853
C	16.19567313860006	-2.04539319589337	-1.67691651838169
C	11.20067029905635	-6.78935152275069	3.38180165467976
H	11.20126754373466	-7.11241673566829	4.43180791638544
C	12.57916328448662	3.86559943591380	3.97144982093085
H	15.35965637555177	4.56767343421085	1.44338358377238
C	14.56925085381971	4.38836178330841	2.16701706905678
H	16.33299454678149	1.37215379462360	-1.80259367262247
C	13.66836731447489	4.64090055562209	4.38869156134442
C	16.62158129176413	0.33858876130991	-1.95414655707832
H	9.63931565480274	-6.97865539834224	-0.45060535340059
C	9.71927481714523	-7.24935741387299	0.60798593062363
C	14.67256114381611	4.93579762128438	3.45093707784439
C	13.70590655618719	5.12302432213218	5.83946148711208
H	9.06137007456703	-8.10813422726333	0.79350045219604
H	13.01192124158577	-3.92611498698137	4.06751162235309
C	15.10540785855212	5.65353530906675	6.19198935905323
C	17.44017534204056	-2.30980094060632	-2.29860186629262
H	17.75346223446698	-3.34443693267717	-2.41294102200557
C	15.90414331009658	5.77749989586335	3.78590430272670
C	12.14055243209907	-4.57979163423904	4.01614994996066
C	17.83326098330538	0.05139414495588	-2.53474634816095
H	15.04804339834576	6.19906915278081	7.14313842558532
C	18.24226925986412	-1.28361465919423	-2.72834513714189
H	10.26533530583097	-6.23686757727998	3.22038857027202
H	11.90753283486760	-4.87932179892995	5.04528724540952
H	18.48629218653781	0.86386905219467	-2.83969884543650
H	9.35353086333635	-6.39582943154994	1.18692304140369
C	15.68077478449095	6.54031641710008	5.10123051846988
H	16.47513334765906	6.35651840565803	1.73846941841656
H	19.19878381420007	-1.49475603493687	-3.19730952314025
H	16.63491267531434	6.97770730933702	5.42376502287549

H	11.30426372947200	-3.99128731040683	3.62908009700048
H	12.83509176937331	7.08443763720991	5.37369658182836
C	12.65264945789510	6.23016355985554	6.03409536838459
C	16.18690014625424	6.81710972658858	2.68792304793628
H	11.64874304010739	5.85358909414920	5.81108495936229
H	17.01315422531744	7.46643867861441	3.00149139823070
H	15.00094662083188	7.38258949994130	4.91246117015993
H	12.66045555534867	6.58535000713408	7.07192563464431
H	15.30682628420598	7.44330517030476	2.51001010649496
P	9.88588786276086	0.60806795182809	0.65949991270516
P	12.48678539378624	-0.33217226721099	1.63811385575598
N	11.00373940399307	0.21105796001170	1.70913325100534
N	8.66793058974464	1.42315870137192	1.30711726915345
N	12.98444740871870	-1.17630831529195	2.91604039907275
S	7.16190853936038	0.95007848809075	1.50201253562190
S	13.04530538417279	-0.66564291482185	4.41486830284802
O	6.95998209885577	-0.46085714228195	1.86872316761027
O	6.45607403658017	1.97743226889795	2.30321906656383
O	12.05762539779475	0.40830725904731	4.77190590680517
O	13.15109790882843	-1.79812819685099	5.34073175642342
C	6.31578086525742	1.15678844098784	-0.19679515743521
C	14.71796453899969	0.22692459558884	4.62234365461141
F	6.52622334482315	2.39694467971666	-0.66245649402844
F	4.99289926401442	0.97600284549139	-0.03994035574003
F	6.75655816336132	0.27703606383773	-1.10017662177377
F	15.69831420216448	-0.54874239485602	4.14913227939087
F	14.74467452103823	1.40349174586264	3.99927158354243
F	14.91877951900235	0.43102963757907	5.93480987364927
Si	10.13586713718985	-0.13178501111457	6.03632000578006
C	9.85654203164813	-1.47434768264709	4.76991556304337
H	8.78782012034704	-1.66401673515454	4.63357313079563
H	10.34785970158724	-2.40135523398382	5.08537288055525
H	10.24819810637195	-1.18888289888297	3.79154646405230
C	11.29673266138595	-0.30770094545234	7.48747120494906
H	12.18898009278144	0.30706965449779	7.35471053224191
H	10.78546745443235	0.00079500353460	8.40547523125544
H	11.62462660204351	-1.34531809306630	7.60191639717646
C	9.59966595868518	1.60044041792313	5.60701848211377
H	8.71022123768486	1.91139691741906	6.16207003383038
H	9.38284933567378	1.68023231290482	4.53617857582899
H	10.41433930984008	2.29258633245319	5.83883919021614
C	7.46696473396087	-0.94588623640448	6.80160177378438
O	8.66932183288223	-0.73873447603394	7.13266613989703
C	6.79960477591882	-0.25576883780402	5.79586056057830

C	6.24946118843243	0.42519188393974	4.95386976552330
H	5.92025326550040	0.93685571212081	4.06926609355178
O	6.76421680278797	-1.88377943166910	7.41851844671302
C	7.47297142463497	-2.69399036616619	8.39025972738728
H	7.81473083907003	-2.07291211781442	9.22179127041700
H	6.74082255531556	-3.42629141442184	8.72727011950989
H	8.32765541362969	-3.18457196933936	7.91752318779798
C	4.40907317480996	1.87651838099959	6.39341763171361
C	3.70466448405866	1.31652036110292	5.38127312618445
O	3.51731524041072	1.88096639156314	4.17477303317909
Si	3.34639217635690	3.51455266870278	3.73475779153412
C	1.89106200881764	4.18119731692152	4.70306955523997
H	1.72101114546762	5.23881118440082	4.47159424028195
H	0.97188880171065	3.63255505113801	4.47051545337831
H	2.06970979651654	4.09991203198378	5.78117243296709
C	4.89394615689702	4.50748814638990	4.06605754225297
H	5.00143422613889	4.81335230534764	5.11141921786322
H	4.85503570449698	5.42188001303139	3.46138424292411
H	5.78843855169892	3.95546825147891	3.75885910190140
C	3.02243283830069	3.40390656363138	1.90506998325022
H	2.17729488258772	2.74390540994073	1.68180854897359
H	2.79928577184099	4.39206743628403	1.48745396742697
H	3.90835637639858	3.01229636863503	1.39349781002274
O	3.19305654516819	0.08732145171568	5.47641329492988
Si	2.45500089763407	-0.81260626001286	4.23090952000048
C	2.31317800804249	-2.49862061637773	5.02169132732915
H	3.30949769911766	-2.89568549172847	5.24721064703726
H	1.81530142299896	-3.20613781827005	4.34902863034348
H	1.74293865043825	-2.45913763854613	5.95586849701615
C	0.77180945672184	-0.07360028853157	3.88152750493859
H	0.18589100226379	0.02958028730386	4.80143949556469
H	0.86452527964628	0.91628316840040	3.42300673516479
H	0.20515887842413	-0.71011271810308	3.19203089244425
C	3.50666536953316	-0.88767933554325	2.69328895688502
H	3.55414587104349	0.07674538213403	2.18019212105603
H	3.07952038578732	-1.62033303205443	1.99671520522508
H	4.53642726207284	-1.19542038703011	2.90634525999154
H	4.81870797772970	2.86599906656193	6.22486352440447
C	4.63084042189431	1.22089416857974	7.71133260635201
H	4.11489459997266	0.25990854377327	7.76663328754531
H	5.70376239453207	1.03492126533661	7.88072173635820
H	4.28531180328720	1.85161281883596	8.53939851935130

TS-2-IDPi

68 conformers were generated by GOAT, which were subjected to a preliminary single point screening at the M06-2X/def2-TZVPP level of theory. All 25 conformers below a threshold of 5.00 kcal/mol relative to the lowest conformer were then subjected to constrained geometry optimizations, transition state optimizations, and frequency calculations.

Electronic energy (a. u.) @ CPCM(MeCy)-ωB97M-V/def2-QZVP: –8437.61243555313
Thermochemical corrections (a. u.) @ r2SCAN-3c: 2.124423810961
Imaginary frequencies: one (–67.89 cm^{-1})
Gibbs Free Energy (a. u.): –8435.48801174217

273

C	9.61349938294580	1.56416480824482	-7.18999184199227
C	9.73785623320123	0.21439319804661	-6.80131482289139
C	9.72023051395075	2.55574595038691	-6.24874549942260
C	10.00016230892404	-0.12072915141123	-5.49535688531998
C	10.15055304212739	0.88039366188445	-4.50296192090423
C	9.97189006338791	2.24119002824211	-4.89104282732739
C	10.02256300693117	3.24676238101678	-3.90739147023755
C	10.18031693104983	2.96675231861252	-2.56573771162228
C	10.36097650675614	1.60833674585152	-2.20042166763244
C	10.43037797295155	0.58465147940456	-3.12961770953312
H	9.41374400523750	1.81269439899509	-8.22814357927544
H	9.61664156595858	-0.57150225673572	-7.54110669931298
H	9.59814862290613	3.59984141115981	-6.52578877165938
H	10.07880632276207	-1.16478446471091	-5.21581642689632
H	9.89254786785987	4.28319722495258	-4.20869604097130
C	10.71878141933647	-0.81675918694268	-2.74493744902678
C	11.74625888170571	-1.56722306580509	-3.39101358348278
C	9.89991944584218	-1.46854085218331	-1.84737715379491
C	11.76603082525892	-2.98502191816991	-3.22356484886348
C	10.80092469808851	-3.60506292863039	-2.40062267192279
C	9.86369666957613	-2.87842815505154	-1.69377877950605
H	10.82018453323643	-4.68745374900815	-2.29431868308828
C	12.73661849996709	-3.74195220559505	-3.92363330098602
C	13.68127168059196	-3.12664716701632	-4.70682695785033
C	13.69480970135259	-1.72108702212842	-4.82281047183084
C	12.74245591660842	-0.95940039423674	-4.19002171978668
H	14.46023788574383	-1.23703677263229	-5.42318071977091
H	12.75368874421762	0.12129438930018	-4.28672819567828
H	12.71870227429219	-4.82263347934693	-3.81989644622106
H	14.42297311880697	-3.71924987108556	-5.23475490642578
C	10.21502200464184	4.13880497941563	-1.65012284703206
C	9.22555629543624	4.41517147040084	-0.71102405908431

C	11.19126038324598	5.11100915678218	-1.88584458095722
C	11.15717338876821	6.31423012929633	-1.20699051576058
C	10.13258318375066	6.62831653115052	-0.30511366570753
C	9.15589594448330	5.64973781346393	-0.04317105041390
H	8.46405350408386	3.66466652451638	-0.52702211121855
H	11.96736079347973	4.91313536807799	-2.61884095065761
H	11.93426894537447	7.04583310965537	-1.40919922669756
C	7.96250300496260	5.91771930804142	0.87580789027373
C	10.14322779891681	8.01535379769436	0.34314483941976
C	8.79158834930787	8.31764286071249	1.00965082288373
C	8.24180809551220	7.13022837494744	1.77830722128481
H	8.07186096938915	8.61901614142256	0.23748855201286
H	8.91208388485114	9.18468492902609	1.67257052512435
H	7.31209133326392	7.40605150888047	2.29362212489098
H	8.95294387483045	6.83753762582056	2.56305964217778
C	7.65161847782373	4.71597925425223	1.78005879734596
H	8.53560567511468	4.38705733013611	2.33669269899097
H	7.27120014045067	3.85650981642484	1.22816724867869
H	6.87457900782799	5.00747173669414	2.49810431020078
C	6.72651828589760	6.19060232269874	-0.00617319873774
H	6.51405416684171	5.32672930492523	-0.64244555586929
H	6.87670036197366	7.05397766039431	-0.66131735201574
H	5.84639710382502	6.37741848196970	0.62133515093719
C	11.27508962924543	8.07012801953407	1.38699293745699
H	11.32480269387510	9.06661301994758	1.84274333794527
H	12.24375596873774	7.85874321147932	0.92192015027060
H	11.13270504261383	7.33104980205164	2.18029923735807
C	10.39374738496947	9.11379717154776	-0.70667604271339
H	9.68693655498012	9.02531921606010	-1.53853867228177
H	11.40808069437898	9.08259591397066	-1.11490877729970
H	10.25885206496410	10.09841597058997	-0.24335614285861
C	8.11801820489065	-4.62514568430241	-1.54551458473606
H	8.40299536748774	-4.89147505469411	-2.55938886761023
C	7.06330064724851	-5.30695842099742	-0.93125302968692
C	6.32110868662239	-6.38108760719269	-1.73038915154972
C	5.43536495499233	-5.67809264612202	-2.77940013723954
H	6.04842502853338	-5.08900990452226	-3.46910198824579
H	4.87396010549491	-6.41783720308364	-3.36356366369848
H	4.72412793548014	-4.99163936619407	-2.31049229435819
C	7.28687008189928	-7.32759015734277	-2.45915207125425
H	6.71744965095685	-8.12320085392417	-2.95459059103173
H	7.87419169248003	-6.82005632650031	-3.23011374993307
H	7.98040235255370	-7.79507221809448	-1.75307539181792
C	5.45289135999622	-7.23983806133940	-0.79744613660550

H	6.10317679431490	-7.93239409159431	-0.24570023238792
H	4.78559876766596	-7.86196350208411	-1.40855572246377
C	4.64788413695948	-6.40453065619909	0.18272243805376
H	3.98415344049913	-7.04652087049063	0.77737246130373
H	3.99794735675486	-5.71060836001829	-0.36700360857685
C	5.54434439203385	-5.59857800303341	1.13467724696328
C	4.66019355576239	-4.57449475188601	1.86505555274285
H	4.32958064188069	-3.78367024408980	1.18213174022363
H	3.77686400049542	-5.07943065934645	2.27491933541719
H	5.19572696867243	-4.11505956385493	2.70139810711764
C	6.17464823615177	-6.52673121694277	2.19154893548031
H	6.79817639330297	-5.94643056327083	2.87957116072214
H	5.39179482767718	-7.03020848702510	2.77349524979497
H	6.81464691323770	-7.28822080512105	1.73442607446920
C	6.66545945704439	-4.90770382466067	0.35872253382930
C	7.35362878474677	-3.85410726903691	0.96705338293801
H	7.08035695710894	-3.53779350209213	1.96923581063663
C	8.38797627979767	-3.18275635377069	0.34284571831540
H	8.88379878797036	-2.37556770331255	0.86600665229939
C	8.79063281677730	-3.56191599777570	-0.93739338850199
O	10.59585921393646	1.30596530585858	-0.85743754428744
O	8.98195067641038	-0.69350201923951	-1.13989878443323
H	15.30227298668100	3.91114778552006	-4.18551845689854
H	15.76964575487655	1.48040535119582	-4.44551398705828
C	15.03656318686807	3.22242786561774	-3.38886071317148
C	15.31142042545028	1.84605450441676	-3.53088072859753
H	12.02475225575157	-10.47781760024260	-0.40596080633877
H	14.22983717040403	4.74728192006058	-2.12146461287723
C	14.43499370792850	3.68751844838170	-2.24642540938651
H	12.19494598627266	-5.31612001046273	3.46358127673634
C	15.01285026722950	0.96570093427103	-2.51968527016147
H	15.22882702739996	-0.09118324600058	-2.63424678766261
C	12.52020281627929	-9.50691759006342	-0.28275866081448
H	12.95991972002978	-9.46710311182096	0.71801831282087
H	13.34197910417825	-9.44934670288757	-1.00409556368118
H	9.95759029500884	-9.47980016356502	0.47699795498293
H	11.39836808675249	-6.74675854132742	4.10960910607293
C	14.07537053238254	2.80109054774137	-1.20147514300457
C	14.40005151545753	1.41739390277742	-1.32650354485291
C	11.98412685412052	-6.36863744556386	3.26238983708968
H	9.20351534448769	4.23595342247574	5.81395354379726
H	13.28163402638349	4.32726889074100	0.09573829877154
H	12.93244287041169	-6.91681160681567	3.22526684950625
H	14.47763399967410	-5.10961379914103	-2.06793302702533

H	13.31876788592736	-7.26304129502289	-2.19381728388381
C	13.45819454336180	3.26140274303280	-0.02072903782613
H	11.53446624963127	1.70382362601996	6.58086260937487
C	13.78047759381843	-5.39580701802286	-1.28615787442402
C	13.12414082098721	-6.60920379564581	-1.34813880256789
O	12.45092640246417	-1.71646212914004	0.04026521669401
C	13.83108247433442	-1.93620123886021	-0.07655033478548
C	13.52198722701012	-4.50675353174825	-0.23554924416065
C	12.22652416912240	-7.01716573061134	-0.35308393896531
C	14.09447096236945	0.54236490255655	-0.24241727421318
C	10.35165786872770	-8.45453030662688	0.48396673523139
C	14.32840227444371	-3.26389836304755	-0.17831347645951
C	11.51680702406146	-8.36067584015108	-0.51372490815975
H	12.76489466482847	0.80529568689062	5.71383699565475
C	14.64637867934422	-0.82792632124252	-0.19893435072358
C	12.64348084385911	-4.91013565995143	0.76348329734855
C	12.02822059311075	-6.17059519657376	0.75086883914934
H	9.24986436294111	5.78642797425455	4.95549538971645
H	12.46554805004622	-4.24203332703385	1.59788428585757
C	9.65474551510305	5.23462468341589	5.80963898907250
H	9.53662988707956	-7.80054125116936	0.14111137185207
C	13.08638545628663	2.40913135785252	1.00000349175509
H	9.33698780085577	5.75595525351939	6.72081084259000
C	12.61563163463235	1.58979006621354	6.45762249381525
H	10.97766196243052	3.64763938511918	7.33059193312801
H	11.14161453751136	4.27549811958442	1.20479426140295
C	11.18481440898512	-6.56699481296022	1.96237668041205
C	13.36696506581259	1.03184175234290	0.82177917875342
O	12.94862538779940	0.11996784314449	1.79650502874264
C	15.69608268176445	-3.41137017571998	-0.30869398928472
H	13.04391486829644	1.25448071132411	7.41048872909162
C	12.52493542531970	2.98272347261081	2.24065934717883
C	11.57041201431662	3.99998583028670	2.16407679495521
C	16.06532555733053	-1.00058846058162	-0.24571222371886
H	16.10138918200162	-4.41760659984957	-0.37372102590585
H	10.40992692043866	5.41856534017450	3.23462262100967
C	16.58855229123396	-2.32430668012129	-0.31241286491107
C	10.77478426750393	-8.04710061537832	1.88406625436103
H	11.61320049366967	-8.67510204581707	2.21322985076528
C	11.16256388781881	4.64200465504108	3.31769091274844
H	13.77842378912005	1.86016254218057	3.55574441751124
C	13.03273068108449	2.64622652969091	3.49587177422525
H	16.60424208126197	1.09293050776659	-0.09217857647047
C	11.67809960441623	4.32817930356564	4.57950172228187

C	16.97992259669529	0.08023398112592	-0.18489901273692
H	10.32119538216426	-7.62769690085115	-2.18538451971760
C	10.93740596334514	-8.49705244454110	-1.93297396418966
C	12.64964847506639	3.31477707787554	4.66540499561825
C	11.19100331502094	5.14038390401198	5.78167987734604
H	11.71652335224212	-8.59756408959090	-2.69448412636121
H	10.19766472881187	-4.62539740623054	2.07756876144656
C	11.64212577721857	4.49086044075572	7.09964425610483
C	17.99026327315192	-2.52041208199253	-0.36752908914526
H	18.37086756943945	-3.53695069600718	-0.42647097901883
C	13.28026242752159	2.89733437629471	5.99439421721176
C	9.93141372339670	-5.68339830471003	2.00508824990826
C	18.33497165535529	-0.14220825312517	-0.22636789302623
H	11.51324190629910	5.21597873898138	7.91424673737596
C	18.84876198020908	-1.45138501006743	-0.33276050190280
H	9.96117488443662	-8.22061813559028	2.60075969201048
H	9.32001244569086	-5.93706954287094	2.87847167385472
H	19.01806659912726	0.70019260315850	-0.16869619687414
H	10.30675790869595	-9.39265772462268	-1.99105007036663
C	13.07921366002223	4.00244276794975	7.04295710329010
H	15.02039312197182	1.77326169695588	5.24970309860446
H	19.92254418060882	-1.60960247405396	-0.37039947255678
H	13.38974343700568	3.61763051128912	8.02329650034872
H	9.32288300784905	-5.80849894367066	1.10514638178931
H	11.43229068681045	7.05362063046234	4.75242916603669
C	11.76476963467248	6.56779001560481	5.67592332954982
C	14.79261938626645	2.65547531411142	5.85455901590839
H	11.42841752695186	7.17773555651739	6.52324928862116
H	15.22693119134522	2.48793149146172	6.84715186238816
H	13.74921732916201	4.84215726147141	6.81195900134153
H	12.85894506065165	6.56051411268020	5.66593832983428
H	15.28996106097273	3.52093082585310	5.40323089393612
P	9.58504999591288	0.33506660992518	-0.03935725063193
P	11.85201761469518	-1.00575206531753	1.36946185497373
N	10.42594403240511	-0.40153381319847	1.08498710625105
N	8.37213050731949	1.24037764757293	0.47822223754163
N	11.90115359599593	-2.14835070103665	2.50369877976322
S	6.89073068333656	0.77078530798139	0.84480897704027
S	11.66671361319184	-2.02413051278104	4.05632113940058
O	6.64514185354457	-0.67949069692196	0.86737442550933
O	6.37882347810190	1.58205635149193	1.96835116726660
O	11.09638832127366	-0.71466927826100	4.50864299773443
O	11.08038516968701	-3.24365030359415	4.62320922124246
C	5.89747492756597	1.38679618815756	-0.64887889755225

C	13.42155851908522	-1.98802480781518	4.77786410684840
F	4.60988575675032	1.05273654068345	-0.49460523774532
F	6.35851480157090	0.83971989732475	-1.77751204899946
F	5.98081933516497	2.72518924429732	-0.75633365189002
F	13.35053040435953	-1.89972706815327	6.11273570442502
F	14.05966528014513	-3.11451184899286	4.44793209891277
F	14.11424844901164	-0.94261187939774	4.30957484452143
Si	8.91202143413827	-0.01895470241704	4.66183823347182
C	9.10269050128823	-0.15047158919211	6.51525120728825
H	10.05303955443732	-0.60460229143904	6.80066012869448
H	8.28132111599784	-0.75203568634400	6.91766635131827
H	9.04505583467176	0.84556168690319	6.96940182906008
C	9.51418326871643	1.52640215330951	3.82579567691265
H	9.36304903462058	2.37236832012728	4.50494548940115
H	10.57322195821394	1.47970737877818	3.57276654587029
H	8.94443700606605	1.72485698604123	2.91340589417290
C	8.30109045976250	-1.51165065377763	3.73622905768896
H	9.09772411643520	-2.08780615963786	3.26604544240566
H	7.60360134787916	-1.21562073054579	2.94775178035531
H	7.76977075499666	-2.17277452505153	4.43074287720007
C	5.95902812196261	0.74785840233183	4.60239943945173
O	7.16351584968925	0.83523412901002	4.90117206160855
C	5.28441841165357	-0.42182497287298	4.18821832185858
C	4.68776625745326	-1.40358818266415	3.82610986336122
H	4.18635299539611	-2.26824450306007	3.46035775379508
O	5.16645529195026	1.79695473274347	4.70461021108974
C	5.82563994815744	3.06907806029120	4.94696974736738
H	5.02779776873796	3.80838190188555	4.89473197397086
H	6.29526625320163	3.06931307135332	5.93429486895682
H	6.57298783669137	3.23800069241399	4.16786807697608
C	4.16120343661050	-3.37919957704724	6.20562460619167
C	5.42301809441850	-2.96907530671239	6.41221910052063
O	5.77117950031188	-1.79086054890957	6.99117198184872
Si	4.93456647745206	-0.92718985870297	8.17805439266848
C	6.13796239596143	0.42194146185427	8.64520533384901
H	6.41781351203142	1.01457741355801	7.76686933838126
H	5.69151726799228	1.09835476339060	9.38268540638282
H	7.05952824892474	0.01318006271283	9.07247160856491
C	3.35877696304051	-0.15621215739496	7.51492221760704
H	2.51909818735667	-0.85735514263541	7.48377620917442
H	3.50225027582501	0.24405137241075	6.50538307628906
H	3.06242406169193	0.67762279843116	8.16285845793082
C	4.57120126796889	-2.07010899693465	9.61326182202781
H	3.92008273964291	-2.89653746411105	9.30935619034727

H	5.49312310490462	-2.49598024903695	10.02451463915306
H	4.06581978341540	-1.52504213781694	10.41888147483150
O	6.49741398701772	-3.66400546872694	5.98479416909635
Si	7.50049655683740	-4.61754204357901	6.96585145351900
C	8.51050066462117	-5.60813732288984	5.75784434557849
H	9.21159872910946	-6.26255990070359	6.28863190808220
H	9.10290096732379	-4.94256150306391	5.11985290019936
H	7.87843233463304	-6.23569012897510	5.12029528176860
C	8.60281368517408	-3.54179000616908	8.02300807322734
H	9.19486793682843	-4.16261196772385	8.70592402122306
H	9.30131010040436	-2.97715272433541	7.39714790255796
H	8.02812414922903	-2.82996101316957	8.62544352933761
C	6.38867863921422	-5.67914626811031	8.03525188239396
H	6.98404430773930	-6.30578253223746	8.70905943351298
H	5.76193640541206	-6.33939208094695	7.42645089282340
H	5.72446896068537	-5.06364124383818	8.65296754453352
H	3.36063038635179	-2.71536999924913	6.51559867695932
C	3.78943389011051	-4.68449334064351	5.57617294482476
H	3.06127725047133	-4.54301381877595	4.76727265809489
H	4.66524744773399	-5.18461035299068	5.15441326296706
H	3.32371247754370	-5.36693448456182	6.29987647062275

TS-2-ent-IDPi

18 conformers were generated by GOAT, all of which were used for constrained geometry optimizations followed by transition state optimizations and frequency calculations.

Electronic energy (a. u.) @ CPCM(MeCy)-ωB97M-V/def2-QZVP: –8437.60774558447
Thermochemical corrections (a. u.) @ r2SCAN-3c: 2.122058454168
Imaginary frequencies: one (–88.79 cm^{-1})
Gibbs Free Energy (a. u.): –8435.48568713030

273

C	-2.80089910737672	5.35388347270881	-1.06617163029219
C	-1.97632409313250	5.77060710301460	-1.83918260231199
C	-1.03110319953445	6.30809868371984	-2.75098716111969
O	-1.39630768903969	6.54753656669367	-3.99334022545348
O	0.12105901900423	6.58482620775784	-2.37650000231449
C	-2.71477049729550	6.10853408114094	-4.43054443617677
H	-2.79454246517161	5.02774590662301	-4.29807497087397
H	-2.75407075787273	6.36861955654862	-5.48834717283279
H	-3.48369797214810	6.64036010514689	-3.86307403017814
C	-4.59680828312641	7.80624840349051	-0.09945043651381
C	-6.00642775423406	7.37508598668895	-0.35422595733527
H	-6.71190484014099	7.90089784851649	0.30157591913225

H	-6.14379297266152	6.30164699587260	-0.16579970263842
H	-6.29506411698418	7.57066551638322	-1.38941248065068
C	-3.79343383245216	8.34182814254649	-1.03076790769542
O	-4.17773362545317	8.47863051838485	-2.31942064017706
O	-2.51790456182244	8.75434329844360	-0.84262543969091
Si	-3.92108527067142	9.88188844988711	-3.23959639848310
C	-5.17443242514311	9.70968769482648	-4.61370106866076
H	-6.19601838671153	9.69341311403774	-4.21957556516609
H	-5.01625204550177	8.78190138952567	-5.17487238443962
H	-5.09758732923603	10.54199947248862	-5.32216252291889
C	-2.18604810541813	9.93314193830716	-3.92917401779283
H	-1.44286052914724	9.92171610904498	-3.12629293285247
H	-2.03709384302644	10.84529584232112	-4.51939365346173
H	-1.98311342204873	9.07837405817773	-4.58267169000753
C	-4.26930004138660	11.36594394697538	-2.15613800230300
H	-3.49740370327963	11.48782525498565	-1.38911567125364
H	-5.23572198081703	11.26464424260216	-1.64995806902333
H	-4.29579324979734	12.28490487886371	-2.75258054164822
Si	-1.66371079809157	9.01215982546599	0.59625531989992
C	-1.33484194056585	7.40199851903661	1.48476161612415
H	-0.84080714419918	6.69269320861028	0.81228440252480
H	-2.23461548219854	6.92051445993725	1.87932983280576
H	-0.65867681252734	7.58561067199416	2.32874631467002
C	-2.59874113910539	10.23151123575307	1.66553786881877
H	-2.03504437786757	10.43081777400483	2.58446210187112
H	-3.58849294151404	9.86384121092802	1.95231702387584
H	-2.73428052263197	11.18631758196236	1.14580212081168
C	-0.05543722866438	9.74027063237181	-0.00423388934257
H	0.56480848797162	10.04831325209789	0.84477376773819
H	-0.21868809154731	10.61888921945742	-0.63798708727711
H	0.51023190594750	8.99933722922674	-0.58045252300062
H	-4.19482330274395	7.68210081587134	0.90090305505981
Si	1.89904277529427	6.85873347109297	-3.34016407562748
C	2.69841491214379	6.47043746558951	-1.69921956939824
H	2.18233991762202	7.02169977077483	-0.90555699978639
H	3.75870536120851	6.72269820328237	-1.67279372637487
H	2.59665735304585	5.40220318533555	-1.47510120381656
C	1.56034986201543	5.58401937456826	-4.65069079833798
H	0.52961106089380	5.23447436851911	-4.68429290471226
H	2.17774230676442	4.69566482306569	-4.49964433699694
H	1.82674961083911	5.99721798562707	-5.62538098417521
C	1.54066562955148	8.65184420214151	-3.72958699790159
H	2.46248748414054	9.20531186024076	-3.92456144862847
H	1.01350179372806	9.13263815982170	-2.90008289881096

H	0.90365884154902	8.72418362786161	-4.61802952916983
H	-3.50837986598720	4.94736038584324	-0.38325815062629
C	0.97544335814811	5.39712691296186	-9.17253178756738
C	-0.44192676124286	5.50641718156936	-9.75986608379660
H	-0.61097537734399	6.53988996414712	-10.08993413229943
C	-1.50579798059934	5.07614013143410	-8.76436608738086
H	-2.51168019037895	5.28310335093199	-9.15369083813758
H	-1.39819506122755	5.66050398242098	-7.83889014779528
C	-1.40084953417090	3.58320918234305	-8.41564874178538
C	-2.27662822425160	3.32737820593243	-7.17456896977613
H	-3.27212952119746	3.76331842706128	-7.32928809956103
H	-1.82420742316979	3.77229095856039	-6.28215480172807
H	-2.41503748153021	2.26096974847526	-6.97441792516182
C	-1.92446138294553	2.72087124507900	-9.57870327971441
H	-2.97613904673920	2.95485966765082	-9.78557541397324
H	-1.34720068689235	2.87792019743999	-10.49492279741082
H	-1.85648811586800	1.65730721234945	-9.32745445437808
C	0.05442929097731	3.22763497684709	-8.11358332906401
C	0.32470737820048	1.99741890798824	-7.50987224422602
H	-0.49819080264536	1.32112471042242	-7.29920993717233
C	1.60436915693970	1.61405410781643	-7.11000147295283
C	2.64634639225141	2.53263445829966	-7.28129543047710
C	2.39583269797971	3.73612830215593	-7.91545599487592
H	3.22524206269525	4.42308875049047	-8.04076985679869
H	3.65467997953640	2.30037186838625	-6.95881627659249
C	1.78515555167305	0.26420231165805	-6.53779860437442
C	2.58481820740502	0.01055402006085	-5.39657594634081
O	3.31030065740578	1.07520555337953	-4.86075804252514
P	2.68759750965293	1.75436653001217	-3.53314565791492
N	3.80385665456869	2.69229403735867	-2.94693463260600
P	5.10787173747744	3.52218977192595	-3.15576167524622
N	4.97735803273687	5.10926359688278	-3.37647458032403
S	4.75265572519277	5.97727530822081	-4.65555900114259
O	3.85039303325140	7.13554543420964	-4.30343786958169
C	6.41134989953090	6.86406144159154	-4.89944814141475
F	6.36310213514623	7.61570220823118	-6.00429957895513
F	6.70258159858168	7.64884210367092	-3.85415122654742
F	7.37535657995041	5.94624486872591	-5.03536194352361
O	4.50727854254233	5.29700703217941	-5.93274789820277
O	6.15365503317479	2.97808700422487	-4.28300982126652
C	6.90133536929160	1.82156856923548	-4.04007043957839
C	6.77881508161125	0.73389449325863	-4.94402738016681
C	6.17962239274671	0.87001429636721	-6.28848323007827
C	6.52654009371531	1.94511376091989	-7.10519703643529

C	6.15596744496880	2.01703489748253	-8.45313004607922
C	6.73417356059724	3.15954582150669	-9.29509844508004
C	6.40296099968758	2.96108578036082	-10.78520962065827
C	4.98658769222172	2.45513340912396	-10.99845732830238
H	4.73980565396651	2.43213456527256	-12.06825433385451
H	4.27064511177231	3.14101693034726	-10.52414634913012
C	4.78006224277025	1.05245147413584	-10.40752978286537
C	3.27106744281676	0.74802327771738	-10.44403672491134
H	2.90441442515648	0.85836363924394	-11.47177874453766
H	2.71713222190002	1.43932305572172	-9.80115035910672
H	3.04376670790976	-0.27287343365961	-10.12188913319958
C	5.51450158872052	-0.00830968702828	-11.25040194952700
H	5.15478662010312	0.01227248726321	-12.28647104475443
H	6.59686305704112	0.15170512522176	-11.25513592948599
H	5.33417057483450	-1.01021692903004	-10.84676857497764
H	6.55596275999702	3.91454990951418	-11.30756963030496
H	7.11587863622712	2.25215386101845	-11.22625043251906
C	6.17812041841910	4.51100109227480	-8.81702862002098
H	6.31628870956562	4.64775590044712	-7.74104411216016
H	5.10629030287808	4.59172033829098	-9.01771390331902
H	6.68583579491154	5.33128576638572	-9.33864954028151
C	8.26840588402611	3.18442338388021	-9.15479572451579
H	8.58869716385366	3.46959596395361	-8.14825138852453
H	8.68855779683167	3.91588646700370	-9.85569285139160
H	8.69558645861790	2.20221496476469	-9.38441102358704
C	5.31673705352815	1.01722135875237	-8.97760403698416
C	4.97482050053834	-0.06202141134495	-8.15213382490481
H	4.33956366317383	-0.85235266578119	-8.54141106045311
H	7.15409146707744	2.72723949220089	-6.68824616778530
C	5.41364012845781	-0.15656670532024	-6.84445518386494
H	5.13566373982174	-1.01101634863557	-6.23619480622708
C	7.41141789791935	-0.44259457196584	-4.59759220776270
H	7.34993391789279	-1.29128147430728	-5.27398619696944
C	8.18354562480091	-0.56491532142704	-3.42339623005916
C	8.41363392268567	0.58996734499029	-2.61606588678427
C	9.22038036364549	0.46468276894238	-1.46023551335174
H	9.37684039687119	1.33422339968736	-0.82963671388118
C	9.78796886403325	-0.74282239531240	-1.13035510552581
C	9.58753206841962	-1.87611394516921	-1.94549138438590
H	10.05191499830637	-2.82132701666777	-1.67909892711116
H	10.39632395058952	-0.82489067416678	-0.23432250829239
C	8.80212086214473	-1.78661349800433	-3.06777338771248
H	8.62899555997839	-2.65633319146993	-3.69489913848097
C	7.79723179018487	1.82058814011264	-2.98832592134311

C	8.16524088533899	3.09561629618577	-2.33264974724877
C	7.20486987573434	3.86788551703222	-1.72075718442971
C	7.47848761836266	5.10281567615802	-1.08280440606775
C	6.46309859388226	5.87874088456015	-0.34213483720164
C	6.35492473059842	7.25161148508908	-0.54723192070927
H	6.94174584946511	7.69127020450613	-1.34602472091869
C	5.52129838372514	8.06898352716708	0.22101156682207
C	5.44197628127955	9.55615920110291	-0.12779634897052
C	6.82693241560049	10.14165027847636	-0.45131106078618
H	6.74716256807800	11.23050971499104	-0.55027751942052
H	7.23496594016779	9.76164167563224	-1.39267434381169
H	7.54250716460302	9.92180980546268	0.34854609472952
C	4.53936044547025	9.72053178908403	-1.36786519229745
H	4.48882326318657	10.77574034400729	-1.66395119646767
H	4.93088145789612	9.14323468650730	-2.21211929608362
H	3.52180722307295	9.36483368763761	-1.17891284635401
C	4.86777225884936	10.34668780456110	1.05832600127242
H	4.64344308225675	11.36905871735645	0.72587090975051
H	5.63885928202220	10.43645156147433	1.83572783167184
C	3.62345963874621	9.69576600114413	1.63462796469675
H	3.19305672557856	10.32390092512660	2.42653072997858
H	2.85952006569156	9.61757661927925	0.85073344260752
C	3.89005233451723	8.29482887554568	2.20686978146811
C	4.62667320538813	8.40299147485021	3.55677286075602
H	5.59829535984351	8.89447162830032	3.44809830319100
H	4.02540347288235	8.97444922957994	4.27455226958090
H	4.80696983815457	7.40793538956794	3.97651335704530
C	2.52609468319435	7.62809554664595	2.44639757605336
H	2.01637202729643	7.42453191493091	1.49884697861557
H	2.60412693553609	6.68431659468723	2.99358369449677
H	1.89420812323831	8.30016640781882	3.04078564575864
C	4.76081421716472	7.48107528627352	1.24804504538980
C	4.86524197946520	6.09647272395720	1.43975381314216
H	4.30050313677113	5.62895354365199	2.24139497506124
C	5.68482826295538	5.29754940534215	0.66067360751217
H	5.76107194856798	4.23224143665666	0.85767806771840
C	8.77847288838777	5.56033259808092	-1.13660504440140
C	9.80006651422793	4.85042017283837	-1.80127469370972
C	11.11880207549517	5.36095365285386	-1.87405676571936
C	12.10034427579125	4.67981422814050	-2.54863078863584
C	11.79733193530097	3.46093367004673	-3.18918628838776
C	10.53114726230588	2.93078709406500	-3.12650613810297
H	10.31084028730332	1.99635398963017	-3.63068298584559
H	12.57122059745452	2.93757759092696	-3.74298642998317

H	13.10737027102874	5.08278640622946	-2.60220035008319
H	11.33581741040614	6.31093303652530	-1.39214403658904
H	9.02691665739965	6.49356042491351	-0.63707869410960
C	9.50185111327515	3.59545410451417	-2.41642954205265
O	5.89243117996188	3.38868226426157	-1.74439192153327
N	1.27378377046913	2.38607472551203	-3.91535422061848
S	-0.02863521615281	2.56657232485280	-3.02784617910912
O	0.17377682041397	2.59600346586407	-1.56954163902700
O	-0.89337470804210	3.60120086379943	-3.63336776340544
C	-1.02402055607747	0.98052133577056	-3.35546626032628
F	-1.23296988169047	0.82362600193375	-4.67200124708824
F	-2.21626182375169	1.07704295846324	-2.74243639740349
F	-0.39210780691528	-0.10302123758019	-2.89157477714032
O	2.39683983632444	0.48049969935735	-2.58056667070558
C	3.32571598602428	-0.54641217053337	-2.48523599260425
C	3.91767824709613	-0.73230705195489	-1.21291710459025
C	3.40739878147638	0.13714040929321	-0.12240106119205
C	3.97410933244092	1.38007215134110	0.14894837263251
C	3.28150185848436	2.27760625515381	0.94340717286685
H	3.70206528054580	3.26769520438478	1.09049000486922
C	2.02946568244123	1.97973959638066	1.49456835880891
C	1.27697813546956	3.08952381754068	2.22792938716045
C	1.23607874967883	4.36351445156740	1.36594235933313
H	2.22861627509082	4.79088587993960	1.19843667740952
H	0.63337278566694	5.12456646160116	1.87661020737106
H	0.78829083680317	4.15262193673626	0.38909261070761
C	1.98952025689037	3.40465504701046	3.55620253504541
H	3.02844338146570	3.70339407928932	3.37795388753056
H	1.48131705179774	4.22586545731938	4.07729993657650
H	2.01196745129402	2.53463188207746	4.22025254819521
C	-0.17817521674040	2.66835482732678	2.49154233634069
H	-0.62194671424614	3.36413585798620	3.21665463330159
H	-0.74840597134939	2.77938544422196	1.55981721942580
C	-0.28398232223991	1.23580105142642	2.98515673192964
H	0.32181746759148	1.11039047427301	3.89323613285528
H	-1.31844648541000	0.99781178666943	3.26661947176737
C	0.18716611007039	0.22603230961799	1.92654936525117
C	-0.86788471835343	0.08961767138821	0.81127044661018
H	-1.82971096963961	-0.22282092045889	1.23693857423205
H	-0.56099864437177	-0.66209734548808	0.07701829673910
H	-1.00508602756877	1.02764277862247	0.26638687547604
C	0.35713007928149	-1.13628280916493	2.62136959595176
H	1.16236456298333	-1.09689422794030	3.36312253835082
H	-0.57283476150563	-1.40690902422798	3.13608274326341

H	0.58650100689685	-1.93762645284417	1.91291385028405
C	1.51073449953737	0.68697480923178	1.31640353061972
H	4.90891329114959	1.66628936228296	-0.32106308664335
C	2.20572084240329	-0.20058770582302	0.48873561518491
H	1.77258484567515	-1.17139152859713	0.26236622385465
C	4.83022495185151	-1.74672429909722	-1.07009175379074
H	5.29284760560116	-1.92186092879765	-0.10205197080596
C	5.17028669989047	-2.58195741169636	-2.16201477622084
C	4.52320523357459	-2.40426723046529	-3.42744225072295
C	4.90482026615494	-3.24845958306347	-4.49880218245114
H	4.43807176710322	-3.11505407508714	-5.46906516442950
C	5.83795365229341	-4.24210795780006	-4.32199380294221
C	6.43967182179136	-4.44392436652664	-3.06396453313875
H	7.16775112226458	-5.23950988230119	-2.93312498745957
H	6.10944435768866	-4.87911843577055	-5.15900820912917
C	6.11744227759559	-3.62225547565989	-2.01254781975169
H	6.59624923788507	-3.75036461146213	-1.04513214075009
C	3.53198240116625	-1.38106504478330	-3.56678338318420
C	2.69683094078137	-1.22348316827145	-4.78280405705067
C	1.91269522304218	-2.30531392405523	-5.29559407081194
C	1.15662464065868	-2.09593961418591	-6.49126165075033
C	0.40832566121971	-3.16933719104387	-7.03418265703868
H	-0.14835418916437	-3.00083415363027	-7.95266961788974
C	0.36280142355621	-4.38698009994030	-6.40383281752902
C	1.05386610665839	-4.57399991928632	-5.18925797534396
H	0.98329168011699	-5.52557835636762	-4.67051495574926
H	-0.22188972145930	-5.19968113164327	-6.82467544414033
C	1.80850039535981	-3.56076744663491	-4.64855715069992
H	2.32469891785426	-3.71757627466920	-3.70821744727103
C	1.12466673725246	-0.81723193836469	-7.08435851504092
H	0.53954881097020	-0.67566536748960	-7.98974811603983
H	-0.50844126110732	4.88066592531451	-10.65956598617602
C	1.26443566821174	6.61307035364380	-8.27418205671792
H	0.54211442998013	6.68955326087448	-7.45481394561184
H	2.26407254396273	6.54749926299460	-7.83137525981647
H	1.21543915998946	7.53618533829411	-8.86463275226562
C	1.97444484212993	5.40963518234346	-10.34477146476855
H	1.78624205682931	6.28210360382228	-10.98242181100123
H	3.01159793588097	5.46719148438677	-10.00057659112496
H	1.86735352626069	4.50588583719151	-10.95473088651695
C	1.12523672673376	4.09677917366668	-8.38119813979208

(*E*)-TMS-4p

33 conformers were generated by GOAT, all of which were used geometry optimizations and frequency calculations.

Electronic energy (a. u.) @ CPCM(MeCy)-ωB97M-V/def2-QZVP: –1391.10718837882
Thermochemical corrections (a. u.) @ r2SCAN-3c: 0.336023070569
Imaginary frequencies: zero
Gibbs Free Energy (a. u.): –1390.77116530825

45

C	-1.39970511807738	6.55613730101785	0.74372479754228
C	-1.64219856570143	6.16848167461180	-0.51828750040777
C	-2.80930448279954	5.30212324112182	-0.83004460028679
O	-3.39108969473154	5.69431766372154	-1.99506946893932
O	-3.19182706151128	4.33829112642664	-0.20288596169528
C	-4.50990967377173	4.88815656448285	-2.41599134585567
H	-4.88101745245662	5.35588601815665	-3.32885327926863
H	-4.18738082093933	3.86105507157725	-2.61057532846504
H	-5.28428807835944	4.87845744106580	-1.64441911608333
H	-0.52383886648922	7.17858878068790	0.93706996841308
C	-2.25345217111708	6.30887036709541	1.96540721460178
C	-1.41356909921549	6.24719735914473	3.23650511372014
H	-0.69761989857811	5.42220819284453	3.17259276758633
H	-2.04893214443411	6.09191749133519	4.11244636906850
H	-0.86484776974271	7.18159685712026	3.38638439638202
C	-3.27255242163089	7.44360534211317	2.02443263224585
O	-4.18384514643042	7.34700665276892	1.04288251800646
O	-3.25418193290726	8.34165931901242	2.83817578127716
Si	-5.31830906467644	8.59282778769282	0.72545374293469
C	-6.18808709617589	7.93822827023028	-0.79196824094249
H	-6.93949624264421	8.64858807234306	-1.15425188180838
H	-6.69621402653546	6.99173755070617	-0.57747870896833
H	-5.46756659401650	7.76293268851075	-1.59879272270963
C	-4.38909566374027	10.16672585725069	0.34516833455912
H	-3.86796615959966	10.54076752320326	1.23115755530236
H	-5.07645883884102	10.94545232740460	-0.00502331073674
H	-3.64938176207657	9.99529006497127	-0.44513825874662
C	-6.48313032965146	8.76602673963677	2.17262419778481
H	-5.95480658256358	9.14203405803306	3.05352967823004
H	-6.93466169024310	7.80122362856153	2.42871086989403
H	-7.29595652938993	9.46160048789879	1.93326693457352
Si	-0.52470385783102	6.71985350564869	-1.94635741412173
C	-0.20993938842608	5.24878639093803	-3.07297442887324
H	0.23582537545680	4.41456004422062	-2.52006774594822

H	0.47761055934953	5.52011979570728	-3.88214371186618
H	-1.13965438945854	4.89318730776977	-3.52939485947922
C	1.10332256022604	7.32643662286127	-1.22523226837921
H	1.79756104163278	7.56827422663341	-2.03844123200021
H	1.57994542380000	6.56594750497249	-0.59709219057232
H	0.97844485075983	8.23486073331854	-0.62587052487844
C	-1.33634055927982	8.12028891271671	-2.90281569284678
H	-2.29222262513073	7.80232140309759	-3.32901477172701
H	-0.68859944395415	8.45729461538217	-3.72074215591135
H	-1.52428995541069	8.97882571706264	-2.24850891509569
H	-2.81669855355384	5.38233168613120	1.81502276308083

(*Z*)-TMS-4p

45 conformers were generated by GOAT, all of which were used geometry optimizations and frequency calculations.

Electronic energy (a. u.) @ CPCM(MeCy)-ωB97M-V/def2-QZVP: –1391.10463050067
Thermochemical corrections (a. u.) @ r2SCAN-3c: 0.336833655905
Imaginary frequencies: zero
Gibbs Free Energy (a. u.): –1390.76779684477

45

C	-2.22294447357322	5.80246256011610	-0.85084947584626
C	-1.69245446875250	6.96739141901307	-1.26370395947708
C	-0.43511559569715	7.41888524703090	-0.60709349263387
O	0.30777051216937	6.41733367174126	-0.07911487235026
O	-0.09224659652478	8.58569247730278	-0.54888997229878
C	1.50679420916095	6.84369963030277	0.59749140598001
H	1.98113914174923	5.92782905525705	0.95171661561754
H	2.16563435249226	7.37832904882069	-0.09266049515001
H	1.26168301207551	7.50139800654732	1.43637590475379
H	-1.70045807124568	5.23405815868511	-0.07910726176596
C	-3.51974823020978	5.16901254620415	-1.27954305429327
C	-3.37642699051336	3.64934663288437	-1.38029602775540
H	-4.32175963252024	3.19114130677027	-1.68589628577543
H	-3.09356885016564	3.22755541883560	-0.41218381715150
H	-2.60946693099093	3.39297164599529	-2.11723116013479
C	-4.54631805452186	5.54849357434424	-0.21419190182823
O	-5.20289661723332	6.67858813276957	-0.52022538078919
O	-4.72177829805268	4.92283108181234	0.80884780684035
Si	-6.34028818193518	7.38448557140777	0.56336720537770
C	-7.70991078579129	6.16763114626251	0.91937429775770
H	-7.34472412576379	5.32690812576656	1.51527255691943
H	-8.13331701603155	5.77164806594483	-0.01045228815111

H	-8.52104035041136	6.65852581307500	1.46987911237212
C	-6.97645473815223	8.83411853835152	-0.42661729230015
H	-7.43329641215297	8.49757470393033	-1.36384718416799
H	-6.17598012567854	9.53835869021543	-0.67595499678692
H	-7.73959624145232	9.38130533089222	0.13847961441893
C	-5.42945095114307	7.92817145830497	2.09778029051181
H	-5.05024238943967	7.06402545028198	2.65160201018708
H	-6.09294934459134	8.49603864385020	2.76023543040997
H	-4.58088361520881	8.57330775945182	1.84371270267818
Si	-2.37030733452372	8.27202330102181	-2.48491901534029
C	-0.96381120364900	8.79917414286078	-3.61609711306122
H	-0.14278082529304	9.24836261291216	-3.05148423989724
H	-1.32532669155019	9.53582480955471	-4.34315086041110
H	-0.56966572796704	7.94584143282098	-4.17998517745252
C	-3.02435948112944	9.72062012897368	-1.48404028938253
H	-3.76843817522701	9.37423892837982	-0.75872275347991
H	-3.50543603902174	10.45940131273265	-2.13607179206673
H	-2.21332298059491	10.20864295296289	-0.93677340117165
C	-3.75585475289012	7.64196678318384	-3.59660120133911
H	-3.96969518393057	8.43373340174296	-4.32591195041142
H	-4.68187163638714	7.44120268365444	-3.04945265568172
H	-3.47580802313359	6.74952390068899	-4.16718466454588
H	-3.85048609781639	5.59448471545063	-2.22757093867834

13. References

(1) Mandrelli, F.; Blond, A.; James, T.; Kim, H.; List, B., Deracemizing alpha-Branched Carboxylic Acids by Catalytic Asymmetric Protonation of Bis-Silyl Ketene Acetals with Water or Methanol. *Angew. Chem. Int. Ed.* **2019,** *58* (33), 11479–11482.

(2) Zhu, C.; Mandrelli, F.; Zhou, H.; Maji, R.; List, B., Catalytic Asymmetric Synthesis of Unprotected beta(2)-Amino Acids. *J. Am. Chem. Soc.* **2021,** *143* (9), 3312-3317.

(3) Gatzenmeier, T.; Kaib, P. S. J.; Lingnau, J. B.; Goddard, R.; List, B., The Catalytic Asymmetric Mukaiyama-Michael Reaction of Silyl Ketene Acetals with alpha,beta-Unsaturated Methyl Esters. *Angew. Chem. Int. Ed.* **2018,** *57* (9), 2464-2468.

(4) Lee, S.; Kaib, P. S. J.; List, B., Asymmetric Catalysis via Cyclic, Aliphatic Oxocarbenium Ions. *J. Am. Chem. Soc.* **2017,** *139* (6), 2156-2159.

(5) Ouyang, J.; Maji, R.; Leutzsch, M.; Mitschke, B.; List, B., Design of an Organocatalytic Asymmetric (4 + 3) Cycloaddition of 2-Indolylalcohols with Dienolsilanes. *J. Am. Chem. Soc.* **2022,** *144* (19), 8460-8466.

(6) Schwengers, S. A.; De, C. K.; Grossmann, O.; Grimm, J. A. A.; Sadlowski, N. R.; Gerosa, G. G.; List, B., Unified Approach to Imidodiphosphate-Type Bronsted Acids with Tunable Confinement and Acidity. *J. Am. Chem. Soc.* **2021,** *143* (36), 14835-14844.

(7) Chung, C. K.; Bulger, P. G.; Kosjek, B.; Belyk, K. M.; Rivera, N.; Scott, M. E.; Humphrey, G. R.; Limanto, J.; Bachert, D. C.; Emerson, K. M., Process Development of C–N Cross-Coupling and Enantioselective Biocatalytic Reactions for the Asymmetric Synthesis of Niraparib. *Org. Process Res. Dev.* **2014,** *18* (1), 215-227.

(8) Neese, F. Software Update: The ORCA Program System—Version 5.0. *Wiley Interdiscip. Rev. Comput. Mol. Sci.* **2022**, *12* (5), e1606.

(9) Bannwarth, C.; Ehlert, S.; Grimme, S. GFN2-xTB—An Accurate and Broadly Parametrized Self-Consistent Tight-Binding Quantum Chemical Method with Multipole Electrostatics and Density-Dependent Dispersion Contributions. *J. Chem. Theory Comput.* **2019**, *15* (3), 1652–1671.

(10) Souza, B. de. GOAT: A Global Optimization Algorithm for Molecules and Atomic Clusters. *Angew. Chem. Int. Ed.* **2025**, *64* (18), e202500393.

(11) Grimme, S.; Hansen, A.; Ehlert, S.; Mewes, J.-M. r2SCAN-3c: A "Swiss Army Knife" Composite Electronic-Structure Method. *J. Chem. Phys.* **2021**, *154* (6), 064103.

(12) Caldeweyher, E.; Bannwarth, C.; Grimme, S. Extension of the D3 Dispersion Coefficient Model. *J. Chem. Phys.* **2017**, *147* (3), 034112.

(13) Caldeweyher, E.; Ehlert, S.; Hansen, A.; Neugebauer, H.; Spicher, S.; Bannwarth, C.; Grimme, S. A Generally Applicable Atomic-Charge Dependent London Dispersion Correction. *J. Chem. Phys.* **2019**, *150* (15), 154122.

(14) Caldeweyher, E.; Mewes, J.-M.; Ehlert, S.; Grimme, S. Extension and Evaluation of the D4 London-Dispersion Model for Periodic Systems. *Phys. Chem. Chem. Phys.* **2020**, *22* (16), 8499–8512.

(15) Wittmann, L.; Gordiy, I.; Friede, M.; Helmich-Paris, B.; Grimme, S.; Hansen, A.; Bursch, M. Extension of the D3 and D4 London Dispersion Corrections to the Full Actinides Series. *Phys. Chem. Chem. Phys.* **2024**, *26* (32), 21379–21394.

(16) Eichkorn, K.; Treutler, O.; Öhm, H.; Häser, M.; Ahlrichs, R. Auxiliary Basis Sets to Approximate Coulomb Potentials. *Chem. Phys. Lett.* **1995**, *240* (4), 283–290.

(17) Neese, F. An Improvement of the Resolution of the Identity Approximation for the Formation of the Coulomb Matrix. *J. Comput. Chem.* **2003**, *24* (14), 1740–1747.

(18) Weigend, F. Accurate Coulomb-Fitting Basis Sets for H to Rn. *Phys. Chem. Chem. Phys.* **2006**, *8* (9), 1057–1065.

(19) Weigend, F.; Ahlrichs, R. Balanced Basis Sets of Split Valence, Triple Zeta Valence and Quadruple Zeta Valence Quality for H to Rn: Design and Assessment of Accuracy. *Phys. Chem. Chem. Phys.* **2005**, *7* (18), 3297–3305.
(20) Cossi, M.; Rega, N.; Scalmani, G.; Barone, V. Energies, Structures, and Electronic Properties of Molecules in Solution with the C-PCM Solvation Model. *J. Comput. Chem.* **2003**, *24* (6), 669–681.
(21) Marenich, A. V.; Cramer, C. J.; Truhlar, D. G. Universal Solvation Model Based on Solute Electron Density and on a Continuum Model of the Solvent Defined by the Bulk Dielectric Constant and Atomic Surface Tensions. *J. Phys. Chem. B* **2009**, *113* (18), 6378–6396.
(22) *otherm.py*. https://github.com/duartegroup/otherm.
(23) Bickelhaupt, F. M.; Houk, K. N. Analyzing Reaction Rates with the Distortion/Interaction-Activation Strain Model. *Angew. Chem. Int. Ed.* **2017**, *56* (34), 10070–10086.
(24) Mardirossian, N.; Head-Gordon, M. ωB97M-V: A Combinatorially Optimized, Range-Separated Hybrid, Meta-GGA Density Functional with VV10 Nonlocal Correlation. *J. Chem. Phys.* **2016**, *144* (21), 214110.
(25) Glendening, E. D.; Landis, C. R.; Weinhold, F. NBO 7.0: New Vistas in Localized and Delocalized Chemical Bonding Theory. *J. Comput. Chem.* **2019**, *40* (25), 2234–2241.
(26) Zhao, Y.; Truhlar, D. G. The M06 Suite of Density Functionals for Main Group Thermochemistry, Thermochemical Kinetics, Noncovalent Interactions, Excited States, and Transition Elements: Two New Functionals and Systematic Testing of Four M06-Class Functionals and 12 Other Functionals. *Theor. Chem. Acc.* **2007**, *120* (1–3), 215–241.
(27) Becke, A. D. Density-functional Thermochemistry. III. The Role of Exact Exchange. *J. Chem. Phys.* **1993**, *98* (7), 5648–5652.
(28) Lee, C.; Yang, W.; Parr, R. G. Development of the Colle-Salvetti Correlation-Energy Formula into a Functional of the Electron Density. *Phys. Rev. B* **1988**, *37* (2), 785–789.
(29) Emde, H.; Simchen, G. Reaktionen Der Trialkylsilyl-trifluormethansulfonate, II. Synthese von O-Alkyl-O-(Trialkylsilyl)Ketenacetalen Und 2-(Trialkylsilyl)Carbonsäureestern. *Liebigs Ann. Chem.* **1983**, No. 5, 816–834.
